# Iridium-Catalyzed Regio- and Enantioselective Reverse Prenylation of Tryptamines and Other 3‑Substituted Indoles

**DOI:** 10.1021/jacs.5c06364

**Published:** 2025-07-17

**Authors:** Leon Sander, Jonas M. Müller, Christian B. W. Stark

**Affiliations:** Fachbereich Chemie, Institut für Organische Chemie, University of Hamburg, Martin-Luther-King-Platz 6, Hamburg 20146, Germany

## Abstract

Prenylated indole alkaloids from bacteria, fungi, plants, and animals comprise a large structural diversity with a broad range of biological activities. A subclass of this alkaloid family are the *C*3a-reverse prenylated hexahydropyrrolo­[2,3-*b*]­indole (HPI) natural products that are in principle synthetically accessible via a metal-catalyzed allylic substitution. Here we report the first catalytic enantioselective reverse prenylation of achiral 3-substituted indoles that furnishes hexahydropyrrolo­[2,3-*b*]­indoles in a single step. The developed catalytic system utilizes a novel iridium–NHC–phosphoramidite catalyst and provides the *C*3-prenylated products with high yields and enantioselectivities as well as complete branched selectivity. This elaborate methodology closes a systematic gap in asymmetric allylic substitution chemistry and offers a convenient strategy for the synthesis of tryptamine-derived alkaloids as demonstrated in a short biomimetic total synthesis of (−)-flustramine A, a prototypical member of this class of natural products. Mechanistic investigations elucidate the catalyst’s structure and reveal a chloride-induced allyl complex isomerization, which is dependent on the hemilabile phosphoramidite-olefin Carreira-type ligand.

## Graphical abstract

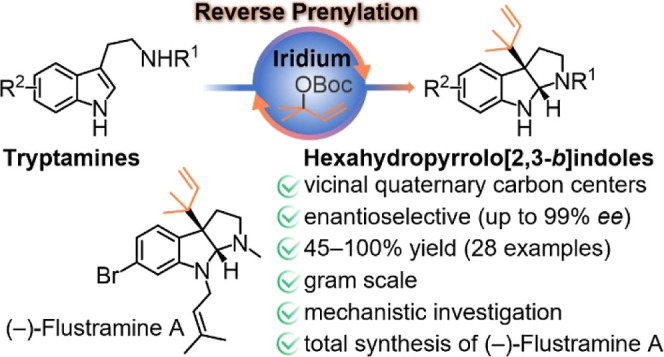

## Introduction

In living organisms introduction of prenyl units represents an omnipresent process in the biosynthesis of alkaloids, phenylpropanoids, polyketides, terpenoids, and proteins. These enzymatic transformations engender a vast structural diversity that is exploited by nature in numerous primary and secondary metabolic pathways as well as in cellular regulation.^1–4^ In the case of indole-alkaloids, prenylations are accomplished by prenyltransferases from the DMATS (dimethylallyltryptophan synthase) superfamily which comprises more than 40 enzymes from fungi and bacteria. These enzymes catalyze prenylations of tryptophan and tryptophan containing cyclic peptides as well as other indole derivatives.^1,5–11^ Chemoenzymatic syntheses of the *C* ^3a^-reverse prenylated hexahydropyrrolo­[2,3-*b*]­indoles rugulosovine A, amauromine and aszonalenin proved that their biosynthetic precursor is indeed a cyclic peptide.^12–14^ As exemplified for rugulosovine A, a cyclic dipeptide C3 prenyl transferase (CdpC3PT) catalyzes the regio- and stereoselective transfer of a prenyl-cation generated from dimethylallylpyrophosphate (DMAPP) to the 3-position of the indole. The resulting indolenine intermediate is subsequently attacked by the proximal piperazine-nitrogen and thereby forms the hexahydropyrrolo­[2,3-*b*]­indole (Scheme 1).^15^ *C* ^3a^-Reverse prenylated hexahydropyrrolo­[2,3-*b*]­indoles show a broad range of biological activities and feature a vast structural diversity that arises from different cyclic peptides and diverse substitution and oxygenation patterns as well as differing stereochemistry.^7,8,16–18^ Flustramines A and C from the marine bryozoan do not contain a cyclic peptide and are formally derived from tryptamines (Figure 1).^19,20^

**Scheme 1:**
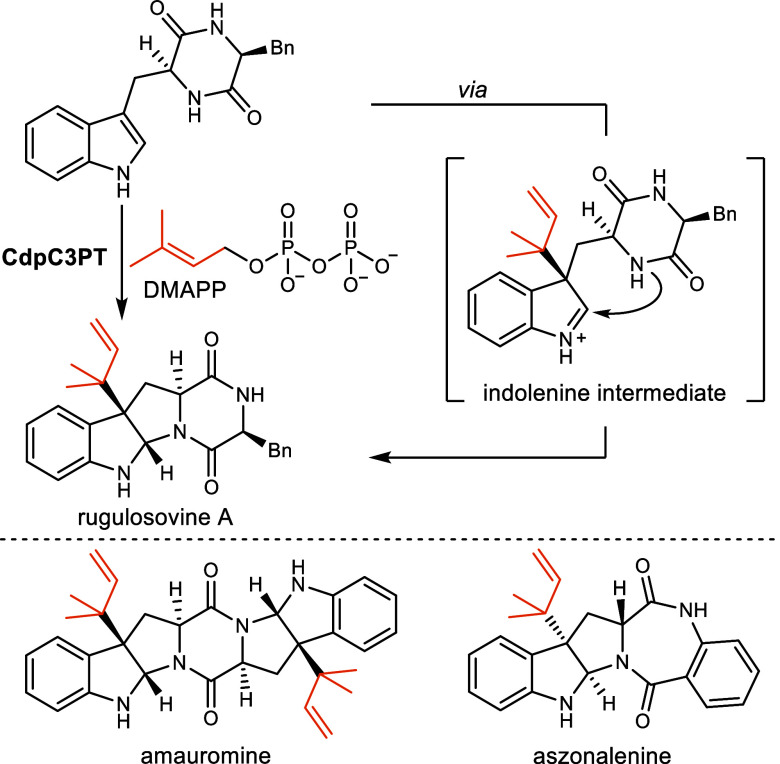
Biosynthesis and Chemoenzymatic Synthesis of Pyrrolo­[2,3-*b*]­Indole Natural Products

**Figure 1:**
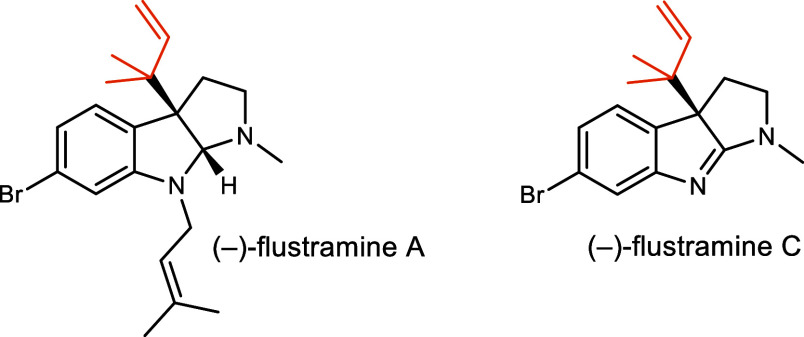
Representative flustramines from .

Early approaches to *C* ^3a^-quaternary pyrrolo­[2,3-*b*]­indoles commonly rely on the reductive cyclization of prefunctionalized 2-oxoindolines, a strategy that is often hampered by multistep syntheses.^21–26^ More advanced strategies employ cascade asymmetric dearomative cyclization reactions of indole derivatives, enabling the construction of the target frameworks in fewer synthetic steps.^27–29^ Among these, the catalytic allylic substitution has attracted considerable attention due to its versatility and reliability in the synthesis of complex organic molecules.^30–32^ The application of this methodology would enable a biomimetic addition of a prenyl electrophile to tryptophans and tryptamines, thereby providing a convenient route to reverse prenylated pyrrolo­[2,3-*b*]­indoles. However, in addition to controlling facial selectivity, two separate issues of regiocontrol have to be considered: (1) The competing *C*-versus *N*-nucleophilicity of indoles. (2) The ambident nature of the prenyl complex, which can be attacked at either allyl terminus, leading to either linear or reverse prenylated products. The first challenge has been addressed through the use of boranes, which serve as temporary *N*-protecting groups while simultaneously activating the indole nucleus.^33–35^ Regioselective attack at the more substituted allyl terminus, resulting in the reverse prenylated product (also termed the branched product) can be achieved with iridium catalysts, which have proven particularly suitable for this purpose.^36–39^

Our research in this field culminated in the development of an iridium-catalyzed diastereodivergent reverse prenylation of tryptophans that achieves stereoselectivity by the simultaneous chiral induction of the substrate and the catalyst in combination with an achiral borane-additive.^40^ As shown by Carreira and co-workers, for a single substrate a comparable diastereoselectivity can be obtained solely by exploiting substrate control. Analogous transformations of achiral 3-substituted indoles could only be achieved racemically (Scheme 2, path b).^41^ Efforts to induce asymmetry in the transformation proved unsuccessful; enantiomeric excesses remained below 20%, and the structures of the employed chiral ligands and borane additives were not disclosed. Trost and co-workers presented a palladium-catalyzed reverse prenylation of 2-oxoindolines that reaches moderate to good branched-selectivity with enantiomeric excesses of 32–99%, however, the hexahydropyrrolo­[2,3-*b*]­indole has to be constructed from the prenylated intermediate by a reductive cyclization (Scheme 2, path d).^42,43^ To date, the enantioselective single-step synthesis of *C* ^3a^-prenylated hexahydropyrrolo­[2,3-*b*]­indoles from achiral tryptamines has only been accomplished for the linear prenylation, as reported by You and co-workers.^44^ This transformation was achieved via a palladium-catalyzed allylic substitution employing the phosphoramidite-olefin ligand (*R*)-Allylphos (Scheme 2, path a).

**Scheme 2:**
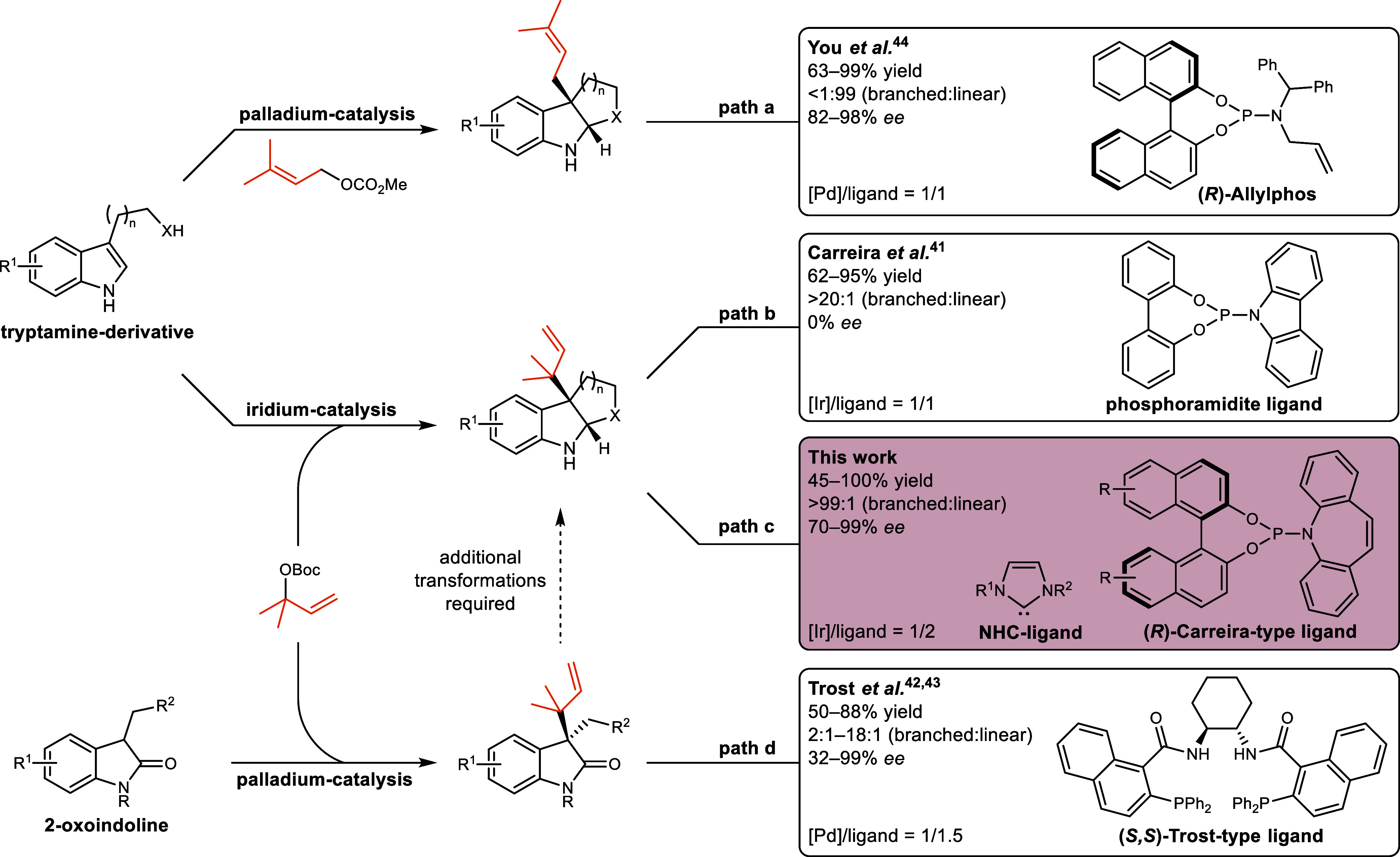
Allylic Substitution Strategies for the *C*3-Prenylation of *C*3-Substituted Indole-Derivatives

We here wish to report a direct method that permits the catalytic enantioselective reverse prenylation of achiral tryptamines and other 3-substituted indoles (Scheme 2, path c). Hexahydropyrrolo­[2,3-*b*]­indoles and related heterocycles are obtained in a single step as opposed to the established enantioselective processes for the reverse prenylation of indoles.^42,43,45,46^ We developed catalytic systems that make use of either an iridium-bis-phosphoramidite catalyst or a novel iridium–NHC–phosphoramidite catalyst. Both systems provide the *C* ^3a^-prenylated products with complete branched selectivity, however, the latter is superior in terms of yield and enantioselectivity in most cases. We elucidated the NHC-catalyst’s structure and implemented the methodology in a short asymmetric total synthesis of (−)-flustramine A.

## Results and Discussion

### Reaction Development with Iridium-Bis-Phosphoramidite-Catalyst

As a result of our preceding investigations, we have developed an operationally simple and high yielding general method for the reverse prenylation of tryptophans.^40^ The catalytic system utilizes triethylborane in combination with a substoichiometric amount of DBU for the activation of the indole and generates the prenyl-electrophile from Boc-protected prenol **1a**. A screening of different catalysts revealed that a complex derived from [Ir­(COD)­Cl]_2_ and the Carreira-ligand (*R*)-**L1** with a ratio of [Ir]:(*R*)-**L1** = 1:2 effectively catalyzed the reverse prenylation. When applying these conditions to Boc-tryptamine **2a**, as a model substrate for an achiral 3-substituted indole, we isolated the reverse prenylated product (−)-**3a** in a yield of 99% albeit with a negligible enantiomeric excess of 13% (Table 1, entry 1). Notably, neither the linear prenylated derivative nor a *N*-prenylated product could be detected. Regarding the assumption that the indole–borane complex represents the active nucleophile in the allylic substitution, we hypothesized that sterically demanding boranes might increase the enantioselectivity. For this purpose, we considered 9-BBN-derivatives as these have been employed successfully in our previous study as well as in protocols reported by Carreira and Ruchti^41^ and Trost and Quancard.^34^ Utilization of *B*-octyl-9-BBN led to an increased but still not synthetically useful enantioselectivity of −61% ee with concomitant preservation of the yield (Table 1, entry 2). Interestingly, in this case an inversion of the stereoselectivity in favor of (+)-**3a** was observed, which supports the assumption that the geometry of the achiral indole-borane-adduct determines the stereochemical outcome of the reaction. However, a further screening of reaction conditions by variation of the amidine base and the trialkylborane did not lead to an improved enantioselectivity (Table S1).

**Table 1:**
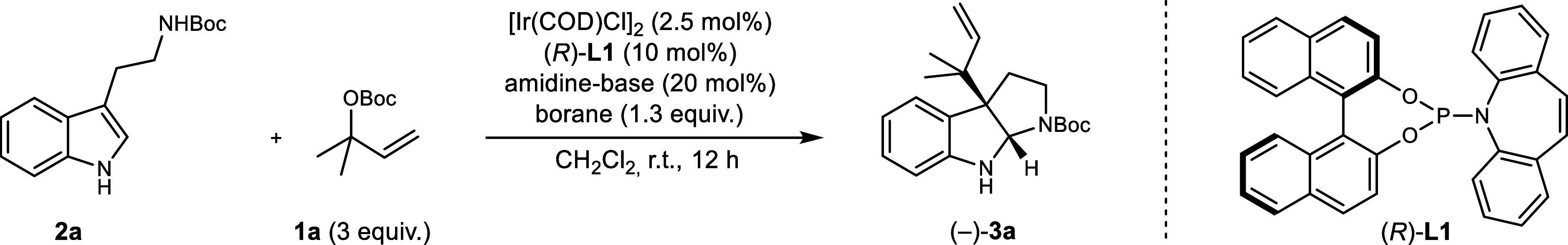
Evaluation of the Reaction Conditions (Selected) for the Enantiodivergent Reverse Prenylation with Iridium-Bis-Phosphoramidite Catalysts^a^

entry	amidine base	borane	yield^b^	ee^c^
1	DBU	BEt_3_	99%	13%
2	DBU	*B*-octyl-9-BBN	99%	–61%
3	DBU	BPh_3_	15%, 37%^d^	94%
4	TBD	BPh_3_	48%^d^	94%

a cf. Table S1 for a complete listing. All reactions were conducted according to the general procedure for the prenylation with iridium-bis-phosphoramidite catalysts (cf. Supporting Information).

b Isolated yield.

c Enantiomeric ratio determined by chiral HPLC.

d **1a** (10 equiv).

With regard to the iridium-catalyst and its phosphoramidite ligands we assumed that an aromatic borane engenders a mode of enantioselectivity that is partially determined by π-interactions which in turn could influence the facial selectivity of the prenylation reaction. Therefore, triphenylborane was applied as an additive which resulted in a yield of only 15% but an excellent stereoselectivity in favor of (−)-**3a** (94% ee; Table 1, entry 3). These findings showcase an astonishing case of enantiodivergent catalysis, the selectivity of which is steered through the variation of the achiral borane-additive. The low yield when using triphenylborane is attributed to a competing reaction in which the intermediate iridium-prenyl-complex undergoes β-hydride elimination to form isoprene with a rate that is comparatively high to that of the nucleophilic attack by the indole.

This undesired side reaction leads to a fast conversion of **1a**, which is also observable by the evolution of carbon dioxide formed by the fragmentation of the leaving group after oxidative addition. Hence the yield of (−)-**3a** could be raised to 37% by utilizing ten equivalents of **1a** and a further improvement of 48% was achieved by changing the amidine base to TBD (Table 1, entries 3 and 4). As with all trialkylboranes, the prenylations using triphenylborane did not yield any linear- or *N*-prenylated products.

At this stage we concluded that the major drawback of the thus far established catalytic system is the unproductive conversion of the allylic substrate **1a** via a β-hydride elimination resulting in the formation of isoprene. Another detrimental aspect is the behavior of the putative active catalyst that accommodates two phosphoramidite ligands of which one forms a chelate via phosphorus and the olefinic double bond whereas the other is solely acting as a monodentate ligand binding via phosphorus. A change to a bidentate coordination mode of the latter results in the formation of the highly symmetric complex Ir­(κ^2^-**L1**)_2_Cl that does not possess any catalytic activity.^47^ As illustrated by our screening of the reaction conditions, this inactivation of the catalyst is not necessarily adverse to the conversion of the indolic substrate but could become a problem with less reactive nucleophiles.

### Reaction Development with Iridium–NHC–Phosphoramidite-Catalysts

Regarding the limitations of the iridium-bis-phosphoramidite catalyst we envisioned that a novel ligand assembly around the iridium center needs to be designed to effectively achieve facial selectivity as well as reactivity for this challenging class of substrates.

Additionally, this new catalyst system needs to meet two major criteria: (a) an altered electronic structure that impedes β-hydride elimination to form isoprene from the prenyl complex and (b) no facile pathway for the inactivation of the catalyst by forming a stable chelate. We thus reasoned that an exchange of one of its ligands (*R*)-**L1** with an appropriate monodentate alternative can fulfill these requirements. A screening of simple trialkyl- and triphenylphosphines revealed that the heteromeric complexes suffered a scrambling of ligands that still resulted in the formation of Ir­(κ^2^-**L1**)_2_Cl. For this reason, we opted for imidazolium derived *N*-heterocyclic carbene (NHC) ligands as their strong σ-donor capability inhibits ligand dissociation, which in turn should result in a kinetically stable heteromeric iridium complex.^48,49^ Our strategy thus aims to induce asymmetry through a catalyst that combines a chiral phosphoramidite ligand with an achiral NHC-ligand, representing an unprecedented approach in catalyst design for iridium-catalyzed allylic substitutions. The use of NHC-ligands in this domain remains scarce, with the first example published by You et al. in 2016, who reported an enantioselective intramolecular *N*-allylation of indoles.^50^ The catalyst used in their study was composed of a chiral NHC-ligand as the sole source of stereocontrol, with a 1:1 iridium-to-ligand ratio and cyclooctadiene (COD) as the ancillary ligand. This concept was later extended to different substrate classes, and to the best of our knowledge, these examples represent the only reported applications of NHC-ligands in iridium-catalyzed allylic substitution reactions.^51–53^

At the outset of our investigation several precatalysts were prepared by reacting [Ir­(COD)­Cl]_2_ (0.5 equiv) with an imidazolium chloride **im-a**–**d** (1 equiv) which led to a halide exchange and thereby provided the iridates **precat. 1**–**4** (Scheme 3).

**Scheme 3:**
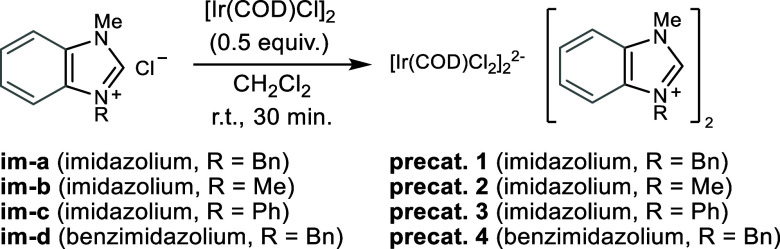
Preparation of Precatalysts

These were subsequently combined with a phosphoramidite ligand **L1**–**L6** (see the Supporting Information for chemical structures) and an amidine base. Addition of the latter generates the carbene from the respective imidazolium salts to ultimately afford the active iridium–NHC–phosphoramidite-complexes, which were used as received from this in situ procedure. Having established that triphenylborane effectuates a high enantioselectivity, a screening of precatalysts, ligands, amidine bases and solvents (Tables 2 and S2) led to the optimized reaction conditions. Notably, with **precat. 1**, **3** and **4** and MTBD (7-Methyl-1,5,7-triazabicyclo[4.4.0]­dec-5-en) as the amidine base a quantitative yield of **3a** was achieved with only five equivalents of the allyl cation precursor **1a**, which shows its reduced unproductive conversion (Table 2, entries 1, 3, 4). The enantiomeric excesses of (−)-**3a** were similar and ranged from 97.3%–97.5%. The developed protocol using **precat. 1** could also be carried out on a gram scale (1.00 g, 3.84 mmol **2a**) providing the chiral prenylated product (−)-**3a** with almost identical yield and enantioselectivity as compared to the reaction run on a small milligram scale (Table 2, entry 1^*d*^). From the optimization experiments, with respect to the carbene ligand it can be concluded that it has to possess a reasonable steric demand in order to achieve a high enantioselectivity (cf. Table 2, entry 2). Phosphoramidite ligands with either a partially saturated H8-BINOL backbone or a substituted aromatic BINOL were inferior to (*R*)-**L1**. The most profound influence on enantioselectivity was provoked by its olefinic coordination site as can be seen by comparison with (*R*)-**L5** that comprises the carbazole fragment instead of the iminostilbene. Another key finding was that the enantioselectivity could be more than doubled by switching the solvent from dichloromethane to benzene (Table S2). However, this approach did not apply when using *B*-octyl-9-BBN, as the product **3a** was generated almost racemically (Table 2, entry 5). In contrast, when dichloromethane was used, the enantiodivergent conversion to (+)-**3a** occurred with a selectivity (−60% ee) that was comparable to the bis-phosphoramidite system (Table 2, entry 5^f^).

**Table 2:**
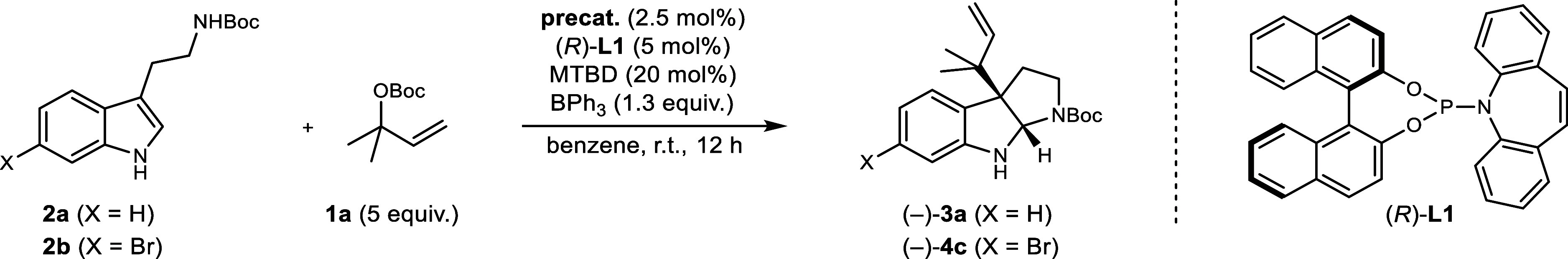
Evaluation of the Reaction Conditions (Selected) for the Enantioselective Reverse Prenylation with Iridium–NHC–Phosphoramidite Catalysts^a^

entry	substrate	catalyst	yield^b^	ee^c^
1	2a	precat. 1	100%, 96%^d^	97%, 97%^d^
2	2a	precat. 2	76%	88%
3	2a	precat. 3	100%	97%
4	2a	precat. 4	100%	98%
5^e^	2a	precat. 1	29%, 31%^f^	–5%, −60%^f^
6	2b	precat. 1	85%	93%

a cf. Table S2 for a complete listing. All reactions were conducted according to the general procedure for the prenylation with in situ generated iridium–NHC–phosphoramidite catalysts (cf. Supporting Information).

b Isolated yield.

c Enantiomeric ratio determined by chiral HPLC.

d Gram scale reaction with 1.00 g (3.84 mmol) **2a**.

e With *B*-octyl-9-BBN (1.3 equiv) instead of BPh_3_.

f With CH_2_Cl_2_ instead of benzene as the solvent.

### Substrate Scope

For the exploration of the substrate scope, we selected 6-bromo-Boc-tryptamine **2b** as the starting point as its reaction product **4c** would be a convenient precursor for the synthesis of the prominent family of flustramine alkaloids. Application of the optimized conditions with **precat. 1** and triphenylborane resulted in a slightly lower yield and enantioselectivity for (−)-**4c** (85%, 93% ee) in comparison to its nonbrominated analog (−)-**3a** (Table 2, entry 6). To solve this issue and restore high yields and selectivities, it appeared to us that an adaption of the borane is the most practical leverage point as its adduct with the indole represents the active nucleophile. Therefore, a screening of simple triarylboranes was conducted (Table 3). For (−)-**3a** and (−)-**4c** the enantioselectivities could be raised to 99% ee and 96% ee, respectively when using tris­(4-chlorophenyl)­borane. In both cases the yields were low, due to the unproductive conversion of the allylic substrate **1a** (Table 3, entries 1 and 2). For this reason, weaker Lewis acidic boranes were investigated as these could attenuate this pathway. Indeed, with either tri*m*-tolylborane or tri*p*-tolylborane the yields of **3a** and **4c** were up to quantitative (Table 3, entries 3–6). The highest enantioselectivity for (−)-**4c** was achieved with tris­(4-methoxyphenyl)­borane although this was accompanied by a reduced yield (Table 3, entry 8). A change of the precatalyst (entries 9–11) was not beneficial in this case but a doubling of the catalyst loading and the amount of amidine base restored the yield (91%, 97% ee, entry 8^e^). Inferring from our screenings, we concluded that the success of the developed catalysis is dependent upon a sophisticated adjustment of each reactant’s properties and as the universally applicable reaction conditions we deduced those from Table 2, entry 1.

**Table 3:**
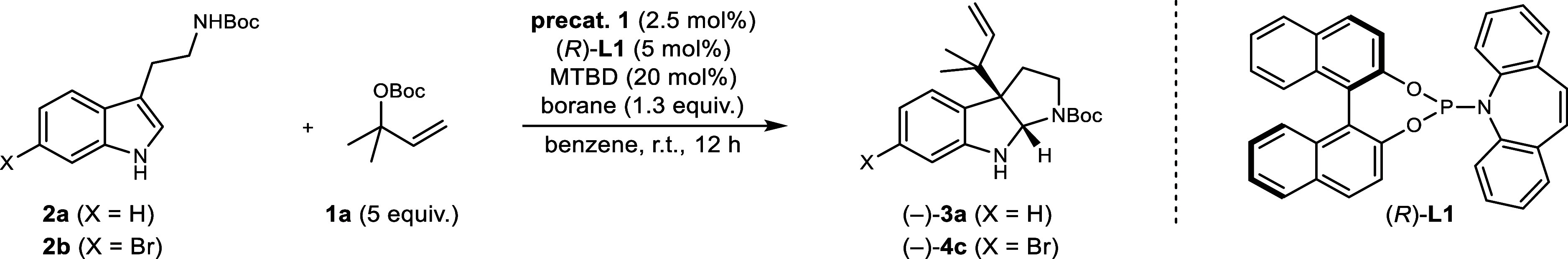
Screening of Triarylboranes and Further Substrate Specific Optimization^a^

entry	substrate	borane	yield^b^	ee^c^
1^d^	2a	B-(4-Cl-Ph)_3_	16%	99%
2^d^	2b	B-(4-Cl-Ph)_3_	18%	96%
3	2a	tri*m*-tolylborane	96%	85%
4	2b	tri*m*-tolylborane	100%	78%
5	2a	tri*p*-tolylborane	100%	97%
6	2b	tri*p*-tolylborane	100%	94%
7	2a	B-(4-MeO-Ph)_3_	88%	85%
8	2b	B-(4-MeO-Ph)_3_	62%, 91%^e^	97%
9^f^	2b	B-(4-MeO-Ph)_3_	42%	80%
10^g^	2b	B-(4-MeO-Ph)_3_	58%	96%
11^h^	2b	B-(4-MeO-Ph)_3_	17%	97%

a All reactions were conducted according to the general procedure for the prenylation with in situ generated iridium–NHC–phosphoramidite catalysts (cf. Supporting Information).

b Isolated yield.

c Determined by chiral HPLC.

d **1a** (10 equiv).

e **Precat. 1** (5.0 mol %), MTBD (40 mol %).

f **Precat. 2** (2.5 mol %).

g **Precat. 3** (2.5 mol %).

h **Precat. 4** (2.5 mol %).

An in-depth exploration of the substrate scope revealed that these conditions are successfully applicable to a variety of protected tryptamines (Figure 2A). With common carbamates and amides, the respective products **3b**–**h** were formed with excellent enantioselectivities (>96% ee). The yields exceeded 96% except for the Fmoc-derivative (−)-**3f** (67% yield), which can be attributed to a partial deprotection and subsequent *N*-prenylation. The further substrate scope included sulfonamides and the sterically demanding

**Figure 2:**
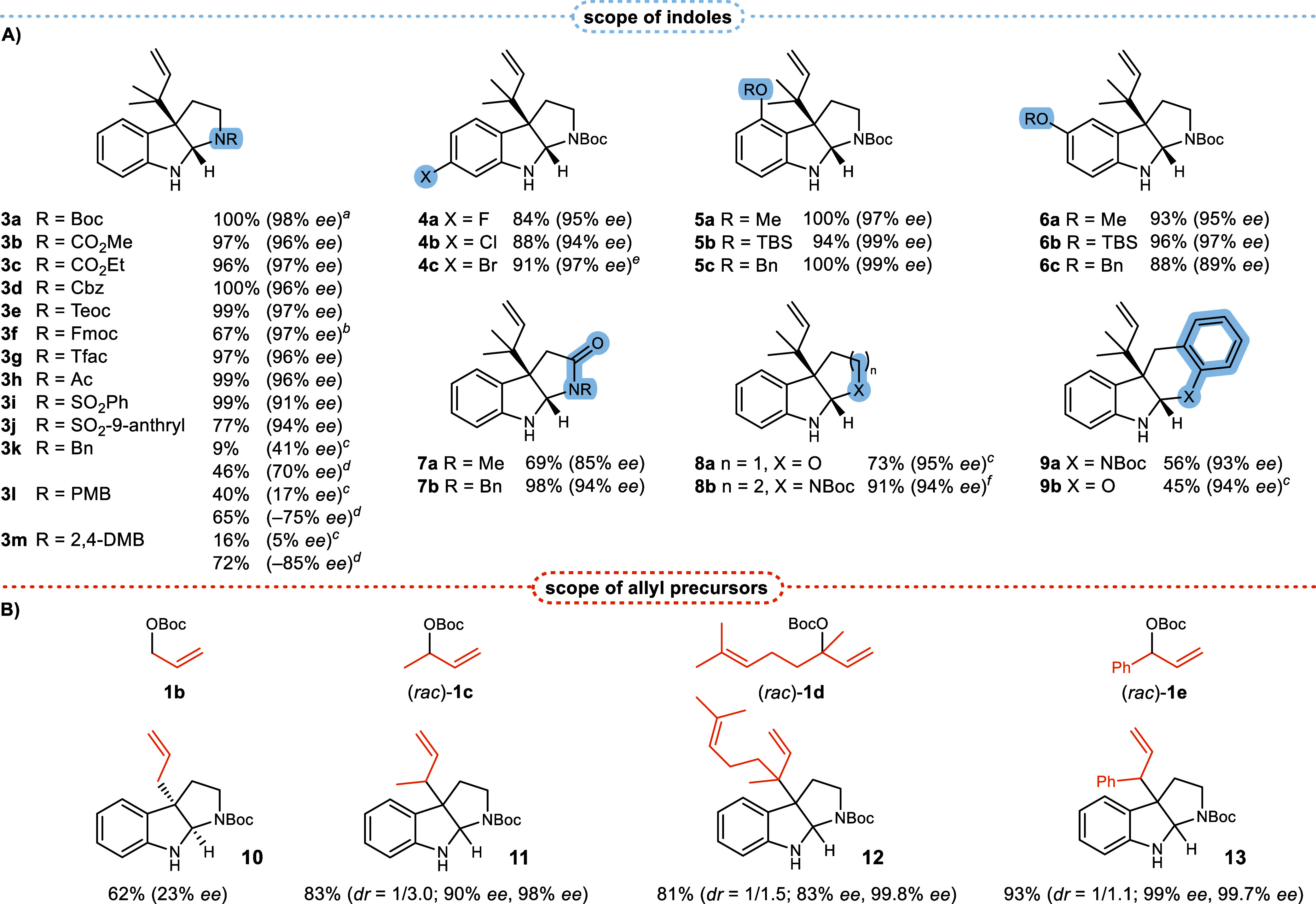
Substrate scope of the reverse prenylation. (**A)** Scope of indoles. (**B)** Scope of allylic substrates. Unless otherwise noted, all reactions were conducted according to the general procedure for the prenylation with in situ generated iridium–NHC–phosphoramidite catalysts (cf. Supporting Information) with **precat. 1** (2.5 mol %), (*R*)-**L1** (5 mol %), MTBD (20 mol %), BPh_3_ (1.3 equiv) and 5 equiv of the denoted allylic substrate (**1a** for section A). Given are the isolated yields. Enantiomeric ratios were determined by chiral HPLC. ^*a*^according to Table 2, entry 4. ^*b*^Reaction time 1 h. ^*c*^BPh_3_ (2.5 equiv). ^*d*^According to the general procedure for the prenylation with iridium-bis-phosphoramidite catalysts (cf. Supporting Information) with BEt_3_ (2.5 equiv). ^e^according to Table 3, entry 8^e^. ^*f*^Obtained after cyclization of the intermediate indolenine.

9-anthryl-derivative did not pose a challenge (products **3i**, **3j**). Benzyl-protected tryptamines were converted smoothly with an increased amount of triphenylborane (2.5 equiv), however the yields of **3k**–**m** were unsatisfactory and the enantioselectivities were minuscule to moderate. We conclude that substrates with a strongly Lewis basic tryptamine-nitrogen are unsuitable due to their ability to form a tight adduct with the borane. On the other hand, this property can be advantageous in certain cases: applying the iridium-bis-phosphoramidite catalyst in combination with triethylborane (2.5 equiv) resulted in improved yields for **3k**–**m** whereat the enantioselectivities were beyond the expected degree (cf. Supporting Information for the enantiomeric excesses of all products shown in Figure 2A when applying the iridium-bis-phosphoramidite catalyst with BEt_3_), which we attribute to the active nucleophile being an *N*,*N*′-bis-borylated tryptamine. Oxygenated tryptamines that are commonly encountered among natural products proved to be excellent substrates and the resulting pyrroloindoles **5a**–**c**, **6a**–**c** were obtained with up to quantitative yield and 99% enantiomeric excess. Halogenated tryptamines, indole-3-acetic acid amides and tryptophol were also converted without difficulty to products **4**, **7**, and **8a**. The precursor of product **8b** merely underwent conversion to the prenylated indolenine but cyclization occurred spontaneously upon storage of this purified intermediate. The established method seems to be incompatible with indoles that are substituted in the 2- or 7-position, which we ascribe to their inability to form an adduct with the activating borane. Accordingly, 2-methyl-*N*-Boc-tryptamine and 7-bromo-4-methoxy-*N*-Boc-tryptamine were unreactive.

A survey of the scope of allylic substrates in combination with **2a** resulted in moderate to excellent yields (Figure 2B). For the allylation using **1b** the pyrroloindole (+)-**10** was obtained with a low enantioselectivity, clearly indicating that our protocol is not applicable to unbranched allyl cation precursors. With the racemic chiral substrates **1c**–**e** the pyrroloindoles **11**–**13** were formed with poor diastereoselectivities but complete regioselectivities, as in none of the cases could a linear allylated product be detected. The enantiomeric excesses for the diastereomers of **11**–**13** differed significantly and reached values of up to 99.8% ee for the major diastereomer of **12**.

### Mechanistic Investigations for the Iridium–NHC–Phosphoramidite Catalyzed Prenylation

Our initial objective regarding the mechanism of the prenylation with iridium–NHC–phosphoramidite complexes was to identify the active catalyst. Probing the in situ prepared complex from **precat. 1** (1 equiv), (*R*)-**L1** (2 equiv) and DBU (8 equiv) in CD_2_Cl_2_ showed two major singlets in the ^31^P NMR spectrum at 134.1 and 132.9 ppm with an intensity ratio of 1/1.1, respectively. The most characteristic signals in the ^13^C NMR spectrum were two doublets at 175.4 and 174.8 ppm both with a coupling constant of 14.4 Hz, corresponding to a carbene that couples to a phosphorus in cis-arrangement which is in accordance with the transphobia effect.^54^ Presumably the two sets of signals arise from different orientations of the unsymmetrically substituted carbene ligand which was confirmed by the NMR-data of the complex that was prepared analogously from **precat. 2** in CDCl_3_. Here the ^31^P NMR spectrum showed a singlet at 132.7 ppm and the ^13^C NMR spectrum exhibited a single doublet (174.9 ppm, *J* = 14.6 Hz) within the range of chemical shifts for carbenes. In order to elucidate their chemical structures, we conceived a simple synthesis of the presumed complexes (Scheme 4). Applying the well-established silver-carbene transmetalation procedure to the imidazolium chlorides **im-a** and **im-b** furnished the iridium carbene complexes **K-1a** and **K-1b**.^55,56^ A subsequent ligand exchange yielded the iridium–NHC–phosphoramidite complexes **K-2a** and **K-2b** that were spectroscopically identical to the main components generated by the corresponding in situ procedures as verified by ^1^H-, ^13^C- and ^31^P NMR spectroscopy. The only observable deviation was the isomeric ratio of **K-2b** as indicated by its ^31^P NMR spectrum showing an intensity ratio of 2/1. A high temperature NMR-experiment of **K-2b** in C_6_D_6_ at 343 K did not lead to a coalescence of the signals. Similarly, **K-2a** possesses a high rotational barrier of its NHC-ligand since the NHC-bound methyl groups are magnetically inequivalent at ambient temperature as observed by ^1^H- and ^13^C NMR-spectroscopy. These iridium­(I)-complexes **K-2a** and **K-2b** feature a typical square-planar geometry in which the olefins are coordinated, as evidenced by their ^13^C NMR downfield shifts (**K-2a** (CDCl_3_): 65.5, 64.5 ppm; **K-2b** (CD_2_Cl_2_): 65.9, 64.6 ppm and 66.6, 63.1 ppm for the major and minor isomer, respectively). **K-2a** and **K-2b** did not suffer any decomposition or ligand exchange leading to Ir­(κ^2^-**L1**)_2_Cl upon prolonged storage in solution.

**Scheme 4:**
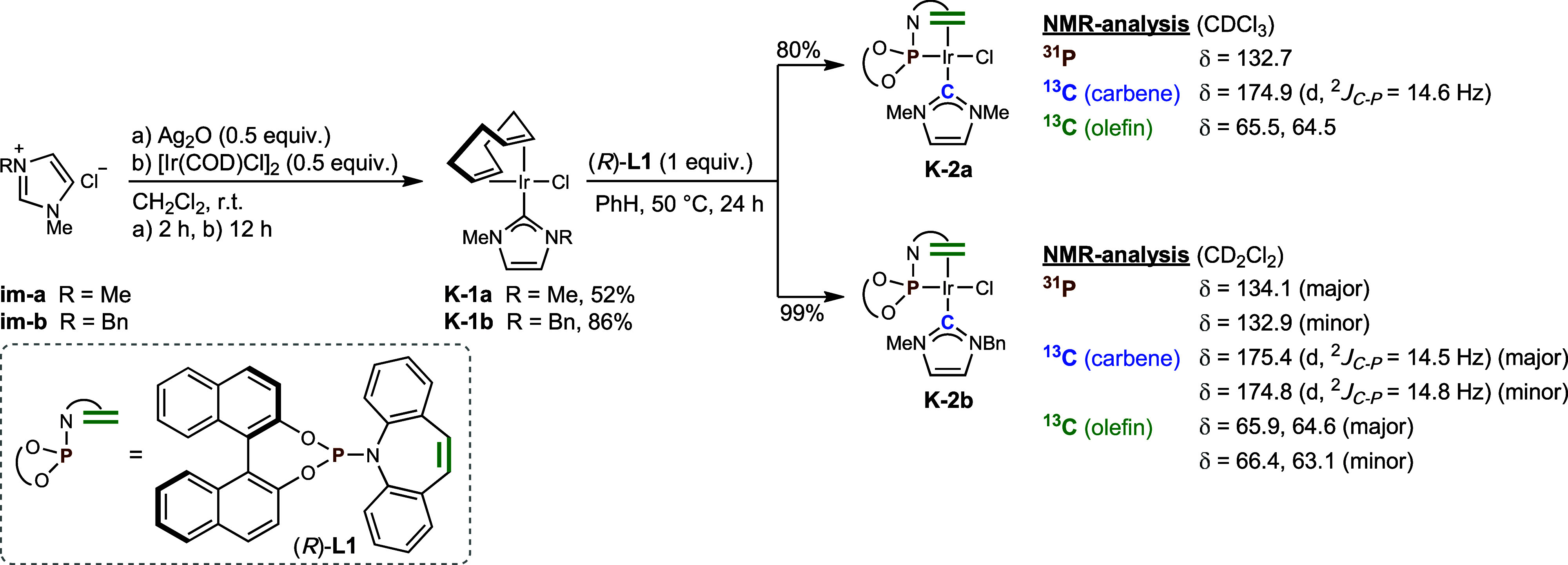
Synthesis of Iridium–NHC–Phosphoramidite Complexes and Selected NMR-Data^a^

a Chemical shifts are reported in ppm.

An evaluation of **K-2b** showed that it possesses a severely reduced catalytic activity in comparison to the in situ generated analog, leading to a diminished yield (58%) and enantioselectivity (94.6% ee) for (−)-**3a** after 20 h. A prolongation of the reaction time led to a quantitative yield (traces of side products could be detected by HPLC that were below the detection limit of ^1^H NMR spectroscopy) and an unexpected increase in enantioselectivity (95.8% ee, Table 4, entry 1^*d*^). We conclude that the active iridium-prenyl-complex can undergo a transformation prior to the nucleophilic attack by the indole. As the concentration of the nucleophile decreases and reaction rates slow down this process becomes a relevant pathway leading to the observed increase in selectivity. The major difference between the isolated catalyst **K-2b** and the in situ generated catalyst is that the latter contains the hydrochloride of the amidine base resulting from the in situ formation of the carbene. Since it is well documented that coordinating ions like chloride can have a profound influence on the course of allylic substitutions, we were prompted to investigate their impact.^42,43,57–64^ Pleasingly, addition of 10 mol % of tetrabutylammonium chloride resulted in a complete restoration of the catalytic activity of **K-2b** (Table 4, entry 2). An equal amount of tetrabutylammonium bromide was almost as effective (Table 4, entry 3). This suggests that additional chloride and bromide ions can coordinate to the cationic prenyl complex formed by the oxidative addition of **1a**, resulting in the formation of a neutral complex. To investigate whether a neutral prenyl complex is reactive toward the indole-nucleophile, the catalysis was performed with 100 mol % tetrabutylammonium chloride, as this should shift the equilibrium between the cationic and neutral complexes in favor of the latter (Table 4, entry 4). Under these conditions, only trace amounts of product **3a** were formed, indicating that a neutral prenyl complex is unreactive. Therefore, we conclude that the chloride/bromide ions induce an isomerization of the initially formed cationic prenyl complex that proceeds via a neutral intermediate. This process could not be provoked by iodide ions as the addition of 10 mol % of tetrabutylammonium iodide resulted in the same enantioselectivity compared to the catalysis without any additional halide (Table 4, entry 5). Instead, the yield was reduced (39% compared to 58%), which we attribute to the formation of an unreactive iodide-containing neutral complex.

**Table 4:**
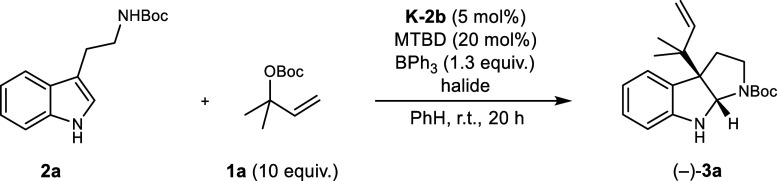
Evaluation of the Catalytic Activity of Complex K-2b^a^

entry	halide	yield^b^	ee^c^
1	none	58%, 100%^d^	94.6%, 95.8%^d^
2	Bu_4_N^+^Cl^–^ (10 mol %)	100%	97.3%
3	Bu_4_N^+^Br^–^ (10 mol %)	98%	97.1%
4	Bu_4_N^+^Cl^–^ (100 mol %)	traces	n.d
5	Bu_4_N^+^I^–^ (10 mol %)	39%	94.4%

a All reactions were conducted according to the general procedure for the prenylation with isolated iridium–NHC–phosphoramidite catalysts (cf. Supporting Information).

b Isolated yield.

c Determined by chiral HPLC.

d Reaction time 5 days. n.d.: not determined.

To provide evidence for the isomerization via a neutral allyl complex, we envisioned that this intermediate could be generated through the oxidative addition of an allylic chloride to either **K-2a** or **K-2b**. The oxidative addition of 1,1-dimethylallyl chloride to both complexes occurred within seconds at room temperature in CDCl_3_, as observed by decolorization. Unfortunately, the putative neutral prenyl complexes could not be detected. Instead, isoprene, resulting from a β-hydride elimination, was identified by ^1^H NMR spectroscopy, and the ^31^P NMR spectra showed a major unknown decomposition product at 4.3 ppm.

In contrast, the reaction of **K-2a** with allyl chloride (2 equiv) in CDCl_3_ at room temperature led to the formation of a stable allyl complex **K-3a** (spectra are provided in the Supporting Information). The ^1^H NMR spectrum of **K-3a** exhibited two sets of signals corresponding to *exo*/*endo*-allyl isomers. Due to *syn*-*anti* isomerization, one set of signals was significantly broadened. Consequently, the ^31^P NMR spectrum showed a broad singlet at 61.3 ppm and a sharp singlet at 62.5 ppm. A low temperature NMR at 243 K resulted in signal sharpening, and the ^31^P NMR spectrum revealed two additional resonances at 65.2 and 95.6 ppm, though with marginal intensity. The *syn*- and *anti*-hydrogen resonances of the two major isomers appeared as distinct signals between 4.03–2.28 ppm. The allyl-C2-hydrogens resonated at 5.40 and 4.72 ppm, respectively. The ^13^C-resonances of the allyl-C2 atoms appeared at 96.8 and 100.5 ppm, respectively. The terminal carbon atoms resonated at 66.5, 32.8 ppm and 79.9, 30.1 ppm, indicative of η^3^-allyl ligands, as opposed to η^1^-allyl ligands, which usually exhibit lower chemical shifts for their methylene carbon atom.^65^ The minor species with a ^31^P-shift of 65.2 ppm was slightly more populated in CD_2_Cl_2_ at 243 K (shifted to 64.7 ppm), which facilitated its identification as an η^3^-allyl complex with ^13^C-shifts of its allyl-C2 carbon atom at 97.5 ppm and its terminal carbon atoms at 71.4 and 35.0 ppm, respectively. Interestingly, in all of the identified allyl complexes, the olefin of (*R*)-**L1** is noncoordinating. Thus, it is assumed that chloride, as the leaving group of allyl chloride, is coordinated to iridium, which would lead to the formation of the 6-fold coordinated, 18-valence electron neutral allyl complex **K-3a** (Scheme 5). ESI mass spectrometry did not show the molecular ion peak of **K-3a** but rather the peak corresponding to [(**K-3a**)−Cl^–^]^+^. For this reason, isolated **K-3a** was reacted with 1 equiv. Silver hexafluoroantimonate, but no precipitation of silver chloride was observed at −20 °C after 2 min, supporting the assumption that chloride is indeed coordinated to iridium. Upon warming to room temperature, however, immediate precipitation occurred, indicating a temperature-dependent exchange rate of the chloride ligand (cf. the Supporting Information for the experimental procedure and additional spectroscopic evidence). We conclude that the recoordination of the olefin of (*R*)-**L1** can either displace a chloride ligand or force the allyl ligand into a η^1^-coordination, which would represent the mechanistic basis of the observed isomerization.

**Scheme 5:**
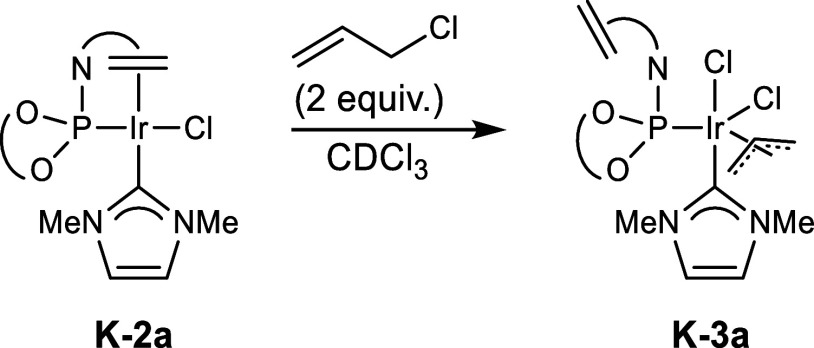
Synthesis of the Neutral Allyl Complex K-3a

Findings related to our observations have been documented, as it is known that chloride ions exert their effect by increasing the equilibration rate of isomeric allyl complexes. Besides this, the equilibrium ratio of allyl isomers can be impacted.^42,43,57–63^ In the event that the ancillary ligands are unaffected by these isomerizations a prenyl complex can adopt four isomeric forms which are interconverted by either a *syn*-*anti* isomerization or an (apparent) allyl rotation (Scheme 6A).^66^ Regarding a prenyl complex derived from either **K-2a** or **K-2b** both processes lead to an overall diastereomerization because the complex bears an axially chiral BINOL-ligand. The former due to an inversion of the planar chirality of the prenyl ligand as well as the iridium-stereocenter whereas the latter only inverts the configuration at iridium. Both isomerization mechanisms proceed via an η^1^-allyl-complex and the *syn*-*anti* isomerization results in the exchange of the methyl groups or the hydrogen atoms, depending on which allyl terminus is bound to iridium in the η^1^-allyl-complex (Scheme 6A exemplifies this process for the *syn*-*anti* exchange of *R* ^1^ and *R* ^2^).

**Scheme 6:**
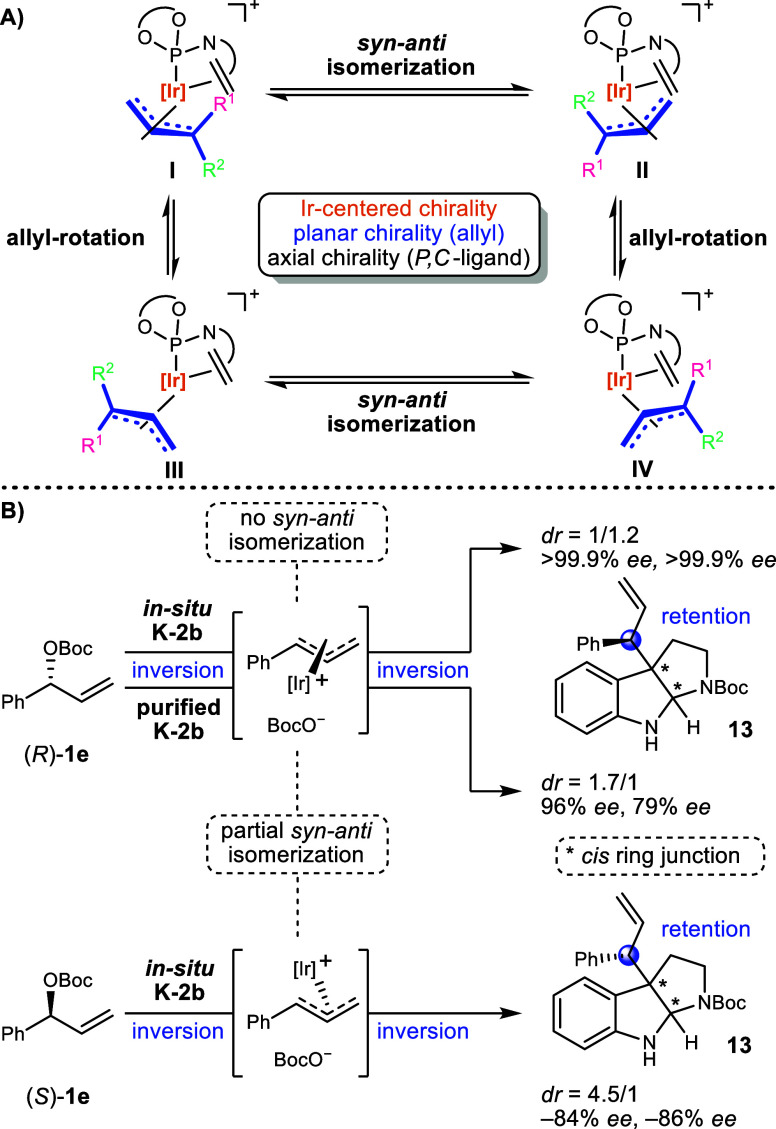
(A) Isomerization of Allyl Complexes. (B) Stereochemical Course for the Formation of 13

The stereochemical course and the type of isomerization that is triggered by the chloride ions cannot be determined in case of the reverse prenylation since the allylic position does not represent a stereogenic center in both the substrate **1a** and the prenylated products. We therefore decided to investigate the allylic alkylation of the chiral precursor **1e** with the model nucleophile **2a** in more detail. Accordingly, the reaction was carried out using either enantiomer of **1e** under the established standard conditions with the in situ generated catalyst **K-2b** (Scheme 6B). When using enantiomerically pure (*R*)-**1e** product **13** was afforded with very similar diastereo- and enantioselectivities (dr = 1/1.2; >99.9% ee, >99.9% ee) as obtained when using an excess (5 equiv) of racemic **1e** (dr = 1/1.1; 99% ee, 99.7% ee). Conversely, with enantiomeric carbonate (*S*)-**1e**, the trend in diastereo- and enantioselectivity was inverted (dr = 4.5/1; −84% ee, −86% ee), clearly indicating that (*R*)-**1e** represents the matched case of the oxidative addition reaction. Since both diastereomers are enantiomerically pure when using (*R*)-**1e** (the minor enantiomers could not be detected by chiral HPLC), the allyl complex does not suffer a *syn*-*anti* isomerization and the configuration at the allylic position can be deduced from the common double-inversion-retention-mechanism for allylic substitutions with soft nucleophiles.^66^ Applying the same rationale for (*S*)-**1e**, its corresponding allyl complex undergoes partial *syn*-*anti* isomerization (via the less substituted allyl terminus) leading to a reduced enantiopurity at the allylic position in both diastereomers of **13** (−84% ee, −86% ee).

To probe the influence of the chloride ions, **13** was synthesized using the purified catalyst **K-2b** and (*R*)-**1e** (Scheme 6B). This resulted in an inverted trend in diastereoselectivity (dr = 1.7/1) and an identical, albeit less pronounced trend in enantioselectivity (96% ee, 79% ee) in comparison to the established chloride-containing system. This clarifies that both reactions mainly proceed via diastereomeric allyl complexes that do not differ in the planar chirality of their allyl ligands. Hence, the chloride ions provoke a fast allyl rotation and the subsequent product formation is equally fast, so that a *syn*-*anti* isomerization becomes a minuscule pathway. In contrast, in the chloride-free system, a *syn*-*anti* isomerization leads to the partial racemization of the allylic position in both diastereomers of **13** (96% ee, 79% ee). In case of the unsymmetrically substituted phenylallyl-ligand Scheme 6A extends to a more complicated scenario as the phenyl substituent can either occupy the *syn*- or *anti*-position in each isomer, resulting in eight possible structures. Due to their lower energy, the involved allyl complexes presumably contain allyl ligands with the *syn*-orientation (e.g., type **I**, *R* ^1^ = H, *R* ^2^ = Ph) and the occurring *syn*-*anti* isomerizations proceed via the less substituted allyl terminus.^31^

Based on our mechanistic investigations, we propose the catalytic cycle for the iridium–NHC–phosphoramidite-catalyzed reverse prenylation, as illustrated in Scheme 7. Coordination of the allylic substrate **1a** to complex **K-2b** forms the olefin complex **K–I**, which, upon oxidative addition, can generate two distinct Ir­(III)-η^3^-prenyl complexes. These *exo*/*endo*-diastereomers, **K-IIa** and **K-IIb**, are both capable of forming the product olefin complex **K**–**III** via nucleophilic attack by the indole. As shown in Table 4, the oxidative addition preferentially yields the less reactive isomer **K-IIa**, whose conversion to the product represents a minor pathway in the presence of chloride ions, as these provoke a fast allyl rotation. This isomerization proceeds via the neutral complex **K-IVa** that forms upon displacement of the phosphoramidite ligand’s olefin by an external chloride ligand. Recoordination of the olefin shifts the prenyl ligand into η^1^-coordination and thereby furnishes **K-Va**. A rotation around the iridium–carbon bond followed by a shift back to η^3^-coordination generates **K-IVb**, the isomer of **K-IVa**, in which the prenyl ligand has undergone a fast and reversible allyl rotation. Displacement of one chloride ligand by the olefin moiety of the phosphoramidite ligand generates the more reactive cationic complex **K-IIb**, which undergoes nucleophilic attack by the indole to form the product olefin complex **K-III**. Transmetalation with the allylic substrate **1a** completes the catalytic cycle.

**Scheme 7:**
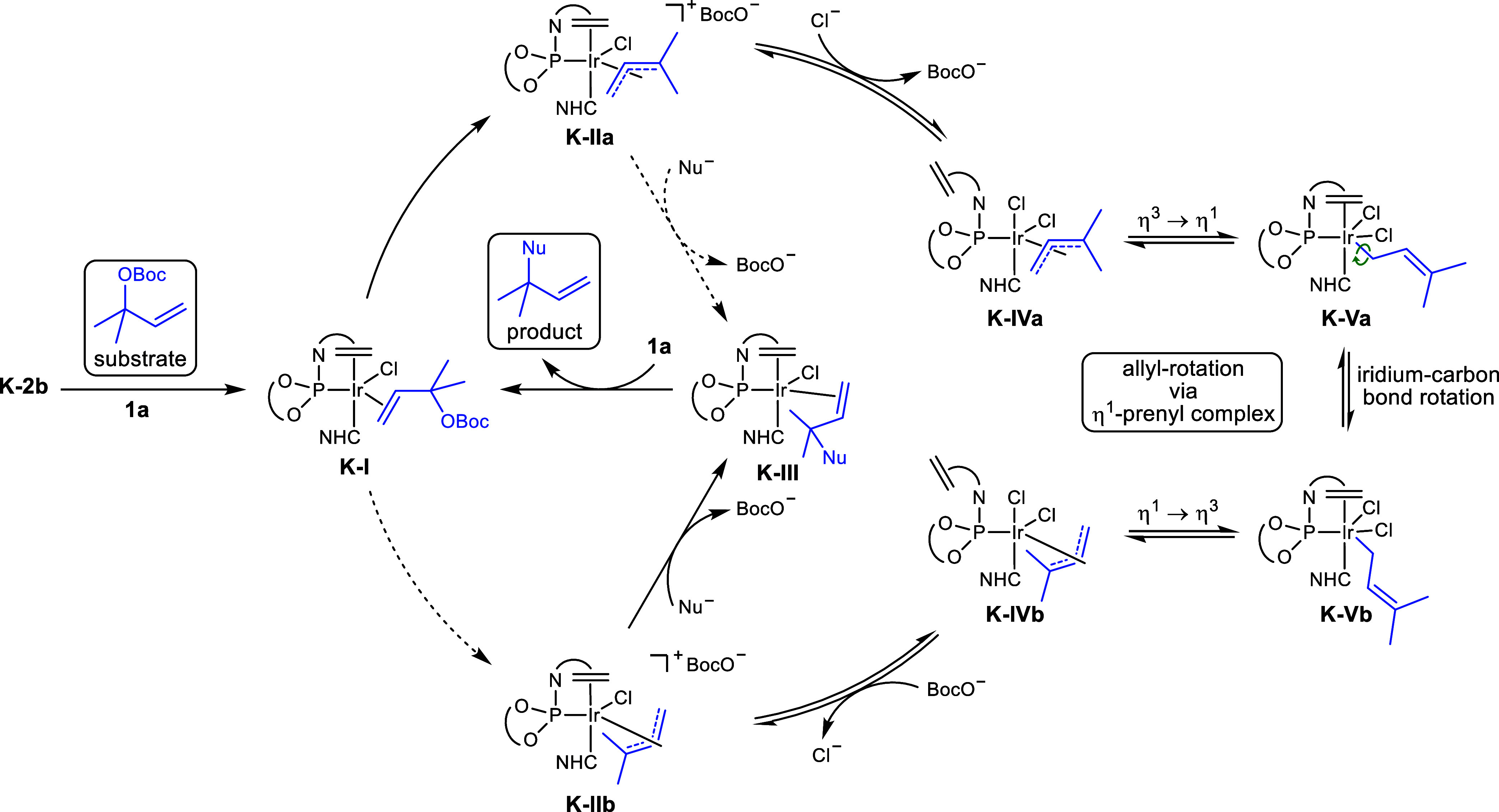
Proposed Catalytic Cycle for the Iridium–NHC–Phosphoramidite Catalyzed Reverse Prenylation^a^

a The structure of the phosphoramidite ligand (*R*)-**L1** is abbreviated as depicted in Scheme 4. Nu^–^ represents the deprotonated indole-nucleophile. Minor reaction pathways are indicted with dashed arrows.

### Total Synthesis of (−)-Flustramine A **(14)**

A series of reverse prenylated indole alkaloids that do not contain a cyclic peptide were isolated from the marine bryozoan .^19,67–71^ As exemplified by (−)-flustramine A (**14**), they possess a brominated *N* ^1^-methyl-HPI-structure though flustramine C features a partially unsaturated tetrahydropyrrolo­[2,3-*b*]­indole core.^20^ In addition to antibacterial^72^ and cytotoxic^73,74^ properties, some reverse prenylated flustramines inhibit biofilm formation by intercepting bacterial quorum sensing.^72,75,76^ (−)-Flustramine A (**14**) also exhibits muscle relaxant effects, as shown by in vitro and in vivo experiments.^77^ To demonstrate the synthetic applicability of our protocol, we developed a concise total synthesis of (−)-Flustramine A (**14**), starting from commercially available 6-bromo-Boc-tryptamine **2b** (Scheme 8). The reverse prenylation of **2b**, according to Table 3 (entry 8^e^), gave the key intermediate (−)-**4c** with a yield of 91% and an enantiomeric excess of 97%. The linear *N* ^8^-prenyl group was installed by reductive amination with an excellent yield of 97% for (−)-**15**. Subsequent deprotection of the Boc-group was accomplished by trimethylsilyl iodide and was accompanied by a partial isomerization of the reverse prenyl group as well as its migration to *N* ^1^ in the linear form. A second reductive amination of the crude mixture furnished (−)-flustramine A (**14**) in high purity after standard silica column chromatography. Thus, (−)-flustramine A (**14**) was obtained from commercially available starting materials in four steps with an overall yield of 53% and an enantiomeric excess of 97%.

**Scheme 8:**
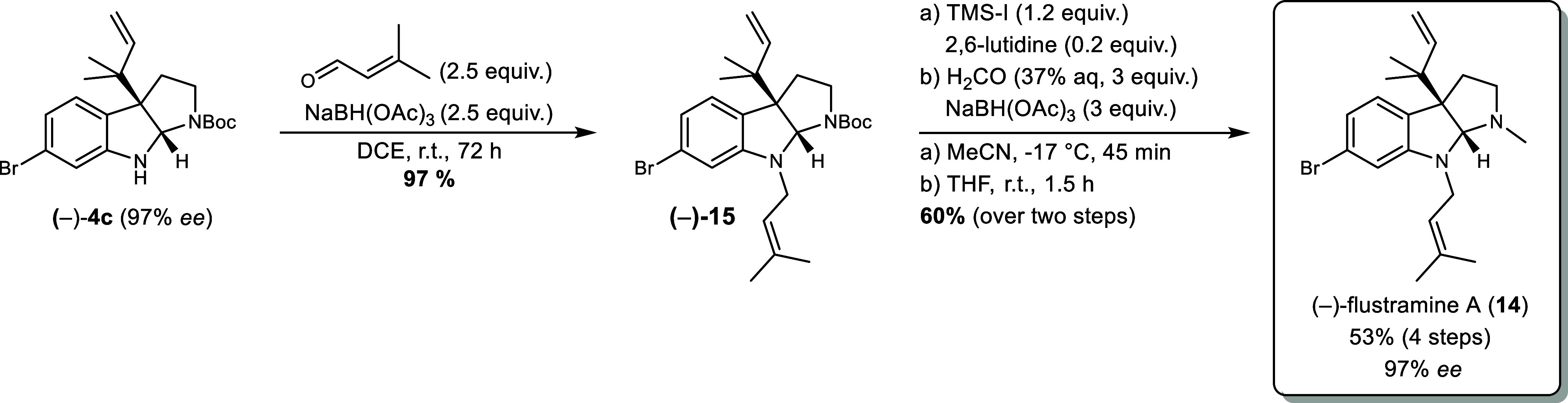
Total Synthesis of (−)-Flustramine A (14)

## Conclusion

The methodology presented here constitutes the first catalytic enantioselective reverse prenylation of achiral tryptamines and other 3-substituted indoles that furnishes hexahydropyrrolo­[2,3-*b*]­indoles in a single step. The products are obtained in good to excellent yields and with enantiomeric excesses of up to 99%. The developed catalytic system is based on an iridium complex comprising a chiral phosphoramidite-olefin ligand in combination with an achiral NHC-ligand. To the best of our knowledge, this is the first example in which both phosphoramidite and NHC-ligands are deliberately integrated into a single iridium complex for use in allylic substitution reactions. It is worth noting, however, that a study by Glorius et al., which explored cooperative NHC-organocatalysis and iridium catalysis, reported the detection of an iridium–NHC–phosphoramidite complex by high-resolution mass spectrometry.^64^ From the mechanistic investigations, we assume that the catalysis follows the common catalytic cycle of an allylic substitution but deviantly features a chloride induced isomerization that generates a more reactive prenyl complex which also leads to an increased enantioselectivity. For a stable allyl complex derivative, this isomerization appears to depend on the hemilability of the phosphoramidite-olefin Carreira-type ligand. Implementation of this novel method resulted in a concise total synthesis of (−)-Flustramine A (**14**) with a significantly improved overall yield and reduced number of synthetic steps compared to previous enantioselective routes to this prominent natural product.^42,43,45^

## Supplementary Material



## Notes

Experimental details, full data for the evaluation of the reaction conditions, full characterization of new compounds including ^1^H and ^13^C NMR spectra, HPLC-traces of all compounds (PDF)
