# Supplementary material for: Iridium-Catalyzed Regio- and Enantioselective Reverse Prenylation of Tryptamines and Other 3‑Substituted Indoles
Source: J Am Chem Soc. 2025 Jul 17;147(30):26506–17. doi: 10.1021/jacs.5c06364 (PMC12314904; doi:10.1021/jacs.5c06364)
Supplement: Supplementary file 1 [file ja5c06364_si_001.pdf]

Supporting Information

Iridium-Catalyzed Regio- and Enantioselective Reverse Prenylation of Tryptamines and Other 3-Substituted Indoles

Leon Sander[1], Jonas M. Müller[1] & Christian B. W. Stark[1]*

[1] Fachbereich Chemie, Institut für Organische Chemie, University of Hamburg, Martin-Luther-King Platz 6, 20146 Hamburg (Germany)

*Corresponding author. e-mail: stark@chemie.uni-hamburg.de

Table of Contents

1. General information 4

2. Preparation of phosphoramidite ligands 5

2.1. Preparation of BINOL-derivatives 5

2.2. General procedure for the preparation of phosphoramidite ligands (GP 1) 5

3. Preparation of allyl carbonates 8

3.1. General procedure for the preparation of allyl carbonates (GP 2) 8

4. Preparation of boranes 10

4.1. General procedure for the preparation of alkyl boranes (GP 3) 10

4.2. Preparation of triarylboranes 12

5. Preparation of indoles 13

6. Preparation of tryptamine-derivatives 14

7. Evaluation of the reaction conditions 23

7.1. Supplementary Table 1 24

7.2. Supplementary Table 2 25

8. Procedures for the catalyst preparation and reverse prenylation 26

8.1. General procedure for the preparation of precatalysts (GP 4) 26

8.2. General procedure for the preparation of iridium-NHC-phosphoramidite complexes (GP 5) 27

8.3. General procedure for the reverse prenylation with iridium-bis-phosphoramidite catalyst (GP 6) 30

8.4. General procedure for the reverse prenylation with *in-situ* generated iridium-NHC-phosphoramidite catalyst (GP 7) 30

8.5. General procedure for the reverse prenylation with isolated iridium-NHC-phosphoramidite catalyst (GP 8) 30

9. allyl complex K-3a 31

9.1. Preparation of K-3a 31

9.2. Chloride abstraction from K-3a 31

10. Reverse prenylation of Tryptamines and Other 3-substituted Indoles 33

11. Synthesis of (–)-Flustramine A (14) 61

12. NMR spectra 63

12.1. Precatalysts 64

12.2. complex K-2a 72

12.3. complex K-2b 78

12.4. complex K-3a 86

12.5. prenylated and allylated compounds 99

12.6. Synthesis of (–)-Flustramine A (14) 166

13. HPLC-chromatograms 171

13.1. compounds 3–10 171
13.2. compound 11 201
13.3. compound 12 203
13.4. compound 13 205
14. References 209

1. General information

Analytical techniques

NMR spectra were recorded on the Bruker spektrometers *FourierHD 300* (^{1}H-resonance: 300 MHz, ^{13}C-resonance: 75 MHz), *AVANCE III HD 400* (^{1}H-resonance: 400 MHz, ^{13}C-resonance: 100 MHz, ^{31}P-resonance: 162 MHz, ^{11}B-resonance: 128 MHz), *AVANCE I 400* (^{1}H-resonance: 400 MHz, ^{13}C-resonance: 100 MHz), *AVANCE I 500* (^{1}H-resonance: 500 MHz, ^{13}C-resonance: 125 MHz), *AVANCE III HD 600* (^{1}H-resonance: 600 MHz, ^{13}C-resonance: 150 MHz, ^{31}P-resonance: 243 MHz, ^{19}F-resonance: 565 MHz, ^{11}B-resonance: 193 MHz). ^{1}H-NMR data are referenced to the residual proton signal of the deuterated solvent: $CDCl_3$, δ = 7.26 (s); CD_2Cl_2, δ = 5.32 (t, *J* = 1.1 Hz); DMSO-d6, δ= 2.50 (p, *J* = 1.9 Hz); MeOH-d4, δ = 3.31 (p, *J* = 1.7 Hz); Benzol-d6, δ = 7.16 (s); THF-d8, δ = 3.58 (m), 1.73 (m). ^{13}C-NMR data are referenced to the deuterated solvent: $CDCl_3$, δ = 77.16 (t, *J* = 32 Hz); DMSO-d6, δ = 39.52 (sep, *J* = 21 Hz); MeOH-d4, δ = 49.00 (sep, *J* = 21.4 Hz); Benzol-d6, δ = 128.06 (t, *J* = 24.3 Hz); THF-d8, δ = 67.57 (p, *J* = 22.2 Hz), 25.37 (p, *J* = 20.2 Hz). ^{31}P-NMR data are externally referenced to 85% H_3PO_4. ^{19}F-NMR data are externally referenced to CCl_3F. ^{11}B-NMR data are externally referenced to $NaBF_4$. Chemical shifts δ are reported in parts per million (ppm) and coupling constants *J* in Hertz (Hz). Multiplicity is denoted as follows: singlet (s), doublet (d), triplet (t), quartet (q), pentet (p), septet (sep), multiplet (m), broad signal (br).

High resolution ESI mass spectra were recorded on an *Agilent 6224 ESI-TOF.* GC-EI mass spectra were recorded on a *ThermoFischer Thermo ISQ LT EI* coupled to a *Thermo Trace 1300* GC-system.

Enantiomeric ratios were determined by HPLC analysis with an *Agilent 1260 Infinity II LC* system using the chiral stationary phase *Daicel Chiralpak AD-H* (4.6 mm×250 mm, particle size: 5 µm) and *Daicel Chiralcel OD-H* (4.6 mm×250 mm, particle size: 5 µm).

Optical rotations were measured on *A. Krüss GmbH P8000* polarimeter at 589 nm (sodium D-line) and the specific optical rotations are reported as follows: $[\alpha]_D^T$ (*c* [g/100 mL], solvent).

IR-spectra were measured on a *Bruker ALPHA Platinum ATR* spectrometer. Absorption maxima $\tilde{\nu}$ are reported in cm^{-1}.

Reagents and solvents

Unless stated otherwise, all reactions were carried out in oven-dried glassware with dry solvents under an inert atmosphere of nitrogen and magnetic stirring. All solvents of technical grade were purified and dried according to standard methods prior to use. Waterfree solvents were obtained from *Acros*. Waterfree and degassed hexane and benzene were obtained by refluxing over NaK-alloy for 48 h and subsequent destillation. Reagents were obtained from *Sigma Aldrich*, *Acros*, *Merck, Fluka*, *TCI*, *ABCR*, *Alfa Aesar*, *FluoroChem* and *BLDpharm* and were used as received.

Isolation and Purification

Substances were purified by flash column chromatography using silica gel 60 F_{254} (230–400 mesh) obtained from *Fluka*. For substances that are eluted by a solvent mixture containing triethylamine the silica gel was wetted with petroleum ether/triethylamine (19/1, v/v) and washed with petroleum ether (1 column volume) prior to loading the substance. Products were identified by analytical thin layer chromatography using *ALUGRAM® Xtra SIL G/UV$_{254}$* TLC-plates obtained from *Macherey-Nagel*. Visualization was achieved with 254 nm light or staining with ceric-reagent (5 g phosphomolybdic acid, 2 g ceric(IV)sulfate, 16 mL H_2SO_4 (96%), 200 mL H_2O) or permanganate-reagent (1.5 g $KMnO_4$, 10 g K_2CO_3, 1.25 mL NaOH (10 %), 200 mL H_2O).

2. Preparation of phosphoramidite ligands

2.1. Preparation of BINOL-derivatives

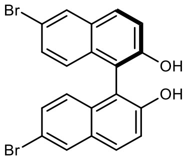

(*R*)-6,6'-dibromo-[1,1'-binaphthalene]-2,2'-diol. Prepared according to a known procedure[1] and purified additionaly by recrystallization from toluene/*n*-hexane.[2] All analytical data are in accordance with the literature.

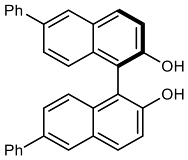

(*R*)-6,6'-diphenyl-[1,1'-binaphthalene]-2,2'-diol. Prepared according to a known procedure.[3] All analytical data are in accordance with the literature.[4]

2.2. General procedure for the preparation of phosphoramidite ligands (GP 1)

To a solution of the BINOL-derivative (1 equiv.) and NEt_3 (3 equiv.) in THF/Et_2O (1/1, 10 mL/mmol) was added dropwise at 0 °C PCl_3 (1 equiv.). The suspension was warmed to room temperature and stirred for 1 hour and subsequently filtered over celite under an atmosphere of nitrogen. The filter cake was washed with dry THF/Et_2O (1/1) and the combined filtrates were stripped of solvent under reduced pressure. The obtained product was stored under an inert atmosphere. Either 5*H*-dibenzo[*b,f*]azepine or 9*H*-carbazole (1 equiv.) were dissolved in THF (5 mL/mmol) and cooled to -78 °C. *n*-Butyllithium (0.95 equiv., 1.6 M in hexane) was added dropwise and the solution was stirred for 10 minutes. The

beforehand prepared chlorodioxaphosphepine was added as a solution in THF (3 mL/mmol). The reaction mixture was warmed to room temperature and stirred for 12 hours. Water and EtOAc were added and the phases separated. The aqueous phase was extracted with EtOAc (×2) and the combined organic phases were dried over $MgSO_4$ and the solvent removed under reduced pressure. The products were purified by flash column chromatography.

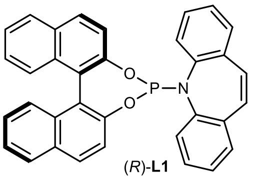

(*R*)-**L1**

5-((11b*R*)-dinaphtho[2,1-*d*:1',2'-*f*][1,3,2]dioxaphosphepin-4-yl)-5*H*-dibenzo[*b,f*]azepine. Prepared according to **GP 1** with (*R*)-[1,1'-binaphthalene]-2,2'-diol (10.0 mmol) and 5*H*-dibenzo[*b,f*]azepine (10.0 mmol). Chromatographic purification: silica gel, petroleum ether:CH_2Cl_2, 4:1, v/v. Yield: 3.82 g, 7.52 mmol, 75%, colorless solid.

TLC (petroleum ether:CH_2Cl_2, 4:1, v/v): $R_f = 0.20$. All analytical data are in accordance with the literature.[5]

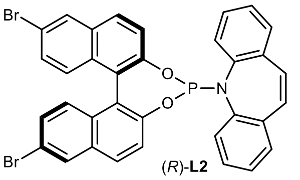

(*R*)-**L2**

5-((11b*R*)-9,14-dibromodinaphtho[2,1-*d*:1',2'-*f*][1,3,2]dioxaphosphepin-4-yl)-5*H*-dibenzo[*b,f*]-azepine. Prepared according to **GP 1** with (*R*)-6,6'-dibromo-[1,1'-binaphthalene]-2,2'-diol (1.00 mmol) and 5*H*-dibenzo[*b,f*]azepine (1.00 mmol). Chromatographic purification: silica gel, petroleum ether:CH_2Cl_2, 4:1, v/v. Yield: 413 mg, 0.621 mmol, 62%, colorless solid.

TLC (petroleum ether:CH_2Cl_2, 4:1, v/v): $R_f = 0.20$. **mp**: 210 °C. $[\alpha]_D^{20}$: –274 (0.5, CH_2Cl_2). All spectroscopic data are in accordance with the data for the (+)-enantiomer.[6]

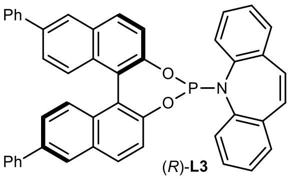

(*R*)-**L3**

5-((11b*R*)-9,14-diphenyldinaphtho[2,1-*d*:1',2'-*f*][1,3,2]dioxaphosphepin-4-yl)-5*H*-dibenzo[*b,f*]-azepine. Prepared according to **GP 1** with (*R*)-6,6'-diphenyl-[1,1'-binaphthalene]-2,2'-diol (1.00 mmol)

and 5*H*-dibenzo[*b,f*]azepine (1.00 mmol). Chromatographic purification: silica gel, petroleum ether:CH_2Cl_2, 5:1, v/v. Yield: 286 mg, 0.433 mmol, 43%, colorless solid.

TLC (petroleum ether:CH_2Cl_2, 5:1 v/v): R_f = 0.15. **mp**: 250 °C. **$[\alpha]_D^{20}$**: –417 (0.5, CH_2Cl_2). **^{1}H-NMR** (600 MHz, $CDCl_3$): δ 8.13 (d, *J* = 1.9 Hz, 1 H), 8.07 (d, *J* = 8.8 Hz, 1 H), 8.00 (d, *J* = 1.9 Hz, 1 H), 7.76–7.68 (m, 4 H), 7.56–7.14 (m, 17 H), 7.02 (d, *J* = 11.5 Hz, 1 H), 6.98–6.92 (m, 2 H), 6.88 (d, *J* = 8.8 Hz, 1 H), 6.59 (td, *J* = 7.6 Hz, 1.6 Hz, 1 H). **^{13}C-NMR** (150 MHz, $CDCl_3$): δ 150.27 (d, *J* = 8.1 Hz), 148.95, 143.05 (d, *J* = 24.5 Hz), 142.64, 140.84, 140.82, 137.70, 136.96, 136.61 (d, *J* = 3.5 Hz), 135.32, 132.16, 131.91, 131.68, 131.50, 131.47, 130.78, 130.65, 129.43, 129.34, 129.23, 129.19, 129.08, 128.80, 127.83, 127.53, 127.46, 126.90, 126.38, 126.23, 125.95, 125.77, 125.40, 128.05 (d, *J* = 10.0 Hz), 124.31 (d, *J* = 5.3 Hz), 122.77, 122.04, 121.12 (d, *J* = 2.7 Hz). **^{31}P-NMR** (162 MHz, $CDCl_3$): δ 137.92. **IR** (ATR): $\tilde{\nu}$ 2957, 2922, 1589, 1485, 1457, 1442, 1335, 1283, 1234, 1201, 1157, 1107, 1076, 1040, 985, 944, 918, 887, 866, 825, 814, 797, 753, 719, 695, 656, 633, 620, 582, 564, 548, 517, 464. **HRMS** (ESI, m/z): $[M+H]^+$ calc. for $C_{46}H_{31}NO_2P$, 660.209; found, 660.209.

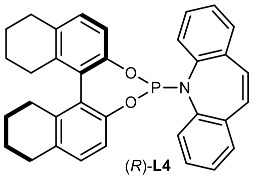

(*R*)-**L4**

5-((11b*R*)-8,9,10,11,12,13,14,15-octahydrodinaphtho[2,1-*d*:1',2'-*f*][1,3,2]dioxaphosphepin-4-yl)-5*H*-dibenzo[*b,f*]azepine. Prepared according to **GP 1** with (*R*)-5,5',6,6',7,7',8,8'-octahydro-[1,1'-binaphthalene]-2,2'-diol (3.00 mmol) and 5*H*-dibenzo[*b,f*]azepine (3.00 mmol). Chromatographic purification: silica gel, petroleum ether:CH_2Cl_2, 4:1, v/v. Yield: 541 mg, 1.05 mmol, 35%, colorless solid.

TLC (petroleum ether:CH_2Cl_2, 4:1, v/v): R_f = 0.20. **mp**: 190 °C (lit[7] 224 °C). **$[\alpha]_D^{20}$**: –180 (0.5, CH_2Cl_2). All spectroscopic data are in accordance with the literature.[7]

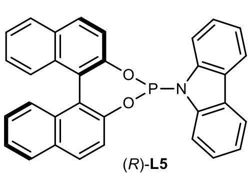

(*R*)-**L5**

9-((11b*R*)-dinaphtho[2,1-*d*:1',2'-*f*][1,3,2]dioxaphosphepin-4-yl)-9*H*-carbazole. Prepared according to **GP 1** with (*R*)-[1,1'-binaphthalene]-2,2'-diol (10.0 mmol) and 9*H*-carbazole (10.0 mmol). Chromatographic purification: silica gel, petroleum ether:CH_2Cl_2, 4:1, v/v. Yield: 1.20 g, 2.50 mmol, 25%, colorless solid.

TLC (petroleum ether:CH_2Cl_2, 4:1, v/v): R_f = 0.20. **$[\alpha]_D^{20}$**: –402 (0.2, $CHCl_3$), lit.[8] $[\alpha]_D^{20}$: –428 (0.17, $CHCl_3$). All spectroscopic data are in accordance with the literature.[8]

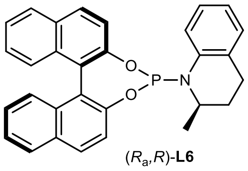

(R_a,R)-**L6**

(2*R*)-1-((11b*R*)-dinaphtho[2,1-*d*:1',2'-*f*][1,3,2]dioxaphosphepin-4-yl)-2-methyl-1,2,3,4-tetrahydro-quinoline. Prepared according to a known procedure. All analytical data are in accordance with the literature.[9]

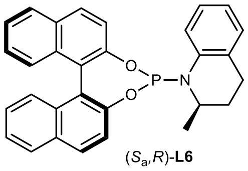

(S_a,R)-**L6**

(2*R*)-1-((11b*S*)-dinaphtho[2,1-*d*:1',2'-*f*][1,3,2]dioxaphosphepin-4-yl)-2-methyl-1,2,3,4-tetrahydro-quinoline. Prepared according to a known procedure. All analytical data are in accordance with the literature.[9]

3. Preparation of allyl carbonates

3.1. General procedure for the preparation of allyl carbonates (GP 2)

To a solution of the allyl alcohol (1 equiv.) in THF (5 mL/mmol) was added dropwise at -78 °C *n*-butyllithium (1 equiv., 1.6 M in hexane). The solution was stirred for 10 minutes and di-*tert*-butyldicarbonate (1 equiv.) was added. The solution was warmed to room temperature and stirred for 12 hours. Five volumes of water were added and the mixture was extracted with Et_2O (×3). The combined organic extracts were washed with brine, dried over $MgSO_4$ and the solvent was removed under reduced pressure. The products were purified by flash column chromatography.

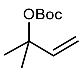

1a

***tert*-butyl (2-methylbut-3-en-2-yl) carbonate**. Prepared according to **GP 2** with 2-methylbut-3-en-2-ol (50 mmol). Chromatographic purification: silica gel, petroleum ether:Et_2O, 99:1, v/v. **TLC**: R_f = 0.30. Yield: 7.8 g, 42 mmol, 84%, colorless oil. All analytical data are in accordance with the literature.[10]

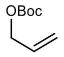

1b

allyl *tert*-butyl carbonate. Prepared according to **GP 2** with prop-2-en-1-ol (50 mmol). Chromatographic purification: silica gel, petroleum ether:Et_2O, 99:1, v/v. **TLC**: R_f = 0.25. Yield: 7.0 g, 45 mmol, 89%, colorless oil. All analytical data are in accordance with the literature.[11]

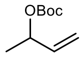

(*rac*)-**1c**

but-3-en-2-yl *tert*-butyl carbonate. Prepared according to **GP 2** with but-3-en-2-ol (10.0 mmol). Chromatographic purification: silica gel, petroleum ether:Et_2O, 99:1, v/v. **TLC**: R_f = 0.30. Yield: 1.58 g, 9.20 mmol, 92%, colorless oil. All analytical data are in accordance with the literature.[12]

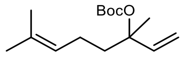

(*rac*)-**1d**

***tert*-butyl (3,7-dimethylocta-1,6-dien-3-yl) carbonate**. Prepared according to **GP 2** with 3,7-dimethylocta-1,6-dien-3-ol (10.0 mmol). Chromatographic purification: silica gel, petroleum ether:Et_2O, 99:1, v/v. **TLC**: R_f = 0.30. Yield: 1.15 g, 4.52 mmol, 45%, colorless oil. All analytical data are in accordance with the literature.[13]

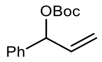

(*rac*)-**1e**

(±)-*tert*-butyl (1-phenylallyl) carbonate. Prepared according to **GP 2** with (±)-1-phenylprop-2-en-1-ol (10.0 mmol). Chromatographic purification: silica gel, petroleum ether:Et_2O, 99:1, v/v. **TLC**: R_f = 0.25. Yield: 1.97 g, 8.42 mmol, 84%, colorless oil. All analytical data are in accordance with the literature.[14]

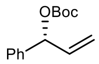

(*R*)-**1e**

(*R*)-*tert*-butyl (1-phenylallyl) carbonate. Prepared according to **GP 2** with (*R*)-1-phenylprop-2-en-1-ol (10.0 mmol). Chromatographic purification: silica gel, petroleum ether:Et_2O, 99:1, v/v. **TLC**: R_f = 0.25. Yield: 1.92 g, 8.20 mmol, 82%, colorless oil. All analytical data are in accordance with the literature.[14]

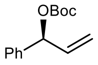

(*S*)-**1e**

(*S*)-*tert*-butyl (1-phenylallyl) carbonate. Prepared according to **GP 2** with (*S*)-1-phenylprop-2-en-1-ol (10.0 mmol). Chromatographic purification: silica gel, petroleum ether:Et_2O, 99:1, v/v. **TLC**: R_f = 0.25. Yield: 1.99 g, 8.51 mmol, 85%, colorless oil. All analytical data are in accordance with the literature.[14]

4. Preparation of boranes

4.1. General procedure for the preparation of alkyl boranes (GP 3)

To a solution of the borane (1 equiv.) in THF (2 mL/mmol) was added the olefin (1.2 equiv.) and the solution was stirred for 12 hours at room temperature. Then, all volatile compounds were removed under high vacuum (10^{-3} mbar) at room temperature and the product was diluted with dry CH_2Cl_2 to reach a concentration of 1 M with regard to the borane. In case the olefin is non-volatile the reagent was used without removing the excess olefin. The stock solutions were stored under nitrogen as some of the boranes are pyrophoric.

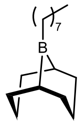

B-octyl-9-BBN

9-octyl-9-borabicyclo[3.3.1]nonane. Prepared according to **GP 3** with 9-borabicyclo[3.3.1]nonane (5.0 mmol, 1 M in THF), 1-octene (6.0 mmol).

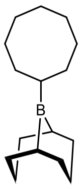

B-cyclooctyl-9-BBN

9-cyclooctyl-9-borabicyclo[3.3.1]nonane. Prepared according to **GP 3** with 9-borabicyclo[3.3.1]-nonane (2.0 mmol, 1 M in THF), (*Z*)-cyclooctene (2.4 mmol).

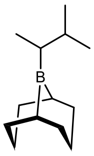

(*rac*)-*B*-siamyl-9-BBN

(*rac*)-9-(3-methylbutan-2-yl)-9-borabicyclo[3.3.1]nonane. Prepared according to **GP 3** with 9-bora-bicyclo[3.3.1]nonane (2.0 mmol, 1 M in THF), 2-methylbut-2-ene (2.4 mmol).

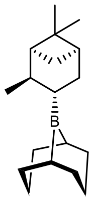

B-(ipc)-9-BBN
(*R*)-Alpine-Borane®

9-((1*S*,2*S*,3*R*,5*S*)-2,6,6-trimethylbicyclo[3.1.1]heptan-3-yl)-9-borabicyclo[3.3.1]nonane. Prepared according to **GP 3** with 9-borabicyclo[3.3.1]nonane (1.0 mmol, 1 M in THF), (1*S*, 5*S*)-(–)-α-pinene (1.2 mmol).

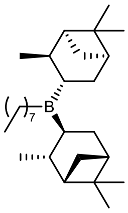

B-(ipc)$_2$octyl

octylbis((1*S*,2*R*,3*S*,5*S*)-2,6,6-trimethylbicyclo[3.1.1]heptan-3-yl)borane. Prepared according to **GP 3** with (+)-Diisopinocampheylborane (1.0 mmol), 1-octene (1.2 mmol).

^{1}H-NMR (600 MHz, C_6D_6): δ 2.43–2.33 (m, 4 H, H-2, H-7$_a$), 2.08–1.94 (m, 6 H, H-1, H-5, H-6$_a$/H-6$_b$), 1.90 (ddd, *J* = 6.7 Hz, 4.9 Hz, 1.9 Hz, 2 H, H-3), 1.57–1.27 (m, 16 H, H-6$_a$/H-6$_b$, H-11–H-17), 1.25 (s, 3 H, H-9), 1.22 (s, 3 H, H-10), 1.08 (d, *J* = 7.1 Hz, 6 H, H-8), 1.02 (d, *J* = 9.5 Hz, 2 H, H-7$_b$), 0.92 (t, *J* = 6.9 Hz, 3 H, H-18). **^{13}C-NMR** (150 MHz, C_6D_6): δ 48.83 (C-3), 41.97 (C-5), 39.66 (C-4), 37.84 (C-2), 34.32 (C-7), 34.07, 30.05, 29.79, 25.48 (C-12, C-13, C-14, C-15), 33.59 (C-1), 32.37 (C-16), 28.85 (C-9), 28.26 (C-6), 23.86 (C-8), 23.15 (C-17), 22.98 (C-10), 14.39 (C-18). **^{11}B-NMR** (193 MHz, C_6D_6): δ 80.0.

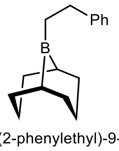

B-(2-phenylethyl)-9-BBN

9-phenethyl-9-borabicyclo[3.3.1]nonane. Prepared according to **GP 3** with 9-borabicyclo[3.3.1]-nonane (2.0 mmol, 1 M in THF), styrene (2.4 mmol).

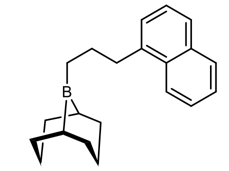

9-(3-(naphthalen-1-yl)propyl)-9-borabicyclo[3.3.1]nonane. Prepared according to **GP 3** with 9-bora-bicyclo[3.3.1]nonane (2.0 mmol, 1 M in THF), 1-allylnaphthalene (2.4 mmol).

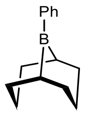

9-phenyl-9-borabicyclo[3.3.1]nonane. Prepared according to a known procedure. All analytical data are in accordance with the literature.[15]

4.2. Preparation of triarylboranes

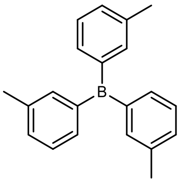

tri-*m*-tolylborane. Prepared according to a known general procedure[16] with 1-bromo-3-methylbenzene (30 mmol). Yield: 3.1 g, 11 mmol, 37%, colorless solid.

^{1}H-NMR (400 MHz, CD_2Cl_2): δ 7.44–7.33 (m, 12 H), 2.39 (s, 9 H). **^{13}C-NMR** (100 MHz, CD_2Cl_2): δ 139.35, 137.14, 135.96, 132.31, 127.61, 21.62. The resonance for the boron-bound carbon atom was not observed. **^{11}B-NMR** (128 MHz, CD_2Cl_2): δ 66.8.

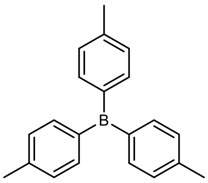

tri-*p*-tolylborane. Prepared according to a known procedure. All analytical data are in accordance with the literature.[16]

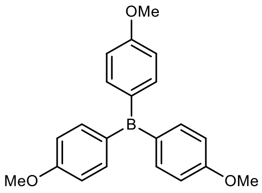

tris(4-methoxyphenyl)borane. Prepared according to a known procedure. All analytical data are in accordance with the literature.[16]

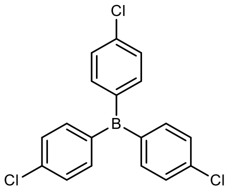

tris(4-chlorophenyl)borane. Prepared according to a known procedure. All analytical data are in accordance with the literature.[16]

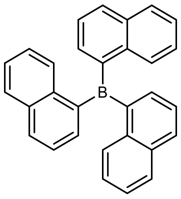

tri(naphthalen-1-yl)borane. Prepared according to a known general procedure[16] with 1-bromo-naphthalene (6.2 g, 30 mmol). Yield: 2.6 g, 6.7 mmol, 22%, colorless solid.

^{1}H-NMR (400 MHz, $CDCl_3$): δ 8.01–7.95 (m, 3 H), 7.90–7.85 (m, 3 H), 7.72–7.67 (m, 3 H), 7.51 (dd, *J* = 6.9 Hz, 1.5 Hz, 3 H), 7.46–7.35 (m, 6 H), 7.10 (ddd, *J* = 8.3 Hz, 6.8 Hz, 1.4 Hz, 3 H). **^{13}C-NMR** (100 MHz, $CDCl_3$): δ 136.46, 135.60, 133.60, 131.67, 129.65, 128.60, 126.02, 125.73, 125.24. The resonance for the boron-bound carbon was not observed. **^{11}B-NMR** (128 MHz, $CDCl_3$): δ 69.0.

5. Preparation of indoles

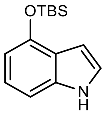

4-((*tert*-butyldimethylsilyl)oxy)-1*H*-indole. Prepared according to a known procedure.[17] All analytical data are in accordance with the literature.[17,18]

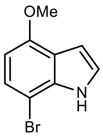

7-bromo-4-methoxy-1*H*-indole. To a solution of 1-bromo-4-methoxy-2-nitrobenzene (2.32 g, 10.0 mmol, 1.00 equiv.) in THF (70 mL) was added at -45 °C vinylmagnesiumbromide (30 mL, 30 mmol, 3.0 equiv., 1 M in THF) in one portion. The solution was stirred at this temperature for one hour whereupon aqueous saturated NH_4Cl solution was added. After warming to room temperature, the mixture was diluted with brine (140 mL) and extracted with EtOAc (×3). The combined organic fractions were washed with brine, dried over $MgSO_4$ and the solvent removed under reduced pressure. Chromatographic purification: silica gel, petroleum ether:EtOAc, 9:1, v/v. Yield: 1.04 g, 4.60 mmol, 46%, off-white solid.

TLC (petroleum ether:EtOAc, 9:1 v/v): R_f = 0.33. **mp**: 114 °C. **^{1}H-NMR** (400 MHz, $CDCl_3$): δ 8.31 (s_{br}, 1 H), 7.24 (d, *J* = 8.2 Hz, 1 H), 7.17 (dd, *J* = 3.2 Hz, 2.3 Hz, 1 H), 6.73 (dd, *J* = 3.2 Hz, 2.3 Hz, 1 H), 6.44 (d, *J* = 8.2 Hz, 1 H), 3.95 (s, 3 H). **^{13}C-NMR** (100 MHz, $CDCl_3$): δ 153.08, 135.57, 124.74, 123.23, 119.68, 101.44, 101.27, 96.29, 55.69. **IR** (ATR): $\tilde{\nu}$ 3407, 3005, 2957, 2923, 2832, 1576, 1505, 1495, 1456, 1352, 1333, 1279, 1264, 1230, 1203, 1180, 1075, 1054, 917, 869, 801, 790, 766, 723, 691, 544, 515, 468. **HRMS** (ESI, m/z): $[M+H]^+$ calc. for C_9H_9BrNO, 225.987; found, 285.985.

6. Preparation of tryptamine-derivatives

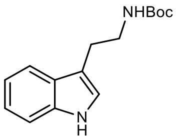

***tert*-butyl (2-(1*H*-indol-3-yl)ethyl)carbamate**. Prepared according to a known procedure. All analytical data are in accordance with the literature.[19]

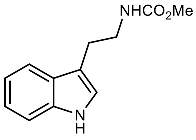

methyl (2-(1*H*-indol-3-yl)ethyl)carbamate. Prepared according to a known procedure. All analytical data are in accordance with the literature.[20]

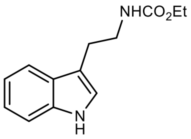

ethyl (2-(1*H*-indol-3-yl)ethyl)carbamate. Prepared according to a known procedure.[21] All analytical data are in accordance with the literature.[22]

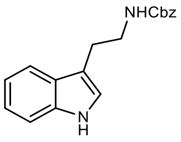

benzyl (2-(1*H*-indol-3-yl)ethyl)carbamate. Prepared according to a known procedure. All analytical data are in accordance with the literature.[23]

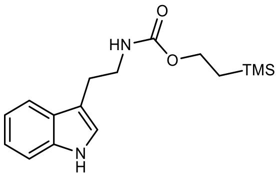

2-(trimethylsilyl)ethyl (2-(1*H*-indol-3-yl)ethyl)carbamate. To a suspension of tryptamine (200 mg, 1.25 mmol, 1.00 equiv.) in CH_2Cl_2 (10 mL) were added triethylamine (0.32 g, 0.44 mL, 3.1 mmol, 2.5 equiv.) and 1-[2-(trimethylsilyl)ethoxycarbonyloxy]pyrrolidin-2,5-dion (340 mg, 1.31 mmol, 1.05 equiv.). The mixture was stirred for two hours at room temperature and then diluted with brine (50 mL). The organic layer was separated and the aqueous layer was extracted with EtOAc (×2). The combined organic fractions were dried over $MgSO_4$ and the solvent was removed under reduced pressure. Chromatographic purification: silica gel, petroleum ether:EtOAc, 2:1, v/v. Yield: 379 mg, 1.25 mmol, 100%, colorless solid.

TLC (petroleum ether:EtOAc, 2:1 v/v): R_f = 0.33. **mp**: 70 °C (lit[24] 69.5–70.5 °C). **^{1}H-NMR** (400 MHz, $CDCl_3$): δ 8.16 (s_{br}, 1 H), 7.62 (d, *J* = 7.8 Hz, 1 H), 7.39–7.34 (m, 1 H), 7.21 (ddd, *J* = 8.2 Hz, 7.0 Hz, 1.2 Hz, 1 H), 7.13 (ddd, *J* = 8.0 Hz, 7.0 Hz, 1.1 Hz, 1 H), 7.02 (d, *J* = 2.2 Hz, 1 H), 4.82–4.49 (m, 1 H,), 4.16 (t, *J* = 8.4 Hz, 2 H), 3.58–3.41 (m, 2 H), 2.97 (t, *J* = 6.9 Hz, 2 H), 0.97 (t, *J* = 8.4 Hz, 2 H), 0.03 (s, 9 H). **^{13}C-NMR** (100 MHz, $CDCl_3$): δ 156.97, 136.50, 127.39, 122.19, 122.25, 119.54, 118.87, 113.05, 111.34, 63.03, 41.28, 17.87, 25.97, -1.43. **IR** (ATR): $\tilde{\nu}$ 3410, 3325, 2952, 2898, 1693, 1619, 1517, 1457, 1421, 1338, 1248, 1178, 1135, 1044, 1010, 976, 942, 857, 835, 766, 740, 694, 664, 610, 583, 483, 424. **HRMS** (ESI, m/z): $[M+H]^+$ calc. for $C_{16}H_{25}N_2O_2Si$, 305.169; found, 305.167.

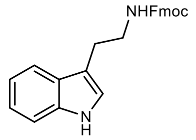

(9*H*-fluoren-9-yl)methyl (2-(1*H*-indol-3-yl)ethyl)carbamate. Prepared according to a known procedure. All analytical data are in accordance with the literature.[25]

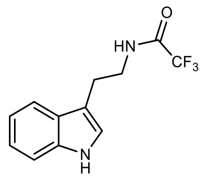

***N*-(2-(1*H*-indol-3-yl)ethyl)-2,2,2-trifluoroacetamide**. Prepared according to a known procedure. Additionaly purified by flash column chromatography: silica gel, petroleum ether:EtOAc, 3:1, v/v. **TLC**: R_f = 0.25. All analytical data are in accordance with the literature.[26]

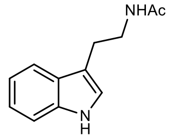

***N*-(2-(1*H*-indol-3-yl)ethyl)acetamide**. Prepared according to a known procedure. All analytical data are in accordance with the literature.[27]

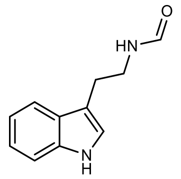

***N*-(2-(1*H*-indol-3-yl)ethyl)formamide**. Prepared according to a known procedure. All analytical data are in accordance with the literature.[28]

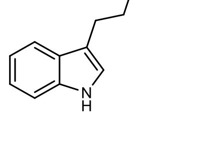

***N*-(2-(1*H*-indol-3-yl)ethyl)benzenesulfonamide**. Prepared according to a known procedure. All analytical data are in accordance with the literature.[29]

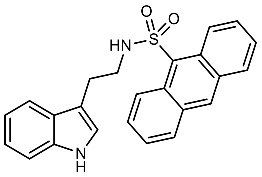

N-(2-(1H-indol-3-yl)ethyl)anthracene-9-sulfonamide. Prepared according to a known procedure.[30] Additionaly purified by flash column chromatography: silica gel, step gradient: petroleum ether:CH_2Cl_2, 1:1, v/v → CH_2Cl_2.

TLC (CH_2Cl_2): R_f = 0.40. **mp**: 167 °C. **^{1}H-NMR** (500 MHz, $CDCl_3$): δ 9.18 (d, *J* = 9.2 Hz, 2 H), 8.63 (s, 1 H), 8.01 (d, *J* = 7.7 Hz, 2 H), 7.80 (s_{br}, 1 H), 7.55 (ddd, *J* = 9.2 Hz, 6.5 Hz, 1.5 Hz, 2 H), 7.48 (ddd, *J* = 7.7 Hz, 6.5 Hz, 1.0 Hz, 2 H), 7.24 (dt, *J* = 8.2 Hz, 1.0 Hz, 1 H), 7.11 (ddd, *J* = 8.2 Hz, 7.0 Hz, 1.0 Hz, 1 H), 7.06 (d, *J* = 8.0 Hz, 1 H), 6.88 (ddd, *J* = 8.0 Hz, 7.0 Hz, 1.0 Hz, 1 H), 6.61 (d, *J* = 2.3 Hz, 1 H), 4.97 (t, *J* = 6.0 Hz, 1 H), 3.16 (q, *J* = 6.4 Hz, 2 H), 2.70 (t, *J* = 6.4 Hz, 2 H). **^{13}C-NMR** (125 MHz, $CDCl_3$): δ 136.36, 135.53, 131.26, 130.46, 129.57, 129.12, 128.81, 126.76, 125.40, 124.86, 122.41, 122.32, 119.61, 118.32, 111.40, 111.27, 43.11, 25.15. **IR** (ATR): $\tilde{\nu}$ 3406, 3051, 1622, 1518, 1456, 1447, 1420, 1405, 1319, 1229, 1159, 1144, 1073, 1027, 940, 906, 846, 823, 779, 736, 671, 647, 613, 583, 515, 480, 424. **HRMS** (ESI, m/z): $[M+H]^+$ calc. for $C_{24}H_{21}N_2O_2S$, 401.132; found, 401.128.

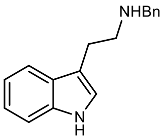

N-benzyl-2-(1H-indol-3-yl)ethan-1-amine. Prepared according to a known procedure. All analytical data are in accordance with the literature.[31]

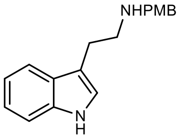

2-(1H-indol-3-yl)-N-(4-methoxybenzyl)ethan-1-amine. Prepared according to a known procedure. All analytical data are in accordance with the literature.[32]

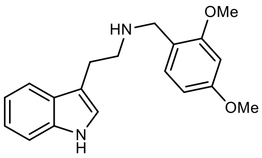

N-(2,4-dimethoxybenzyl)-2-(1H-indol-3-yl)ethan-1-amine. Prepared according to a known procedure. All analytical data are in accordance with the literature.[32]

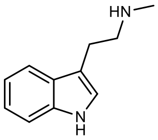

2-(1*H*-indol-3-yl)-*N*-methylethan-1-amine. Prepared according to a known procedure. All analytical data are in accordance with the literature.[22]

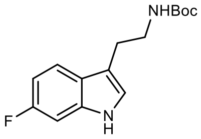

***tert*-butyl (2-(6-fluoro-1*H*-indol-3-yl)ethyl)carbamate**. Prepared according to a known general procedure[33] with 6-fluoro-1*H*-indole (203 mg, 1.50 mmol). Chromatographic purification: silica gel, petroleum ether:EtOAc, 4:1, v/v. **TLC**: R_f = 0.20. Yield: 191 mg, 0.687 mmol, 69%, colorless solid. All analytical data are in accordance with the literature.[34] **^{19}F-NMR** (565 MHz, $CDCl_3$): δ -121.14 (td, *J* = 9.6 Hz, 5.3 Hz).

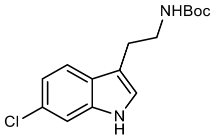

***tert*-butyl (2-(6-chloro-1*H*-indol-3-yl)ethyl)carbamate**. Prepared according to a known general procedure[33] with 6-chloro-1*H*-indole (227 mg, 1.50 mmol). Chromatographic purification: silica gel, petroleum ether:EtOAc, 4:1, v/v. **TLC**: R_f = 0.20. Yield: 221 mg, 0.750 mmol, 75%, colorless solid. All analytical data are in accordance with the literature.[35]

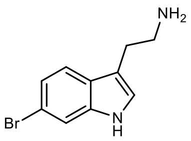

2-(6-bromo-1*H*-indol-3-yl)ethan-1-amine. Prepared according to a known procedure. All analytical data are in accordance with the literature.[36]

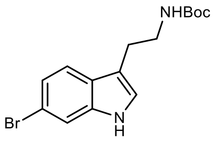

***tert*-butyl (2-(6-bromo-1*H*-indol-3-yl)ethyl)carbamate**. To a solution of 2-(6-bromo-1*H*-indol-3-yl)ethan-1-amine (1.31 g, 5.48 mmol, 1.00 equiv.) in CH_2Cl_2 (30 mL) were added NEt_3 (1.2 g, 1.7 mL, 12 mmol, 2.2 equiv.) and di-*tert*-butyldicarbonat (1.3 g, 1.3 mL, 5.8 mmol, 1.1 equiv.) and the solution was stirred for three hours at room temperature. Silica gel was added and the solvent removed under reduced pressure. Chromatographic purification: silica gel, petroleum ether:EtOAc, 3:1, v/v. **TLC**: R_f = 0.25. Yield: 1.58 g, 4.66 mmol, 85%, colorless solid. All analytical data are in accordance with the literature.[37] **mp**: 92 °C.

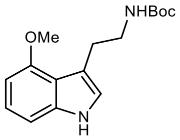

***tert*-butyl (2-(4-methoxy-1*H*-indol-3-yl)ethyl)carbamate**. Prepared according to a known general procedure[33] with 4-methoxy-1*H*-indole (221 mg, 1.50 mmol). Chromatographic purification: silica gel, petroleum ether:EtOAc, 4:1, v/v. Yield: 193 mg, 0.666 mmol, 67%, colorless solid.

TLC (petroleum ether:EtOAc, 4:1, v/v): R_f = 0.15. **mp**: 87 °C. **^{1}H-NMR** (400 MHz, $CDCl_3$): δ 8.17 (s_{br}, 1 H), 7.09 (t, *J* = 8.0 Hz, 1 H), 6.97 (d, *J* = 8.1 Hz, 1 H), 6.87 (s, 1 H), 6.49 (d, *J* = 7.7 Hz, 1 H), 4.86 (s_{br}, 1 H), 3.93 (s, 3 H), 3.46 (q, *J* = 6.3 Hz, 2 H), 3.05 (t, *J* = 6.3 Hz, 2 H), 1.41 (s, 9 H). **^{13}C-NMR** (100 MHz, $CDCl_3$): δ 156.31, 154.73, 138.34, 122.94, 121.31, 117.39, 113.73, 104.73, 99.49, 78.87, 55.18, 42.11, 28.55, 27.16. **IR** (ATR): $\tilde{\nu}$ 3409, 3323, 2975, 2933, 1690, 1617, 1587, 1508, 1456, 1437, 1392, 1365, 1254, 1167, 1123, 1084, 1049, 973, 780, 732. **HRMS** (ESI, m/z): $[M+Na]^+$ calc. for $C_{16}H_{22}N_2NaO_3$, 313.153; found, 313.153.

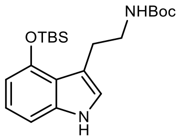

***tert*-butyl (2-(4-((*tert*-butyldimethylsilyl)oxy)-1*H*-indol-3-yl)ethyl)carbamate**. Prepared according to a known general procedure[33] with 4-((*tert*-butyldimethylsilyl)oxy)-1*H*-indole (371 mg, 1.50 mmol). Chromatographic purification: silica gel, petroleum ether:EtOAc, 4:1, v/v. Yield: 261 mg, 0.668 mmol, 67%, colorless solid.

TLC (petroleum ether:EtOAc, 4:1, v/v): R_f = 0.25. **mp**: 128 °C. **^{1}H-NMR** (500 MHz, $CDCl_3$): δ 8.13 (s_{br}, 1 H), 7.02–6.93 (m, 2 H), 6.88 (s, 1 H), 6.49 (dd, *J* = 7.4 Hz, 1.1 Hz, 1 H), 4.78 (s_{br}, 1 H), 3.45 (q, *J* = 5.9 Hz, 2 H), 3.09 (t, *J* = 6.6 Hz, 2 H), 1.41 (s, 9 H), 1.04 (s, 9 H), 0.34 (s, 6 H). **^{13}C-NMR** (125 MHz, $CDCl_3$): δ 156.24, 150.35, 139.01, 122.69, 121.31, 119.42, 113.51, 107.87, 104.73, 78.96, 42.09, 28.56, 26.82, 26.24, 18.74, -3.66. **IR** (ATR): $\tilde{\nu}$ 3410, 3326, 2955, 2930, 2896, 2858, 1693, 1615, 1581, 1503, 1472, 1435, 1392, 1364, 1354, 1253, 1168, 1067, 1048, 1005, 869, 837, 781, 733, 677. **HRMS** (ESI, m/z): $[M+Na]^+$ calc. for $C_{21}H_{34}N_2NaO_3Si$, 413.224; found, 413.224.

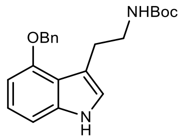

***tert*-butyl (2-(4-(benzyloxy)-1*H*-indol-3-yl)ethyl)carbamate**. Prepared according to a known general procedure[33] with 4-(benzyloxy)-1*H*-indole (335 mg, 1.50 mmol). Chromatographic purification: silica gel, petroleum ether:EtOAc, 4:1, v/v. Yield: 235 mg, 0.642 mmol, 64%, colorless solid.

TLC (petroleum ether:EtOAc, 4:1, v/v): R_f = 0.17. **mp**: 115 °C (lit[38] clear grey oil). **^{1}H-NMR** (500 MHz, $CDCl_3$): δ 8.09 (s_{br}, 1 H), 7.51–7.47 (m, 2 H), 7.44–7.39 (m, 2 H), 7.38–7.33 (m, 1 H), 7.09 (t, *J* = 7.9 Hz, 1 H), 6.99 (d, *J* = 8.1 Hz, 1 H), 6.88 (s, 1 H), 6.58 (d, *J* = 7.7 Hz, 1 H), 5.18 (s, 2 H), 4.54 (s_{br}, 1 H), 3.41–3.29 (m, 2 H), 3.02 (t, *J* = 6.9 Hz, 2 H), 1.40 (s, 9 H). **^{13}C-NMR** (125 MHz, $CDCl_3$): δ 156.21, 153.85, 138.45, 137.44, 128.75, 128.12, 127.74, 122.97, 121.36, 117.54, 113.84, 104.97, 100.59, 78.84, 70.12, 42.19, 28.57, 27.36. **IR** (ATR): $\tilde{\nu}$ 3455, 3288, 3004, 2973, 2934, 2905, 2861, 1697, 1620, 1587, 1507, 1453, 1382, 1364, 1344, 1312, 1269, 1241, 1165, 1140, 1085, 1071, 1054, 1025, 1002, 982, 955, 871, 842, 814, 771, 738, 729, 721, 695, 631, 610, 591, 533, 476, 432. **HRMS** (ESI, m/z): $[M+Na]^+$ calc. for $C_{22}H_{26}N_2NaO_3$, 389.184; found, 389.184.

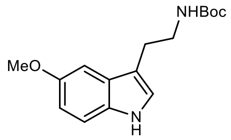

***tert*-butyl (2-(5-methoxy-1*H*-indol-3-yl)ethyl)carbamate**. Prepared according to a known general procedure[33] with 5-methoxy-1*H*-indole (221 mg, 1.50 mmol). Chromatographic purification: silica gel, petroleum ether:EtOAc, 4:1, v/v. **TLC**: R_f = 0.17. Yield: 250 mg, 0.862 mmol, 86%, light brown oil. All analytical data are in accordance with the literature.[39]

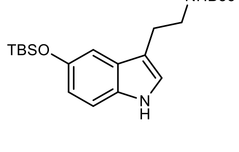

***tert*-butyl (2-(5-((*tert*-butyldimethylsilyl)oxy)-1*H*-indol-3-yl)ethyl)carbamate**. Prepared according to a known general procedure[33] with 5-((*tert*-butyldimethylsilyl)oxy)-1*H*-indole (371 mg, 1.50 mmol). Chromatographic purification: silica gel, petroleum ether:EtOAc, 4:1, v/v. **TLC**: R_f = 0.25. Yield: 293 mg, 0.751 mmol, 75%, colorless solid. All analytical data are in accordance with the literature.[40] **mp**: 93 °C. **HRMS** (ESI, m/z): $[M+Na]^+$ calc. for $C_{21}H_{34}N_2NaO_3Si$, 413.224; found, 413.224.

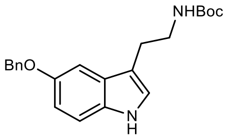

***tert*-butyl (2-(5-(benzyloxy)-1*H*-indol-3-yl)ethyl)carbamate**. Prepared according to a known general procedure[33] with 5-(benzyloxy)-1*H*-indole (335 mg, 1.50 mmol). Chromatographic purification: silica gel, petroleum ether:EtOAc, 4:1, v/v. **TLC**: R_f = 0.20. Yield: 304 mg, 0.829 mmol, 83%, yellowish oil. All analytical data are in accordance with the literature.[41]

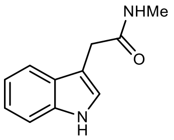

2-(1*H*-indol-3-yl)-*N*-methylacetamide. Prepared according to a known procedure. All analytical data are in accordance with the literature.[42]

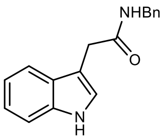

***N*-benzyl-2-(1*H*-indol-3-yl)acetamide**. Prepared according to a known procedure. All analytical data are in accordance with the literature.[43]

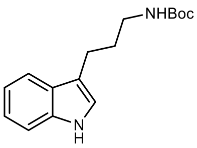

***tert*-butyl (3-(1*H*-indol-3-yl)propyl)carbamate**. Prepared according to a known procedure. All analytical data are in accordance with the literature.[44]

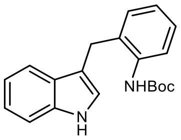

***tert*-butyl (2-((1*H*-indol-3-yl)methyl)phenyl)carbamate**. Prepared according to a known procedure. All analytical data are in accordance with the literature.[45]

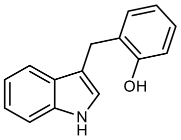

2-((1*H*-indol-3-yl)methyl)phenol. Prepared according to a known procedure. All analytical data are in accordance with the literature.[45]

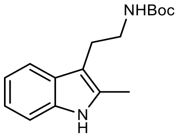

***tert*-butyl (2-(2-methyl-1*H*-indol-3-yl)ethyl)carbamate**. Prepared according to a known general procedure[33] with 2-methyl-1*H*-indole (197 mg, 1.50 mmol). Chromatographic purification: silica gel, petroleum ether:EtOAc, 4:1, v/v. Yield: 122 mg, 0.443 mmol, 44%, orange-brown solid.

TLC (petroleum ether:EtOAc, 4:1, v/v): $R_f = 0.25$. **mp**: 95–97 °C (lit[46] yellow oil). **HRMS** (ESI, m/z): $[M+Na]^+$ calc. for $C_{16}H_{22}N_2NaO_2$, 297.158; found, 297.157. The spectroscopic data are in accordance with the literature.[46]

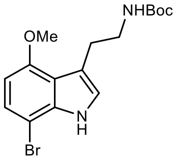

***tert*-butyl (2-(7-bromo-4-methoxy-1*H*-indol-3-yl)ethyl)carbamate**. Prepared according to a known general procedure[33] with 7-bromo-4-methoxy-1*H*-indole (339 mg, 1.50 mmol). Chromatographic purification: silica gel, petroleum ether:EtOAc, 4:1, v/v. Yield: 224 mg, 0.607 mmol, 61%, colorless solid.

TLC (petroleum ether:EtOAc, 4:1, v/v): $R_f = 0.20$. **mp**: 173 °C. **^{1}H-NMR** (400 MHz, $CDCl_3$): δ 8.20 (s_{br}, 1 H), 7.20 (d, J = 8.3 Hz, 1 H), 6.95 (s, 1 H), 6.39 (d, J = 8.3 Hz, 1 H), 4.77 (s_{br}, 1 H), 3.91 (s, 3 H), 3.44 (q, J = 6.4 Hz, 2 H), 3.02 (t, J = 6.7 Hz, 2 H), 1.40 (s, 9 H). **^{13}C-NMR** (100 MHz, $CDCl_3$): δ 156.20, 154.27, 136.31, 124.77, 121.72, 118.39, 115.27, 101.05, 96.51, 79.02, 55.48, 41.91, 28.54, 27.18. **IR** (ATR): $\tilde{\nu}$ 3244, 2971, 2914, 1694, 1495, 1455, 1433, 1366, 1337, 1286, 1271, 1252, 1231, 1166, 1129, 1096, 1080, 1057, 1030, 978, 848, 778, 760, 679, 620, 556, 474, 433. **HRMS** (ESI, m/z): $[M+Na]^+$ calc. for $C_{16}H_{21}BrN_2NaO_3$, 391.063; found, 391.062.

7. Evaluation of the reaction conditions

Structures of phosphoramidite ligands

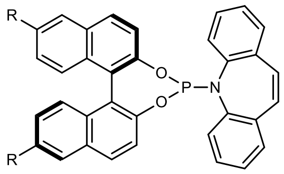

(*R*)-**L1** R = H
(*R*)-**L2** R = Br
(*R*)-**L3** R = Ph

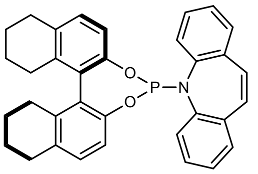

(*R*)-**L4**

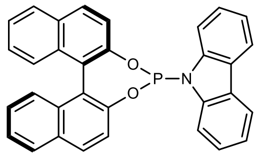

(*R*)-**L5**

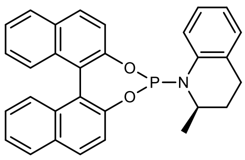

(R_a,*R*)-**L6**
(S_a,*R*)-**L6**

Structures of boranes

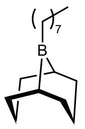

B-octyl-9-BBN

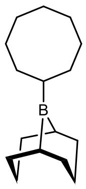

B-cyclooctyl-9-BBN

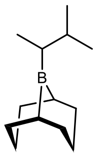

(*rac*)-*B*-siamyl-9-BBN

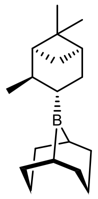

B-(ipc)-9-BBN
(*R*)-Alpine-Borane®

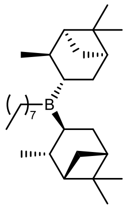

B-(ipc)$_2$octyl

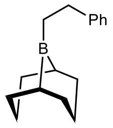

B-(2-phenylethyl)-9-BBN

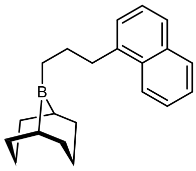

B-(3-(1-naphthyl)propyl)-9-BBN

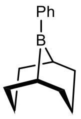

B-phenyl-9-BBN

7.1. Supplementary Table 1

Supplementary Table 1: Evaluation of the reaction conditions for the reverse prenylation with iridium-bis-phosphoramidite-complexes. All reactions were performed according to **GP 6**. [a] Isolated yield. [b] Determined by chiral HPLC. [c] **1a** (10 equiv.).

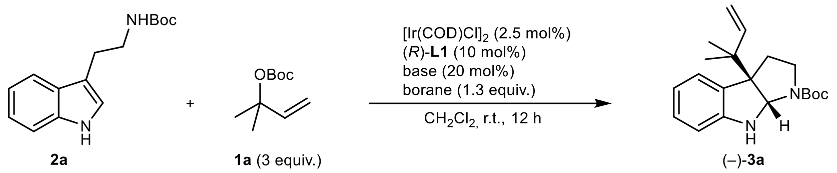

entry	borane	base	yield[a]	*ee*[b]
1	BEt_3	DBU	99 %	13%
2	*B*-*n*-octyl-9-BBN	DBU	99%	–61%
3	*B*-*n*-octyl-9-BBN	MTBD	99%	–61%
4	*B*-*n*-octyl-9-BBN	TBD	60%	–62%
5	*B*-cyclooctyl-9-BBN	DBU	15%	13%
6	(*rac*)-*B*-siamyl-9-BBN	DBU	9%	–20%
7	*B*-(ipc)-9-BBN	DBU	traces	n.d.
8	*B*-$(ipc)_2$-*n*-octyl	DBU	0 %	-
9	*B*-(2-phenylethyl)-9-BBN	DBU	99%	–57%
10	*B*-(3-(1-naphthyl)propyl)-9-BBN	DBU	11 %	–61%
11	*B*-phenyl-9-BBN	DBU	82 %	–35%
12	BPh_3	DBU	15 %, 37%[c]	94%
13	BPh_3	MTBD	37%[c]	94%
14	BPh_3	TBD	48%[c]	94%
15	B-$(1\text{-naphthyl})_3$	DBU	0%	-

7.2. Supplementary Table 2

Supplementary Table 2: Evaluation of the reaction conditions for the reverse prenylation with *in-situ* generated iridium-NHC-phosphoramidite-complexes. All reactions were performed according to **GP 7** with the denoted solvent. [a] Isolated yield. [b] Determined by chiral HPLC.

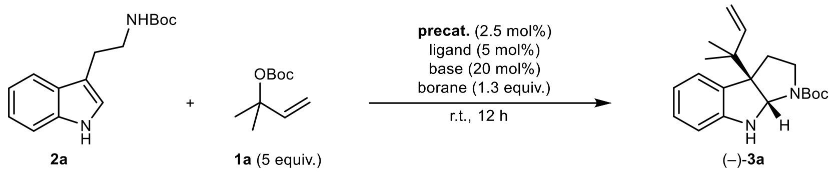

entry	catalyst	ligand	base	borane	solvent	yield[a]	*ee*[b]
1	**precat. 1**	(*R*)-**L1**	DBU	BPh_3	CH_2Cl_2	39%	91%
2	**precat. 1**	(*R*)-**L1**	TBD	BPh_3	CH_2Cl_2	94%	92%
3	**precat. 1**	(*R*)-**L1**	MTBD	BPh_3	CH_2Cl_2	93%	94%
4	**precat. 1**	(*R*)-**L1**	MTBD	BPh_3	$CHCl_3$	100%	95%
5	**precat. 1**	(*R*)-**L1**	MTBD	BPh_3	benzene	100%	97%
6	**precat. 2**	(*R*)-**L1**	MTBD	BPh_3	benzene	76%	88%
7	**precat. 3**	(*R*)-**L1**	MTBD	BPh_3	benzene	100%	97%
8	**precat. 4**	(*R*)-**L1**	MTBD	BPh_3	benzene	100%	98%
9	**precat. 1**	(*R*)-**L2**	MTBD	BPh_3	benzene	100%	97%
10	**precat. 1**	(*R*)-**L3**	MTBD	BPh_3	benzene	100%	97%
11	**precat. 1**	(*R*)-**L4**	MTBD	BPh_3	benzene	98%	89%
12	**precat. 1**	(*R*)-**L5**	MTBD	BPh_3	benzene	100%	71%
13	**precat. 1**	(R_a,*R*)-**L6**	MTBD	BPh_3	benzene	100%	64%
14	**precat. 1**	(S_a,*R*)-**L6**	MTBD	BPh_3	benzene	100%	–60%

8. Procedures for the catalyst preparation and reverse prenylation

8.1. General procedure for the preparation of precatalysts (GP 4)

A solution of $[Ir(COD)Cl]_2$ (67 mg, 0.1 mmol, 1 equiv.) and the imidazolium chloride (0.2 mmol, 2 equiv.) in dichloromethane (2 mL) was stirred for 30 minutes at room temperature whereupon the solvent was removed in high vacuum. The products are reasonably stable in air but have to be kept under nitrogen for prolonged storage.

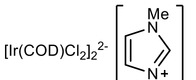

precat. 1

(3-benzyl-1-methyl-1*H*-imidazol-3-ium)-di-*μ*-chlorodichlorobis[(1,2,5,6-*η*)-1,5-cyclooctadiene]di-iridat(I). Prepared according to **GP 4** with 3-benzyl-1-methyl-1*H*-imidazol-3-ium chloride (**im-a**).

^{1}H-NMR (400 MHz, CD_2Cl_2): δ 10.12 (s, 2 H), 7.54–7.38 (m, 10 H), 7.20 (t, *J* = 1.8 Hz, 2 H), 7.15 (t, *J* = 1.8 Hz, 2 H), 5.59 (s, 4 H), 4.04 (s, 6 H), 3.98–3.68 (m, 8 H), 2.22–1.99 (m, 8 H), 1.44–1.20 (m, 8 H). **^{13}C-NMR** (100 MHz, CD_2Cl_2): δ 138.6,[a] 133.8, 129.7, 129.7, 129.4, 123.5, 121.7, 60.5 (broad), 53.7, 37.2, 32.3.

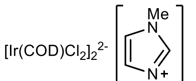

precat. 2

(1,3-dimethyl-1*H*-imidazol-3-ium)-di-μ-chlorodichlorobis[(1,2,5,6-η)-1,5-cyclooctadiene]di-iridat(I). Prepared according to **GP 4** with 1,3-dimethyl-1*H*-imidazol-3-ium chloride (**im-b**).

^{1}H-NMR (600 MHz, $CDCl_3$): δ 10.18 (s, 2 H), 7.22 (d, *J* = 1.6 Hz, 4 H), 4.08 (s, 12 H), 3.95–3.87 (m, 8 H), 2.23–2.10 (m, 8 H), 1.40–1.29 (m, 8 H). **^{13}C-NMR** (150 MHz, $CDCl_3$): δ 138.9,[b] 122.9, 60.6, 37.0, 32.1.

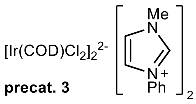

precat. 3

(1-methyl-3-phenyl-1*H*-imidazol-3-ium)-di-μ-chlorodichlorobis[(1,2,5,6-η)-1,5-cyclooctadiene]di-iridat(I). Prepared according to **GP 4** with 1-methyl-3-phenyl-1*H*-imidazol-3-ium chloride (**im-c**).

---

a The signal was only observed in the ^{1}H,^{13}C HMBC-NMR spectrum.
b The signal was only observed in the ^{1}H,^{13}C HMBC-NMR spectrum.

^{1}H-NMR (500 MHz, $CDCl_3$): δ 10.43 (s, 2 H), 7.81–7.77 (m, 4 H), 7.61 (t, *J* = 1.9 Hz, 2 H), 7.59–7.54 (m, 6 H), 7.53–7.49 (m, 2 H), 4.25 (s, 6 H), 3.93–3.84 (m, 8 H), 2.18–2.04 (m, 8 H), 1.39–1.25 (m, 8 H). **^{13}C-NMR** (125 MHz, $CDCl_3$): δ 137.3, 134.6, 130.6, 130.4, 124.3, 122.4, 120.6, 60.4, 37.9, 32.1.

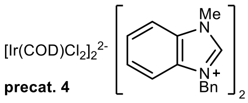

precat. 4

(3-benzyl-1-methyl-1*H*-benzo[*d*]imidazol-3-ium)-di-µ-chlorodichlorobis[(1,2,5,6-η)-1,5-cycloocta-diene]diiridat(I). Prepared according to **GP 4** with 3-benzyl-1-methyl-1*H*-benzo[*d*]imidazol-3-ium chloride (**im-d**).

^{1}H-NMR (400 MHz, $CDCl_3$): δ 11.19 (s, 2 H), 7.68–7.48 (m, 12 H), 7.42–7.30 (m, 6 H), 5.92 (2, 4 H), 4.30 (s, 6 H), 4.06–3.89 (m, 8 H), 2.26–2.07 (m, 8 H), 1.44–1.25 (m, 8 H). **^{13}C-NMR** (100 MHz, $CDCl_3$): δ 144.5, 133.1, 132.4, 131.2, 129.5, 129.4, 128.6, 127.2, 127.1, 113.7, 112.8, 60.8, 51.6, 34.0, 32.1.

8.2. General procedure for the preparation of iridium-NHC-phosphoramidite complexes (GP 5)

The reactions were performed with the exclusion of light. To a solution of the respective imidazolium chloride (2 equiv.) in CH_2Cl_2 (20 mL/mmol) was added finely powdered silver(I)oxide (1 equiv.) and the suspension was stirred for 2 hours at room temperature whereupon $[Ir(COD)Cl]_2$ (1 equiv.) was added and stirred for 12 hours. The suspension was filtered over a plug of silica gel which was washed with CH_2Cl_2/Et_2O (1/1). The filtrates were combined and the solvent removed under reduced pressure. The residue was purified by flash column chromatography. The obtained Ir-NHC-complex **K-1a**/**K-1b** (1 equiv.) and (*R*)-**L1** (1 equiv.) were dissolved in benzene (10 mL/mmol) and stirred at 50 °C for 24 hours whereupon the solvent was removed in high vacuum. The obtained complex was purified by vapor diffusion crystallization (using a two-chambered Schlenk-flask) at room temperature (18–20 °C) under a nitrogen atmosphere from a saturated solution in benzene with hexane as the anti-solvent. After complete precipitation (48 hours) the supernatant and residual hexane were revomed via syringe. The complex was washed with hexane (×2) and dried in high vacuum.

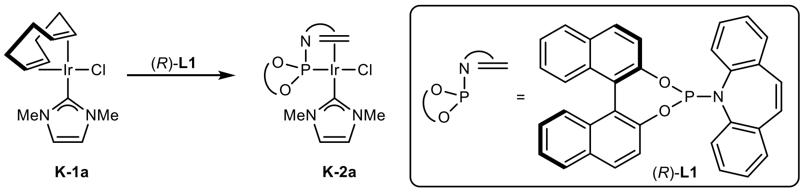

K-1a: Prepared according to **GP 5** with 1,3-dimethyl-1*H*-imidazol-3-ium chloride (**im-b**) (222 mg, 1.67 mmol, 2 equiv.), silver(I)oxide (193 mg, 0.835 mmol, 1 equiv.), $[Ir(COD)Cl]_2$ (561 mg, 0.835 mmol,

1 equiv.). Chromatographic purification: silica gel, CH_2Cl_2:Et_2O, 9:1, v/v. Yield: 373 mg, 0.864 mmol, 52%, yellow solid.

TLC (CH_2Cl_2:Et_2O, 9:1, v/v): R_f = 0.50. **mp**: 205 °C (decomposition). **^{1}H-NMR** (500 MHz, $CDCl_3$): δ 6.80 (s, 2 H), 4.62–4.54 (m, 2 H), 3.95 (s, 6 H), 2.97–2.90 (m, 2 H), 2.27–2.15 (m, 4 H), 1.79–1.69 (m, 2 H), 1.65–1.55 (m, 2 H). **^{13}C-NMR** (125 MHz, $CDCl_3$): δ 180.80, 121.71, 84.57, 51.41, 37.50, 33.71, 29.72. **IR** (ATR): $\tilde{\nu}$ 3155, 3119, 3099, 2998, 2978, 2922, 2860, 2835, 1568, 1462, 1443, 1394, 1385, 1353, 1322, 1298, 1226, 1171, 1156, 1131, 1082, 1014, 997, 968, 906, 881, 862, 843, 820, 808, 777, 733, 703, 685, 610, 598, 520, 508, 480, 462.

K-2a: Prepared according to **GP 5** with **K-1a** (353 mg, 0.817 mmol, 1 equiv.), (*R*)-**L1** (415 mg, 0.817 mmol, 1 equiv.). Yield: 544 mg, 0.655 mmol, 80%, red solid.

mp: 260 °C (decomposition). **$[\alpha]_D^{24}$**: –278 (1.0, CH_2Cl_2). **^{1}H-NMR** (400 MHz, $CDCl_3$): δ 7.99 (d, *J* = 8.9 Hz, 1 H), 7.96 (d, *J* = 8.2 Hz, 1 H), 7.72 (d, *J* = 8.2 Hz, 1 H), 7.67 (dd, *J* = 7.6 Hz, 1.6 Hz, 1 H), 7.64 (dd, *J* = 7.3 Hz, 1.8 Hz, 1 H), 7.55 (d, *J* = 8.8 Hz, 1 H), 7.46–7.30 (m, 5 H), 7.24–7.06 (m, 8 H), 7.01 (d, *J* = 8.9 Hz, 1 H), 6.38 (d, *J* = 1.8 Hz, 1 H), 5.69 (d, *J* = 1.8 Hz, 1 H), 5.17 (d, *J* = 9.1 Hz, 1 H), 5.11 (d, *J* = 9.1 Hz, 1 H), 3.94 (s, 3 H), 3.11 (s, 3 H). **^{13}C-NMR**[c] (100 MHz, $CDCl_3$): δ 174.88_q (d, *J* = 14.6 Hz), 148.36_q (d, *J* = 14.2 Hz), 146.83_q (d, *J* = 5.7 Hz), 142.50_q (d, *J* = 5.5 Hz), 142.18_q (d, *J* = 7.1 Hz), 141.81_q (d, *J* = 7.4 Hz), 141.42_q (d, *J* = 7.3 Hz), 132.55_q (d, *J* = 16.2 Hz), 132.53_q (d, *J* = 16.2 Hz), 131.11_q (d, *J* = 29.6 Hz), 130.45 (d, *J* = 4.1 Hz), 129.89*, 129.85*, 129.20, 128.48*, 128.44*, 128.17*, 127.98*, 127.66*, 127.64*, 127.43*, 127.35*, 127.15*, 126.97*, 126.71*, 126.28, 125.17, 123.01_q (d, *J* = 2.8 Hz), 122.19 (d, *J* = 1.4 Hz), 121.87, 121.67_q (d, *J* = 2.8 Hz), 121.38 (d, *J* = 2.3 Hz), 121.09, 65.54, 64.45, 38.81, 37.59. **^{31}P-NMR** (162 MHz, $CDCl_3$): δ 132.69. **IR** (ATR): $\tilde{\nu}$ 3370, 2973, 2930, 1679, 1636, 1608, 1590, 1493, 1470, 1405, 1393, 1367, 1336, 1285, 1242, 1162, 1127, 1078, 1046, 1014, 916, 854, 748, 518, 462. **HRMS** (ESI, m/z): $[M+H]^+$ calc. for $C_{39}H_{31}ClIrN_3O_2P$, 832.1472; found, 832.1462 (19% intensity). $[M-Cl^-]^+$ calc. for $C_{39}H_{30}IrN_3O_2P$, 796.1705; found, 796.1704 (100% intensity).

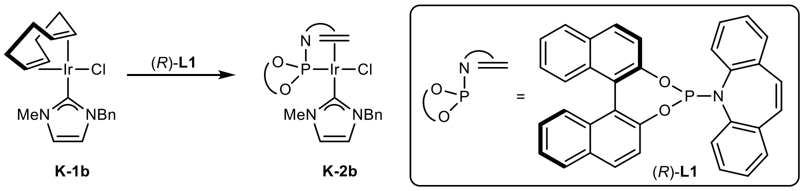

K-1b: Prepared according to **GP 5** with with 3-benzyl-1-methyl-1*H*-imidazol-3-ium chloride (**im-a**) (509 mg, 2.44 mmol, 2 equiv.), silver(I)oxide (283 mg, 1.22 mmol, 1 equiv.), $[Ir(COD)Cl]_2$ (819 mg,

---

c Signals of quartenary carbons are denoted by the subscript *q*. Sinals of C_{aryl}-H that are overlapping or possibly arise from a carbon-phosphorous-coupling are marked with an asterisk and are reported as singlets. The NMR spectra are pictured in section 12.1.

1.22 mmol, 1 equiv.). Chromatographic purification: silica gel, petroleum ether:EtOAc, 3:1, v/v. Yield: 1.07 g, 2.09 mmol, 86%, yellow solid.

TLC (petroleum ether:EtOAc, 3:1, v/v): R_f = 0.25. **mp**: 166 °C. **^{1}H-NMR** (500 MHz, $CDCl_3$): δ 7.37–7.27 (m, 5 H), 6.80 (d, *J* = 2.0 Hz, 1 H), 6.66 (d, *J* = 2.0 Hz, 1 H), 5.76 (d, *J* = 14.8 Hz, 1 H), 5.53 (d, *J* = 14.8 Hz, 1 H), 4.65–4.57 (m, 2 H), 3.98 (s, 3 H), 3.02 (td, *J* = 7.0 Hz, 2.7 Hz, 1 H), 2.88 (td, *J* = 7.3 Hz, 3.2 Hz, 1 H), 2.30–2.14 (m, 3 H), 2.12–2.04 (m, 1 H), 1.79–1.60 (m, 3 H), 1.56–1.47 (m, 1 H). **^{13}C-NMR** (125 MHz, $CDCl_3$): δ 180.86, 136.47, 128.95, 128.33, 128.22, 122.23, 120.13, 85.01, 84.63, 54.26, 51.81, 51.61, 37.60, 34.00, 33.34, 29.89, 29.39. **IR** (ATR): ṽ 3156, 3097, 3003, 2971, 2948, 2922, 2864, 2835, 1737, 1569, 1453, 1429, 1398, 1356, 1324, 1241, 1226, 1188, 1080, 1032, 996, 968, 959, 882, 863, 841, 818, 779, 735, 725, 708, 701, 690, 610, 597, 578, 520, 506, 479, 468, 445, 407, 390, 378.

K-2b: Prepared according to **GP 5** with **K-1b** (440 mg, 0.864 mmol, 1 equiv.), (*R*)-**L1** (438 mg, 0.864 mmol, 1 equiv.). Yield: 777 mg, 0.855 mmol, 99%, red solid.

mp: 240 °C (decomposition). **$[\alpha]_D^{24}$**: –382 (1.0, CH_2Cl_2). **^{1}H-NMR** (600 MHz, CD_2Cl_2): δ 8.04–6.79 (m, 25 H), 6.63 (d, *J* = 1.9 Hz, 1 H_{minor}), 6.47 (d, *J* = 1.9 Hz, 1 H_{major}), 5.80 (d, *J* = 8.9 Hz, 1 H_{minor}), 5.74 (d, *J* = 14.3 Hz, 1 H_{minor}), 5.73 (d, *J* = 1.9 Hz, 1 H_{minor}), 5.64 (d, *J* = 1.9 Hz, 1 H_{major}), 5.58 (d, *J* = 14.3 Hz, 1 H_{minor}), 5.41 (d, *J* = 14.6 Hz, 1 H_{major}), 5.36 (d, *J* = 9.1 Hz, 1 H_{minor}), 5.20 (d, *J* = 9.1 Hz, 1 H_{major}), 5.11 (d, *J* = 9.1 Hz, 1 H_{major}), 5.03 (d, *J* = 9.1 Hz, 1 H_{minor}), 4.01 (d, *J* = 14.6 Hz, 1 H_{major}), 3.96 (s, 3 H_{major}), 2.90 (s, 3 H_{minor}). **^{13}C-NMR**[d] (150 MHz, CD_2Cl_2): δ $175.43_{q, major}$ (d, *J* = 14.5 Hz), $174.76_{q, minor}$ (d, *J* = 14.8 Hz), $148.72_{q, major}$ (d, *J* = 14.0 Hz), $148.44_{q, minor}$ (d, *J* = 14.0 Hz), $147.03_{q, major}$ (d, *J* = 5.6 Hz), $146.92_{q, minor}$ (d, *J* = 5.9 Hz), $143.08_{q, minor}$ (d, *J* = 5.7 Hz), $142.85_{q, major}$ (d, *J* = 5.5 Hz), $142.4–142.2_{q, major+minor}$ (2 × d), $142.1–141.9_{q, major+minor}$ (2 × d), $141.76_{q, major}$ (d, *J* = 7.0 Hz), $141.62_{q, minor}$ (d, *J* = 6.2 Hz), $136.79_{q, minor}$, $136.68_{q, major}$, 132.81_q^*, 132.75_q^*, 132.66_q^*, 131.63_q^*, $131,59_q^*$, 131.41_q^*, 131.06*, 131.04*, 131.01*, 130.84*, 130.81*, 130.41*, 130.22*, 130.08*, 129.98*, 129.96*, 129.89*, 129.82*, 129.04*, 128.84*, 128.74*, 128.70*, 128.68*, 128.56*, 128.48*, 128.30*, 128.27*, 128.16*, 128.08*, 128.01*, 127.98*, 127.90*, 127.86*, 127.76*, 127.70*, 127.42*, 127.20*, 127.17*, 126.93*, 126.82*, 126.70*, 126.64*, 126.59*, 126.57*, 125.60*, 125.58*, 125.55*, $123.25_{q, major}$ (d, *J* = 2.5 Hz), 122.75, $122.71_{q, minor}$, 122.57, 122.52, $121.91_{q, major}$ (d, *J* = 2.4 Hz), 121.88, $121.79_{q, minor}$ (d, *J* = 2.4 Hz), 121.36, 120.99, 120.92, 119.77_{major}, 66.35_{minor}, 65.87_{major}, 64.57_{major}, 63.08_{minor}, 54.98_{minor}, 54.27_{major}, 39.06_{major}, 37.87_{minor}. **^{31}P-NMR** (243 MHz, CD_2Cl_2): δ 134.06_{major}, 132.93_{minor}. **IR** (ATR): ṽ 3031, 2927, 1588, 1505, 1477, 1455, 1432, 1401, 1358, 1321, 1226, 1203, 1189, 1154, 1110, 1069, 1033, 984, 949, 930, 907, 865, 853, 828, 794, 749, 730, 712, 696, 676, 650, 631, 619, 601, 577, 566, 539, 526, 504, 472, 424. **HRMS** (ESI, m/z): $[M+H]^+$ calc. for $C_{45}H_{35}ClIrN_3O_2P$, 908.1785; found, 908.1776 (20% intensity). $[M-Cl^-]^+$ calc. for $C_{45}H_{34}IrN_3O_2P$, 872.2018; found, 872.2025 (72% intensity).

---

[d] Signals of quartenary carbons are denoted by the subscript *q*. Sinals that are overlapping or possibly arise from a carbon-phosphorous-coupling are marked with an asterisk and are reported as singlets. The NMR spectra are pictured in section 12.2.

8.3. General procedure for the reverse prenylation with iridium-bis-phosphoramidite catalyst (GP 6)

The catalyst was prepared by stirring a solution of $[Ir(COD)Cl]_2$ (1.7 mg, 2.5 µmol, 2.5 mol%) and (*R*)-**L1** (5.1 mg, 10 µmol, 10 mol%) in CH_2Cl_2 (0.5 mL) for 15 minutes. To a solution of the tryptamine derivative (0.1 mmol, 1 equiv.) in CH_2Cl_2 (1 mL) were added successively the borane (1.3 equiv.; BEt_3: 1 M in hexane; BPh_3 as solid; other boranes: 1 M in CH_2Cl_2) and the amidine base (DBU/TBD/MTBD: 20 mol%). After stirring for 10 minutes the beforehand prepared solution of the iridium catalyst was added, followed by the allylic substrate **1a**–**e** (0.30 mmol, 3.0 equiv.). After stirring for 20 hours silica gel (100 mg) was added and the solvent removed under reduced pressure. The products were purified by flash column chromatography.

8.4. General procedure for the reverse prenylation with *in-situ* generated iridium-NHC-phosphoramidite catalyst (GP 7)

The catalyst was prepared by adding the amidine base (DBU/TBD/MTBD) to a solution of **precat. 1**–**4** and the phosphoramidite ligand (**L1**–**L6**) in benzene (0.5 mL) and stirring for 15 minutes. To a solution of the tryptamine derivative (0.1 mmol, 1 equiv.) in benzene (1 mL) was added the borane (triarylboranes as solid; *B*-octyl-9-BBN: 1 M in benzene). To this solution were successively added the beforehand prepared solution of the iridium catalyst and the allylic substrate **1a**–**1e** (0.50 mmol, 5.0 equiv.). After stirring for 20 hours silica gel (100 mg) was added and the solvent removed under reduced pressure. The products were purified by flash column chromatography.

8.5. General procedure for the reverse prenylation with isolated iridium-NHC-phosphoramidite catalyst (GP 8)

To a solution of *N*-Boc-tryptamine **2a** (26 mg, 0.1 mmol, 1 equiv.) and BPh_3 (31 mg, 0.13 mmol, 1.3 equiv.) in benzene (1.5 mL) were added successively MTBD (3 mg, 3 µL, 0.02 mmol, 20 mol%), **K-2b** (4.5 mg, 5 µmol, 5 mol%), tetrabutylammonium halide (if applicable 10–100 mol%, 1 M in CH_2Cl_2) and the allylic substrate **1a** or (*R*)-**1e** (5–10 equiv.). After stirring for 20 hours silica gel (100 mg) was added and the solvent removed in high vacuum. The product was purified by flash column chromatography.

9. allyl complex K-3a

9.1. Preparation of K-3a

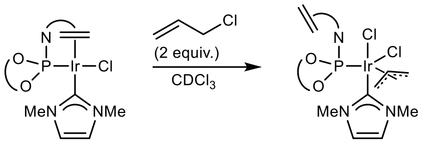

K-2a **K-3a**

Allyl chloride[e] (8.1 mg, 7.7 µL, 100 µmol, 2.0 equiv.) was added to a solution of **K-2a** (42.5 mg, 50.0 µmol, 1.00 equiv.) in $CDCl_3$ (1 mL), and the mixture was stirred for two minutes. The solution was then transferred to a Young NMR tube and analyzed by NMR at 300 K and 243 K. Using the same procedure in CD_2Cl_2, the ^{13}C-resonances of the minor allyl isomer were identified. Due to their complexity, the NMR spectra are presented in detail in Section 12.3. The evaluation of the allyl resonances is included within the spectra.

HRMS (ESI, m/z): [(**K-3a**)–Cl^-]$^+$ calc. for $C_{42}H_{35}ClIrN_3O_2P$, 872.1785; found, 872.1796.

The molecular ion peak of **K-3a** was not observed.

9.2. Chloride abstraction from K-3a

K-3a (45 mg, 50 µmol, 1.0 equiv.) was prepared as described above in $CDCl_3$ (1 mL). The solution was diluted with hexane (1 mL), and the solvent along with any residual allyl chloride was removed under high vacuum, yielding **K-3a** as an off-white crystalline solid, which was then re-dissolved in $CDCl_3$ (1 mL).[f] A 0.1 M solution of $AgSbF_6$ (0.50 mL, 50 µmol, 1.0 equiv.) in CD_2Cl_2 was added at –20 °C. After two minutes, the solution was drawn into a syringe at room temperature, at which point silver chloride precipitated immediately. After five minutes, 1 mL of the solution was transferred to a Young-NMR tube via a syringe filter and subjected to NMR analysis.

The ^{31}P-NMR spectrum (see below) displayed four major signals (49.2, 48.9, 43.9, 42.8 ppm), indicating that either chloride ligand of **K-3a** can be displaced, resulting in two diastereomeric complexes, both of which exist as mixtures of *exo/endo*-isomers. Due to *syn/anti*-isomerization, two of the signals are broadened.

[e] Allyl chloride was dried over 4 Å molecular sieves and distilled under a nitrogen atmosphere.
[f] A control NMR analysis confirmed that **K-3a** remained fully intact after isolation.

^{31}P-NMR (243 MHz, $CDCl_3$, 298 K) of [(**K-3a**) – $Cl^–$]

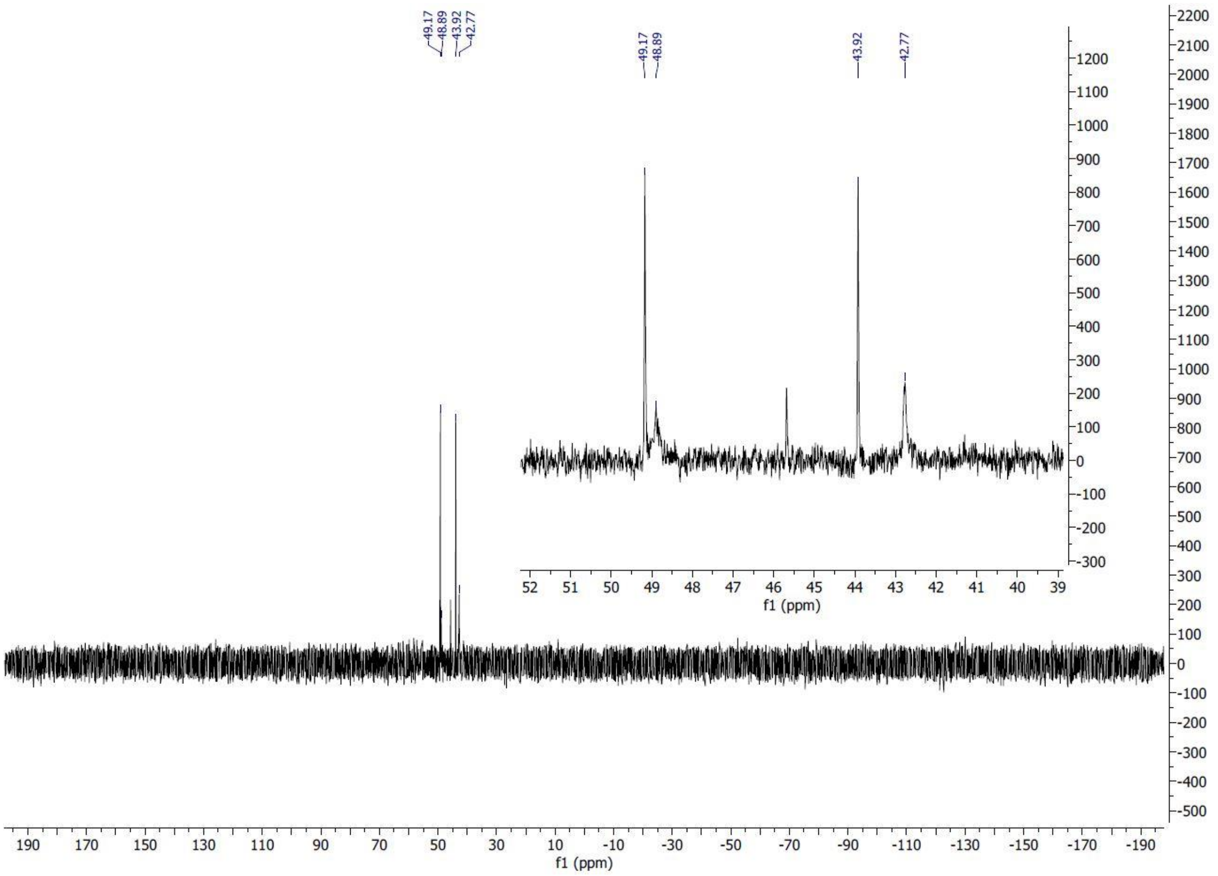

10. Reverse prenylation of Tryptamines and Other 3-substituted Indoles

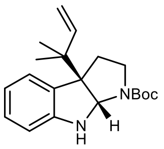

***tert*-butyl (3a*R*,8a*S*)-3a-(2-methylbut-3-en-2-yl)-3,3a,8,8a-tetrahydropyrrolo[2,3-*b*]-indole-1(2*H*)-carboxylate**

(–)-3a

Prepared according to **GP 7**: *N*-Boc-Tryptamine (26.0 mg, 0.100 mmol, 1.00 equiv.), **precat. 4** (3.0 mg, 2.5 µmol, 2.5 mol%), (*R*)-**L1** (2.5 mg, 5.0 µmol, 5.0 mol%), MTBD (3 mg, 3 µL, 20 µmol, 20 mol%), BPh_3 (31 mg, 0.13 mmol, 1.3 equiv.), **1a** (93 mg, 97 µL, 0.50 mmol, 5.0 equiv.). Chromatographic purification: silica gel, petroleum ether:EtOAc:NEt_3, 98.5:1:0.5, v/v. Yield: 32.8 mg, 0.100 mmol, 100%, colorless solid. **mp**: 97 °C (lit[47] 115–118 °C, *ee* = 99%). **HPLC** (AD-H, hexane:2-propanol, 9:1, v/v, 1.0 mL/min, 254 nm): t_R = 4.7 min (major), 7.2 min (minor), *er* = 80.1/1.00, *ee* = 97.5%. **$[\alpha]_D^{20}$**: –384 (1.0, CH_2Cl_2), lit.[47] $[\alpha]_D^{21}$: –361 (0.13, MeOH).

Prepared according to **GP 6**: *N*-Boc-Tryptamine (26.0 mg, 0.100 mmol, 1.00 equiv.), BEt_3 (0.13 mL, 1.3 equiv.), DBU (3 mg, 3 µL, 20 µmol, 20 mol%), **1a** (56 mg, 59 µL, 0.30 mmol, 3.0 equiv.). Chromatographic purification: silica gel, petroleum ether:EtOAc:NEt_3, 98.5:1:0.5, v/v. Yield: 32.5 mg, 0.0991 mmol, 99%, colorless solid. **mp**: 115 °C. **HPLC** (AD-H, hexane:2-propanol, 9:1, v/v, 1.0 mL/min, 254 nm): t_R = 4.7 min (major), 7.2 min (minor), *er* = 1.26/1.00, *ee* = 11.6%.

Prepared according to **GP 8**: *N*-Boc-Tryptamine (26.0 mg, 0.100 mmol, 1.00 equiv.), tetrabutyl-ammonium chloride (10 mol%, 10 µL: 1 M in CH_2Cl_2), **1a** (0.19 g, 0.20 mL, 1.0 mmol, 10 equiv.). Chromatographic purification: silica gel, petroleum ether:EtOAc:NEt_3, 98.5:1:0.5, v/v. Yield: 32.8 mg, 0.100 mmol, 100%, colorless solid. **mp**: 95 °C (lit[47] 115–118 °C, *ee* = 99%). **HPLC** (AD-H, hexane:2-propanol, 9:1, v/v, 1.0 mL/min, 254 nm): t_R = 4.7 min (major), 7.2 min (minor), *er* = 72.4/1.00, *ee* = 97.3%.

NMR-data from lit[47] are reported in DMSO-d6, 80 °C. In $CDCl_3$ at 20 °C **3a** exhibits two carbamate-rotamers (A/B = 0.6/0.4). In case the resonance signals show a splitting and an assignment is possible the respective rotamer is denoted by the subscript A/B.

TLC (petroleum ether:EtOAc: NEt_3, 98.5:1:0.5, v/v): R_f = 0.25. **^{1}H-NMR** (600 MHz, $CDCl_3$): δ 7.12–7.05 (m, 2 H), 6.75–6.68 (m, 1 H), 6.57 (d, *J* = 7.7 Hz, 1 H), 6.06–5.98 (m, 1 H), 5.27_A (s, 0.6 H), 5.16_B (s, 0.4 H), 5.12–5.01 (m, 2H), 4.87 (s_{br}, 1 H), 3.67_B (dd, *J* = 10.7 Hz, 8.2 Hz, 0.4 H), 3.53_A (dd, *J* = 10.7 Hz, 8.2 Hz, 0.6 H), 2.94–2.85 (m, 1 H), 2.32–2.23 (m, 1 H), 2.01–1.96 (m, 1 H), 1.52_B (s, 3.6 H), 1.43_A (s, 5.4 H), 1.10_B (s, 1.2 H), 1.08_A (s, 1.8 H), 1.00 (s, 3 H). **^{13}C-NMR** (76 MHz, $CDCl_3$): δ 154.52_A, 153.48_B, 150.70_A, 150.26_B, 144.59, 130.42_A, 130.35_B, 128.51_B, 128.41_A, 125.13_B, 125.03_A, 118.61_B, 118.06_A, 113.69, 108.99, 80.28_B, 79.86_A, 77.86_A, 77.77_B, 64.25_B, 63.03_A, 45.93_A, 45.50_B, 41.12, 32.15_A, 31.90_B, 28.83_B, 28.62_A, 23.16_{A+B} (two signals), 22.64_B, 22.55_A. **IR** (ATR): $\tilde{v}$ 3388, 2971, 2932, 1672, 1603, 1479,

1462, 1402, 1364, 1307, 1250, 1166, 1128, 1048, 1008, 925, 890, 782, 756, 741, 633, 517, 454. **HRMS** (ESI, m/z): $[M+H]^+$ calc. for $C_{20}H_{29}N_2O_2$, 329.222; found, 329.217.

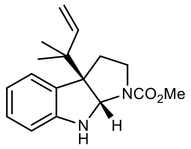

methyl (3a*R*,8a*S*)-3a-(2-methylbut-3-en-2-yl)-3,3a,8,8a-tetrahydropyrrolo[2,3-*b*]-indole-1(2*H*)-carboxylate
(–)-3b

<u>Prepared according to **GP 7**:</u> methyl (2-(1*H*-indol-3-yl)ethyl)carbamate (21.8 mg, 0.100 mmol, 1.00 equiv.), **precat. 1** (2.7 mg, 2.5 µmol, 2.5 mol%), (*R*)-**L1** (2.5 mg, 5.0 µmol, 5.0 mol%), MTBD (3 mg, 3 µL, 20 µmol, 20 mol%), BPh_3 (31 mg, 0.13 mmol, 1.3 equiv.), **1a** (93 mg, 97 µL, 0.50 mmol, 5.0 equiv.). Chromatographic purification: silica gel, step gradient: petroleum ether:CH_2Cl_2:NEt_3, 89.5:10:0.5 → 79.5:20:0.5, v/v. Yield: 28.4 mg, 0.0992 mmol, 99%, colorless solid. **mp**: 90 °C. **HPLC** (AD-H, hexane:2-propanol, 9:1, v/v, 1.0 mL/min, 254 nm): t_R = 7.2 min (minor), 7.6 min (major), *er* = 1.00/51.8, *ee* = 96.2%. $[\alpha]_D^{20}$: –348 (0.5, CH_2Cl_2).

<u>Prepared according to **GP 6**:</u> methyl (2-(1*H*-indol-3-yl)ethyl)carbamate (21.8 mg, 0.100 mmol, 1.00 equiv.), BEt_3 (0.13 mL, 1.3 equiv.), DBU (3 mg, 3 µL, 20 µmol, 20 mol%), **1a** (56 mg, 59 µL, 0.30 mmol, 3.0 equiv.). Chromatographic purification: silica gel, step gradient: petroleum ether:CH_2Cl_2:NEt_3, 89.5:10:0.5 → 79.5:20:0.5, v/v. Yield: 27.7 mg, 0.0967 mmol, 97%, colorless solid. **mp**: 83 °C (lit[48] 89–90.5 °C, from MeOH, racemic). **HPLC** (AD-H, hexane:2-propanol, 9:1, v/v, 1.0 mL/min, 254 nm): t_R = 7.2 min (minor), 7.6 min (major), *er* = 1.00/1.04, *ee* = 2.1%.

NMR-data from lit[48] are reported incompletely. In $CDCl_3$ at 20 °C **3b** exhibits two carbamate-rotamers (A/B = 0.6/0.4). In case the resonance signals show a splitting and an assignment is possible the respective rotamer is denoted by the subscript A/B.

TLC (petroleum ether:CH_2Cl_2:NEt_3, 79.5:20:0.5, v/v): R_f = 0.23. **^{1}H-NMR** (400 MHz, $CDCl_3$): δ 7.13–7.03 (m, 2 H), 6.76–6.68 (m, 1 H), 6.58–6.52 (m, 1 H), 6.06–5.93 (m, 1 H), 5.30_A (s, 0.6 H), 5.23_B (s, 0.4 H), 5.12–4.99 (m, 2.6 H), 4.58_B (s_{br}, 0.4 H), 3.76_B (s, 1.2 H), 3.73_B (dd, *J* = 10.8 Hz, 8.3 Hz, 0.4 H), 3.67_A (s, 1.8 H), 3.59_A (dd, *J* = 10.8 Hz, 8.3 Hz, 0.6 H), 2.99–2.87 (m, 1 H), 2.36–2.23 (m, 1 H), 2.06–1.97 (m, 1 H), 1.09 (s, 3 H), 1.01–0.97 (m, 3 H). **^{13}C-NMR** (100 MHz, $CDCl_3$): δ 155.47_A, 154.56_B, 150.48_A, 150.16_B, 144.46, 130.20_A, 130.13_B, 128.57_B, 128.52_A, 125.06_B, 125.00_A, 118.63_B, 118.31_A, 113.82_A, 113.78_B, 109.13_A, 109.05_B, 78.24_A, 77.64_B, 64.33_B, 63.08_A, 52.67_B, 52.33_A, 46.03_B, 45.70_A, 41.12_B, 41.08_A, 32.28_A, 32.05_B, 23.09_{A+B} (two signals), 22.62, 22.59. **IR** (ATR): $\tilde{\nu}$ 3366, 2969, 2877, 1686, 1606, 1482, 1448, 1380, 1309, 1290, 1253, 1201, 1161, 1111, 1091, 1071, 1053, 1023, 1006, 964, 943, 917, 886, 773, 742, 703, 634, 541, 512, 492, 468. **HRMS** (ESI, m/z): $[M+H]^+$ calc. for $C_{17}H_{23}N_2O_2$, 287.176; found, 287.175.

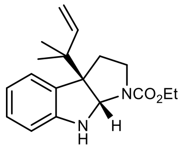

ethyl (3a*R*,8a*S*)-3a-(2-methylbut-3-en-2-yl)-3,3a,8,8a-tetrahydropyrrolo[2,3-*b*]-indole-1(2*H*)-carboxylate.
(–)-3c

<u>Prepared according to **GP 7**:</u> ethyl (2-(1*H*-indol-3-yl)ethyl)carbamate (23.2 mg, 0.100 mmol, 1.00 equiv.), **precat. 1** (2.7 mg, 2.5 µmol, 2.5 mol%), (*R*)-**L1** (2.5 mg, 5.0 µmol, 5.0 mol%), MTBD (3 mg, 3 µL, 20 µmol, 20 mol%), BPh_3 (31 mg, 0.13 mmol, 1.3 equiv.), **1a** (93 mg, 97 µL, 0.50 mmol, 5.0 equiv.). Chromatographic purification: silica gel, step gradient: CH_2Cl_2:Et_2O, 99:1 → 39:1, v/v. Yield: 28.9 mg, 0.0962 mmol, 96%, colorless oil. **HPLC** (AD-H, hexane:2-propanol, 9:1, v/v, 1.0 mL/min, 254 nm): t_R = 7.3 min (major), 7.6 min (minor), *er* = 77.0/1.00, *ee* = 97.4%. **$[\alpha]_D^{20}$**: –299 (1.0, CH_2Cl_2).

<u>Prepared according to **GP 6**:</u> ethyl (2-(1*H*-indol-3-yl)ethyl)carbamate (23.2 mg, 0.100 mmol, 1.00 equiv.), BEt_3 (0.13 mL, 1.3 equiv.), DBU (3 mg, 3 µL, 20 µmol, 20 mol%), **1a** (56 mg, 59 µL, 0.30 mmol, 3.0 equiv.). Chromatographic purification: silica gel, step gradient: CH_2Cl_2:Et_2O, 99:1 → 39:1, v/v. Yield: 7.9 mg, 0.026 mmol, 26%, colorless oil. **HPLC** (AD-H, hexane:2-propanol, 9:1, v/v, 1.0 mL/min, 254 nm): t_R = 7.3 min (major), 7.6 min (minor), *er* = 1.02/1.00, *ee* = 1.0%.

In $CDCl_3$ at 20 °C **3c** exhibits two carbamate-rotamers (A/B = 0.6/0.4). In case the resonance signals show a splitting and an assignment is possible the respective rotamer is denoted by the subscript A/B.

TLC (CH_2Cl_2:Et_2O, 39:1, v/v): R_f = 0.27. **^{1}H-NMR** (400 MHz, $CDCl_3$): δ 7.13–7.04 (m, 2 H), 6.76–6.69 (m, 1 H), 6.59–6.54 (m, 1 H), 6.05–5.97 (m, 1 H), 5.30_A (s, 0.6 H), 5.23_B (s, 0.4 H), 5.12–5.00 (m, 2.6 H), 4.58_B (s_{br}, 0.4 H), 4.26–4.04 (m, 2 H), 3.72_B (dd, *J* = 10.7 Hz, 8.1 Hz, 0.4 H), 3.60_A (dd, *J* = 10.7 Hz, 8.1 Hz, 0.6 H), 2.93 (ddd, *J* = 11.4 Hz, 10.5 Hz, 6.1 Hz, 1 H), 2.35–2.24 (m, 1 H), 2.05–1.99 (m, 1 H), 1.33_B (t, *J* = 7.1 Hz, 1.2 H), 1.23_A (t, *J* = 7.1 Hz, 1.8 H), 1.11–1.08 (m, 3 H), 1.00 (s, 3 H). **^{13}C-NMR** (150 MHz, $CDCl_3$): δ 155.16_A, 154.21_B, 150.54_A, 150.17, 144.48, 130.26_A, 130.17, 128.55_B, 128.50_A, 125.08_B, 125.01_A, 118.65_B, 118.26_A, 113.79_A, 113.76_B, 109.11_A, 109.06_B, 78.15_A, 77.60_B, 64.31_B, 63.07_A, 61.44_B, 61.13_A, 45.91_B, 45.67_A, 41.11_B, 41.08_A, 32.23_A, 32.01_B, 23.10, 22.62, 22.58, 15.02_B, 14.79_A. **IR** (ATR): $\tilde{v}$ 3359, 2972, 2877, 1685, 1606, 1482, 1466, 1417, 1380, 1348, 1319, 1254, 1201, 1110, 1091, 1054, 1023, 1009, 916, 893, 774, 743, 514, 469. **HRMS** (ESI, m/z): $[M+H]^+$ calc. for $C_{18}H_{25}N_2O_2$, 301.192; found, 301.193.

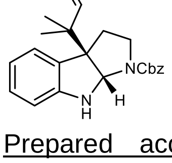

benzyl (3a*R*,8a*S*)-3a-(2-methylbut-3-en-2-yl)-3,3a,8,8a-tetrahydropyrrolo[2,3-*b*]-indole-1(2*H*)-carboxylate
(–)-3d

<u>Prepared according to **GP 7**:</u> benzyl (2-(1*H*-indol-3-yl)ethyl)carbamate (29.4 mg, 0.100 mmol, 1.00 equiv.), **precat. 1** (2.7 mg, 2.5 µmol, 2.5 mol%), (*R*)-**L1** (2.5 mg, 5.0 µmol, 5.0 mol%), MTBD (3 mg, 3 µL, 20 µmol, 20 mol%), BPh_3 (31 mg, 0.13 mmol, 1.3 equiv.), **1a** (93 mg, 97 µL, 0.50 mmol, 5.0 equiv.). Chromatographic purification: silica gel, step gradient: petroleum ether:EtOAc:NEt_3, 97.5:2:0.5 → 95.5:4:0.5, v/v. Additional chromatographic purification: silica gel, petroleum ether:EtOAc, 9:1, v/v. Yield: 36.0 mg, 0.0993 mmol, 99%, colorless solid. **mp**: 79 °C. **HPLC** (AD-H,

hexane:2-propanol, 9:1, v/v, 1.0 mL/min, 254 nm): t_R = 10.5 min (minor), 11.3 min (major), *er* = 1.00/55.4, *ee* = 96.5%. **$[\alpha]_D^{20}$**: –328 (1.0, CH_2Cl_2).

Prepared according to **GP 6**: benzyl (2-(1*H*-indol-3-yl)ethyl)carbamate (29.4 mg, 0.100 mmol, 1.00 equiv.), BEt_3 (0.13 mL, 1.3 equiv.), DBU (3 mg, 3 µL, 20 µmol, 20 mol%), **1a** (56 mg, 59 µL, 0.30 mmol, 3.0 equiv.). Chromatographic purification: silica gel, step gradient: petroleum ether:EtOAc:NEt_3, 97.5:2:0.5 → 95.5:4:0.5, v/v. Additional chromatographic purification: silica gel, petroleum ether:EtOAc, 9:1, v/v. Yield: 24.4 mg, 0.0673 mmol, 67%, colorless solid. **mp**: 93 °C. **HPLC** (AD-H, hexane:2-propanol, 9:1, v/v, 1.0 mL/min, 254 nm): t_R = 10.5 min (minor), 11.3 min (major), *er* = 1.00/1.02, *ee* = 1.0%.

In $CDCl_3$ at 20 °C **3d** exhibits two carbamate-rotamers (A/B = 0.6/0.4). In case the resonance signals show a splitting and an assignment is possible the respective rotamer is denoted by the subscript A/B.

TLC (petroleum ether:EtOAc:NEt_3, 97.5:2:0.5, v/v): R_f = 0.20; (petroleum ether:EtOAc, 9:1, v/v): R_f = 0.33. **^{1}H-NMR** (400 MHz, $CDCl_3$): δ 7.44–7.27 (m, 5 H), 7.13–7.02 (m, 2 H), 6.77–6.68 (m, 1 H), 6.58$_A$ (d, *J* = 8.0 Hz, 0.6 H), 6.50$_B$ (d, *J* = 8.0 Hz, 0.4 H), 6.07–5.94 (m, 1 H), 5.33$_A$ (s, 0.6 H), 5.30–5.23 (m, 0.8 H), 5.19–5.00 (m, 4.2 H), 4.52$_B$ (s_{br}, 0.4 H), 3.74$_B$ (dd, *J* = 10.7 Hz, 8.1 Hz, 0.4 H), 3.66$_A$ (dd, *J* = 10.7 Hz, 8.1 Hz, 0.6 H), 3.03–2.91 (m, 1 H), 2.37–2.23 (m, 1 H), 2.03 (dd, *J* = 12.4 Hz, 6.2 Hz, 1 H), 1.12–1.07 (m, 3 H), 1.01 (s, 1.8 H), 0.99 (s, 1.2 H). **^{13}C-NMR** (100 MHz, $CDCl_3$): δ 154.86$_A$, 153.94$_B$, 150.47$_A$, 150.08$_B$, 144.46, 136.78$_B$, 136.66$_A$, 130.20$_A$, 130.08$_B$, 128.83, 128.61, 128.58, 128.55, 128.37, 128.20, 128.15, 128.02, 125.06$_B$, 125.02, 118.66$_B$, 118.34$_A$, 113.86$_A$, 113.82$_B$, 109.16$_A$, 109.02$_B$, 78.32$_A$, 77.67$_B$, 67.16$_B$, 66.89$_A$, 64.35$_B$, 63.12$_A$, 46.10$_B$, 45.79$_A$, 41.12$_B$, 41.10$_A$, 32.24$_A$, 32.02$_B$, 23.11, 23.08, 22.64, 22.60. **IR** (ATR): $\tilde{\nu}$ 3368, 2969, 2877, 1687, 1606, 1497, 1483, 1466, 1412, 1383, 1351, 1308, 1290, 1253, 1199, 1161, 1104, 1071, 1052, 1004, 942, 913, 887, 771, 740, 696, 642, 586, 517, 467. **HRMS** (ESI, m/z): $[M+H]^+$ calc. for $C_{23}H_{27}N_2O_2$, 363.207; found, 363.207.

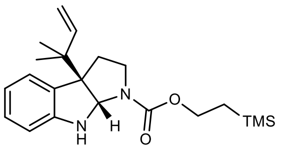

2-(trimethylsilyl)ethyl (3a*R*,8a*S*)-3a-(2-methylbut-3-en-2-yl)-3,3a,8,8a-tetrahydropyrrolo[2,3-*b*]indole-1(2*H*)-carboxylate.

(–)-3e

Prepared according to **GP 7**: 2-(trimethylsilyl)ethyl (2-(1*H*-indol-3-yl)ethyl)carbamate (30.4 mg, 0.100 mmol, 1.00 equiv.), **precat. 1** (2.7 mg, 2.5 µmol, 2.5 mol%), (*R*)-**L1** (2.5 mg, 5.0 µmol, 5.0 mol%), MTBD (3 mg, 3 µL, 20 µmol, 20 mol%), BPh_3 (31 mg, 0.13 mmol, 1.3 equiv.), **1a** (93 mg, 97 µL, 0.50 mmol, 5.0 equiv.). Chromatographic purification: silica gel, petroleum ether:EtOAc:NEt_3, 98.5:1:0.5, v/v. Yield: 36.9 mg, 0.0990 mmol, 99%, colorless oil. **HPLC** (AD-H, hexane:2-propanol, 9:1, v/v, 0.25 mL/min, 254 nm): t_R = 20.7 min (minor), 21.8 min (major), *er* = 1.00/69.3, *ee* = 97.2%. **$[\alpha]_D^{20}$**: –361 (1.0, CH_2Cl_2).

Prepared according to **GP 6**: 2-(trimethylsilyl)ethyl (2-(1*H*-indol-3-yl)ethyl)carbamate (30.4 mg, 0.100 mmol, 1.00 equiv.), BEt_3 (0.13 mL, 1.3 equiv.), DBU (3 mg, 3 µL, 20 µmol, 20 mol%), **1a** (56 mg, 59 µL, 0.30 mmol, 3.0 equiv.). Chromatographic purification: silica gel, petroleum ether:EtOAc:NEt_3,

98.5:1:0.5, v/v. Yield: 37.2 mg, 0.0998 mmol, 100%, colorless solid. **mp**: 90 °C. **HPLC** (AD-H, hexane:2-propanol, 9:1, v/v, 0.25 mL/min, 254 nm): t_R = 20.7 min (minor), 21.8 min (major), *er* = 1.00/1.09, *ee* = 4.4%.

In $CDCl_3$ at 20 °C **3e** exhibits two carbamate-rotamers (A/B = 0.6/0.4). In case the resonance signals show a splitting and an assignment is possible the respective rotamer is denoted by the subscript A/B.

TLC (petroleum ether:EtOAc:NEt_3, 98.5:1:0.5, v/v): R_f = 0.25. **^{1}H-NMR** (500 MHz, $CDCl_3$): δ 7.12–7.09 (m, 1 H), 7.07 (td, *J* = 7.6 Hz, 1.2 Hz, 1 H), 6.75–6.69 (m, 1 H), 6.58–6.54 (m, 1 H), 6.05–5.96 (m, 1 H), 5.30$_A$ (s, 0.6 H), 5.23$_B$ (s, 0.4 H), 5.11–5.01 (m, 2 H), 4.28–4.08 (m, 2 H), 3.71$_B$ (dd, *J* = 10.7 Hz, 8.1 Hz, 0.4 H), 3.59$_A$ (dd, *J* = 10.4 Hz, 8.1 Hz, 0.6 H), 2.96–2.88 (m, 1 H), 2.29 (qd, *J* = 12.0 Hz, 8.1 Hz, 1 H), 2.04–1.98 (m, 1 H), 1.12–0.94 (m, 8 H), 0.07$_B$ (s, 3.6 H), 0.02$_A$ (s, 5.4 H). **^{13}C-NMR** (125 MHz, $CDCl_3$): δ 155.31$_A$, 154.36$_B$, 150.57$_A$, 150.23$_B$, 144.51, 130.28$_A$, 130.22$_B$, 128.54$_B$, 128.50$_A$, 125.08$_B$, 125.01$_A$, 118.62$_B$, 118.24$_A$, 113.77$_A$, 113.75$_B$, 109.12$_A$, 109.04$_B$, 78.17$_A$, 77.60$_B$, 64.31$_B$, 63.66$_B$, 63.37$_A$, 63.06$_A$, 45.88$_B$, 45.68$_A$, 41.12$_B$, 41.10$_A$, 32.29$_A$, 32.05$_B$, 23.12, 23.09, 22.66, 22.60, 18.22$_B$, 17.90$_A$, –1.30$_B$, –1.34$_A$. **IR** (ATR): $\tilde{\nu}$ 3357, 2953, 2878, 1682, 1606, 1482, 1466, 1413, 1350, 1319, 1249, 1200, 1105, 1054, 935, 916, 887, 855, 835, 772, 741, 693, 515, 468. **HRMS** (ESI, m/z): $[M+H]^+$ calc. for $C_{21}H_{33}N_2O_2Si$, 373.231; found, 373.229.

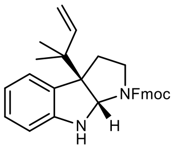

(9*H*-fluoren-9-yl)methyl (3a*R*,8a*S*)-3a-(2-methylbut-3-en-2-yl)-3,3a,8,8a-tetra-hydropyrrolo[2,3-*b*]indole-1(2*H*)-carboxylate
(–)-3f

Prepared according to **GP 7**: Deviantly with reaction time: 1 hour. (9*H*-fluoren-9-yl)methyl (2-(1*H*-indol-3-yl)ethyl)carbamate (38.2 mg, 0.100 mmol, 1.00 equiv.), **precat. 1** (2.7 mg, 2.5 µmol, 2.5 mol%), (*R*)-**L1** (2.5 mg, 5.0 µmol, 5.0 mol%), MTBD (3 mg, 3 µL, 20 µmol, 20 mol%), BPh_3 (31 mg, 0.13 mmol, 1.3 equiv.), **1a** (93 mg, 97 µL, 0.50 mmol, 5.0 equiv.). Chromatographic purification: silica gel, petroleum ether:EtOAc, 9:1, v/v. Yield: 30.0 mg, 0.0666 mmol, 67%, yellowish oil. **HPLC** (AD-H, hexane:2-propanol, 1:1, v/v, 1 mL/min, 254 nm): t_R = 8.0 min (major), 10.0 min (minor), *er* = 60.5/1.00, *ee* = 96.7%. **$[\alpha]_D^{20}$**: –269 (1.38, CH_2Cl_2).

Prepared according to **GP 6**: Deviantly with reaction time: 1 hour. (9*H*-fluoren-9-yl)methyl (2-(1*H*-indol-3-yl)ethyl)carbamate (38.2 mg, 0.100 mmol, 1.00 equiv.), BEt_3 (0.13 mL, 1.3 equiv.), DBU (3 mg, 3 µL, 20 µmol, 20 mol%), **1a** (56 mg, 59 µL, 0.30 mmol, 3.0 equiv.). Chromatographic purification: silica gel, petroleum ether:EtOAc, 9:1, v/v. Yield: 32.0 mg, 0.0710 mmol, 71%, yellowish oil. **HPLC** (AD-H, hexane:2-propanol, 1:1, v/v, 1 mL/min, 254 nm): t_R = 8.0 min (minor), 10.0 min (major), *er* = 1.00/1.18, *ee* = 8.4%. The major enantiomer is **(+)-3f**.

In $CDCl_3$ at 20 °C **3f** exhibits two carbamate-rotamers (A/B = 0.55/0.45). In case the resonance signals show a splitting and an assignment is possible the respective rotamer is denoted by the subscript A/B.

TLC (petroleum ether:EtOAc, 9:1, v/v): R_f = 0.25. **^{1}H-NMR** (500 MHz, $CDCl_3$): δ 7.84–7.25 (m, 8 H), 7.14$_A$ (d, *J* = 7.5 Hz, 0.55 H), 7.10$_A$ (td, *J* = 7.5 Hz, 1.2 Hz, 0.55 H), 7.05–7.01 (m, 0.9 H), 6.75$_A$ (td,

J = 7.5 Hz, 1.1 Hz, 0.55 H), 6.67$_B$ (td, J = 7.5 Hz, 1.1 Hz, 0.45 H), 6.59$_A$ (d, J = 8.1 Hz, 0.55 H), 6.33$_B$ (dd, J = 8.1 Hz, 1.0 Hz, 0.45 H), 6.04$_A$ (dd, J = 17.4 Hz, 10.8 Hz, 0.55 H), 5.86$_B$ (dd, J = 17.4 Hz, 10.8 Hz, 0.45 H), 5.33$_A$ (s, 0.55 H), 5.12$_A$ (dd, J = 10.8 Hz, 1.3 Hz, 0.55 H), 5.10–5.01 (m, 1.55 H), 4.98$_B$ (dd, J = 17.4 Hz, 1.3 Hz, 0.45 H), 4.80$_B$ (s, 0.45 H), 4.61$_B$ (dd, J = 5.1 Hz, 1.2 Hz, 0.9 H), 4.37$_A$ (dd, J = 7.2 Hz, 4.5 Hz, 1.1 H), 4.31$_B$ (t, J = 5.1 Hz, 0.45 H), 4.21$_A$ (t, J = 7.2 Hz, 0.55 H), 3.82$_B$ (s_{br}, 0.45 H), 3.68$_A$ (dd, J = 10.3 Hz, 8.2 Hz, 0.55 H), 3.61$_B$ (dd, J = 10.6 Hz, 8.2 Hz, 0.45 H), 3.03$_A$ (ddd, J = 11.5 Hz, 10.3 Hz, 6.0 Hz, 0.55 H), 2.84$_B$ (td, J = 11.1 Hz, 6.0 Hz, 0.45 H), 2.36$_A$ (ddd, J = 12.5 Hz, 11.5 Hz, 8.2 Hz, 0.55 H), 2.21–2.12 (m, 0.9 H), 2.08$_A$ (dd, J = 12.5 Hz, 6.0 Hz, 0.55 H), 1.93$_B$ (dd, J = 12.5 Hz, 6.0 Hz, 0.45 H), 1.12 (s, 1.65 H), 1.03–1.00 (m, 3 H), 0.86 (s, 1.35 H). **^{13}C-NMR** (125 MHz, $CDCl_3$): δ 154.83$_A$, 153.74$_B$, 150.44$_A$, 150.03$_B$, 144.43$_A$, 144.39$_B$, 144.21, 144.19, 144.08, 143.98, 141.72$_B$, 141.56$_A$, 141.42 (two signals), 130.13$_A$, 129.75$_B$ (C-9), 128.58$_A$ (C-6), 128.46$_B$ (C-6), 127.94, 127.86, 127.84, 127.82, 127.36, 127.34, 127.15, 127.13, 125.19, 125.18, 125.00, 124.93, 124.87, 124.72, 120.29, 120.16, 120.09 (two signals), 118.35$_A$, 118.12$_B$, 113.91$_A$, 113.72$_B$, 109.22$_A$, 108.48$_B$, 78.30$_A$, 77.49$_B$, 67.22$_A$, 66.50$_B$, 63.83$_B$, 63.11$_A$, 47.49$_B$, 47.37$_A$, 45.82$_A$, 45.62$_B$, 41.11$_A$, 41.03$_B$, 32.34$_A$, 32.01$_B$, 23.11, 22.81, 22.61, 22.51. **IR** (ATR): $\tilde{\nu}$ 3411, 3041, 2969, 2876, 1687, 1606, 1479, 1466, 1450, 1415, 1382, 1349, 1319, 1289, 1247, 1200, 1155, 1109, 1054, 1023, 1006, 910, 891, 756, 735, 621, 544. **HRMS** (ESI, m/z): $[M+H]^+$ calc. for $C_{30}H_{31}N_2O_2$, 451.239; found, 451.238.

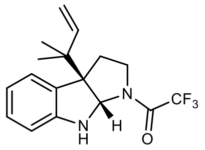

2,2,2-trifluoro-1-((3a*R*,8a*S*)-3a-(2-methylbut-3-en-2-yl)-3,3a,8,8a-tetrahydro-pyrrolo[2,3-*b*]indol-1(2*H*)-yl)ethan-1-one
(–)-3g

Prepared according to **GP 7**: *N*-(2-(1*H*-indol-3-yl)ethyl)-2,2,2-trifluoroacetamide (25.6 mg, 0.100 mmol, 1.00 equiv.), **precat. 1** (2.7 mg, 2.5 µmol, 2.5 mol%), (*R*)-**L1** (2.5 mg, 5.0 µmol, 5.0 mol%), MTBD (3 mg, 3 µL, 20 µmol, 20 mol%), BPh_3 (31 mg, 0.13 mmol, 1.3 equiv.), **1a** (93 mg, 97 µL, 0.50 mmol, 5.0 equiv.). Chromatographic purification: silica gel, step gradient: petroleum ether:EtOAc:NEt_3, 99.25:0.25:0.5 → 98.5:1:0.5, v/v. Yield: 31.6 mg, 0.0974 mmol, 97%, colorless oil. **HPLC** (AD-H, hexane:2-propanol, 9:1, v/v, 1 mL/min, 254 nm): t_R = 4.5 min (minor), 5.5 min (major), *er* = 1.00/53.1, *ee* = 96.3%. **$[\alpha]_D^{20}$**: –424 (1.0, CH_2Cl_2).

Prepared according to **GP 6**: *N*-(2-(1*H*-indol-3-yl)ethyl)-2,2,2-trifluoroacetamide (25.6 mg, 0.100 mmol, 1.00 equiv.), BEt_3 (0.13 mL, 1.3 equiv.), DBU (3 mg, 3 µL, 20 µmol, 20 mol%), **1a** (56 mg, 59 µL, 0.30 mmol, 3.0 equiv.). Chromatographic purification: silica gel, step gradient: petroleum ether:EtOAc:NEt_3, 99.25:0.25:0.5 → 98.5:1:0.5, v/v. Yield: 32.3 mg, 0.0996 mmol, 100%, colorless solid. **mp**: 88 °C. **HPLC** (AD-H, hexane:2-propanol, 9:1, v/v, 1 mL/min, 254 nm): t_R = 4.5 min (major), 5.5 min (minor), *er* = 1.06/1.00, *ee* = 2.9%. The major enantiomer is **(+)-3g**.

In $CDCl_3$ at 20 °C **3g** exhibits two amide-rotamers (A/B = 0.9/0.1). In case the resonance signals show a splitting and an assignment is possible the respective rotamer is denoted by the subscript A/B.

TLC (petroleum ether:EtOAc:NEt$_3$, 99.25:0.25:0.5, v/v): $R_f = 0.25$. **^{1}H-NMR** (600 MHz, CDCl$_3$): δ 7.17–7.09 (m, 2 H), 6.79–6.74 (m, 1 H), 6.60–6.56 (m, 1 H), 5.99$_A$ (dd, J = 17.4 Hz, 10.8 Hz, 0.9 H), 5.94$_B$ (dd, J = 17.4 Hz, 10.8 Hz, 0.1 H), 5.60$_B$ (s, 0.1 H), 5.51$_A$ (s, 0.9 H), 5.16–5.03 (m, 2.9 H), 4.51$_B$ (s$_{br}$, 0.1 H), 4.15$_B$ (dd, J = 12.2 Hz, 7.7 Hz, 0.1 H), 3.89–3.83$_A$ (m, 0.9 H), 3.17$_A$ (ddd, J = 12.1 Hz, 11.0 Hz, 6.1 Hz, 0.9 H), 3.00$_B$ (td, J = 12.2 Hz, 5.9 Hz, 0.1 H), 2.40$_A$ (td, J = 12.4 Hz, 8.0 Hz, 0.9 H), 2.24$_B$ (td, J = 12.4 Hz, 7.7 Hz, 0.1 H), 2.17$_A$ (dd, J = 12.4 Hz, 6.1 Hz, 0.9 H), 2.05$_B$ (dd, J = 12.4 Hz, 5.9 Hz, 0.1 H), 1.13$_B$ (s, 0.3 H), 1.12$_A$ (s, 2.7 H), 1.01$_A$ (s, 2.7 H), 1.00$_B$ (s, 0.3 H). **^{13}C-NMR**[g] (150 MHz, CDCl$_3$): δ 156.04$_A$ (q, J = 37 Hz), 149.81$_A$, 149.36$_B$, 143.79$_A$, 143.55$_B$, 129.48$_B$, 128.99$_B$, 128.97$_{A+B}$ (two signals), 125.01$_B$, 124.91$_A$, 119.18$_B$, 118.85$_A$, 116.15$_A$ (q, J = 288 Hz), 114.62$_B$, 114.51$_A$, 109.13$_A$, 108.67$_B$, 79.52$_A$, 77.90$_B$, 65.78$_B$, 61.82$_A$, 46.62$_A$ (q, J = 6.6 Hz), 46.31$_B$, 41.43$_B$, 40.97$_A$, 32.68$_A$, 32.53$_B$, 22.94$_A$, 22.86$_B$, 22.59$_{A+B}$ (two signals). **^{19}F-NMR** (565 MHz, CDCl$_3$): δ –69.87$_B$, –72.61$_A$. **IR** (ATR): $\tilde{\nu}$ 3384, 2974, 2894, 1681, 1638, 1608, 1483, 1467, 1415, 1384, 1366, 1347, 1314, 1257, 1202, 1137, 1093, 1071, 1010, 920, 881, 747, 695, 643, 539, 524, 467. **HRMS** (ESI, m/z): $[M+H]^+$ calc. for $C_{17}H_{20}F_3N_2O$, 325.153; found, 325.154

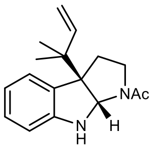

1-((3a*R*,8a*S*)-3a-(2-methylbut-3-en-2-yl)-3,3a,8,8a-tetrahydropyrrolo[2,3-*b*]indol-1(2*H*)-yl)ethan-1-one
(–)-3h

Prepared according to **GP 7**: *N*-(2-(1*H*-indol-3-yl)ethyl)acetamide (20.2 mg, 0.100 mmol, 1.00 equiv.), **precat. 1** (2.7 mg, 2.5 µmol, 2.5 mol%), (*R*)-**L1** (2.5 mg, 5.0 µmol, 5.0 mol%), MTBD (3 mg, 3 µL, 20 µmol, 20 mol%), BPh$_3$ (31 mg, 0.13 mmol, 1.3 equiv.), **1a** (93 mg, 97 µL, 0.50 mmol, 5.0 equiv.). Chromatographic purification: silica gel, step gradient: petroleum ether:EtOAc:NEt$_3$, 66.5:33:0.5 → 49.5:50:0.5, v/v. Yield: 26.8 mg, 0.0991 mmol, 99%, colorless solid. **mp**: 75 °C. **HPLC** (AD-H, hexane (0.1% NHEt$_2$):2-propanol, 9:1, v/v, 1 mL/min, 254 nm): t_R = 8.2 min (minor), 11.3 min (major), *er* = 1.00/47.5, *ee* = 95.9%. **$[\alpha]_D^{24}$**: –386 (1.0, CHCl$_3$).

Prepared according to **GP 6**: *N*-(2-(1*H*-indol-3-yl)ethyl)acetamide (20.2 mg, 0.100 mmol, 1.00 equiv.), BEt$_3$ (0.13 mL, 1.3 equiv.), DBU (3 mg, 3 µL, 20 µmol, 20 mol%), **1a** (56 mg, 59 µL, 0.30 mmol, 3.0 equiv.). Chromatographic purification: silica gel, step gradient: petroleum ether:EtOAc:NEt$_3$, 66.5:33:0.5 → 49.5:50:0.5, v/v. Yield: 21.4 mg, 0.0791 mmol, 79%, colorless oil. **HPLC** (AD-H, hexane (0.1% NHEt$_2$):2-propanol, 9:1, v/v, 1 mL/min, 254 nm): t_R = 8.2 min (minor), 11.3 min (major), *er* = 1.00/1.06, *ee* = 3.1%.

In CDCl$_3$ at 20 °C **3h** exhibits two amide-rotamers (A/B = 0.90/0.10). In case the resonance signals show a splitting and an assignment is possible the respective rotamer is denoted by the subscript A/B.

TLC (petroleum ether:EtOAc:NEt$_3$, 99.25:0.25:0.5, v/v): $R_f = 0.25$. **^{1}H-NMR** (600 MHz, CDCl$_3$): δ 7.14$_B$ (dd, J = 7.4 Hz, 1.2 Hz, 0.1 H), 7.12–7.05 (m, 1.9 H), 6.78$_B$ (td, J = 7.5 Hz, 1.1 Hz, 0.1 H), 6.71$_A$ (td,

[g] For the carbon atoms C(O) and CF_3 only the major resonances are reported.

J = 7.5 Hz, 1.1 Hz, 0.9 H), 6.59$_B$ (d, J = 7.8 Hz, 0.1 H), 6.55$_A$ (d, J = 7.8 Hz, 0.9 H), 6.01$_A$ (dd, J = 17.4 Hz, 10.8 Hz, 0.9 H), 5.97$_B$ (dd, J = 17.4 Hz, 10.8 Hz, 0.1 H), 5.41$_A$ (s, 0.9 H), 5.29 (s, 0.1 H), 5.16 (s$_{br}$, 1 H), 5.12–5.02 (m, 2 H), 4.05$_B$ (dd, J = 11.6 Hz, 7.9 Hz, 0.1 H), 3.55$_A$ (ddd, J = 10.0 Hz, 8.3 Hz, 0.8 Hz, 0.9 H), 3.09$_A$ (ddd, J = 11.6 Hz, 10.0 Hz, 6.3 Hz, 0.9 H), 2.83$_B$ (td, J = 11.6 Hz, 6.0 Hz, 0.1 H), 2.36$_A$ (ddd, J = 12.5 Hz, 11.6 Hz, 8.3 Hz, 0.9 H), 2.21$_B$ (td, J = 12.1 Hz, 8.0 Hz, 0.1 H), 2.16$_B$ (s, 0.3 H), 2.09$_A$ (dd, J = 12.5 Hz, 6.3 Hz, 0.9 H), 2.02–1.96 (m, 2.8 H), 1.12$_B$ (s, 0.3 H), 1.09$_A$ (s, 2.7 H), 0.99 (s, 3 H). **^{13}C-NMR** (150 MHz, $CDCl_3$): δ 170.03$_A$, 168.55$_B$, 150.60$_A$, 149.86$_B$, 144.35$_A$, 144.25$_B$, 130.60$_B$, 129.82$_A$, 128.73$_B$, 128.61$_A$, 125.12$_B$, 124.95$_A$, 119.47$_B$, 118.21$_A$, 114.04$_B$, 113.97$_A$, 109.61$_B$, 109.10$_A$, 78.48$_B$, 77.79$_A$, 65.68$_B$, 62.42$_A$, 47.37$_A$, 44.79$_B$, 41.37$_B$, 41.06$_A$, 32.33$_B$, 32.25$_A$, 23.05$_A$, 22.94$_B$, 22.78$_A$, 22.69$_B$, 22.52$_A$, 22.14$_B$. **IR** (ATR): $\tilde{\nu}$ 3329, 2970, 2874, 1635, 1606, 1483, 1466, 1417, 1382, 1364, 1316, 1257, 1205, 1153, 1092, 1061, 1009, 917, 886, 744. **HRMS** (ESI, m/z): $[M+H]^+$ calc. for $C_{17}H_{23}N_2O$, 271.1810; found, 271.1798.

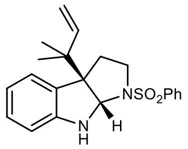

(3a*R*,8a*S*)-3a-(2-methylbut-3-en-2-yl)-1-(phenylsulfonyl)-1,2,3,3a,8,8a-hexahydropyrrolo[2,3-*b*]indole.

(–)-3i

<u>Prepared according to **GP 7**:</u> *N*-(2-(1*H*-indol-3-yl)ethyl)benzenesulfonamide (30.0 mg, 0.100 mmol, 1.00 equiv.), **precat. 1** (2.7 mg, 2.5 µmol, 2.5 mol%), (*R*)-**L1** (2.5 mg, 5.0 µmol, 5.0 mol%), MTBD (3 mg, 3 µL, 20 µmol, 20 mol%), BPh_3 (31 mg, 0.13 mmol, 1.3 equiv.), **1a** (93 mg, 97 µL, 0.50 mmol, 5.0 equiv.). Chromatographic purification: silica gel, petroleum ether:CH_2Cl_2:NEt_3, 80:19.5:0.5, v/v. Yield: 36.6 mg, 0.0993 mmol, 99%, colorless solid. **mp**: 153 °C. **HPLC** (AD-H, hexane:2-propanol, 1:1, v/v, 1 mL/min, 254 nm): t_R = 6.2 min (minor), 7.7 min (major), *er* = 1.00/22.1, *ee* = 91.3%. **$[\alpha]_D^{20}$**: –218 (1.0, CH_2Cl_2).

<u>Prepared according to **GP 6**:</u> *N*-(2-(1*H*-indol-3-yl)ethyl)benzenesulfonamide (30.0 mg, 0.100 mmol, 1.00 equiv.), BEt_3 (0.13 mL, 1.3 equiv.), DBU (3 mg, 3 µL, 20 µmol, 20 mol%), **1a** (56 mg, 59 µL, 0.30 mmol, 3.0 equiv.). Chromatographic purification: silica gel, petroleum ether:CH_2Cl_2:NEt_3, 80:19.5:0.5, v/v. Yield: 36.2 mg, 0.0985 mmol, 99%, colorless solid. **mp**: 141 °C. **HPLC** (AD-H, hexane:2-propanol, 1:1, v/v, 1 mL/min, 254 nm): t_R = 6.2 min (minor), 7.7 min (major), *er* = 1.00/1.22, *ee* = 9.9%.

TLC (petroleum ether:CH_2Cl_2:NEt_3, 80:19.5:0.5, v/v): R_f = 0.23. **^{1}H-NMR** (400 MHz, $CDCl_3$): δ 7.88–7.82 (m, 2 H), 7.63–7.56 (m, 1 H), 7.55–7.49 (m, 2 H), 7.08 (td, J = 7.6 Hz, 1.3 Hz, 1 H), 7.04 (d, J = 7.6 Hz, 1 H), 6.72 (td, J = 7.6 Hz, 1.1 Hz, 1 H), 6.58 (d, J = 7.6 Hz, 1 H), 5.67 (dd, J = 17.4 Hz, 10.8 Hz, 1 H), 5.17 (s, 1 H), 4.96–4.84 (m, 2 H), 4.72 (s$_{br}$, 1 H), 3.44 (ddd, J = 10.6 Hz, 7.8 Hz, 1.9 Hz, 1 H), 3.06 (td, J = 10.6 Hz, 6.0 Hz, 1 H), 2.09–1.89 (m, 2 H), 0.97 (s, 3 H), 0.83 (s, 3 H). **^{13}C-NMR** (100 MHz, $CDCl_3$): δ 150.03, 143.89, 139.41, 132.80, 129.73, 129.25, 128.73, 127.15, 124.84, 118.80, 113.95, 109.43, 80.75, 64.74, 47.96, 41.10, 33.29, 22.71, 22.64. **IR** (ATR): $\tilde{\nu}$ 3394, 2971, 2893, 1607, 1483, 1466, 1446, 1413, 1382, 1333, 1253, 1216, 1156, 1117, 1090, 1042, 997, 910, 882, 855, 837, 817, 790, 733, 714,

689, 648, 607, 581, 554, 510, 476. **HRMS** (ESI, m/z): $[M+H]^+$ calc. for $C_{21}H_{25}N_2O_2S$, 369.164; found, 369.164.

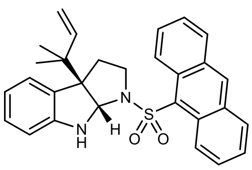

(3a*R*,8a*S*)-1-(anthracen-9-ylsulfonyl)-3a-(2-methylbut-3-en-2-yl)-1,2,3,3a,8,8ahexahydropyrrolo[2,3-*b*]indole
(–)-3j

Prepared according to **GP 7**: *N*-(2-(1*H*-indol-3-yl)ethyl)anthracene-9-sulfonamide (40.0 mg, 0.100 mmol, 1.00 equiv.), **precat. 1** (2.7 mg, 2.5 µmol, 2.5 mol%), (*R*)-**L1** (2.5 mg, 5.0 µmol, 5.0 mol%), MTBD (3 mg, 3 µL, 20 µmol, 20 mol%), BPh_3 (31 mg, 0.13 mmol, 1.3 equiv.), **1a** (93 mg, 97 µL, 0.50 mmol, 5.0 equiv.). Chromatographic purification: silica gel, petroleum ether:CH_2Cl_2:NEt_3, 80:19.5:0.5, v/v. Yield: 36.0 mg, 0.0768 mmol, 77%, yellow oil. **HPLC** (AD-H, hexane:2-propanol, 1:1, v/v, 1 mL/min, 254 nm): t_R = 8.3 min (minor), 15.6 min (major), *er* = 1.00/30.2, *ee* = 93.6%. $[\alpha]_D^{20}$: –13.9 (0.50, CH_2Cl_2).

Prepared according to **GP 6**: *N*-(2-(1*H*-indol-3-yl)ethyl)anthracene-9-sulfonamide (40.0 mg, 0.100 mmol, 1.00 equiv.), BEt_3 (0.13 mL, 1.3 equiv.), DBU (3 mg, 3 µL, 20 µmol, 20 mol%), **1a** (56 mg, 59 µL, 0.30 mmol, 3.0 equiv.). Chromatographic purification: silica gel, petroleum ether:CH_2Cl_2:NEt_3, 80:19.5:0.5, v/v. Yield: 32.9 mg, 0.0702 mmol, 70%, yellow solid. **mp**: 89 °C. **HPLC** (AD-H, hexane:2-propanol, 1:1, v/v, 1 mL/min, 254 nm): t_R = 8.3 min (minor), 15.6 min (major), *er* = 1.00/1.30, *ee* = 13.0%.

TLC (petroleum ether:CH_2Cl_2:NEt_3, 80:19.5:0.5, v/v): R_f = 0.25. **^{1}H-NMR** (400 MHz, $CDCl_3$): δ 9.32 (dd, *J* = 9.4 Hz, 1.0 Hz, 2 H), 8.72 (s, 1 H), 8.05 (dd, *J* = 8.5 Hz, 1.5 Hz, 2 H), 7.65 (ddd, *J* = 9.4 Hz, 6.5 Hz, 1.5 Hz, 2 H), 7.54 (ddd, *J* = 8.5 Hz, 6.5 Hz, 1.0 Hz, 2 H), 7.06 (td, *J* = 7.6 Hz, 1.3 Hz, 1 H), 7.03 (dd, *J* = 7.6 Hz, 1.3 Hz, 1 H), 6.68 (td, *J* = 7.6 Hz, 1.1 Hz, 1 H), 6.54 (d, *J* = 7.6 Hz, 1 H), 5.69 (dd, *J* = 17.4 Hz, 10.8 Hz, 1 H), 5.46 (s, 1 H), 4.86 (dd, *J* = 17.4 Hz, 1.3 Hz, 1 H), 4.84 (dd, *J* = 10.8 Hz, 1.3 Hz, 1 H), 3.36 (ddd, *J* = 9.2 Hz, 7.4 Hz, 1.4 Hz, 1 H), 3.06 (ddd, *J* = 11.1 Hz, 9.8 Hz, 5.5 Hz, 1 H), 2.11–1.99 (m, 1 H), 1.86 (ddd, *J* = 12.1 Hz, 5.5 Hz, 1.4 Hz, 1 H), 0.96 (s, 3 H), 0.83 (s, 3 H). **^{13}C-NMR** (100 MHz, $CDCl_3$): δ 150.24, 143.93, 136.10, 131.43, 131.15, 129.74, 129.48, 128.75, 128.67, 128.52, 125.51, 125.49, 124.88, 118.49, 113.72, 108.78, 80.04, 64.98, 47.12, 41.17, 33.88, 22.84, 22.65. **IR** (ATR): $\tilde{v}$ 3405, 2967, 2927, 2874, 1262, 1607, 1519, 1484, 1467, 1446, 1413, 1366, 1316, 1255, 1230, 1158, 1145, 1081, 1040, 1024, 971, 910, 848, 826, 779, 738, 672, 647, 625, 584, 564, 513, 468. **HRMS** (ESI, m/z): $[M+H]^+$ calc. for $C_{29}H_{29}N_2O_2S$, 469.195; found, 469.196.

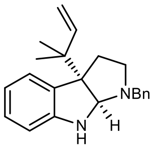

(3a*S*,8a*R*)-1-benzyl-3a-(2-methylbut-3-en-2-yl)-1,2,3,3a,8,8a-hexahydropyrrolo-[2,3-*b*]indole
(+)-3k

Prepared according to **GP 7**: *N*-benzyl-2-(1*H*-indol-3-yl)ethan-1-amine (25.0 mg, 0.100 mmol, 1.00 equiv.), **precat. 1** (2.7 mg, 2.5 µmol, 2.5 mol%), (*R*)-**L1** (2.5 mg, 5.0 µmol, 5.0 mol%), MTBD (3 mg, 3 µL, 20 µmol, 20 mol%), BPh_3 (61 mg, 0.25 mmol, 2.5 equiv.), **1a** (93 mg, 97 µL, 0.50 mmol, 5.0 equiv.). Chromatographic purification: silica gel, petroleum ether:EtOAc:NEt_3, 38.5:1:0.5, v/v. Yield: 3.0 mg, 0.0094 mmol, 9%, colorless oil. **HPLC** (OD-H, hexane (0.1% $NHEt_2$):2-propanol, 9:1, v/v, 0.25 mL/min, 254 nm): t_R = 19.3 min (minor), 21.7 min (major), *er* = 1.00/2.44, *ee* = 41.8%. The major enantiomer **(–)-3k**.

Prepared according to **GP 6**: *N*-benzyl-2-(1*H*-indol-3-yl)ethan-1-amine (25.0 mg, 0.100 mmol, 1.00 equiv.), BEt_3 (0.25 mL, 2.5 equiv.), DBU (3 mg, 3 µL, 20 µmol, 20 mol%), **1a** (56 mg, 59 µL, 0.30 mmol, 3.0 equiv.). Chromatographic purification: silica gel, petroleum ether:EtOAc:NEt_3, 38.5:1:0.5, v/v. Yield: 14.5 mg, 0.0455 mmol, 46%, colorless oil. **HPLC** (OD-H, hexane (0.1% $NHEt_2$):2-propanol, 9:1, v/v, 0.25 mL/min, 254 nm): t_R = 19.3 min (major), 21.7 min (minor), *er* = 5.68/1.00, *ee* = 70.0%. $[\alpha]_D^{22}$: +154 (1.0, CH_2Cl_2).

TLC (petroleum ether:EtOAc:NEt_3, 38.5:1:0.5, v/v): R_f = 0.30. **^{1}H-NMR** (400 MHz, $CDCl_3$): δ 7.41–7.37 (m, 2 H), 7.36–7.30 (m, 2 H), 7.29–7.23 (m, 1 H), 7.15 (dd, *J* = 7.4 Hz, 1.3 Hz, 1 H), 7.02 (td, *J* = 7.6 Hz, 1.3 Hz, 1 H), 6.67 (td, *J* = 7.5 Hz, 1.1 Hz, 1 H), 6.51 (dd, *J* = 7.8 Hz, 1.1 Hz, 1 H), 6.04 (dd, *J* = 17.4 Hz, 10.8 Hz, 1 H), 5.07 (dd, *J* = 10.8 Hz, 1.5 Hz, 1 H), 5.00 (dd, *J* = 17.4 Hz, 1.5 Hz, 1 H), 4.60 (s, 1 H), 3.85 (d, *J* = 13.4 Hz, 1 H), 3.79 (d, *J* = 13.4 Hz, 1 H), 3.60 (s_{br}, 1 H), 2.72 (ddd, *J* = 8.9 Hz, 6.8 Hz, 3.2 Hz, 1 H), 2.56 (td, *J* = 8.9 Hz, 5.8 Hz, 1 H), 2.32 (ddd, *J* = 11.9 Hz, 8.9 Hz, 6.8 Hz, 1 H), 1.83 (ddd, *J* = 11.9 Hz, 5.8 Hz, 3.2 Hz, 1 H), 1.05 (s, 3 H), 1.00 (s, 3 H). **^{13}C-NMR** (100 MHz, $CDCl_3$): δ 151.46, 145.48, 139.43, 133.63, 128.73, 128.50, 127.74, 127.08, 125.35, 118.14, 112.84, 108.75, 82.07, 64.47, 54.68, 51.46, 41.79, 34.99, 23.60, 22.80. **IR** (ATR): $\tilde{v}$ 3369, 3081, 3030, 2965, 2871, 2807, 1604, 1486, 1466, 1413, 1379, 1365, 1348, 1313, 1255, 1174, 1147, 1072, 1027, 1005, 945, 913, 738, 699, 462. **HRMS** (ESI, m/z): $[M+H]^+$ calc. for $C_{22}H_{27}N_2$, 319.217; found, 319.218.

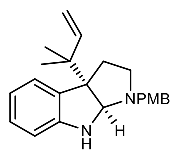

(3a*S*,8a*R*)-1-(4-methoxybenzyl)-3a-(2-methylbut-3-en-2-yl)-1,2,3,3a,8,8a-hexahydropyrrolo[2,3-*b*]indole
(+)-3l

Prepared according to **GP 7**: 2-(1*H*-indol-3-yl)-*N*-(4-methoxybenzyl)ethan-1-amine (28.0 mg, 0.100 mmol, 1.00 equiv.), **precat. 1** (2.7 mg, 2.5 µmol, 2.5 mol%), (*R*)-**L1** (2.5 mg, 5.0 µmol, 5.0 mol%), MTBD (3 mg, 3 µL, 20 µmol, 20 mol%), BPh_3 (61 mg, 0.25 mmol, 2.5 equiv.), **1a** (93 mg, 97 µL, 0.50 mmol, 5.0 equiv.). Chromatographic purification: silica gel, petroleum ether:EtOAc:NEt_3, 95:4.5:0.5, v/v. Yield: 13.8 mg, 0.0396 mmol, 40%, colorless oil. **HPLC** (OD-H, hexane (0.1% $NHEt_2$):2-propanol, 9:1, v/v, 0.5 mL/min, 254 nm): t_R = 9.8 min (major), 10.5 min (minor), *er* = 1.42/1.00, *ee* = 17.2%. The major enantiomer is **(–)-3l**.

Prepared according to **GP 6**: 2-(1*H*-indol-3-yl)-*N*-(4-methoxybenzyl)ethan-1-amine (28.0 mg, 0.100 mmol, 1.00 equiv.), BEt_3 (0.25 mL, 2.5 equiv.), DBU (3 mg, 3 µL, 20 µmol, 20 mol%), **1a** (56 mg,

59 µL, 0.30 mmol, 3.0 equiv.). Chromatographic purification: silica gel, petroleum ether:EtOAc:NEt_3, 95:4.5:0.5, v/v. Yield: 22.5 mg, 0.0646 mmol, 65%, colorless oil. **HPLC** (OD-H, hexane (0.1% $NHEt_2$):2-propanol, 9:1, v/v, 0.5 mL/min, 254 nm): t_R = 9.8 min (minor), 10.5 min (major), *er* = 1.00/7.13, *ee* = 75.5%. **$[\alpha]_D^{22}$**: +152 (1.0 CH_2Cl_2).

TLC (petroleum ether:EtOAc:NEt_3, 95:4.5:0.5, v/v): R_f = 0.20. **^{1}H-NMR** (400 MHz, $CDCl_3$): δ 7.33–7.27 (m, 2 H), 7.13 (dd, *J* = 7.5 Hz, 1.2 Hz, 1 H), 7.01 (td, *J* = 7.6 Hz, 1.2 Hz, 1 H), 6.90–6.85 (m, 2 H), 6.67 (td, *J* = 7.5 Hz, 1.1 Hz, 1 H), 6.51 (dd, *J* = 7.9 Hz, 1.1 Hz, 1 H), 6.03 (dd, *J* = 17.4 Hz, 10.8 Hz, 1 H), 5.07 (dd, *J* = 10.8 Hz, 1.5 Hz, 1 H), 5.00 (dd, *J* = 17.4 Hz, 1.5 Hz, 1 H), 4.56 (s, 1 H), 3.82 (s, 3 H), 3.78 (d, *J* = 13.1 Hz, 1 H), 3.72 (d, *J* = 13.1 Hz, 1 H), 2.69 (ddd, *J* = 8.9 Hz, 6.8 Hz, 3.6 Hz, 1 H), 2.55 (ddd, *J* = 8.9 Hz, 8.6 Hz, 5.8 Hz, 1 H), 2.31 (ddd, *J* = 12.0 Hz, 8.6 Hz, 6.8 Hz, 1 H), 1.82 (ddd, *J* = 12.0 Hz, 5.8 Hz, 3.6 Hz, 1 H), 1.05 (s, 3H), 0.99 (s, 3H). **^{13}C-NMR** (100 MHz, $CDCl_3$): δ 158.76, 151.40, 145.49, 133.67, 131.40, 129.82, 127.71, 125.36, 118.09, 113.89, 112.81, 108.73, 82.14, 64.39, 55.40, 54.22, 51.46, 41.77, 34.96, 23.56c, 22.79. **IR** (ATR): $\tilde{v}$ 3388, 2963, 2930, 2872, 2833, 1725, 1606, 1586, 1512, 1486, 1465, 1414, 1379, 1365, 1348, 1300, 1246, 1171, 1147, 1103, 1036, 1007, 914, 819, 742. **HRMS** (ESI, m/z): $[M+H]^+$ calc. for $C_{23}H_{29}N_2O$, 349.228; found, 349.231.

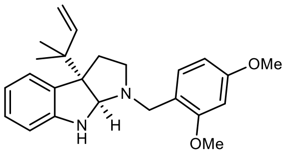

(3a*S*,8a*R*)-1-(2,4-dimethoxybenzyl)-3a-(2-methylbut-3-en-2-yl)-1,2,3,3a,8,8a-hexahydropyrrolo[2,3-*b*]indole
(+)-3m

Prepared according to **GP 7**: *N*-(2,4-dimethoxybenzyl)-2-(1*H*-indol-3-yl)ethan-1-amine (31.0 mg, 0.100 mmol, 1.00 equiv.), **precat. 1** (2.7 mg, 2.5 µmol, 2.5 mol%), (*R*)-**L1** (2.5 mg, 5.0 µmol, 5.0 mol%), MTBD (3 mg, 3 µL, 20 µmol, 20 mol%), BPh_3 (61 mg, 0.25 mmol, 2.5 equiv.), **1a** (93 mg, 97 µL, 0.50 mmol, 5.0 equiv.). Chromatographic purification: silica gel, petroleum ether:EtOAc:NEt_3, 90:9.5:0.5, v/v. Yield: 6.0 mg, 0.0159 mmol, 16%, colorless oil. **HPLC** (AD-H, hexane (0.1% $NHEt_2$):2-propanol, 9:1, v/v, 0.5 mL/min, 254 nm): t_R = 10.5 min (major), 11.2 min (minor), *er* = 1.10/1.00, *ee* = 4.6%.

Prepared according to **GP 6**: *N*-(2,4-dimethoxybenzyl)-2-(1*H*-indol-3-yl)ethan-1-amine (31.0 mg, 0.100 mmol, 1.00 equiv.), BEt_3 (0.25 mL, 2.5 equiv.), DBU (3 mg, 3 µL, 20 µmol, 20 mol%), **1a** (56 mg, 59 µL, 0.30 mmol, 3.0 equiv.). Chromatographic purification: silica gel, petroleum ether:EtOAc:NEt_3, 90:9.5:0.5, v/v. Yield: 27.4 mg, 0.0724 mmol, 72%, colorless oil. **HPLC** (AD-H, hexane (0.1% $NHEt_2$):2-propanol, 9:1, v/v, 0.5 mL/min, 254 nm): t_R = 10.5 min (major), 11.2 min (minor), *er* = 11.6/1.00, *ee* = 84.1%. **$[\alpha]_D^{22}$**: +176 (1.0, CH_2Cl_2).

TLC (petroleum ether:EtOAc:NEt_3, 90:9.5:0.5, v/v): R_f = 0.20. **^{1}H-NMR** (400 MHz, $CDCl_3$): δ 7.30–7.25 (m, 1 H), 7.12 (dd, *J* = 7.5 Hz, 1.3 Hz, 1 H), 7.01 (td, *J* = 7.5 Hz, 1.3 Hz, 1 H), 6.65 (td, *J* = 7.5 Hz, 1.1 Hz, 1 H), 6.53–6.45 (m, 3 H), 6.02 (dd, *J* = 17.4 Hz, 10.9 Hz, 1 H), 5.04 (dd, *J* = 10.9 Hz, 1.5 Hz, 1 H), 4.99 (dd, *J* = 17.4 Hz, 1.5 Hz, 1 H), 4.54 (s, 1 H), 3.88–3.83 (m, 4 H), 3.82 (s, 3 H), 3.68 (d,

J = 13.8 Hz, 1 H), 2.72–2.57 (m, 2 H), 2.30 (dt, J = 12.1 Hz, 7.2 Hz, 1 H), 1.83 (ddd, J = 12.1 Hz, 6.0 Hz, 4.5 Hz, 1 H), 1.05 (s, 3 H), 0.99 (s, 3 H). **^{13}C-NMR** (100 MHz, $CDCl_3$): δ 159.96, 158.42, 151.33, 145.61, 133.77, 131.08, 127.66, 125.41, 119.57, 117.78, 112.67, 108.51, 104.37, 98.67, 82.41, 64.08, 55.64, 55.49, 51.44, 47.79, 41.71, 34.97, 23.53, 22.80. **IR** (ATR): $\tilde{\nu}$ 3394, 2962, 2834, 1605, 1587, 1505, 1485, 1463, 1438, 1414, 1379, 1364, 1350, 1311, 1288, 1254, 1205, 1153, 1133, 1109, 1036, 1005, 908, 832, 801, 732, 692, 671, 647, 558, 544, 521, 465. **HRMS** (ESI, m/z): $[M+H]^+$ calc. for $C_{24}H_{31}N_2O_2$, 379.239; found, 379.240.

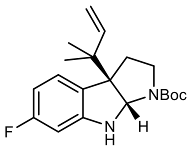

tert-butyl (3a*R*,8a*S*)-6-fluoro-3a-(2-methylbut-3-en-2-yl)-3,3a,8,8a-tetrahydro-pyrrolo[2,3-*b*]indole-1(2*H*)-carboxylate
(–)-4a

<u>Prepared according to **GP 7**:</u> *tert*-butyl (2-(6-fluoro-1*H*-indol-3-yl)ethyl)carbamate (27.8 mg, 0.100 mmol, 1.00 equiv.), **precat. 1** (2.7 mg, 2.5 µmol, 2.5 mol%), (*R*)-**L1** (2.5 mg, 5.0 µmol, 5.0 mol%), MTBD (3 mg, 3 µL, 20 µmol, 20 mol%), BPh_3 (31 mg, 0.13 mmol, 1.3 equiv.), **1a** (93 mg, 97 µL, 0.50 mmol, 5.0 equiv.). Chromatographic purification: silica gel, petroleum ether:EtOAc:NEt_3, 98.5:1:0.5, v/v. Yield: 29.0 mg, 0.0837 mmol, 84%, colorless solid. **mp**: 96 °C. **HPLC** (AD-H, hexane:2-propanol, 9:1, v/v, 1.0 mL/min, 254 nm): t_R = 4.8 min (major), 6.3 min (minor), *er* = 42.7/1.00, *ee* = 95.4%. **$[\alpha]_D^{20}$**: –340 (1.0, CH_2Cl_2).

<u>Prepared according to **GP 6**:</u> *tert*-butyl (2-(6-fluoro-1*H*-indol-3-yl)ethyl)carbamate (27.8 mg, 0.100 mmol, 1.00 equiv.), BEt_3 (0.13 mL, 1.3 equiv.), DBU (3 mg, 3 µL, 20 µmol, 20 mol%), **1a** (56 mg, 59 µL, 0.30 mmol, 3.0 equiv.). Chromatographic purification: silica gel, petroleum ether:EtOAc:NEt_3, 98.5:1:0.5, v/v. Yield: 34.5 mg, 0.0996 mmol, 100%, colorless solid. **mp**: 94 °C. **HPLC** (AD-H, hexane:2-propanol, 9:1, v/v, 1.0 mL/min, 254 nm): t_R = 4.8 min, 6.3 min, *er* = 1.00/1.00, *ee* = 0.0%.

In $CDCl_3$ at 20 °C **4a** exhibits two carbamate-rotamers (A/B = 0.6/0.4). In case the resonance signals show a splitting and an assignment is possible the respective rotamer is denoted by the subscript A/B.

TLC (petroleum ether:EtOAc:NEt_3, 98.5:1:0.5, v/v): R_f = 0.25. **^{1}H-NMR** (400 MHz, $CDCl_3$): δ 7.02–6.94 (m, 1 H), 6.42–6.31 (m, 1 H), 6.28–6.22 (m, 1 H), 6.04–5.93 (m, 1 H), 5.28_A (s, 0.6 H), 5.17_B (s, 0.4 H), 5.14–4.98 (m, 2.6 H), 4.62_B (s_{br}, 0.4 H), 3.68_B (dd, J = 10.7 Hz, 8.1 Hz, 0.4 H), 3.54_A (dd, J = 10.7 Hz, 8.1 Hz, 0.6 H), 2.97–2.84 (m, 1 H), 2.33–2.19 (m, 1 H), 1.93 (dd, J = 12.4 Hz, 6.5 Hz, 1 H), 1.52_B (s, 3.6 H), 1.44_A (s, 5.4 H), 1.08–1.04 (m, 3 H), 0.99 (s, 3 H). **^{13}C-NMR** (100 MHz, $CDCl_3$): δ 163.90_A (d, J = 242 Hz), 163.87_B (d, J = 242 Hz), 154.49_A, 153.34_B, 152.09_A (d, J = 11.9 Hz), 151.64_B (d, J = 11.9 Hz), 144.32, 125.91–125.74_{A+B} (2×d)[h], 125.61_B (d, J = 10.4 Hz), 125.46_A (d, J = 10.4 Hz), 113.88, 104.71_B (d, J = 22.7 Hz, 104.16_A (d, J = 22.7 Hz), 96.55 (d, J = 26.1 Hz), 80.44_B, 80.06_A, 78.36_A, 78.24_B, 63.64_B, 62.39_A, 45.88_A, 45.44_B, 41.13, 32.23_A, 31.98_B, 28.81_B, 28.60_A, 23.11, 22.53, 22.44. **^{19}F-NMR** (565 MHz, $CDCl_3$): δ $–114.65_B$ (td, J = 9.5 Hz, 5.7 Hz), $–114.86_A$ (td, J = 9.5 Hz, 5.7 Hz). **IR**

[h] The overlapping resonances belong to C-3b of the hexahydropyrrolo[2,3-*b*]indole core. $^4J_{C\text{-}F}$ ≈ 2.4 Hz.

(ATR): ṽ 3356, 2973, 2933, 2877, 1677, 1613, 1494, 1456, 1398, 1365, 1325, 1288, 1262, 1210, 1163, 1138, 1121, 1104, 1091, 1070, 1049, 1007, 958, 916, 887, 833, 792, 774, 733, 689, 641, 607, 575, 512, 480, 464. **HRMS** (ESI, m/z): $[M+H]^+$ calc. for $C_{20}H_{28}FN_2O_2$, 347.214; found, 347.214.

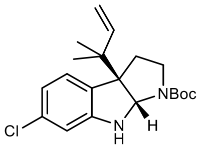

***tert*-butyl (3a*R*,8a*S*)-6-chloro-3a-(2-methylbut-3-en-2-yl)-3,3a,8,8a-tetrahydro-pyrrolo[2,3-*b*]indole-1(2*H*)-carboxylate**
(–)-4b

Prepared according to **GP 7**: *tert*-butyl (2-(6-chloro-1*H*-indol-3-yl)ethyl)carbamate (29.4 mg, 0.100 mmol, 1.00 equiv.), **precat. 1** (2.7 mg, 2.5 µmol, 2.5 mol%), (*R*)-**L1** (2.5 mg, 5.0 µmol, 5.0 mol%), MTBD (3 mg, 3 µL, 20 µmol, 20 mol%), BPh_3 (31 mg, 0.13 mmol, 1.3 equiv.), **1a** (93 mg, 97 µL, 0.50 mmol, 5.0 equiv.). Chromatographic purification: silica gel, petroleum ether:EtOAc:NEt_3, 98.5:1:0.5, v/v. Yield: 31.8 mg, 0.0876 mmol, 88%, colorless glass. **mp**: 59 °C. **HPLC** (AD-H, hexane:2-propanol, 9:1, v/v, 1.0 mL/min, 254 nm): t_R = 4.8 min (major), 5.7 min (minor), *er* = 32.3/1.00, *ee* = 94.0%. $[\alpha]_D^{20}$: –315 (1.0, CH_2Cl_2).

Prepared according to **GP 6**: *tert*-butyl (2-(6-chloro-1*H*-indol-3-yl)ethyl)carbamate (29.4 mg, 0.100 mmol, 1.00 equiv.), BEt_3 (0.13 mL, 1.3 equiv.), DBU (3 mg, 3 µL, 20 µmol, 20 mol%), **1a** (56 mg, 59 µL, 0.30 mmol, 3.0 equiv.). Chromatographic purification: silica gel, petroleum ether:EtOAc:NEt_3, 98.5:1:0.5, v/v. Yield: 36.2 mg, 0.0998 mmol, 100%, colorless solid. **mp**: 119 °C. **HPLC** (AD-H, hexane:2-propanol, 9:1, v/v, 1.0 mL/min, 254 nm): t_R = 4.8 min (minor), 5.7 min (major), *er* = 1.00/1.11, *ee* = 5.2%. The major enantiomer is **(+)-4b**.

In $CDCl_3$ at 20 °C **4b** exhibits two carbamate-rotamers (A/B = 0.6/0.4). In case the resonance signals show a splitting and an assignment is possible the respective rotamer is denoted by the subscript A/B.

TLC (petroleum ether:EtOAc:NEt_3, 98.5:1:0.5, v/v): R_f = 0.25. **^{1}H-NMR** (400 MHz, $CDCl_3$): δ 7.01–6.94 (m, 1 H), 6.70–6.62 (m, 1 H), 6.56–6.50 (m, 1 H), 6.03–5.92 (m, 1 H), 5.27_A (s, 0.6 H), 5.16_B (s, 0.4 H), 5.14–4.98 (m, 2.6 H), 4.61_B (s_{br}, 0.4 H), 3.67_B (dd, *J* = 10.7 Hz, 8.2 Hz, 0.4 H), 3.53_A (dd, *J* = 10.7 Hz, 8.2 Hz, 0.6 H), 2.96–2.82 (m, 1 H), 2.34–2.19 (m, 1 H), 1.93 (dd, *J* = 12.4 Hz, 6.1 Hz, 1 H), 1.51 (s, 3.6 H), 1.43 (s, 5.4 H), 1.08–1.04 (m, 3 H), 0.99 (s, 3 H). **^{13}C-NMR** (100 MHz, $CDCl_3$): δ 154.45_A, 153.29_B, 151.80_A, 151.37_B, 144.17, 134.12_B, 134.05_A, 129.09_A, 129.03_B, 125.78_B, 125.68_A, 118.38_B, 117.86_A, 113.99, 109.02, 80.47_B, 80.10_A, 78.09_A, 77.97_B, 63.86_B, 62.60_A, 45.85_A, 45.43_B, 41.06, 32.15_A, 31.89_B, 28.81_B, 28.59_A, 23.12, 22.52, 22.42. **IR** (ATR): ṽ 3356, 2973, 2932, 2877, 1676, 1638, 1603, 1481, 1450, 1396, 1365, 1314, 1285, 1242, 1209, 1157, 1113, 1095, 1076, 1062, 1049, 1008, 908, 885, 841, 795, 775, 732, 646, 619, 524, 461. **HRMS** (ESI, m/z): $[M+H]^+$ calc. for $C_{20}H_{28}ClN_2O_2$, 363.184; found, 363.185.

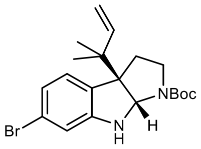

tert-butyl (3a_R_,8a_S_)-6-bromo-3a-(2-methylbut-3-en-2-yl)-3,3a,8,8a-tetrahydro-pyrrolo[2,3-_b_]indole-1(2_H_)-carboxylate
(–)-4c

Prepared according to **GP 7**: _tert_-butyl (2-(6-bromo-1_H_-indol-3-yl)ethyl)carbamate (33.8 mg, 0.100 mmol, 1.00 equiv.), **precat. 1** (5.4 mg, 5.0 µmol, 5.0 mol%), (_R_)-**L1** (5.0 mg, 10 µmol, 10 mol%), MTBD (6 mg, 6 µL, 40 µmol, 40 mol%), tris(4-methoxyphenyl)borane (43 mg, 0.13 mmol, 1.3 equiv.), **1a** (93 mg, 97 µL, 0.50 mmol, 5.0 equiv.). Chromatographic purification: silica gel, petroleum ether:EtOAc:NEt_3, 98.5:1:0.5, v/v. Yield: 36.9 mg, 0.0906 mmol, 91%, colorless solid. **mp**: 125 °C. **HPLC** (AD-H, hexane:2-propanol, 9:1, v/v, 1.0 mL/min, 254 nm): t_R = 4.8 min (major), 5.8 min (minor), _er_ = 61.9/1.00, _ee_ = 96.8%. $[\alpha]_D^{24}$: –282 (1.0, $CHCl_3$).

Prepared according to **GP 6**: _tert_-butyl (2-(6-bromo-1_H_-indol-3-yl)ethyl)carbamate (33.8 mg, 0.100 mmol, 1.00 equiv.), BEt_3 (0.13 mL, 1.3 equiv.), DBU (3 mg, 3 µL, 20 µmol, 20 mol%), **1a** (56 mg, 59 µL, 0.30 mmol, 3.0 equiv.). Chromatographic purification: silica gel, petroleum ether:EtOAc:NEt_3, 98.5:1:0.5, v/v. Yield: 36.9 mg, 0.0906 mmol, 91%, colorless solid. **mp**: 130 °C. **HPLC** (AD-H, hexane:2-propanol, 9:1, v/v, 1.0 mL/min, 254 nm): t_R = 4.8 min (minor), 5.8 min (major), _er_ = 1.00/1.13, _ee_ = 6.3%. The major enantiomer is **(+)-4c**.

In $CDCl_3$ at 20 °C **4c** exhibits two carbamate-rotamers (A/B = 0.6/0.4). In case the resonance signals show a splitting and an assignment is possible the respective rotamer is denoted by the subscript A/B.

TLC (petroleum ether:EtOAc: NEt_3, 98.5:1:0.5, v/v): R_f = 0.25. ^{1}**H-NMR** (500 MHz, $CDCl_3$): δ 6.95–6.90 (m, 1 H), 6.83_B (dd, _J_ = 8.0 Hz, 1.8 Hz, 0.4 H), 6.80_A (dd, _J_ = 8.0 Hz, 1.8 Hz, 0.6 H), 6.70_B (d, _J_ = 1.8 Hz, 0.4 H), 6.69_A (d, _J_ = 1.8 Hz, 0.6 H), 6.02–5.93 (m, 1 H), 5.26_A (s, 0.6 H), 5.15_B (s, 0.4 H), 5.13–5.00 (m, 2.6 H), 4.61_B (s_{br}, 0.4 H), 3.67_B (dd, _J_ = 10.7 Hz, 8.2 Hz, 0.4 H), 3.53_A (dd, _J_ = 10.5 Hz, 8.2 Hz, 0.6 H), 2.95–2.85 (m, 1 H), 2.32–2.22 (m, 1 H), 1.96–1.89 (m, 1 H), 1.51_B (s, 3.6 H), 1.44_A (s, 5.4 H), 1.08–1.02 (m, 3 H), 0.99 (s, 3 H). ^{13}**C-NMR** (125 MHz, $CDCl_3$): δ 154.48_A, 153.30_B, 152.07_A, 151.65_B, 144.16, 129.69, 126.25_B, 126.16_A, 122.16_B, 122.13_A, 121.32_B, 120.80_A, 114.03, 111.93_B, 111.90_A, 80.51_B, 80.14_A, 78.03_A, 77.92_B, 64.00_B, 62.71_A, 45.87_A, 45.45_B, 41.04, 32.15_A, 31.88_B, 28.83_B, 28.61_A, 23.14, 22.54, 22.44. **IR** (ATR): $\tilde{v}$ 3352, 2971, 2930, 2875, 1677, 1599, 1479, 1453, 1397, 1365, 1313, 1257, 1241, 1209, 1159, 1114, 1049, 896, 883, 775, 611, 524, 457. **HRMS** (ESI, m/z): $[M+H]^+$ calc. for $C_{20}H_{28}BrN_2O_2$, 407.133; found, 407.132.

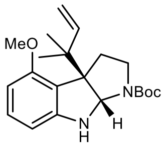

tert-butyl (3a_R_,8a_S_)-4-methoxy-3a-(2-methylbut-3-en-2-yl)-3,3a,8,8a-tetrahydro-pyrrolo[2,3-_b_]indole-1(2_H_)-carboxylate
(–)-5a

Prepared according to **GP 7**: _tert_-butyl (2-(4-methoxy-1_H_-indol-3-yl)ethyl)carbamate (29.0 mg, 0.100 mmol, 1.00 equiv.), **precat. 1** (2.7 mg, 2.5 µmol, 2.5 mol%), (_R_)-**L1** (2.5 mg, 5.0 µmol, 5.0 mol%), MTBD (3 mg, 3 µL, 20 µmol, 20 mol%), BPh_3 (31 mg, 0.13 mmol, 1.3 equiv.), **1a** (93 mg, 97 µL, 0.50 mmol, 5.0 equiv.). Chromatographic purification: silica gel, petroleum ether:EtOAc:NEt_3,

97.5:2:0.5, v/v. Yield: 35.8 mg, 0.0999 mmol, 100%, colorless solid. **mp**: 76 °C. **HPLC** (AD-H, hexane:2-propanol, 9:1, v/v, 1.0 mL/min, 254 nm): t_R = 4.5 min (major), 5.8 min (minor), *er* = 67.5/1.00, *ee* = 97.0%. **[α]$_D^{20}$**: –395 (1.0, CH_2Cl_2).

<u>Prepared according to **GP 6**</u>: *tert*-butyl (2-(4-methoxy-1*H*-indol-3-yl)ethyl)carbamate (29.0 mg, 0.100 mmol, 1.00 equiv.), BEt_3 (0.13 mL, 1.3 equiv.), DBU (3 mg, 3 µL, 20 µmol, 20 mol%), **1a** (56 mg, 59 µL, 0.30 mmol, 3.0 equiv.). Chromatographic purification: silica gel, petroleum ether:EtOAc:NEt_3, 97.5:2:0.5, v/v. Yield: 18.6 mg, 0.0519 mmol, 52%, colorless solid. **mp**: 94 °C. **HPLC** (AD-H, hexane:2-propanol, 9:1, v/v, 1.0 mL/min, 254 nm): t_R = 4.5 min (major), 5.8 min (minor), *er* = 1.37/1.00, *ee* = 15.5%.

In $CDCl_3$ at 20 °C **5a** exhibits two carbamate-rotamers (A/B = 0.6/0.4). In case the resonance signals show a splitting and an assignment is possible the respective rotamer is denoted by the subscript A/B.

TLC (petroleum ether:EtOAc:NEt_3, 97.5:2:0.5, v/v): R_f = 0.30. **^{1}H-NMR** (400 MHz, $CDCl_3$): δ 7.04 (t, *J* = 8.0 Hz, 1 H), 6.30 (t, *J* = 8.0 Hz, 1 H), 6.26–6.20 (m, 1 H), 6.12–6.00 (m, 1 H), 5.14_A (s, 0.6 H), 5.07–4.95 (m, 3 H), 4.53_B (s_{br}, 0.4 H), 3.75 (s, 3 H), 3.64_B (dd, *J* = 10.5 Hz, 8.2 Hz, 0.4 H), 3.50_A (dd, *J* = 10.5 Hz, 8.2 Hz, 0.6 H), 3.02–2.88 (m, 1 H), 2.66 (dt, *J* = 12.7 Hz, 6.4 Hz, 1 H), 2.16–2.01 (m, 1 H), 1.50_B (s, 3.6 H), 1.42_A (s, 5.4 H), 1.16–1.10 (m, 3 H), 1.01 (s, 3 H). **^{13}C-NMR** (100 MHz, $CDCl_3$): δ 157.58_A, 157.52_B, 154.55_A, 153.54_B, 152.91_A, 152.42_B, 145.81_B, 145.78_A, 129.88_B, 129.77_A, 115.29, 112.55_A, 112.43_B, 103.07_A, 102.98_B, 102.51_B, 102.02_A, 80.09_B, 79.65_A, 78.57_A, 78.49_{Bc}, 65.11_B, 63.93_A, 54.85_B, 54.82_A, 46.58_A, 46.17_B, 42.33_A, 42.29_B, 28.84_B, 28.63_A, 28.28_A, 27.95_B, 24.55, 24.47, 23.76_c. **IR** (ATR): $\tilde{v}$ 3358, 2972, 2879, 1681, 1637, 1599, 1489, 1463, 1394, 1366, 1326, 1305, 1271, 1242, 1161, 1109, 1092, 1080, 1059, 1006, 916, 886, 775, 728, 665, 588, 514. **HRMS** (ESI, m/z): $[M+H]^+$ calc. for $C_{21}H_{31}N_2O_3$, 359.234; found, 359.233.

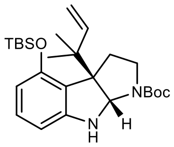

***tert*-butyl (3a*R*,8a*S*)-4-((*tert*-butyldimethylsilyl)oxy)-3a-(2-methylbut-3-en-2-yl)-3,3a,8,8a-tetrahydropyrrolo[2,3-*b*]indole-1(2*H*)-carboxylate (–)-5b**

<u>Prepared according to **GP 7**</u>: *tert*-butyl (2-(4-((*tert*-butyldimethylsilyl)oxy)-1*H*-indol-3-yl)ethyl)carbamate (39.0 mg, 0.100 mmol, 1.00 equiv.), **precat. 1** (2.7 mg, 2.5 µmol, 2.5 mol%), (*R*)-**L1** (2.5 mg, 5.0 µmol, 5.0 mol%), MTBD (3 mg, 3 µL, 20 µmol, 20 mol%), BPh_3 (31 mg, 0.13 mmol, 1.3 equiv.), **1a** (93 mg, 97 µL, 0.50 mmol, 5.0 equiv.). Chromatographic purification: silica gel, petroleum ether:EtOAc:NEt_3, 98.5:1:0.5, v/v. Yield: 43.2 mg, 0.0942 mmol, 94%, colorless solid. **mp**: 120 °C. **HPLC** (AD-H, hexane:2-propanol, 9:1, v/v, 1.0 mL/min, 254 nm): t_R = 3.7 min (major), 4.3 min (minor), *er* = 259/1.00, *ee* = 99.2%. **[α]$_D^{20}$**: –287 (1.0, CH_2Cl_2).

<u>Prepared according to **GP 6**</u>: *tert*-butyl (2-(4-((*tert*-butyldimethylsilyl)oxy)-1*H*-indol-3-yl)ethyl)carbamate (39.0 mg, 0.100 mmol, 1.00 equiv.), BEt_3 (0.13 mL, 1.3 equiv.), DBU (3 mg, 3 µL, 20 µmol, 20 mol%), **1a** (56 mg, 59 µL, 0.30 mmol, 3.0 equiv.). Chromatographic purification: silica gel, petroleum ether:EtOAc:NEt_3, 98.5:1:0.5, v/v. Yield: 40.8 mg, 0.0890 mmol, 89%, colorless oil. **HPLC** (AD-H,

hexane:2-propanol, 9:1, v/v, 1.0 mL/min, 254 nm): t_R = 3.7 min (major), 4.3 min (minor), *er* = 1.42/1.00, *ee* = 17.4%.

In $CDCl_3$ at 20 °C **5b** exhibits two carbamate-rotamers (A/B = 0.6/0.4). In case the resonance signals show a splitting and an assignment is possible the respective rotamer is denoted by the subscript A or B.

TLC (petroleum ether:EtOAc:NEt_3, 98.5:1:0.5, v/v): R_f = 0.20. **^{1}H-NMR** (500 MHz, $CDCl_3$): δ 6.92 (t, *J* = 7.9 Hz, 1 H), 6.27–6.16 (m, 2 H), 6.13–6.04 (m, 1 H), 5.12$_A$ (s, 0.6 H), 5.08–4.97 (m, 3 H), 4.55$_B$ (s$_{br}$, 0.4 H), 3.62$_B$ (dd, *J* = 10.5 Hz, 8.1 Hz, 0.4 H), 3.52$_A$ (dd, *J* = 10.5 Hz, 8.1 Hz, 0.6 H), 3.02–2.93 (m, 1 H), 2.71–2.62 (m, 1 H), 2.14–2.03 (m, 1 H), 1.50$_B$ (s, 3.6 H), 1.43$_A$ (s, 5.4 H), 1.19–1.15 (m, 3 H), 1.03 (s, 3 H), 1.00 (s, 9 H), 0.32–0.29 (m, 3 H), 0.25 (s, 3 H). **^{13}C-NMR** (125 MHz, $CDCl_3$): δ 154.55$_A$, 153.71, 153.67, 153.61, 153.47$_A$, 152.96$_B$, 145.71, 129.42$_B$, 129.31$_A$, 117.13$_B$, 117.09$_A$, 112.90$_A$, 112.78$_B$, 110.25$_B$, 109.68$_A$, 102.77, 80.13$_B$, 79.72$_A$, 78.49$_A$, 78.36$_B$, 64.98$_B$, 63.82$_A$, 46.54$_A$, 46.18$_B$, 42.57$_A$, 42.51$_B$, 28.86$_B$, 28.66$_A$, 28.27$_A$, 27.76$_B$, 26.42, 25.05, 23.75, 18.86$_B$, 18.83$_A$, -3.07 (two signals), –3.58$_A$, –3.61$_B$. **IR** (ATR): $\tilde{v}$ 3417, 2963, 2930, 2898, 2858, 1690, 1591, 1479, 1455, 1416, 1392, 1375, 1365, 1348, 1321, 1286, 1253, 1236, 1174, 1162, 1107, 1055, 1004, 984, 939, 918, 885, 865, 839, 809, 791, 780, 727, 679, 632, 572, 507, 457. **HRMS** (ESI, m/z): $[M+H]^+$ calc. for $C_{26}H_{43}N_2O_3Si$, 459.304; found, 459.304.

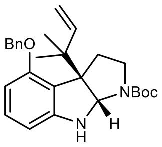

tert-butyl (3a*R*,8a*S*)-4-(benzyloxy)-3a-(2-methylbut-3-en-2-yl)-3,3a,8,8a-tetra-hydropyrrolo[2,3-*b*]indole-1(2*H*)-carboxylate
(–)-5c

Prepared according to **GP 7**: *tert*-butyl (2-(4-(benzyloxy)-1*H*-indol-3-yl)ethyl)carbamate (36.6 mg, 0.100 mmol, 1.00 equiv.), **precat. 1** (2.7 mg, 2.5 µmol, 2.5 mol%), (*R*)-**L1** (2.5 mg, 5.0 µmol, 5.0 mol%), MTBD (3 mg, 3 µL, 20 µmol, 20 mol%), BPh_3 (31 mg, 0.13 mmol, 1.3 equiv.), **1a** (93 mg, 97 µL, 0.50 mmol, 5.0 equiv.). Chromatographic purification: silica gel, step gradient: petroleum ether:EtOAc:NEt_3, 98.5:1:0.5 → 97.5:2:0.5, v/v. Yield: 43.4 mg, 0.100 mmol, 100%, colorless glass. **mp**: 40–50 °C. **HPLC** (AD-H, hexane:2-propanol, 9:1, v/v, 0.25 mL/min, 254 nm): t_R = 26.4 min (minor), 27.6 min (major), *er* = 1.00/177, *ee* = 98.8%. **$[\alpha]_D^{20}$**: –324 (1.0, CH_2Cl_2).

Prepared according to **GP 6**: *tert*-butyl (2-(4-(benzyloxy)-1*H*-indol-3-yl)ethyl)carbamate (36.6 mg, 0.100 mmol, 1.00 equiv.), BEt_3 (0.13 mL, 1.3 equiv.), DBU (3 mg, 3 µL, 20 µmol, 20 mol%), **1a** (56 mg, 59 µL, 0.30 mmol, 3.0 equiv.). Chromatographic purification: silica gel, step gradient: petroleum ether:EtOAc:NEt_3, 98.5:1:0.5 → 97.5:2:0.5, v/v. Yield: 31.1 mg, 0.0716 mmol, 72%, colorless glass. **mp**: 95–106 °C. **HPLC** (AD-H, hexane:2-propanol, 9:1, v/v, 0.25 mL/min, 254 nm): t_R = 26.4 min (minor), 27.6 min (major), *er* = 1.00/2.10, *ee* = 35.9%.

In $CDCl_3$ at 20 °C **5c** exhibits two carbamate-rotamers (A/B = 0.6/0.4). In case the resonance signals show a splitting and an assignment is possible the respective rotamer is denoted by the subscript A/B.

TLC (petroleum ether:EtOAc:NEt$_3$, 98.5:1:0.5, v/v): R_f = 0.17. **^{1}H-NMR** (500 MHz, $CDCl_3$): δ 7.43–7.29 (m, 5 H), 7.04 (t, *J* = 8.0 Hz, 1 H), 6.41–6.34 (m, 1 H), 6.29–6.24 (m, 1 H), 6.11–6.02 (m, 1 H), 5.16$_A$ (s, 0.6 H), 5.11–4.94 (m, 5 H), 4.57$_B$ (s$_{br}$, 0.4 H), 3.64$_B$ (dd, *J* = 10.5 Hz, 8.2 Hz, 0.4 H), 3.51$_A$ (dd, *J* = 10.5 Hz, 8.2 Hz, 0.6 H), 3.05–2.93 (m, 1 H), 2.75–2.65 (m, 1 H), 2.13–2.01 (m, 1 H), 1.51$_B$ (s, 3.6 H), 1.43$_A$ (s, 5.4 H), 1.16–1.12 (m, 3 H), 1.04 (s, 3 H). **^{13}C-NMR** (125 MHz, $CDCl_3$): δ 156.80$_A$, 156.73$_B$, 154.55$_A$, 153.54$_B$, 153.13$_A$, 152.63$_B$, 145.78$_B$, 145.76$_A$, 137.38$_A$, 137.24$_B$, 129.94$_B$, 129.83$_A$, 128.61, 127.91$_B$, 127.88$_A$, 127.49$_B$, 127.45$_A$, 115.42$_B$, 115.38$_A$, 112.64$_A$, 112.53$_B$, 103.52$_B$, 103.32$_A$, 103.22$_B$, 102.99$_A$, 80.12$_B$, 79.68$_A$, 78.62$_A$, 78.53$_B$, 69.98$_B$, 69.94$_A$, 65.18$_B$, 64.01$_A$, 46.64$_A$, 46.22$_B$, 42.41$_A$, 42.38$_B$, 28.84$_B$, 28.63$_A$, 28.28$_A$, 27.96$_B$, 24.84$_B$, 24.75$_A$, 23.83, 23.81. **IR** (ATR): $\tilde{v}$ 3401, 2972, 2930, 2876, 1736, 1679, 1637, 1596, 1486, 1455, 1393, 1365, 1324, 1304, 1270, 1238, 1159, 1106, 1067, 1030, 1006, 982, 938, 914, 885, 844, 816, 774, 728, 697, 673, 644, 598, 580, 515, 460. **HRMS** (ESI, m/z): $[M+H]^+$ calc. for $C_{27}H_{35}N_2O_3$, 435.265; found, 435.265.

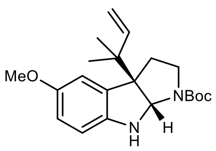

tert-butyl (3a*R*,8a*S*)-5-methoxy-3a-(2-methylbut-3-en-2-yl)-3,3a,8,8a-tetra-hydropyrrolo[2,3-*b*]indole-1(2*H*)-carboxylate
(–)-6a

Prepared according to **GP 7**: *tert*-butyl (2-(5-methoxy-1*H*-indol-3-yl)ethyl)carbamate (29.0 mg, 0.100 mmol, 1.00 equiv.), **precat. 1** (2.7 mg, 2.5 µmol, 2.5 mol%), (*R*)-**L1** (2.5 mg, 5.0 µmol, 5.0 mol%), MTBD (3 mg, 3 µL, 20 µmol, 20 mol%), BPh_3 (31 mg, 0.13 mmol, 1.3 equiv.), **1a** (93 mg, 97 µL, 0.50 mmol, 5.0 equiv.). Chromatographic purification: silica gel, step gradient: petroleum ether:EtOAc:NEt$_3$, 97.5:2:0.5 → 95.5:4:0.5, v/v. Additional chromatographic purification: silica gel, petroleum ether:EtOAc, 9:1, v/v. Yield: 33.3 mg, 0.0929 mmol, 93%, colorless solid. **mp**: 69 °C. **HPLC** (AD-H, hexane:2-propanol, 9:1, v/v, 1.0 mL/min, 254 nm): t_R = 6.4 min (major), 11.3 min (minor), *er* = 43.1/1.00, *ee* = 95.5%. **$[\alpha]_D^{20}$**: –304 (1.0, CH_2Cl_2).

Prepared according to **GP 6**: *tert*-butyl (2-(5-methoxy-1*H*-indol-3-yl)ethyl)carbamate (29.0 mg, 0.100 mmol, 1.00 equiv.), BEt_3 (0.13 mL, 1.3 equiv.), DBU (3 mg, 3 µL, 20 µmol, 20 mol%), **1a** (56 mg, 59 µL, 0.30 mmol, 3.0 equiv.). Chromatographic purification: silica gel, step gradient: petroleum ether:EtOAc:NEt$_3$, 97.5:2:0.5 → 95.5:4:0.5, v/v. Additional chromatographic purification: silica gel, petroleum ether:EtOAc, 9:1, v/v. Yield: 13.4 mg, 0.0374 mmol, 37%, colorless oil. **HPLC** (AD-H, hexane:2-propanol, 9:1, v/v, 1.0 mL/min, 254 nm): t_R = 6.4 min (major), 11.3 min (minor), *er* = 1.38/1.00, *ee* = 15.9%.

In $CDCl_3$ at 20 °C **6a** exhibits two carbamate-rotamers (A/B = 0.6/0.4). In case the resonance signals show a splitting and an assignment is possible the respective rotamer is denoted by the subscript A/B.

TLC (petroleum ether:EtOAc:NEt$_3$, 97.5:2:0.5, v/v): R_f = 0.20; (petroleum ether:EtOAc, 9:1, v/v): R_f = 0.33. **^{1}H-NMR** (400 MHz, $CDCl_3$): δ 6.73 (d, *J* = 2.6 Hz, 1 H, H-4), 6.65 (dd, *J* = 8.4 Hz, 2.6 Hz, 1 H, H-6), 6.50 (d, *J* = 8.4 Hz, 1 H, H-7), 6.07–5.96 (m, 1 H, H-13), 5.26$_A$ (s, 0.6 H, H-2), 5.15$_B$ (s, 0.4 H, H-2), 5.13–5.00 (m, 2 H, H-14), 4.92–4.25 (m, 1 H, H-1), 3.75 (s, 3 H, H-19), 3.67$_B$ (dd, *J* = 10.6 Hz, 8.2 Hz,

0.4 H, H-10), 3.53$_A$ (dd, J = 10.6 Hz, 8.2 Hz, 0.6 H, H-10), 2.96–2.83 (m, 1 H, H-10), 2.32–2.19 (m, 1 H, H-11), 2.00–1.91 (m, 1 H, H-11), 1.52$_B$ (s, 3.6 H, H-18), 1.43$_A$ (s, 5.4 H, H-18), 1.10–1.06 (m, 3 H, H-15/H-15‘), 1.01 (s, 3 H, H-15/H-15‘). **^{13}C-NMR** (100 MHz, $CDCl_3$): δ 154.51$_A$, 153.51$_B$, 153.29$_B$, 152.95$_A$, 144.82$_A$, 144.60$_A$, 144.56$_B$, 144.32$_B$, 132.25$_A$, 132.14$_B$, 113.74, 113.02$_B$, 112.84$_A$, 112.56, 109.36$_B$, 109.30$_A$, 80.28$_B$, 79.86$_A$, 78.60$_A$, 78.51$_B$, 64.64$_B$, 63.43$_A$, 56.15, 45.88$_A$, 45.45$_B$, 41.08, 32.12$_A$, 31.89$_B$, 28.85$_B$, 28.64$_A$, 23.26 (two signals), 22.72$_B$, 22.63. **IR** (ATR): $\tilde{v}$ 2972, 2931, 1683, 1491, 1455, 1434, 1393, 1365, 1321, 1299, 1282, 1246, 1204, 1160, 1119, 1042, 916, 882, 805, 775, 733. **HRMS** (ESI, m/z): $[M+H]^+$ calc. for $C_{21}H_{31}N_2O_3$, 359.234; found, 359.233.

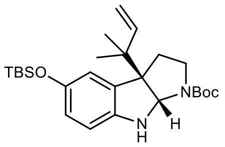

tert-butyl (3a*R*,8a*S*)-5-((_tert_-butyldimethylsilyl)oxy)-3a-(2-methylbut-3-en-2-yl)-3,3a,8,8a-tetrahydropyrrolo[2,3-*b*]indole-1(2*H*)-carboxylate (–)-6b

Prepared according to **GP 7**: *tert*-butyl (2-(5-((*tert*-butyldimethylsilyl)oxy)-1*H*-indol-3-yl)ethyl)carbamate (39.0 mg, 0.100 mmol, 1.00 equiv.), **precat. 1** (2.7 mg, 2.5 µmol, 2.5 mol%), (*R*)-**L1** (2.5 mg, 5.0 µmol, 5.0 mol%), MTBD (3 mg, 3 µL, 20 µmol, 20 mol%), BPh_3 (31 mg, 0.13 mmol, 1.3 equiv.), **1a** (93 mg, 97 µL, 0.50 mmol, 5.0 equiv.). Chromatographic purification: silica gel, petroleum ether:EtOAc:NEt_3, 98.5:1:0.5, v/v. Yield: 44.0 mg, 0.0959 mmol, 96%, colorless solid. **mp**: 71 °C. **HPLC** (AD-H, hexane:2-propanol, 9:1, v/v, 1.0 mL/min, 254 nm): t_R = 4.1 min (major), 9.2 min (minor), *er* = 59.5/1.00, *ee* = 96.7%. **$[\alpha]_D^{20}$**: –259 (1.0, CH_2Cl_2).

Prepared according to **GP 6**: *tert*-butyl (2-(5-((*tert*-butyldimethylsilyl)oxy)-1*H*-indol-3-yl)ethyl)carbamate (39.0 mg, 0.100 mmol, 1.00 equiv.), BEt_3 (0.13 mL, 1.3 equiv.), DBU (3 mg, 3 µL, 20 µmol, 20 mol%), **1a** (56 mg, 59 µL, 0.30 mmol, 3.0 equiv.). Chromatographic purification: silica gel, petroleum ether:EtOAc:NEt_3, 98.5:1:0.5, v/v. Yield: 25.0 mg, 0.0545 mmol, 55%, colorless oil. **HPLC** (AD-H, hexane:2-propanol, 9:1, v/v, 1.0 mL/min, 254 nm): t_R = 4.1 min (major), 9.2 min (minor), *er* = 1.56/1.00, *ee* = 22.0%.

In $CDCl_3$ at 20 °C **6b** exhibits two carbamate-rotamers (A/B = 0.6/0.4). In case the resonance signals show a splitting and an assignment is possible the respective rotamer is denoted by the subscript A/B.

TLC (petroleum ether:EtOAc:NEt_3, 98.5:1:0.5, v/v): R_f = 0.20. **^{1}H-NMR** (500 MHz, $CDCl_3$): δ 6.62 (d, J = 2.4 Hz, 1 H), 6.60–6.56 (m, 1 H), 6.43 (dd, J = 8.2 Hz, 3.6 Hz, 1 H), 6.05–5.95 (m, 1 H), 5.26$_A$ (s, 0.6 H), 5.14$_B$ (s, 0.4 H), 5.11–4.98 (m, 2 H), 4.71$_A$ (s_{br}, 0.6 H), 4.28$_B$ (s_{br}, 0.4 H), 3.66$_B$ (dd, J = 10.6 Hz, 8.1 Hz, 0.4 H), 3.54$_A$ (dd, J = 10.6 Hz, 8.1 Hz, 0.6 H), 2.88 (tt, J = 11.3 Hz, 5.7 Hz, 1 H), 2.24 (tt, J = 11.8 Hz, 8.3 Hz, 1 H), 1.93 (dt, J = 12.4 Hz, 6.2 Hz, 1 H), 1.51$_B$ (s, 3.6 H), 1.44$_A$ (s, 5.4 H), 1.09–1.05 (m, 3 H), 1.01 (s, 3 H), 0.97 (s, 9 H), 0.15 (s, 3 H), 0.14 (s, 3 H). **^{13}C-NMR** (125 MHz, $CDCl_3$): δ 154.49$_A$, 153.55$_B$, 148.43$_B$, 148.07$_A$, 145.08$_A$, 144.64$_B$, 144.58$_A$, 144.55$_B$, 131.76$_A$, 131.68$_B$, 119.87$_B$, 119.70$_A$, 117.47$_B$, 117.37$_A$, 113.67$_A$, 113.65$_B$, 109.49$_B$, 109.44$_A$, 80.28$_B$, 79.87$_A$, 78.57$_A$, 78.50$_B$, 64.53$_B$, 63.35$_A$, 45.89$_A$, 45.49$_B$, 41.12$_A$, 41.06$_B$, 32.22$_A$, 31.85$_B$, 28.86$_B$, 28.65$_A$, 25.94, 23.29$_A$, 23.24$_B$, 22.67$_B$, 22.56$_A$, 18.38, –4.27, –4.33. **IR** (ATR): $\tilde{v}$ 2960, 2930, 2884, 2858, 1685, 1485, 1391, 1365, 1280, 1250,

1198, 1159, 1121, 1051, 1007, 966, 934, 916, 888, 858, 837, 810, 778, 732. **HRMS** (ESI, m/z): $[M+H]^+$ calc. for $C_{26}H_{43}N_2O_3Si$, 459.304; found, 459.304.

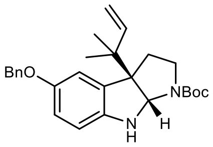

tert-butyl (3a_R_,8a_S_)-5-(benzyloxy)-3a-(2-methylbut-3-en-2-yl)-3,3a,8,8a-tetra-hydropyrrolo[2,3-_b_]indole-1(2_H_)-carboxylate
(–)-6c

Prepared according to **GP 7**: *tert*-butyl (2-(5-(benzyloxy)-1*H*-indol-3-yl)ethyl)carbamate (36.6 mg, 0.100 mmol, 1.00 equiv.), **precat. 1** (2.7 mg, 2.5 µmol, 2.5 mol%), (*R*)-**L1** (2.5 mg, 5.0 µmol, 5.0 mol%), MTBD (3 mg, 3 µL, 20 µmol, 20 mol%), BPh_3 (31 mg, 0.13 mmol, 1.3 equiv.), **1a** (93 mg, 97 µL, 0.50 mmol, 5.0 equiv.). Chromatographic purification: silica gel, step gradient: petroleum ether:EtOAc:NEt_3, 97.5:2:0.5 → 95.5:4:0.5, v/v. Additional chromatographic purification: silica gel, petroleum ether:EtOAc, 9:1, v/v. Yield: 38.3 mg, 0.0881 mmol, 88%, colorless oil. **HPLC** (AD-H, hexane:2-propanol, 9:1, v/v, 1.0 mL/min, 254 nm): t_R = 8.9 min (major), 19.3 min (minor), *er* = 17.3/1.00, *ee* = 89.1%. $[\alpha]_D^{20}$: –253 (1.0, CH_2Cl_2).

Prepared according to **GP 6**: *tert*-butyl (2-(5-(benzyloxy)-1*H*-indol-3-yl)ethyl)carbamate (36.6 mg, 0.100 mmol, 1.00 equiv.), BEt_3 (0.13 mL, 1.3 equiv.), DBU (3 mg, 3 µL, 20 µmol, 20 mol%), **1a** (56 mg, 59 µL, 0.30 mmol, 3.0 equiv.). Chromatographic purification: silica gel, step gradient: petroleum ether:EtOAc:NEt_3, 97.5:2:0.5 → 95.5:4:0.5, v/v. Additional chromatographic purification: silica gel, petroleum ether:EtOAc, 9:1, v/v. Yield: 19.4 mg, 0.0446 mmol, 45%, colorless oil. **HPLC** (AD-H, hexane:2-propanol, 9:1, v/v, 1.0 mL/min, 254 nm): t_R = 8.9 min (major), 19.3 min (minor), *er* = 1.11/1.00, *ee* = 5.2%.

In $CDCl_3$ at 20 °C **6c** exhibits two carbamate-rotamers (A/B = 0.6/0.4). In case the resonance signals show a splitting and an assignment is possible the respective rotamer is denoted by the subscript A/B.

TLC (petroleum ether:EtOAc:NEt_3, 97.5:2:0.5, v/v): R_f = 0.20; (petroleum ether:EtOAc, 9:1, v/v): R_f = 0.33. **^{1}H-NMR** (500 MHz, $CDCl_3$): δ 7.44–7.40 (m, 2 H), 7.40–7.35 (m, 2 H), 7.33–7.29 (m, 1 H), 6.79–6.76 (m, 1 H), 6.74 (dd, *J* = 8.4 Hz, 2.6 Hz, 1 H), 6.52–6.48 (m, 1 H), 6.03–5.94 (m, 1 H), 5.26$_A$ (s, 0.6 H), 5.15$_B$ (s, 0.4 H), 5.10–4.93 (m, 4 H), 4.71 (s_{br}, 1 H), 3.66$_B$ (dd, *J* = 10.6 Hz, 8.2 Hz, 0.4 H), 3.53$_A$ (dd, *J* = 10.6 Hz, 8.2 Hz, 0.6 H), 2.95–2.84 (m, 1 H), 2.29–2.18 (m, 1 H), 1.93 (dd, *J* = 12.4 Hz, 6.1 Hz, 1 H), 1.52$_B$ (s, 3.6 H), 1.44$_A$ (s, 5.4 H), 1.08–1.04 (m, 3 H), 0.99 (s, 3 H). **^{13}C-NMR** (125 MHz, $CDCl_3$): δ 154.51$_A$, 153.50$_B$, 152.39$_B$, 152.09$_A$, 145.11$_A$, 144.62$_B$, 144.57$_A$, 144.54$_B$, 137.67$_A$, 137.59$_B$, 132.12$_A$, 132.01$_B$, 128.64, 127.99, 127.82, 114.74$_B$, 114.53$_A$, 113.91$_B$, 113.88$_A$, 113.73$_A$, 113.70$_B$, 109.34$_B$, 109.27$_A$, 80.28$_B$, 79.87$_A$, 78.57$_A$, 78.49$_B$, 71.50, 64.62$_B$, 63.41$_A$, 45.88$_A$, 45.45$_B$, 41.06$_A$, 41.04$_B$, 32.13$_A$, 31.88$_B$, 28.84$_B$, 28.64$_A$, 23.21, 22.73$_B$, 22.64$_A$. **IR** (ATR): $\tilde{\nu}$ 3392, 2972, 2913, 2876, 1682, 1637, 1489, 1453, 1393, 1366, 1314, 1297, 1281, 1248, 1195, 1160, 1120, 1050, 1026, 915, 882, 845, 807, 775, 735, 697, 634, 572, 502, 462. **HRMS** (ESI, m/z): $[M+H]^+$ calc. for $C_{27}H_{35}N_2O_3$, 435.265; found, 435.265.

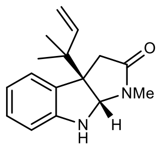

(3a*R*,8a*S*)-1-methyl-3a-(2-methylbut-3-en-2-yl)-3,3a,8,8a-tetrahydropyrrolo[2,3-*b*]-indol-2(1*H*)-one
(–)-7a

Prepared according to **GP 7**: Deviantly on a 0.200 mmol scale. 2-(1*H*-indol-3-yl)-*N*-methylacetamide (37.6 mg, 0.200 mmol, 1.00 equiv.), **precat. 1** (5.4 mg, 5.0 µmol, 2.5 mol%), (*R*)-**L1** (5.0 mg, 10 µmol, 5.0 mol%), MTBD (6 mg, 6 µL, 40 µmol, 20 mol%), BPh_3 (63 mg, 0.26 mmol, 1.3 equiv.), **1a** (0.19 g, 0.19 mL, 1.0 mmol, 5.0 equiv.). Chromatographic purification: silica gel, step gradient: petroleum ether:CH_2Cl_2:NEt_3, 74.5:25:0.5 → 49.5:50:0.5 → 66.5:33:0.5, v/v. Yield: 35.2 mg, 0.137 mmol, 69%, colorless solid. **mp**: 177 °C. **HPLC** (AD-H, hexane:2-propanol, 1:1, v/v, 0.5 mL/min, 254 nm): t_R = 6.8 min (major), 7.3 min (minor), *er* = 12.4/1.00, *ee* = 85.1%. $[\alpha]_D^{22}$: –125 (0.50, CH_2Cl_2).

Prepared according to **GP 6**: Deviantly on a 0.200 mmol scale. 2-(1*H*-indol-3-yl)-*N*-methylacetamide (37.6 mg, 0.200 mmol, 1.00 equiv.), BEt_3 (0.26 mL, 1.3 equiv.), DBU (6 mg, 6 µL, 40 µmol, 20 mol%), **1a** (0.11 g, 0.12 mL, 0.60 mmol, 3.0 equiv.). Chromatographic purification: silica gel, step gradient: petroleum ether:CH_2Cl_2:NEt_3, 74.5:25:0.5 → 49.5:50:0.5 → 66.5:33:0.5, v/v. Yield: 32.3 mg, 0.126 mmol, 63%, colorless solid. **mp**: 162 °C (lit[49] 199–201 °C, from Et_2O/hexane, racemic). **HPLC** (AD-H, hexane:2-propanol, 1:1, v/v, 0.5 mL/min, 254 nm): t_R = 6.8 min (minor), 7.3 min (major), *er* = 1.00/1.09, *ee* = 4.1%. The major enantiomer is **(+)-7a**.

NMR-data from lit[49] are reported in DMSO-d6.

TLC (petroleum ether:CH_2Cl_2:NEt_3, 49.5:50:0.5, v/v): R_f = 0.15. **^{1}H-NMR** (600 MHz, $CDCl_3$): δ 7.13 (d, *J* = 7.5 Hz, 1 H), 7.10 (td, *J* = 7.6 Hz, 1.2 Hz, 1 H), 6.79 (td, *J* = 7.5 Hz, 1.1 Hz, 1 H), 6.64 (d, *J* = 7.6 Hz, 1 H), 5.85 (dd, *J* = 17.3 Hz, 10.8 Hz, 1 H), 5.10 (dd, *J* = 10.8 Hz, 1.2 Hz, 1 H), 5.06 (dd, *J* = 17.3 Hz, 1.2 Hz, 1 H), 5.03 (d, *J* = 2.6 Hz, 1 H), 4.49 (s_{br}, 1 H), 2.88 (d, *J* = 17.2 Hz, 1 H), 2.80 (s, 3 H), 2.58 (d, *J* = 17.2 Hz, 1 H), 1.07 (s, 3 H), 0.97 (s, 3 H). **^{13}C-NMR** (150 MHz, $CDCl_3$): δ 172.66, 148.50, 143.78, 132.91, 128.91, 125.66, 119.97, 114.47, 111.06, 80.13, 57.28, 41.38, 39.80, 26.66, 22.67, 22.12. **IR** (ATR): $\tilde{v}$ 3306, 2967, 2932, 1664, 1606, 1485, 1469, 1416, 1397, 1382, 1366, 1338, 1317, 1291, 1249, 1200, 1112, 1076, 1058, 1010, 979, 914, 737. **HRMS** (ESI, m/z): $[M+H]^+$ calc. for $C_{16}H_{21}N_2O$, 257.165; found, 257.165.

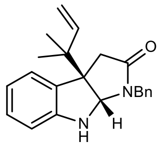

(3a*R*,8a*S*)-1-benzyl-3a-(2-methylbut-3-en-2-yl)-3,3a,8,8a-tetrahydropyrrolo[2,3-*b*]-indol-2(1*H*)-one
(–)-7b

Prepared according to **GP 7**: *N*-benzyl-2-(1*H*-indol-3-yl)acetamide (26.4 mg, 0.100 mmol, 1.00 equiv.), **precat. 1** (2.7 mg, 2.5 µmol, 2.5 mol%), (*R*)-**L1** (2.5 mg, 5.0 µmol, 5.0 mol%), MTBD (3 mg, 3 µL, 20 µmol, 20 mol%), BPh_3 (31 mg, 0.13 mmol, 1.3 equiv.), **1a** (93 mg, 97 µL, 0.50 mmol, 5.0 equiv.). Chromatographic purification: silica gel, step gradient: petroleum ether:CH_2Cl_2:NEt_3, 66.5:33:0.5 → 49.5:50:0.5, v/v. Yield: 32.4 mg, 0.0975 mmol, 98%, colorless solid. **mp**: 131 °C. **HPLC** (AD-H,

hexane:2-propanol, 7:3, v/v, 1.0 mL/min, 254 nm): t_R = 4.9 min (major), 7.7 min (minor), *er* = 29.9/1.00, *ee* = 93.5%. **$[\alpha]_D^{22}$**: –147 (0.50, CH_2Cl_2).

<u>Prepared according to **GP 6**:</u> *N*-benzyl-2-(1*H*-indol-3-yl)acetamide (26.4 mg, 0.100 mmol, 1.00 equiv.), BEt_3 (0.13 mL, 1.3 equiv.), DBU (3 mg, 3 µL, 20 µmol, 20 mol%), **1a** (56 mg, 59 µL, 0.30 mmol, 3.0 equiv.). Chromatographic purification: silica gel, step gradient: petroleum ether:CH_2Cl_2:NEt_3, 66.5:33:0.5 → 49.5:50:0.5, v/v. Yield: 29.3 mg, 0.0881 mmol, 88%, colorless solid. **mp**: 121 °C. **HPLC** (AD-H, hexane:2-propanol, 7:3, v/v, 1.0 mL/min, 254 nm): t_R = 4.9 min (major), 7.7 min (minor), *er* = 1.01/1.00, *ee* = 0.7%.

TLC (petroleum ether:CH_2Cl_2:NEt_3, 49.5:50:0.5, v/v): R_f = 0.50. **^{1}H-NMR** (600 MHz, $CDCl_3$): δ 7.35–7.31 (m, 2 H), 7.30–7.26 (m, 1 H), 7.23–7.19 (m, 2 H), 7.15 (d, *J* = 7.5 Hz, 1 H), 7.09 (td, *J* = 7.6 Hz, 1.2 Hz, 1 H), 6.81 (td, *J* = 7.5 Hz, 1.1 Hz, 1 H), 6.54 (d, *J* = 7.8 Hz, 1 H), 5.80 (dd, *J* = 17.4 Hz, 10.8 Hz, 1 H), 5.06 (dd, *J* = 10.8 Hz, 1.1 Hz, 1 H), 4.99 (dd, *J* = 17.4 Hz, 1.1 Hz, 1 H), 4.99 (d, *J* = 2.7 Hz, 1 H), 4.83 (d, *J* = 15.2 Hz, 1 H), 4.24 (d, *J* = 15.2 Hz, 1 H), 4.17 (s_{br}, 1 H), 2.98 (d, *J* = 17.4 Hz, 1 H), 2.68 (d, *J* = 17.4 Hz, 1 H), 1.03 (s, 3 H), 0.90 (s, 3 H). **^{13}C-NMR** (150 MHz, $CDCl_3$): δ 172.90, 148.51, 143.66, 136.37, 132.99, 128.93, 128.89, 127.90, 127.70, 125.63, 120.06, 114.55, 111.18, 78.02, 57.13, 43.66, 41.48, 39.96, 22.61, 22.09. **IR** (ATR): $\tilde{\nu}$ 3316, 3031, 2967, 2933, 1667, 1607, 1482, 1449, 1415, 1382, 1365, 1340, 1315, 1290, 1259, 1201, 1155, 1096, 1077, 1062, 1028, 1010, 919, 745, 705. **HRMS** (ESI, m/z): $[M+H]^+$ calc. for $C_{22}H_{25}N_2O$, 333.197; found, 333.196.

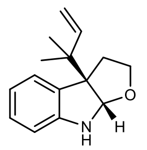

(3a*R*,8a*S*)-3a-(2-methylbut-3-en-2-yl)-3,3a,8,8a-tetrahydro-2*H*-furo[2,3-*b*]indole (–)-8a

<u>Prepared according to **GP 7**:</u> Deviantly on a 0.200 mmol scale. 2-(1*H*-indol-3-yl)ethan-1-ol (32.2 mg, 0.200 mmol, 1.00 equiv.), **precat. 1** (5.4 mg, 5.0 µmol, 2.5 mol%), (*R*)-**L1** (5.0 mg, 10 µmol, 5.0 mol%), MTBD (6 mg, 6 µL, 40 µmol, 20 mol%), BPh_3 (0.12 g, 0.50 mmol, 2.5 equiv.), **1a** (0.19 g, 0.19 mL, 1.0 mmol, 5.0 equiv.). Chromatographic purification: silica gel, CH_2Cl_2. Yield: 33.7 mg, 0.147 mmol, 73%, colorless oil. **HPLC** (AD-H, hexane:2-propanol, 7:3, v/v, 1.0 mL/min, 254 nm): t_R = 5.2 min (minor), 6.6 min (major), *er* = 1.00/41.8, *ee* = 95.3%. **$[\alpha]_D^{20}$**: –109 (1.0, CH_2Cl_2), lit[50] $[\alpha]_D^{23}$: –117.8 (1.0, $CHCl_3$).

<u>Prepared according to **GP 6**:</u> Deviantly on a 0.200 mmol scale. 2-(1*H*-indol-3-yl)ethan-1-ol (32.2 mg, 0.200 mmol, 1.00 equiv.), BEt_3 (0.50 mL, 0.50 mmol, 2.5 equiv.), DBU (6 mg, 6 µL, 40 µmol, 20 mol%), **1a** (0.11 g, 0.12 mL, 0.60 mmol, 3.0 equiv.). Chromatographic purification: silica gel, CH_2Cl_2. Yield: 23.4 mg, 0.102 mmol, 51%, colorless oil. **HPLC** (AD-H, hexane:2-propanol, 7:3, v/v, 1.0 mL/min, 254 nm): t_R = 5.2 min (minor), 6.6 min (major), *er* = 1.00/1.05, *ee* = 2.4%.

TLC (CH_2Cl_2): R_f = 0.30. All analytical data are in accordance with the literature.[50,51]

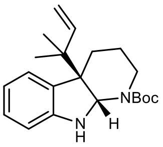

tert-butyl (4a*R*,9a*S*)-4a-(2-methylbut-3-en-2-yl)-2,3,4,4a,9,9a-hexahydro-1*H*-pyrido[2,3-*b*]indole-1-carboxylate
(–)-8b

Prepared according to **GP 7** (with subsequent cyclization): *tert*-butyl (3-(1*H*-indol-3-yl)propyl)carbamate (27.4 mg, 0.100 mmol, 1.00 equiv.), **precat. 1** (2.7 mg, 2.5 µmol, 2.5 mol%), (*R*)-**L1** (2.5 mg, 5.0 µmol, 5.0 mol%), MTBD (3 mg, 3 µL, 20 µmol, 20 mol%), BPh_3 (31 mg, 0.13 mmol, 1.3 equiv.), **1a** (93 mg, 97 µL, 0.50 mmol, 5.0 equiv.). Chromatographic purification: silica gel, petroleum ether:EtOAc:NEt_3, 79.5:20:0.5, v/v. The obtained intermediate was dissolved in CH_2Cl_2 (1 mL) and stored for 12 h at room temperature. After removal of the solvent the product was purified by chromatography: silica gel, petroleum ether:EtOAc:NEt_3, 97.5:2:0.5, v/v. Yield: 31.2 mg, 0.0911 mmol, 91%, colorless oil. **HPLC** (AD-H, hexane:2-propanol, 9:1, v/v, 1.0 mL/min, 254 nm): t_R = 3.8 min (major), 5.3 min (minor), *er* = 30.4/1.00, *ee* = 93.6%. $[\alpha]_D^{20}$: –136 (1.0, CH_2Cl_2).

Prepared according to **GP 6** (with subsequent cyclization): *tert*-butyl (3-(1*H*-indol-3-yl)propyl)carbamate (27.4 mg, 0.100 mmol, 1.00 equiv.), BEt_3 (0.13 mL, 1.3 equiv.), DBU (3 mg, 3 µL, 20 µmol, 20 mol%), **1a** (56 mg, 59 µL, 0.30 mmol, 3.0 equiv.). Chromatographic purification: silica gel, petroleum ether:EtOAc, 75:25, v/v. The obtained intermediate was dissolved in CH_2Cl_2 (1 mL) and stored for 12 h at room temperature. After removal of the solvent the product was purified by chromatography: silica gel, petroleum ether:EtOAc:NEt_3, 97.5:2:0.5, v/v. Yield: 17.9 mg, 0.0523 mmol, 52%, colorless oil. **HPLC** (AD-H, hexane:2-propanol, 9:1, v/v, 1.0 mL/min, 254 nm): t_R = 3.8 min (major), 5.3 min (minor), *er* = 1.19/1.00, *ee* = 8.5%.

In $CDCl_3$ at 20 °C **8b** exhibits two carbamate-rotamers (A/B = 0.55/0.45). In case the resonance signals show a splitting and an assignment is possible the respective rotamer is denoted by the subscript A/B.

TLC (petroleum ether:EtOAc:NEt_3, 97.5:2:0.5, v/v): R_f = 0.20. **^{1}H-NMR** (600 MHz, $CDCl_3$): δ 7.10–7.01 (m, 2 H), 6.71–6.64 (m, 1 H), 6.51 (d, *J* = 7.8 Hz, 1 H), 6.00–5.75 (m, 2 H), 5.08–4.99 (m, 2 H), 4.50–3.89 (m, 1 H), 3.61–3.51 (m, 0.45 H_B), 3.45–3.35 (m, 0.55 H_A), 3.14–3.04 (m, 0.55 H_A), 3.00–2.90 (m, 0.45 H_B), 1.83–1.56 (m, 3.55 H), 1.53–1.44 (m, 9 H), 1.28–1.18 (m, 0.45 H_B), 1.07–1.00 (m, 6 H). **^{13}C-NMR** (150 MHz, $CDCl_3$): δ 156.11, 155.07, 150.05, 145.02_B, 144.78_A, 131.15_B, 130.52_A, 128.14_B, 128.07_A, 125.42_B, 125.18_A, 117.89, 113.43_A, 113.21_B, 108.27_A, 108.02_B, 80.08_B, 79.83_A, 71.41_B, 70.34_A, 55.42_A, 55.38_B, 43.22, 39.42_A, 37.49_B, 28.66_B, 28.59_A, 26.62_A, 25.58_B, 22.77, 22.59, 21.66, 18.53_A, 18.14_B. **IR** (ATR): $\tilde{v}$ 3334, 2972, 2931, 2871, 1693, 1512, 1453, 1415, 1390, 1365, 1270, 1247, 1165, 1040, 1005, 958, 917, 870, 774, 752, 711, 692, 436. **HRMS** (ESI, m/z): $[M+H]^+$ calc. for $C_{21}H_{31}N_2O_2$, 343.239; found, 343.239.

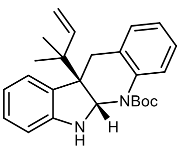

tert-butyl (5a*S*,10b*R*)-10b-(2-methylbut-3-en-2-yl)-5a,6,10b,11-tetrahydro-5*H*-indolo[2,3-*b*]quinoline-5-carboxylate
(–)-9a

Prepared according to **GP 7**: *tert*-butyl (2-((1*H*-indol-3-yl)methyl)phenyl)carbamate (32.2 mg, 0.100 mmol, 1.00 equiv.), **precat. 1** (2.7 mg, 2.5 µmol, 2.5 mol%), (*R*)-**L1** (2.5 mg, 5.0 µmol, 5.0 mol%), MTBD (3 mg, 3 µL, 20 µmol, 20 mol%), BPh_3 (31 mg, 0.13 mmol, 1.3 equiv.), **1a** (93 mg, 97 µL, 0.50 mmol, 5.0 equiv.). Chromatographic purification: silica gel, step gradient: petroleum ether:EtOAc:NEt_3, 94.5:5:0.5 → 89.5:10:0.5, v/v. Yield: 22.0 mg, 0.0563 mmol, 56%, colorless oil. **HPLC** (AD-H, hexane:2-propanol, 9:1, v/v, 1.0 mL/min, 254 nm): t_R = 4.9 min (major), 12.6 min (minor), *er* = 25.5/1.00, *ee* = 92.5%. **$[\alpha]_D^{22}$**: –270 (1.0, CH_2Cl_2).

Prepared according to **GP 6**: *tert*-butyl (2-((1*H*-indol-3-yl)methyl)phenyl)carbamate (32.2 mg, 0.100 mmol, 1.00 equiv.), BEt_3 (0.13 mL, 1.3 equiv.), DBU (3 mg, 3 µL, 20 µmol, 20 mol%), **1a** (56 mg, 59 µL, 0.30 mmol, 3.0 equiv.). Chromatographic purification: silica gel, step gradient: petroleum ether:EtOAc, 95:5 → 90:10, v/v. Yield: 28.4 mg, 0.0727 mmol, 73%, colorless solid. **mp**: 145 °C. **HPLC** (AD-H, hexane:2-propanol, 9:1, v/v, 1.0 mL/min, 254 nm): t_R = 4.9 min (minor), 12.6 min (major), *er* = 1.00/1.44, *ee* = 17.9%. The major enantiomer is **(+)-9a**.

TLC (petroleum ether:EtOAc:NEt_3, 94.5:5:0.5, v/v): R_f = 0.25, (petroleum ether:EtOAc, 90:10, v/v): R_f = 0.25. **^{1}H-NMR** (600 MHz, $CDCl_3$): δ 7.23 (s_{br}, 1 H), 7.01 (ddd, *J* = 8.3 Hz, 5.5 Hz, 2.9 Hz, 1 H), 6.96 (d, *J* = 7.5 Hz, 1 H), 6.87–6.79 (m, 3 H), 6.52 (td, *J* = 7.5 Hz, 1.1 Hz, 1 H), 6.28 (d, *J* = 7.5 Hz, 1 H), 6.15 (s_{br}, 1 H), 6.02 (dd, *J* = 17.2 Hz, 11.0 Hz, 1 H), 5.18–5.12 (m, 2 H), 4.21 (s_{br}, 1 H), 3.00 (d, *J* = 13.6 Hz, 1 H), 2.89 (d, *J* = 13.6 Hz, 1 H), 1.52 (s, 9 H), 1.18 (s, 3 H), 1.13 (s, 3 H). **^{13}C-NMR**[i] (150 MHz, $CDCl_3$): δ 150.50, 144.61, 137.41, 133.02, 129.61, 128.23, 127.79, 126.11, 125.26, 125.05, 124.86, 117.66, 113.83, 107.62, 81.19, 74.06, 62.28, 43.10, 32.21, 28.52, 22.69, 22.11. **IR** (ATR): $\tilde{\nu}$ 3370, 2973, 2930, 1679, 1636, 1608, 1590, 1493, 1470, 1405, 1393, 1367, 1336, 1285, 1242, 1162, 1127, 1078, 1046, 1014, 916, 854, 748, 518, 462. **HRMS** (ESI, m/z): $[M+H]^+$ calc. for $C_{25}H_{31}N_2O_2$, 391.2386; found, 391.2384.

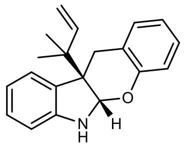

(5a*S*,10b*R*)-10b-(2-methylbut-3-en-2-yl)-5a,6,10b,11-tetrahydrochromeno-[2,3-*b*]indole
(+)-9b

Prepared according to **GP 7**: 2-((1*H*-indol-3-yl)methyl)phenol (22.3 mg, 0.100 mmol, 1.00 equiv.), **precat. 1** (2.7 mg, 2.5 µmol, 2.5 mol%), (*R*)-**L1** (2.5 mg, 5.0 µmol, 5.0 mol%), MTBD (3 mg, 3 µL, 20 µmol, 20 mol%), BPh_3 (31 mg, 0.13 mmol, 1.3 equiv.), **1a** (93 mg, 97 µL, 0.50 mmol, 5.0 equiv.). Chromatographic purification: silica gel, petroleum ether:EtOAc:NEt_3, 94.5:5:0.5, v/v. Yield: 13.1 mg, 0.0450 mmol, 45%, colorless solid. **mp**: 74 °C. **HPLC** (AD-H, hexane:2-propanol, 9:1, v/v, 1.0 mL/min, 254 nm): t_R = 8.5 min (minor), 9.2 min (major), *er* = 1.00/32.5, *ee* = 94.0%. **$[\alpha]_D^{22}$**: +39.8 (0.50, CH_2Cl_2).

Prepared according to **GP 6**: 2-((1*H*-indol-3-yl)methyl)phenol (22.3 mg, 0.100 mmol, 1.00 equiv.), BEt_3 (0.13 mL, 1.3 equiv.), DBU (3 mg, 3 µL, 20 µmol, 20 mol%), **1a** (56 mg, 59 µL, 0.30 mmol, 3.0 equiv.).

[i] The resonance of the carbonyl-carbon atom was not observed.

Chromatographic purification: silica gel, petroleum ether:EtOAc:NEt_3, 94.5:5:0.5, v/v. Yield: 10.8 mg, 0.0371 mmol, 37%, colorless oil. **HPLC** (AD-H, hexane:2-propanol, 9:1, v/v, 1.0 mL/min, 254 nm): t_R = 8.5 min (minor), 9.2 min (major), *er* = 1.00/1.08, *ee* = 3.8%.

TLC (petroleum ether:EtOAc:NEt_3, 94.5:5:0.5, v/v): R_f = 0.25. **^{1}H-NMR** (400 MHz, $CDCl_3$): δ 7.02–6.94 (m, 2 H), 6.91–6.83 (m, 2 H), 6.80 (dd, *J* = 8.0 Hz, 1.2 Hz, 1 H), 6.76 (td, *J* = 7.4 Hz, 1.2 Hz, 1 H), 6.59 (td, *J* = 7.5 Hz, 1.1 Hz, 1 H), 6.38 (dd, *J* = 7.8 Hz, 1.1 Hz, 1 H), 6.05 (dd, *J* = 17.4 Hz, 10.9 Hz, 1 H), 5.70 (s, 1 H), 5.18–5.08 (m, 2 H), 4.56 (s_{br}, 1 H), 3.14 (d, *J* = 14.1 Hz, 1 H), 2.94 (d, *J* = 14.1 Hz, 1 H), 1.18 (s, 3 H), 1.09 (s, 3 H). **^{13}C-NMR** (100 MHz, $CDCl_3$): δ 153.99, 149.86, 144.47, 130.04, 128.47, 128.43, 127.89, 127.04, 125.12, 122.49, 118.15, 117.86, 113.85, 107.46, 93.34, 59.25, 42.29, 31.07, 22.59, 22.34. **IR** (ATR): $\tilde{v}$ 3411, 2962, 2925, 1706, 1609, 1593, 1487, 1455, 1410, 1393, 1377, 1359, 1333, 1260, 1226, 1168, 1076, 1067, 740. **HRMS** (ESI, m/z): $[M+H]^+$ calc. for $C_{20}H_{22}NO$, 292.1701; found, 292.1699.

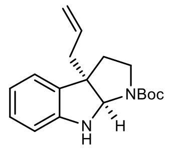

***tert*-butyl (3a*S*,8a*S*)-3a-allyl-3,3a,8,8a-tetrahydropyrrolo[2,3-*b*]indole-1(2*H*)-carboxylate**
(+)-10

Prepared according to **GP 7**: *N*-Boc-Tryptamine (26.0 mg, 0.100 mmol, 1.00 equiv.), **precat. 1** (2.7 mg, 2.5 µmol, 2.5 mol%), (*R*)-**L1** (2.5 mg, 5.0 µmol, 5.0 mol%), MTBD (3 mg, 3 µL, 20 µmol, 20 mol%), BPh_3 (31 mg, 0.13 mmol, 1.3 equiv.), **1a** (93 mg, 97 µL, 0.50 mmol, 5.0 equiv.). Chromatographic purification: silica gel, petroleum ether:EtOAc:NEt_3, 98.5:1:0.5, v/v. Yield: 18.6 mg, 0.0619 mmol, 62%, colorless solid. **mp**: 73 °C. **HPLC** (AD-H, hexane:2-propanol, 9:1, v/v, 1.0 mL/min, 254 nm): t_R = 8.0 min (minor), 15.1 min (major), *er* = 1.00/1.63, *ee* = 24.0%. **$[\alpha]_D^{22}$**: +99.0 (1.0, CH_2Cl_2), lit.[52] $[\alpha]_D^{24.4}$: +337.5 (0.41, CH_2Cl_2, *ee* = 72%).

Prepared according to **GP 6**: *N*-Boc-Tryptamine (26.0 mg, 0.100 mmol, 1.00 equiv.), BEt_3 (0.13 mL, 1.3 equiv.), DBU (3 mg, 3 µL, 20 µmol, 20 mol%), **1b** (47 mg, 0.30 mmol, 3.0 equiv.). Chromatographic purification: silica gel, petroleum ether:EtOAc:NEt_3, 98.5:1:0.5, v/v. Yield: 0.9 mg, 0.003 mmol, 3%, colorless oil. **HPLC** (AD-H, hexane:2-propanol, 9:1, v/v, 1.0 mL/min, 254 nm): t_R = 8.0 min (minor), 15.1 min (major), *er* = 1.00/1.47, *ee* = 19.0%.

TLC (petroleum ether:EtOAc: NEt_3, 98.5:1:0.5, v/v): R_f = 0.20. All analytical data are in accordance with the literature.[52]

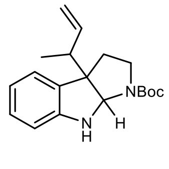

***tert*-butyl 3a-(but-3-en-2-yl)-3,3a,8,8a-tetrahydropyrrolo[2,3-*b*]indole-1(2*H*)-carboxylate**
11

Prepared according to **GP 7**: *N*-Boc-Tryptamine (26.0 mg, 0.100 mmol, 1.00 equiv.), **precat. 1** (2.7 mg, 2.5 µmol, 2.5 mol%), (*R*)-**L1** (2.5 mg, 5.0 µmol, 5.0 mol%), MTBD (3 mg, 3 µL, 20 µmol, 20 mol%), BPh_3 (31 mg, 0.13 mmol, 1.3 equiv.), (*rac*)-**1c** (86 mg, 0.50 mmol, 5.0 equiv.). Chromatographic purification: silica gel, petroleum ether:EtOAc:NEt_3, 98.5:1:0.5, v/v. Yield: 26.0 mg, 0.0827 mmol, 83%, colorless oil. *dr* (diastereomer 1/diastereomer 2) = 1.0/3.0. **HPLC**[j] (AD-H, hexane:2-propanol, 9:1, v/v, 0.5 mL/min, 254 nm): t_R = 10.3 min (diastereomer 2, major), 10.8 min (diastereomer 1, minor), 16.6 min (diastereomer 1, major), 20.6 min (diastereomer 2, minor). diastereomer 1: *er* = 19/1, *ee* = 90.1%; diastereomer 2: *er* = 122/1, *ee* = 98.4%.

Prepared according to **GP 6**: *N*-Boc-Tryptamine (26.0 mg, 0.100 mmol, 1.00 equiv.), BEt_3 (0.13 mL, 1.3 equiv.), DBU (3 mg, 3 µL, 20 µmol, 20 mol%), (*rac*)-**1c** (52 mg, 0.30 mmol, 3.0 equiv.). Chromatographic purification: silica gel, petroleum ether:EtOAc:NEt_3, 98.5:1:0.5, v/v. Yield: 6.0 mg, 0.0191 mmol, 19%, colorless oil. *dr* (diastereomer 1/diastereomer 2) = 2.8/1.0. **HPLC**[j] (AD-H, hexane:2-propanol, 9:1, v/v, 0.5 mL/min, 254 nm): t_R = 10.3 min. (diastereomer 2, major), 10.8 min (diastereomer 1, minor), 16.6 min (diastereomer 1, major), 20.6 min (diastereomer 2, minor). diastereomer 1: *er* = 1.40/1, *ee* = 16.5%; diastereomer 2: *er* = 3.16/1, *ee* = 51.9%.

In case the NMR signals show a splitting and an assignment is possible the respective diastereomer is denoted by the subscript 1/2. Each diastereomer exhibits two carbamate-rotamers (A/B = 0.6/0.4) in $CDCl_3$ at 20 °C. In case the resonance signals show a splitting and an assignment is possible the respective rotamer is denoted by the subscript A/B.

TLC (petroleum ether:EtOAc:NEt_3, 98.5:1:0.5, v/v): R_f = 0.25. **^{1}H-NMR** (600 MHz, $CDCl_3$): δ 7.10–7.01 (m, 2 H), 6.77–6.68 (m, 1 H), 6.60–6.55 (m, 1 H), 5.94–5.86 (m, 1 H_1), 5.65–5.54 (m, 1 H_2), 5.18 (s, 0.6 H_{2A}), 5.14 (s, 0.6 H_{1A}), 5.10–4.94 (m, 3 H), 4.68–4.44 (m, 0.4 H), 3.71–3.64 (m, 0.4 H), 3.59–3.51 (m, 0.6 H), 3.02–2.90 (m, 1 H), 2.50–2.39 (m, 1 H), 2.19–2.05 (m, 2 H), 1.52 (s, 3.6 H), 1.44 (s, 5.4 H), $1.07–1.02_2$ (m, 3 H), 0.95 (d, *J* = 6.8 Hz, 3 H_1). **^{13}C-NMR** (150 MHz, $CDCl_3$): δ 154.58, 153.53, 153.50, 150.62_{1A}, 150.49_{2A}, 150.20_{1B}, 150.09_{2B}, 140.34_2, 139.92_{1A}, 139.85_{1B}, 130.36_{2A}, 130.17_{2B}, 129.79_{1A}, 129.71_{1B}, 128.55, 128.47, 128.44, 124.62_{1B}, 124.54_{1A}, 124.23_{2B}, 124.08_{2A}, 118.86_{2B}, 118.79_{1B}, 118.35_{2A}, 118.28_{1A}, 115.71_{1B}, 115.68_{1A}, 115.58_{2A}, 115.52_{2B}, 109.17, 109.15, 109.11, 109.08, 80.29, 80.27_{1A}, 80.22_{1B}, 79.86, 78.99_{2B}, 78.90_{2A}, 61.03_{2B}, 60.94_{1B}, 59.88_{2A}, 59.79_{1A}, 46.07, 45.97_{2B}, 45.90_{2A}, 45.60, 45.55, 45.53_{1A}, 35.10_{2A}, 34.96_{1A}, 34.85_{2B}, 34.74_{1B}, 28.83, 28.62, 16.32_{2B}, 16.23_{2A}, 15.24_{1B}, 15.21_{1A}. **HRMS** (ESI, m/z): $[M+H]^+$ calc. for $C_{19}H_{27}N_2O_2$, 315.207; found, 315.206.

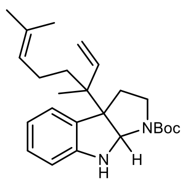

***tert*-butyl 3a-(3,7-dimethylocta-1,6-dien-3-yl)-3,3a,8,8a-tetrahydropyrrolo-[2,3-*b*]indole-1(2*H*)-carboxylate**
12

[j] For the assignment of the signals and the determination of the enantiomeric ratios see section 13.

Prepared according to **GP 7**: *N*-Boc-Tryptamine (26.0 mg, 0.100 mmol, 1.00 equiv.), **precat. 1** (2.7 mg, 2.5 µmol, 2.5 mol%), (*R*)-**L1** (2.5 mg, 5.0 µmol, 5.0 mol%), MTBD (3 mg, 3 µL, 20 µmol, 20 mol%), BPh_3 (31 mg, 0.13 mmol, 1.3 equiv.), (*rac*)-**1d** (0.13 g, 0.50 mmol, 5.0 equiv.). Chromatographic purification: silica gel: petroleum ether:EtOAc:NEt_3, 98.5:1:0.5, v/v. Yield: 32.1 mg, 0.0809 mmol, 81%, colorless oil. *dr* (diastereomer 1/diastereomer 2) = 1.00/1.46.

In case the NMR signals show a splitting and an assignment is possible the respective diastereomer is denoted by the subscript 1/2. Each diastereomer exhibits two carbamate-rotamers (A/B = 0.6/0.4) in $CDCl_3$ at 20 °C. In case the resonance signals show a splitting and an assignment is possible the respective rotamer is denoted by the subscript A/B.

TLC (petroleum ether:EtOAc:NEt_3, 98.5:1:0.5, v/v): R_f = 0.20. **HPLC**[k] (AD-H, hexane:2-propanol, 9:1, v/v, 0.5 mL/min, 254 nm): t_R = 7.6 min (diastereomer 1, major and diastereomer 2, major), 10.4 min (diastereomer 2, minor), 11.2 min (diastereomer 1, minor). diastereomer 1: *er* = 11/1, *ee* = 83.2%; diastereomer 2: *er* = 922/1, *ee* = 99.8%. **^{1}H-NMR** (600 MHz, $CDCl_3$): δ 7.14–7.04 (m, 2 H), 6.76–6.67 (m, 1 H), 6.56 (d, *J* = 7.8 Hz, 1 H), 6.00–5.93 (m, 1 H_1), 5.85–5.76 (m, 1 H_2), 5.34 (s, 0.6 H_A), 5.27–4.95 (m, 4 H), 4.53 (s_{br}, 0.4 H_B), 3.68–3.62 (m, 0.4 H_B), 3.52 (dd, *J* = 10.5 Hz, 8.2 Hz, 0.6 H_A), 2.92–2.82 (m, 1 H), 2.35–2.22 (m, 1 H), 2.02–1.93 (m, 1 H), 1.85–1.71 (m, 2 H), 1.67–1.18 (m, 17 H), 1.10–1.04 (m, 3 H_2), 0.99–0.96 (m, 3 H_1). **^{13}C-NMR** (150 MHz, $CDCl_3$): δ 154.50_{2A}, 154.46_{1A}, 153.47_{1B}, 153.43_{2B}, 150.86, 150.44, 142.78_{2A}, 142.65_{2B}, 142.45_{1B}, 142.28_{1A}, 131.43, 131.35, 131.31, 131.21, 130.67_{1A}, 130.57_{1B}, 130.33_{2A}, 130.23_{2B}, 128.50, 128.47, 128.39, 128.36, 125.48_{2B}, 125.36_{2A}, 125.27_{1B}, 125.13_{1A}, 125.02, 125.00, 124.95, 118.65_{1B}, 118.55_{2B}, 118.09_{1A}, 117.97_{2A}, 115.95_{2B}, 115.85_{2A}, 115.67_{1A}, 115.51_{1B}, $109.12_{1A,1B}$ (two signals), 108.96_{2B}, 108.91_{2A}, 80.30_{2B}, 80.22_{1B}, 79.86_{2A}, 79.82_{1A}, 78.26_{1A}, 78.19_{1B}, 77.33_{2A}, 77.18_{2B}, 65.02, 63.81, 63.78, 45.86_{1A}, 45.74_{2A}, 45.38_{1B}, 45.33_{2B}, 44.92_{2A}, 44.86_{2B}, 44.56_{1B}, 44.54_{1A}, 35.20_{1B}, 35.15_{1A}, $35.05_{2A,2B}$ (two signals), 32.45_{2A}, 32.12_{2B}, 31.98_{1A}, 31.80_{1B}, 28.85, 28.83, 28.62, 25.82, 25.80, 23.15, 23.12, 17.78, 17.76, 17.73, 17.24_{2B}, 17.17_{2A}, 17.11_{1B}, 17.08_{1A}. **HRMS** (ESI, m/z): $[M+H]^+$ calc. for $C_{25}H_{37}N_2O_2$, 397.2855; found, 397.2850.

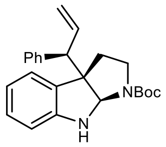

diastereomer 1

tert-butyl (3a*S*,8a*R*)-3a-((*R*)-1-phenylallyl)-3,3a,8,8a-tetrahydropyrrolo-[2,3-*b*]indole-1(2*H*)-carboxylate
13 (diastereomer 1)

k For the assignment of the signals and the determination of the enantiomeric ratios see section 13.

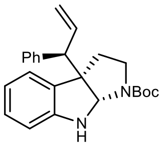

diastereomer 2

tert-butyl (3a_R_,8a_S_)-3a-((_R_)-1-phenylallyl)-3,3a,8,8a-tetrahydropyrrolo-[2,3-_b_]indole-1(2_H_)-carboxylate 13 (diastereomer 2)

Prepared according to **GP 7**: _N_-Boc-Tryptamine (26.0 mg, 0.100 mmol, 1.00 equiv.), **precat. 1** (2.7 mg, 2.5 µmol, 2.5 mol%), (_R_)-**L1** (2.5 mg, 5.0 µmol, 5.0 mol%), MTBD (3 mg, 3 µL, 20 µmol, 20 mol%), BPh_3 (31 mg, 0.13 mmol, 1.3 equiv.), (_rac_)-**1e** (0.12 g, 0.50 mmol, 5.0 equiv.). Chromatographic purification: silica gel, step gradient: petroleum ether:EtOAc:NEt_3, 97.5:2:0.5 → 96.5:3:0.5, v/v. Yield: 35.1 mg, 0.0932 mmol, 93%, colorless oil. _dr_ (diastereomer 1/diastereomer 2) = 1.0/1.1. **HPLC**[I] (AD-H, hexane:2-propanol, 99:1, v/v, 1.0 mL/min, 254 nm): t_R = 7.2 min (diastereomer 2, major), 9.2 min (diastereomer 1, minor), 12.9 min (diastereomer 2, minor), 21.1 min (diastereomer 1, major). diastereomer 1: _er_ = 157/1, _ee_ = 98.7%; diastereomer 2: _er_ = 680/1, _ee_ = 99.7%.

Prepared according to **GP 7**: _N_-Boc-Tryptamine (26.0 mg, 0.100 mmol, 1.00 equiv.), **precat. 1** (2.7 mg, 2.5 µmol, 2.5 mol%), (_R_)-**L1** (2.5 mg, 5.0 µmol, 5.0 mol%), MTBD (3 mg, 3 µL, 20 µmol, 20 mol%), BPh_3 (31 mg, 0.13 mmol, 1.3 equiv.), (_R_)-**1e** (0.12 g, 0.50 mmol, 5.0 equiv.). Chromatographic purification: silica gel, step gradient: petroleum ether:EtOAc:NEt_3, 97.5:2:0.5 → 96.5:3:0.5, v/v. Yield: 35.5 mg, 0.0943 mmol, 94%, colorless oil. _dr_ (diastereomer 1/diastereomer 2) = 1.0/1.2. **HPLC**[I] (AD-H, hexane:2-propanol, 99:1, v/v, 1.0 mL/min, 254 nm): t_R = 7.2 min (diastereomer 2, major), 21.1 min (diastereomer 1, major). diastereomer 1: _er_ = 1/0, _ee_ = >99.9%; diastereomer 2: _er_ = 1/0, _ee_ = >99.9%. (The minor enantiomer of either diastereomer could not be detected).

Prepared according to **GP 8**: _N_-Boc-Tryptamine (26.0 mg, 0.100 mmol, 1.00 equiv.), no additional tetra-butylammonium chloride, (_R_)-**1e** (0.12 g, 0.50 mmol, 5.0 equiv.). Chromatographic purification: silica gel, step gradient: petroleum ether:EtOAc:NEt_3, 97.5:2:0.5 → 96.5:3:0.5, v/v. Yield: 37.5 mg, 0.0997 mmol, 100%, colorless oil. _dr_ (diastereomer 1/diastereomer 2) = 1.7/1.0. **HPLC**[I] (AD-H, hexane:2-propanol, 99:1, v/v, 1.0 mL/min, 254 nm): t_R = 7.2 min (diastereomer 2, major), 9.2 min (diastereomer 1, minor), 12.9 min (diastereomer 2, minor), 21.1 min (diastereomer 1, major). diastereomer 1: _er_ = 45/1, _ee_ = 95.6%; diastereomer 2: _er_ = 8.4/1, _ee_ = 78.6%.

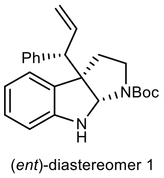

(_ent_)-diastereomer 1

tert-butyl (3a_R_,8a_S_)-3a-((_S_)-1-phenylallyl)-3,3a,8,8a-tetrahydropyrrolo-[2,3-_b_]indole-1(2_H_)-carboxylate 13 (_ent_-diastereomer 1)

[I] For the assignment of the signals and the determination of the enantiomeric ratios see section 13.

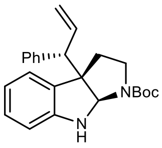

(*ent*)-diastereomer 2

tert-butyl (3a*R*,8a*S*)-3a-((*R*)-1-phenylallyl)-3,3a,8,8a-tetrahydropyrrolo-[2,3-*b*]indole-1(2*H*)-carboxylate 13 (*ent*-diastereomer 2)

Prepared according to **GP 7**: *N*-Boc-Tryptamine (26.0 mg, 0.100 mmol, 1.00 equiv.), **precat. 1** (2.7 mg, 2.5 µmol, 2.5 mol%), (*R*)-**L1** (2.5 mg, 5.0 µmol, 5.0 mol%), MTBD (3 mg, 3 µL, 20 µmol, 20 mol%), BPh_3 (31 mg, 0.13 mmol, 1.3 equiv.), (*S*)-**1e** (0.12 g, 0.50 mmol, 5.0 equiv.). Chromatographic purification: silica gel, step gradient: petroleum ether:EtOAc:NEt_3, 97.5:2:0.5 → 96.5:3:0.5, v/v. Yield: 37.6 mg, 0.100 mmol, 100%, colorless oil. *dr* (*ent*-diastereomer 1/*ent*-diastereomer 2) = 4.5/1.0. **HPLC** (AD-H, hexane:2-propanol, 99:1, v/v, 1.0 mL/min, 254 nm): t_R = 7.2 min (diastereomer 2, minor), 9.2 min (diastereomer 1, major), 12.9 min (diastereomer 2, major), 21.1 min (diastereomer 1, minor). diastereomer 1: *er* = 1/11.2, *ee* = –83.6%; diastereomer 2: *er* = 1/13.3, *ee* = –86.0%.

In case the NMR signals show a splitting and an assignment is possible the respective diastereomer is denoted by the subscript 1/2. Each diastereomer exhibits two carbamate-rotamers (A/B = 0.6/0.4) in $CDCl_3$ at 20 °C. In case the resonance signals show a splitting and an assignment is possible the respective rotamer is denoted by the subscript A/B.

The spectroscopic data of diastereomer 1/(*ent*)-diastereomer 1 are in accordance with the literature.[53]

TLC (petroleum ether:EtOAc:NEt_3, 97.5:2:0.5, v/v): R_f = 0.20. **^{1}H-NMR** (600 MHz, $CDCl_3$): δ 7.30–7.16 (m, 3 H), 7.15–7.12 (m, 1 H), 7.09–7.02 (m, 1 H), 6.93–6.89 (m, 1 H), 6.69–6.61 (m, 2 H), 6.60–6.57 (m, 1 H_2), 6.53–6.49 (m, 1 H_1), 6.34–6.26 (m, 1 H_1), 6.12–6.01 (m, 1 H_2), 5.28–5.06 (m, 3 H), 5.04 (s_{br}, 0.6 H_{2A}), 4.89 (s_{br}, 0.6 H_{1A}), 4.58 (s_{br}, 0.4 H_{2B}), 4.45 (s_{br}, 0.4 H_{1B}), 3.70–3.64 (m, 0.4 H_B), 3.60–3.50 (m, 0.6 H_A, 1 H_1), 3.47 (d, *J* = 9.0 Hz, 1 H_1), 2.98–2.84 (m, 1 H), 2.36–2.23 (m, 1 H), 2.19–2.12 (m, 0.4 H_B), 1.98–1.91 (m, 0.6 H_A), 1.52–1.48 (m, 3.6 H), 1.42 (s, 5.4 H). **^{13}C-NMR** (150 MHz, $CDCl_3$): δ 154.52_{1A}, 154.47_{2A}, 153.55_{1B}, 153.47_{2B}, 150.66_{2A}, 150.32_{2B}, 150.27_{1A}, 149.90_{1B}, 140.88_2, 140.60_1, 137.59_{1B}, 137.50_{2B}, 137.46_{1A}, 137.33_{2A}, 130.18, 130.04, 129.34, 129.33, 128.97, 128.89, 128.73, 128.62, 128.15, 128.11, 128.02, 126.96, 126.87_2, 125.73_{2A}, 125.56_{2B}, 124.71_{1B}, 124.60_{1A}, 118.66_{1B}, 118.48_{2B}, 118.17_{1A}, 117.94_{2A}, 117.78_{1A}, 117.68_{1B}, 117.54_{2A}, 117.43_{2B}, 109.40, 109.29, 109.25, 80.36, 80.30, 80.04_2, 79.90, 79.89_1, 79.12_1, 61.64_{1B}, 61.19_{2B}, 60.61_{1A}, 60.12_{2A}, 57.38_{2B}, 57.27_{2A}, 56.99_{1B}, 56.82_{1A}, 45.96, 45.94, 45.55, 34.71_{2B}, 34.56_{2A}, 33.87_{1A}, 33.80_{1B}, 28.82, 28.61. **HRMS** (ESI, m/z): $[M+H]^+$ calc. for $C_{24}H_{29}N_2O_2$, 377.2229; found, 377.2224.

11. Synthesis of (–)-Flustramine A (14)

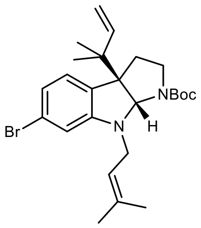

***tert*-butyl (3a*R*,8a*S*)-6-bromo-8-(3-methylbut-2-en-1-yl)-3a-(2-methylbut-3-en-2-yl)-3,3a,8,8a-tetrahydropyrrolo[2,3-*b*]indole-1(2*H*)-carboxylate**
(–)-15

To a solution of (–)-**4c** (163 mg, 400 µmol, 1.00 equiv., *er* = 62/1) in DCE (5 mL) were added 3-methylbut-2-enal (34 mg, 0.40 mmol, 1.0 equiv.) and NaBH(OAc)$_3$ (85 mg, 0.40 mmol, 1.0 equiv.) at room temperature. After stirring for 24 hours there was added another 1 equiv. of each reagent. After another 24 hours there was added 0.5 equiv. of each reagent. After 72 hours overall, the starting material was converted completely (monitored by TLC) and the solution was diluted with saturated NaHCO$_3$ solution (10 mL) and brine (10 mL) and extracted with CH$_2$Cl$_2$ (3×5 mL). The combined organic extracts were dried over MgSO$_4$ and the solvent removed under reduced pressure. The residue was purified by column chromatography: silica gel, petroleum ether:EtOAc, 98:2, v/v. Yield: 185 mg, 388 µmol, 97%, colorless oil.

In CDCl$_3$ at 20 °C **(–)-15** exhibits two carbamate-rotamers, A/B = 0.5/0.5.

TLC (petroleum ether:EtOAc, 98:2, v/v): R_f = 0.20. **[α]$_D$^{22}**: –230 (1.0, CH$_2$Cl$_2$). **^{1}H-NMR** (600 MHz, CDCl$_3$): δ 6.92–6.83 (m, 1 H), 6.70–6.62 (m, 1 H), 6.37–6.31 (m, 1 H), 5.90 (dd, *J* = 17.4 Hz, 10.9 Hz, 1 H), 5.52 (s, 0.5 H), 5.40 (s, 0.5 H), 5.15–5.10 (m, 1 H), 5.09–4.98 (m, 2H), 4.08–3.81 (m, 2.5 H), 3.76–3.69 (m, 0.5 H), 2.90–2.75 (m, 1H), 2.19–2.08 (m, 1 H), 1.79 (dd, *J* = 12.1 Hz, 5.5 Hz, 1 H), 1.72 (s, 3 H), 1.69 (s, 3 H), 1.50–1.41 (m, 9 H), 1.03 (s, 3 H), 0.95 (s, 3 H). **^{13}C-NMR** (150 MHz, CDCl$_3$): δ 154.30, 153.57, 152.54, 152.37, 144.19, 134.06, 133.96, 129.98, 125.74, 122.39, 121.67, 121.28, 118.92, 118.58, 113.90, 108.07, 82.74, 82.09, 80.32, 79.74, 63.66, 62.43, 45.64, 44.87, 43.52, 43.37, 41.36, 34.13, 34.02, 28.54, 25.81, 25.68, 23.39, 23.13, 22.48, 22.26, 18.26, 18.13. **IR** (ATR): $\tilde{\nu}$ 2970, 2930, 2876, 1693, 1596, 1492, 1451, 1392, 1366, 1319, 1247, 1220, 1158, 1142, 1095, 1063, 1045, 1010, 920, 890, 822, 779. **HRMS** (ESI, m/z): [M+H]$^+$ calc. for $C_{25}H_{36}BrN_2O_2$, 475.1960; found, 475.1965.

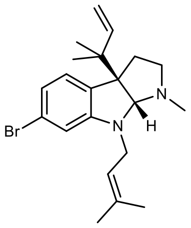

(3a*R*,8a*R*)-6-bromo-1-methyl-8-(3-methylbut-2-en-1-yl)-3a-(2-methylbut-3-en-2-yl)-1,2,3,3a,8,8a-hexahydropyrrolo[2,3-*b*]indole
(–)-Flustramine A (14)

To a solution of **(–)-15** (47.5 mg, 100 µmol, 1.00 equiv.) in MeCN (2 mL) were added at –17 °C 2,6-lutidine (2 mg, 2 µL, 20 µmol, 0.2 equiv.) and trimethylsilyl iodide (24 mg, 16 µL, 0.12 mmol, 1.2 equiv.). After stirring for 45 minutes the solution was diluted with saturated NaHCO$_3$ solution (5 mL)

and brine (5 mL) and extracted with EtOAc (3×5 mL). The combined organic extracts were dried over $MgSO_4$ and the solvent removed under reduced pressure. The residue was dissolved in THF (2 mL) whereupon 12.7 M aqueous formaldehyde solution (24 µL, 0.30 mmol, 3 equiv.) and $NaBH(OAc)_3$ (64 mg, 0.30 mmol, 3 equiv.) were added at room temperature. After stirring for 2 hours the solution was diluted with saturated $NaHCO_3$ solution (5 mL) and brine (5 mL) and extracted with EtOAc (3×5 mL). The combined organic extracts were dried over $MgSO_4$ and the solvent removed under reduced pressure. The residue was purified by coloumn chromatography: silica gel, petroleum ether:EtOAc:NEt_3, 18.5:1:0.5, v/v. Yield: 23.4 mg, 60.2 µmol, 60%, colorless oil.

TLC (petroleum ether:EtOAc:NEt_3, 18.5:1:0.5, v/v): R_f = 0.25.

$[\alpha]_D^{20}$: –148 (1.0, CH_2Cl_2)

$[\alpha]_D^{24}$: –151 (1.0, $CHCl_3$)

$[\alpha]_D^{24}$: –158 (0.1, $CHCl_3$)

literature value ref.[54]: $[\alpha]_D^{20}$: –40.0 (0.1, $CHCl_3$)

HRMS (ESI, m/z): $[M+H]^+$ calc. for $C_{21}H_{30}BrN_2$, 389.159; found, 389.160. **IR** (ATR): $\tilde{\nu}$ 2964, 2928, 2872, 2793, 1731, 1593, 1488, 1376, 1314, 1288, 1256, 1158, 1118, 1046, 912, 823, 798, 781. All NMR-spectroscopic data are in accordance with the literature.[55]

12. NMR spectra

12.1. Precatalysts

^{1}H-NMR (400 MHz, CD_2Cl_2)

$[Ir(COD)Cl_2]_2^{2-}$ 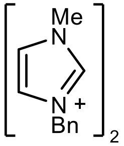

precat. 1

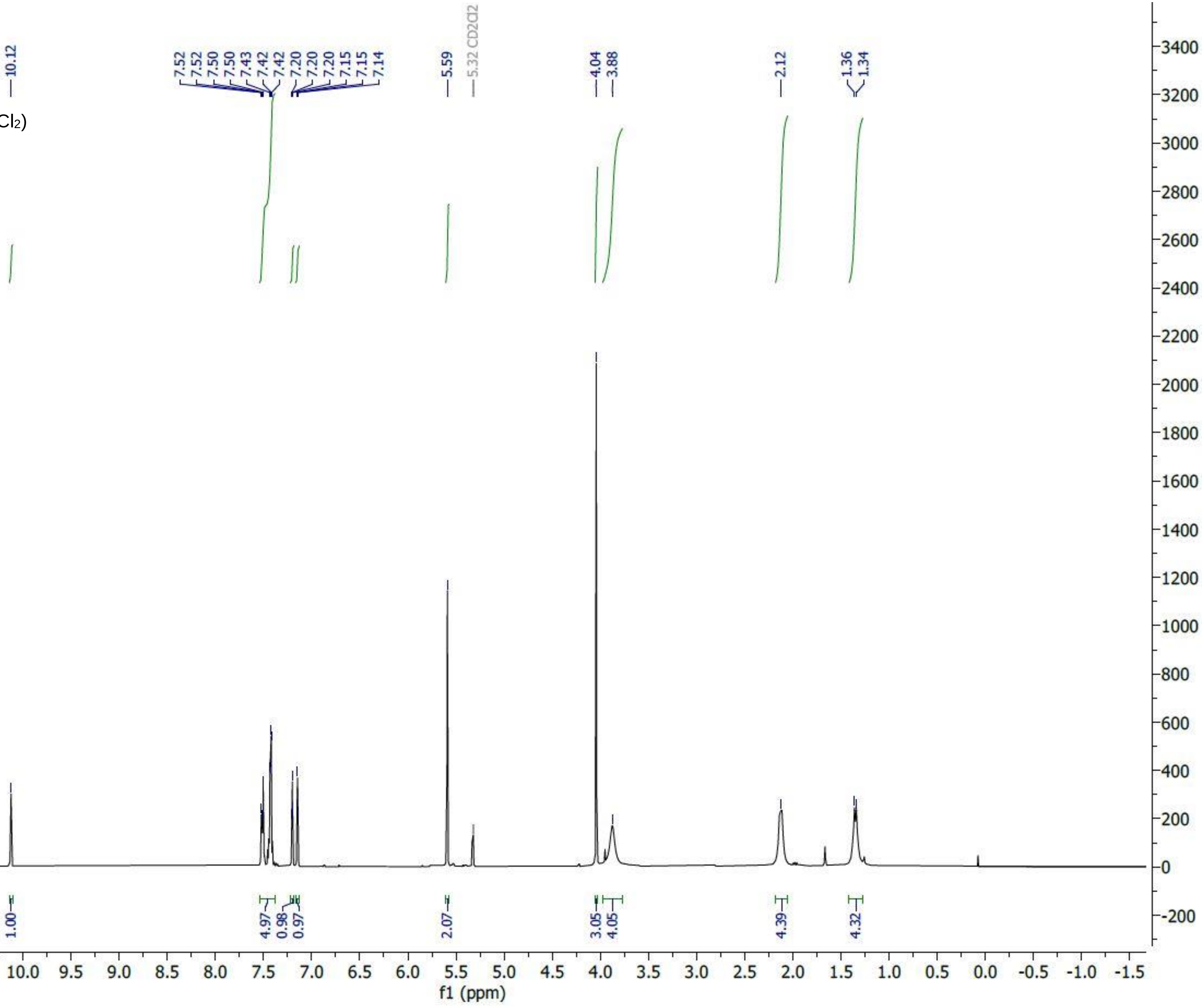

^{13}C-DEPTQ-NMR (100 MHz, CD_2Cl_2)

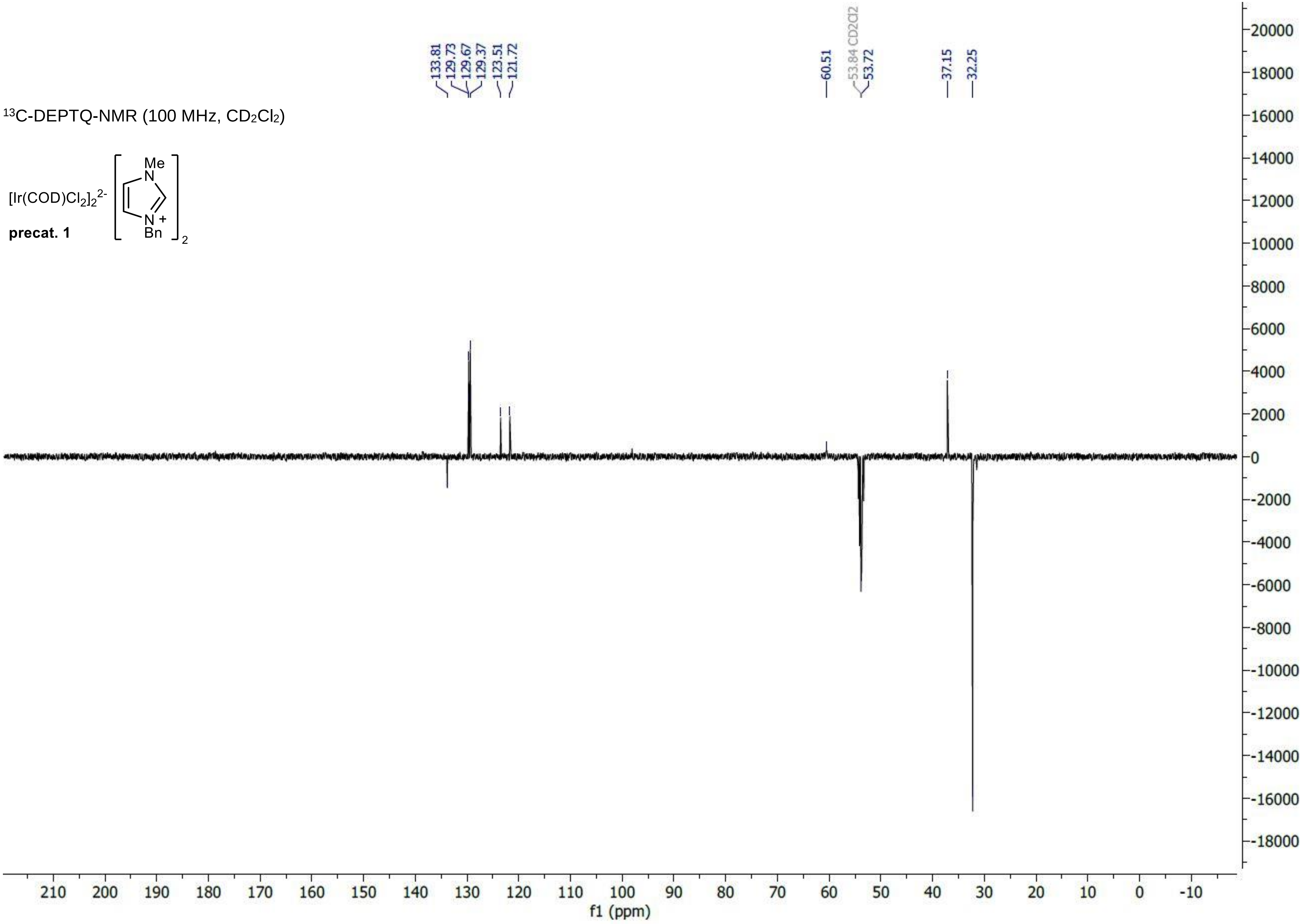

precat. 1

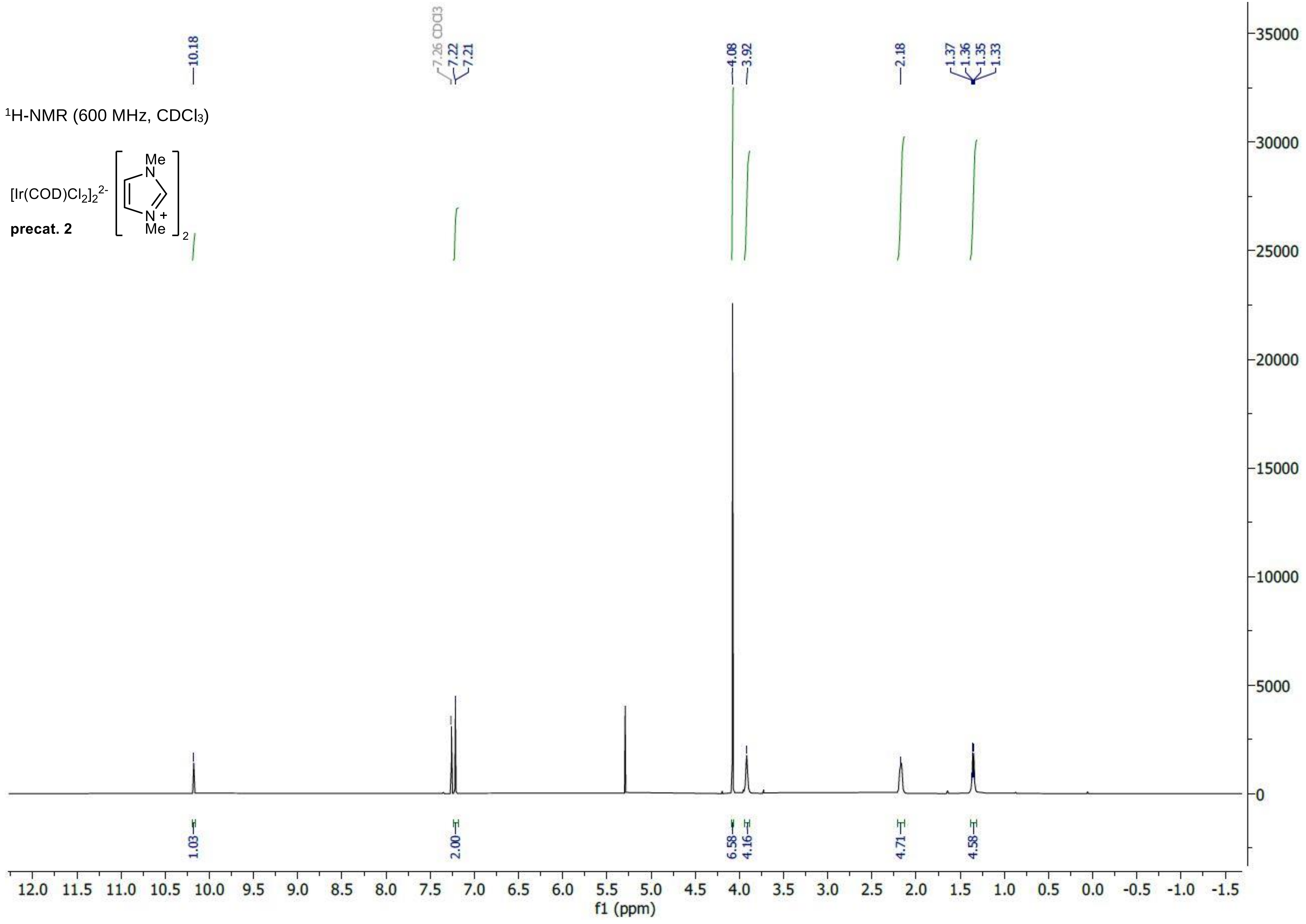

1H-NMR (600 MHz, CDCl3)
[Ir(COD)Cl2]2 2-
precat. 2
Me
N
N+
Me
10.18
7.26 CDCl3
7.22
7.21
4.08
3.92
2.18
1.37
1.36
1.35
1.33
1.03
2.00
6.58
4.16
4.71
4.58
f1 (ppm)

^{13}C-DEPTQ-NMR (150 MHz, $CDCl_3$)

$[Ir(COD)Cl_2]_2^{2-}$

precat. 2

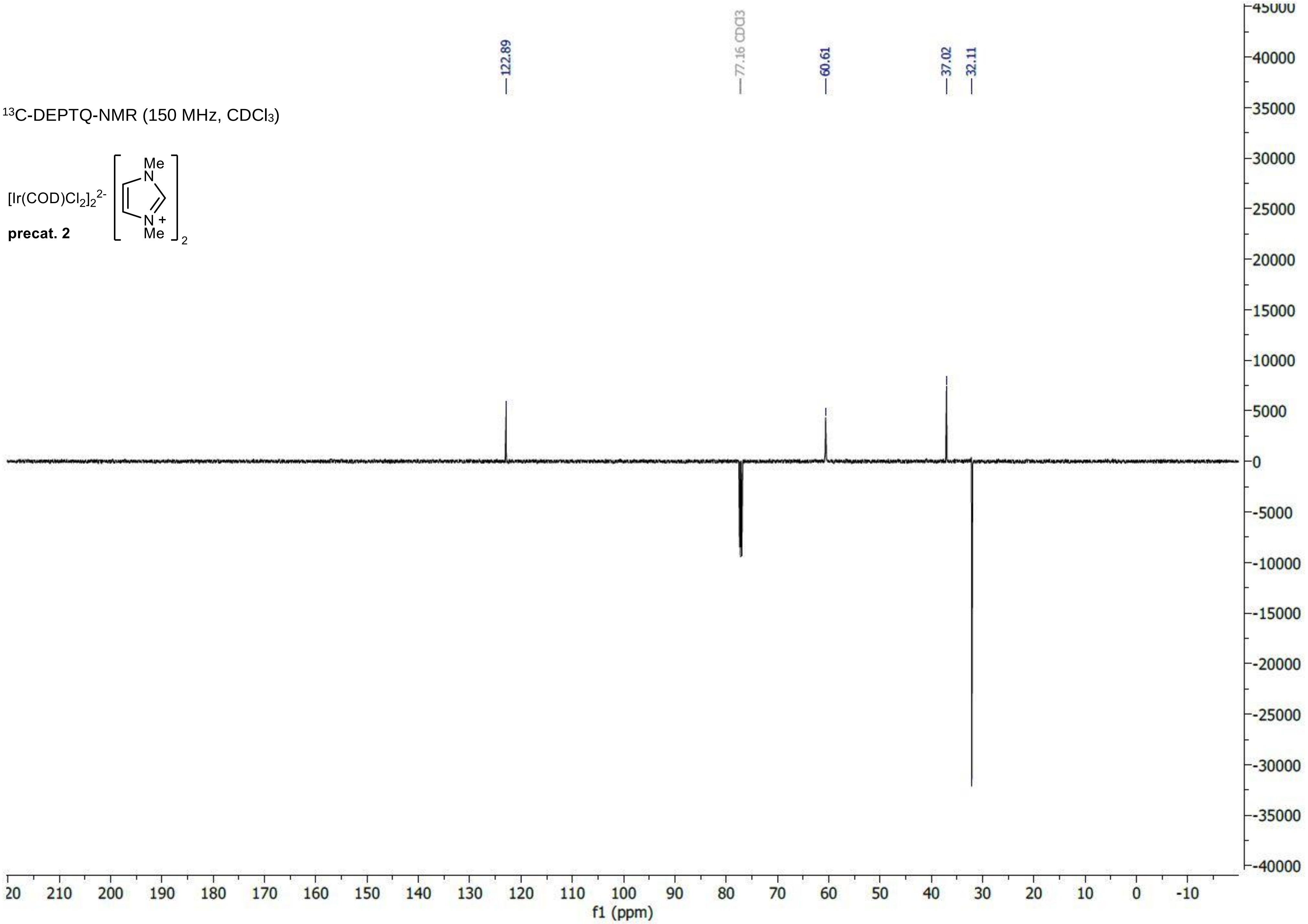

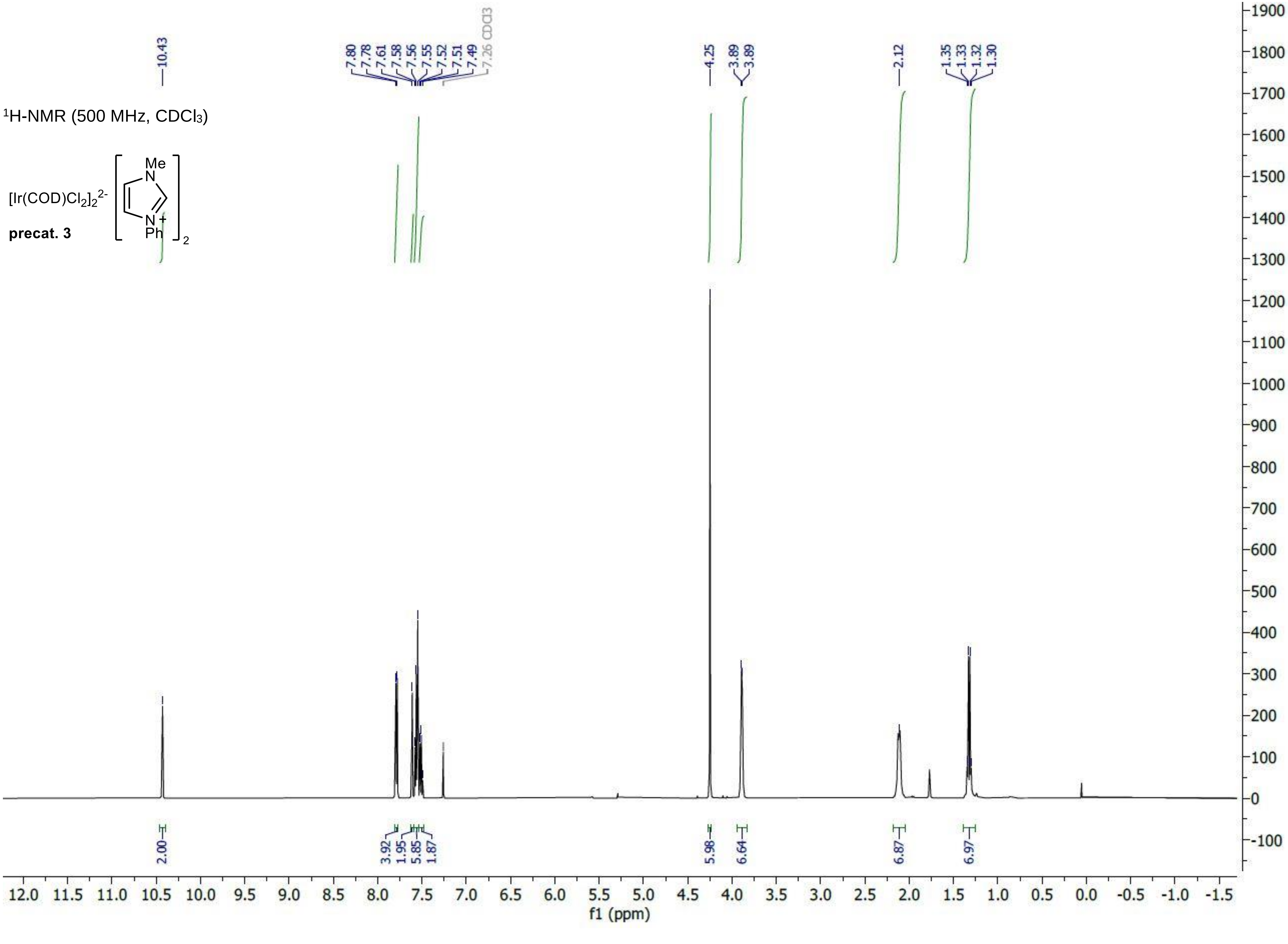

1H-NMR (500 MHz, CDCl3)
[Ir(COD)Cl2]2 2-
Me
N
N+
Ph
2
precat. 3
10.43
7.80
7.78
7.61
7.58
7.56
7.55
7.52
7.51
7.49
7.26 CDCl3
4.25
3.89
3.89
2.12
1.35
1.33
1.32
1.30
2.00
3.92
1.95
5.85
1.87
5.98
6.64
6.87
6.97
12.0 11.5 11.0 10.5 10.0 9.5 9.0 8.5 8.0 7.5 7.0 6.5 6.0 5.5 5.0 4.5 4.0 3.5 3.0 2.5 2.0 1.5 1.0 0.5 0.0 -0.5 -1.0 -1.5
f1 (ppm)
1900 1800 1700 1600 1500 1400 1300 1200 1100 1000 900 800 700 600 500 400 300 200 100 0 -100

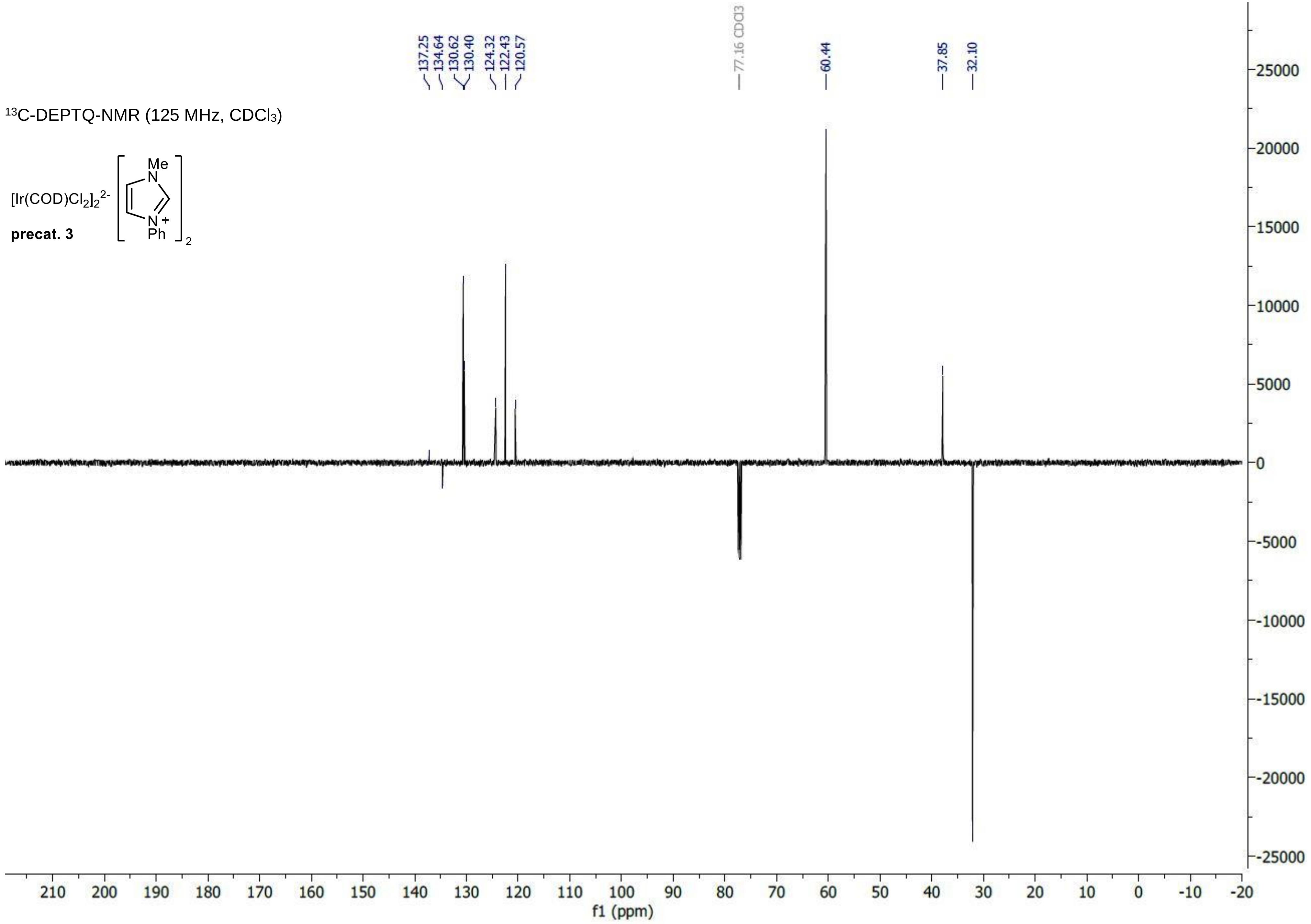

137.25
134.64
130.62
130.40
124.32
122.43
120.57
77.16 CDCl3
60.44
37.85
32.10
13C-DEPTQ-NMR (125 MHz, CDCl3)
[Ir(COD)Cl2]2 2-
Me
N
N+
Ph
2
precat. 3
25000
20000
15000
10000
5000
0
-5000
-10000
-15000
-20000
-25000
210 200 190 180 170 160 150 140 130 120 110 100 90 80 70 60 50 40 30 20 10 0 -10 -20
f1 (ppm)

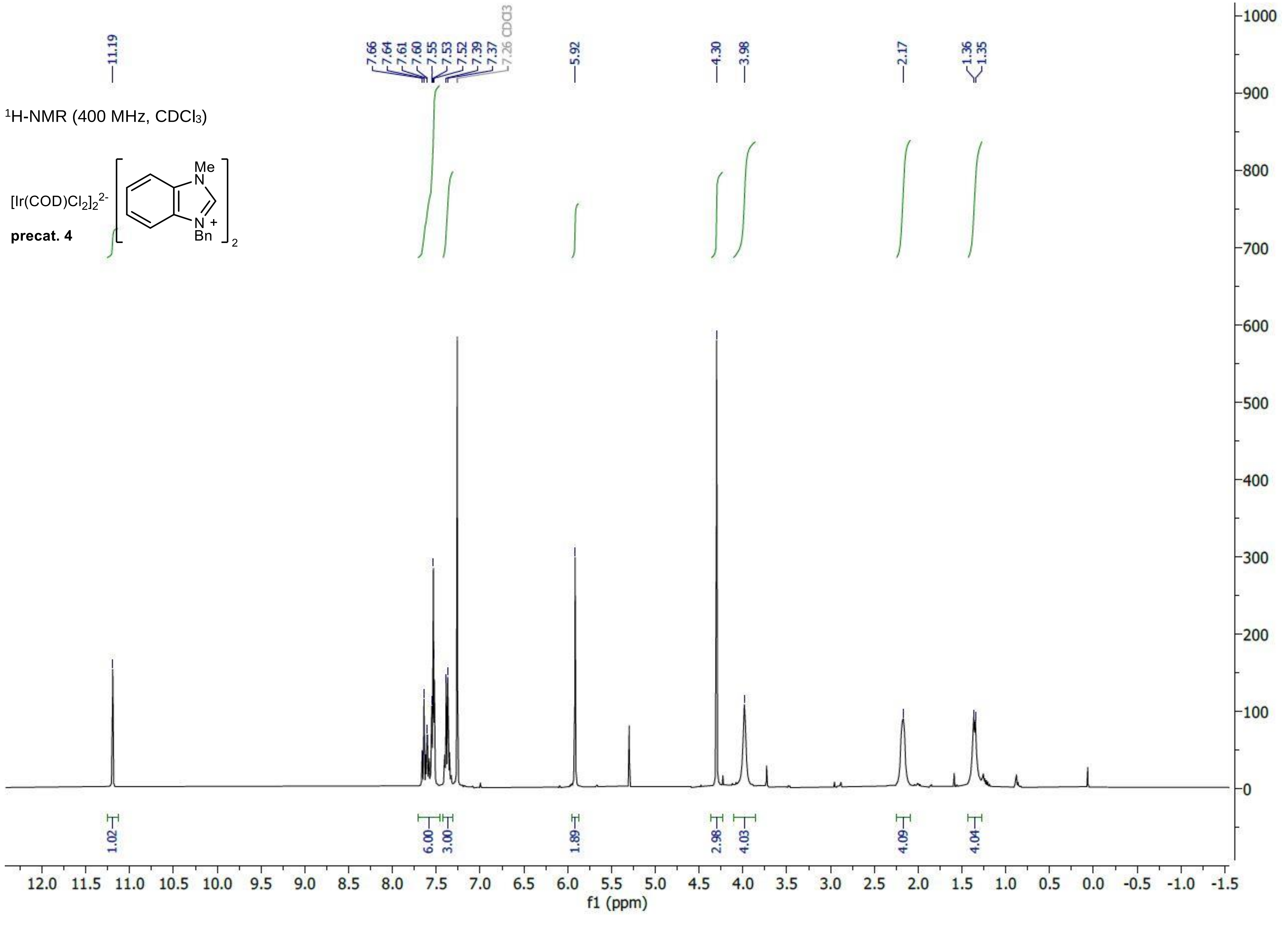

1H-NMR (400 MHz, CDCl3)
[Ir(COD)Cl2]2 2-
Me
N
N+
Bn
2
precat. 4
11.19
7.66
7.64
7.61
7.60
7.55
7.53
7.52
7.39
7.37
7.26 CDCl3
5.92
4.30
3.98
2.17
1.36
1.35
1.02
6.00
3.00
1.89
2.98
4.03
4.09
4.04
f1 (ppm)

^{13}C-DEPTQ-NMR (100 MHz, $CDCl_3$)

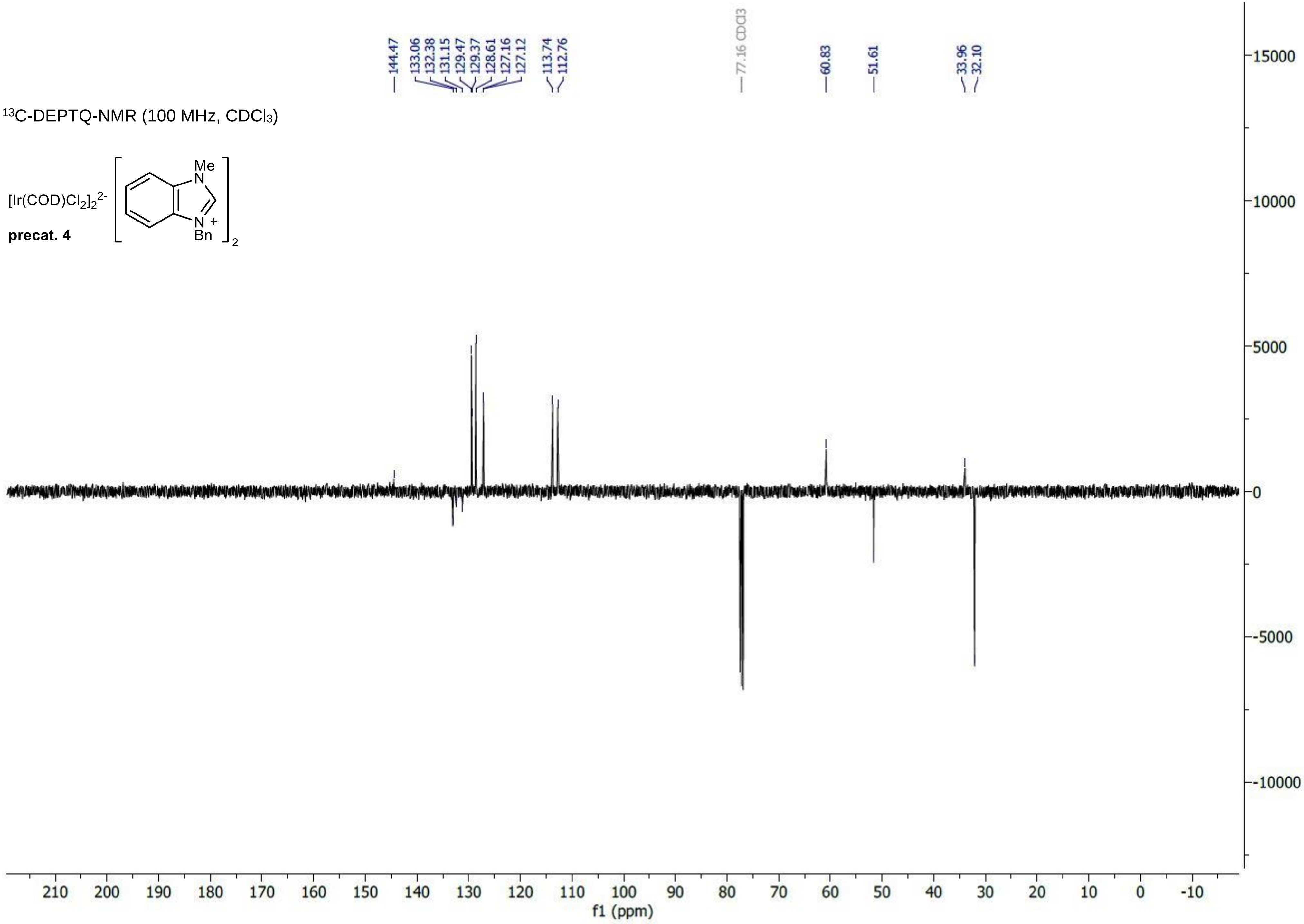

precat. 4

12.2. complex K-2a

^{1}H-NMR (400 MHz, $CDCl_3$)

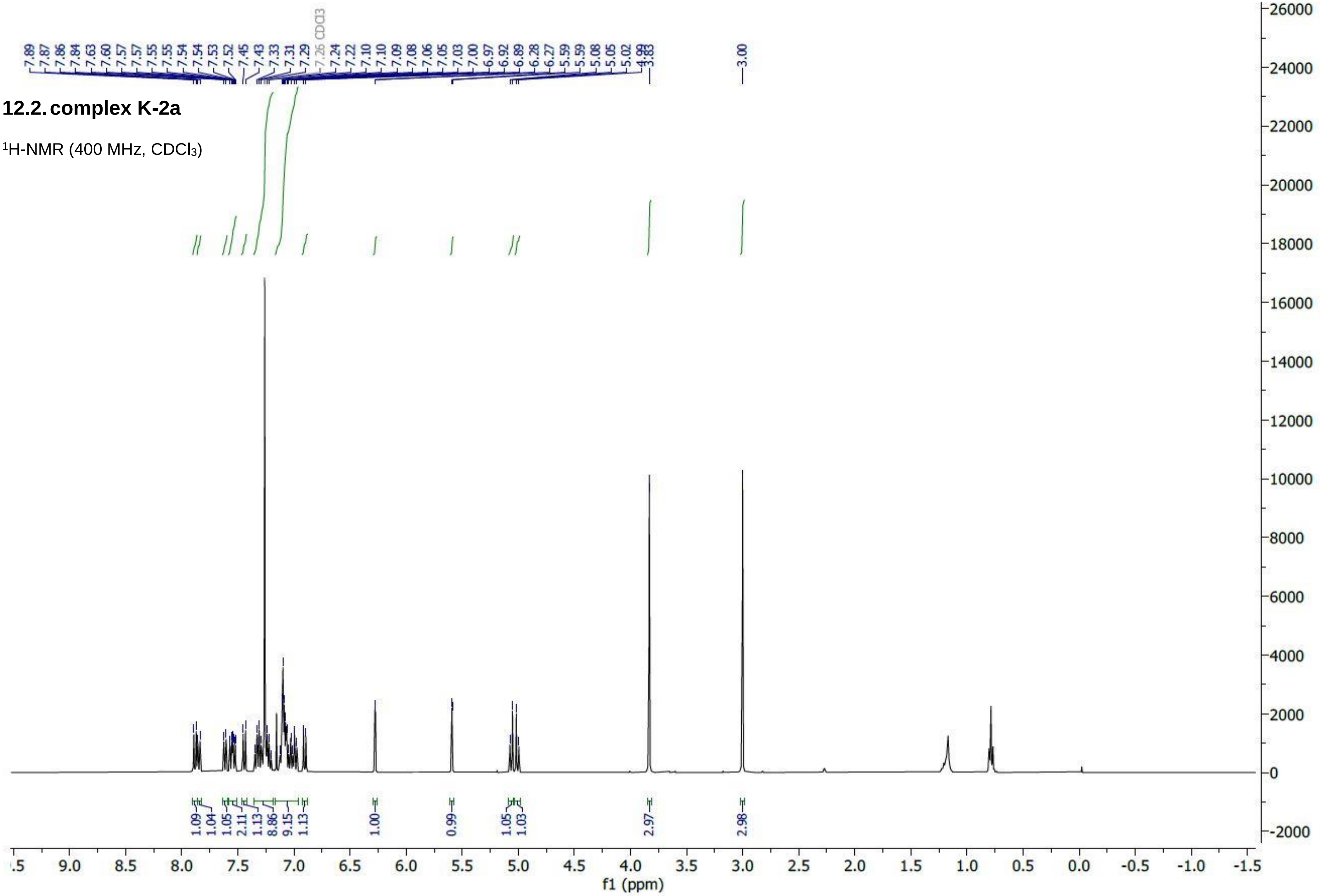

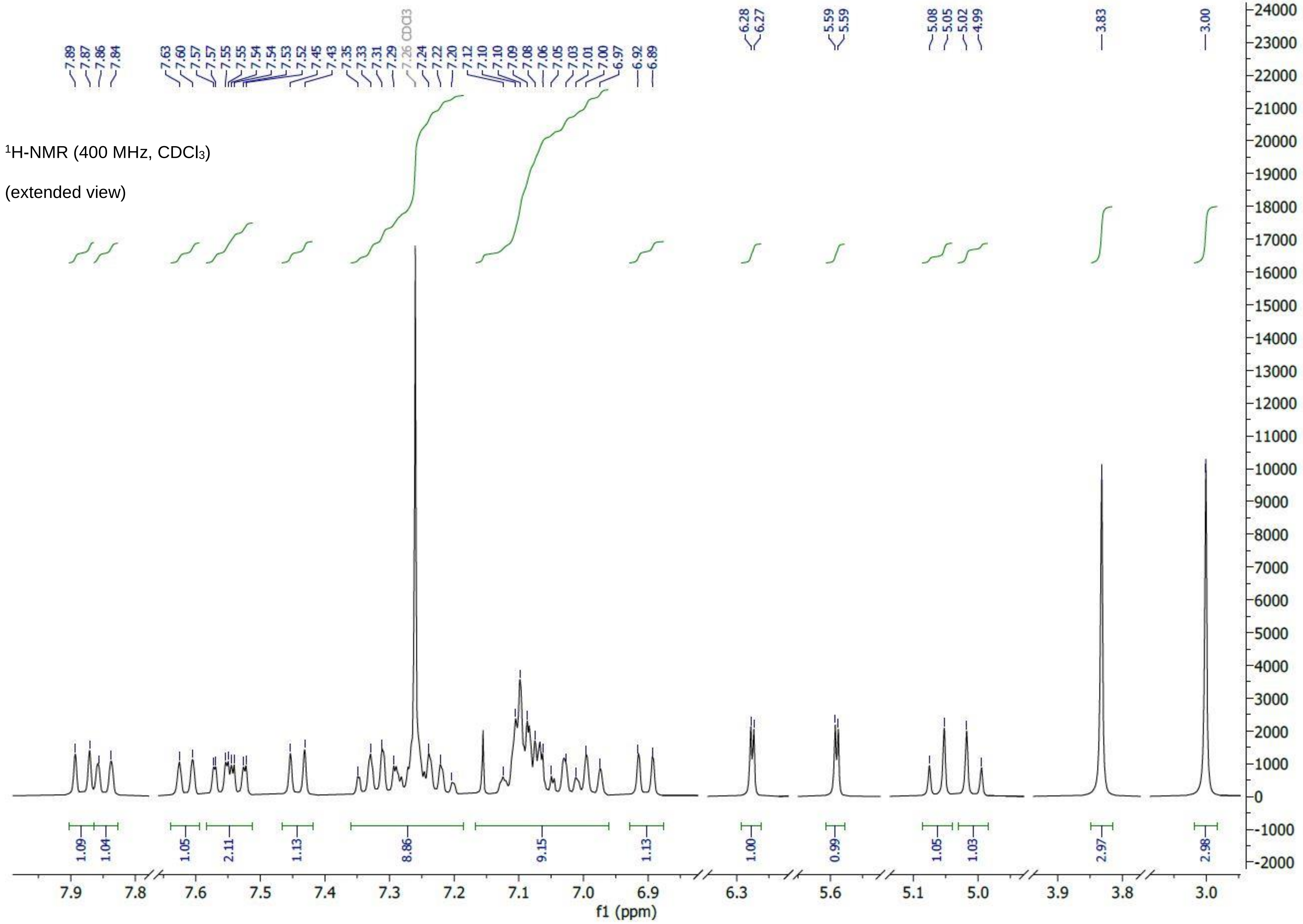

1H-NMR (400 MHz, CDCl3)
(extended view)
f1 (ppm)

^{13}C-DEPTQ-NMR (100 MHz, $CDCl_3$)

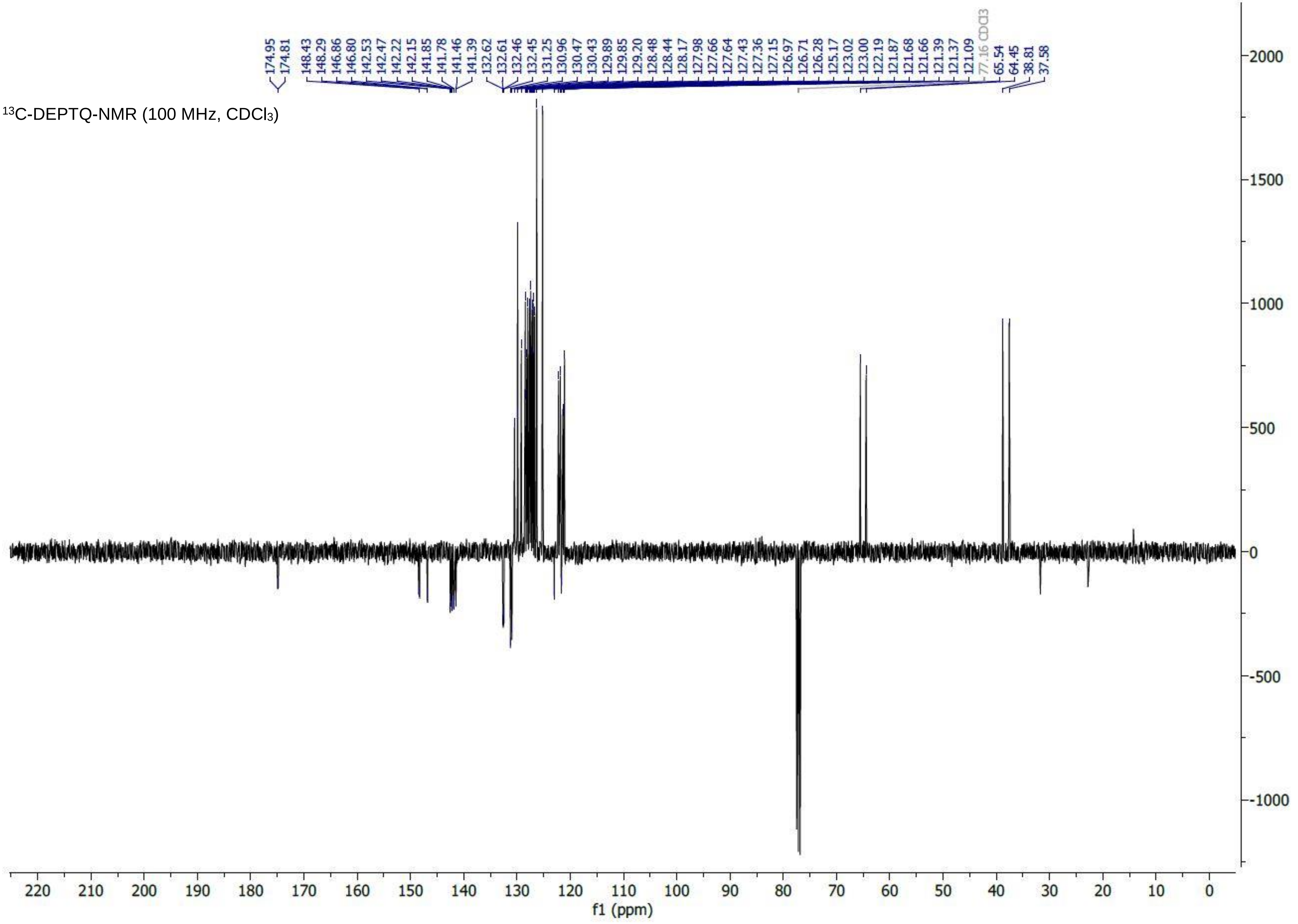

^{13}C-DEPTQ-NMR (100 MHz, $CDCl_3$)

(extended view)

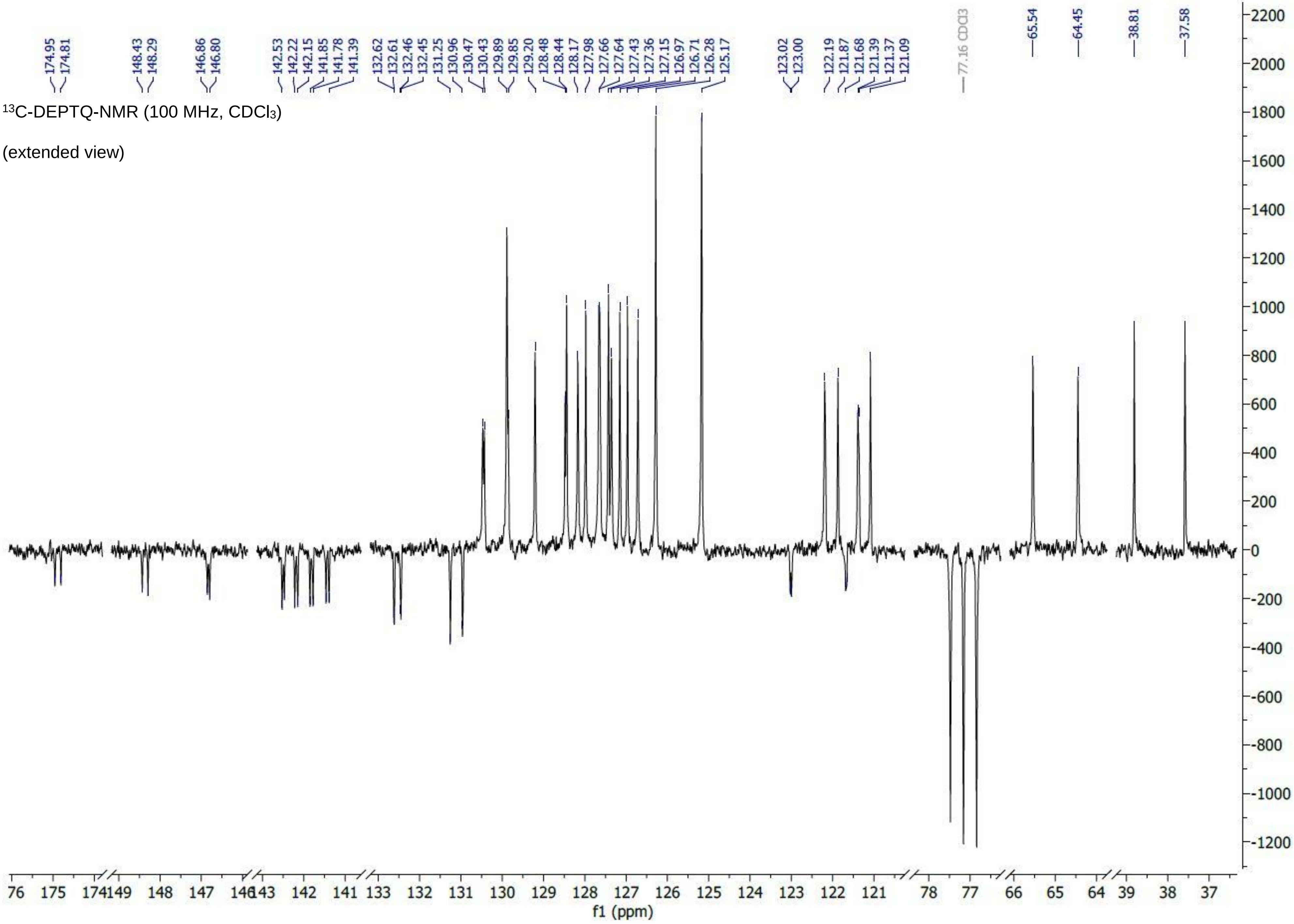

^{13}C-DEPTQ-NMR (100 MHz, $CDCl_3$)

(extended view, section of overlapping signals)

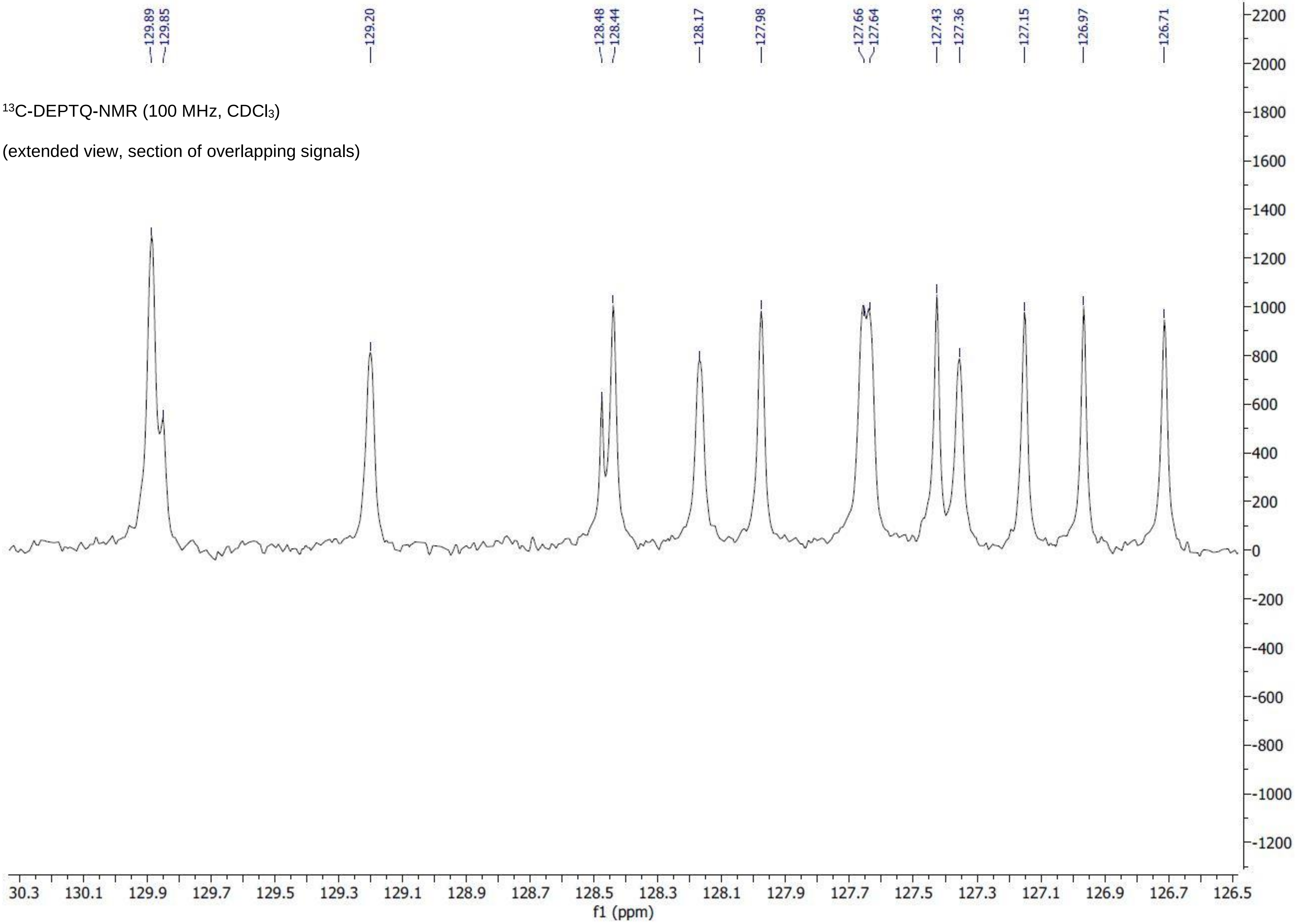

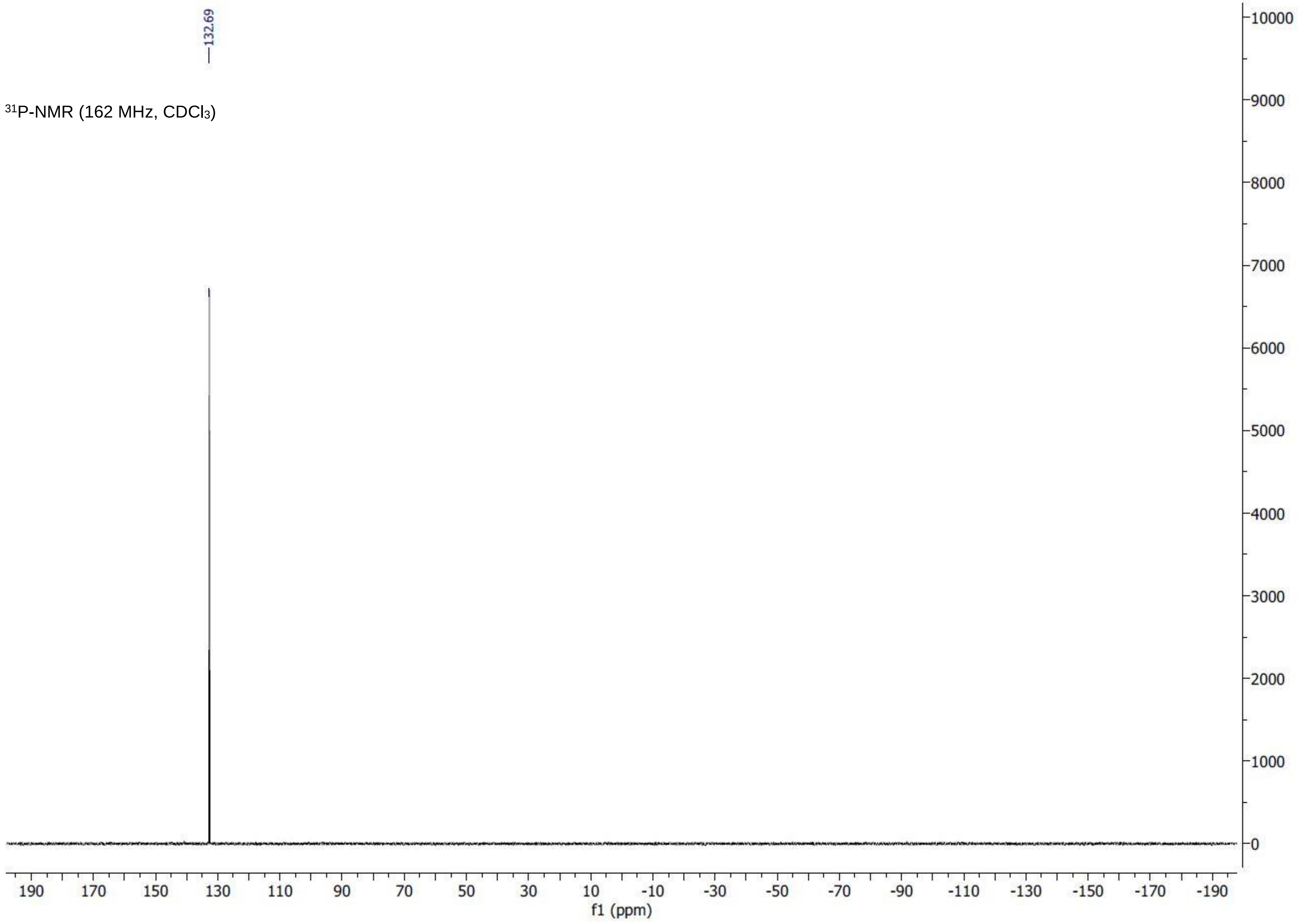

132.69
31P-NMR (162 MHz, CDCl3)
f1 (ppm)

12.3. complex K-2b

^{1}H-NMR (600 MHz, CD_2Cl_2)

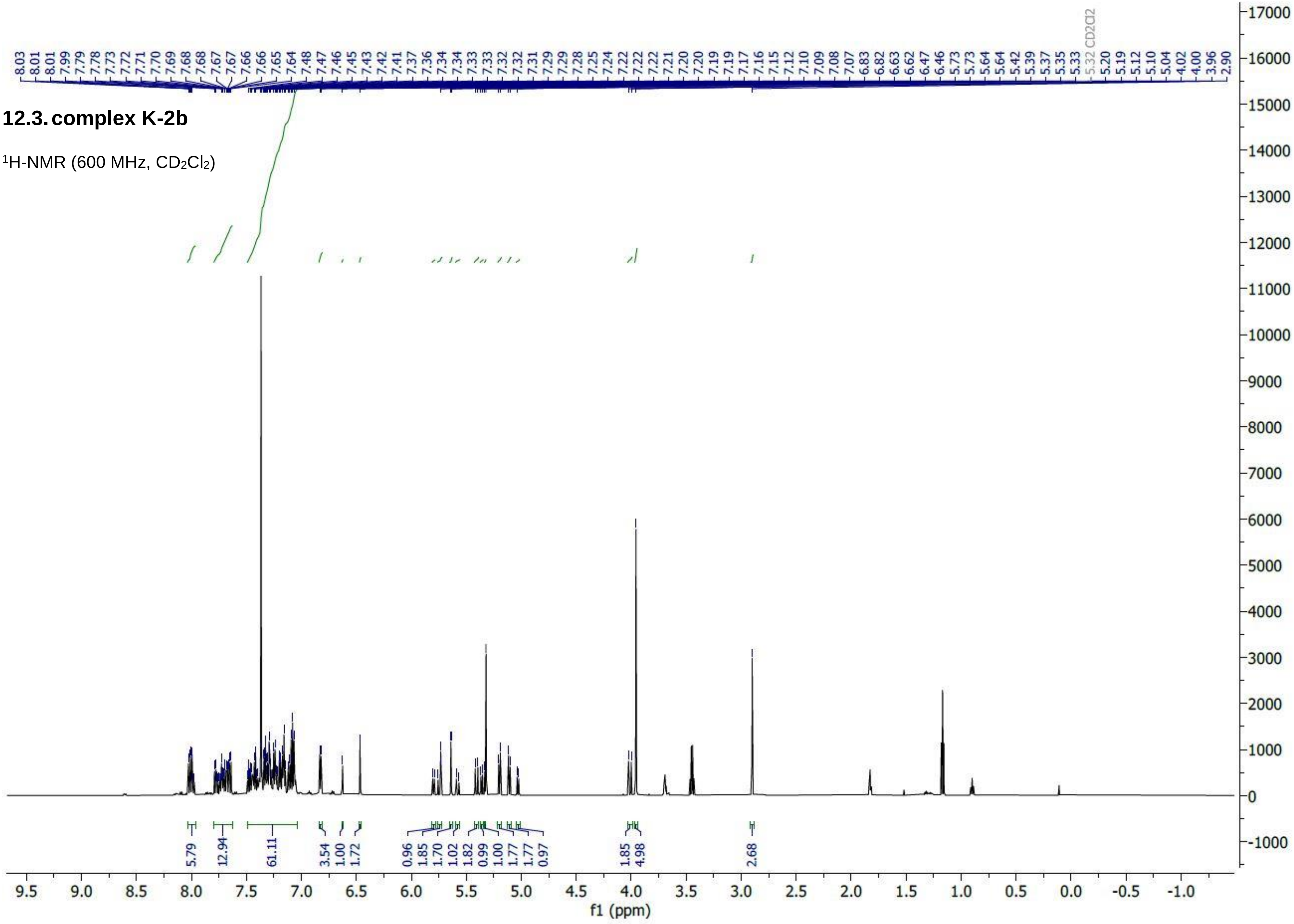

^1H-NMR (600 MHz, CD_2Cl_2)

(extended view)

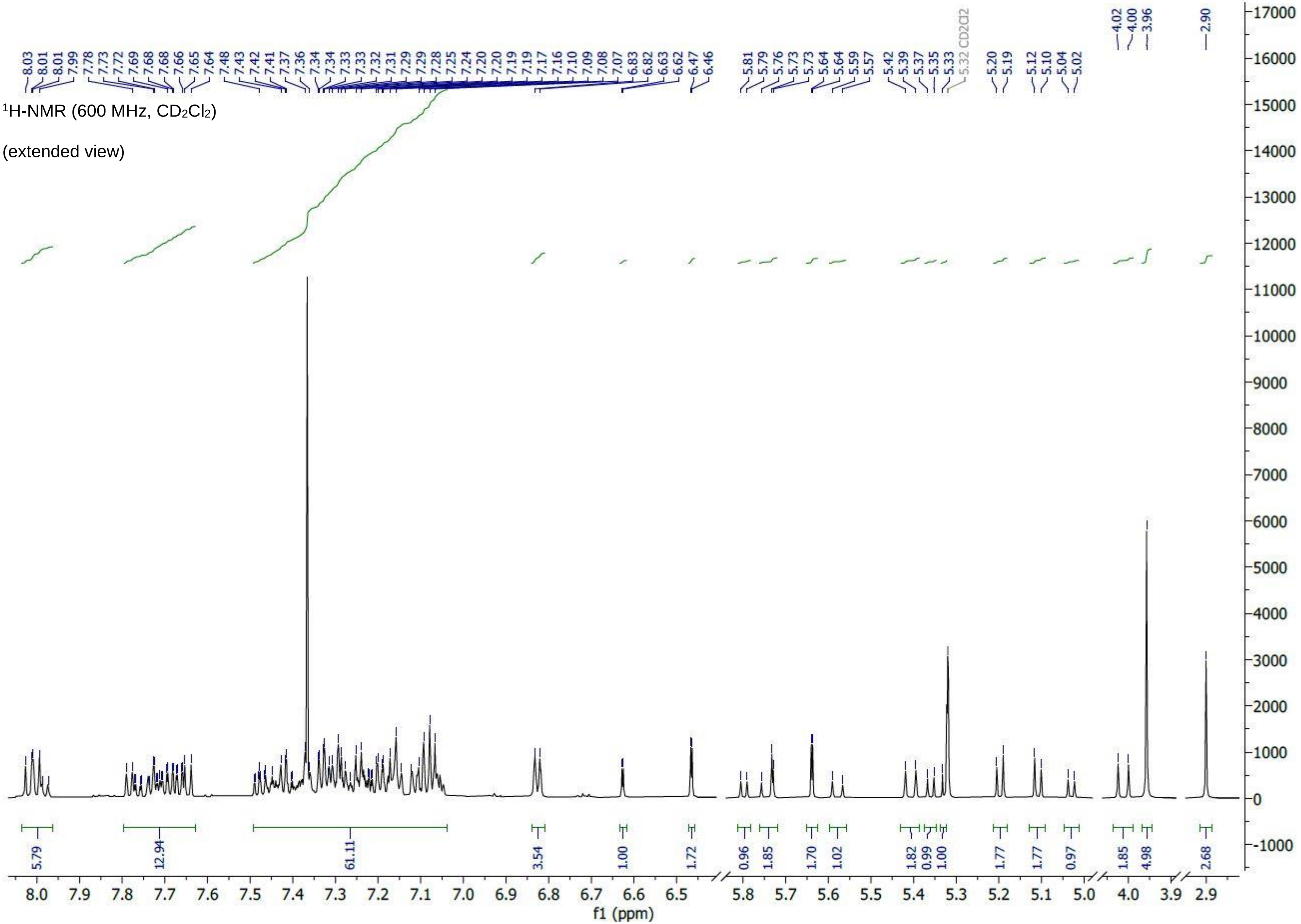

^{13}C-DEPTQ-NMR (150 MHz, CD_2Cl_2)

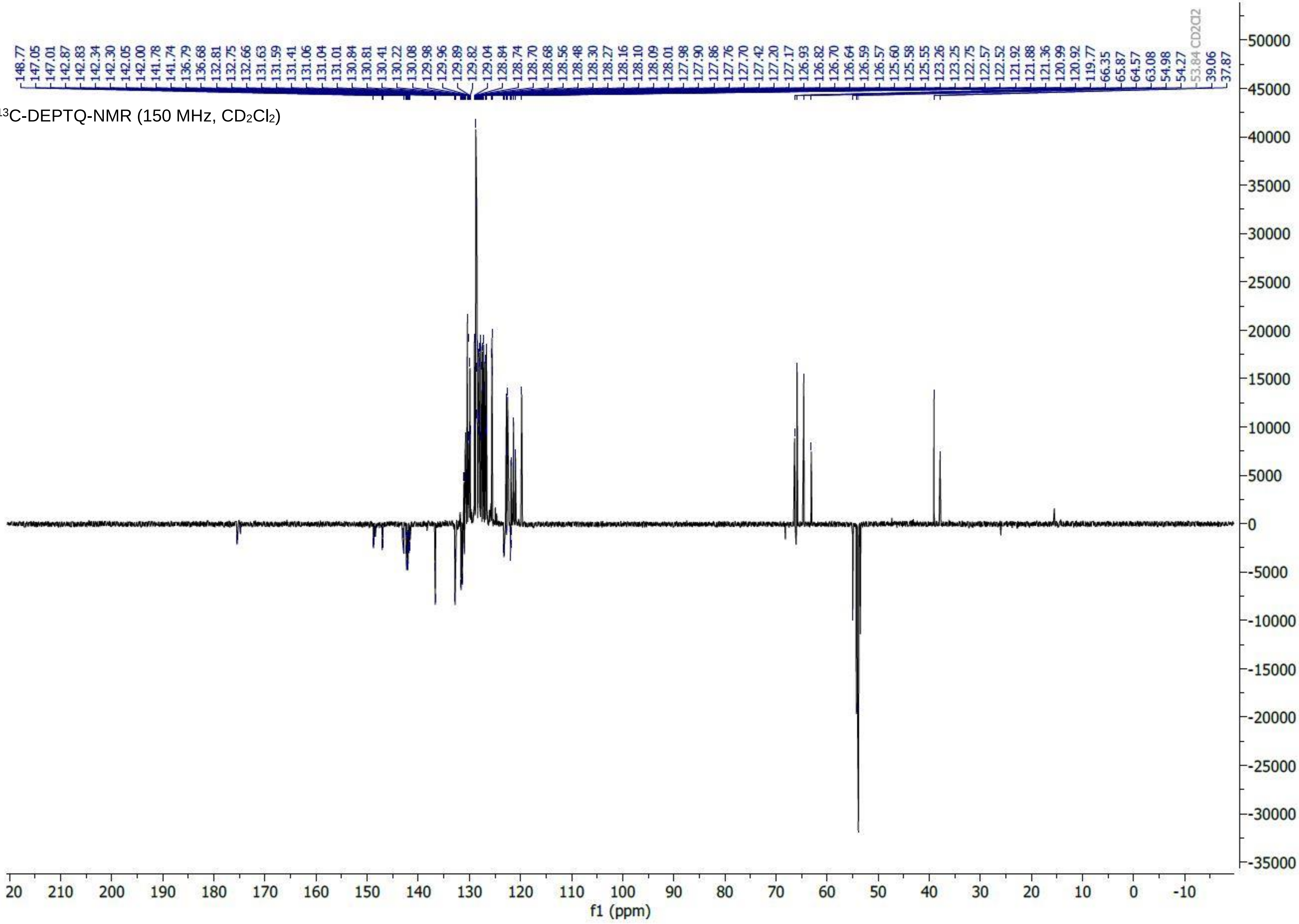

^{13}C-DEPTQ-NMR (150 MHz, CD_2Cl_2)

(extended view, section 1)

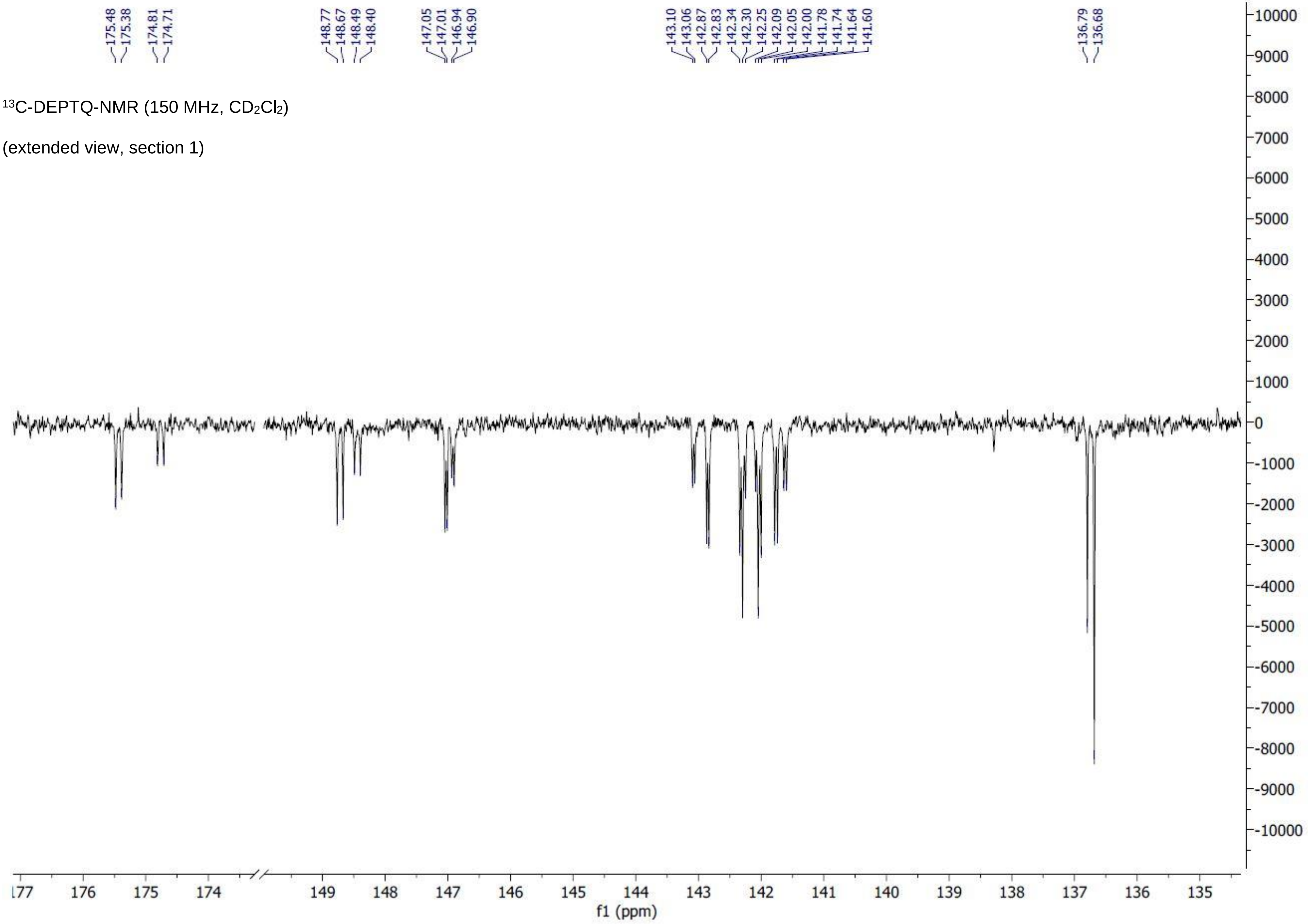

^{13}C-DEPTQ-NMR (150 MHz, CD_2Cl_2)

(extended view, section 2)

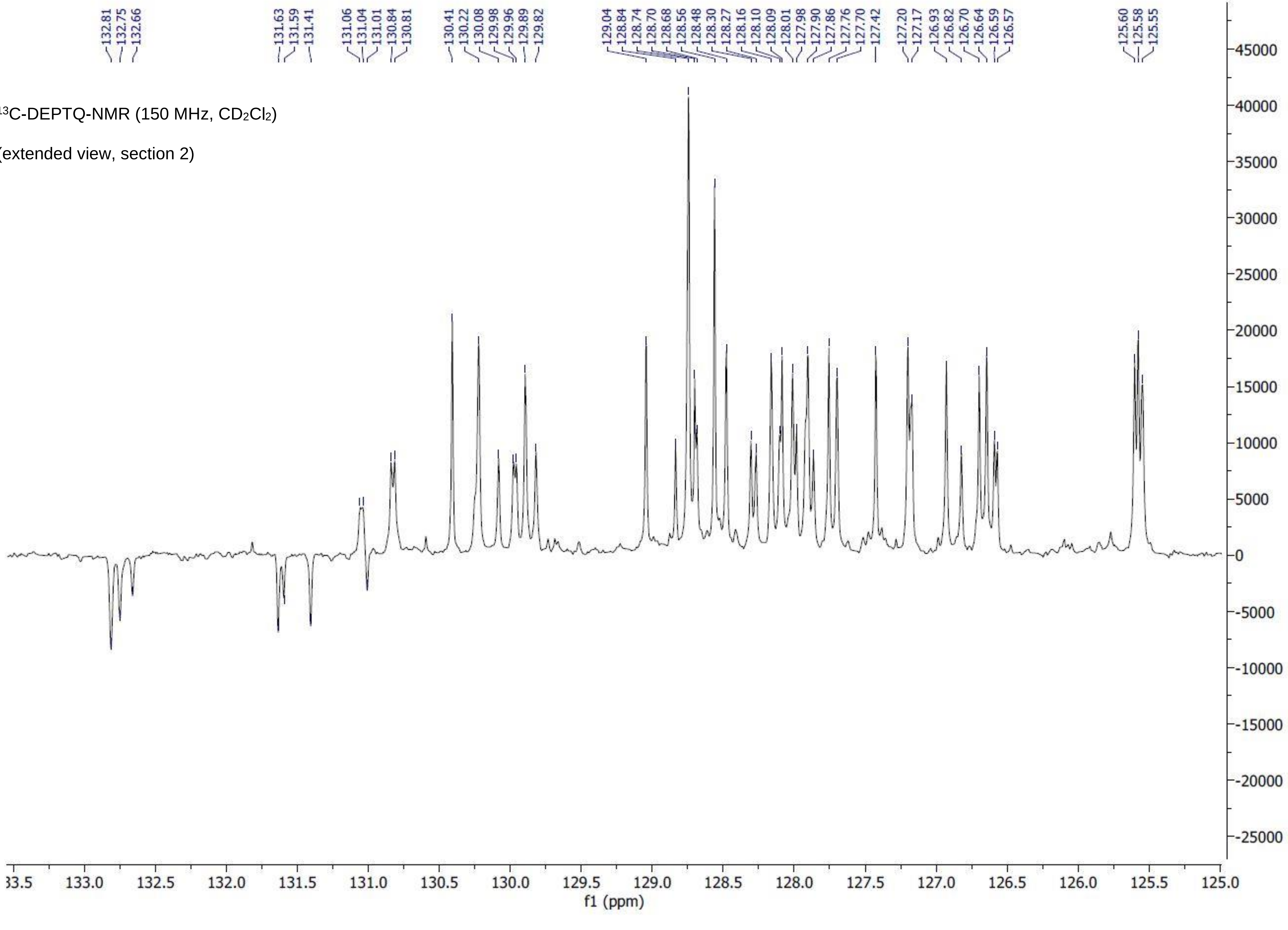

^{13}C-DEPTQ-NMR (150 MHz, CD_2Cl_2)

(extended view, section 3)

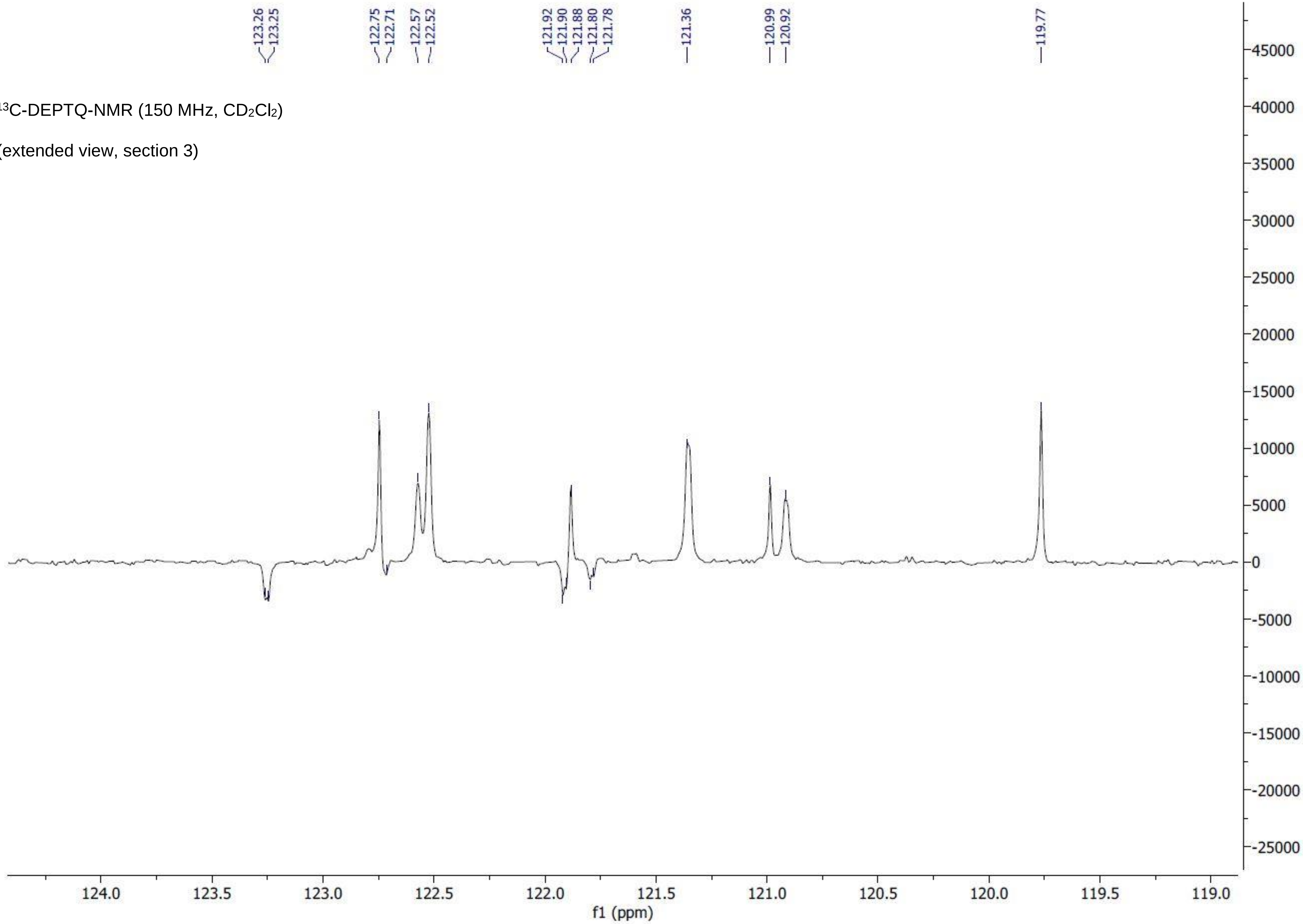

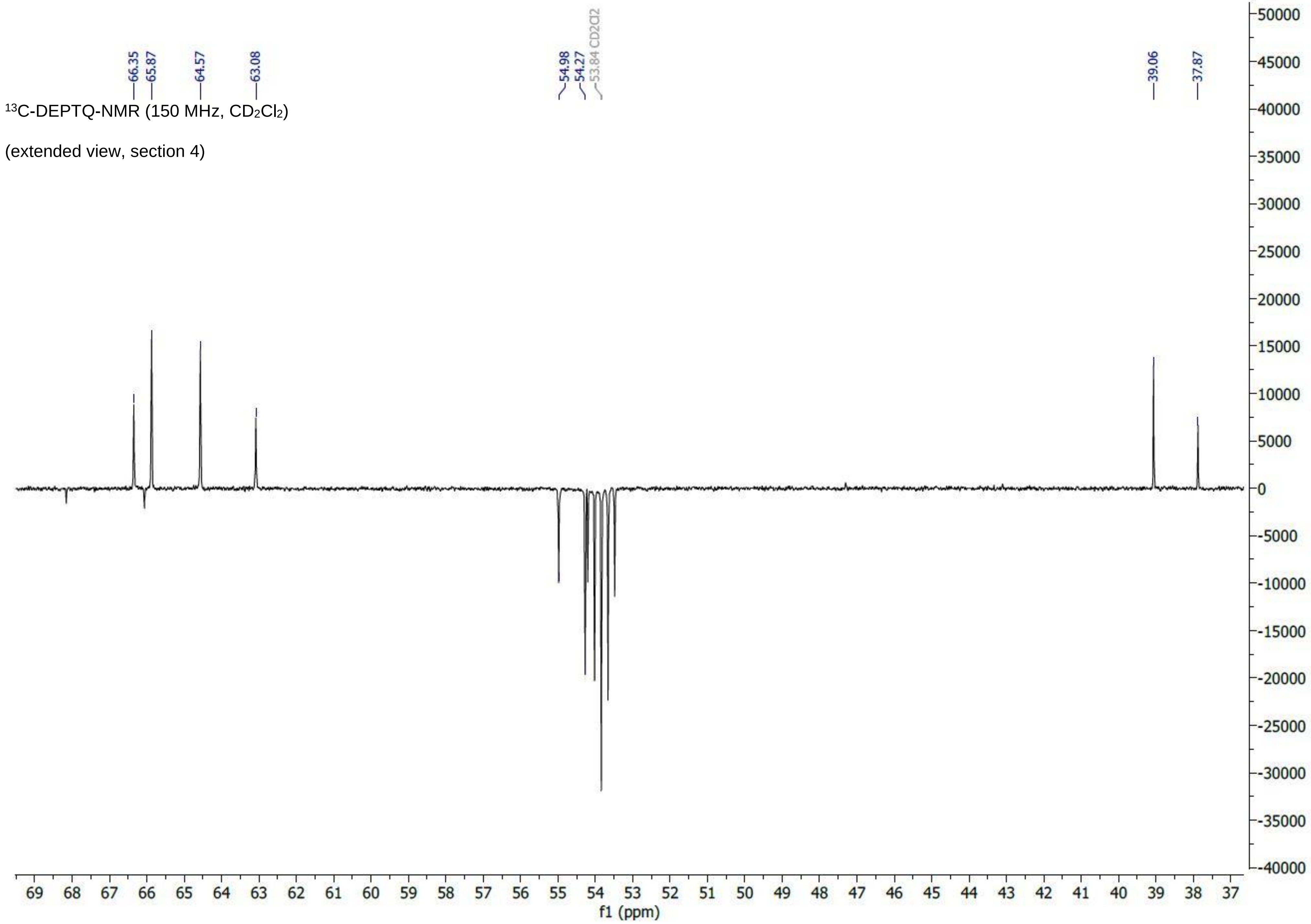

13C-DEPTQ-NMR (150 MHz, CD2Cl2)
(extended view, section 4)
66.35
65.87
64.57
63.08
54.98
54.27
53.84 CD2Cl2
39.06
37.87
f1 (ppm)

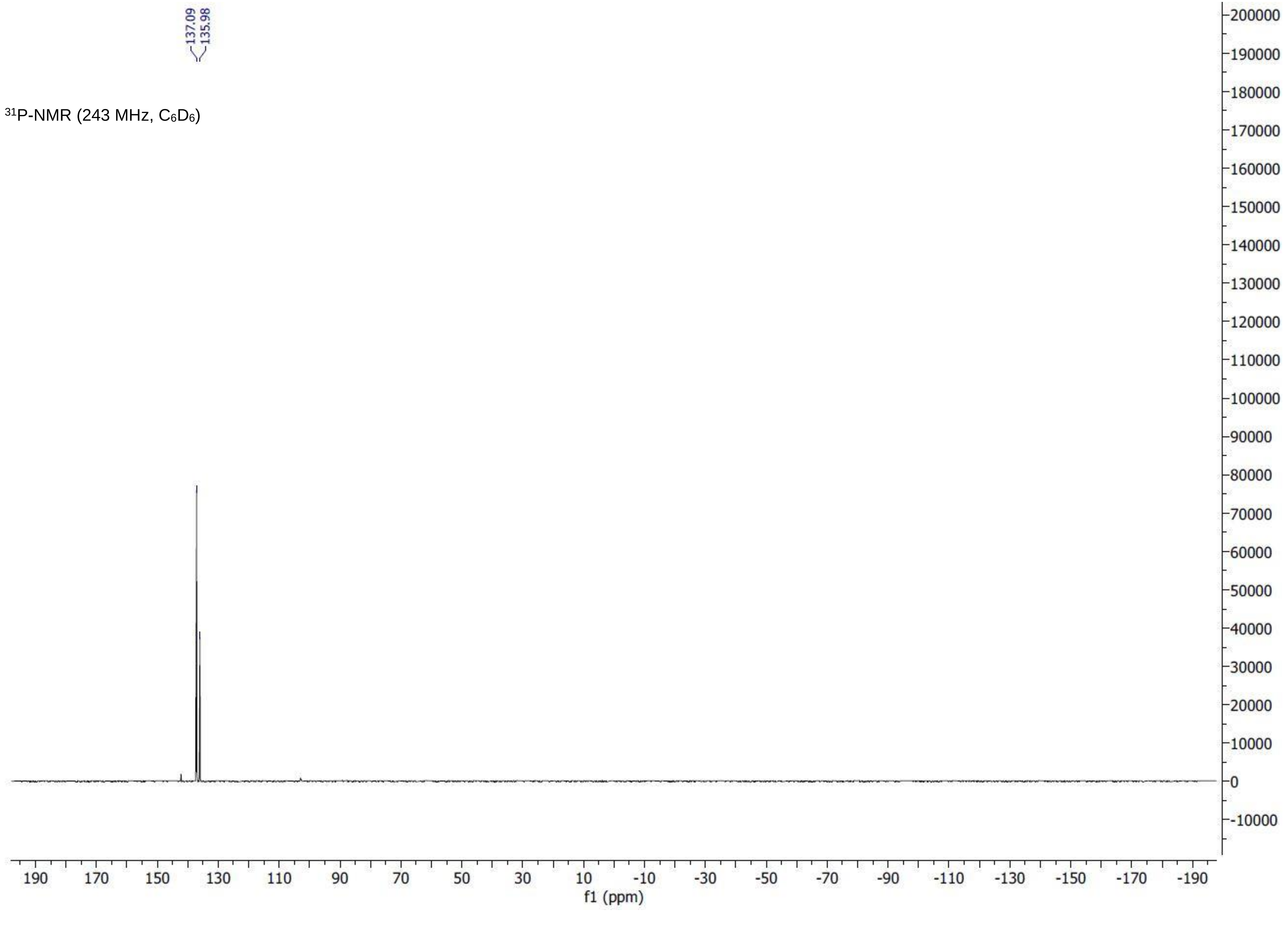
137.09
135.98
31P-NMR (243 MHz, C6D6)
f1 (ppm)

12.4. complex K-3a

^{1}H-NMR (400 MHz, $CDCl_3$, 300 K)

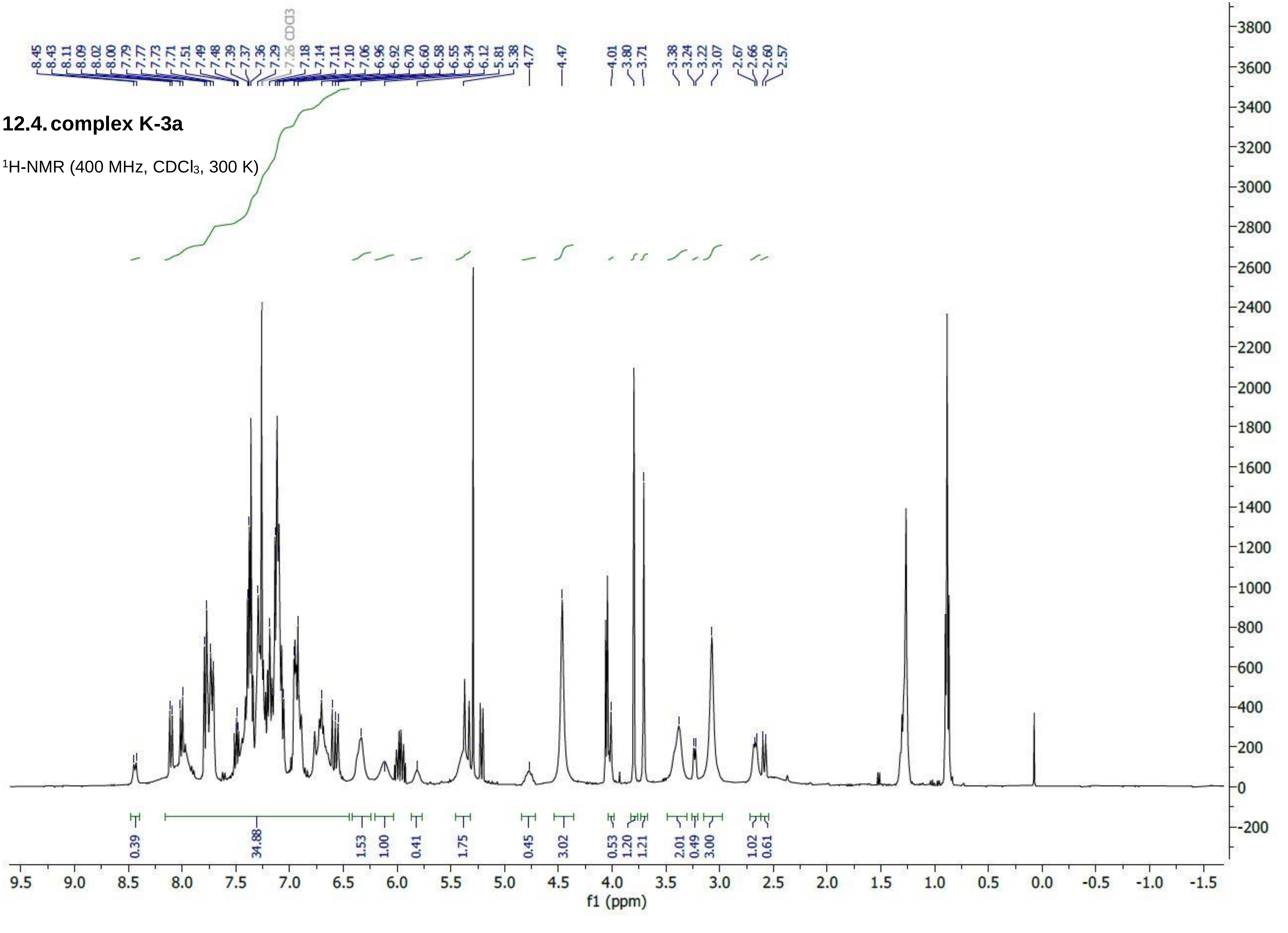

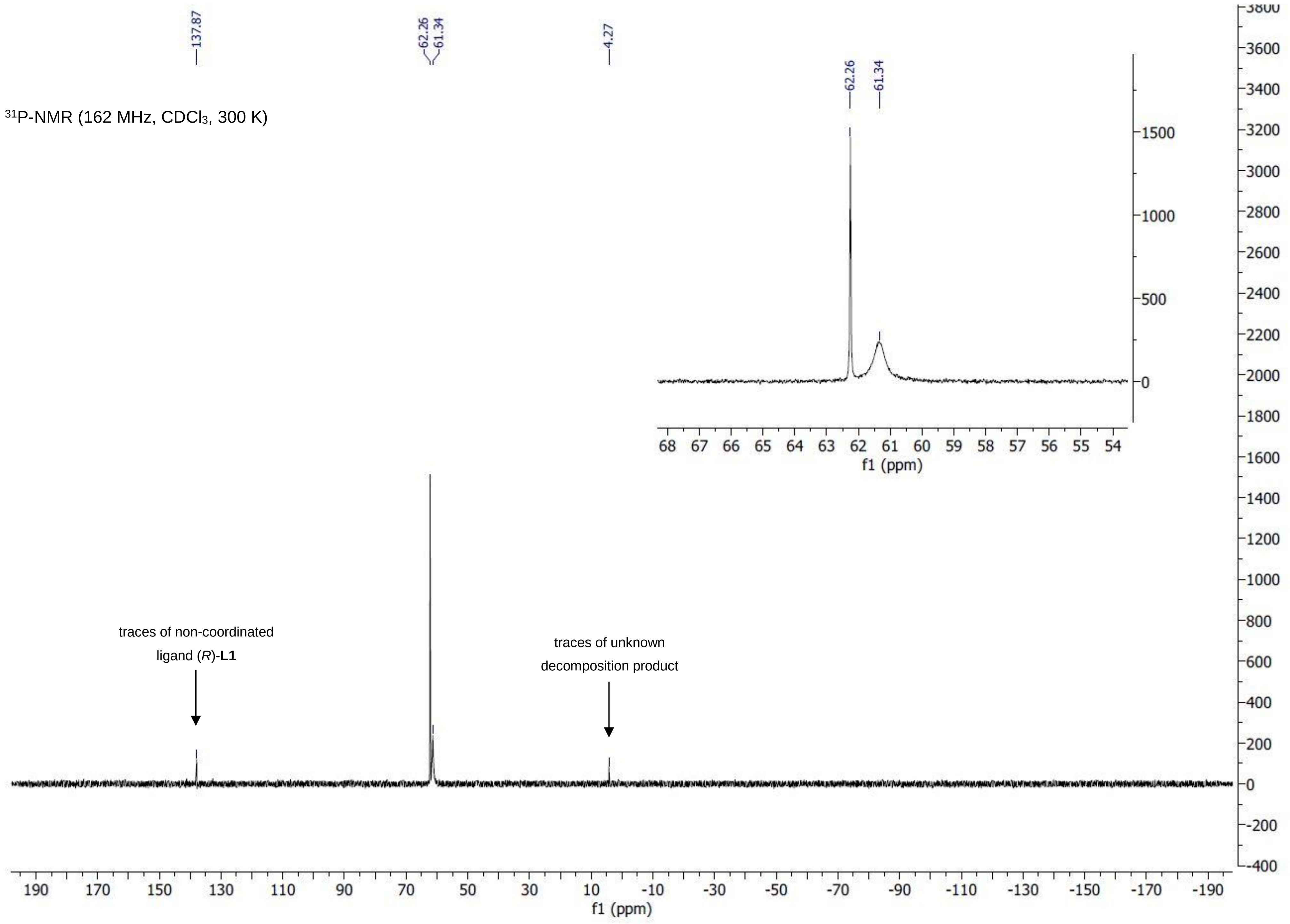
31P-NMR (162 MHz, CDCl3, 300 K)
137.87
62.26
61.34
4.27
traces of non-coordinated ligand (R)-L1
traces of unknown decomposition product
f1 (ppm)

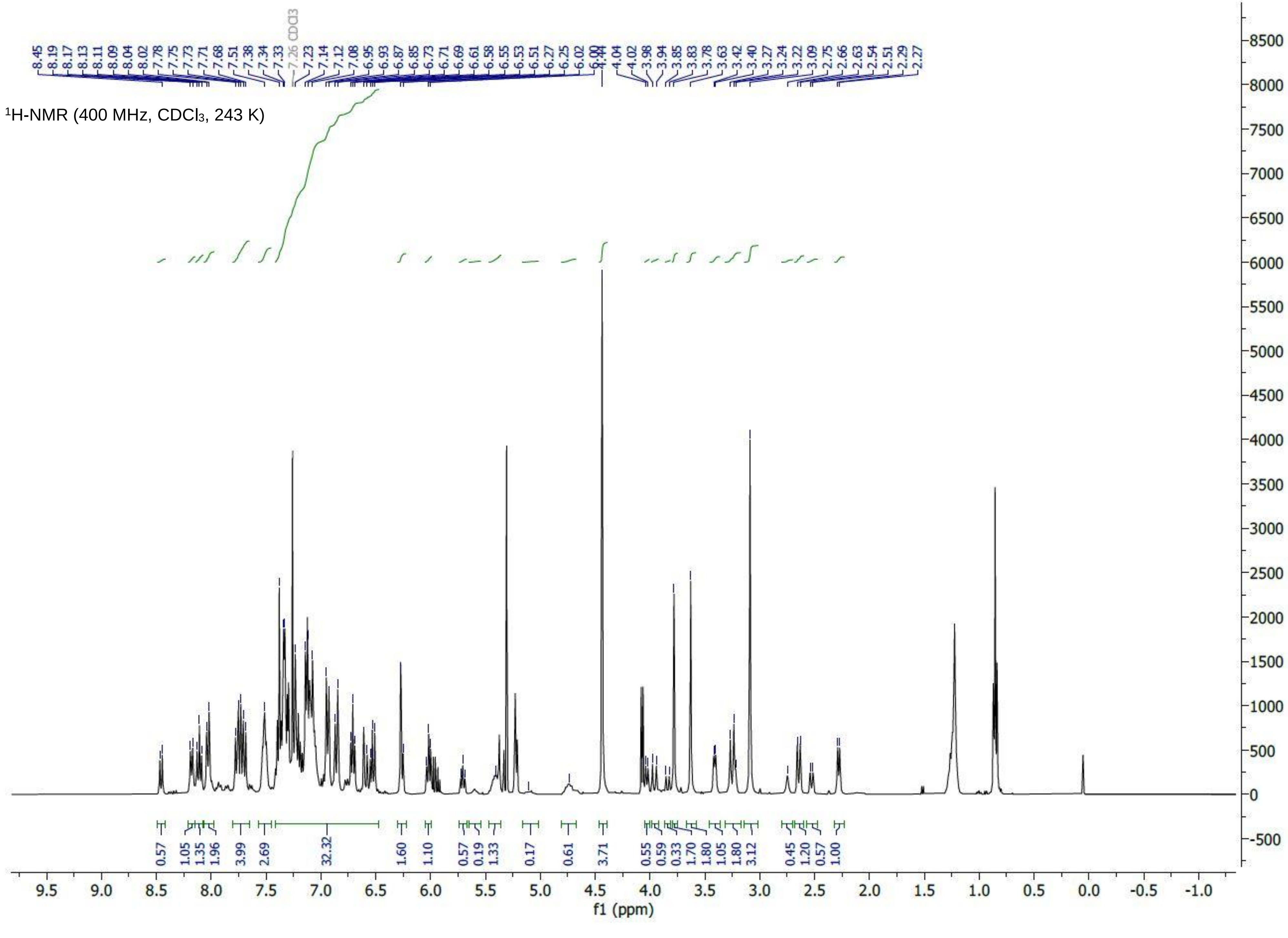

^{1}H-NMR (400 MHz, CDCl$_3$, 243 K)
f1 (ppm)

^{1}H-NMR (400 MHz, $CDCl_3$, 243 K)

(extended view, section 1)

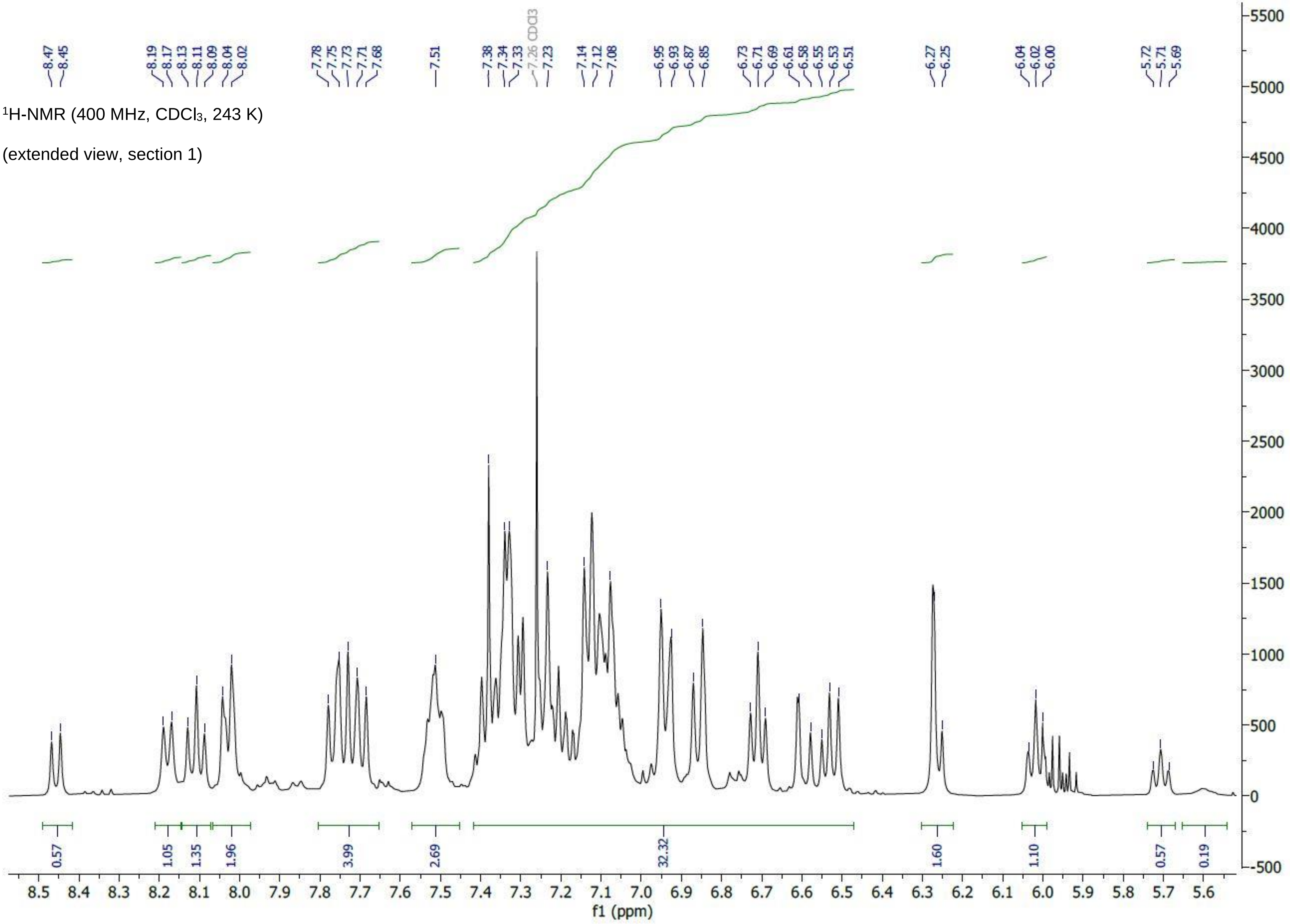

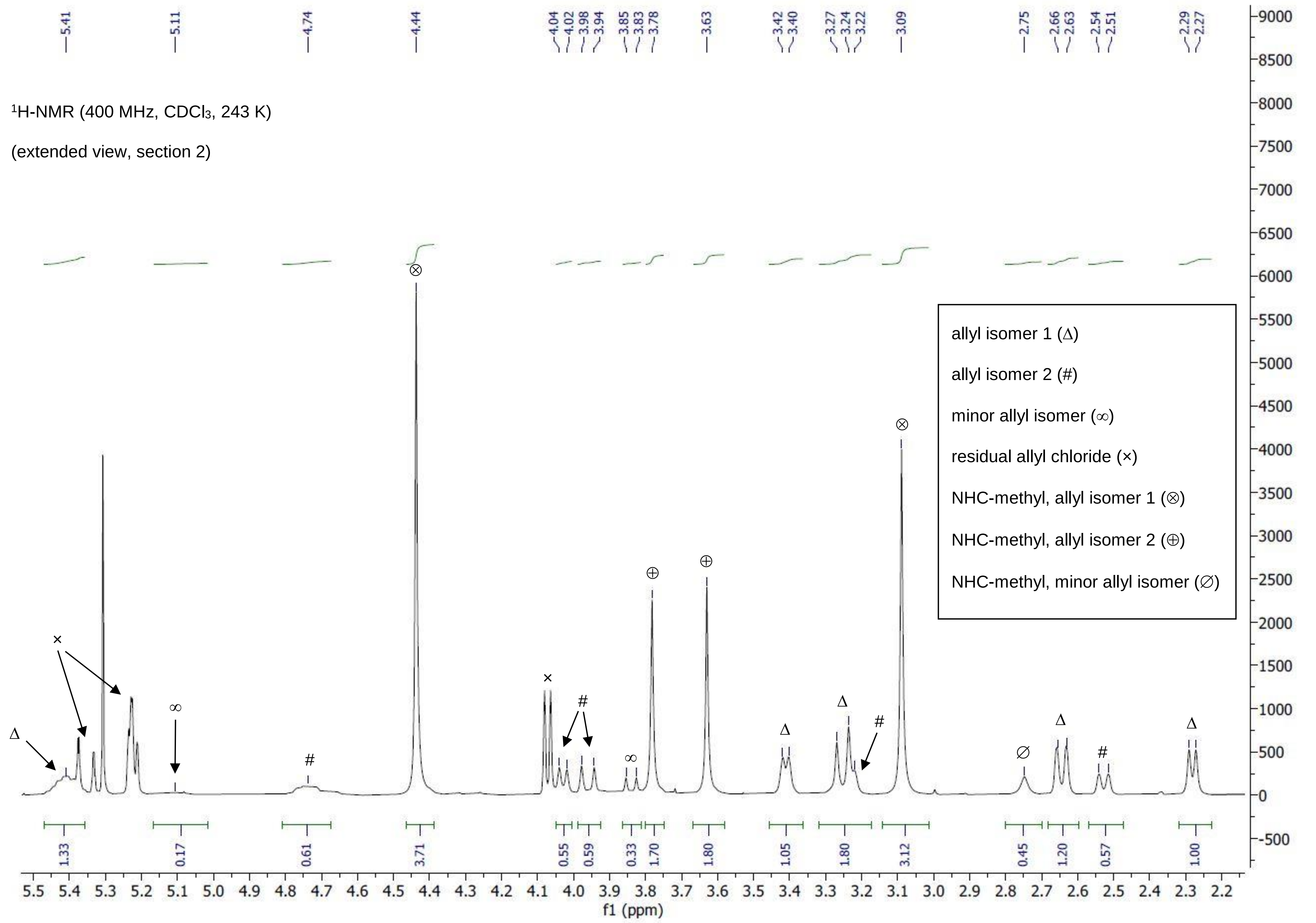

1H-NMR (400 MHz, CDCl3, 243 K)
(extended view, section 2)
allyl isomer 1 (Δ)
allyl isomer 2 (#)
minor allyl isomer (∞)
residual allyl chloride (×)
NHC-methyl, allyl isomer 1 (⊗)
NHC-methyl, allyl isomer 2 (⊕)
NHC-methyl, minor allyl isomer (∅)
5.41
5.11
4.74
4.44
4.04
4.02
3.98
3.94
3.85
3.83
3.78
3.63
3.42
3.40
3.27
3.24
3.22
3.09
2.75
2.66
2.63
2.54
2.51
2.29
2.27
1.33
0.17
0.61
3.71
0.55
0.59
0.33
1.70
1.80
1.05
1.80
3.12
0.45
1.20
0.57
1.00
f1 (ppm)

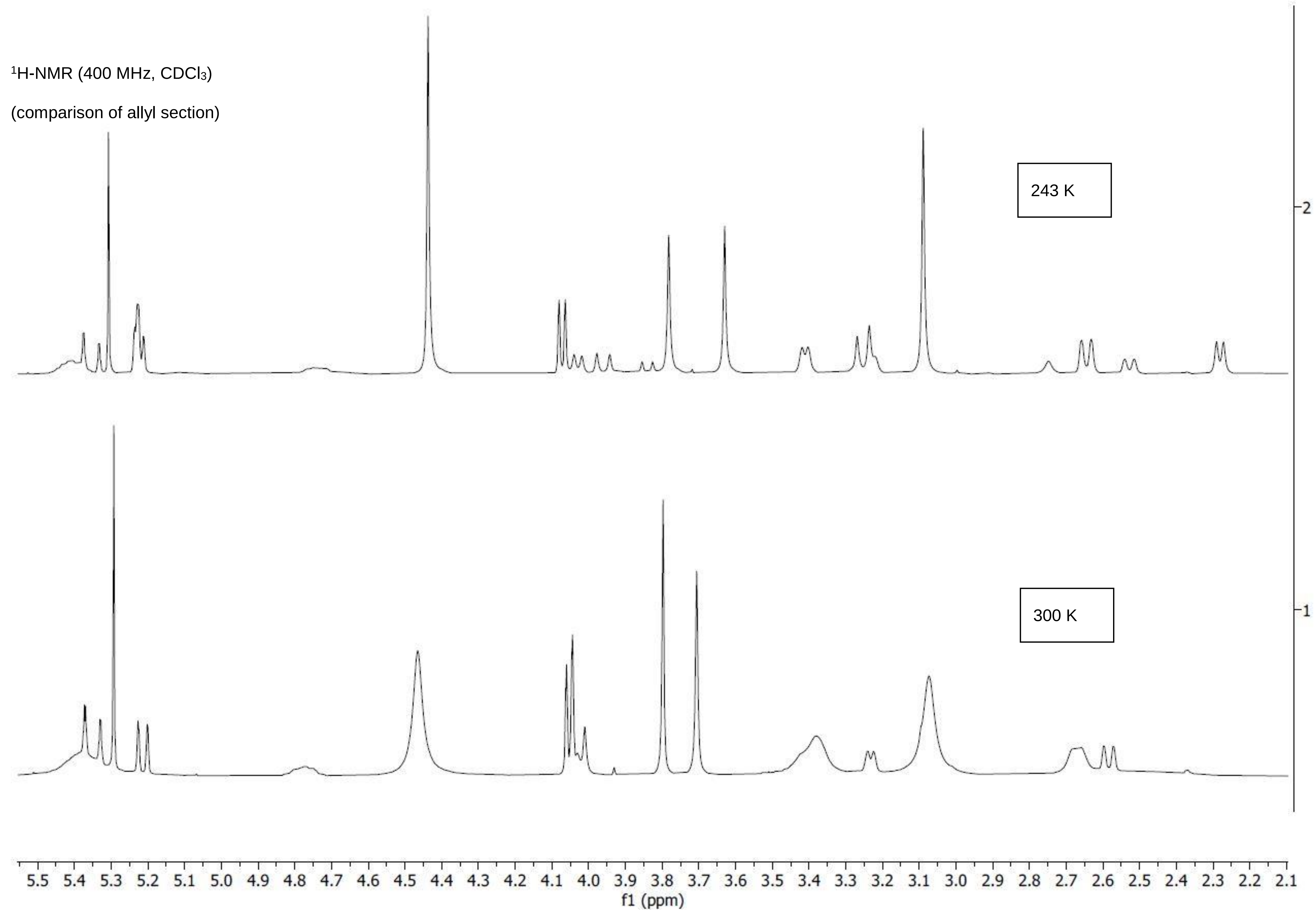

^{1}H-NMR (400 MHz, $CDCl_3$)
(comparison of allyl section)
243 K
300 K
2
1
5.5 5.4 5.3 5.2 5.1 5.0 4.9 4.8 4.7 4.6 4.5 4.4 4.3 4.2 4.1 4.0 3.9 3.8 3.7 3.6 3.5 3.4 3.3 3.2 3.1 3.0 2.9 2.8 2.7 2.6 2.5 2.4 2.3 2.2 2.1
f1 (ppm)

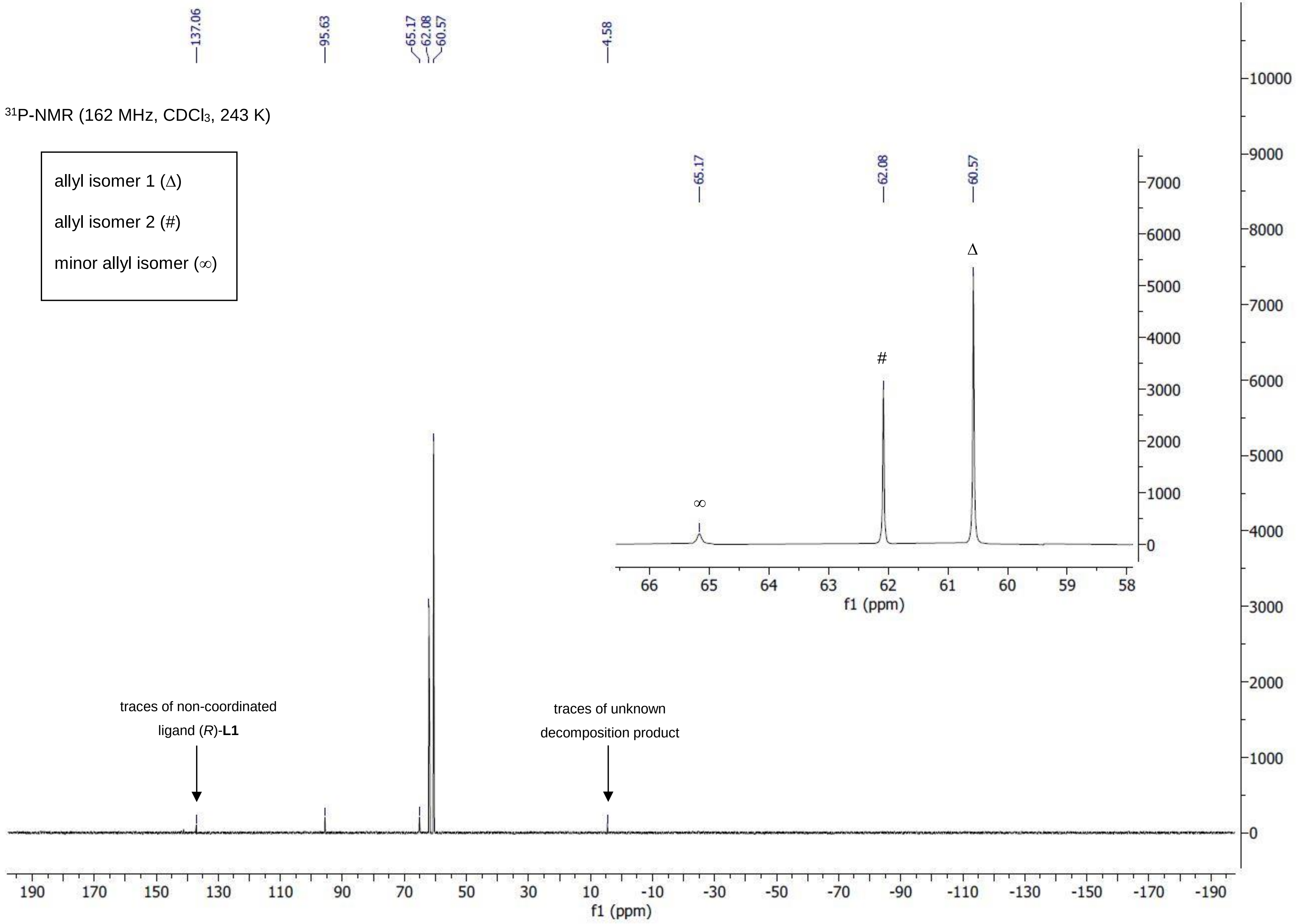

31P-NMR (162 MHz, CDCl3, 243 K)
allyl isomer 1 (Δ)
allyl isomer 2 (#)
minor allyl isomer (∞)
137.06
95.63
65.17
62.08
60.57
4.58
traces of non-coordinated ligand (R)-L1
traces of unknown decomposition product
f1 (ppm)

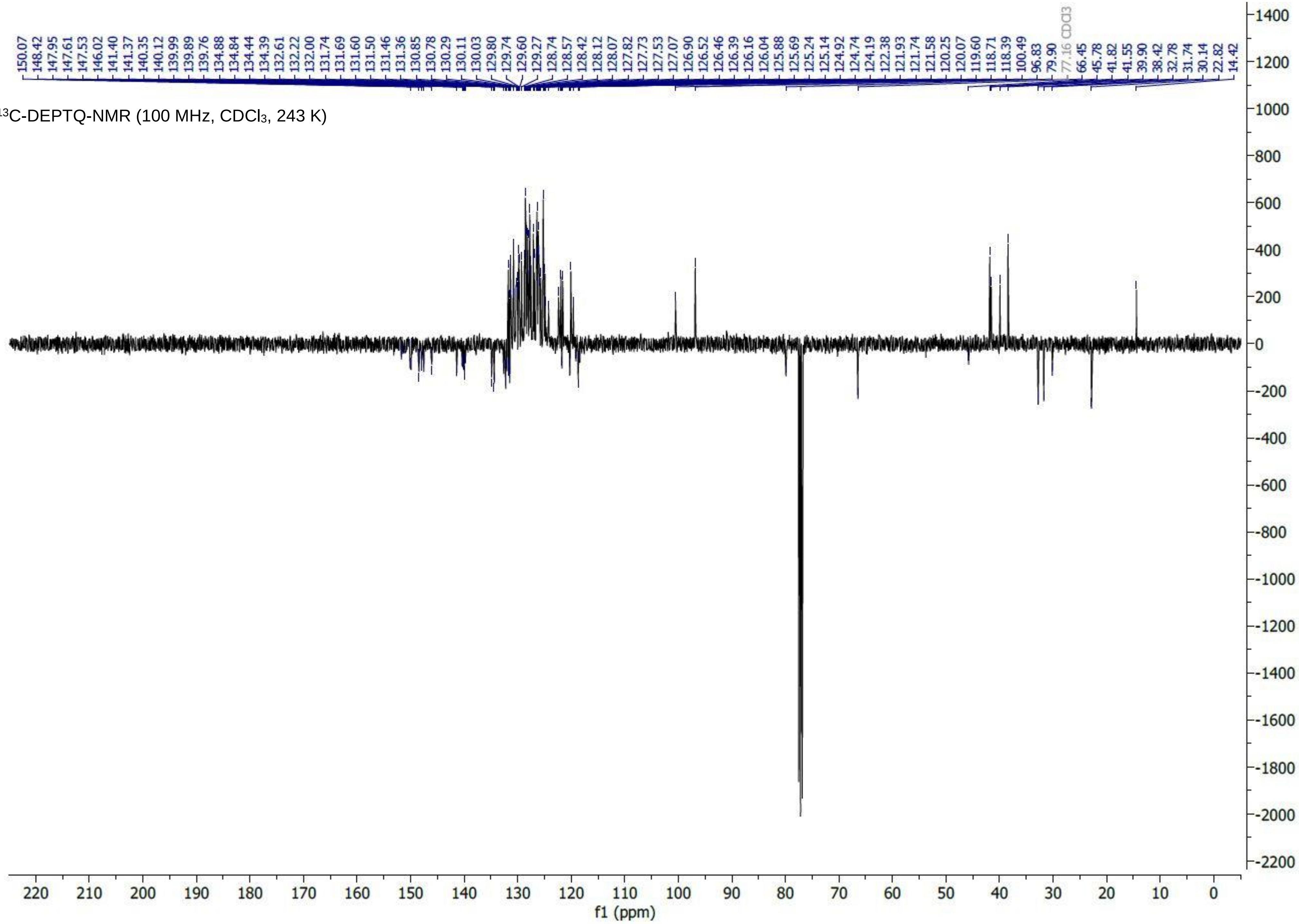
13C-DEPTQ-NMR (100 MHz, CDCl3, 243 K)
150.07
148.42
147.95
147.61
147.53
146.02
141.40
141.37
140.35
140.12
139.99
139.89
139.76
134.88
134.84
134.44
134.39
132.61
132.22
132.00
131.74
131.69
131.60
131.50
131.46
131.36
130.85
130.78
130.29
130.11
130.03
129.80
129.74
129.60
129.27
128.74
128.57
128.42
128.12
128.07
127.82
127.73
127.53
127.07
126.90
126.52
126.46
126.39
126.16
126.04
125.88
125.69
125.24
125.14
124.92
124.74
124.19
122.38
121.93
121.74
121.58
120.25
120.07
119.60
118.71
118.39
100.49
96.83
79.90
77.16 CDCl3
66.45
45.78
41.82
41.55
39.90
38.42
32.78
31.74
30.14
22.82
14.42
f1 (ppm)

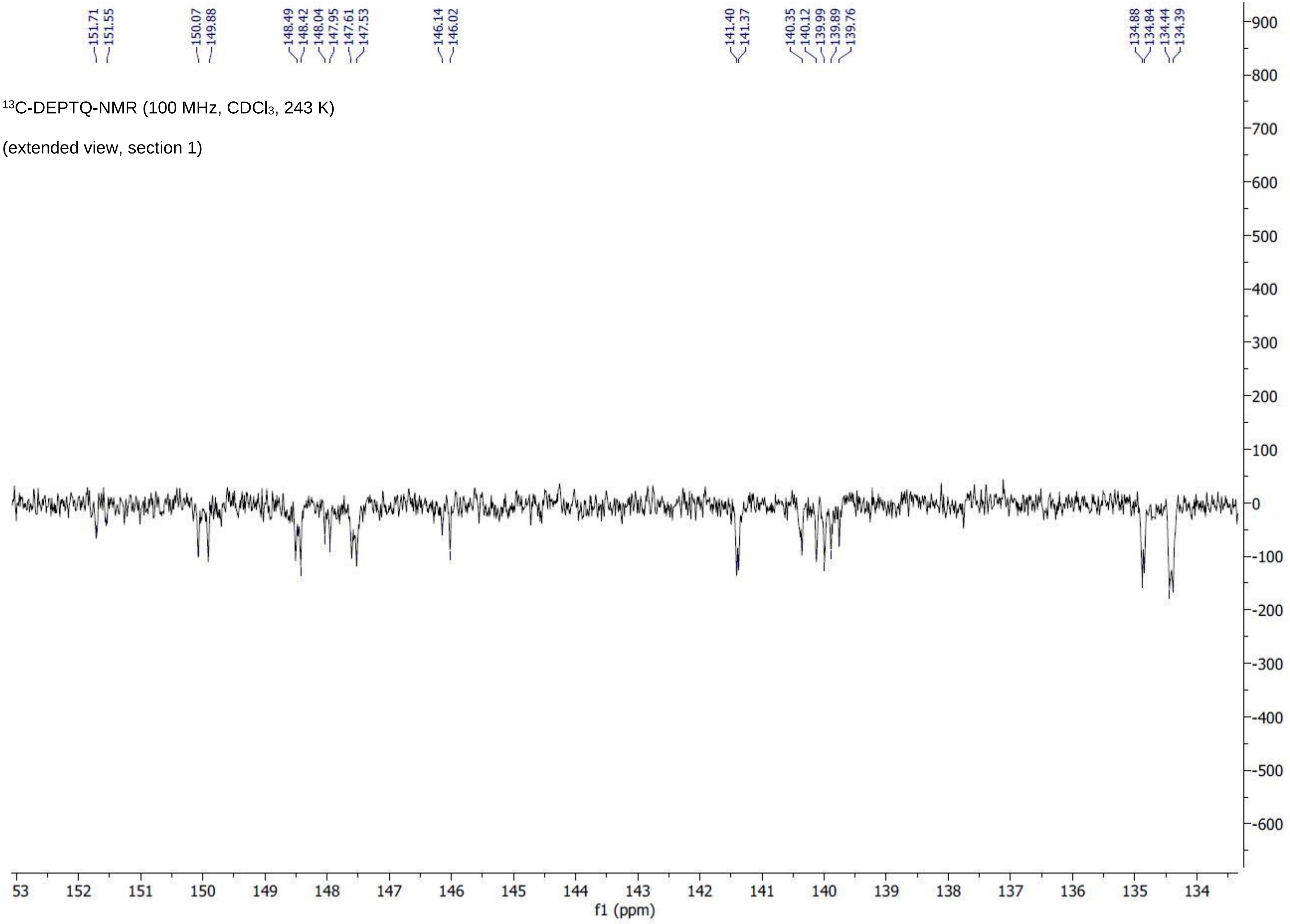
151.71
151.55
150.07
149.88
148.49
148.42
148.04
147.95
147.61
147.53
146.14
146.02
141.40
141.37
140.35
140.12
139.99
139.89
139.76
134.88
134.84
134.44
134.39
^{13}C-DEPTQ-NMR (100 MHz, $CDCl_3$, 243 K)
(extended view, section 1)
f1 (ppm)

^{13}C-DEPTQ-NMR (100 MHz, $CDCl_3$, 243 K)

(extended view, section 2)

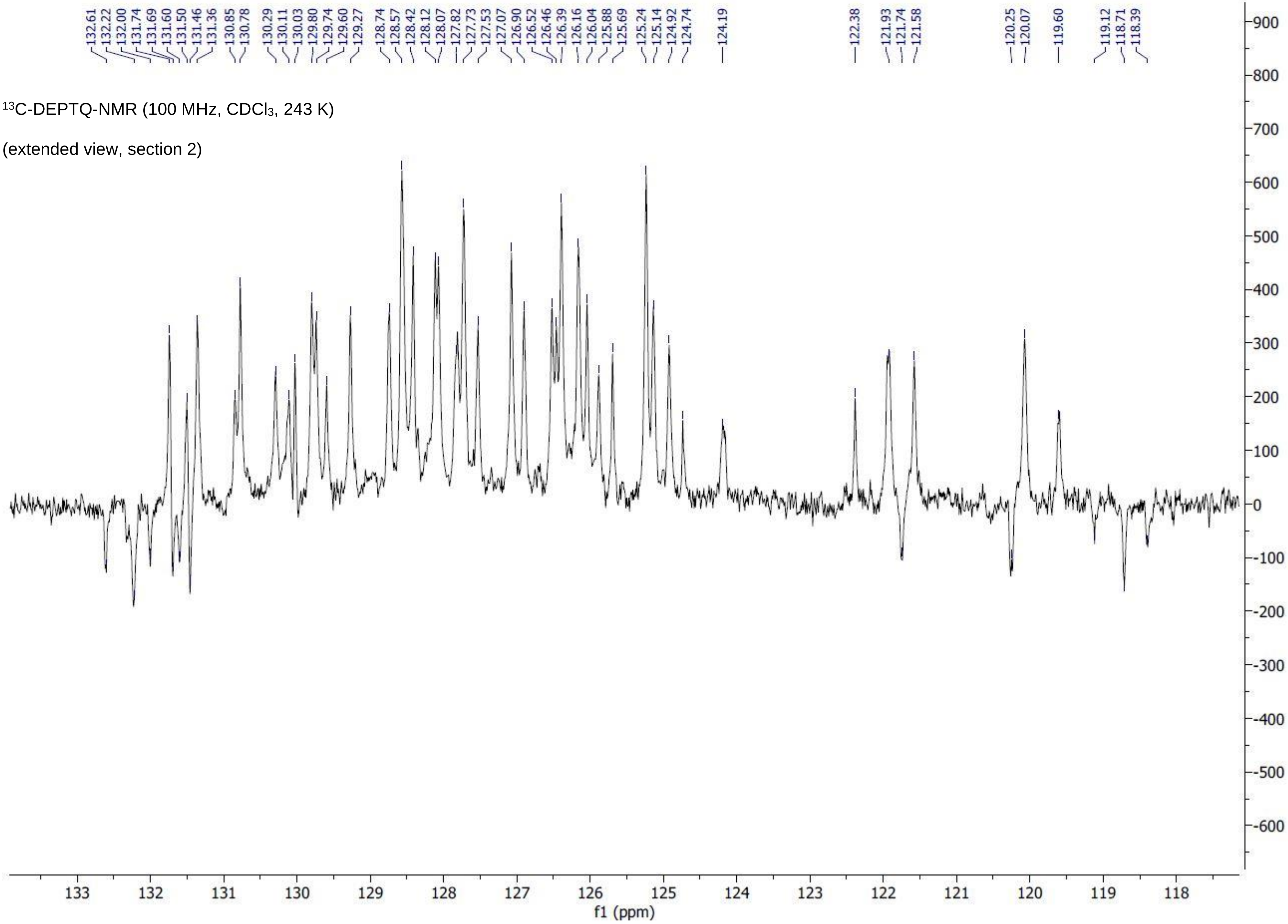

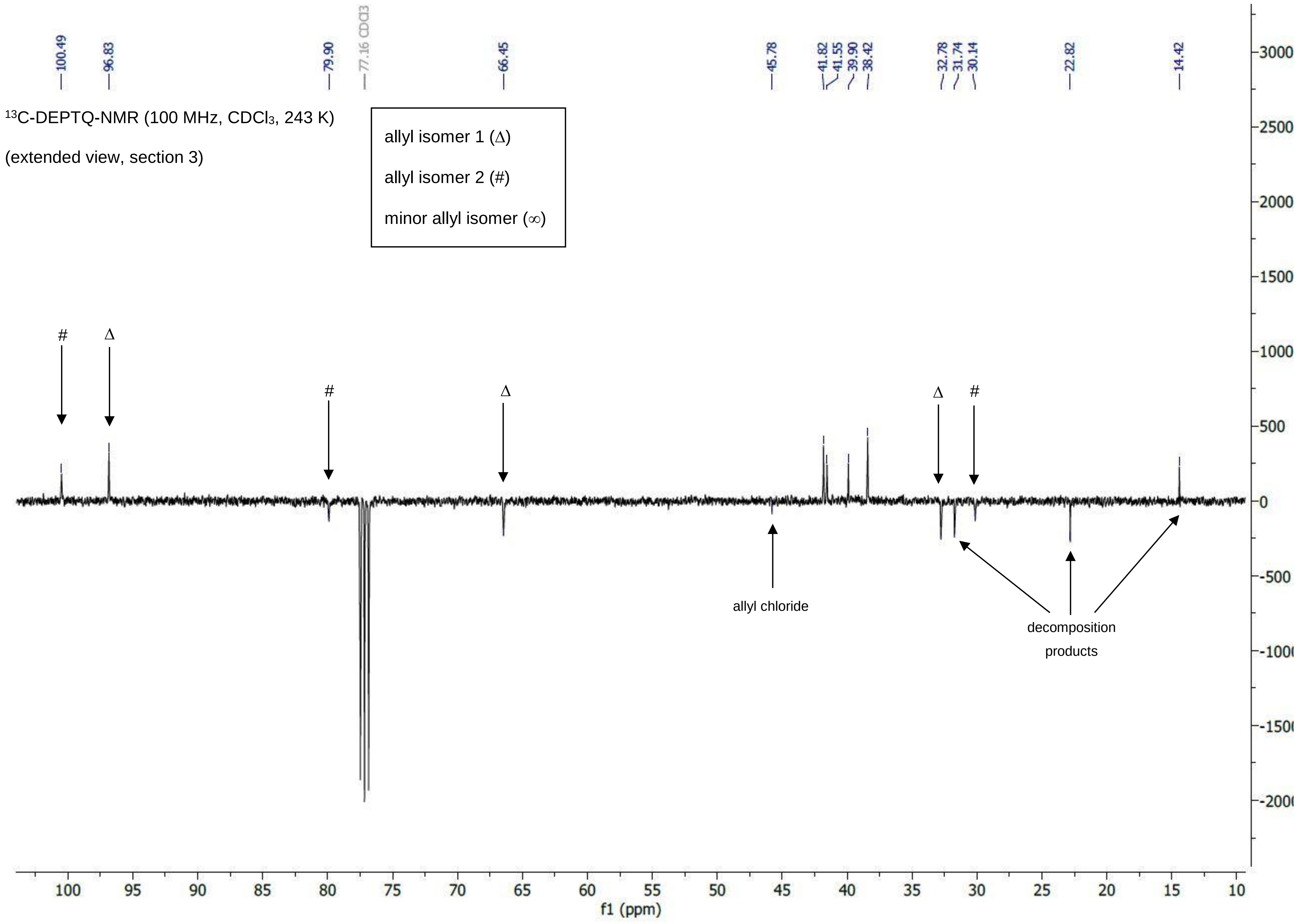

^{13}C-DEPTQ-NMR (100 MHz, CDCl$_3$, 243 K)
(extended view, section 3)
allyl isomer 1 (Δ)
allyl isomer 2 (#)
minor allyl isomer (∞)
100.49
96.83
79.90
77.16 CDCl3
66.45
45.78
41.82
41.55
39.90
38.42
32.78
31.74
30.14
22.82
14.42
#
Δ
#
Δ
Δ
#
allyl chloride
decomposition products
f1 (ppm)

^{31}P-NMR (162 MHz, CD_2Cl_2, 243 K)

(showing a different population of allyl isomers compared to $CDCl_3$)

allyl isomer 1 (Δ)

allyl isomer 2 (#)

minor allyl isomer (∞)

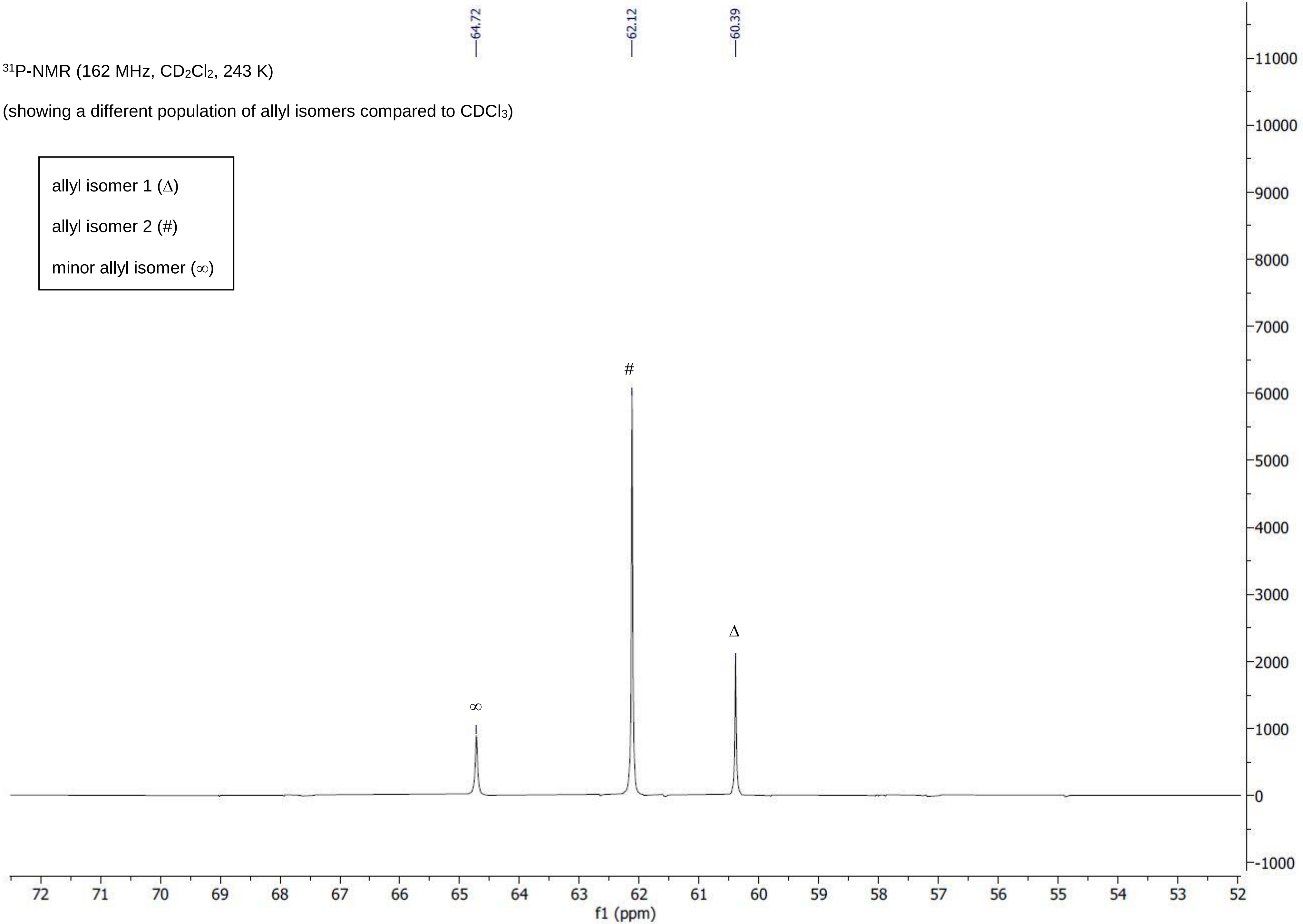

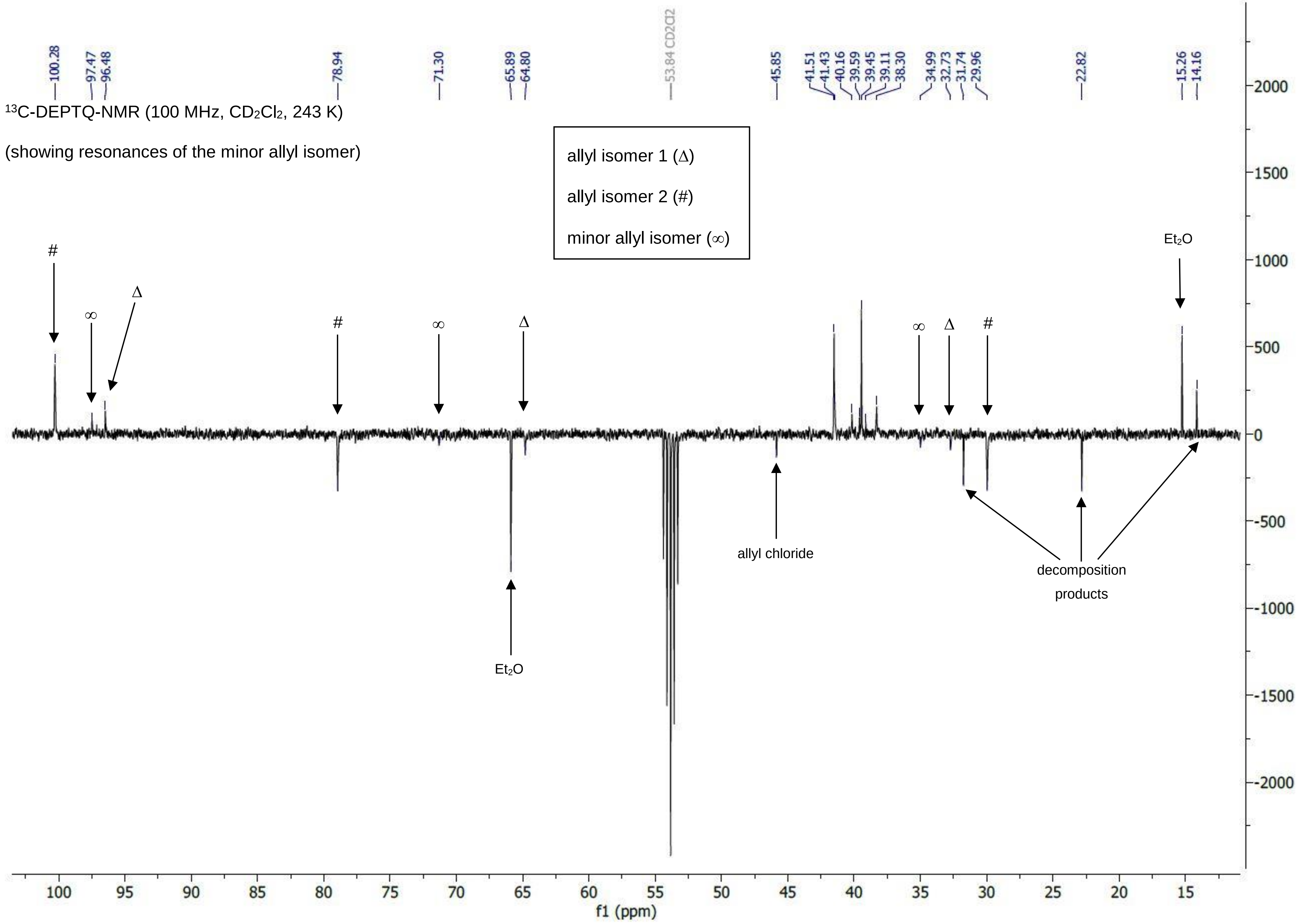

13C-DEPTQ-NMR (100 MHz, CD2Cl2, 243 K)
(showing resonances of the minor allyl isomer)
allyl isomer 1 (Δ)
allyl isomer 2 (#)
minor allyl isomer (∞)
100.28
97.47
96.48
78.94
71.30
65.89
64.80
53.84 CD2Cl2
45.85
41.51
41.43
40.16
39.59
39.45
39.11
38.30
34.99
32.73
31.74
29.96
22.82
15.26
14.16
Et2O
Et2O
allyl chloride
decomposition products
f1 (ppm)

12.5. prenylated and allylated compounds

^{1}H-NMR (300 MHz, $CDCl_3$)

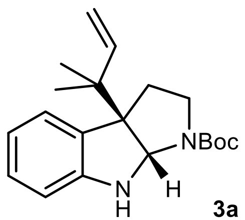

3a

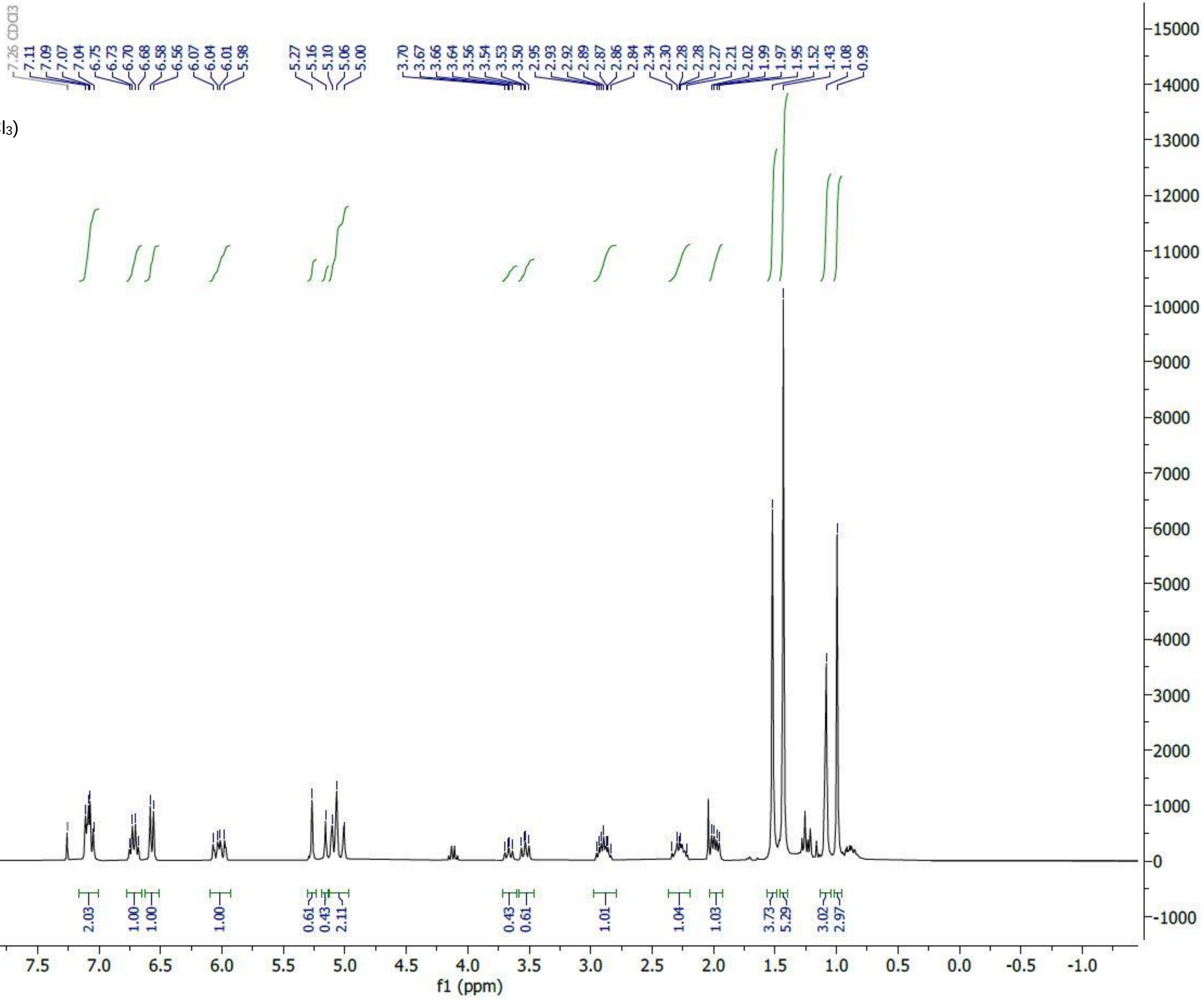

^{13}C-DEPTQ-NMR (76 MHz, $CDCl_3$)

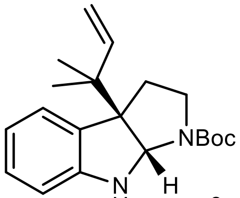

3a

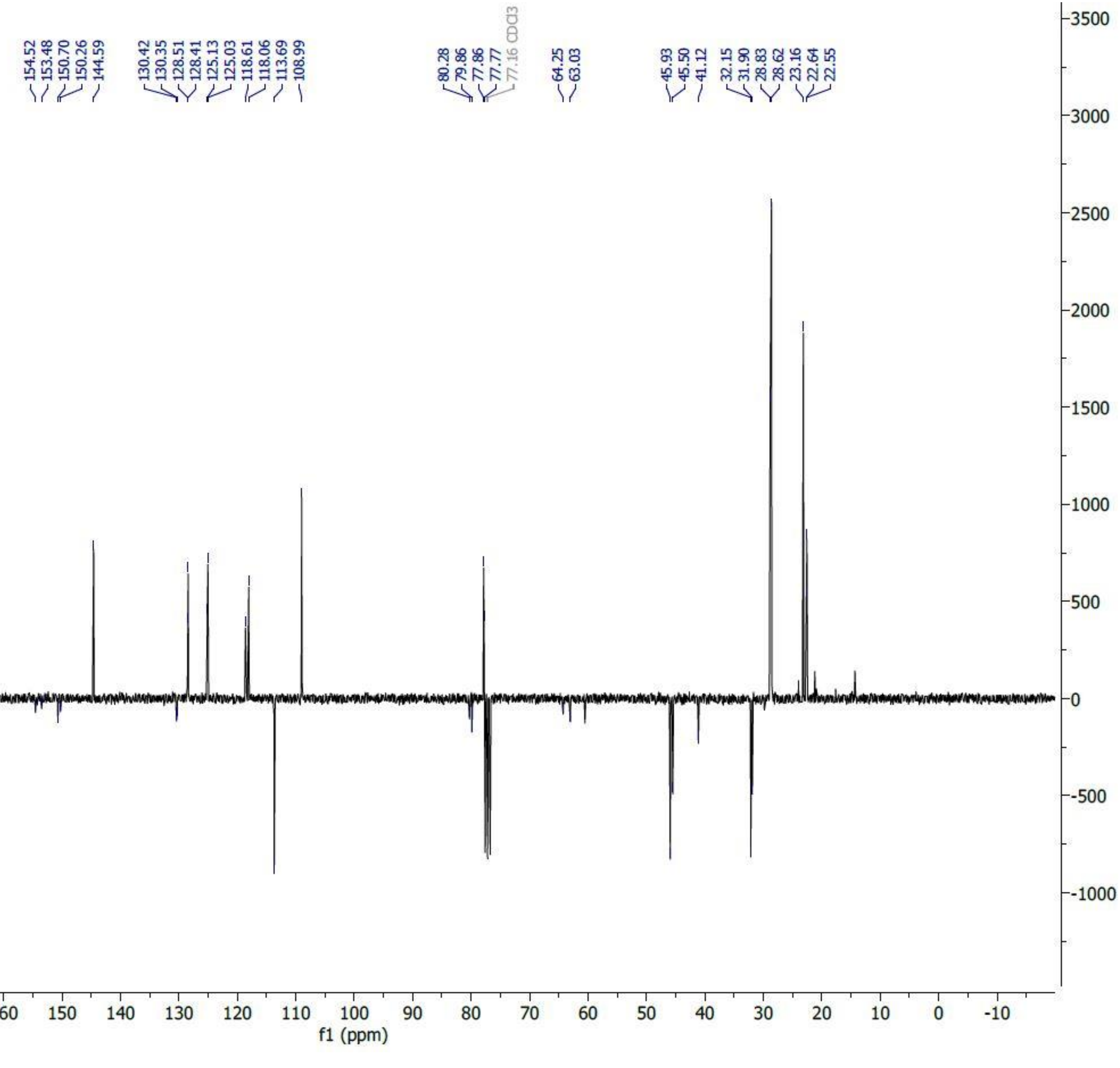

^{1}H-NMR (400 MHz, $CDCl_3$)

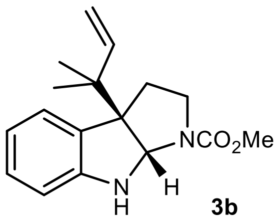

3b

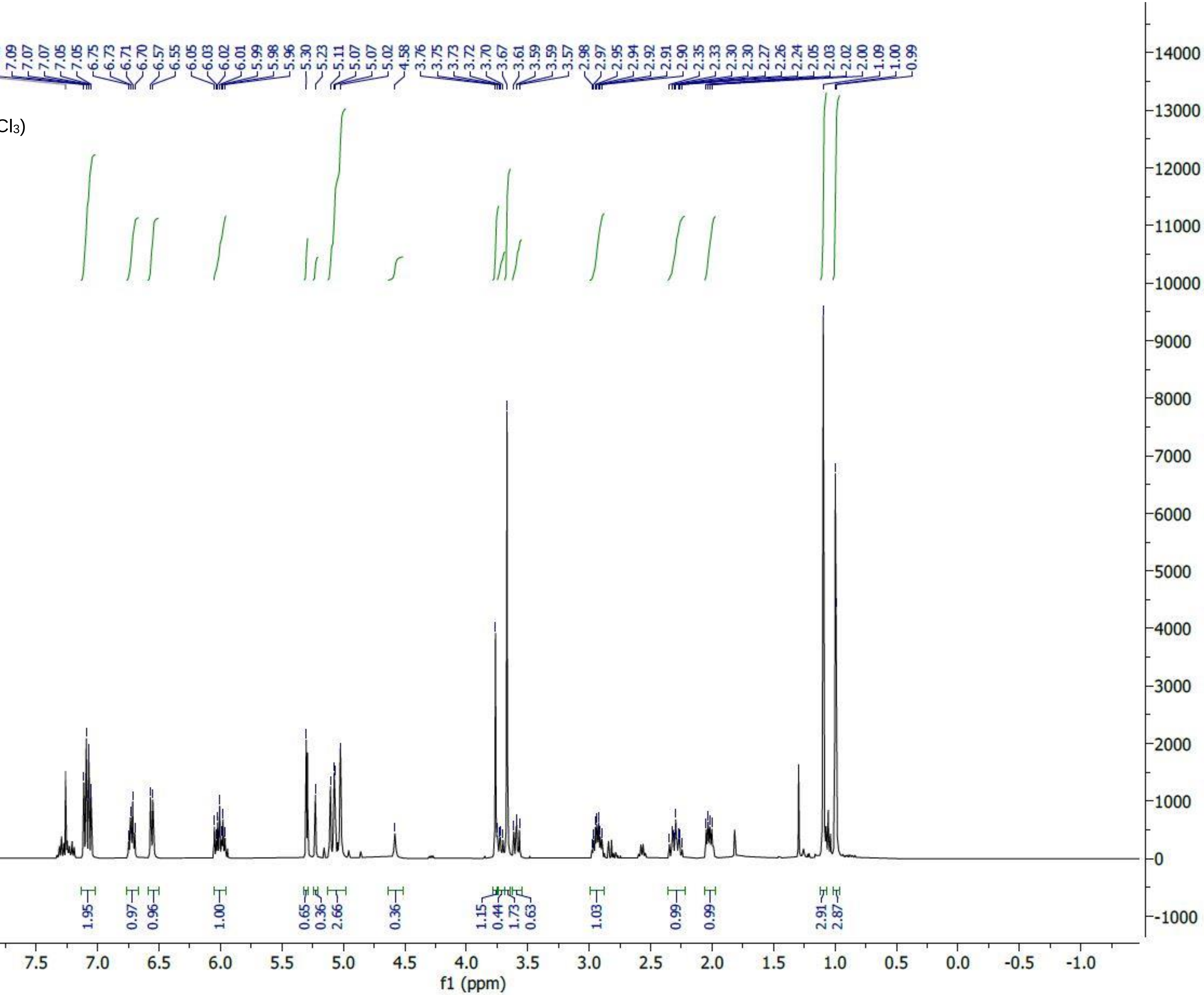

^{13}C-DEPTQ-NMR (100 MHz, $CDCl_3$)

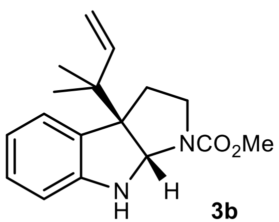

3b

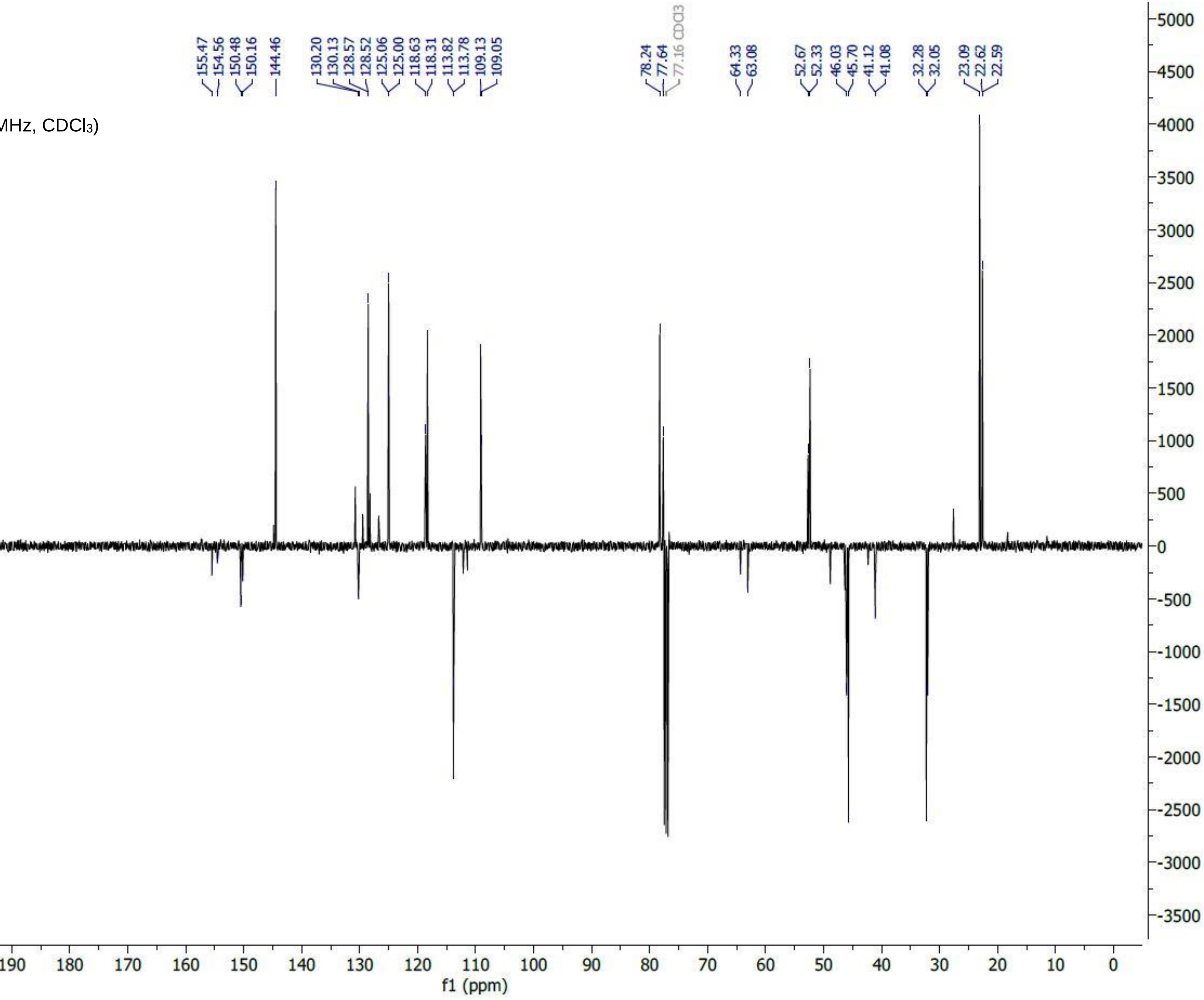

^{1}H-NMR (600 MHz, $CDCl_3$)

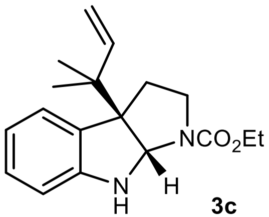

3c

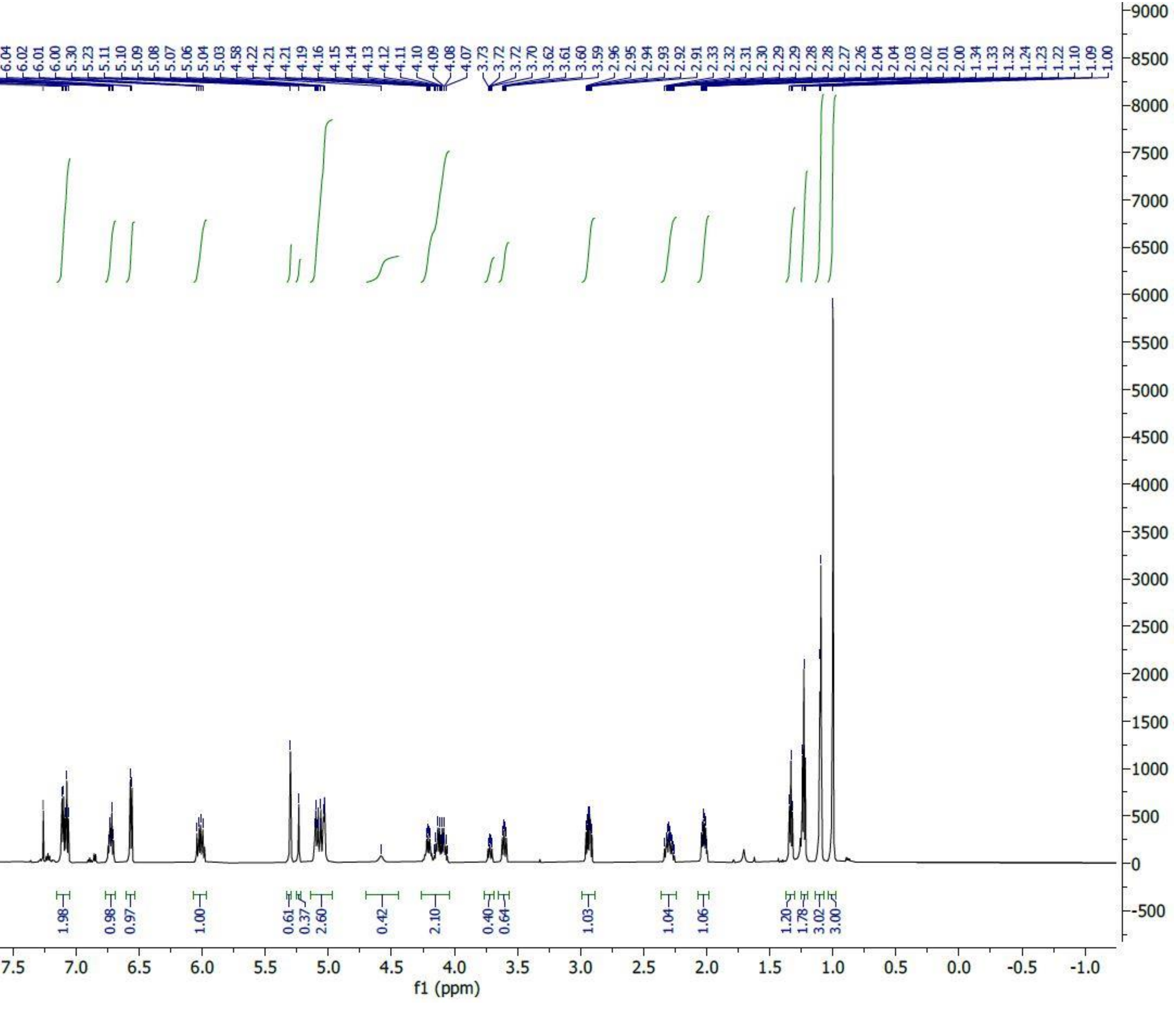

13C-DEPTQ-NMR (150 MHz, $CDCl_3$)

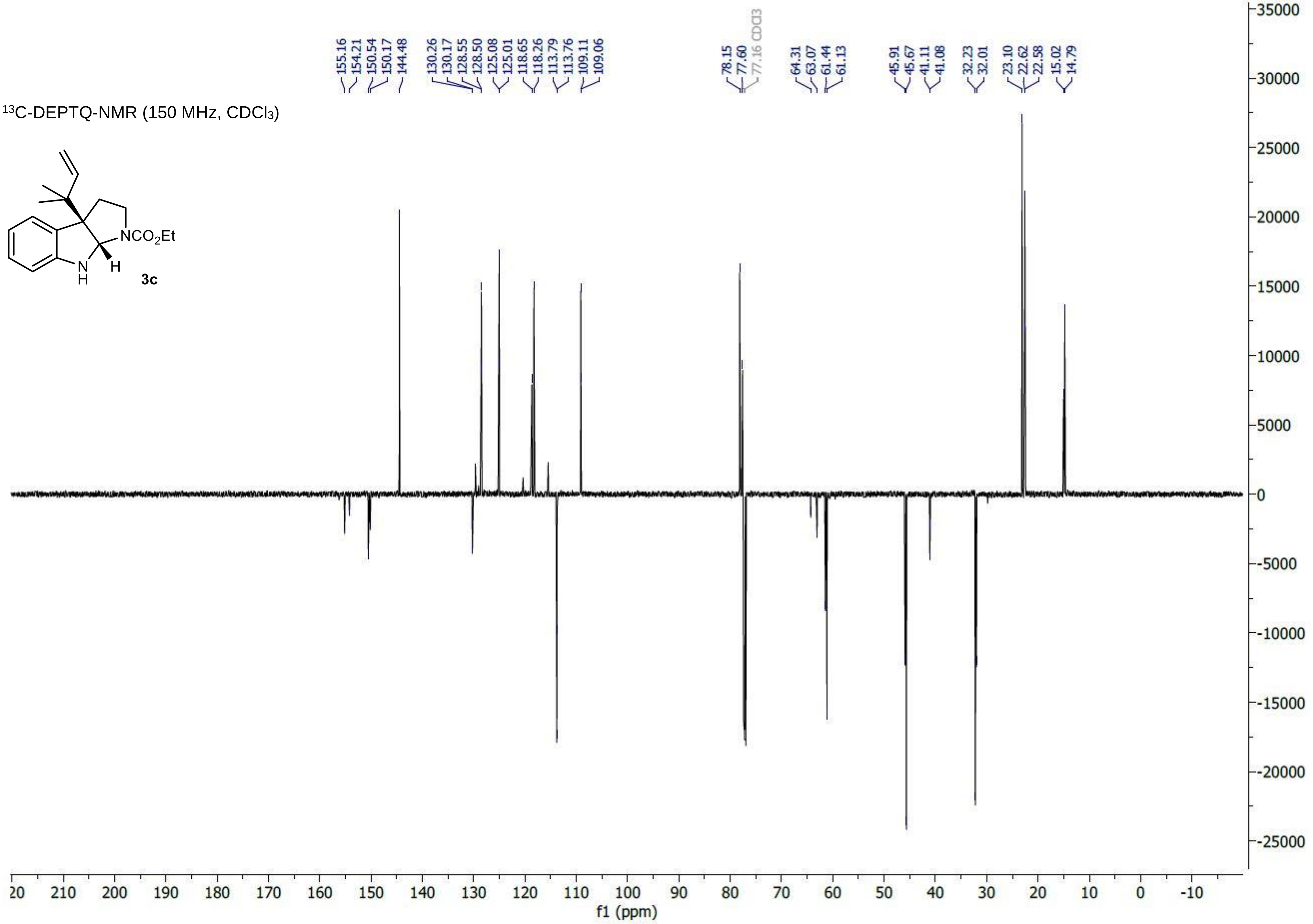

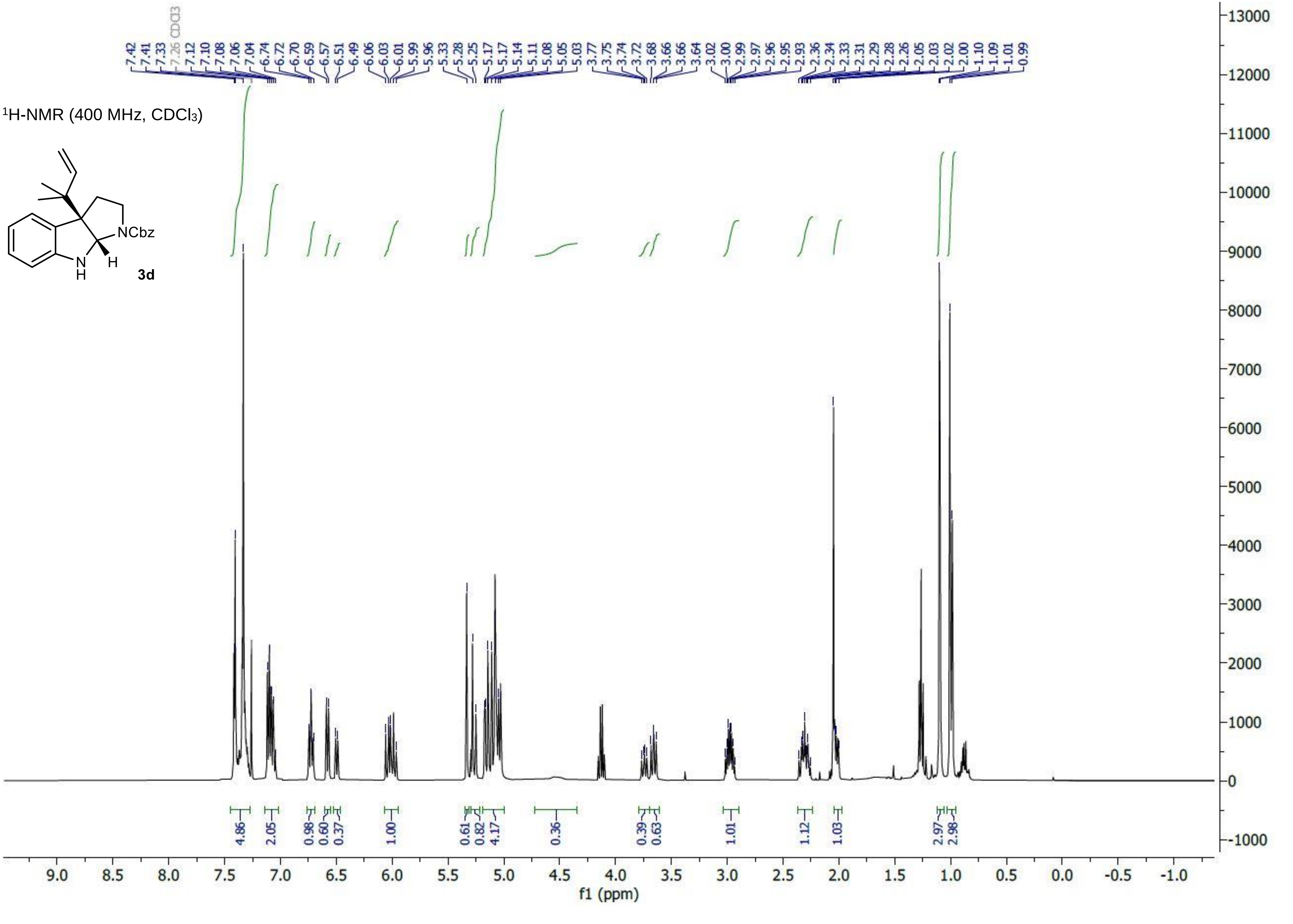

^{1}H-NMR (400 MHz, CDCl$_3$)
NCbz
N
H
H
3d
CDCl3
7.42 7.41 7.33 7.26 7.12 7.10 7.08 7.06 7.04 6.74 6.72 6.70 6.59 6.57 6.51 6.49 6.06 6.03 6.01 5.99 5.96 5.33 5.28 5.25 5.17 5.17 5.14 5.11 5.08 5.05 5.03 3.77 3.75 3.74 3.72 3.68 3.66 3.66 3.64 3.02 3.00 2.99 2.97 2.96 2.95 2.93 2.36 2.34 2.33 2.31 2.29 2.28 2.26 2.05 2.03 2.02 2.00 1.10 1.09 1.01 0.99
4.86 2.05 0.98 0.60 0.37 1.00 0.61 0.82 4.17 0.36 0.39 0.63 1.01 1.12 1.03 2.97 2.98
f1 (ppm)

^{13}C-DEPTQ-NMR (100 MHz, $CDCl_3$)

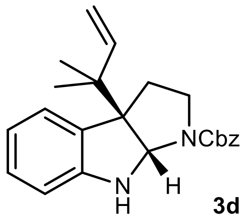

3d

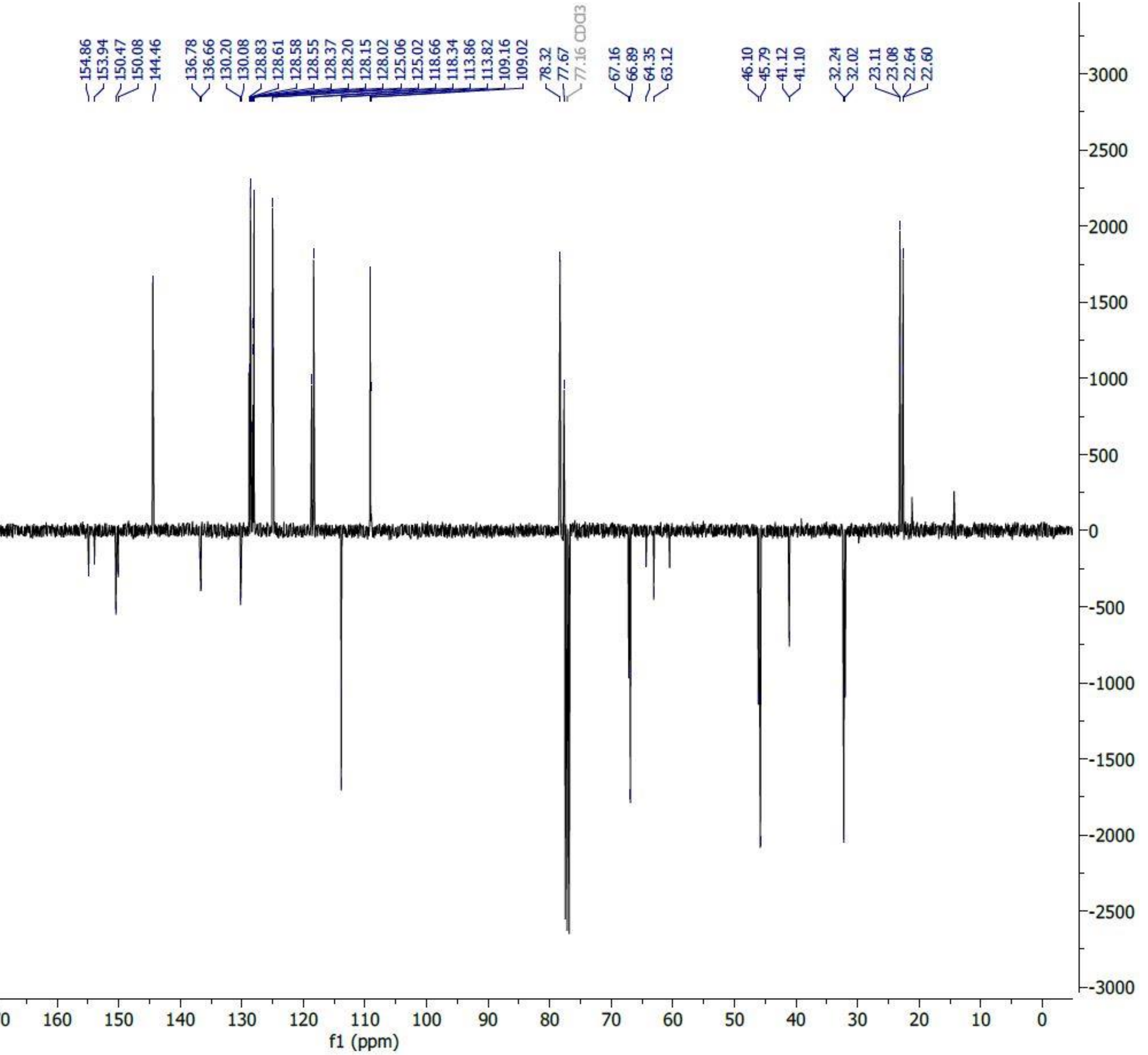

^{1}H-NMR (500 MHz, $CDCl_3$)

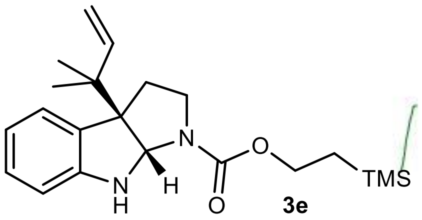

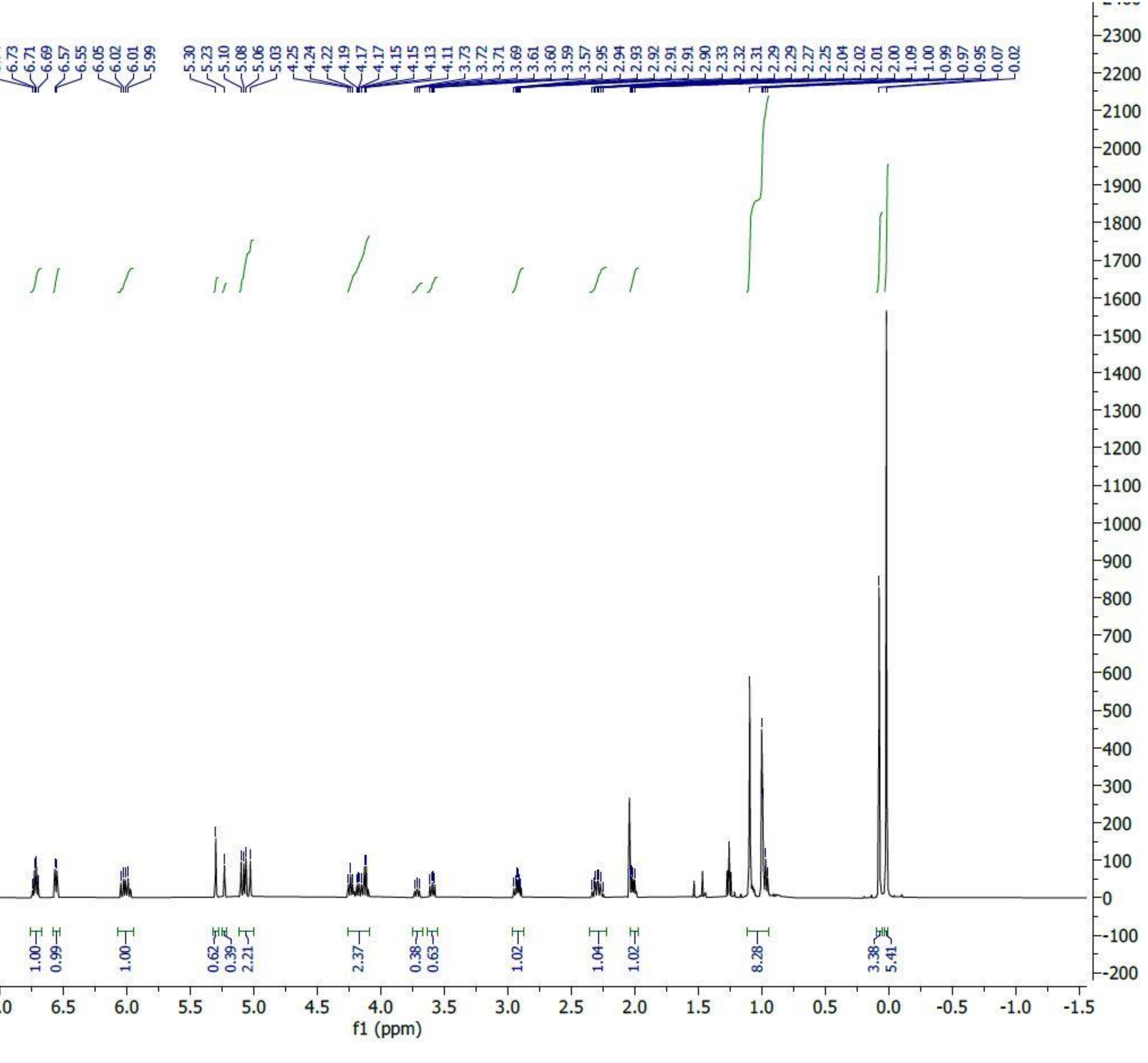

^{13}C-DEPTQ-NMR (125 MHz, $CDCl_3$)

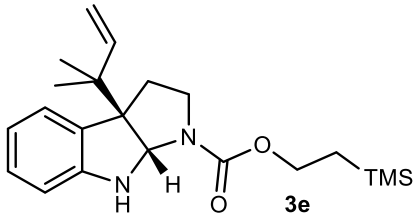

3e

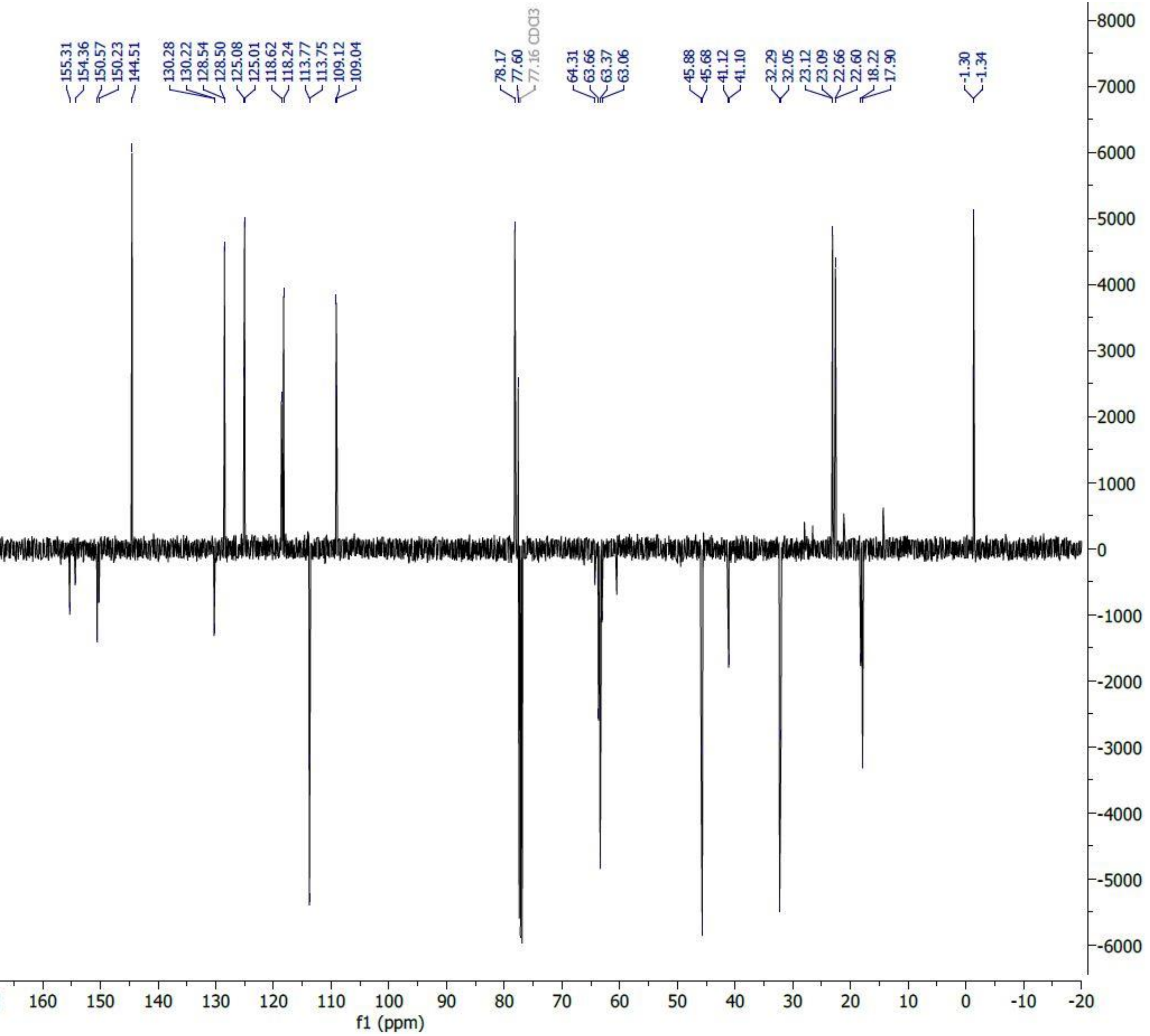

^{1}H-NMR (500 MHz, $CDCl_3$)

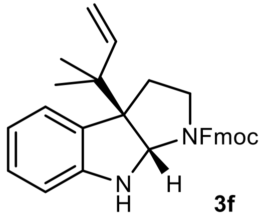

3f

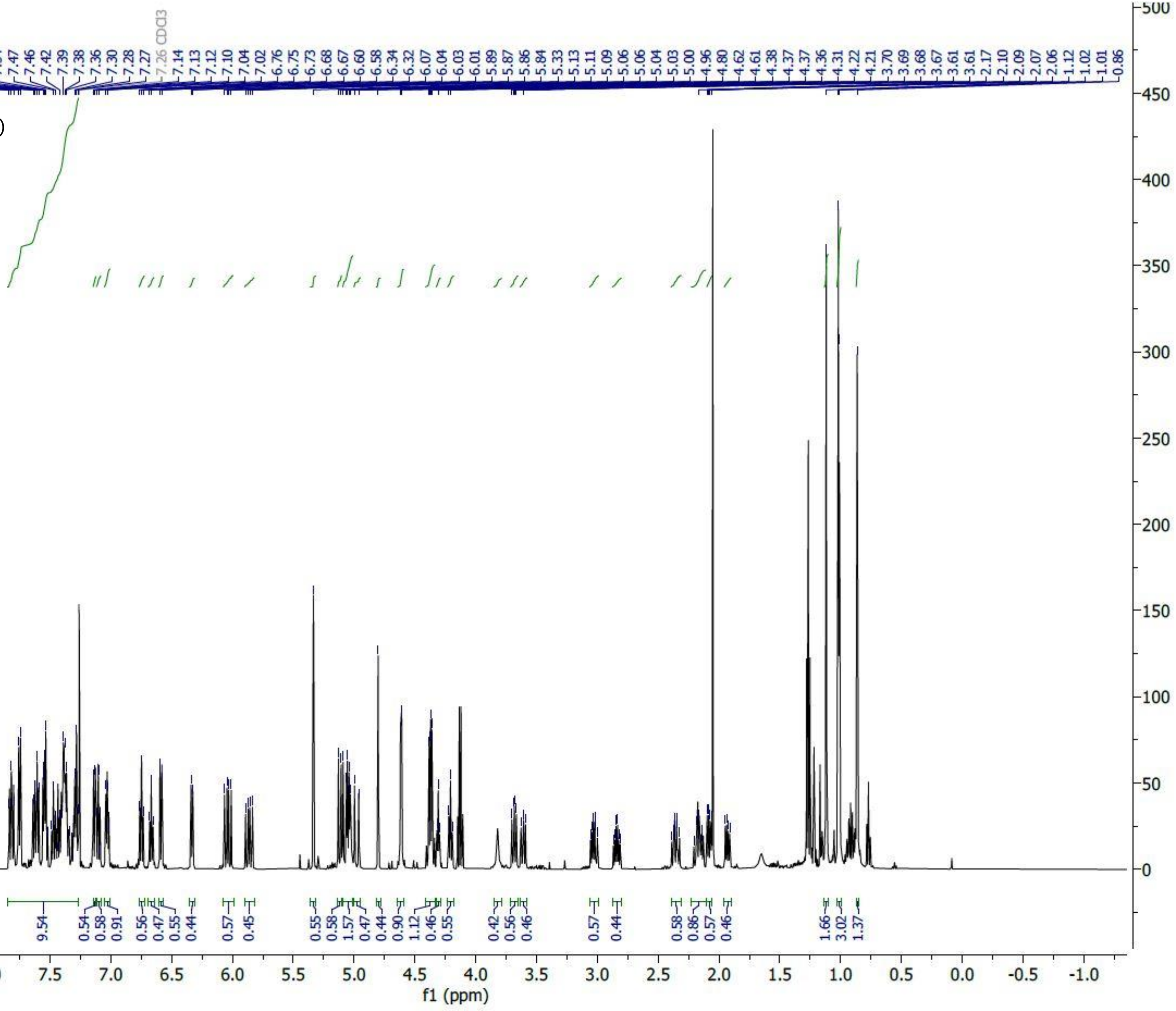

^{13}C-DEPTQ-NMR (125 MHz, $CDCl_3$)

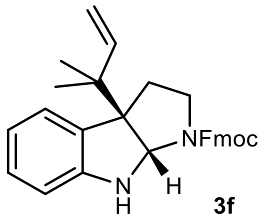

3f

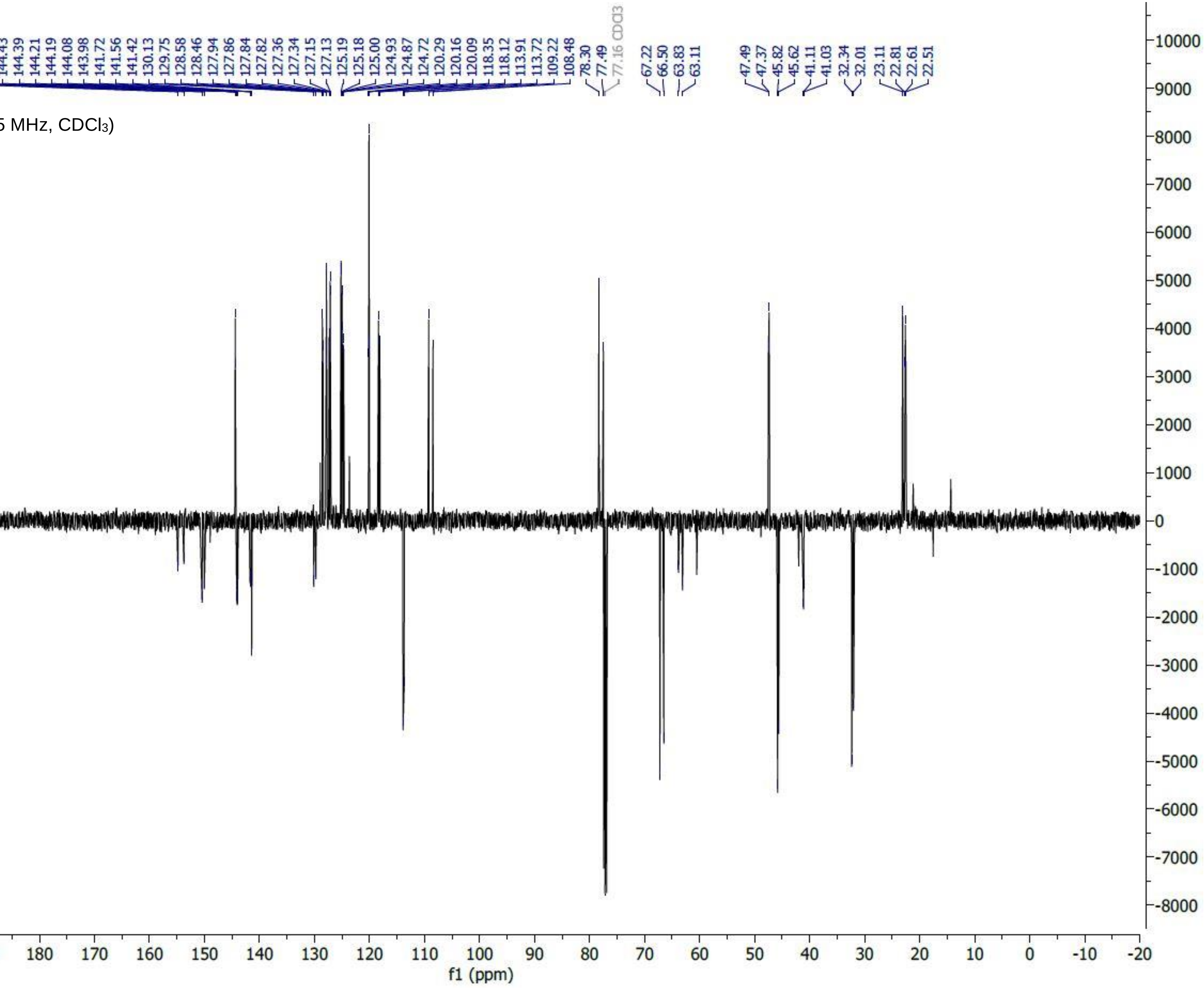

^{1}H-NMR (600 MHz, $CDCl_3$)

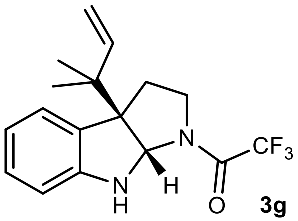

3g

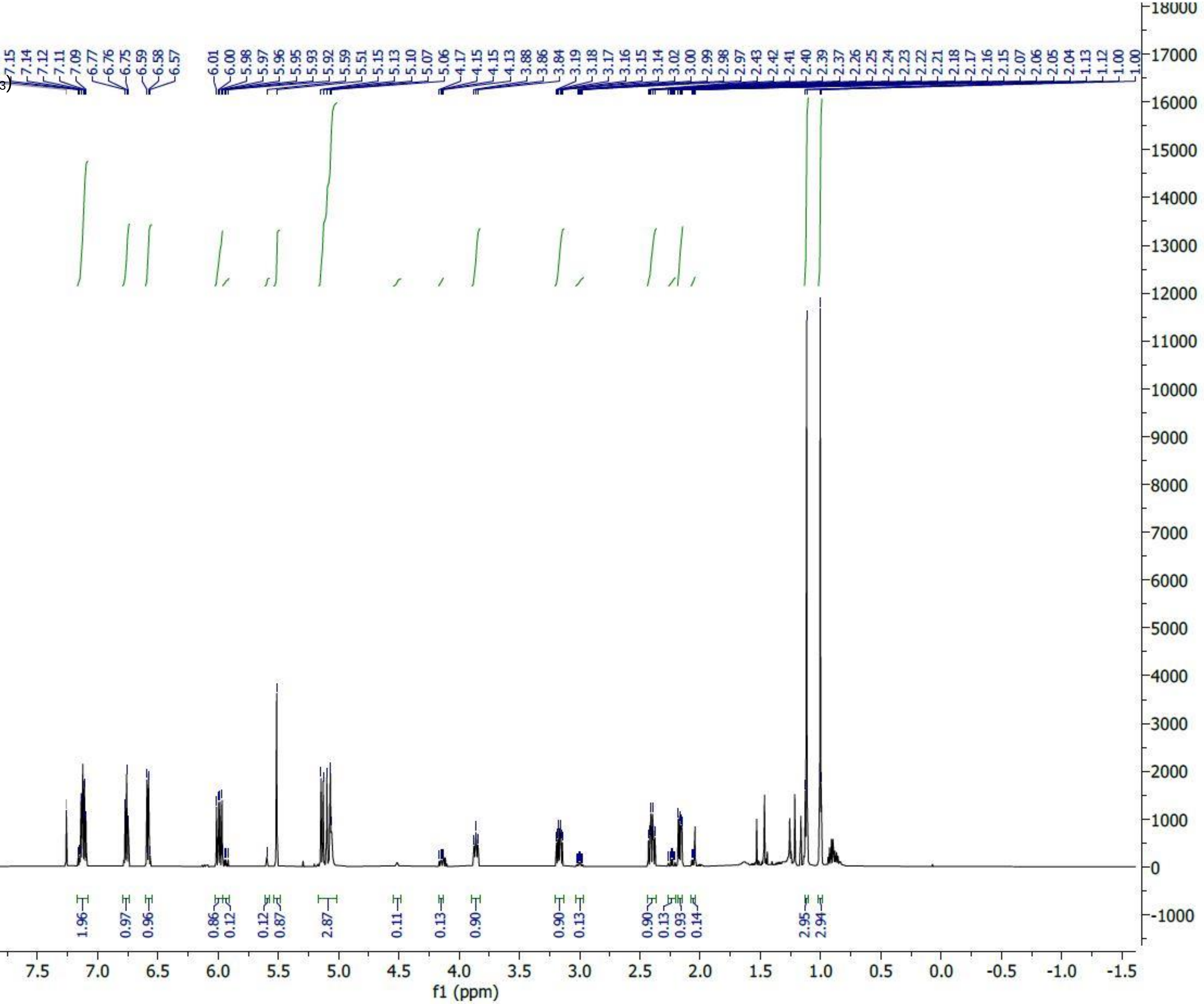

^{13}C-DEPTQ-NMR (150 MHz, $CDCl_3$)

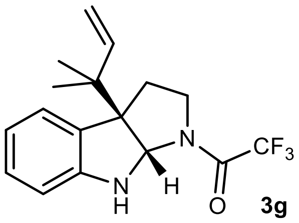

3g

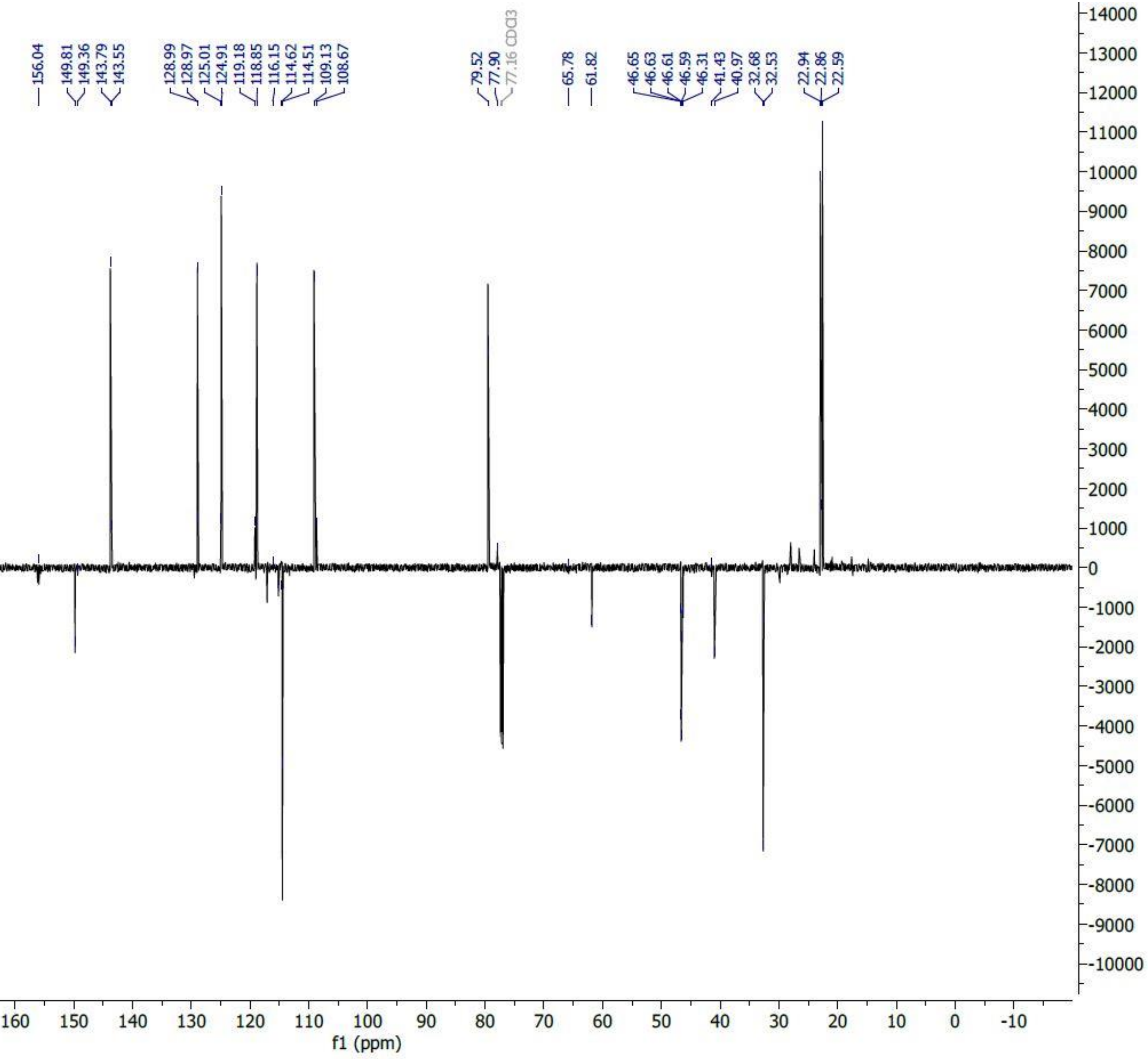

^{19}F-NMR (565 MHz, $CDCl_3$)

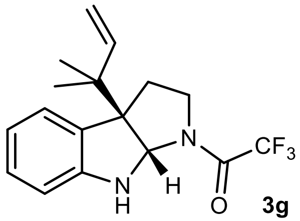

3g

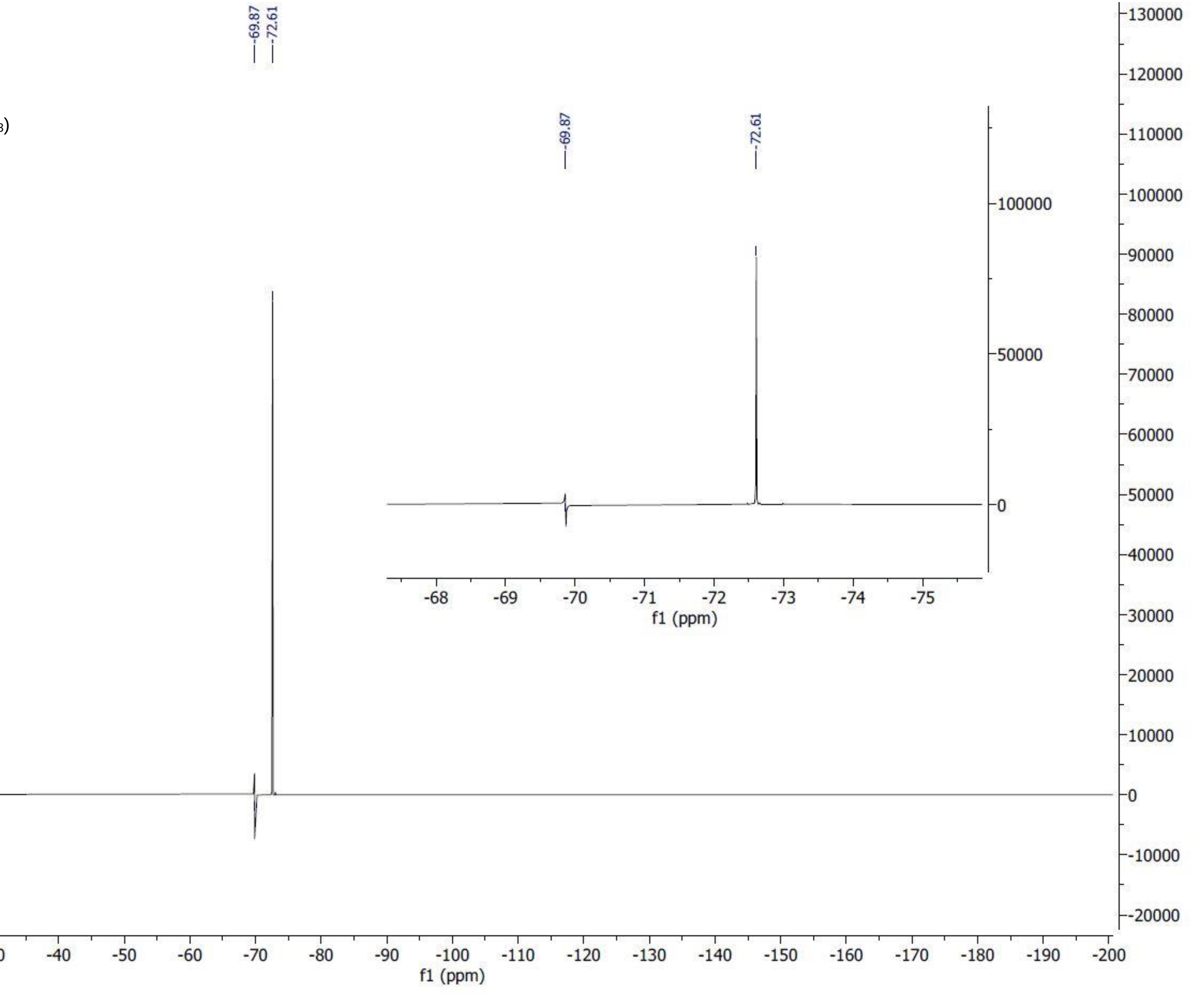

^{1}H-NMR (600 MHz, $CDCl_3$)

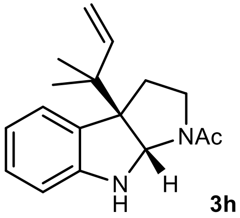

3h

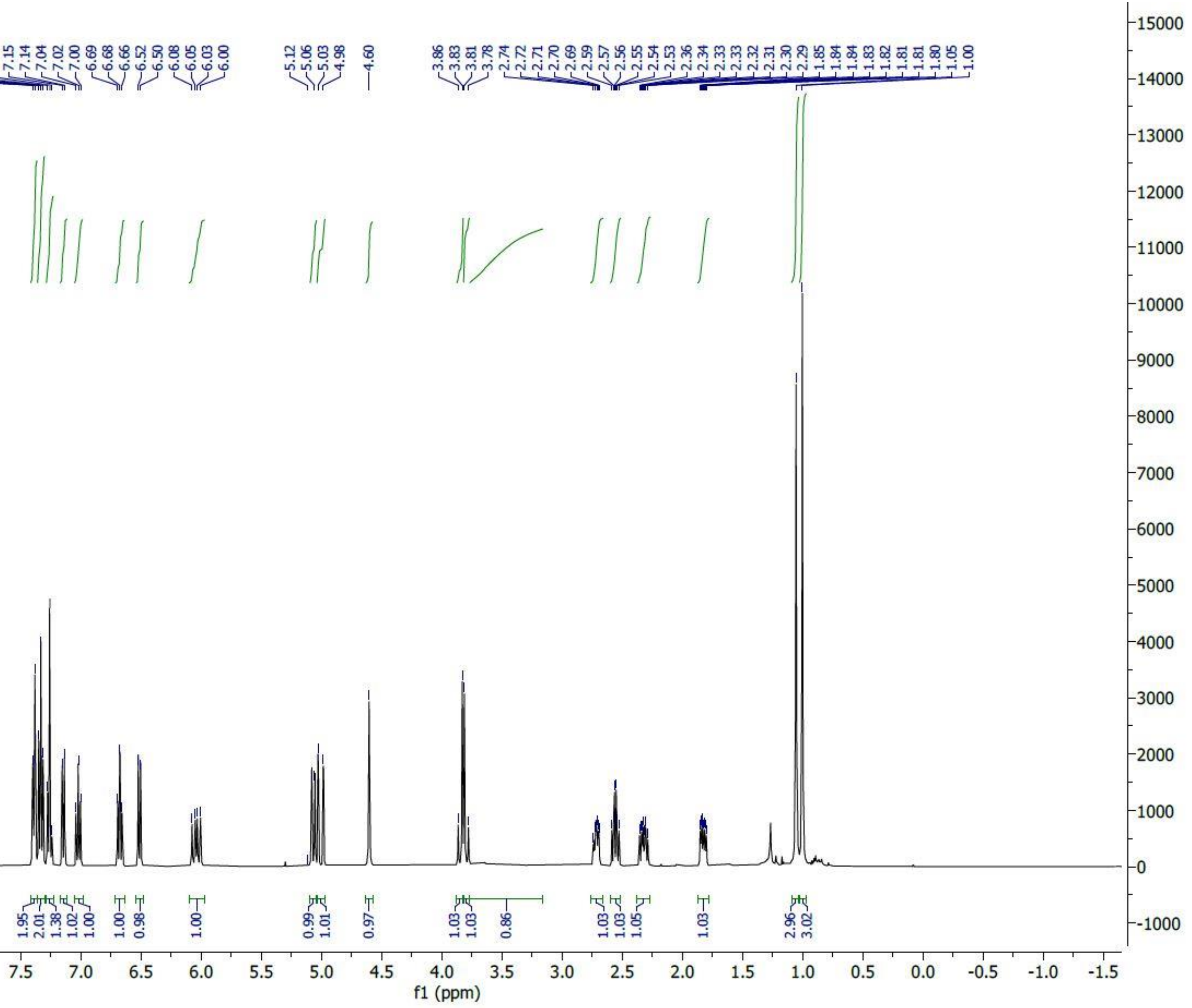

^{13}C-DEPTQ-NMR (150 MHz, $CDCl_3$)

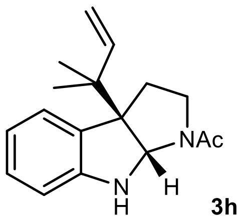

3h

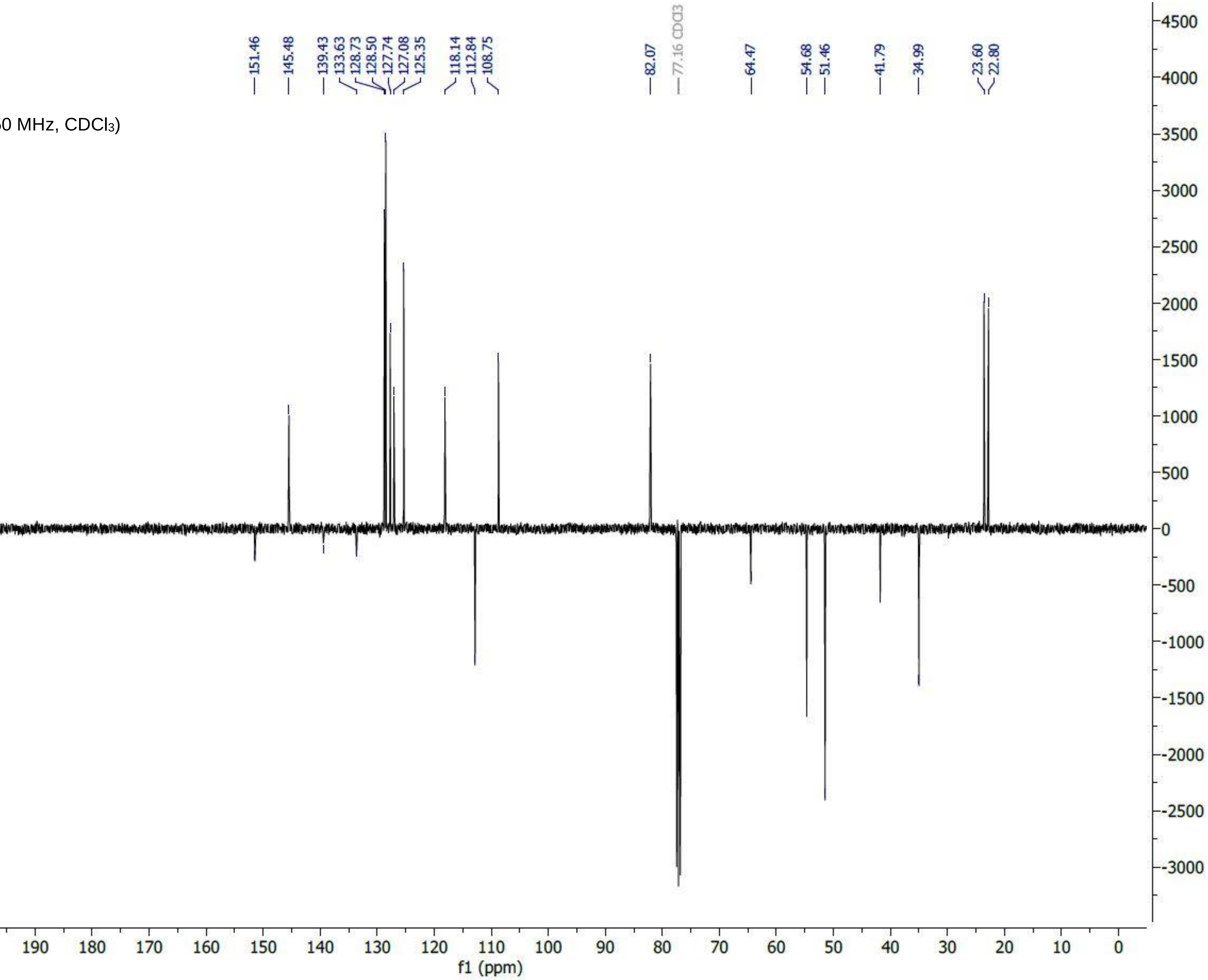

^{1}H-NMR (400 MHz, $CDCl_3$)

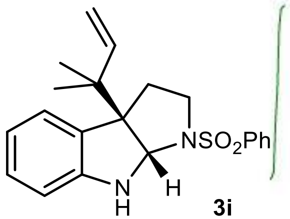

3i

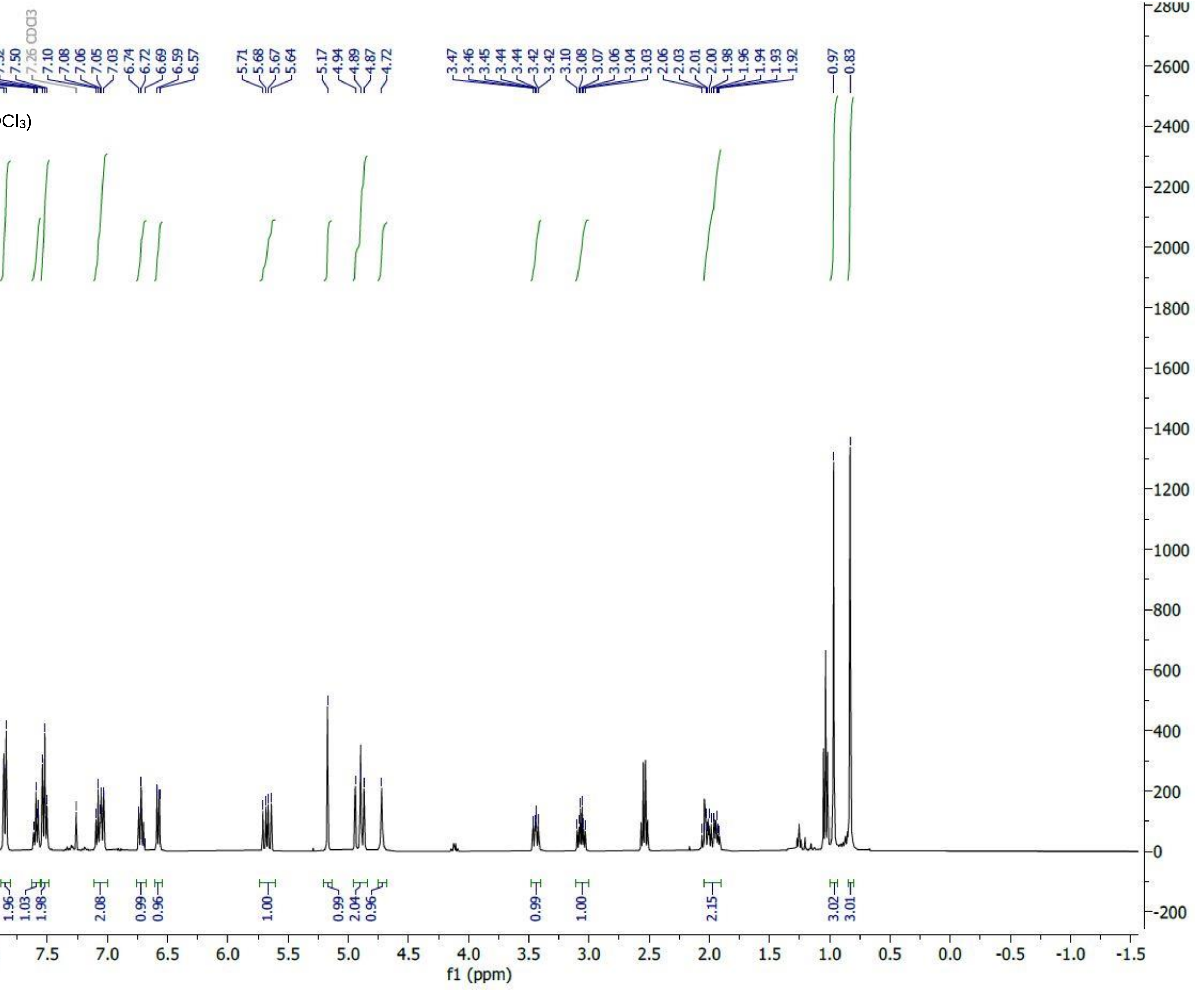

^{13}C-DEPTQ-NMR (100 MHz, $CDCl_3$)

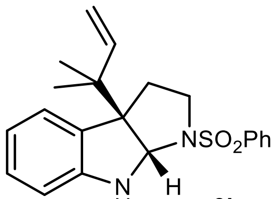

3i

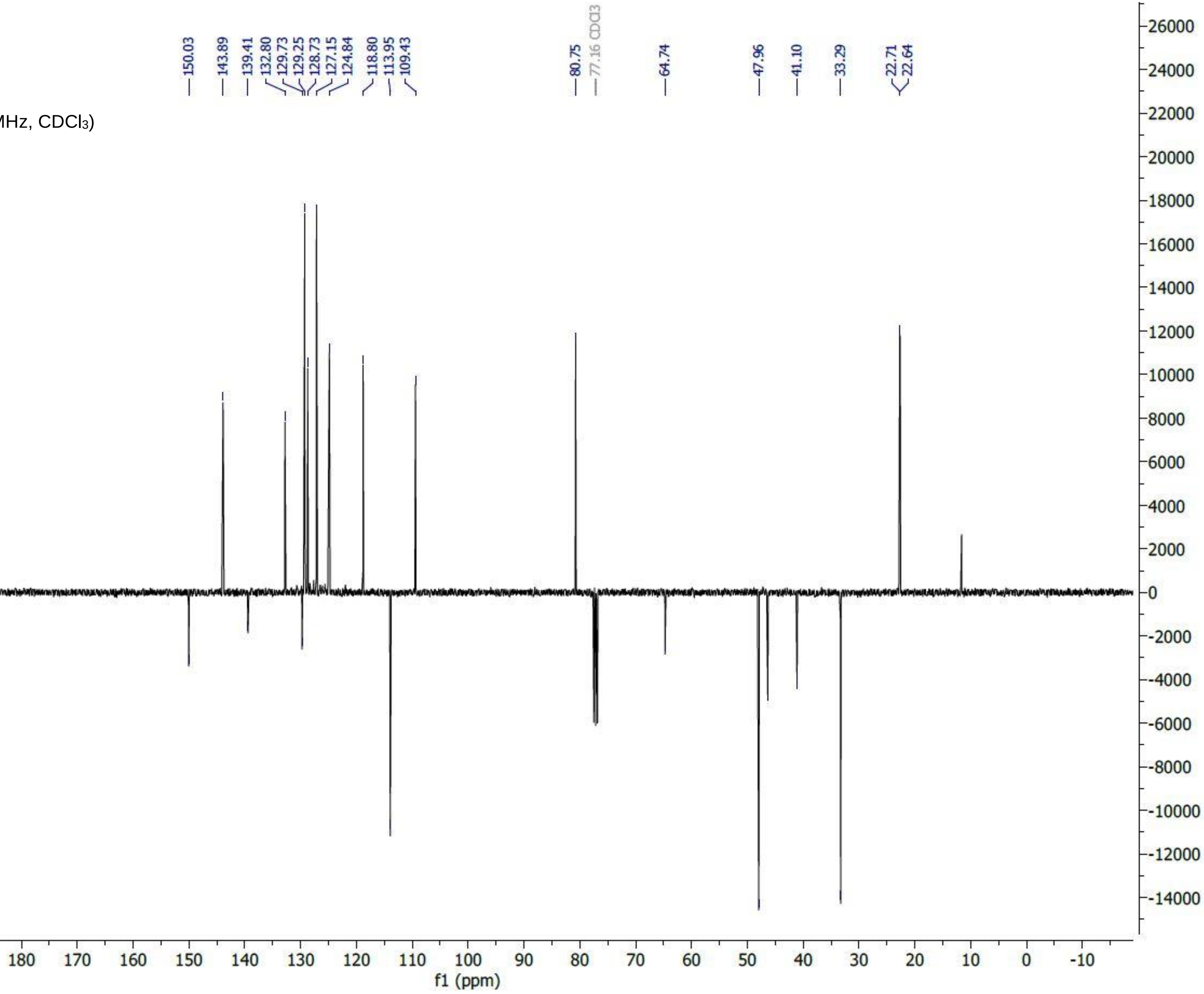

^1H-NMR (400 MHz, $CDCl_3$)

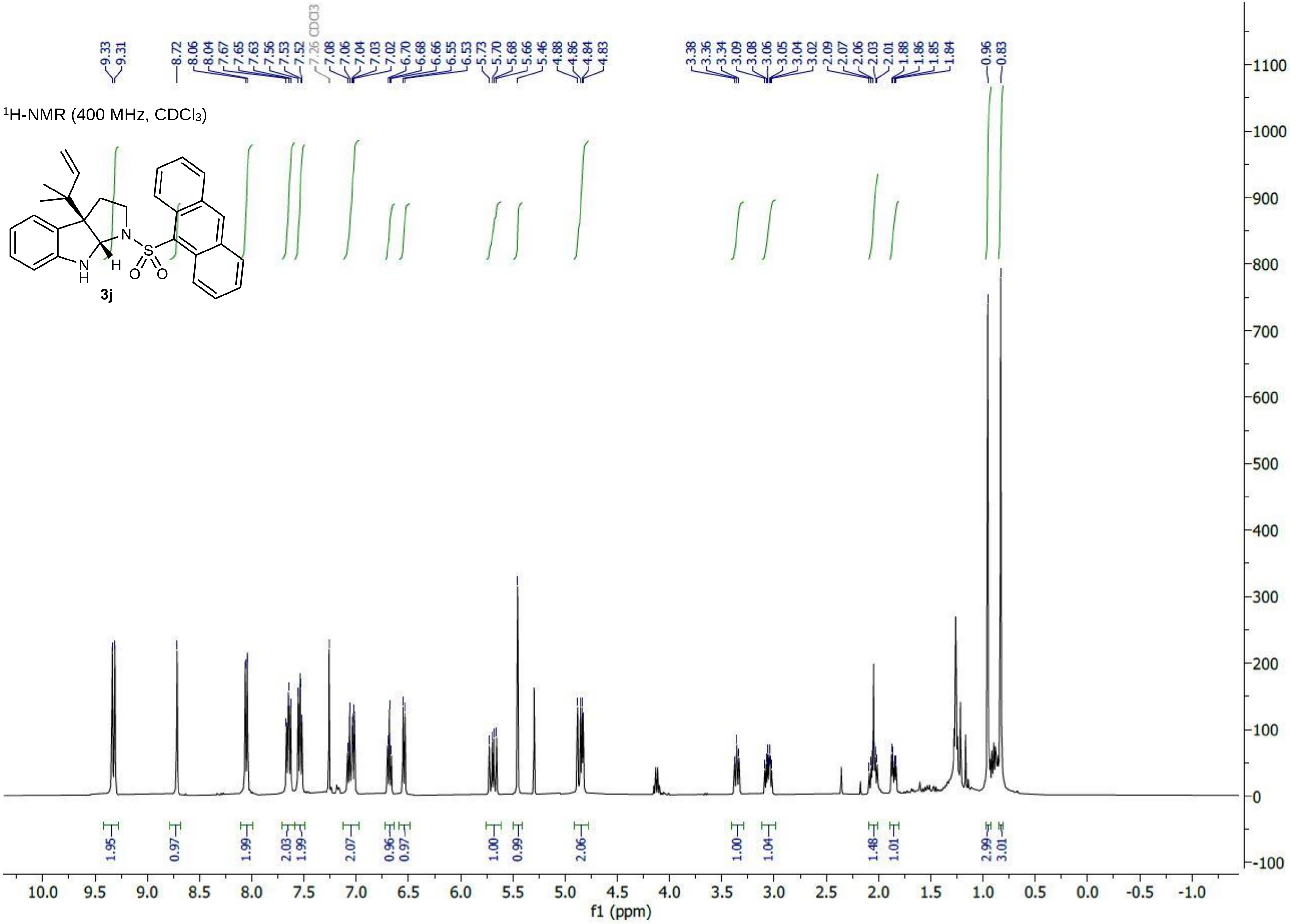

^{13}C-DEPTQ-NMR (100 MHz, $CDCl_3$)

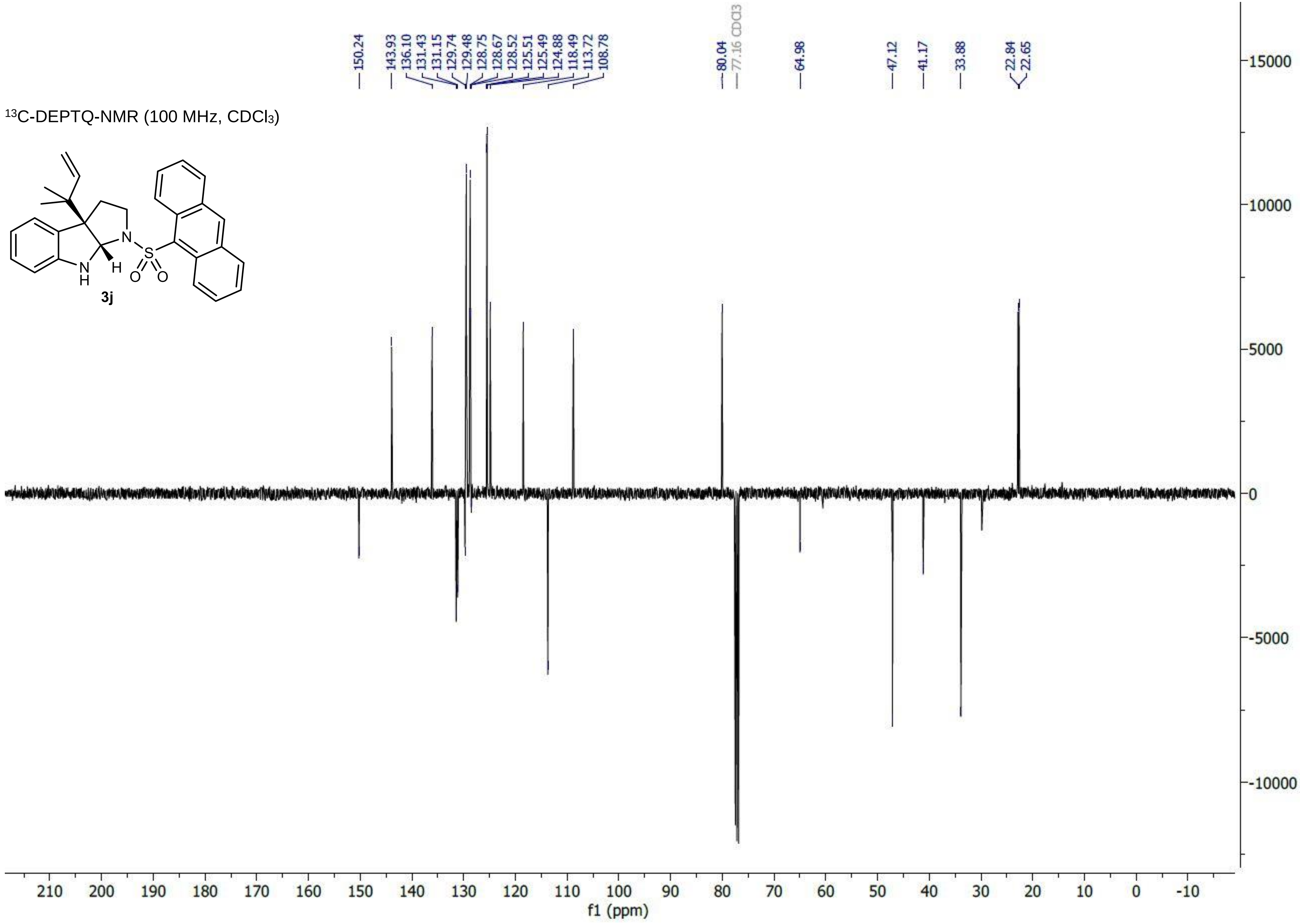

^{1}H-NMR (400 MHz, $CDCl_3$)

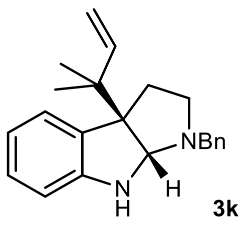

3k

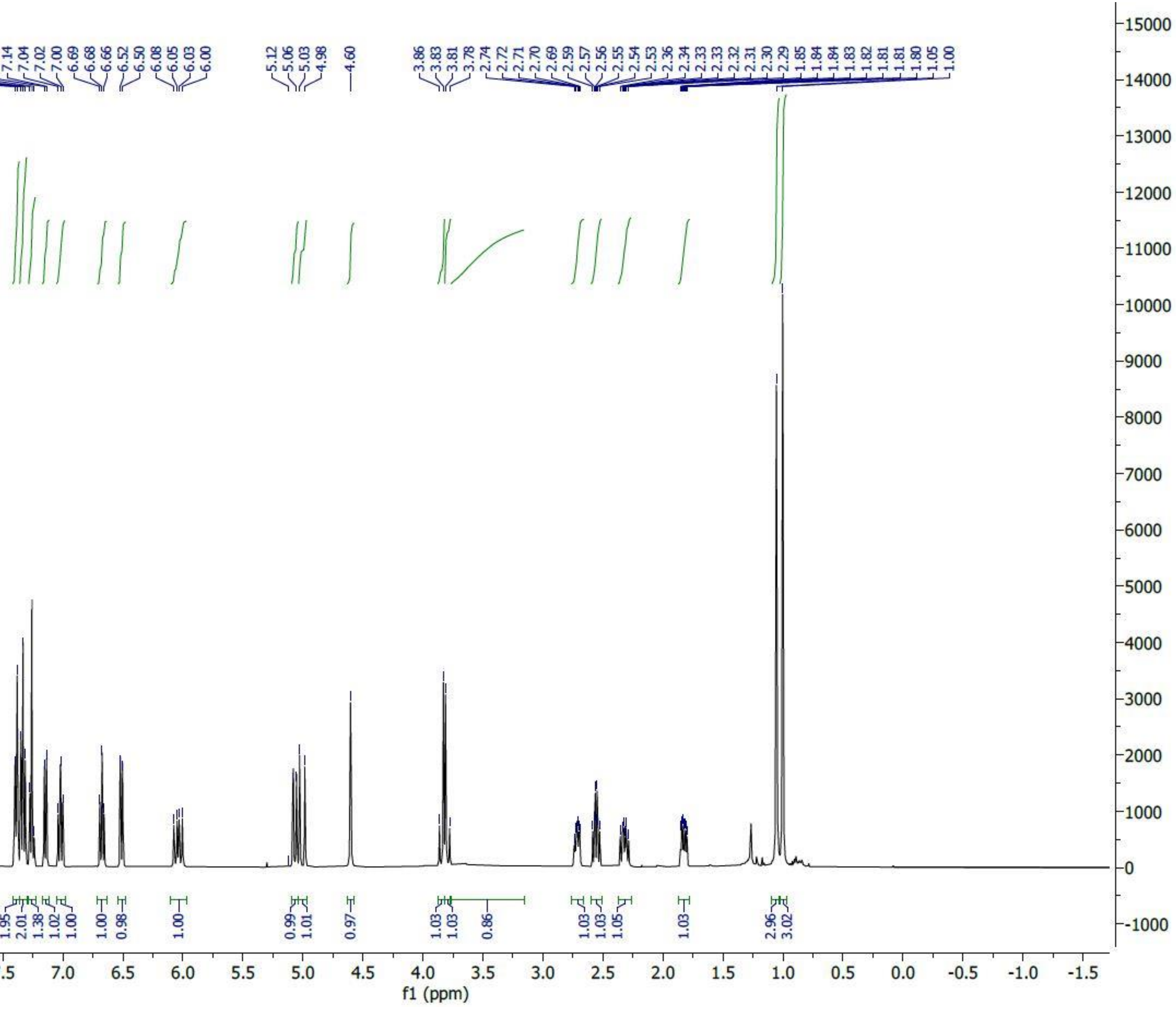

^{13}C-DEPTQ-NMR (100 MHz, $CDCl_3$)

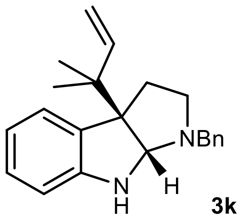

3k

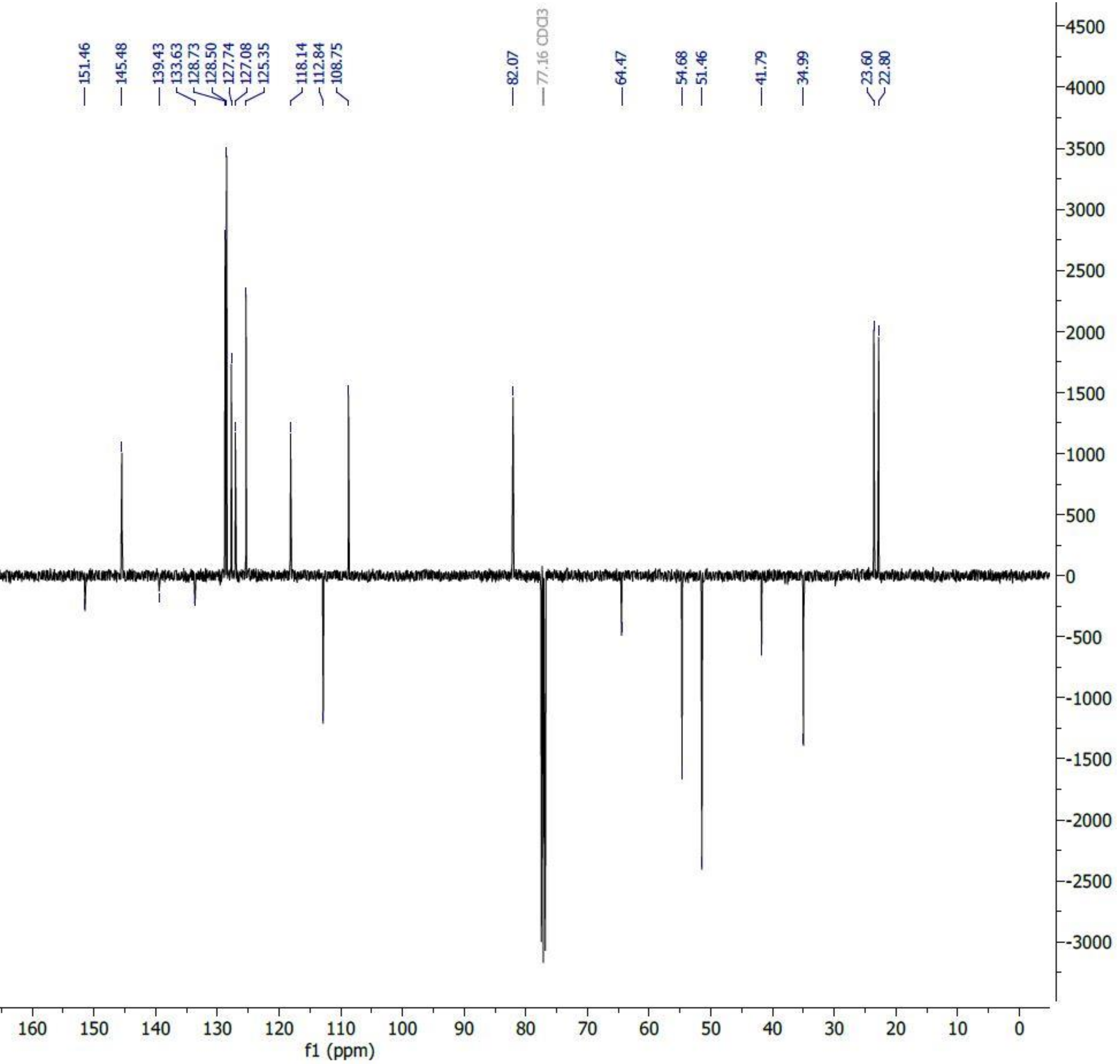

^{1}H-NMR (400 MHz, $CDCl_3$)

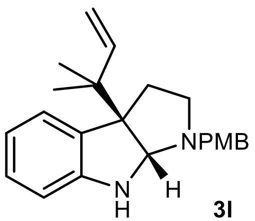

3l

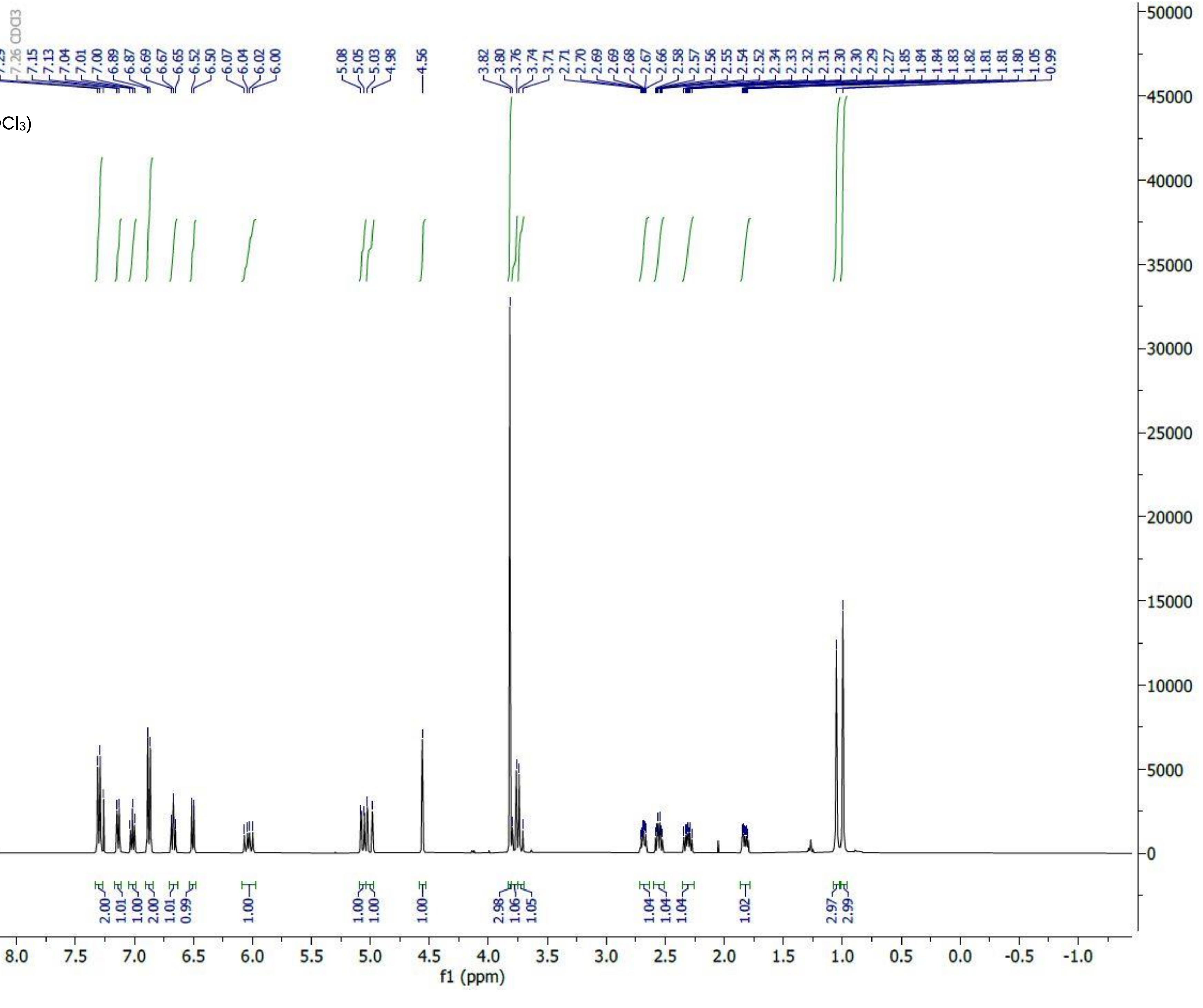

^{13}C-DEPTQ-NMR (100 MHz, $CDCl_3$)

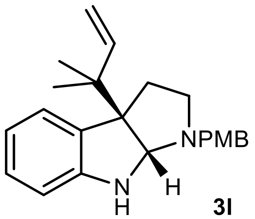

3l

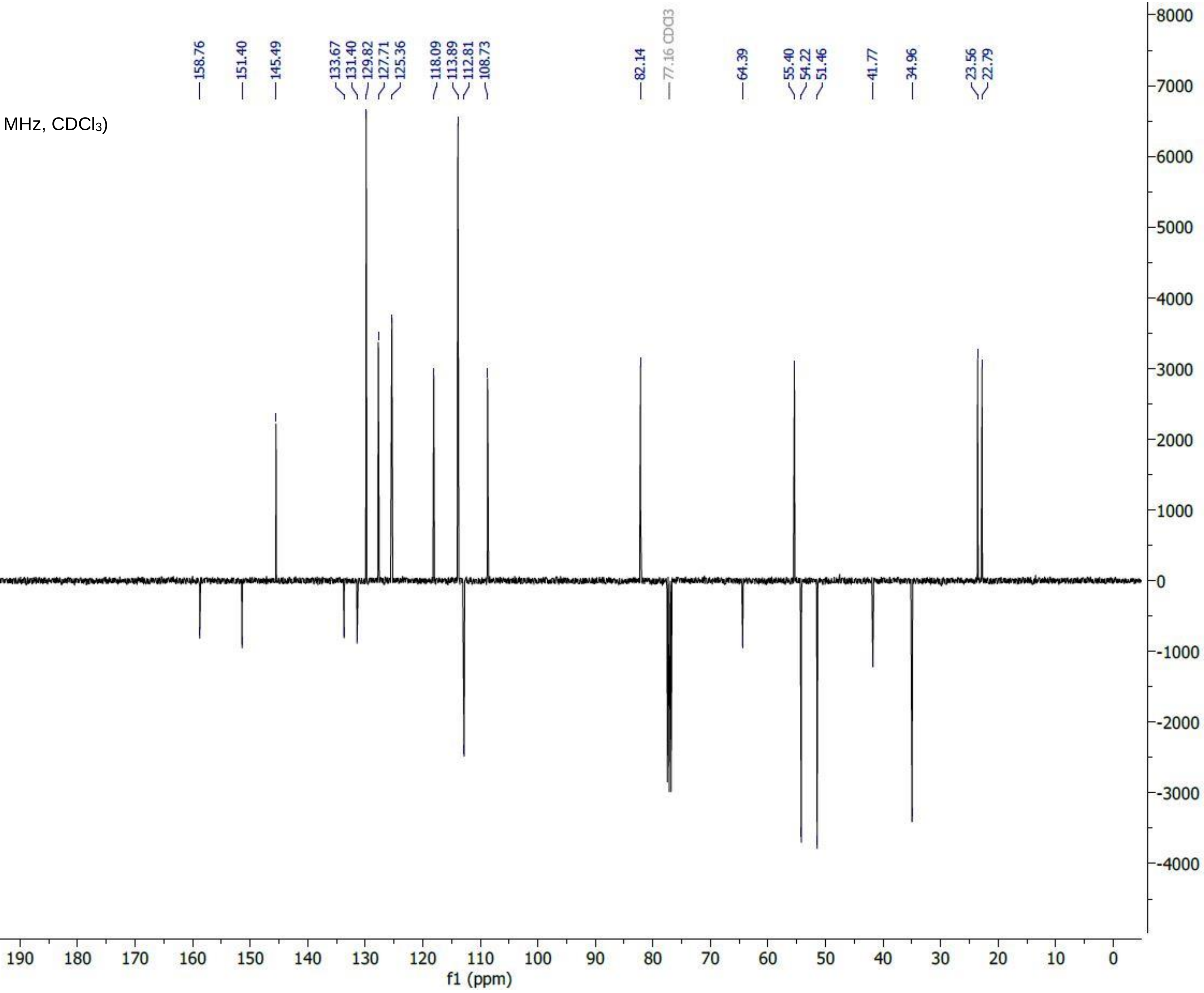

^{1}H-NMR (400 MHz, $CDCl_3$)

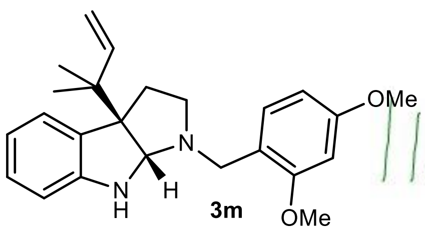

3m

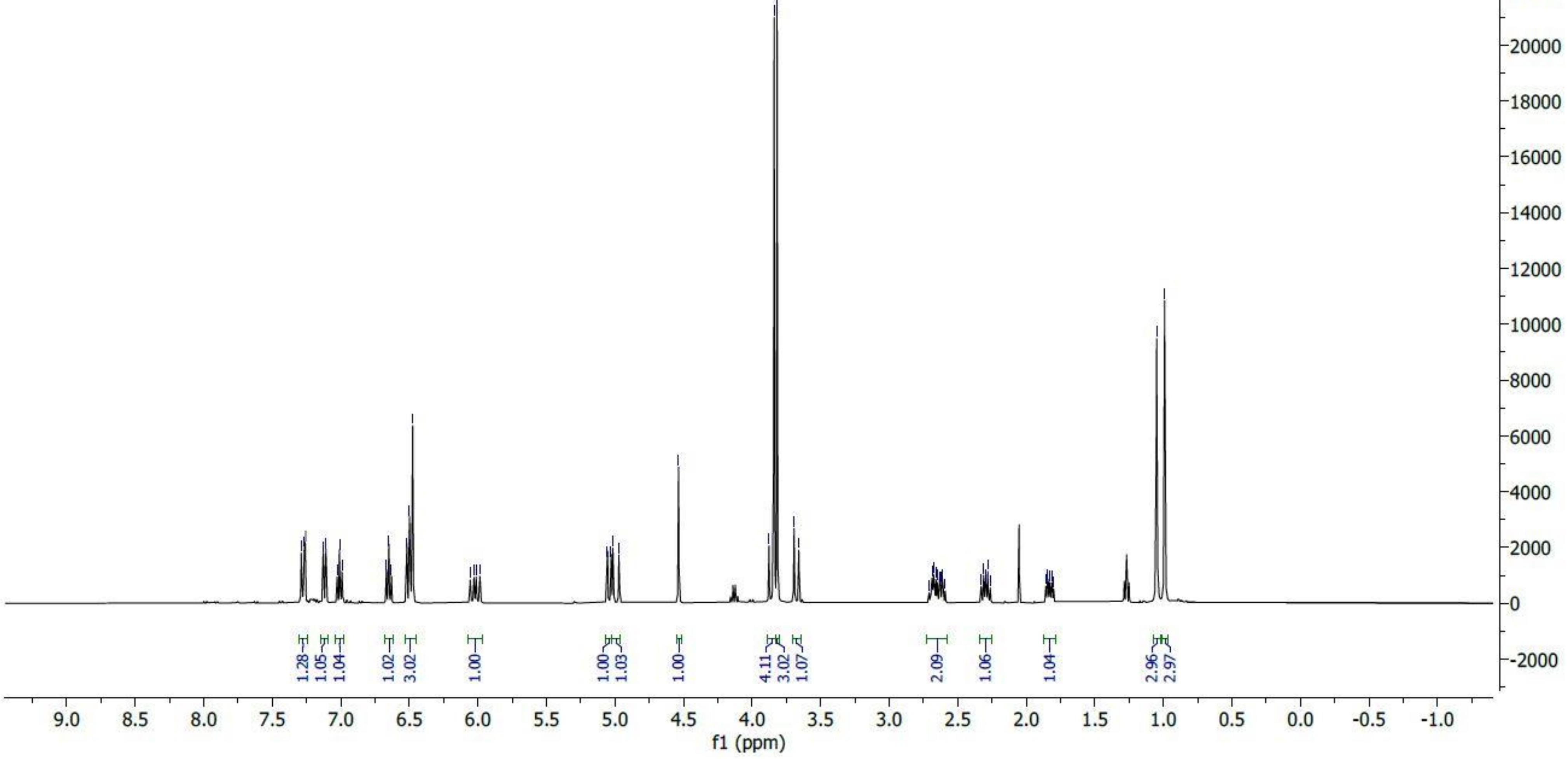

^{13}C-DEPTQ-NMR (100 MHz, $CDCl_3$)

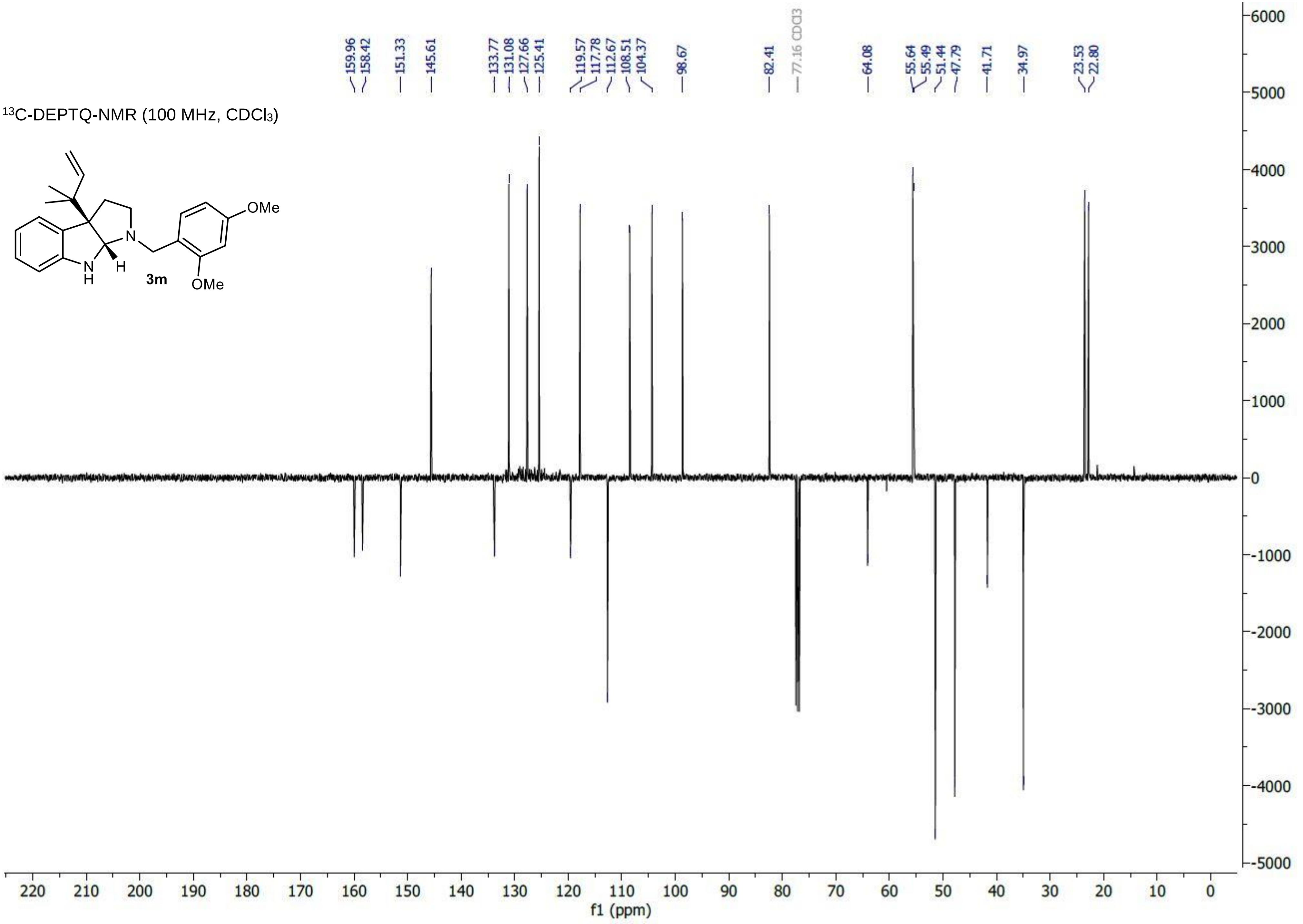

3m

^{1}H-NMR (400 MHz, $CDCl_3$)

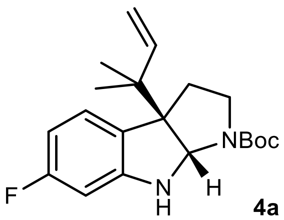

4a

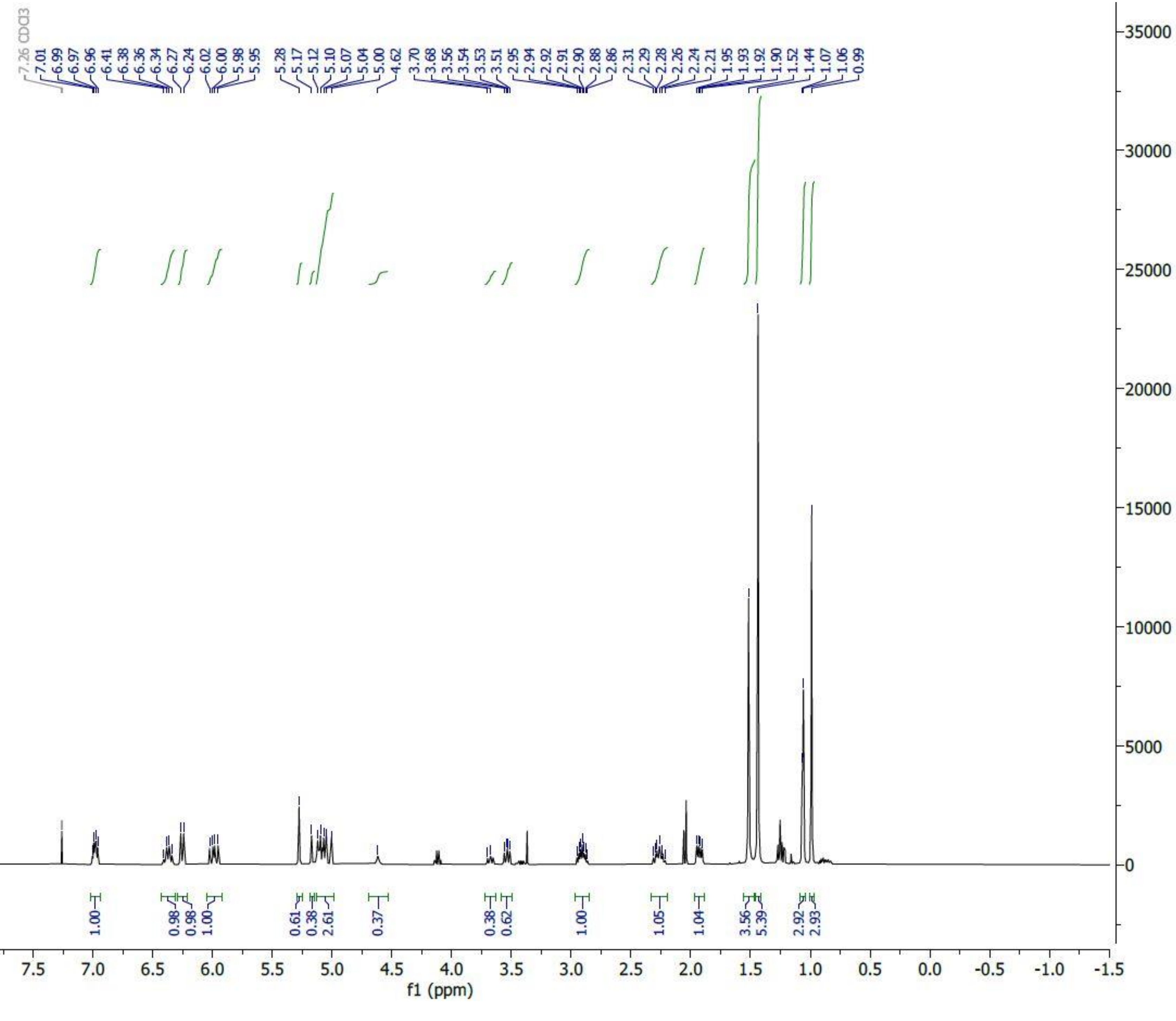

^{13}C-DEPTQ-NMR (100 MHz, $CDCl_3$)

4a

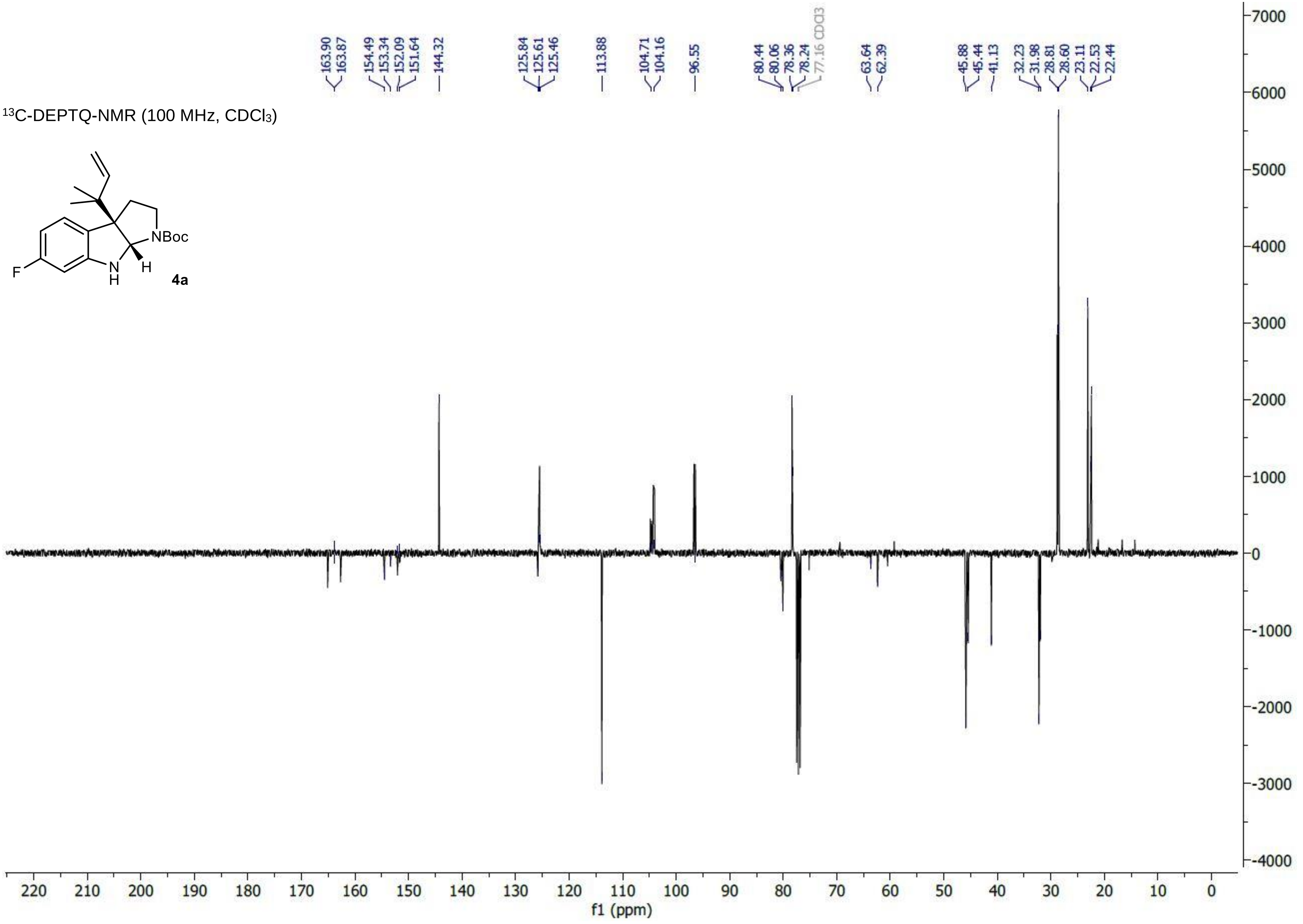

^{19}F-NMR (565 MHz, $CDCl_3$)

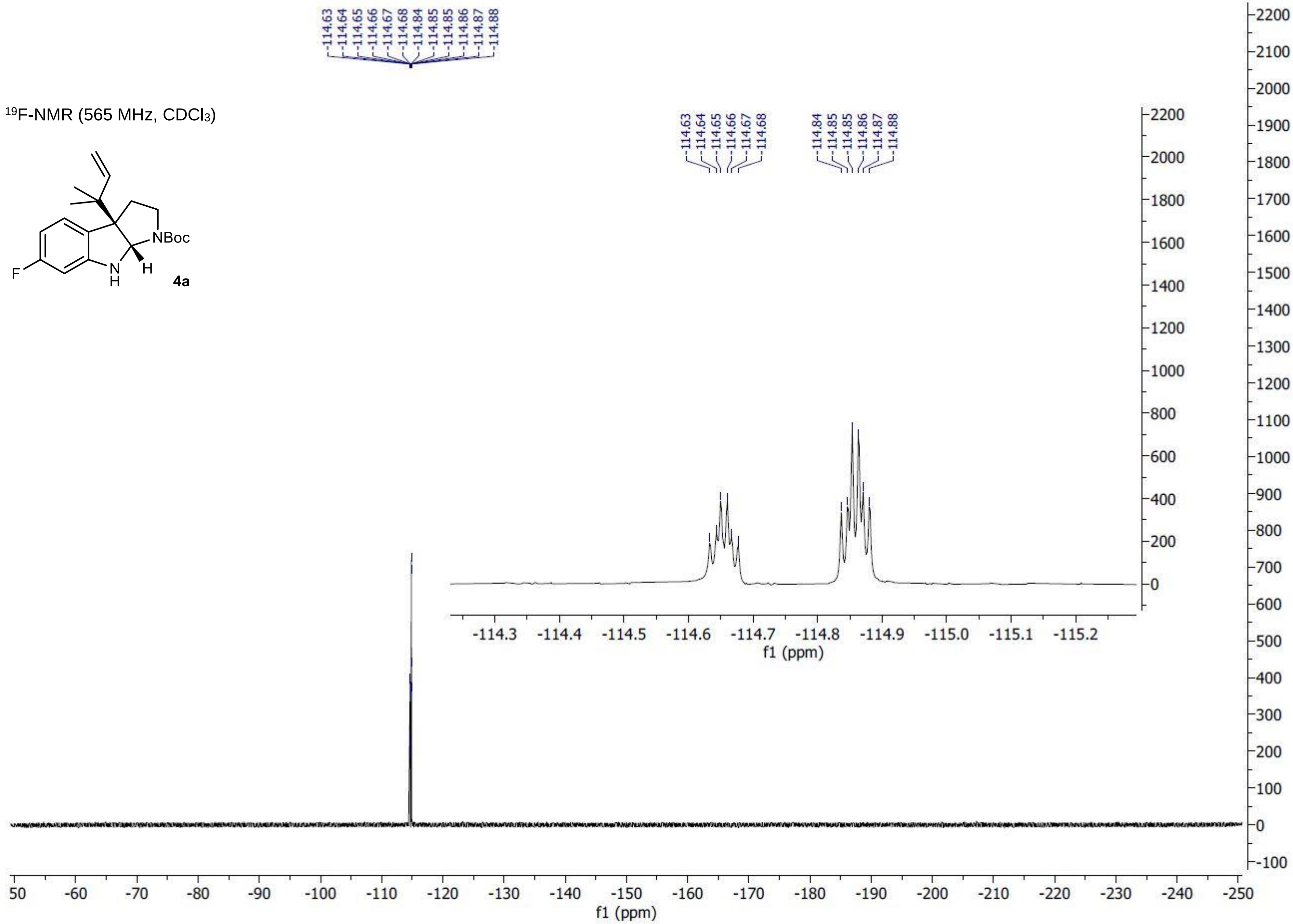

4a

^{1}H-NMR (400 MHz, $CDCl_3$)

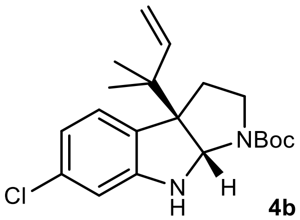

4b

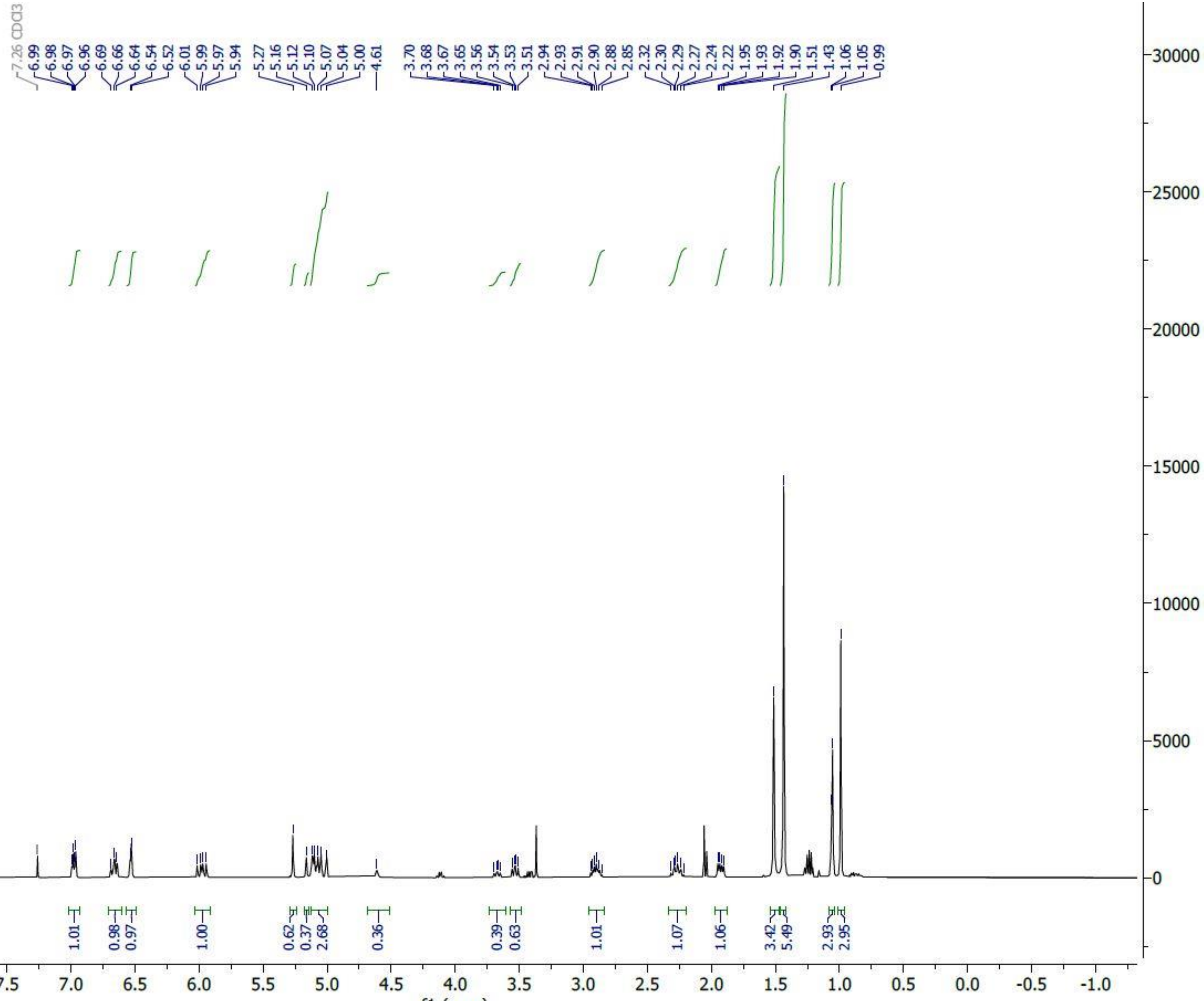

^{13}C-DEPTQ-NMR (100 MHz, $CDCl_3$)

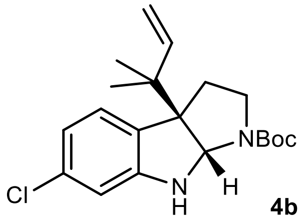

4b

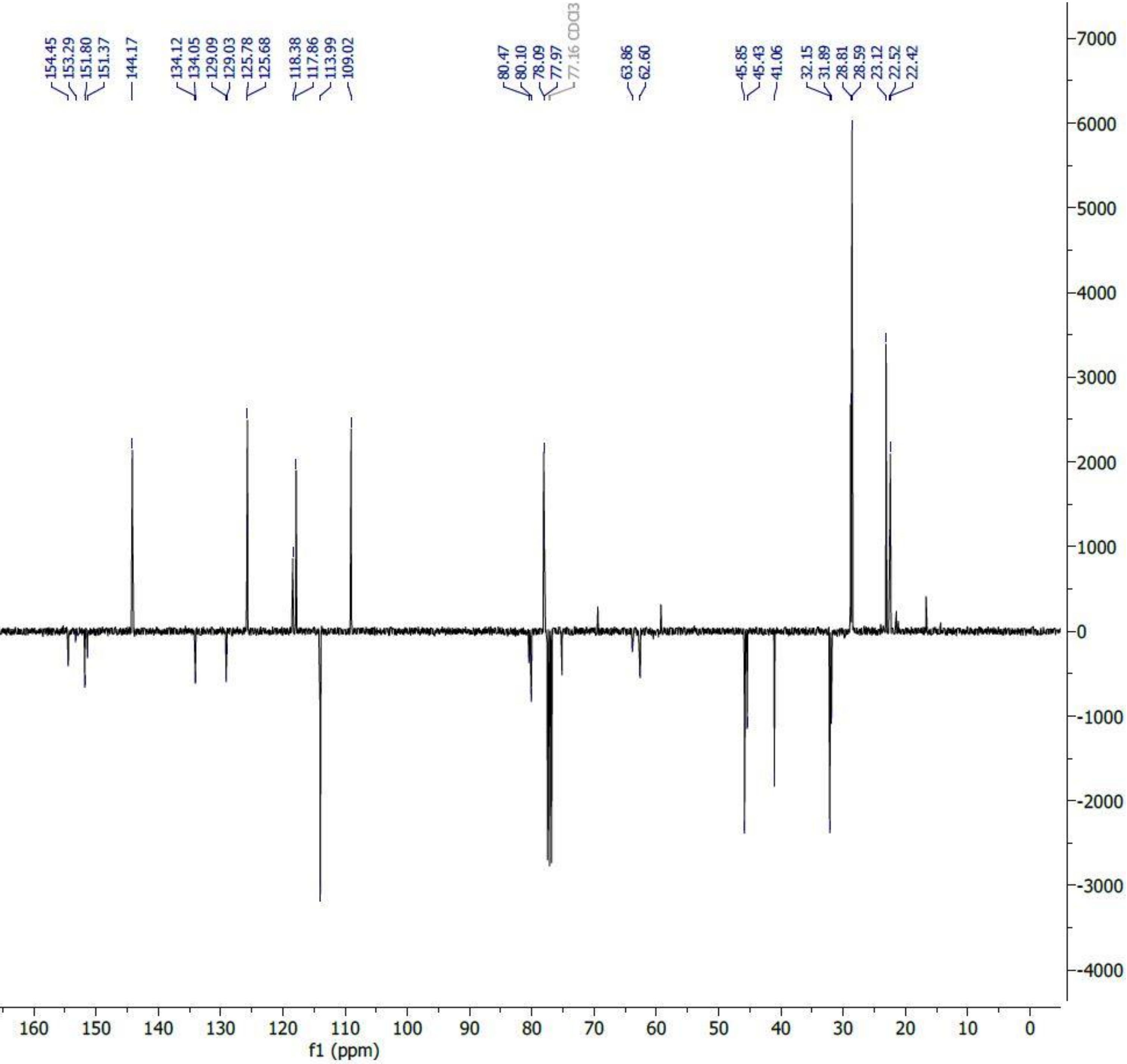

^{1}H-NMR (500 MHz, $CDCl_3$)

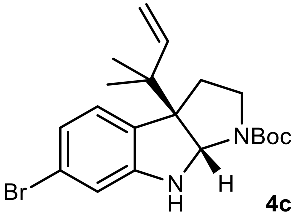

4c

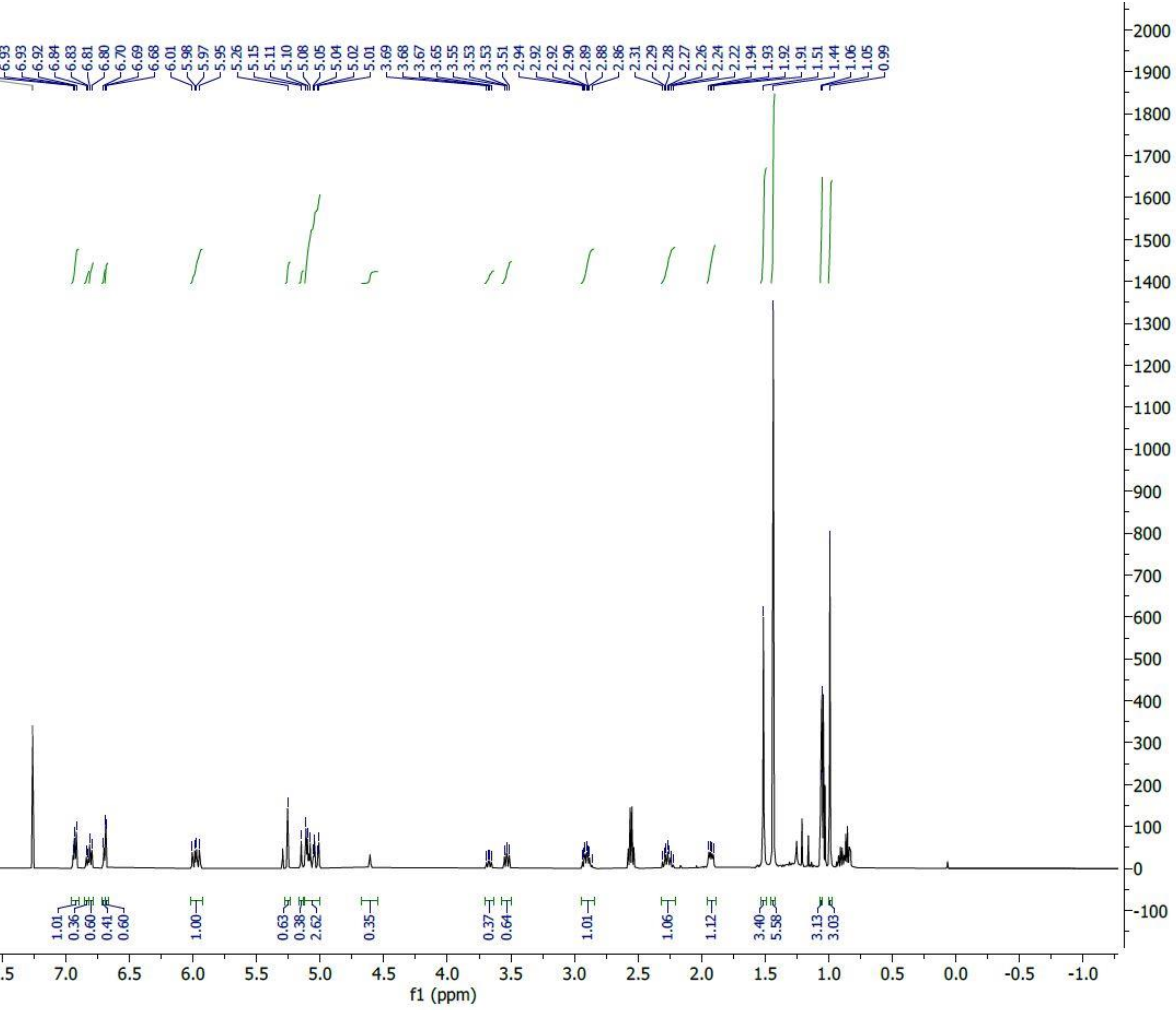

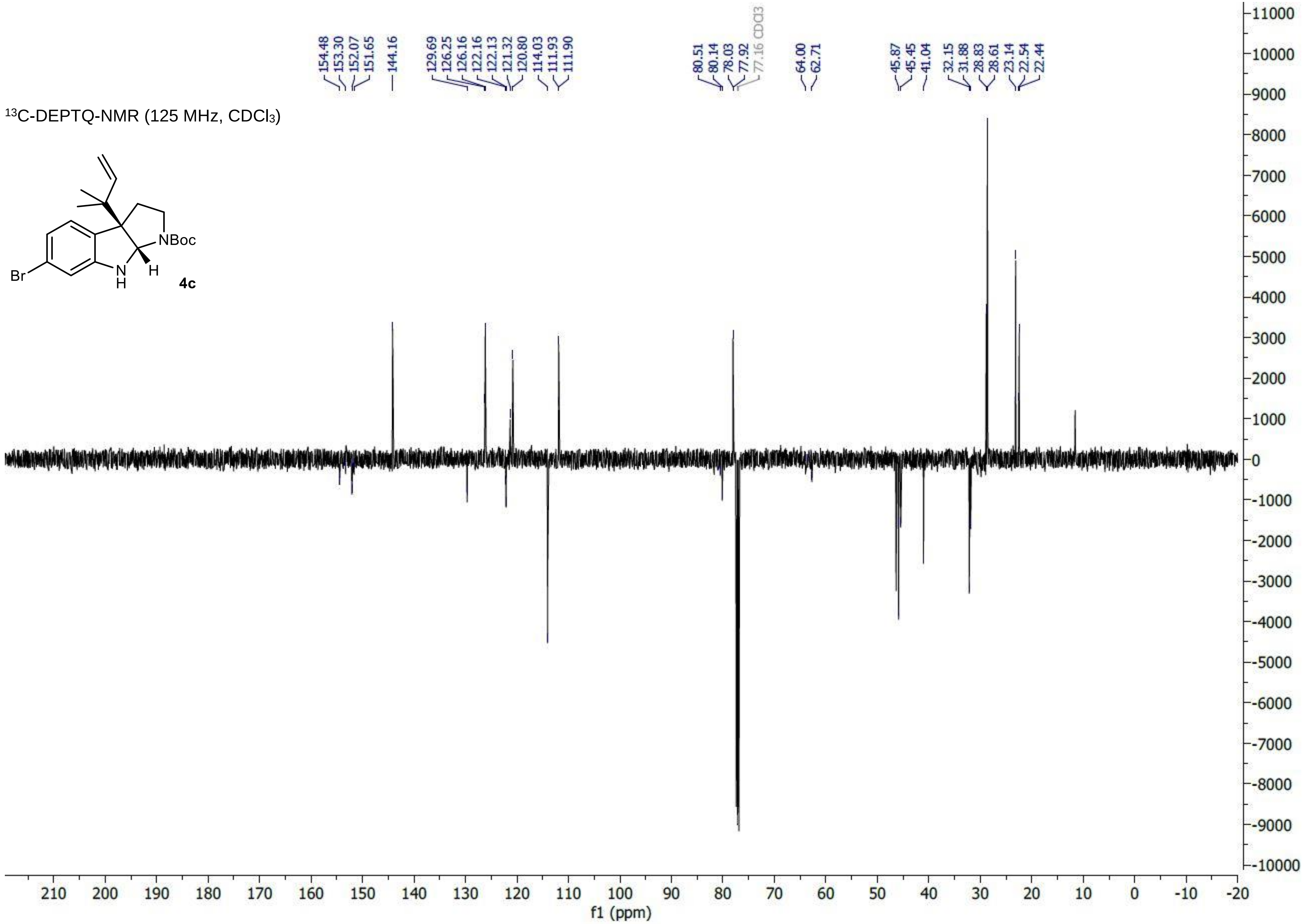

13C-DEPTQ-NMR (125 MHz, CDCl3)
NBoc
Br
N
H
H
4c
154.48
153.30
152.07
151.65
144.16
129.69
126.25
126.16
122.16
122.13
121.32
120.80
114.03
111.93
111.90
80.51
80.14
78.03
77.92
77.16 CDCl3
64.00
62.71
45.87
45.45
41.04
32.15
31.88
28.83
28.61
23.14
22.54
22.44
f1 (ppm)

^{1}H-NMR (400 MHz, $CDCl_3$)

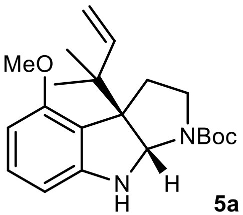

5a

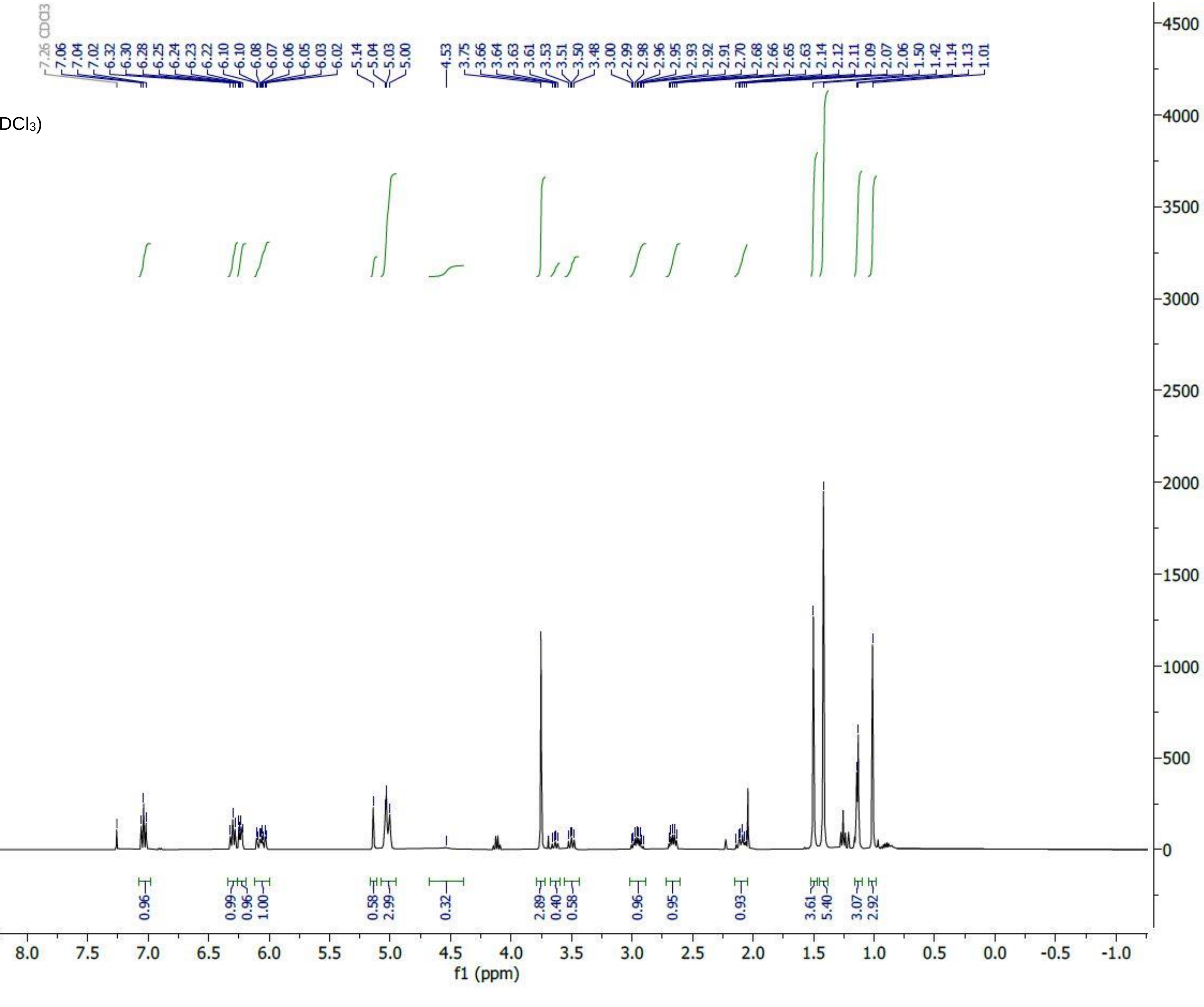

^{13}C-DEPTQ-NMR (100 MHz, $CDCl_3$)

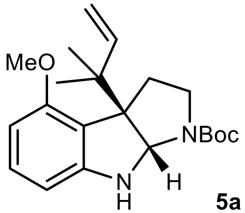

5a

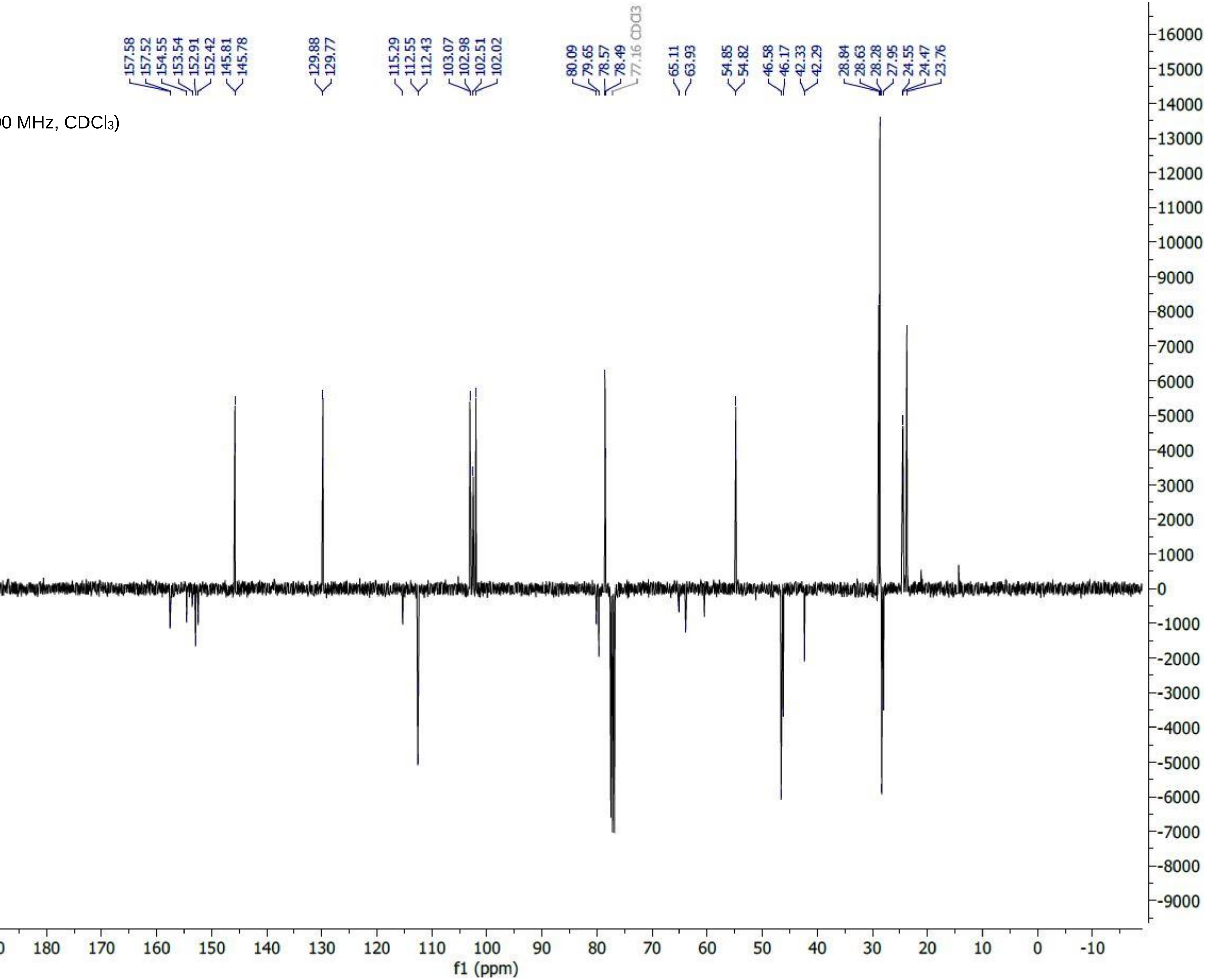

^{1}H-NMR (500 MHz, $CDCl_3$)

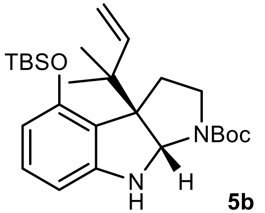

5b

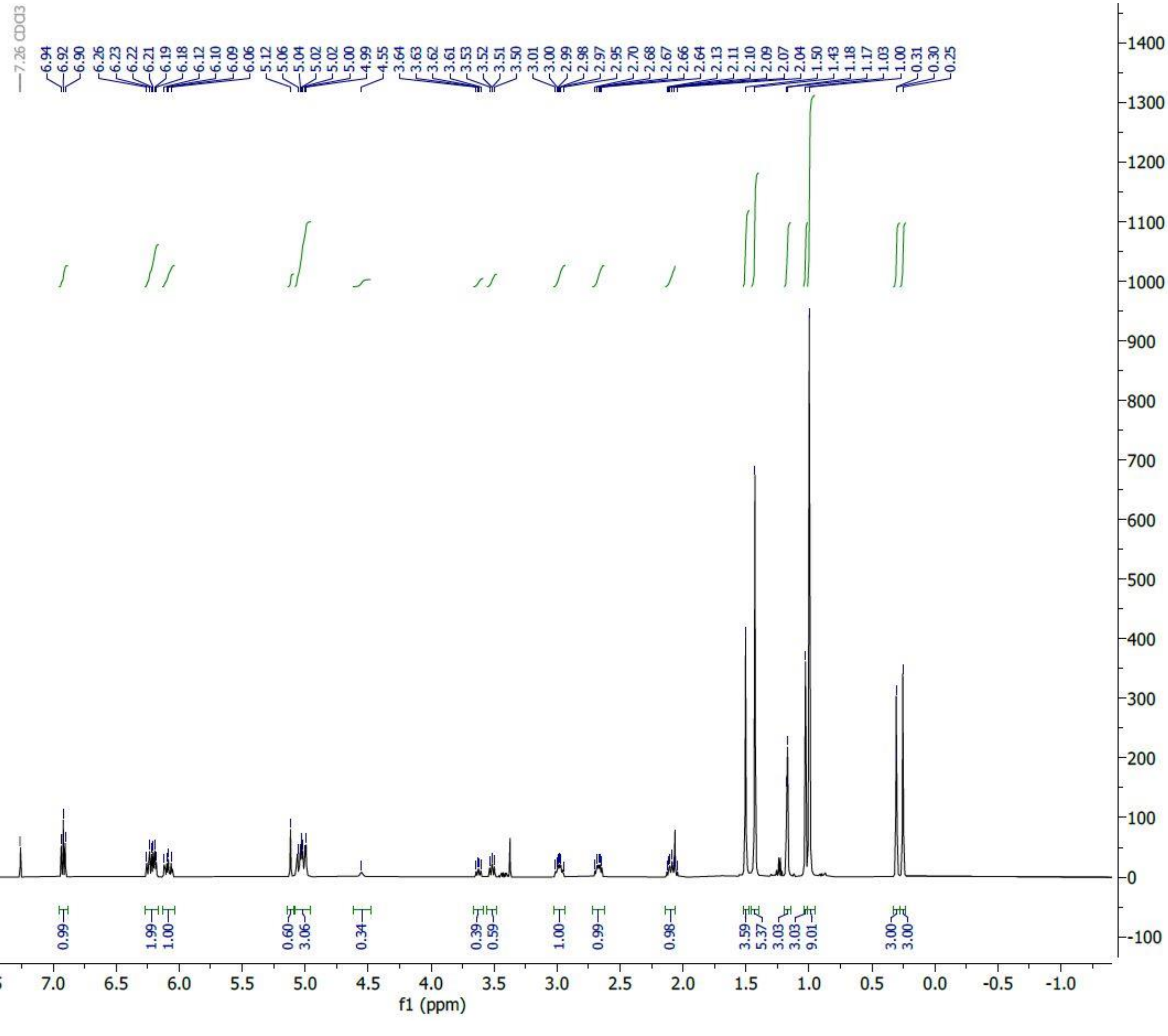

^{13}C-DEPTQ-NMR (125 MHz, $CDCl_3$)

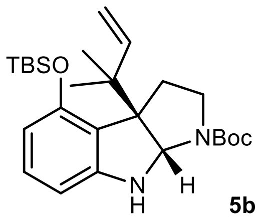

5b

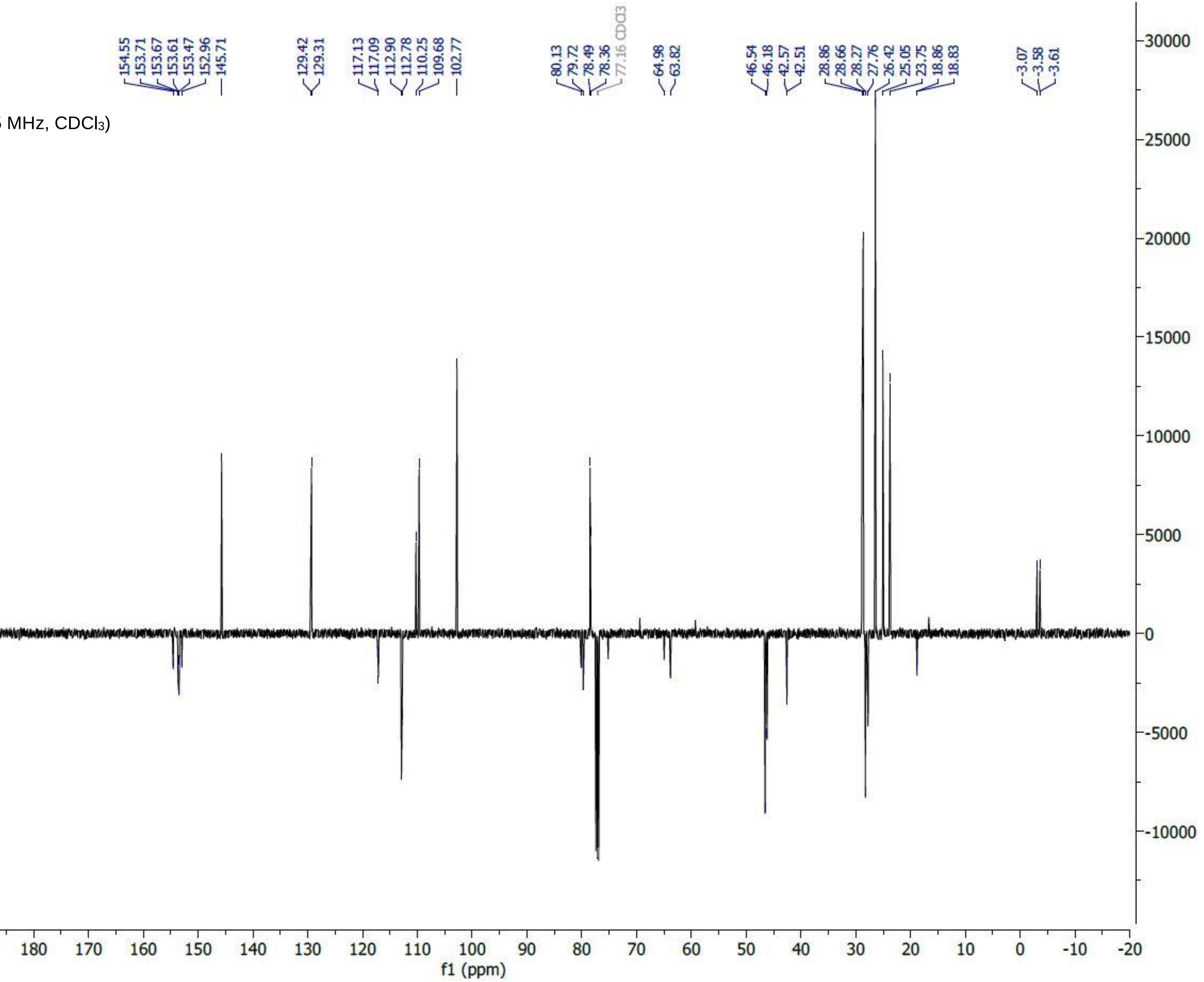

^{1}H-NMR (500 MHz, $CDCl_3$)

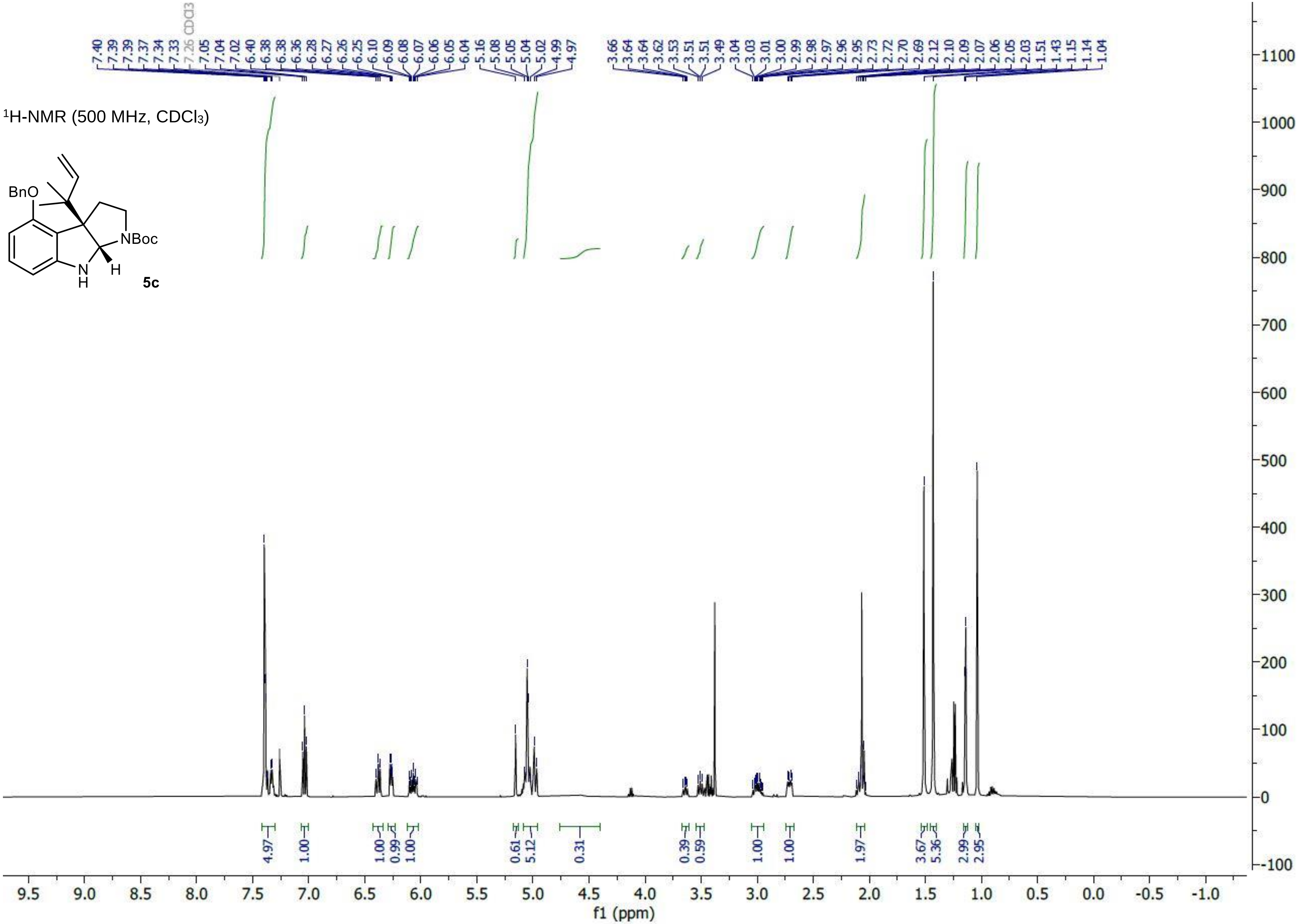

^{13}C-DEPTQ-NMR (125 MHz, $CDCl_3$)

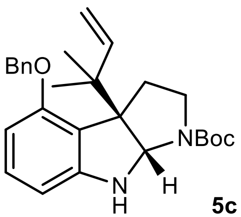

5c

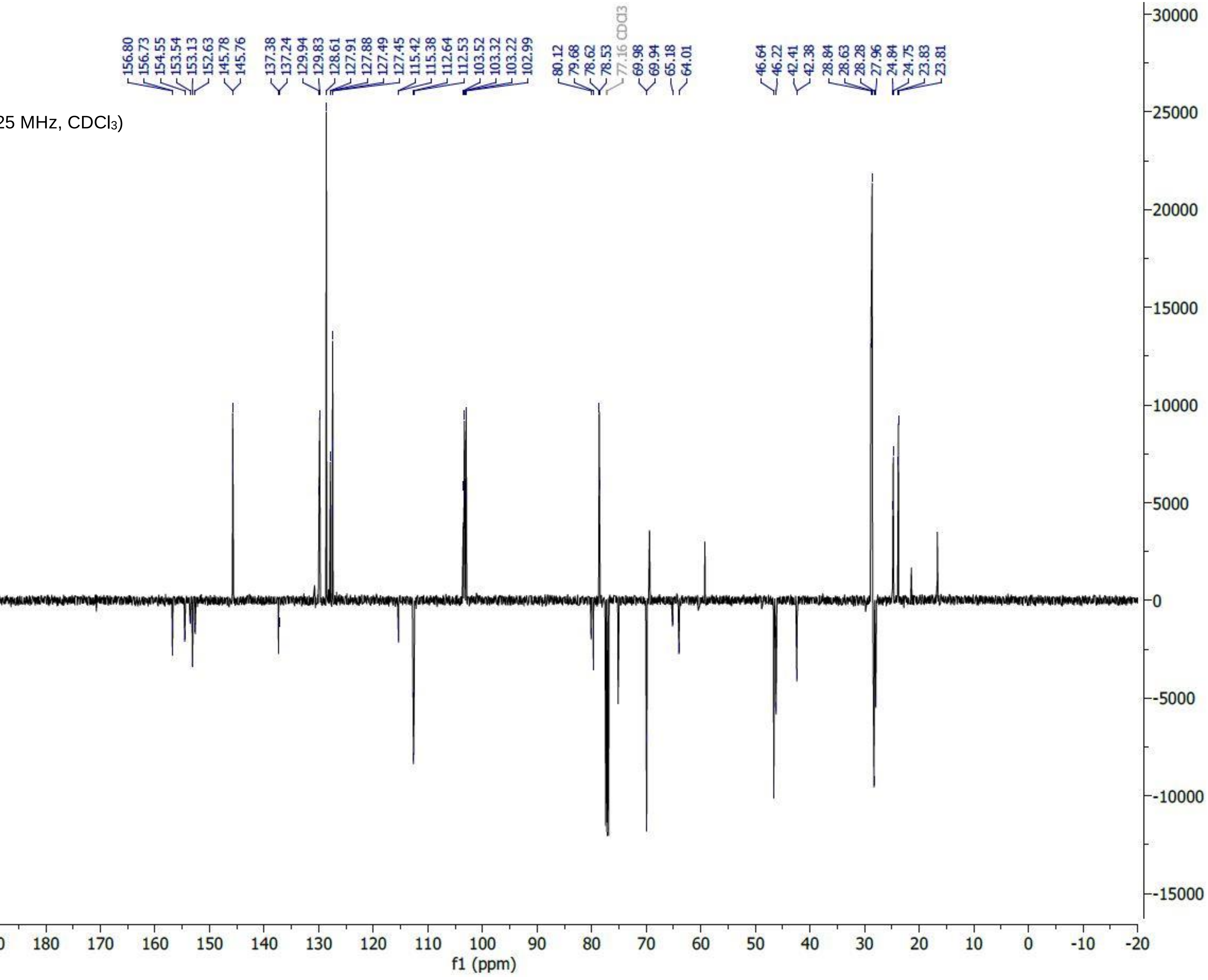

¹H-NMR (400 MHz, CDCl₃)

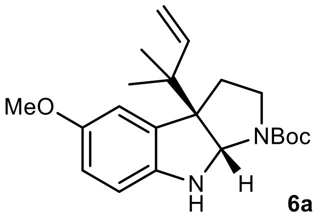

6a

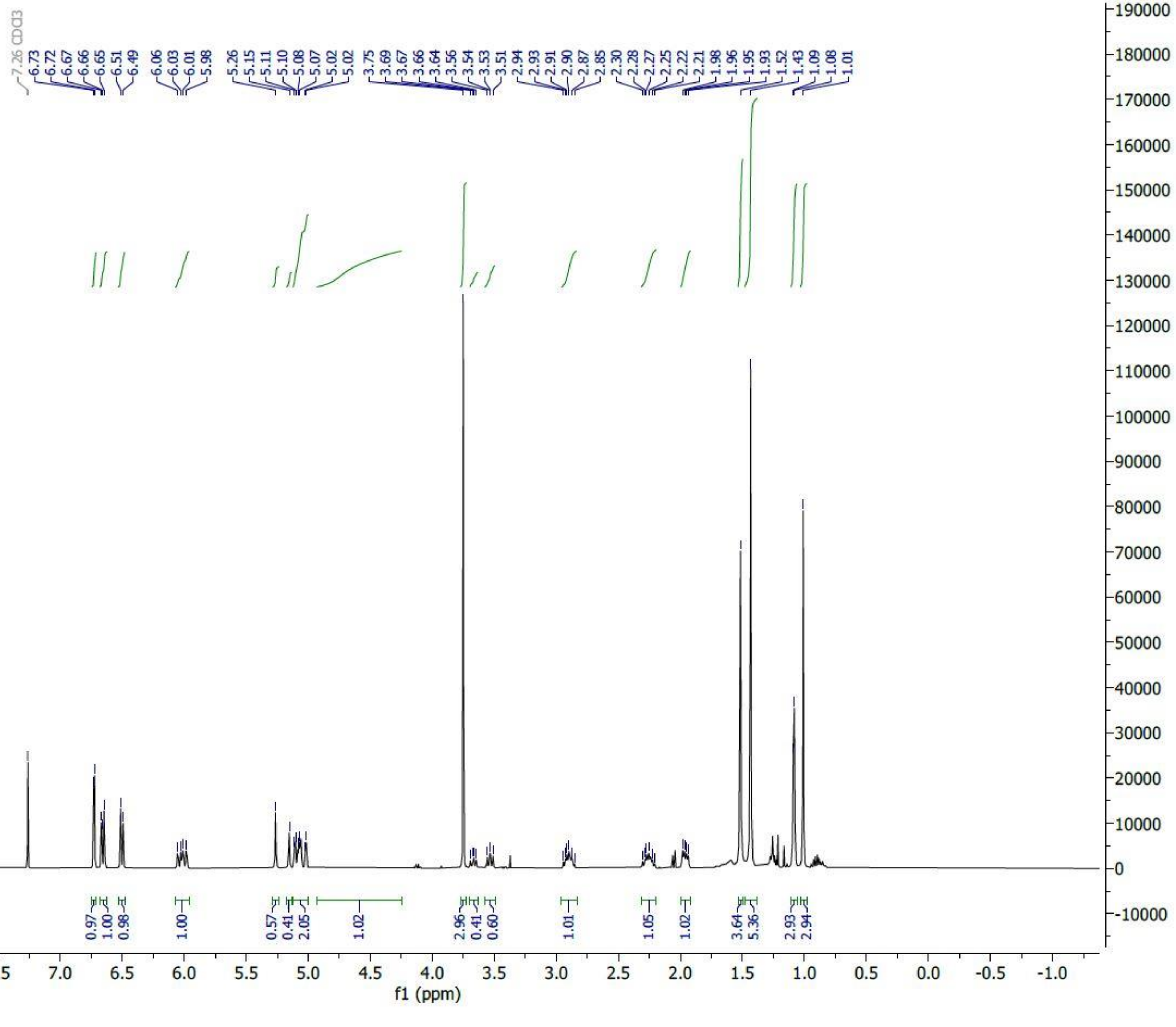

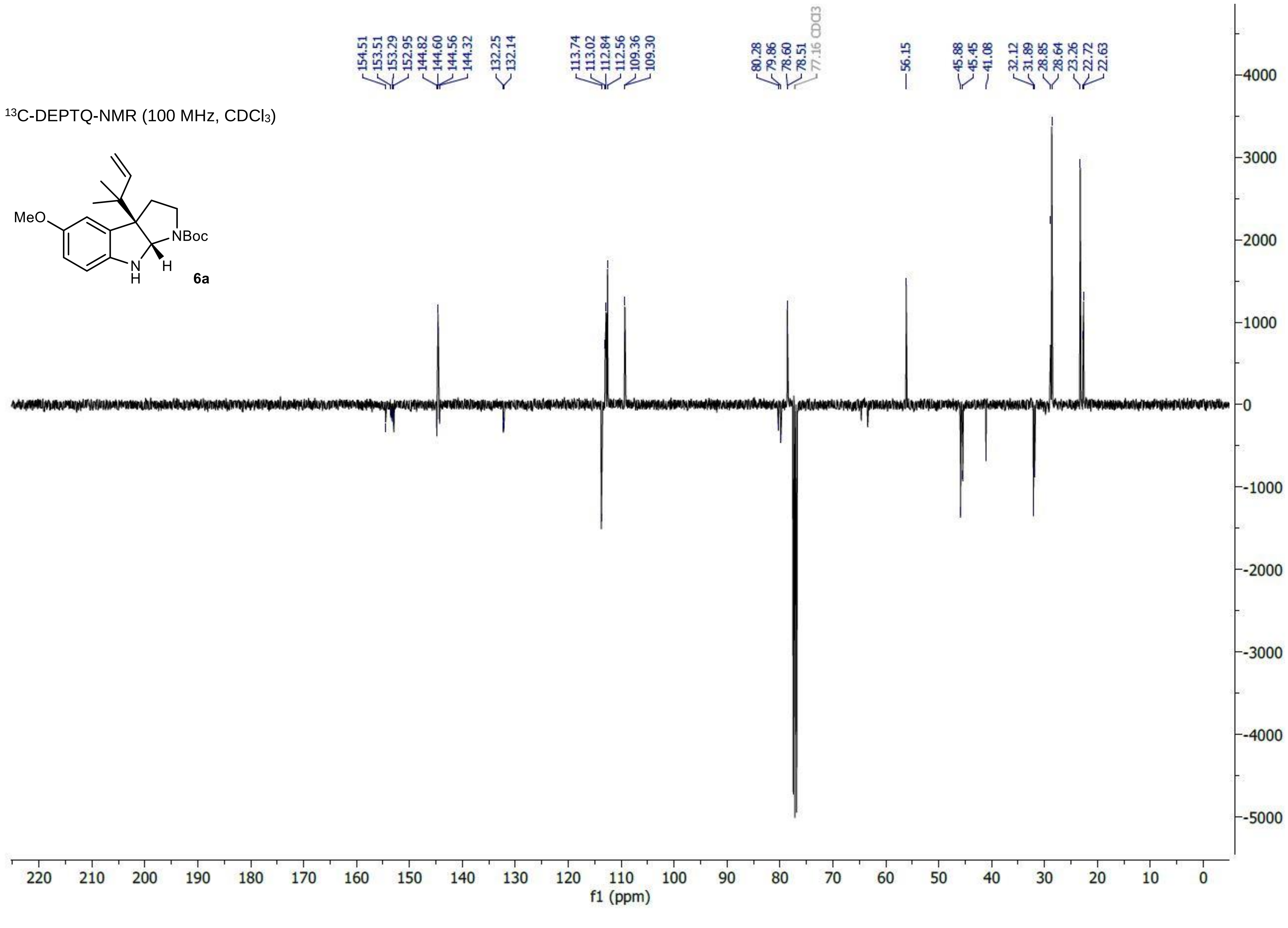
13C-DEPTQ-NMR (100 MHz, CDCl3)
MeO
NBoc
N
H
H
6a
154.51
153.51
153.29
152.95
144.82
144.60
144.56
144.32
132.25
132.14
113.74
113.02
112.84
112.56
109.36
109.30
80.28
79.86
78.60
78.51
77.16 CDCl3
56.15
45.88
45.45
41.08
32.12
31.89
28.85
28.64
23.26
22.72
22.63
f1 (ppm)

^{1}H-NMR (500 MHz, $CDCl_3$)

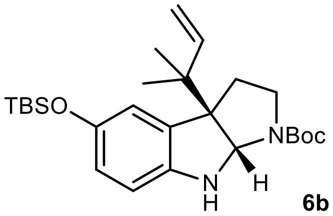

6b

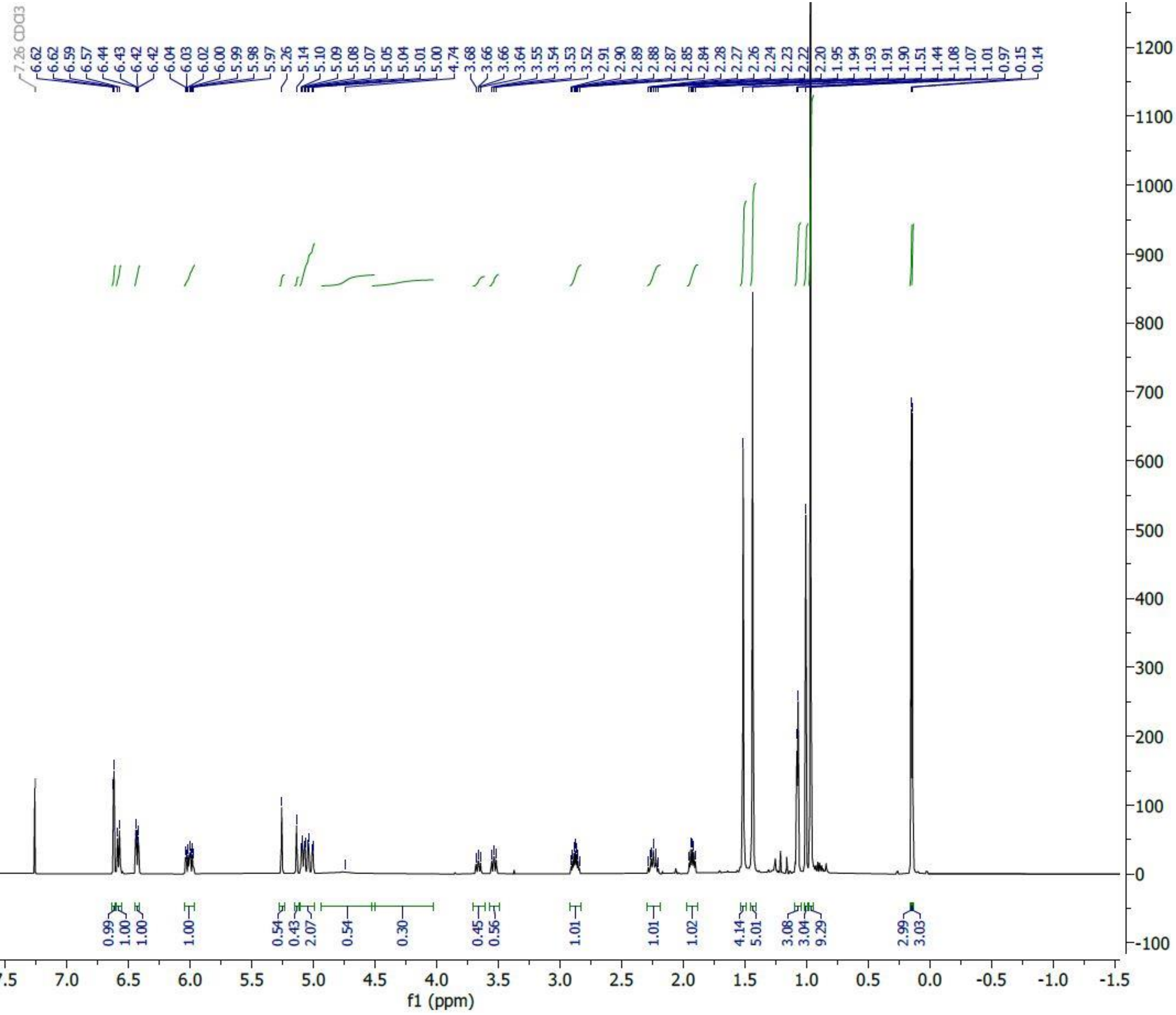

^{13}C-DEPTQ-NMR (125 MHz, $CDCl_3$)

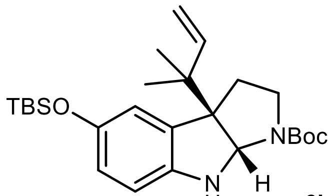

6b

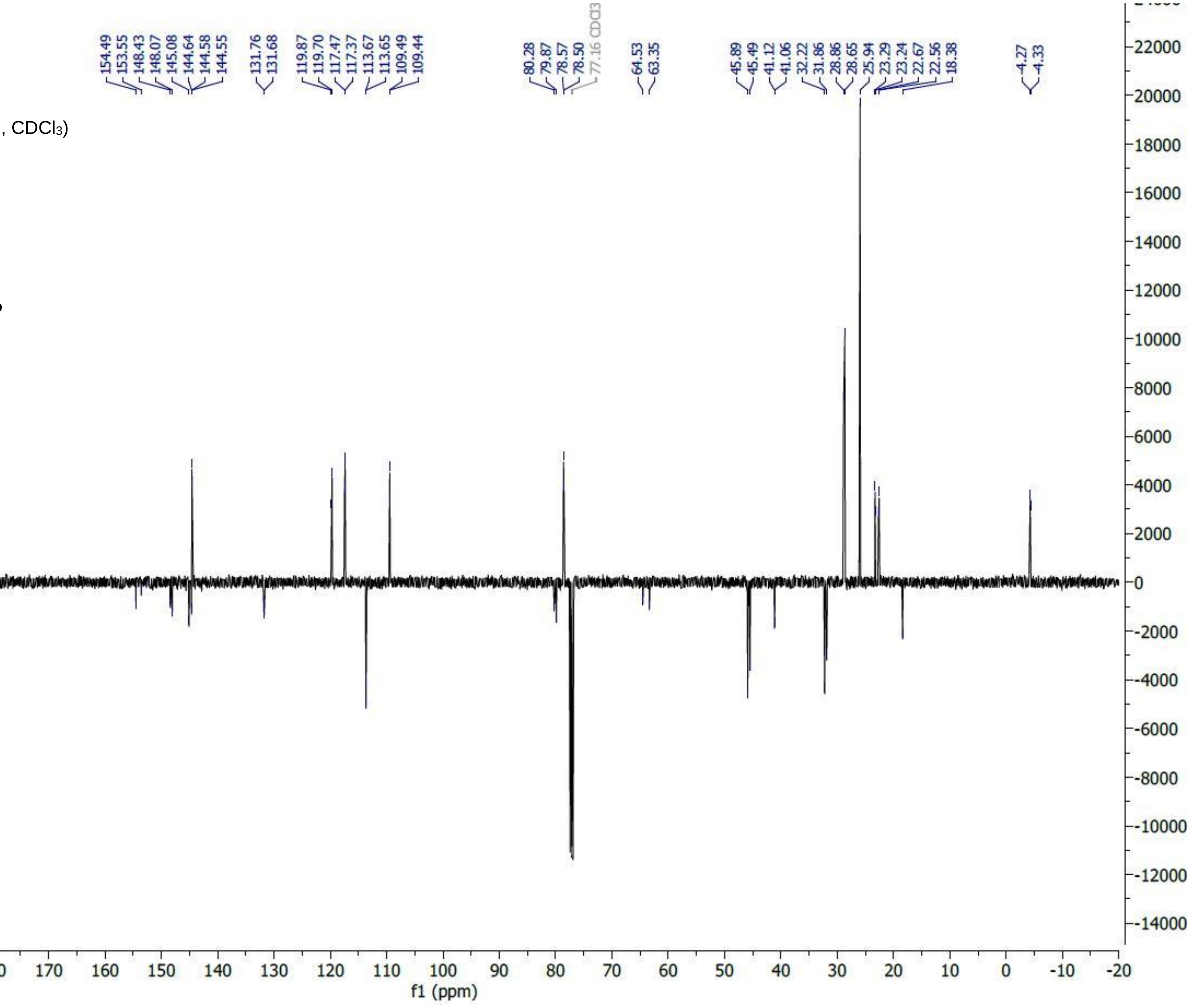

^{1}H-NMR (500 MHz, CDCl3)

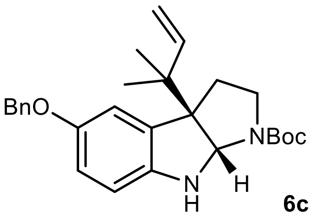

6c

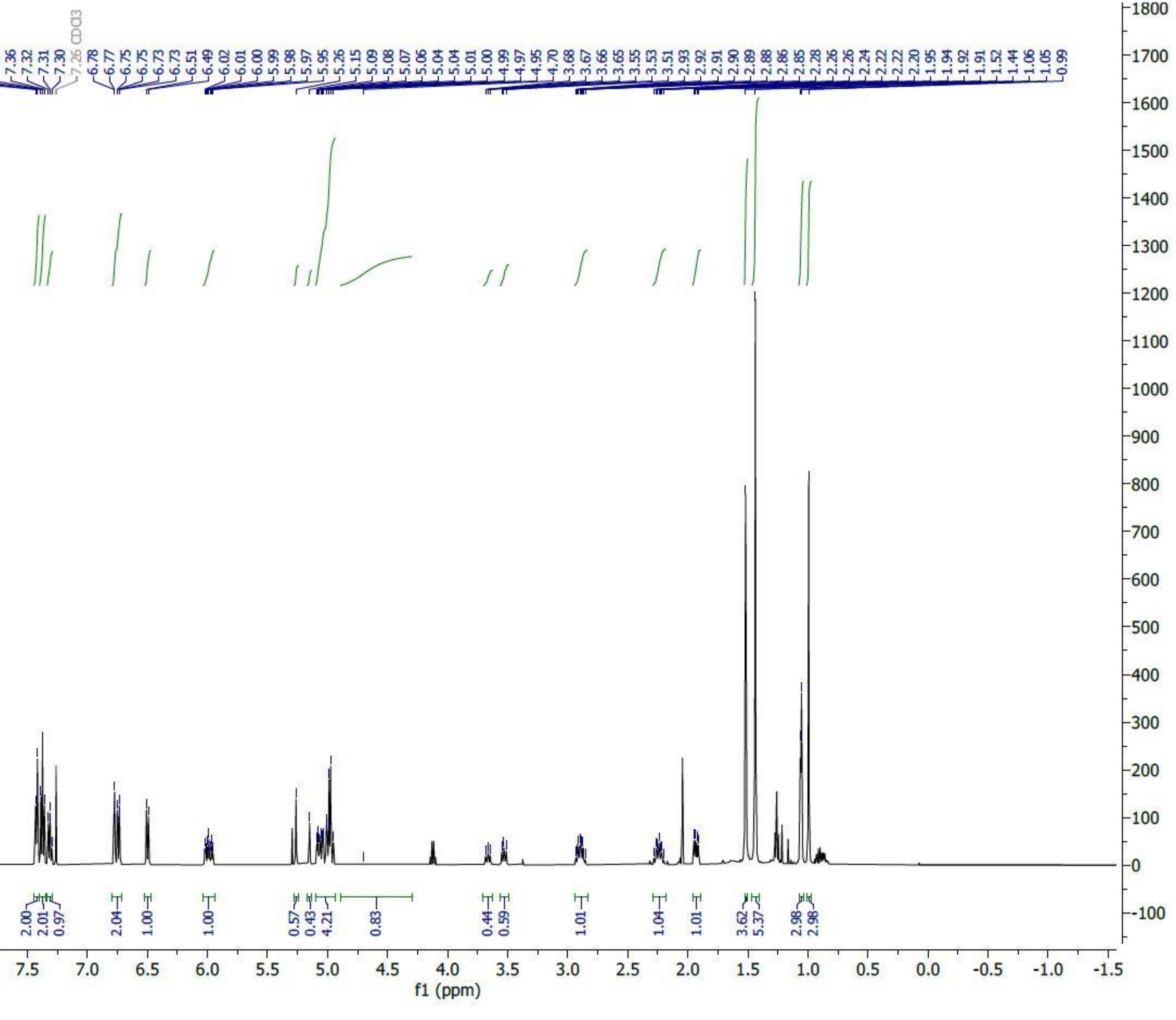

^{13}C-DEPTQ-NMR (125 MHz, $CDCl_3$)

6c

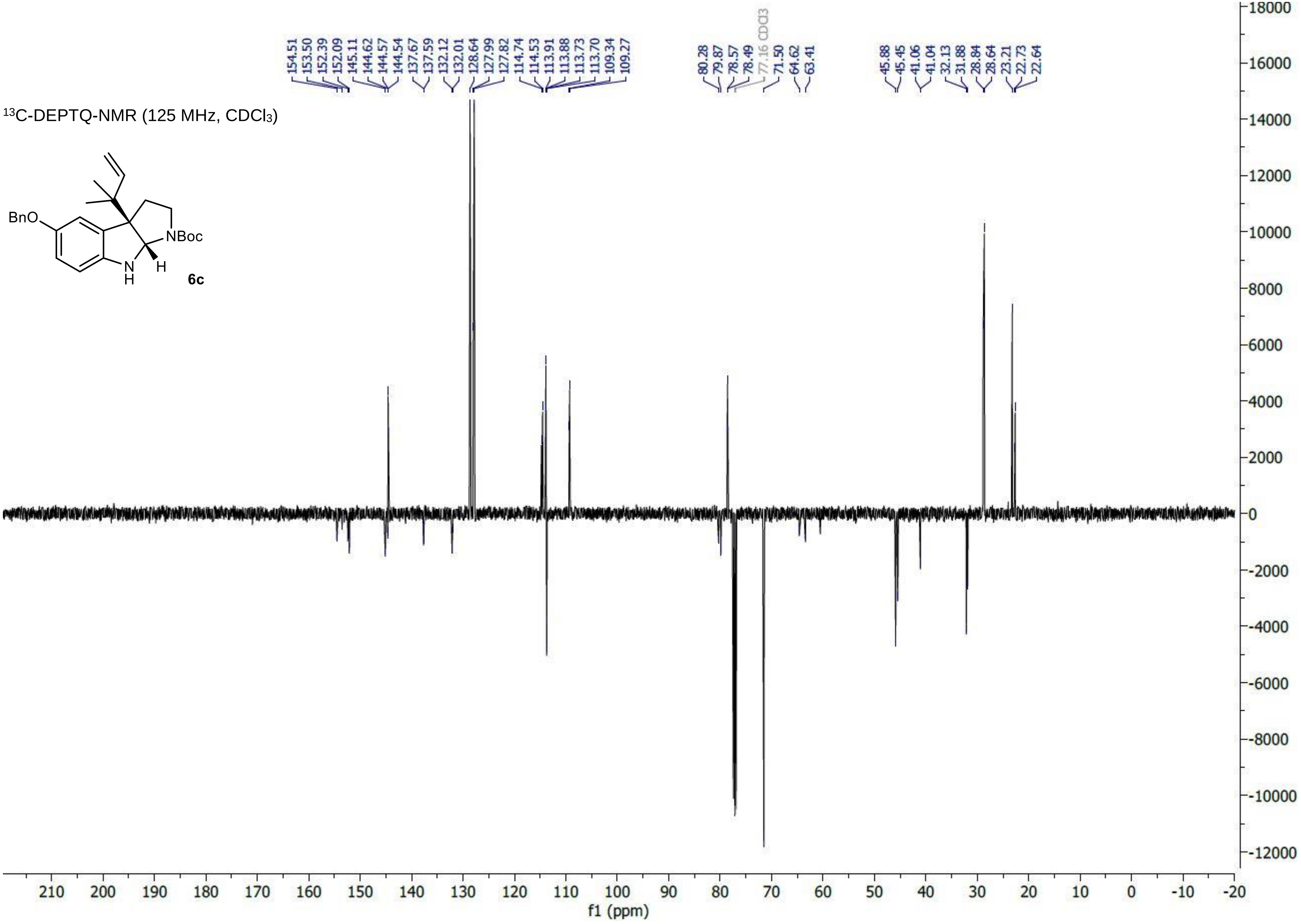

^{1}H-NMR (600 MHz, $CDCl_3$)

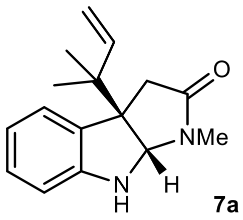

7a

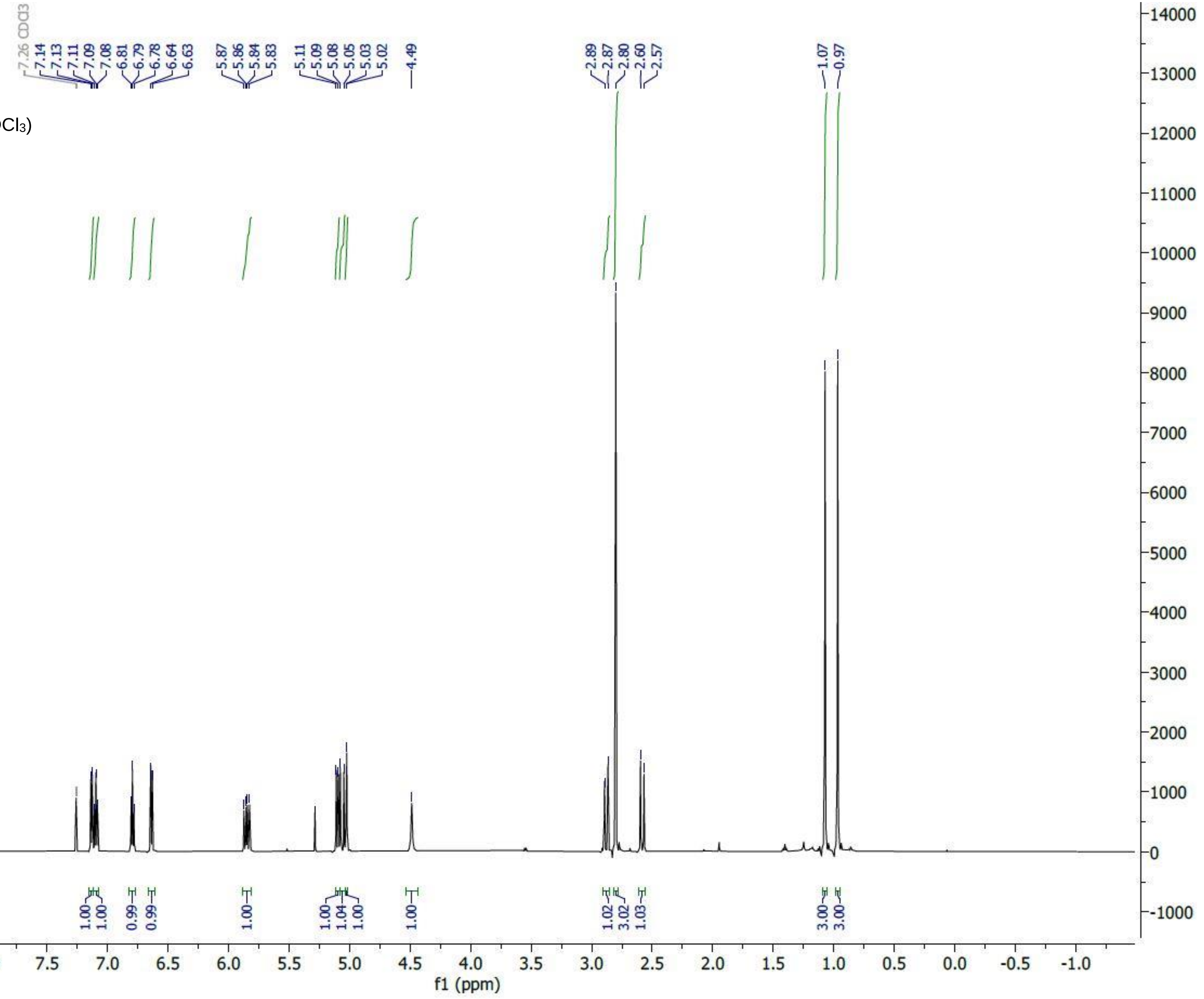

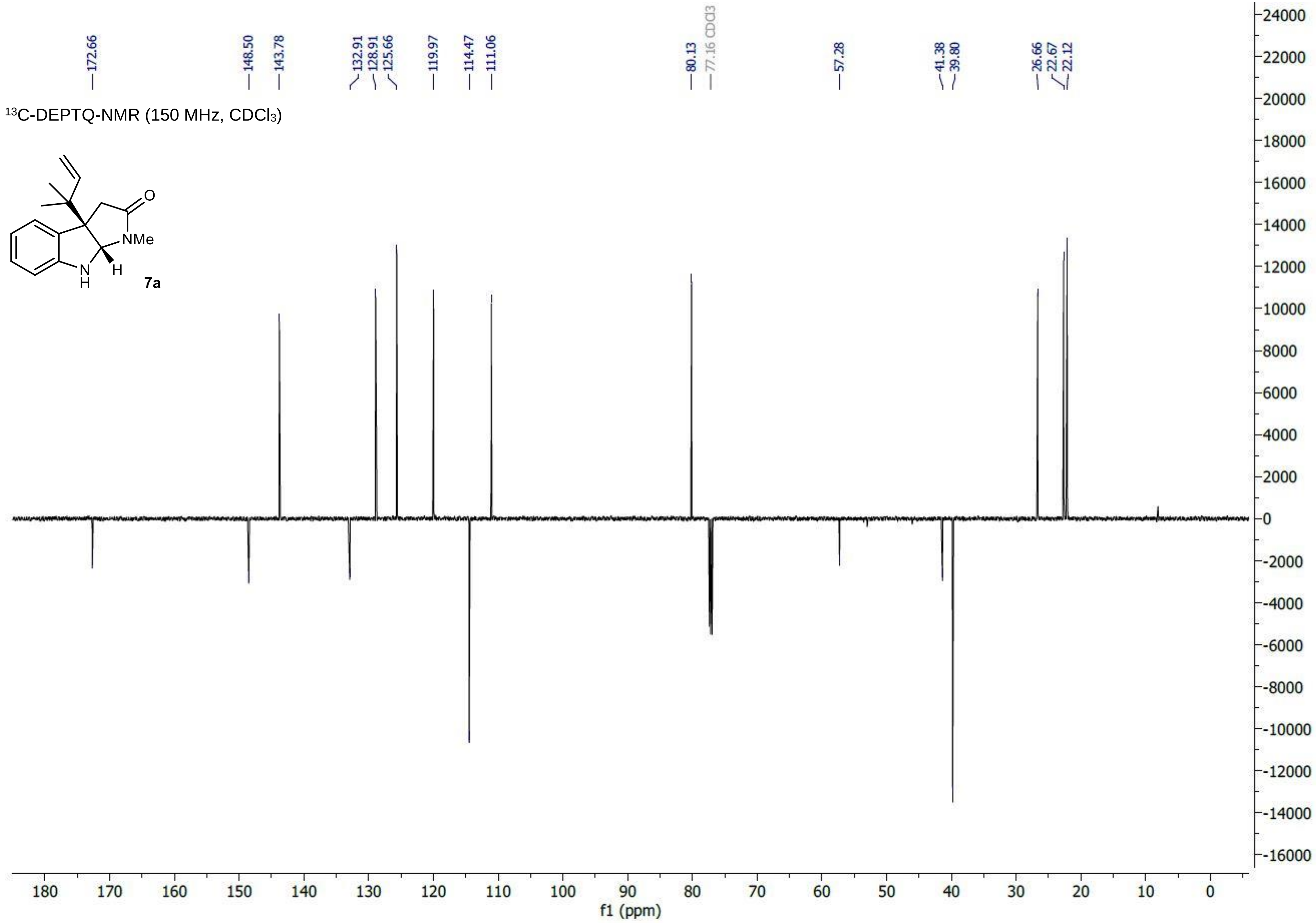
^{13}C-DEPTQ-NMR (150 MHz, $CDCl_3$)
7a
O
NMe
N
H
H
172.66
148.50
143.78
132.91
128.91
125.66
119.97
114.47
111.06
80.13
77.16 CDCl3
57.28
41.38
39.80
26.66
22.67
22.12
f1 (ppm)

^{1}H-NMR (600 MHz, $CDCl_3$)

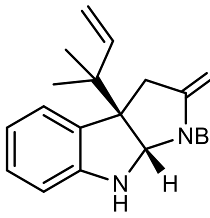

7b

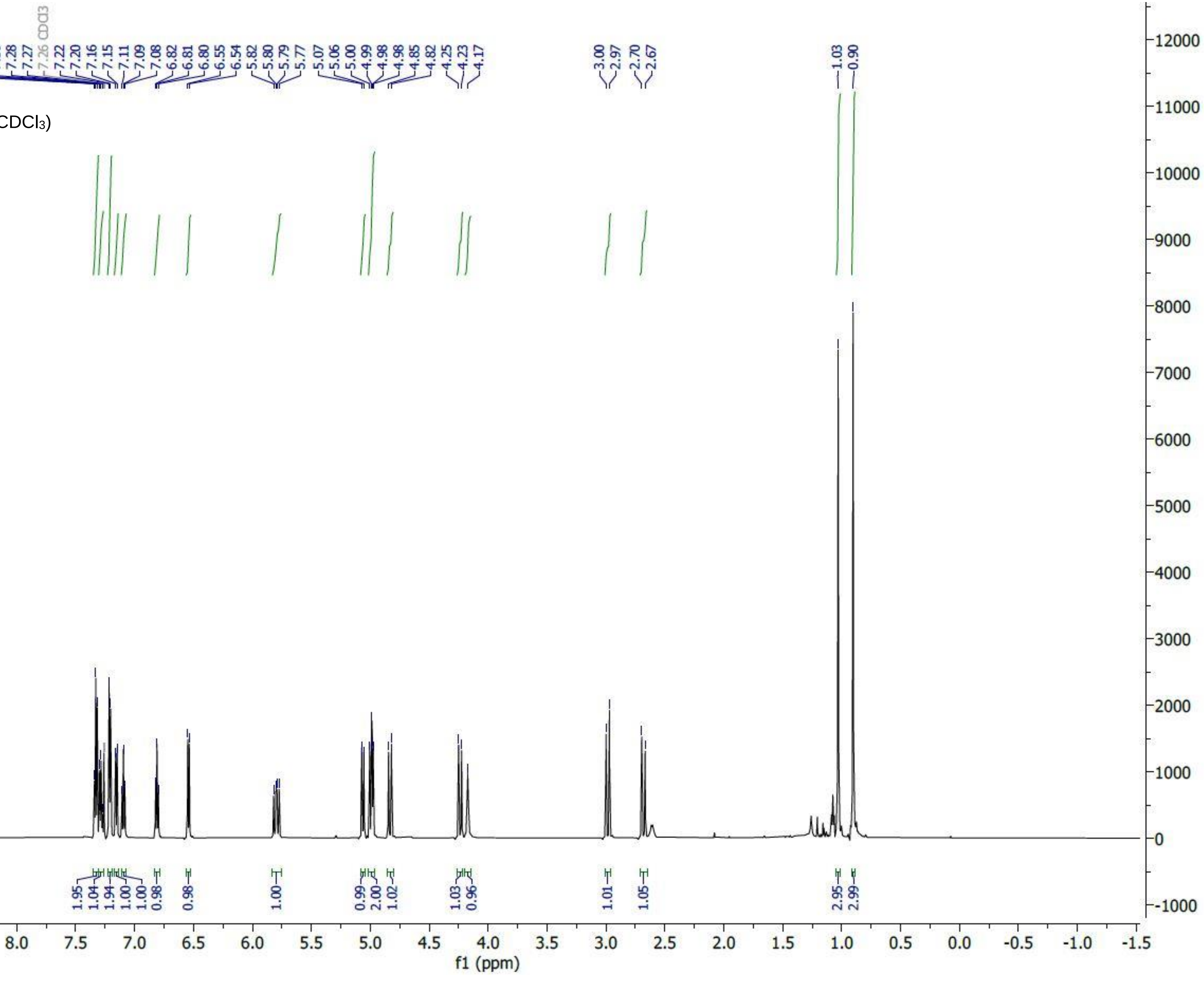

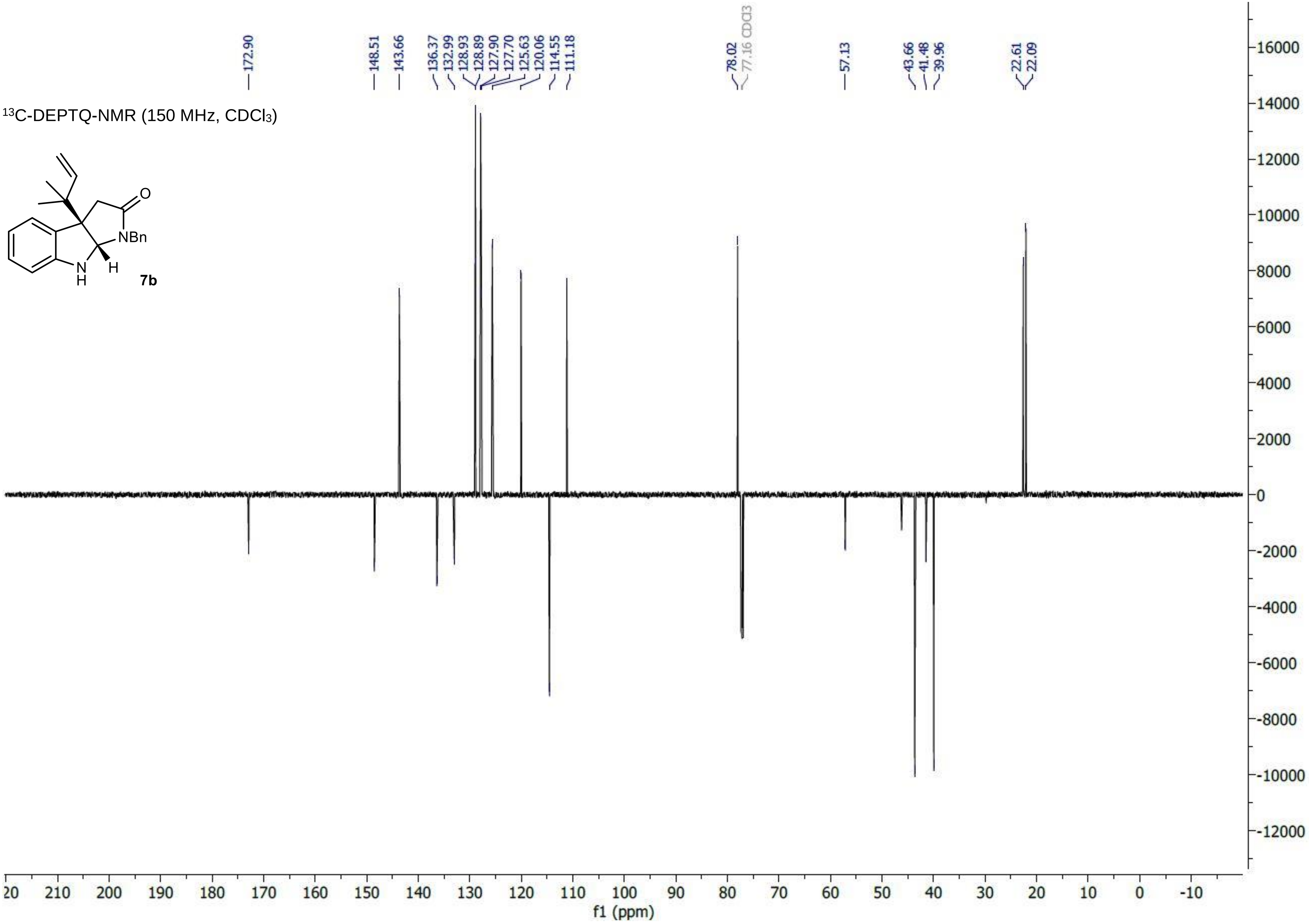

13C-DEPTQ-NMR (150 MHz, CDCl3)
NBn
O
N
H
H
7b
172.90
148.51
143.66
136.37
132.99
128.93
128.89
127.90
127.70
125.63
120.06
114.55
111.18
78.02
77.16 CDCl3
57.13
43.66
41.48
39.96
22.61
22.09
f1 (ppm)

^{1}H-NMR (500 MHz, $CDCl_3$)

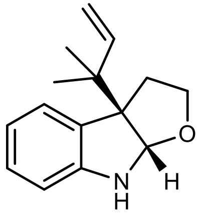

8a

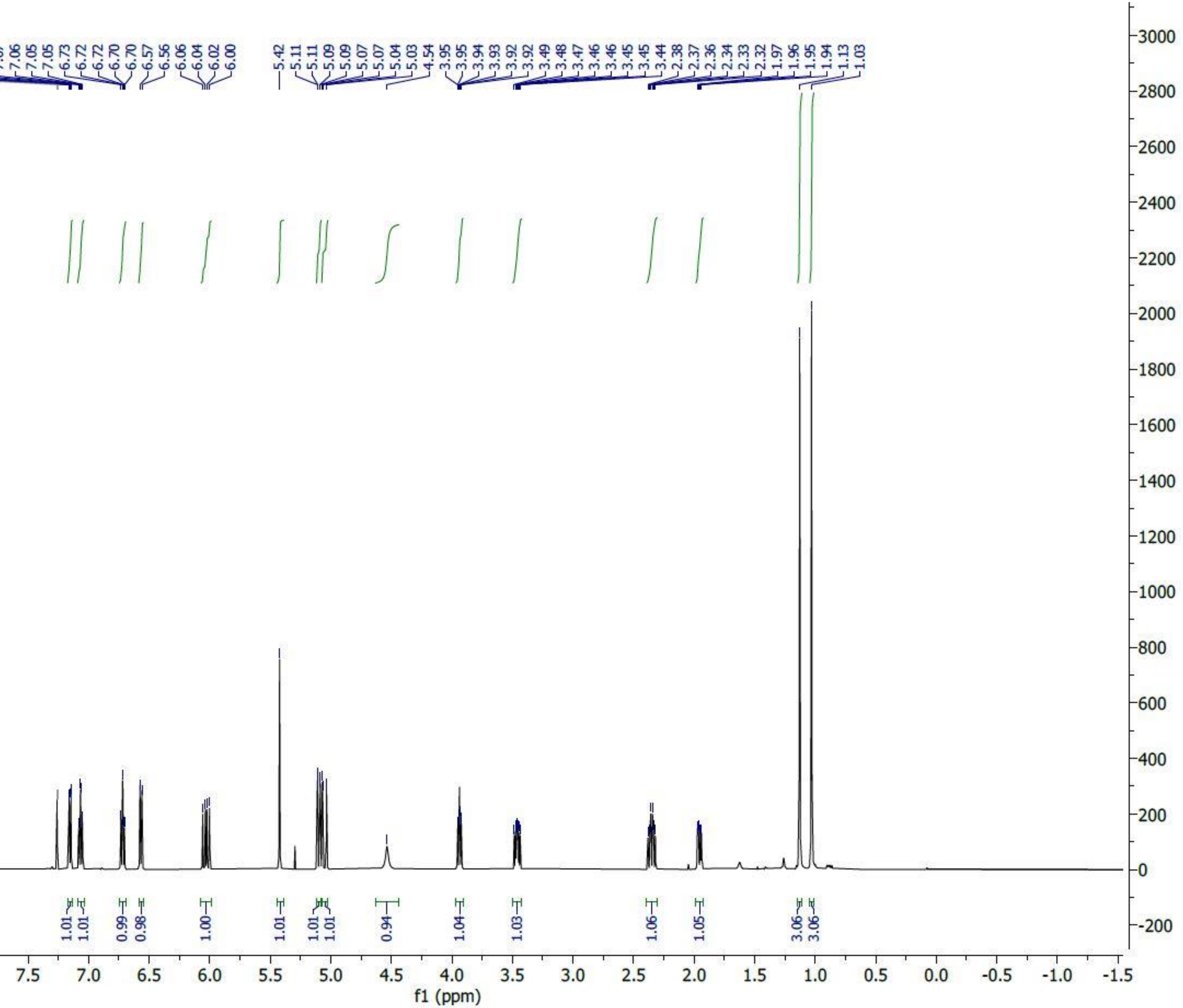

^{13}C-DEPTQ-NMR (125 MHz, $CDCl_3$)

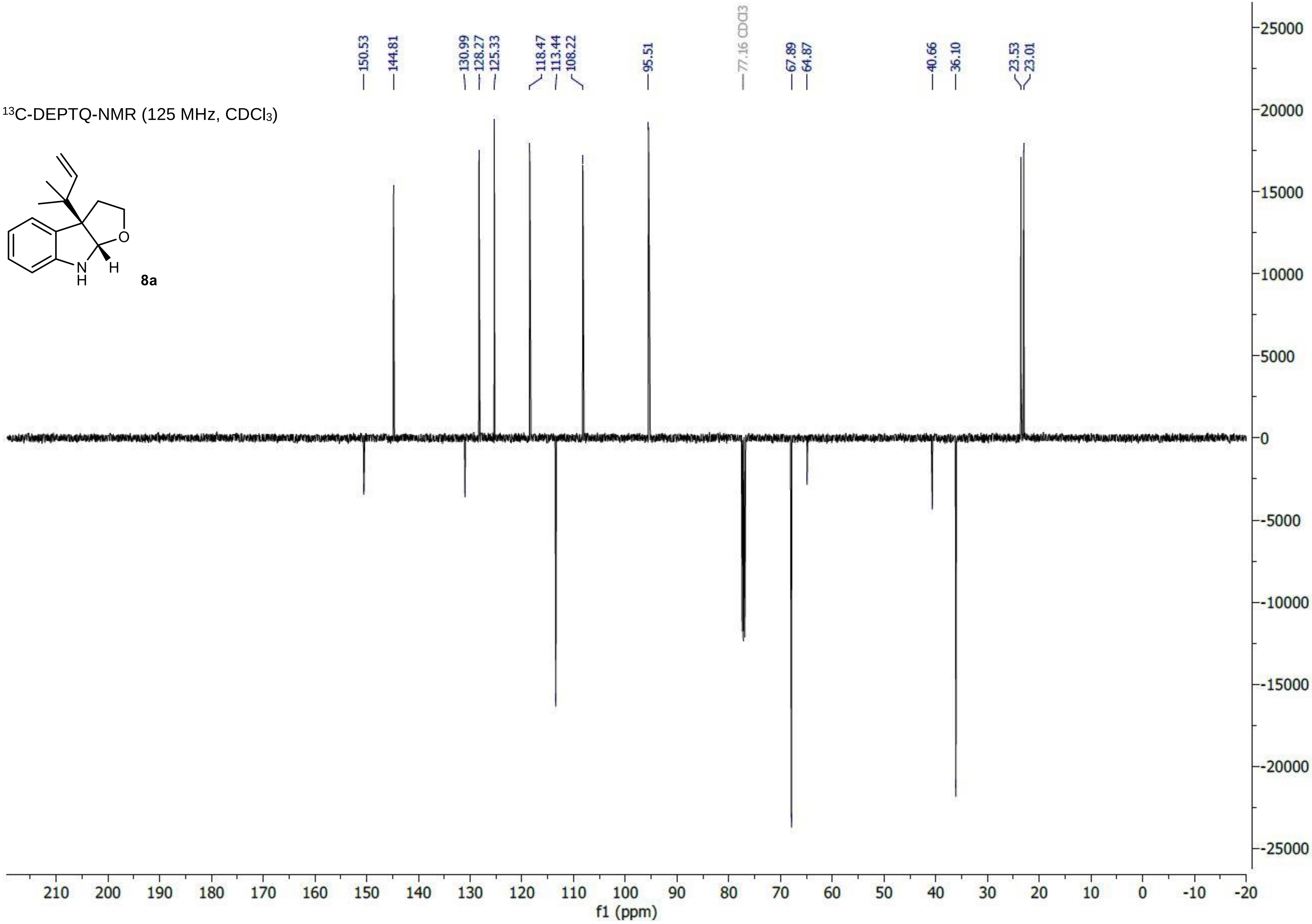

8a

^{1}H-NMR (600 MHz, $CDCl_3$)

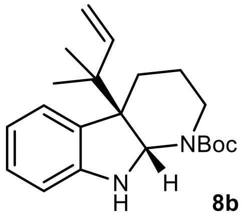

8b

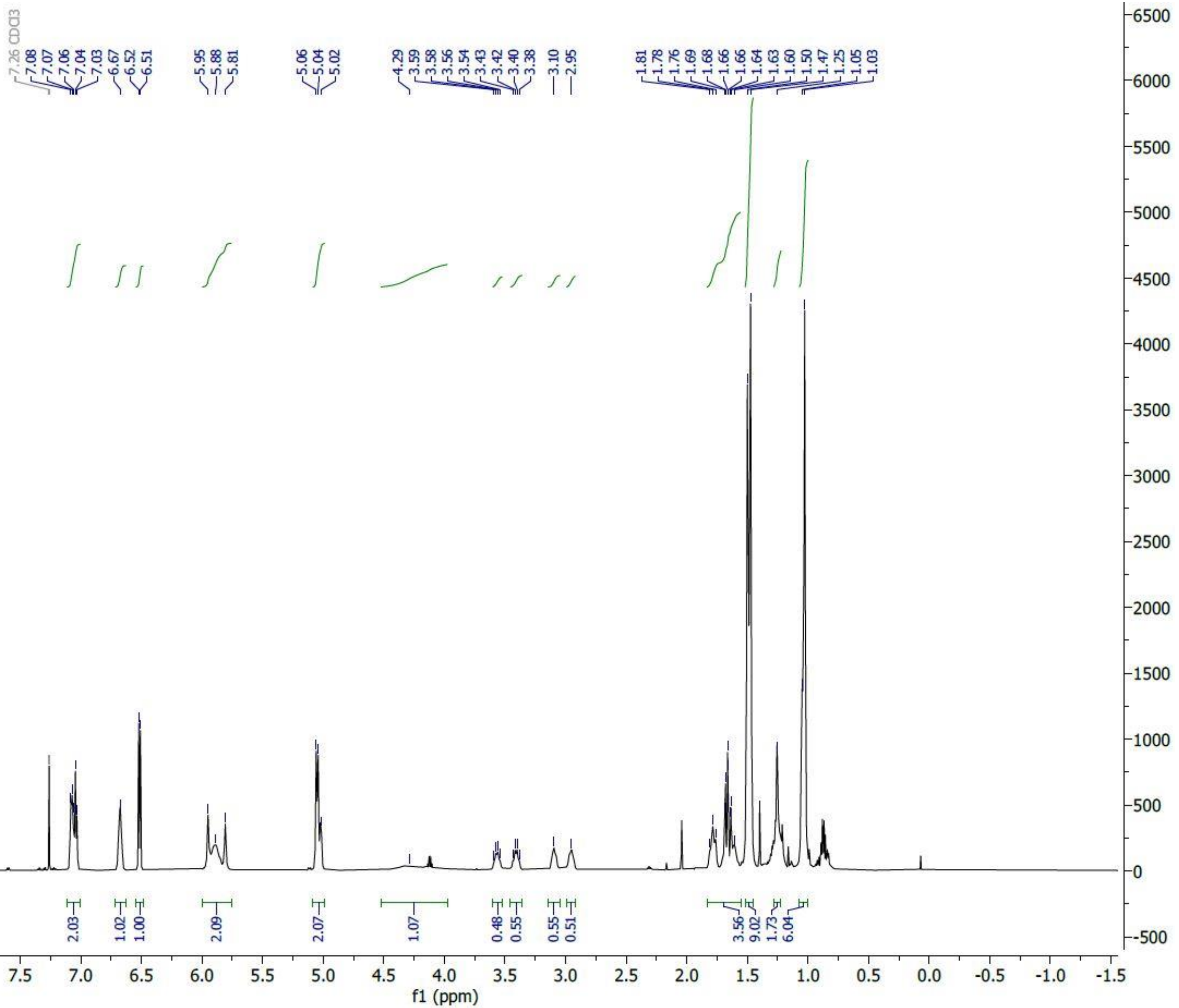

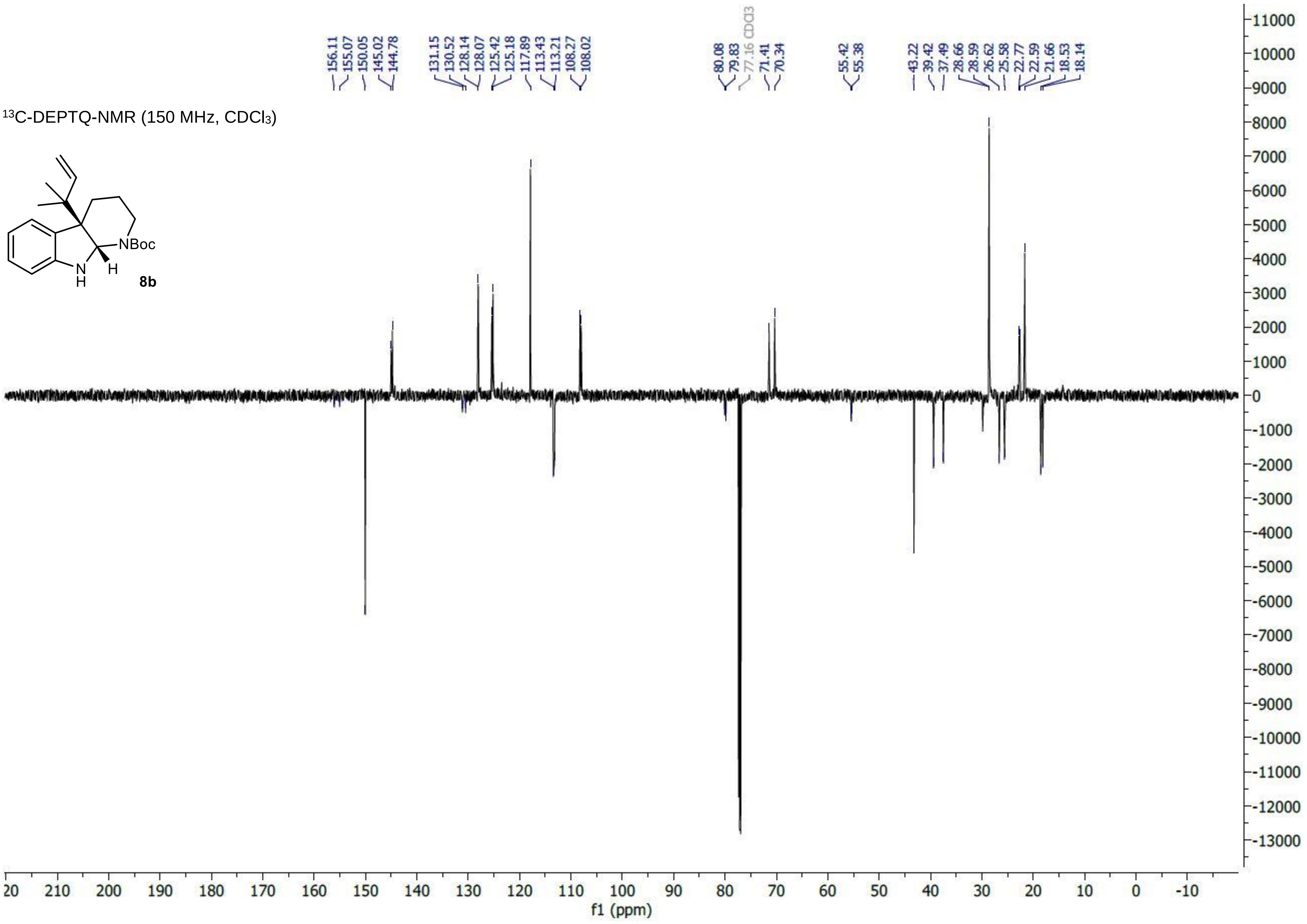

^{13}C-DEPTQ-NMR (150 MHz, $CDCl_3$)
NBoc
N
H
H
8b
156.11
155.07
150.05
145.02
144.78
131.15
130.52
128.14
128.07
125.42
125.18
117.89
113.43
113.21
108.27
108.02
80.08
79.83
77.16 CDCl3
71.41
70.34
55.42
55.38
43.22
39.42
37.49
28.66
28.59
26.62
25.58
22.77
22.59
21.66
18.53
18.14
f1 (ppm)

^{1}H-NMR (600 MHz, $CDCl_3$)

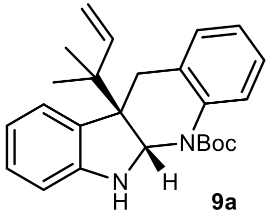

9a

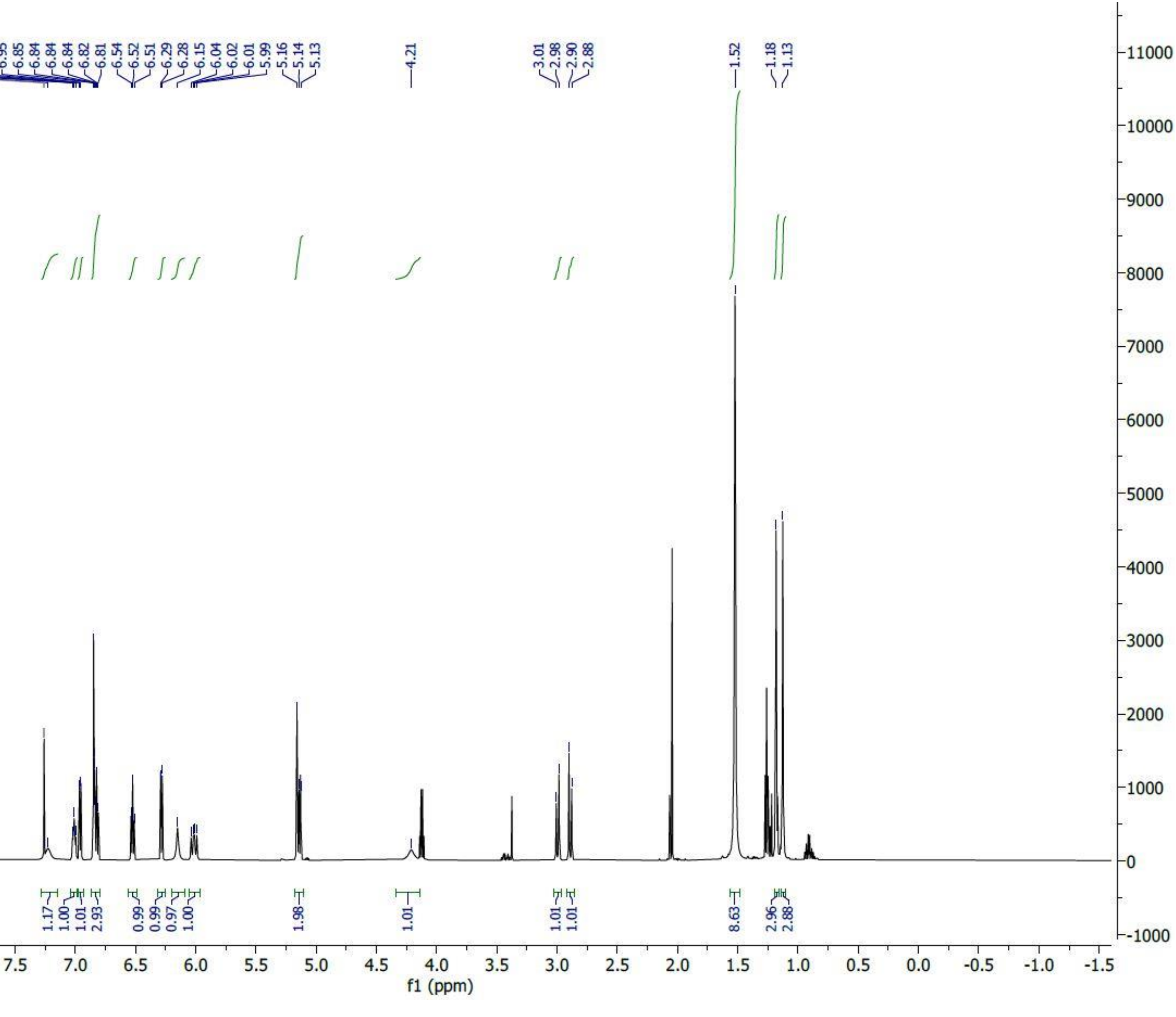

^{13}C-DEPTQ-NMR (125 MHz, $CDCl_3$)

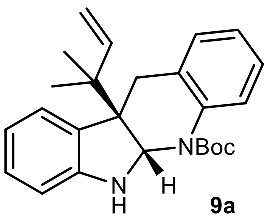

9a

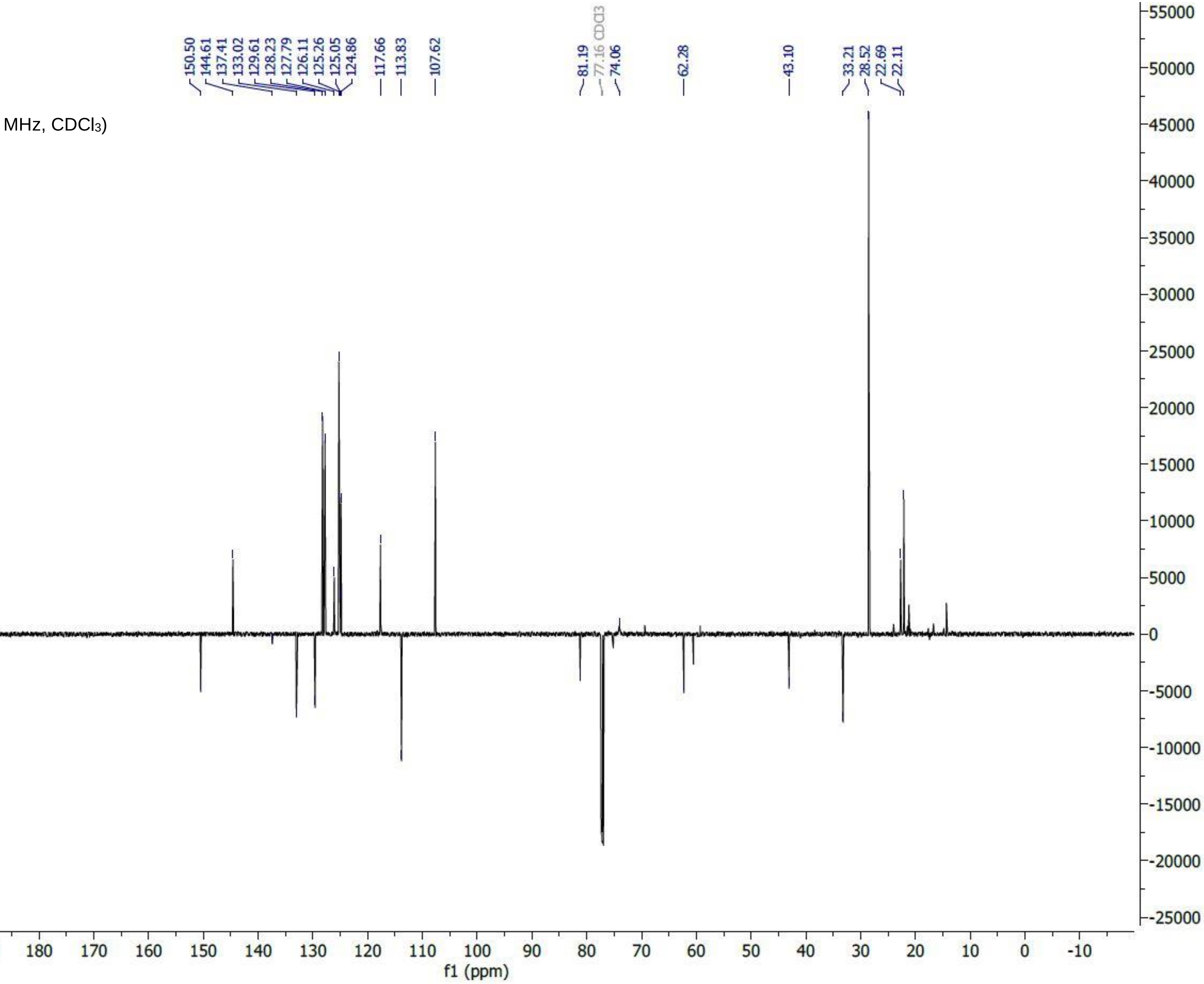

^{1}H-NMR (400 MHz, $CDCl_3$)

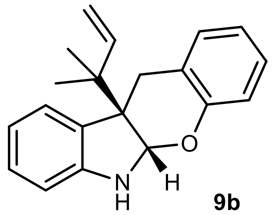

9b

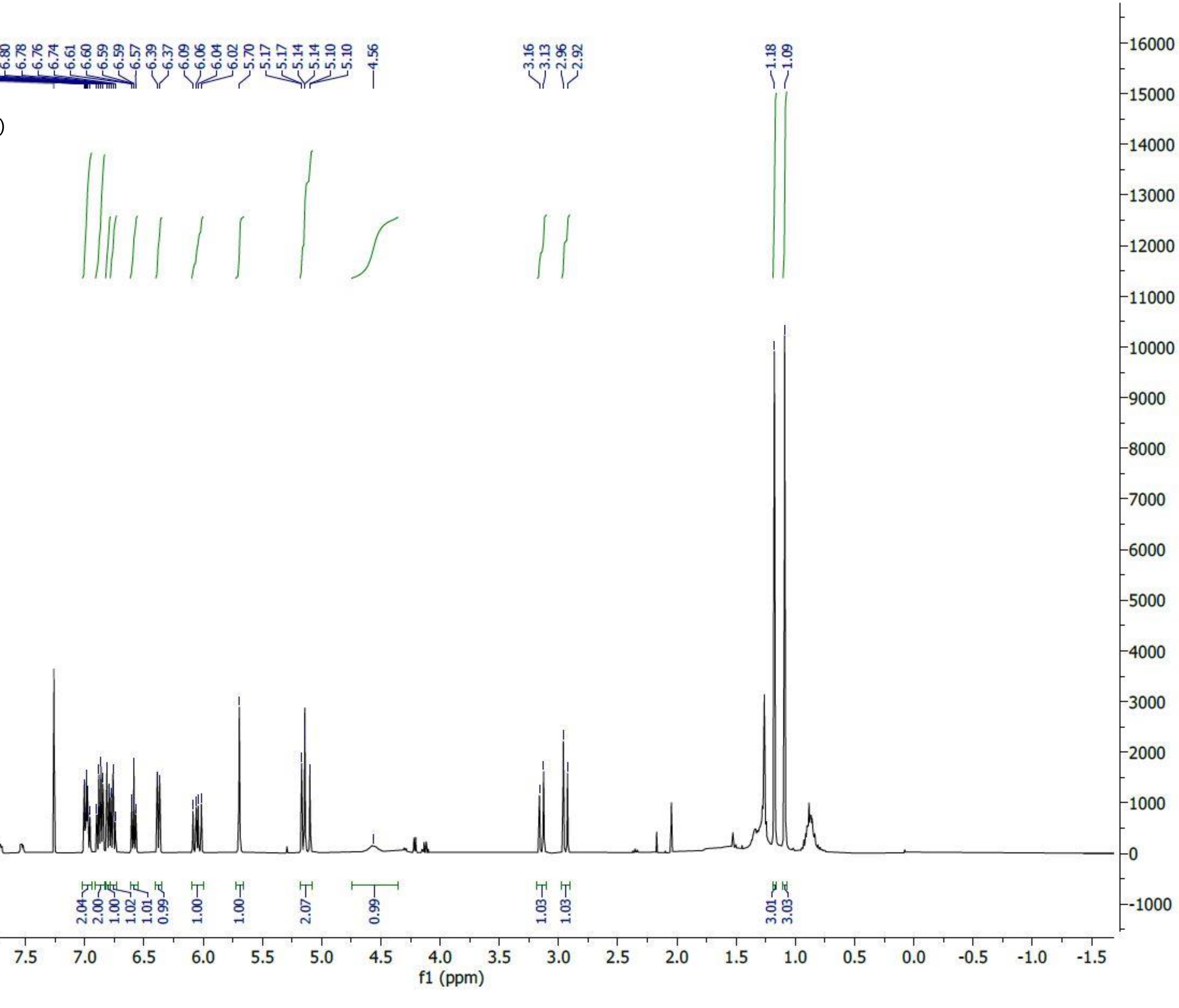

^{13}C-DEPTQ-NMR (100 MHz, $CDCl_3$)

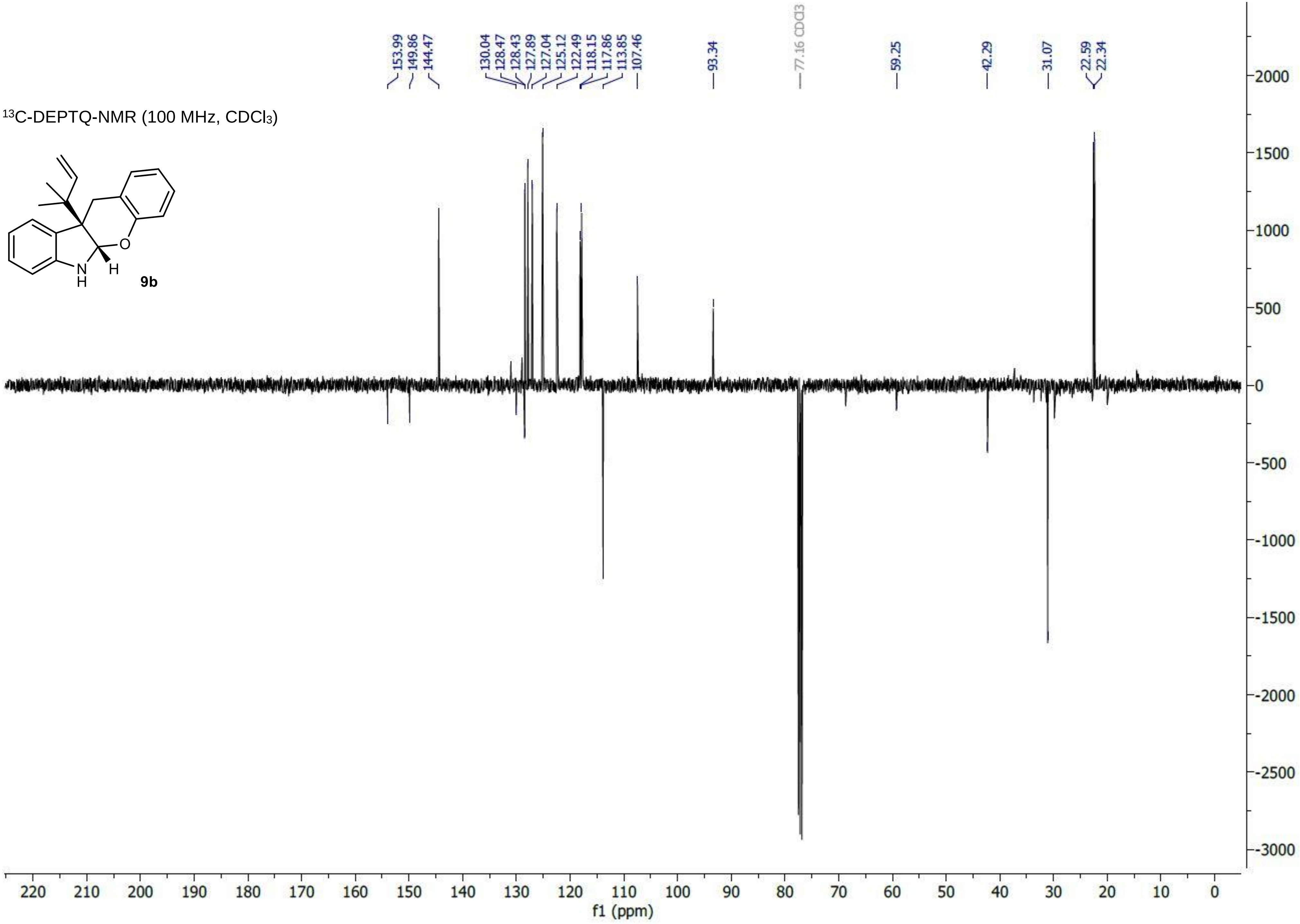

9b

^{1}H-NMR (400 MHz, $CDCl_3$)

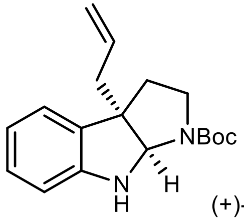

(+)-**10**

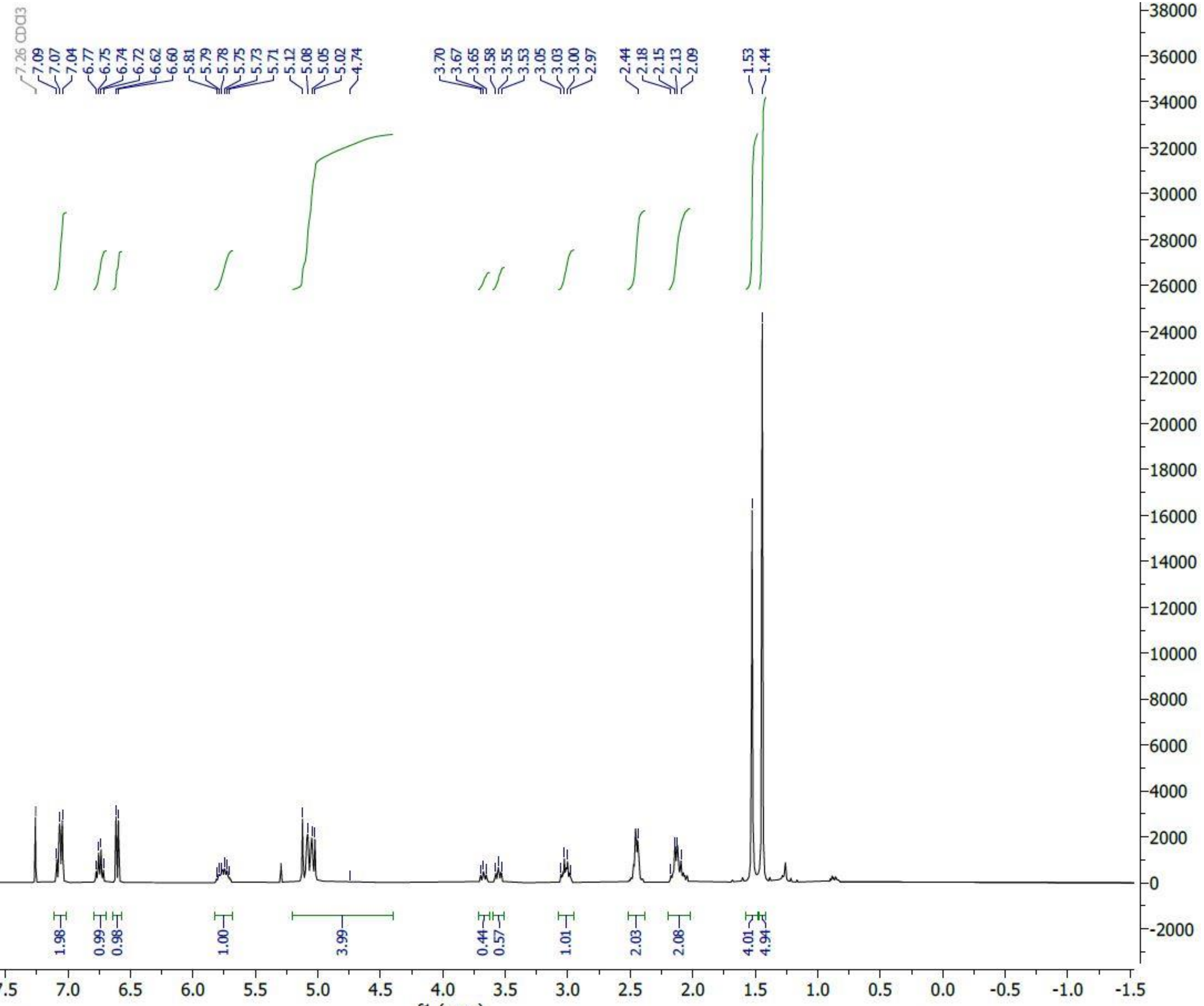

^{13}C-DEPTQ-NMR (100 MHz, $CDCl_3$)

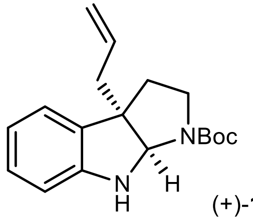

(+)-**10**

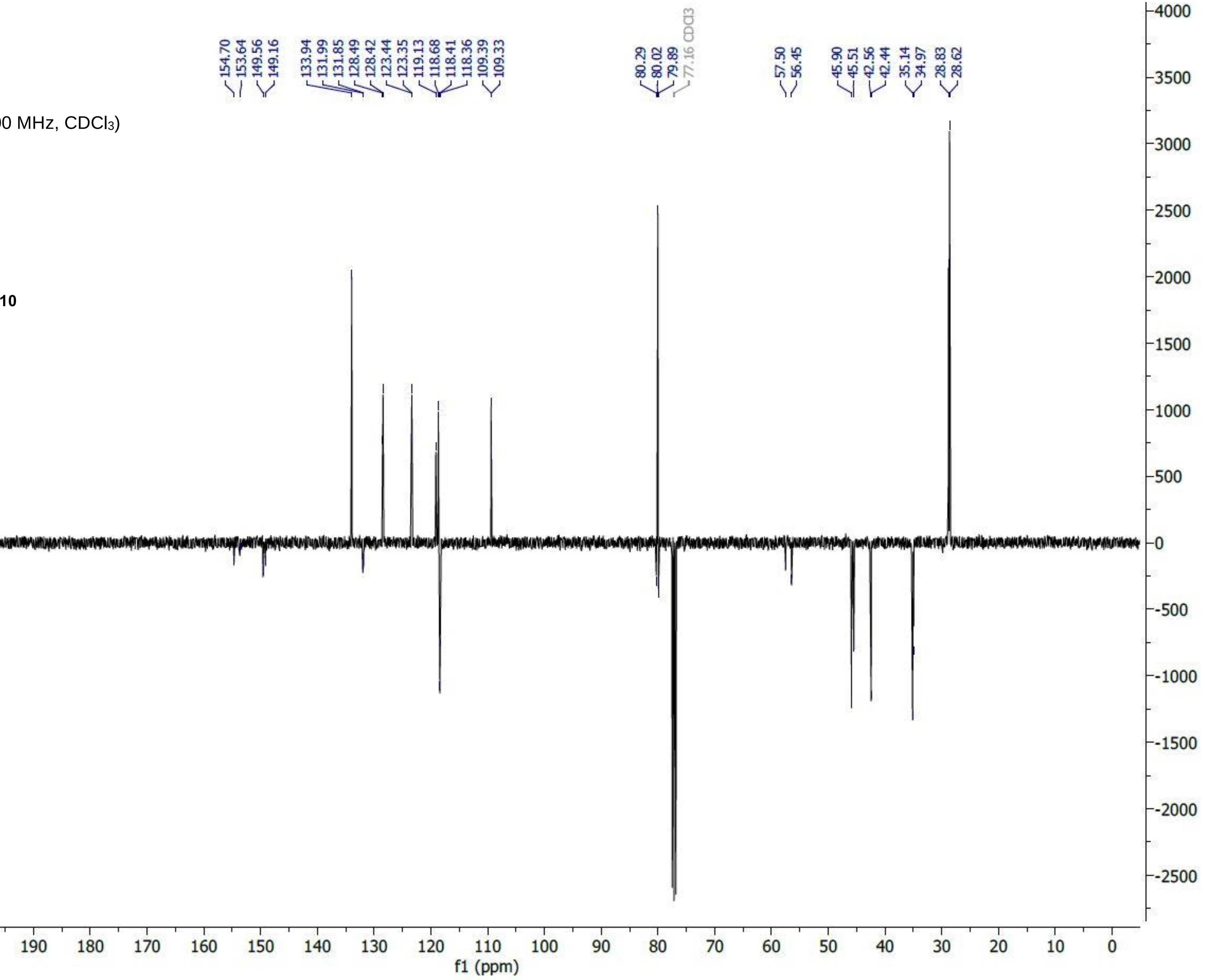

^{1}H-NMR (600 MHz, $CDCl_3$)

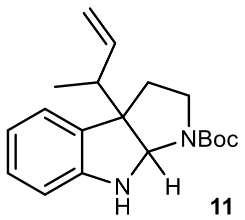

11

prepared according to

GP 7

dr = 1/3.0

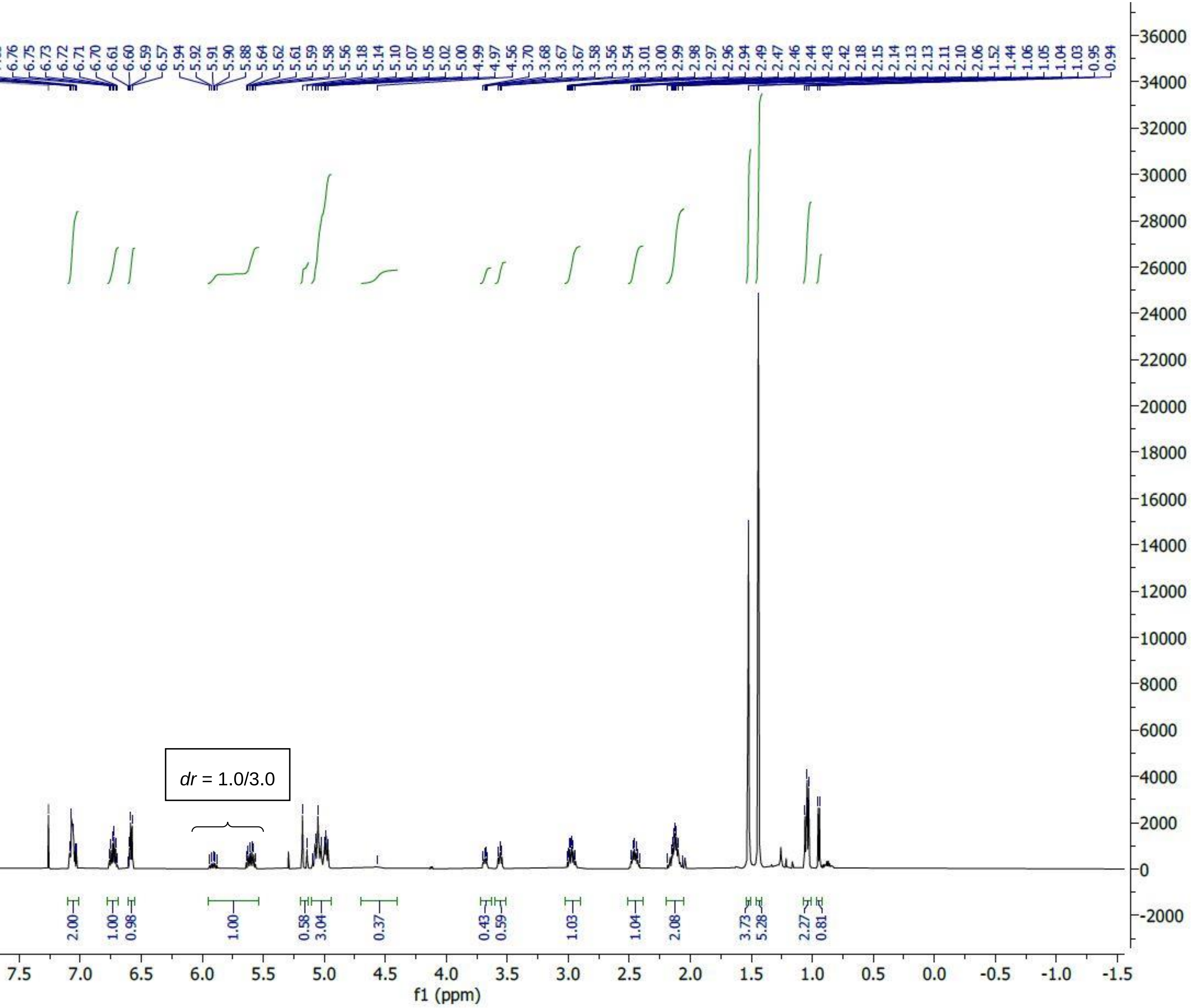

^{13}C-DEPTQ-NMR (150 MHz, $CDCl_3$)

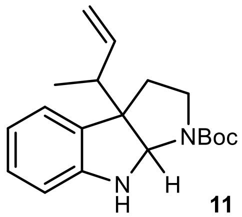

11

prepared according to
GP 7
dr = 1/3.0

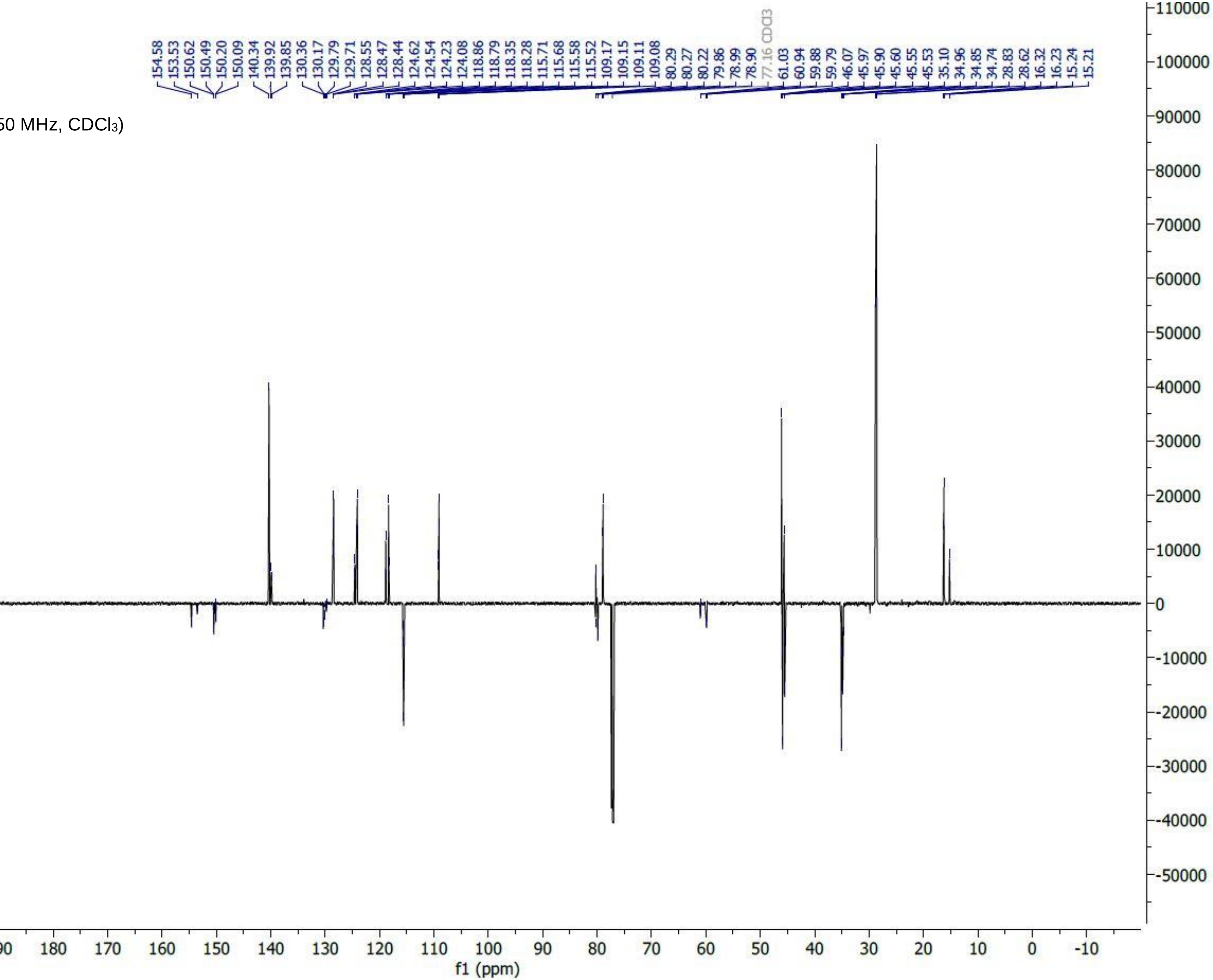

^{1}H-NMR (600 MHz, $CDCl_3$)

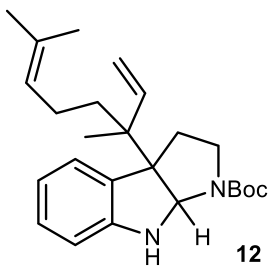

12

prepared according to
GP 7
dr = 1/1.46

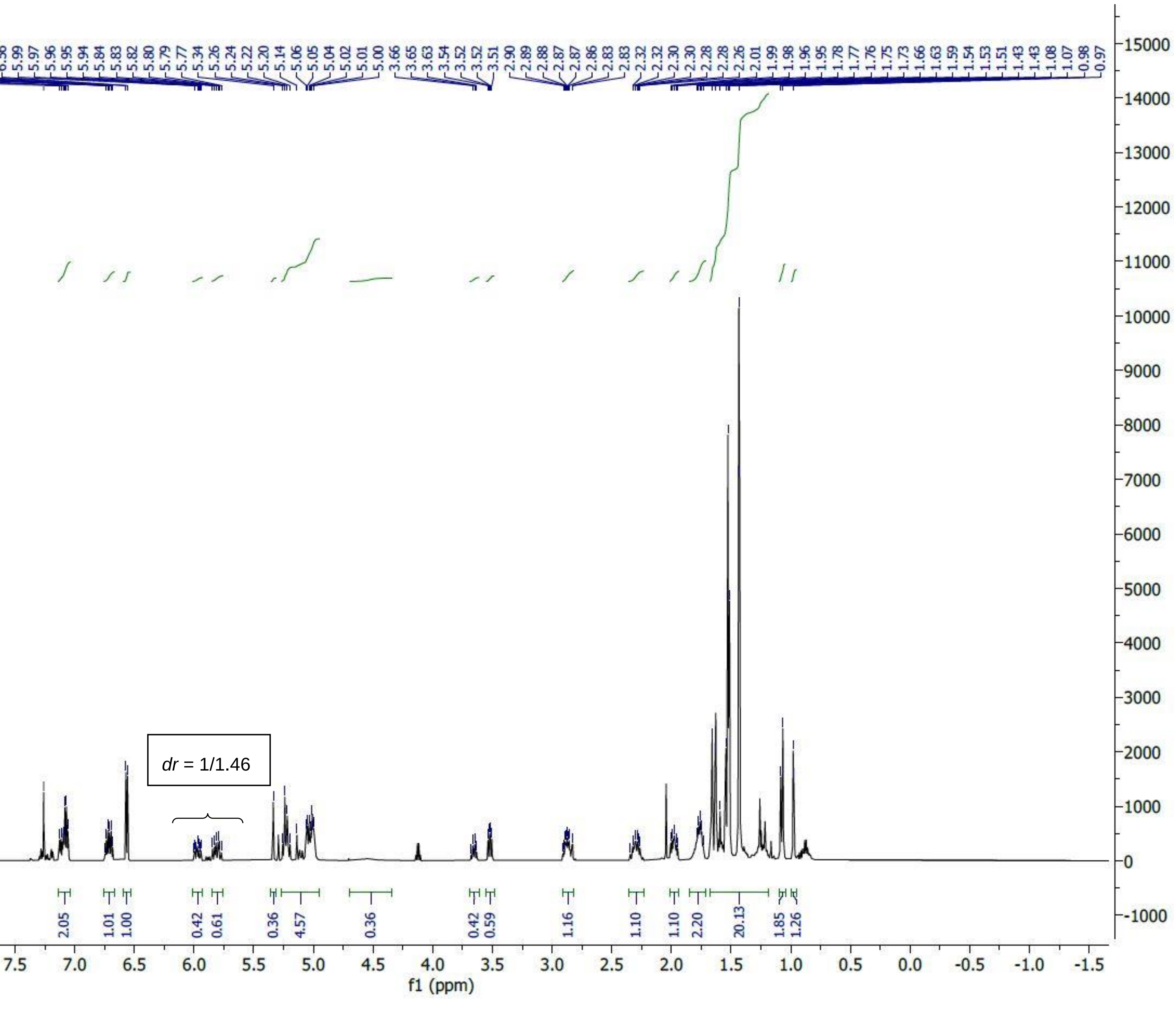

^{13}C-DEPTQ-NMR (150 MHz, $CDCl_3$)

12

prepared according to
GP 7
dr = 1/1.46

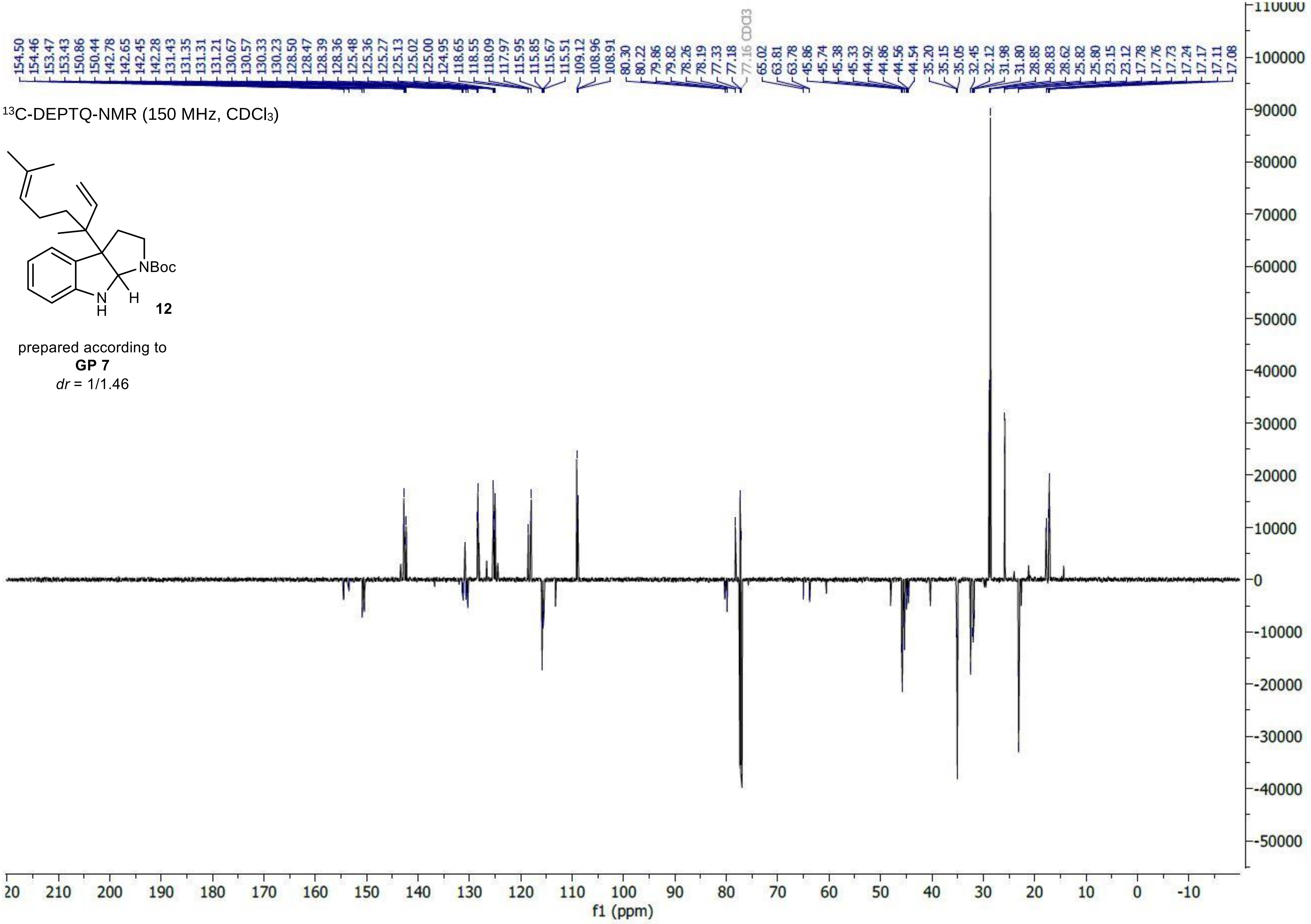

1H-NMR (600 MHz, $CDCl_3$)

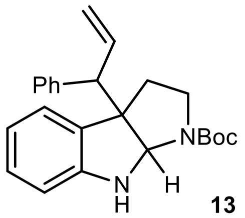

13

prepared according to
GP 7, (*rac*)-**1e**, (*R*)-**L1**
dr = 1/1.1

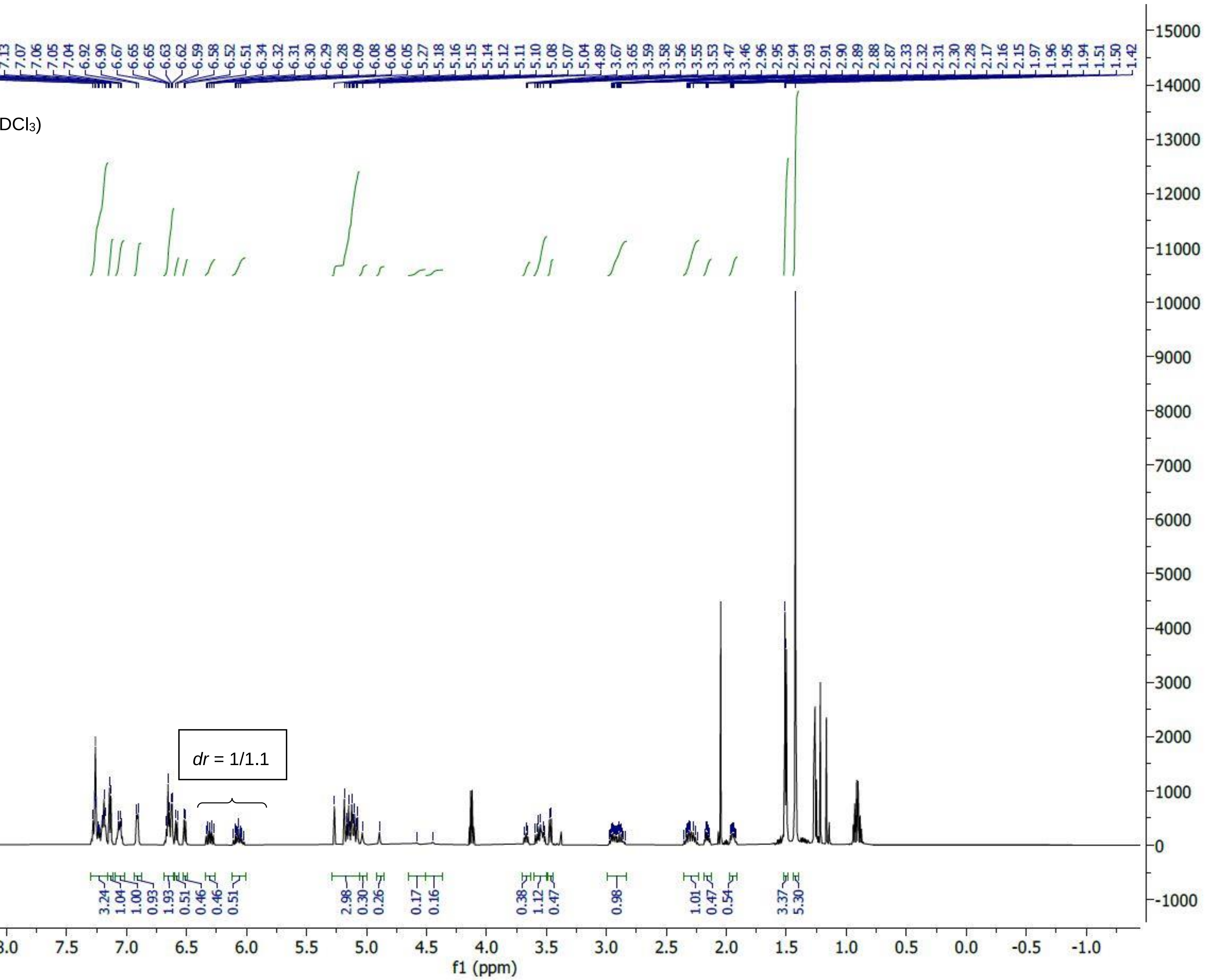

^{13}C-DEPTQ-NMR (150 MHz, $CDCl_3$)

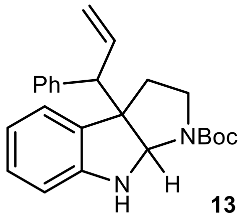

13

prepared according to
GP 7, (*rac*)-**1e**, (*R*)-**L1**
dr = 1/1.1

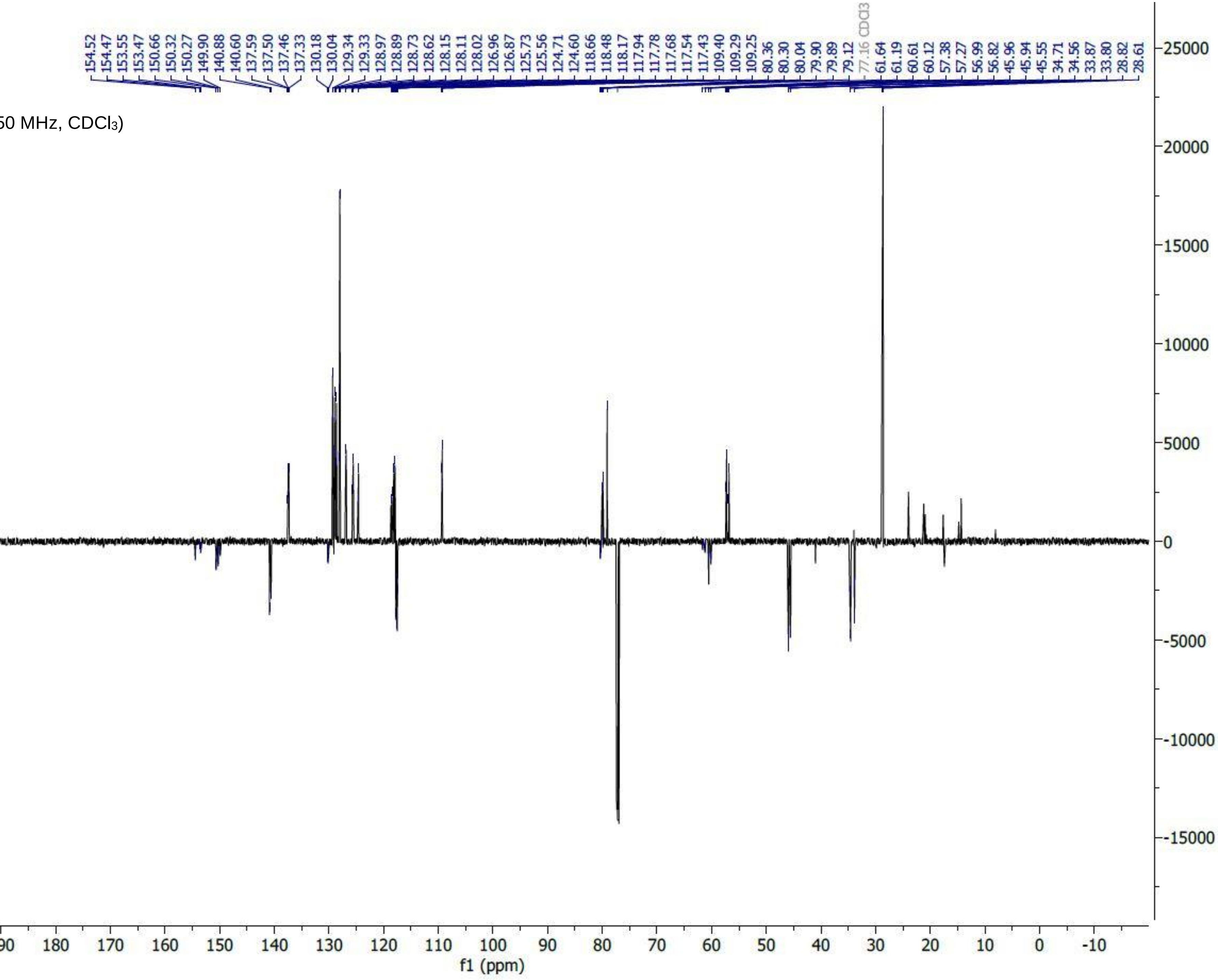

12.6. Synthesis of (–)-Flustramine A (14)

^{1}H-NMR (600 MHz, $CDCl_3$)

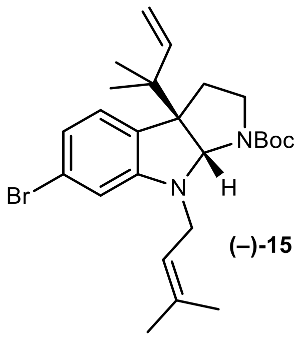

(–)-15

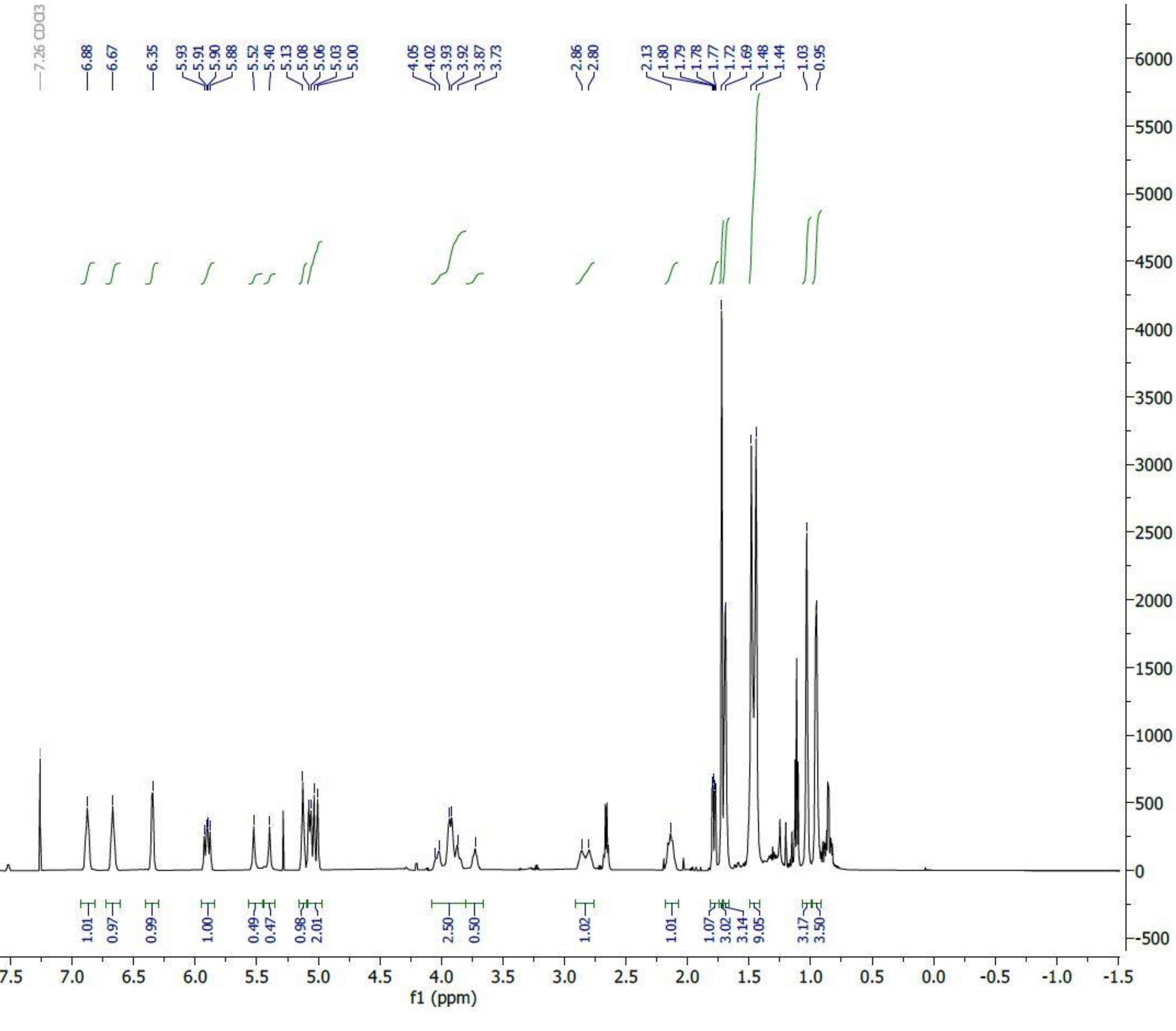

^{13}C-DEPTQ-NMR (150 MHz, $CDCl_3$)

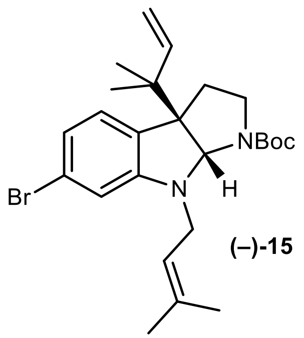

(–)-15

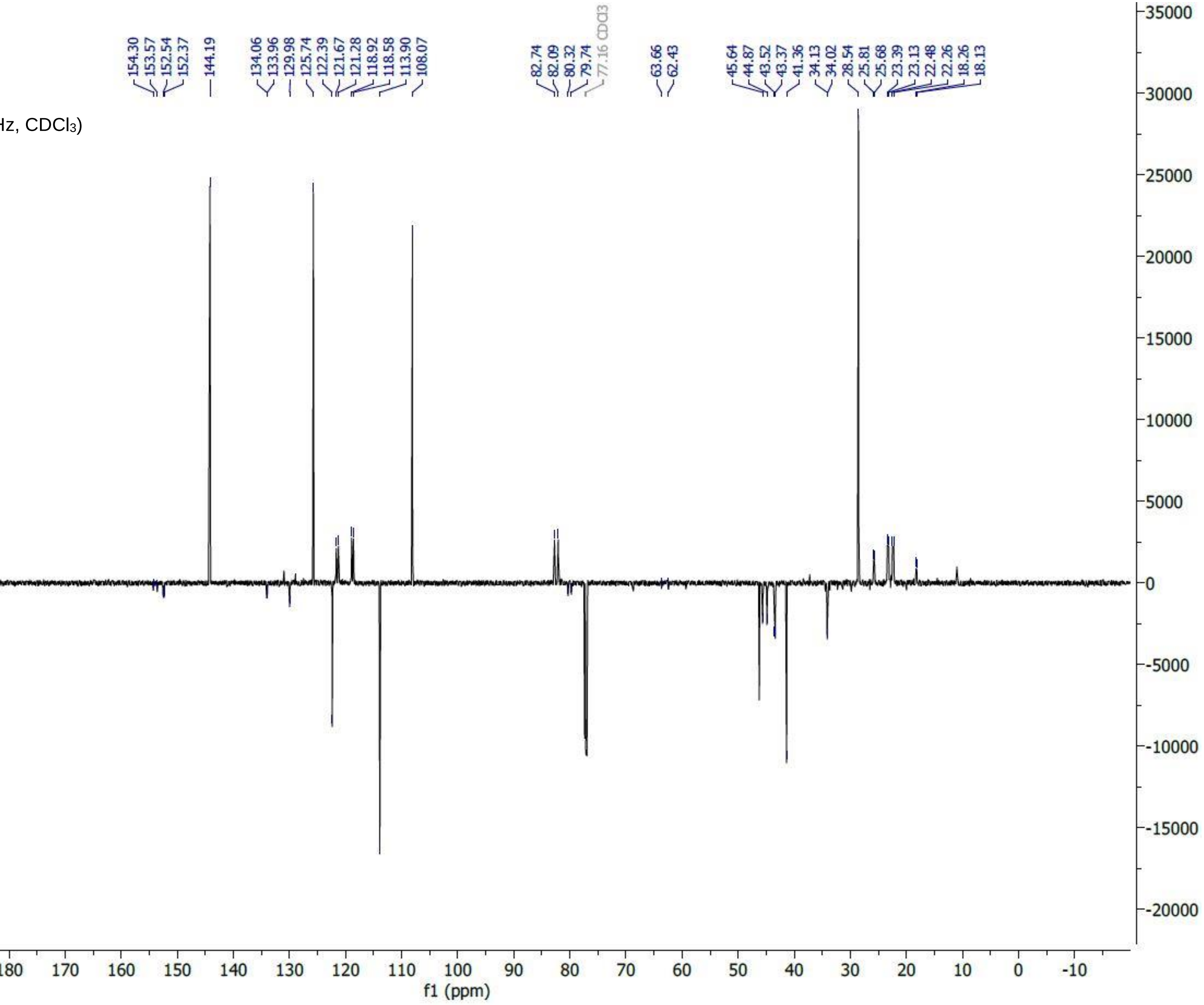

¹H-NMR (600 MHz, $CDCl_3$)

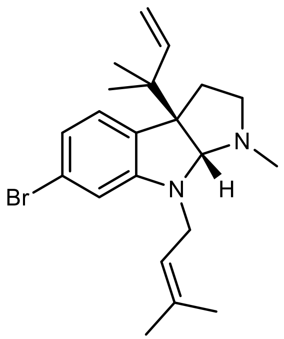

(–)-Flustramine A (14)

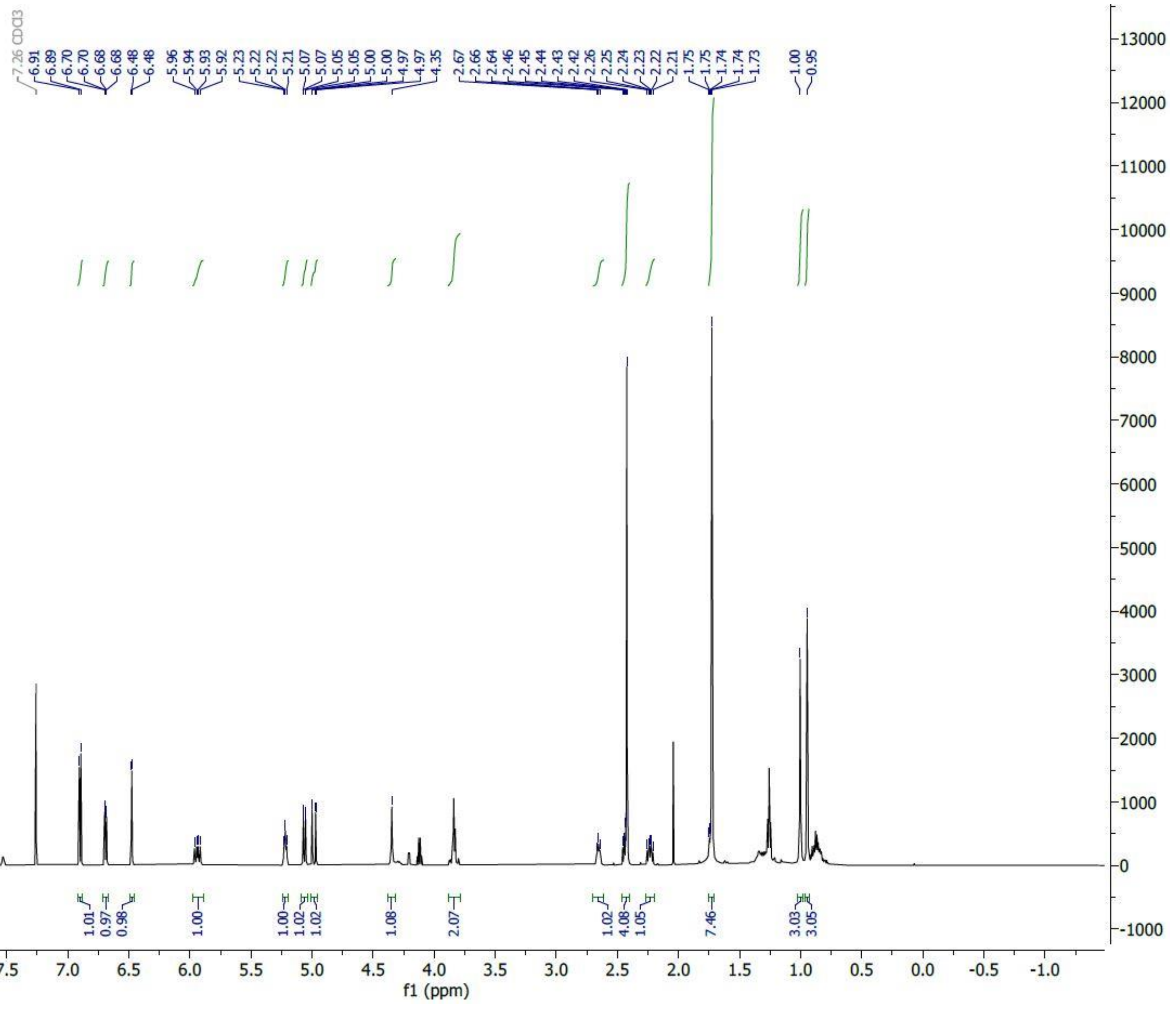

^{13}C-DEPTQ-NMR (150 MHz, $CDCl_3$)

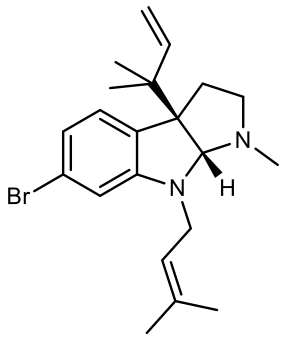

(–)-Flustramine A (14)

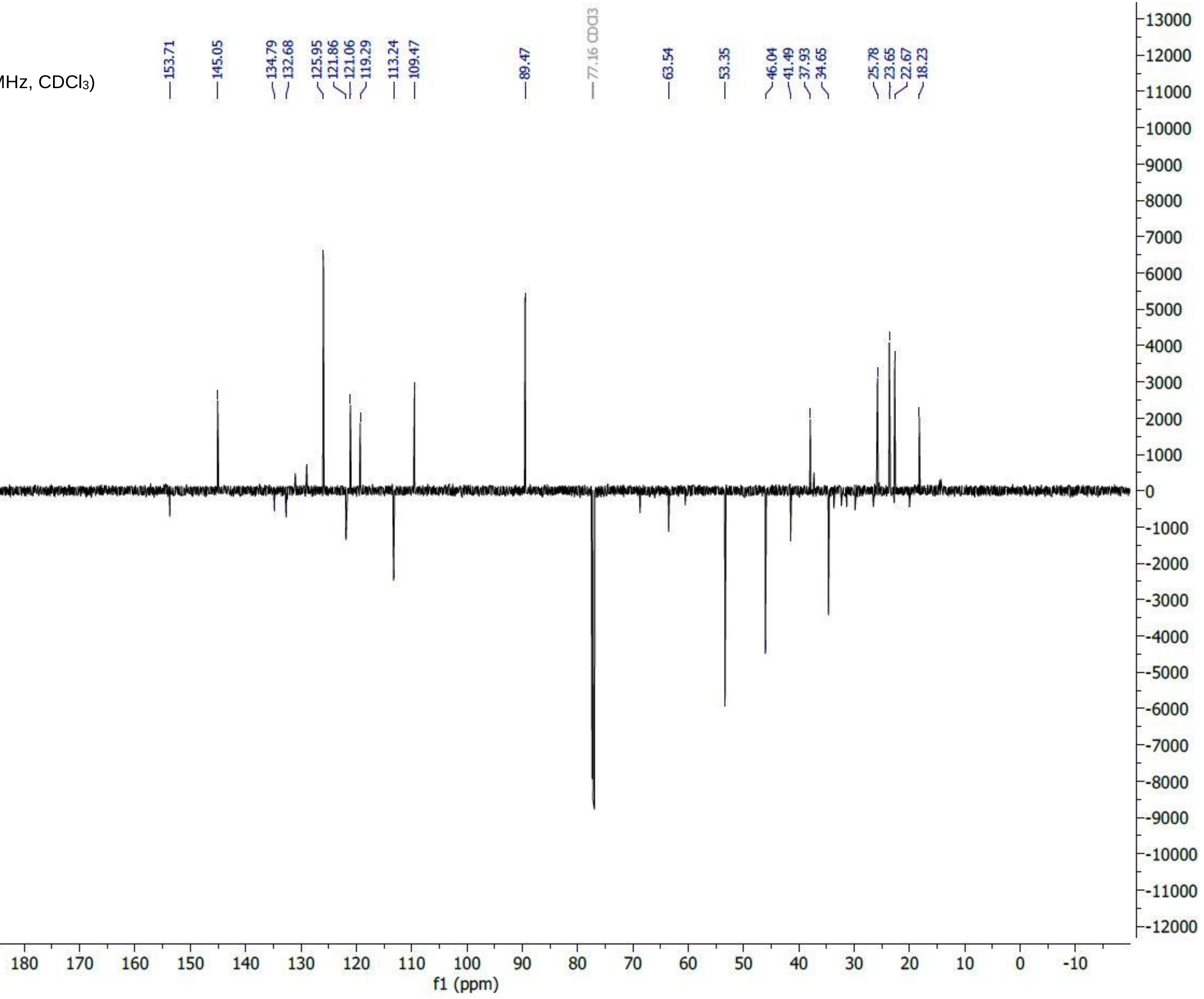

13. HPLC-chromatograms

13.1. compounds 3–10

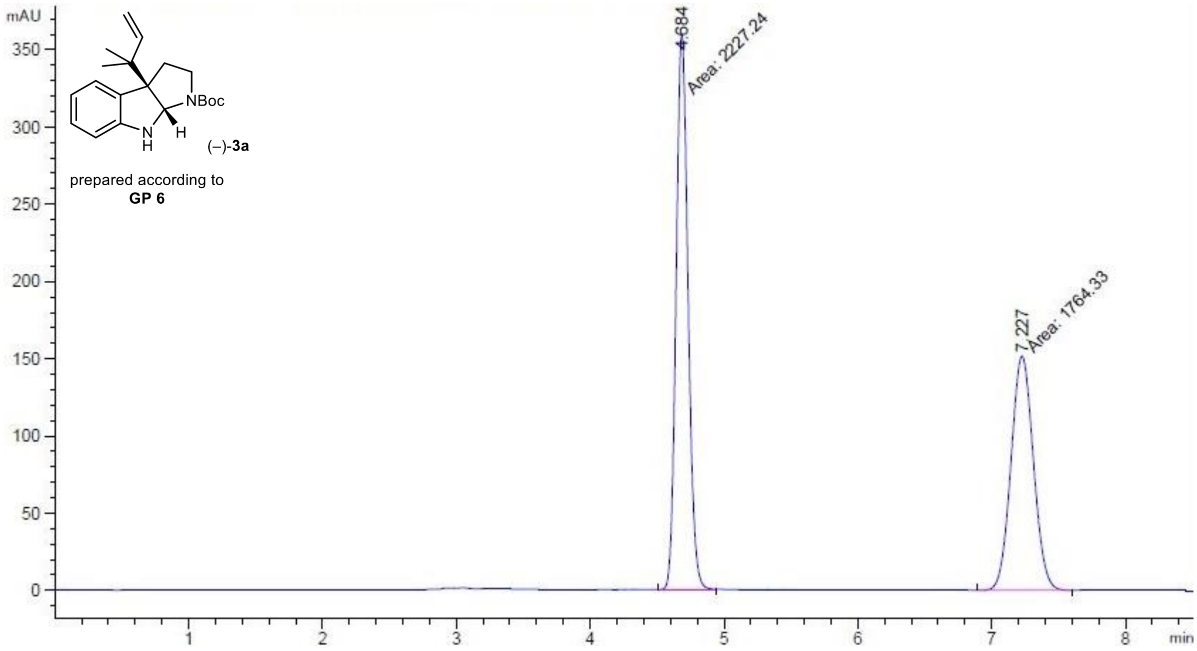

Peak #	RetTime [min]	Type	Width [min]	Area [mAU*s]	Height [mAU]	Area %
1	4.684	MF	0.1033	2227.24243	359.29556	55.7986
2	7.227	MF	0.1941	1764.32959	151.48192	44.2014

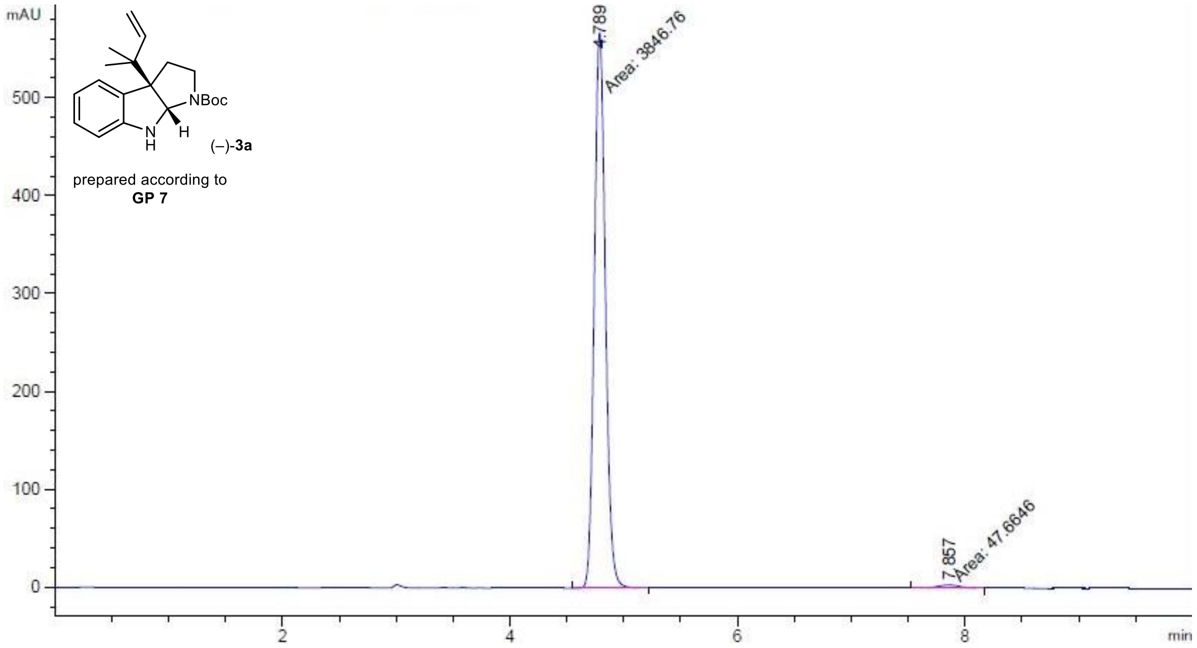

Peak #	RetTime [min]	Type	Width [min]	Area [mAU*s]	Height [mAU]	Area %
1	4.789	MF	0.1130	3846.76343	567.24286	98.7761
2	7.857	MF	0.2385	47.66460	3.33094	1.2239

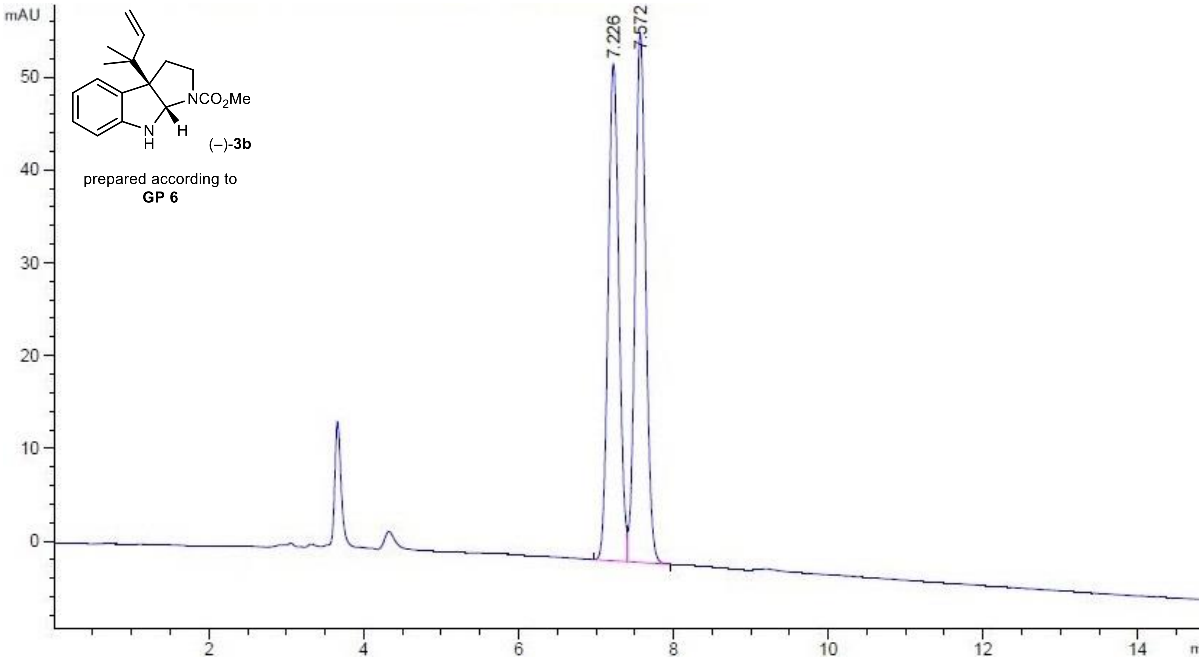

Peak #	RetTime [min]	Type	Width [min]	Area [mAU*s]	Height [mAU]	Area %
1	7.226	BV	0.1510	518.51685	53.54984	48.9544
2	7.572	VB	0.1468	540.66736	56.92676	51.0456

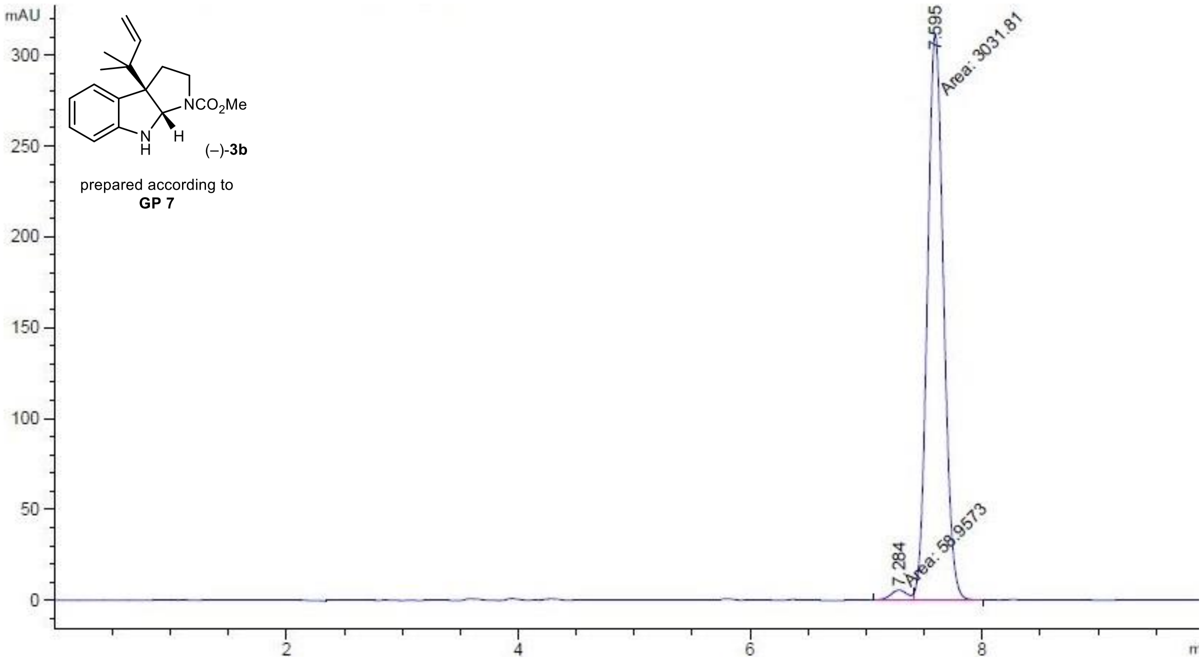

Peak #	RetTime [min]	Type	Width [min]	Area [mAU*s]	Height [mAU]	Area %
1	7.284	MF	0.1746	58.95728	5.62812	1.9075
2	7.595	FM	0.1621	3031.81348	311.74371	98.0925

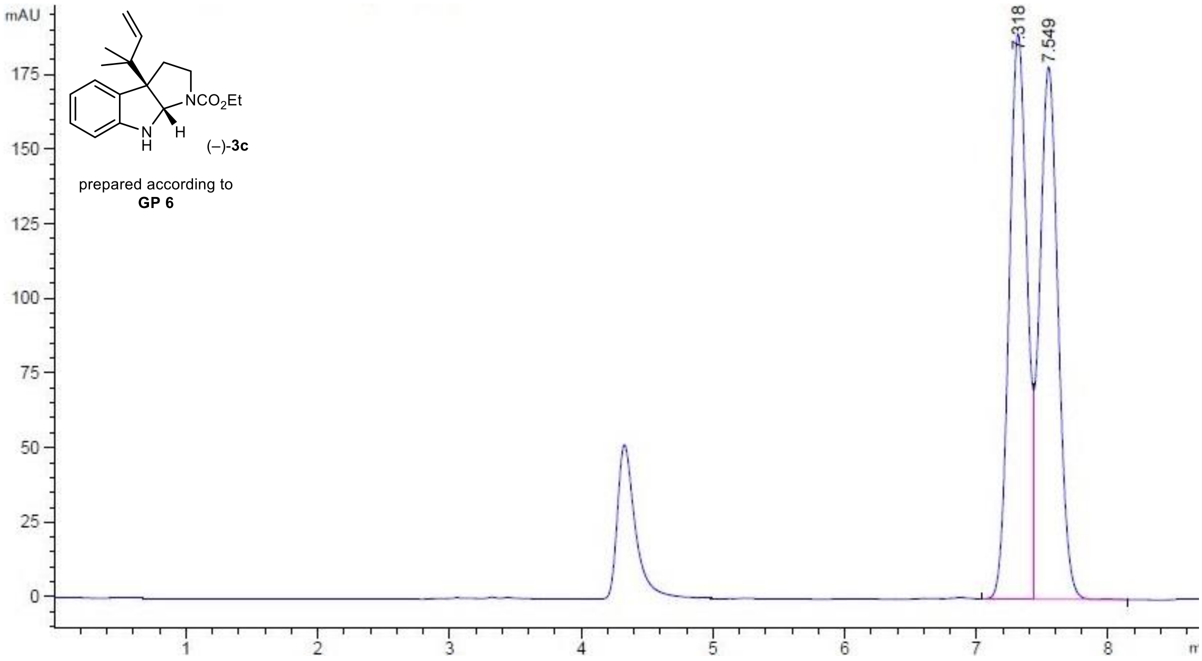

Peak #	RetTime [min]	Type	Width [min]	Area [mAU*s]	Height [mAU]	Area %
1	7.226	BV	0.1510	518.51685	53.54984	48.9544
2	7.572	VB	0.1468	540.66736	56.92676	51.0456

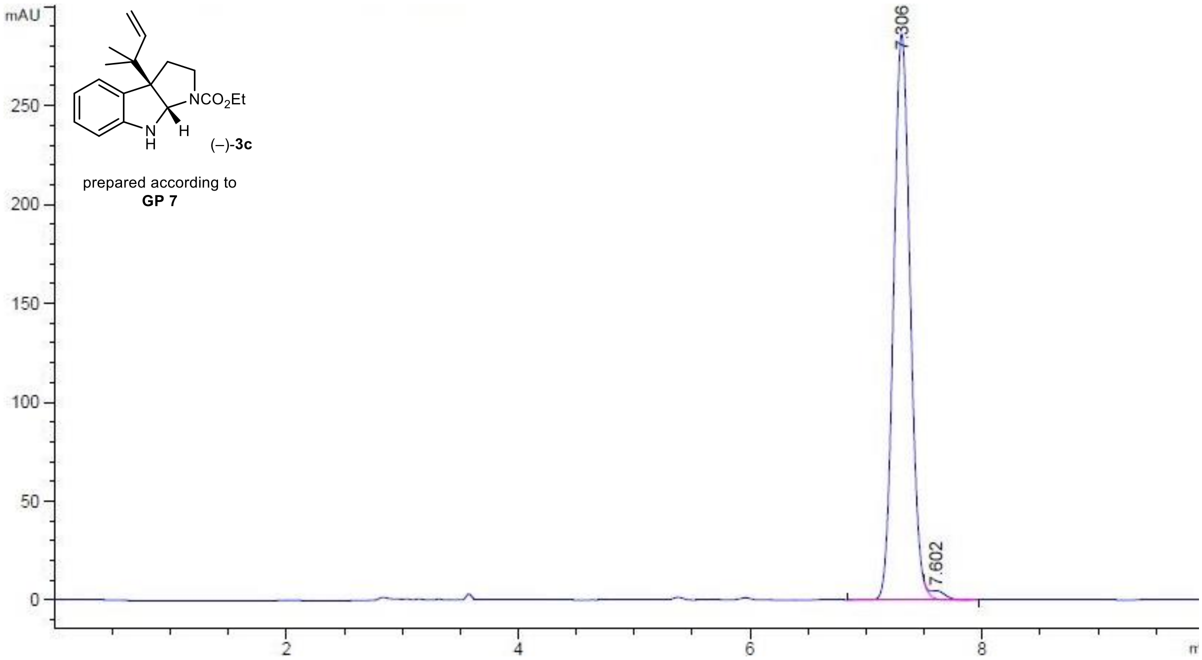

Peak #	RetTime [min]	Type	Width [min]	Area [mAU*s]	Height [mAU]	Area %
1	7.284	MF	0.1746	58.95728	5.62812	1.9075
2	7.595	FM	0.1621	3031.81348	311.74371	98.0925

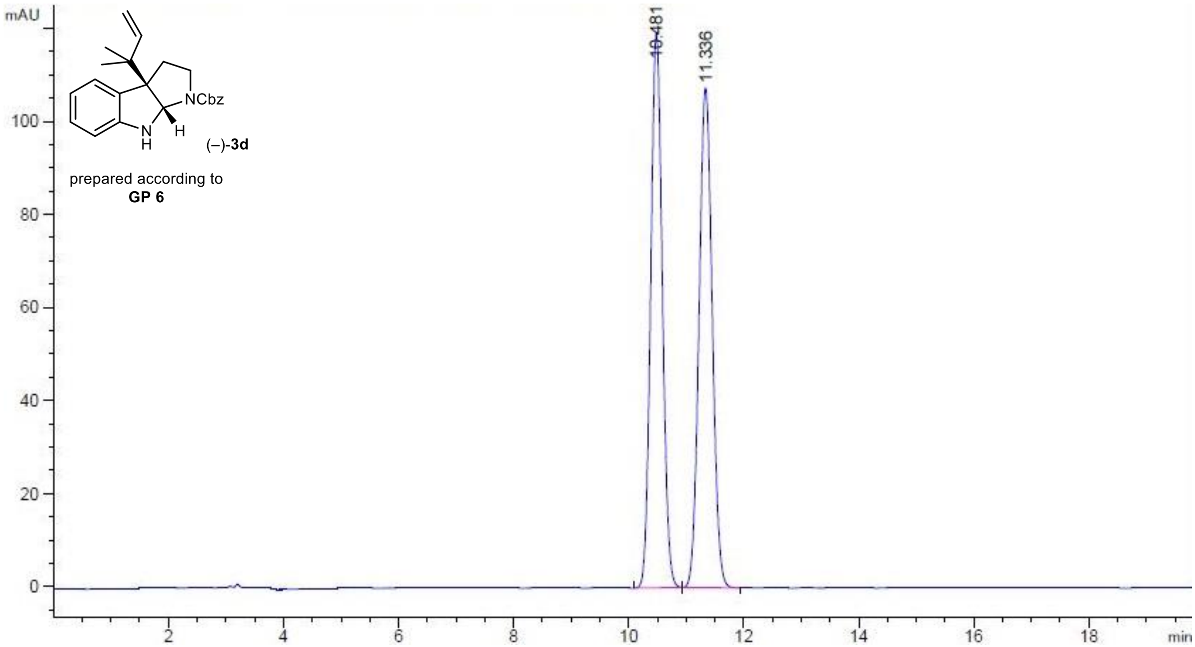

Peak #	RetTime [min]	Type	Width [min]	Area [mAU*s]	Height [mAU]	Area %
1	10.481	BB	0.2177	1672.77637	119.18709	49.4972
2	11.336	BB	0.2479	1706.75854	107.37337	50.5028

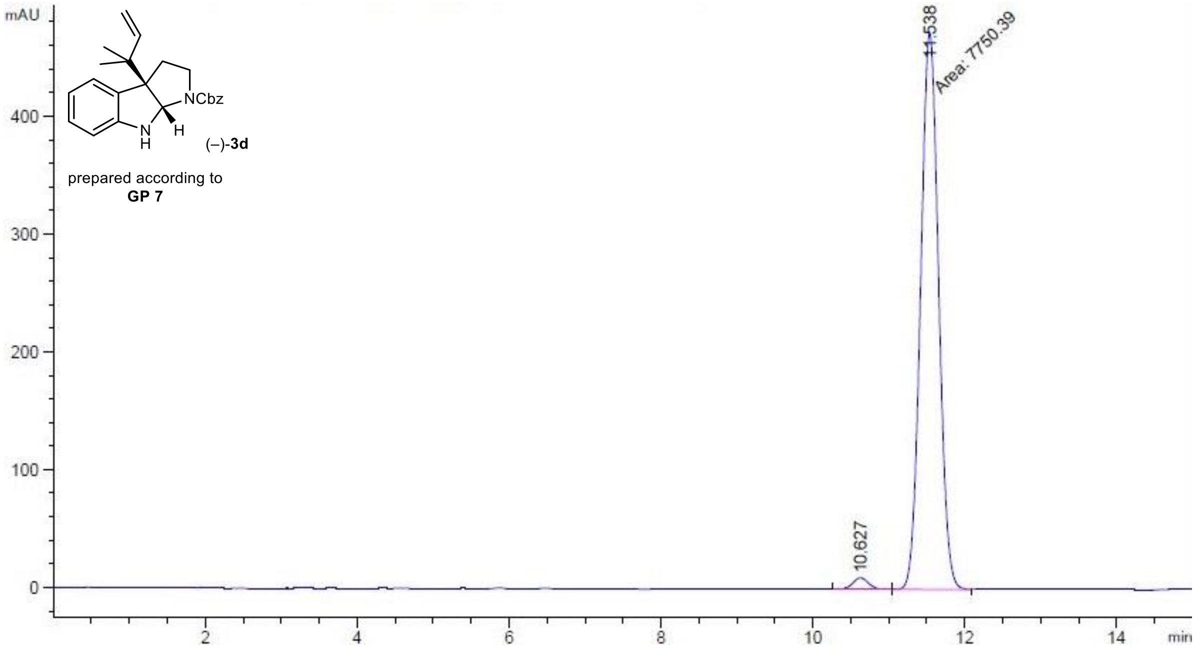

Peak #	RetTime [min]	Type	Width [min]	Area [mAU*s]	Height [mAU]	Area %
1	10.627	BV	0.2214	140.02145	9.78917	1.7746
2	11.538	MF	0.2737	7750.39355	472.01129	98.2254

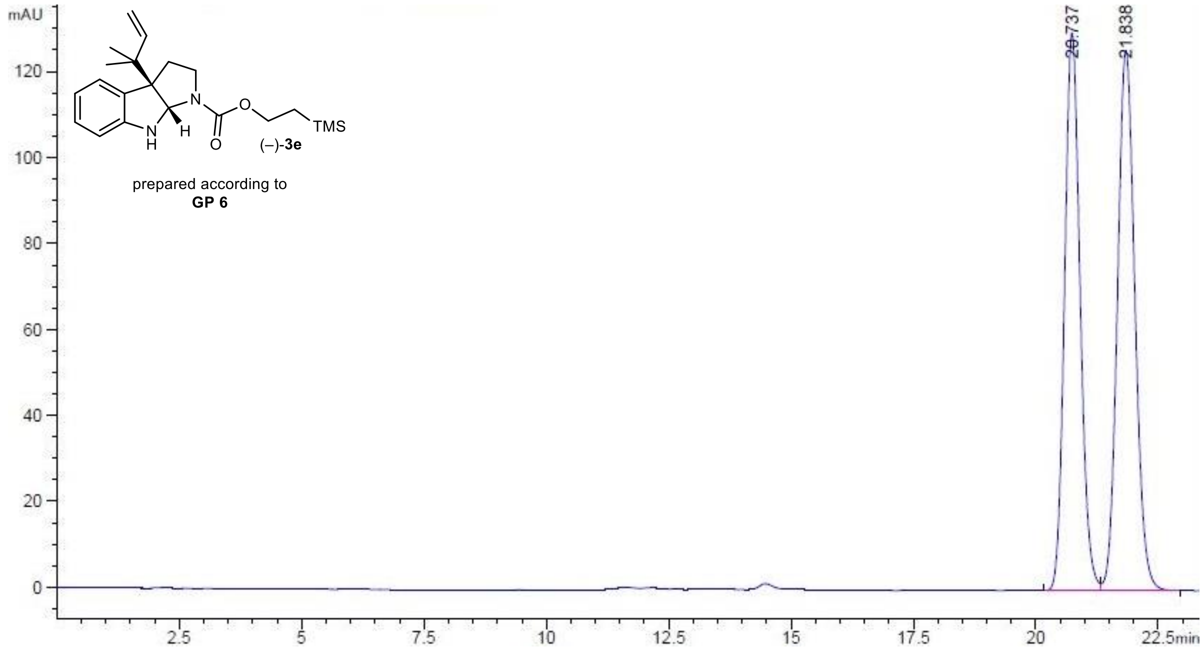

Peak #	RetTime [min]	Type	Width [min]	Area [mAU*s]	Height [mAU]	Area %
1	20.737	BV	0.3390	2859.60767	129.81328	47.7983
2	21.838	VB	0.3752	3123.05322	125.77016	52.2017

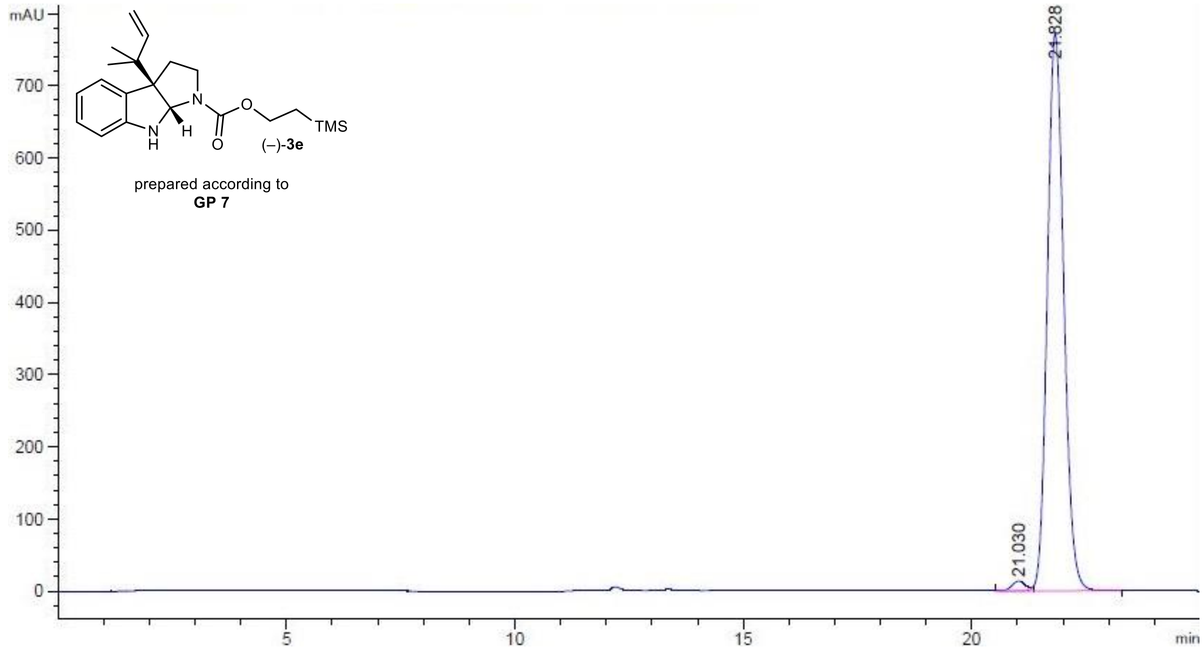

Peak #	RetTime [min]	Type	Width [min]	Area [mAU*s]	Height [mAU]	Area %
1	21.030	BV E	0.2962	279.53476	13.72056	1.4245
2	21.828	VB R	0.3878	1.93434e4	772.07690	98.5755

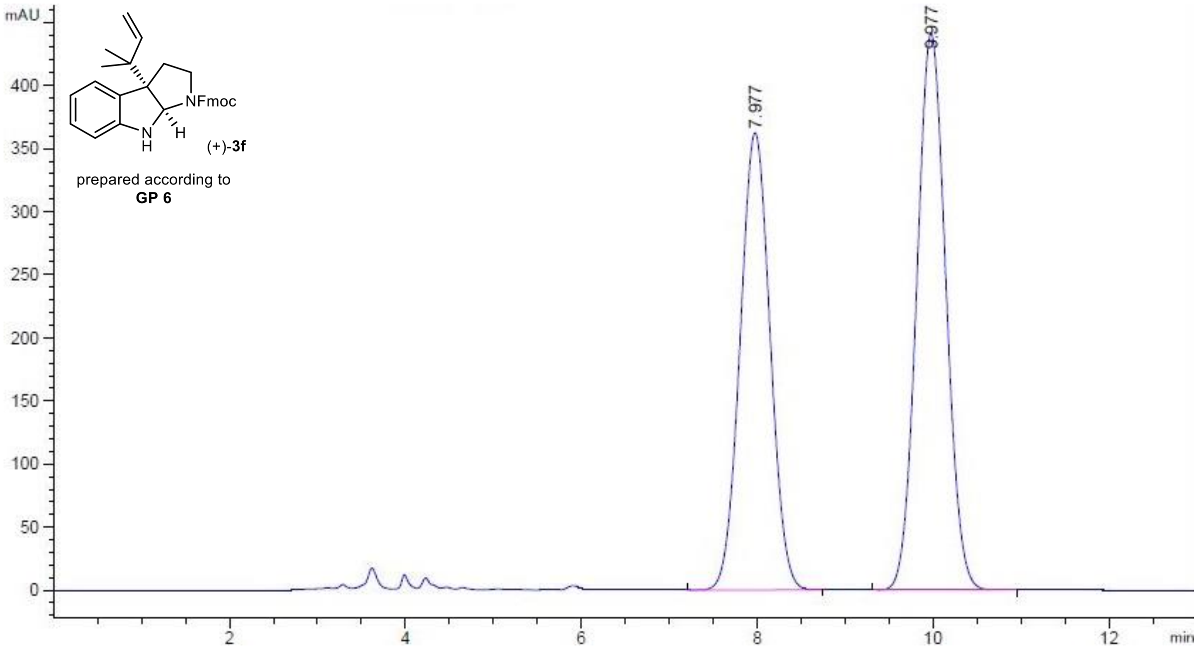

Peak #	RetTime [min]	Type	Width [min]	Area [mAU*s]	Height [mAU]	Area %
1	7.977	BB	0.3713	8578.86230	362.23920	45.8113
2	9.977	BB	0.3594	1.01477e4	441.02328	54.1887

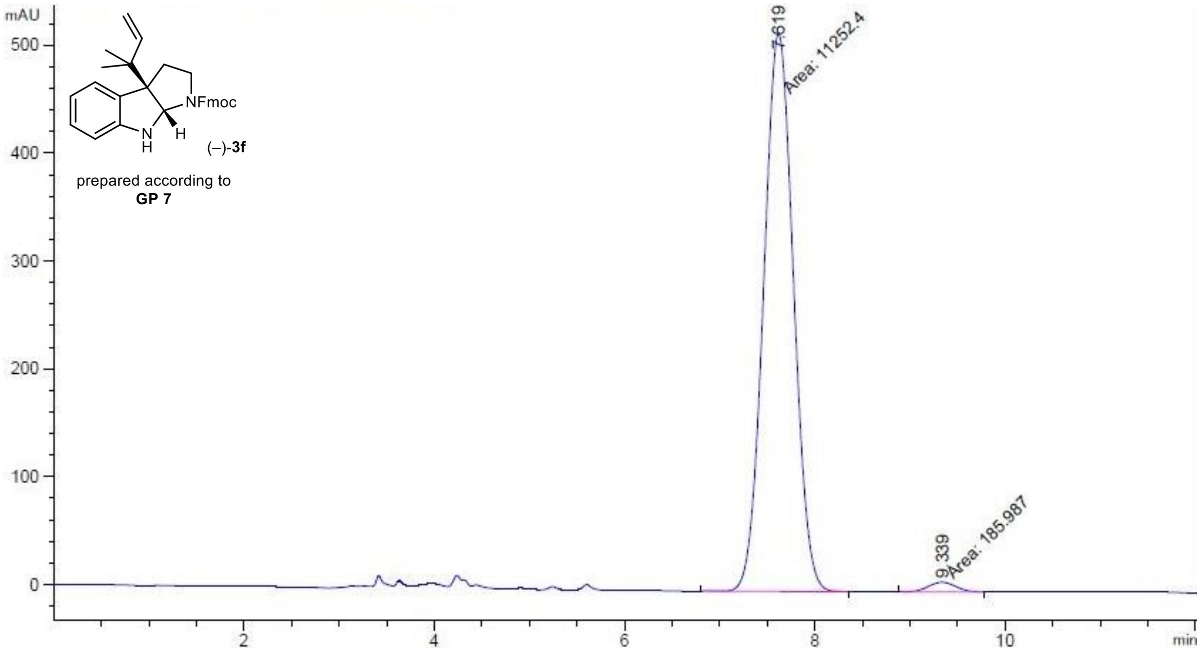

Peak #	RetTime [min]	Type	Width [min]	Area [mAU*s]	Height [mAU]	Area %
1	7.619	MF	0.3625	1.12524e4	517.33636	98.3740
2	9.339	MF	0.3414	185.98697	9.08052	1.6260

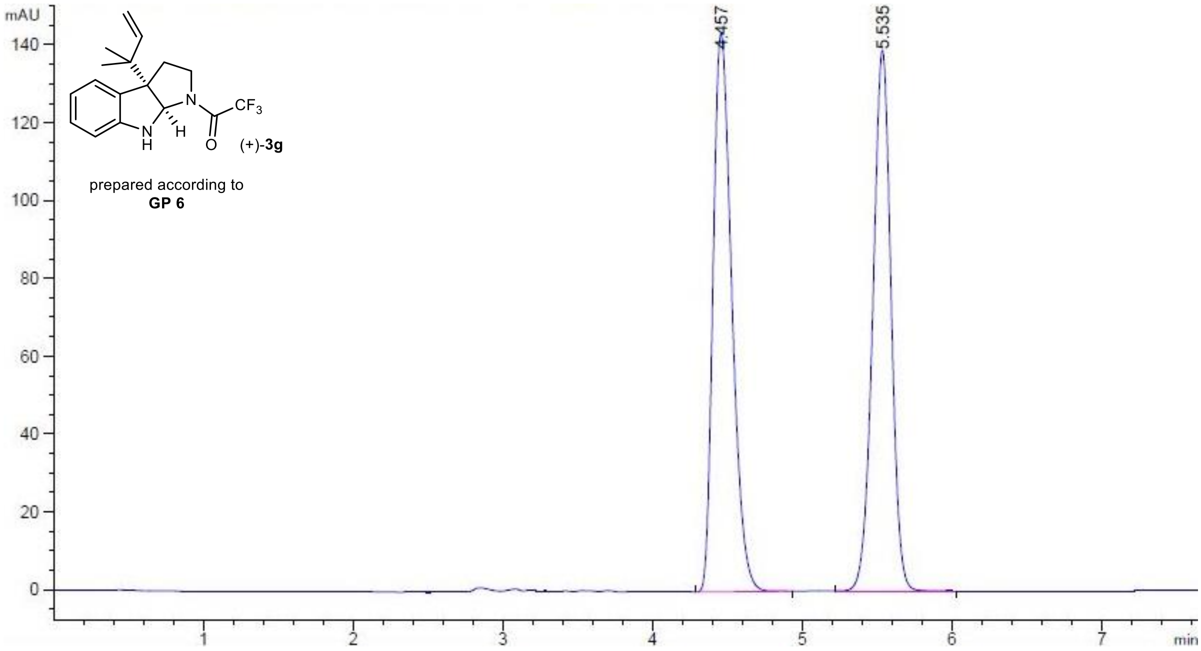

Peak #	RetTime [min]	Type	Width [min]	Area [mAU*s]	Height [mAU]	Area %
1	4.457	BB	0.1347	1244.63770	143.54228	51.4481
2	5.535	BB	0.1301	1174.57202	138.93901	48.5519

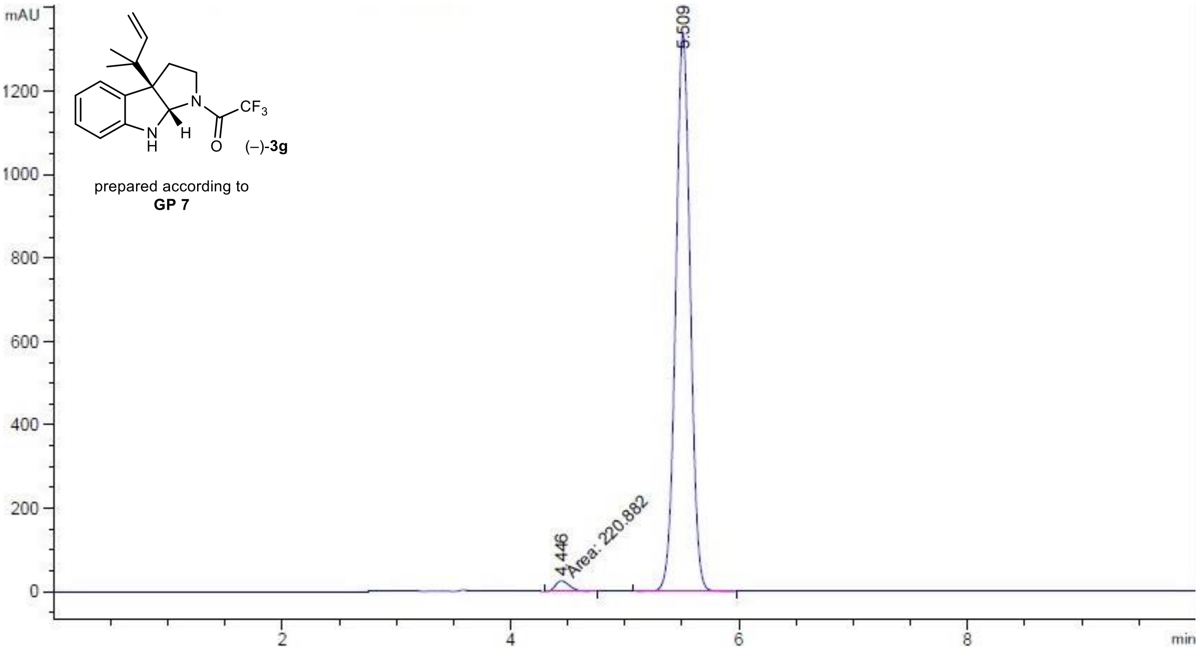

Peak #	RetTime [min]	Type	Width [min]	Area [mAU*s]	Height [mAU]	Area %
1	4.446	MF	0.1450	220.88232	25.39663	1.8321
2	5.509	VB R	0.1367	1.18356e4	1337.54834	98.1679

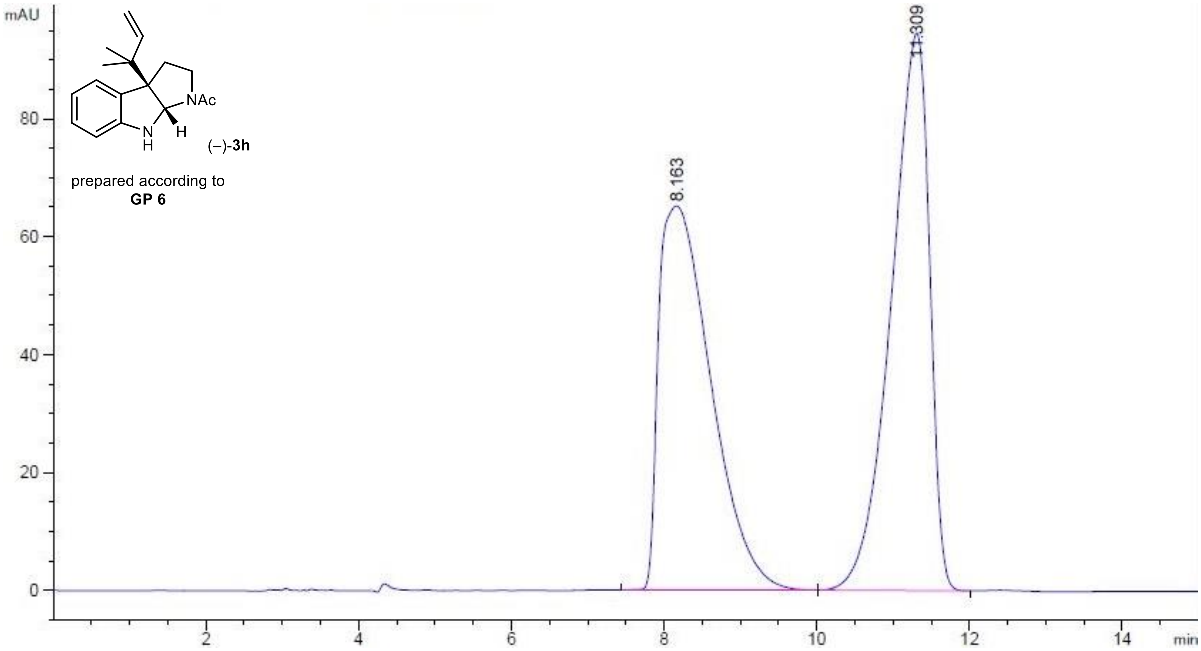

Peak #	RetTime [min]	Type	Width [min]	Area [mAU*s]	Height [mAU]	Area %
1	8.163	BB	0.7422	3094.01978	65.13514	48.4481
2	11.309	BB	0.5372	3292.23364	94.55968	51.5519

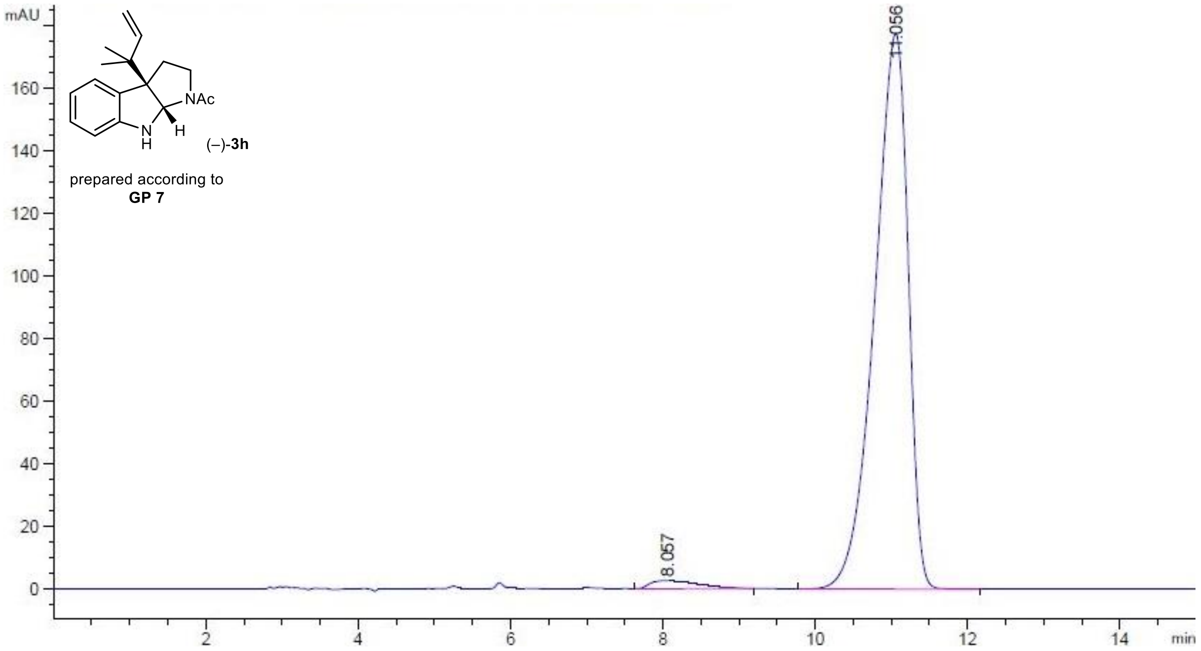

Peak #	RetTime [min]	Type	Width [min]	Area [mAU*s]	Height [mAU]	Area %
1	8.057	BB	0.5260	114.02225	2.53846	2.0622
2	11.056	BB	0.4719	5415.22363	177.53702	97.9378

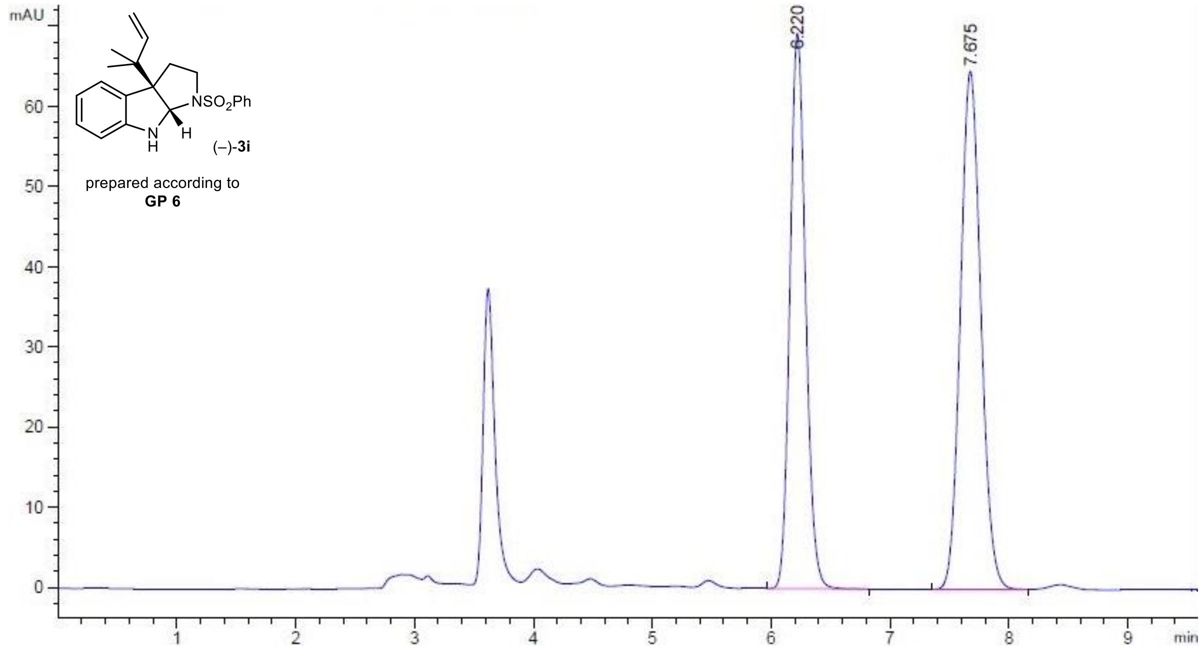

Peak #	RetTime [min]	Type	Width [min]	Area [mAU*s]	Height [mAU]	Area %
1	6.220	BB	0.1418	633.74286	69.22653	45.0490
2	7.675	BB	0.1861	773.04376	64.59293	54.9510

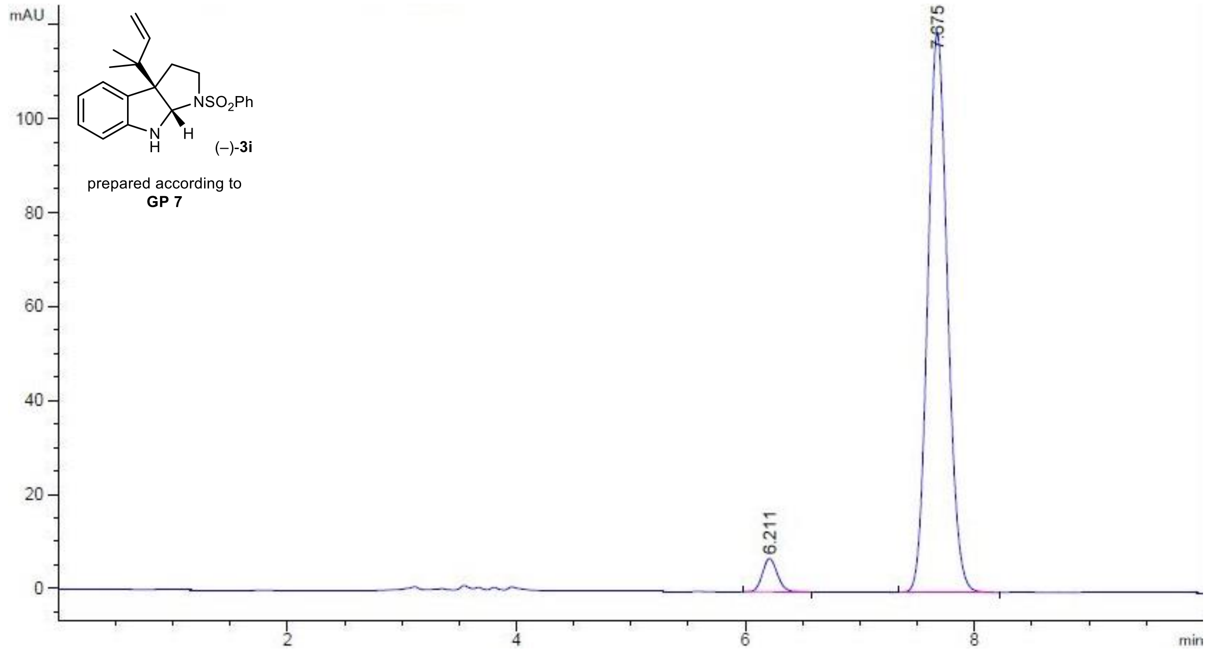

Peak #	RetTime [min]	Type	Width [min]	Area [mAU*s]	Height [mAU]	Area %
1	6.211	BB	0.1374	64.47101	7.09954	4.3291
2	7.675	BB	0.1862	1424.78088	118.96921	95.6709

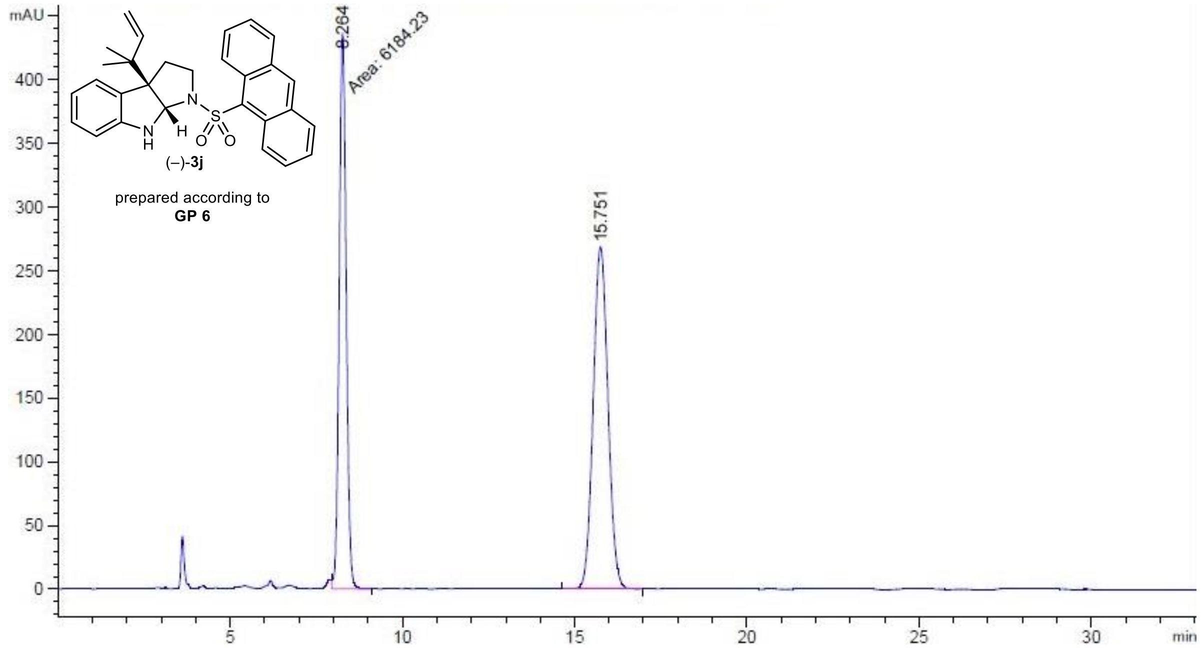

Peak #	RetTime [min]	Type	Width [min]	Area [mAU*s]	Height [mAU]	Area %
1	8.264	MM	0.2364	6184.23145	435.97610	43.5532
2	15.751	BB	0.4632	8015.01123	268.56180	56.4468

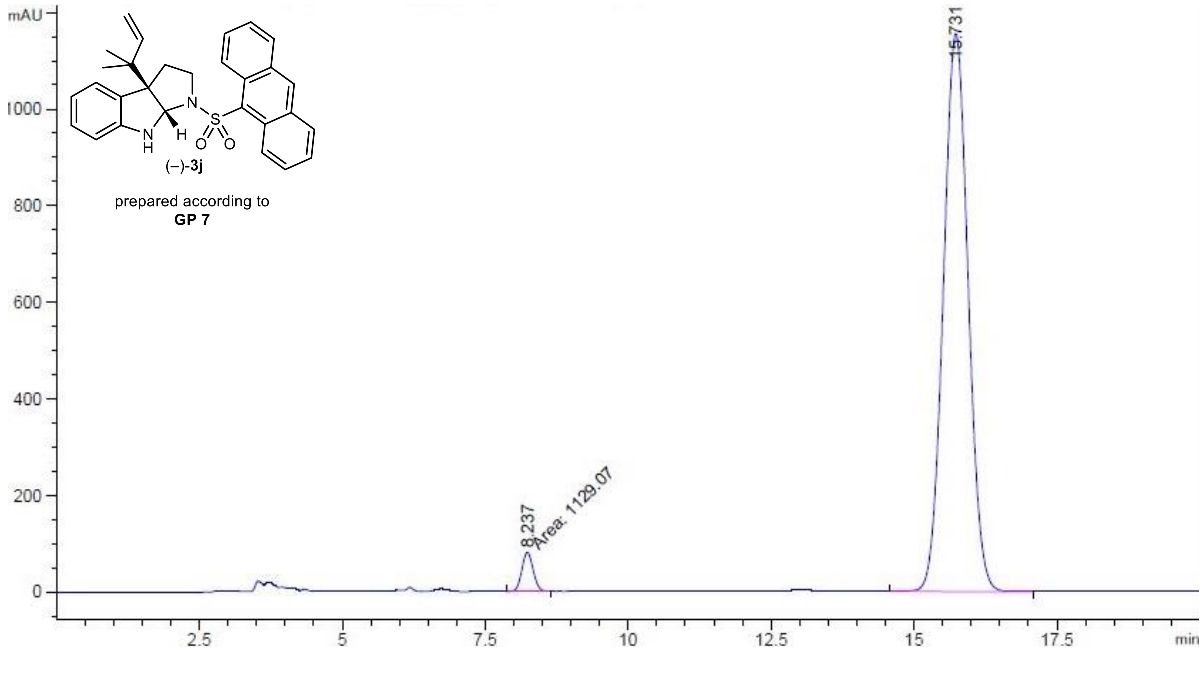

Peak #	RetTime [min]	Type	Width [min]	Area [mAU*s]	Height [mAU]	Area %
1	8.237	MF	0.2315	1129.06799	81.27500	3.1525
2	15.731	BB	0.4698	3.46857e4	1155.36560	96.8475

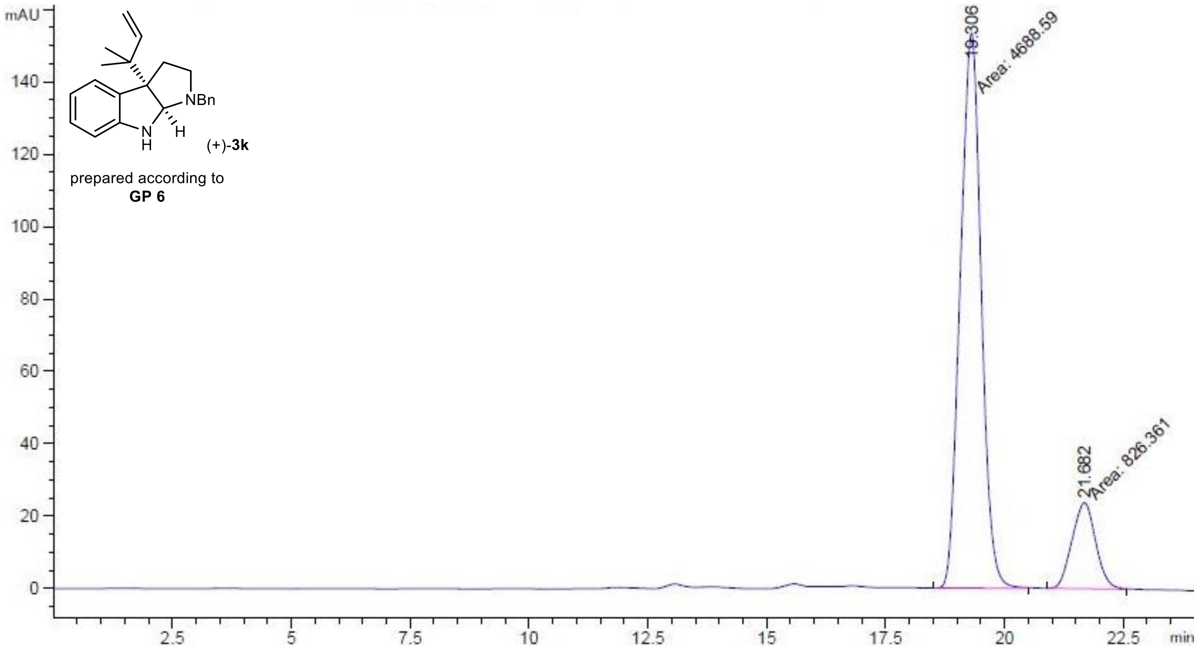

Peak #	RetTime [min]	Type	Width [min]	Area [mAU*s]	Height [mAU]	Area %
1	19.306	MF	0.5098	4688.59277	153.26848	85.0160
2	21.682	MF	0.5796	826.36053	23.76372	14.9840

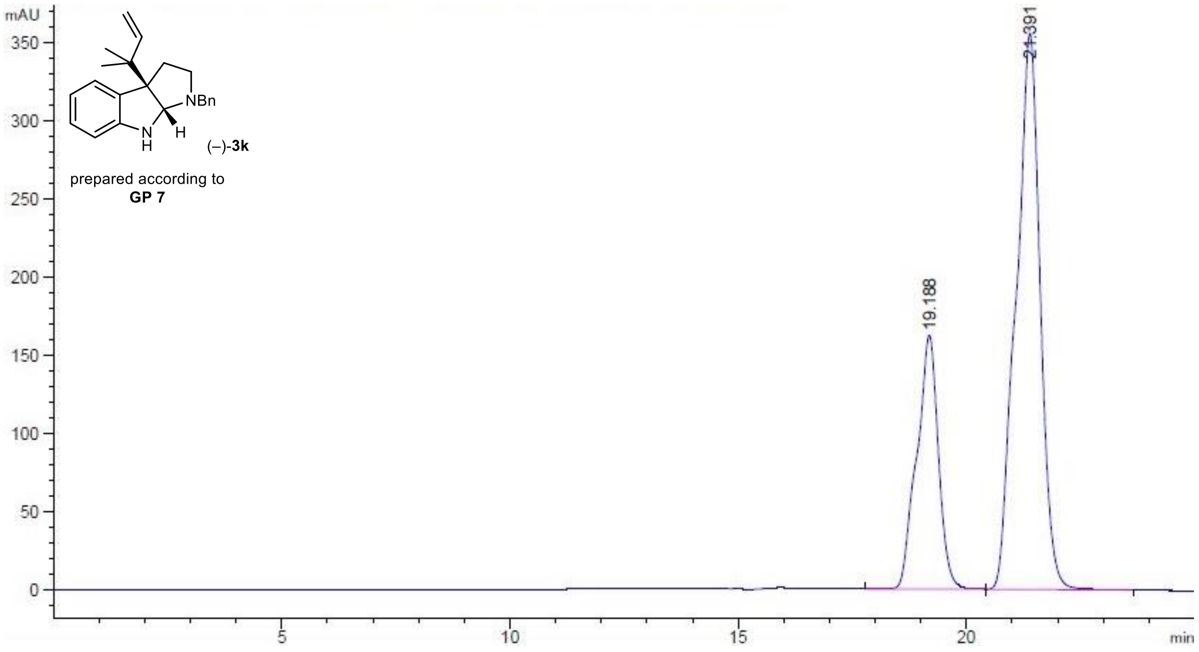

Peak #	RetTime [min]	Type	Width [min]	Area [mAU*s]	Height [mAU]	Area %
1	19.188	BB	0.4605	5344.01416	162.36124	29.0837
2	21.391	BB	0.5221	1.30306e4	355.55569	70.9163

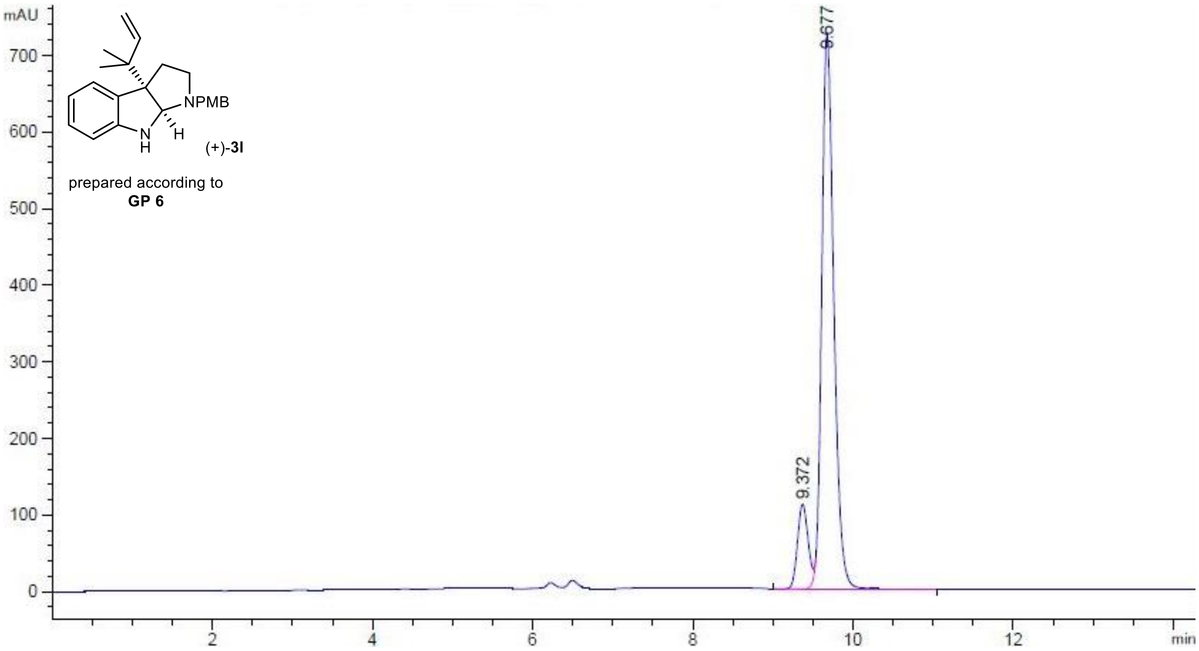

Peak #	RetTime [min]	Type	Width [min]	Area [mAU*s]	Height [mAU]	Area %
1	9.372	BV E	0.1432	1015.55823	110.54952	11.7735
2	9.677	VB R	0.1609	7610.23975	725.09705	88.2265

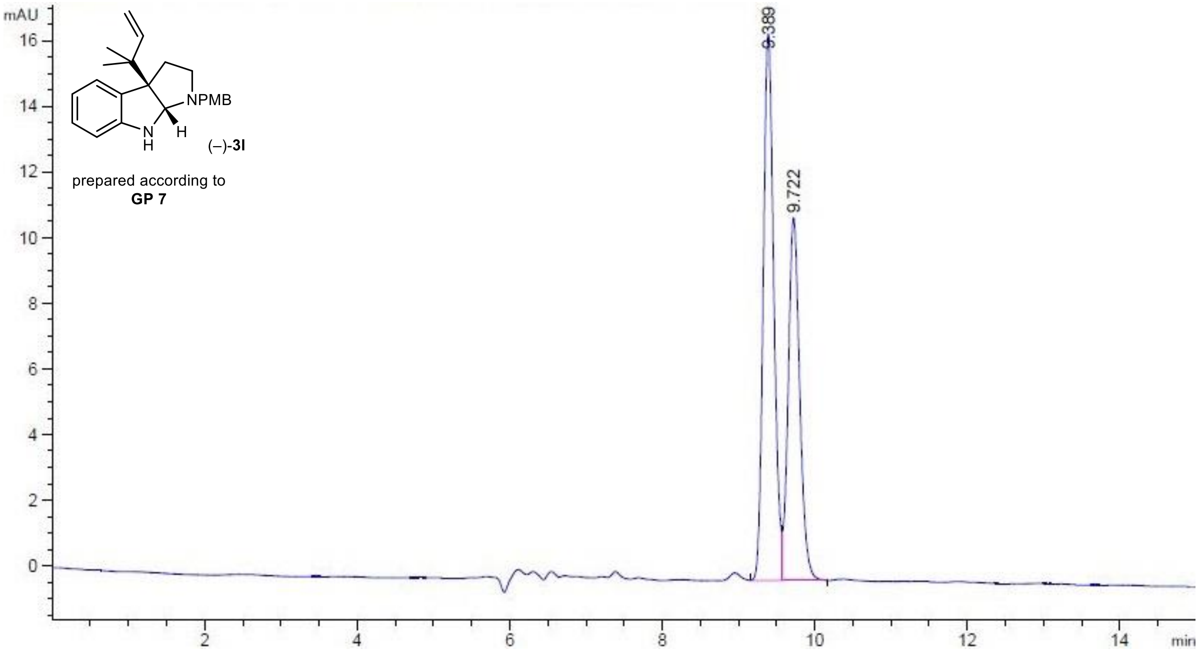

Peak #	RetTime [min]	Type	Width [min]	Area [mAU*s]	Height [mAU]	Area %
1	9.389	VV	0.1467	159.45633	16.65785	57.8667
2	9.722	VB	0.1566	116.10158	11.05140	42.1333

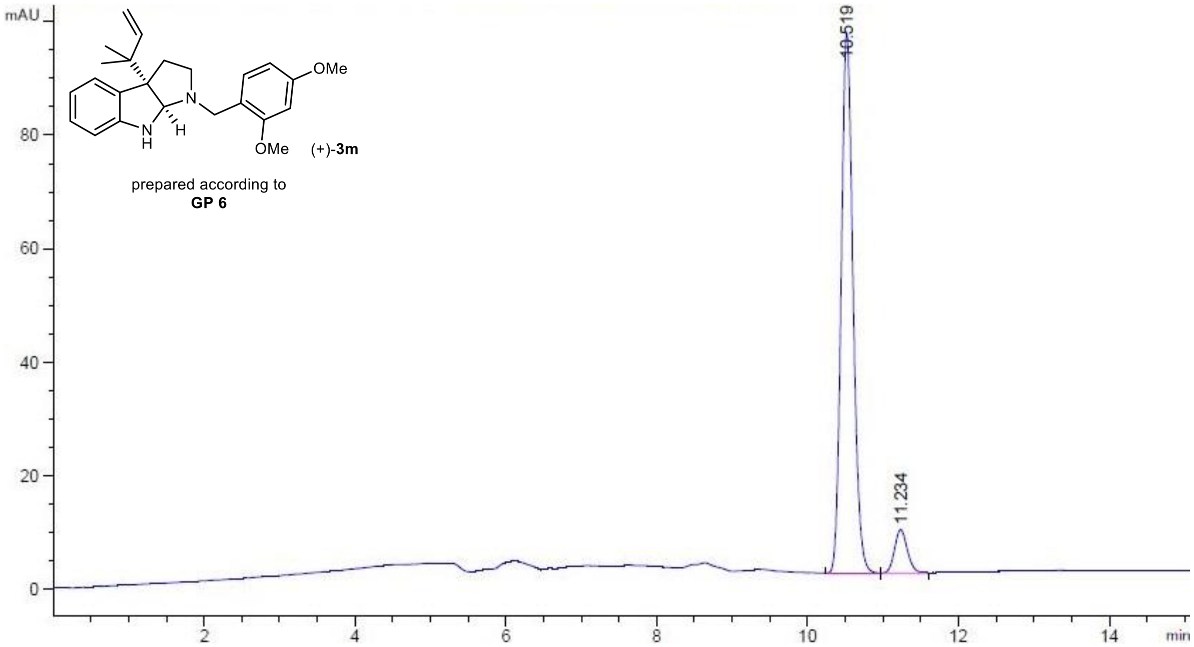

Peak #	RetTime [min]	Type	Width [min]	Area [mAU*s]	Height [mAU]	Area %
1	10.519	BV	0.1684	1037.55261	95.02978	92.0536
2	11.234	VB	0.1734	89.56503	7.63476	7.9464

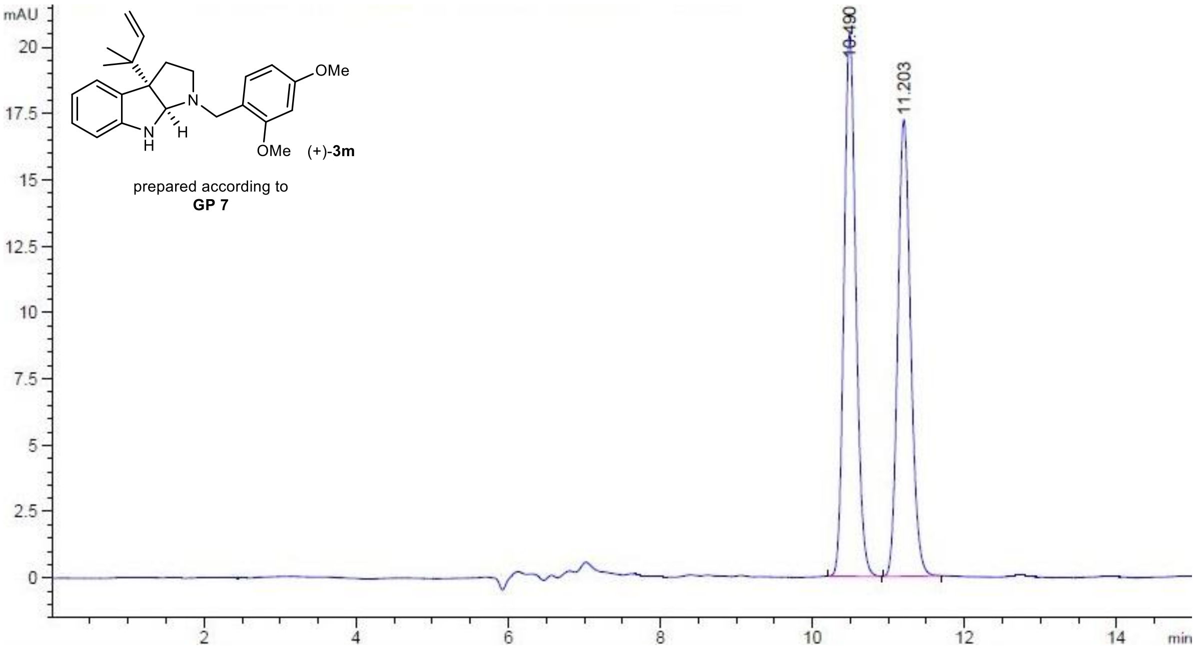

Peak #	RetTime [min]	Type	Width [min]	Area [mAU*s]	Height [mAU]	Area %
1	10.490	BB	0.1656	222.13876	20.47440	52.2544
2	11.203	BB	0.1785	202.97118	17.21859	47.7456

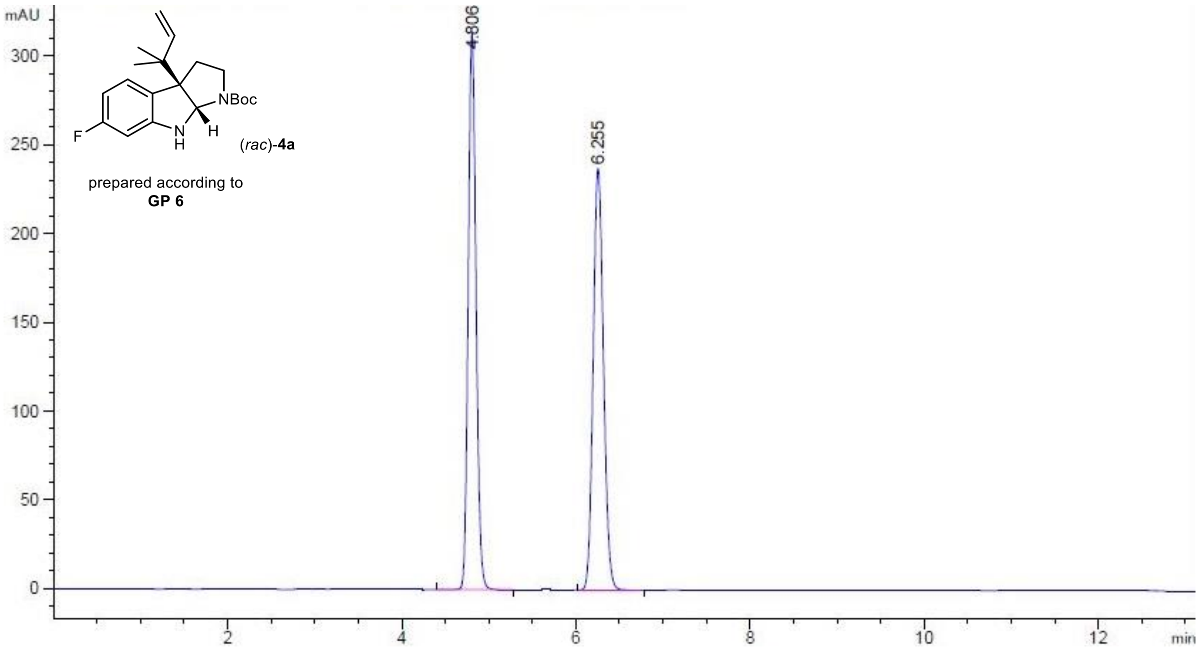

Peak #	RetTime [min]	Type	Width [min]	Area [mAU*s]	Height [mAU]	Area %
1	4.806	VB R	0.0969	1959.56018	313.38684	50.0533
2	6.255	BB	0.1276	1955.38416	237.48613	49.9467

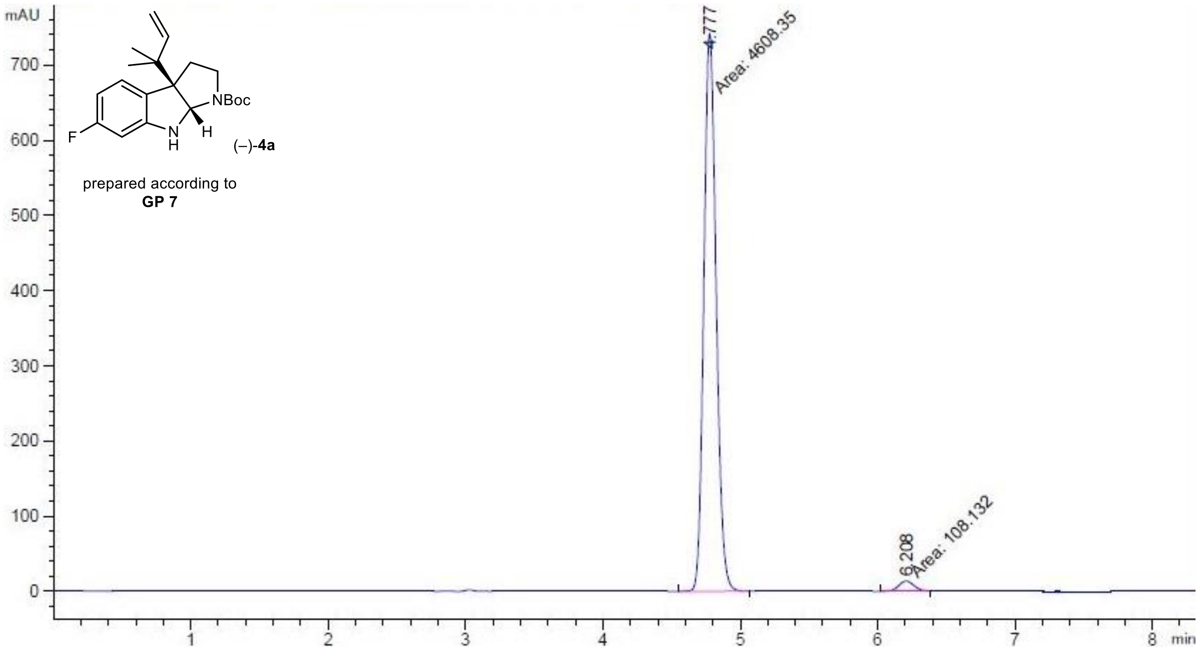

Peak #	RetTime [min]	Type	Width [min]	Area [mAU*s]	Height [mAU]	Area %
1	4.777	FM	0.1035	4608.34570	741.75519	97.7074
2	6.208	MF	0.1320	108.13179	13.65246	2.2926

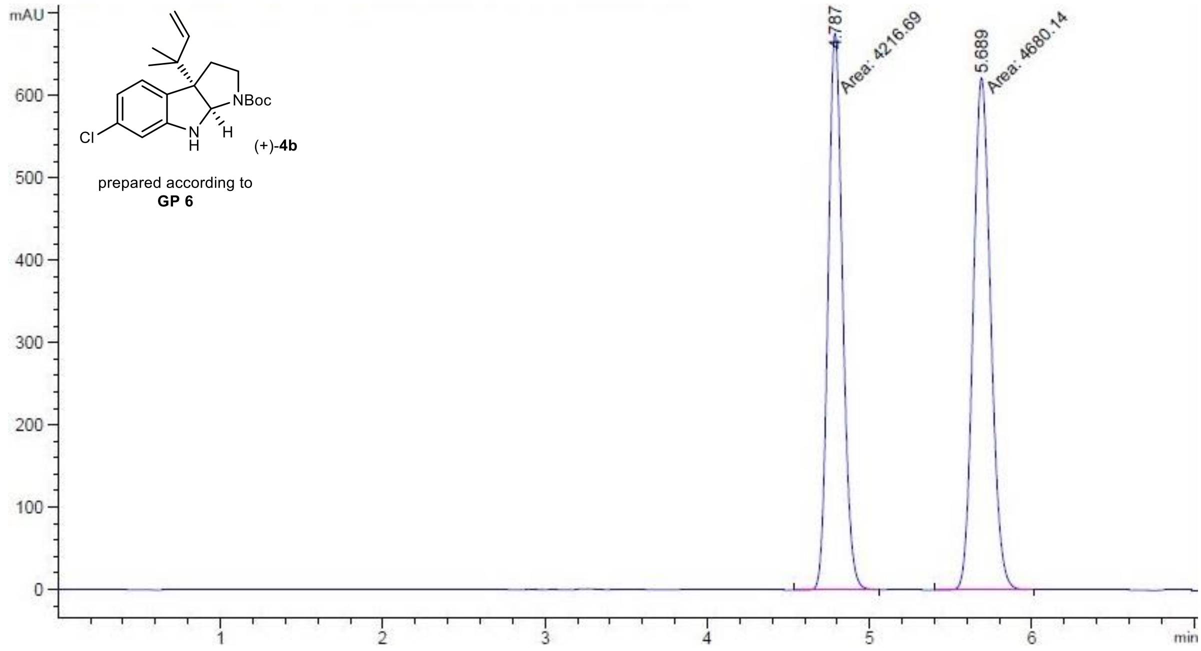

Peak #	RetTime [min]	Type	Width [min]	Area [mAU*s]	Height [mAU]	Area %
1	4.787	MF	0.1039	4216.68994	676.39514	47.3954
2	5.689	MF	0.1253	4680.13818	622.35638	52.6046

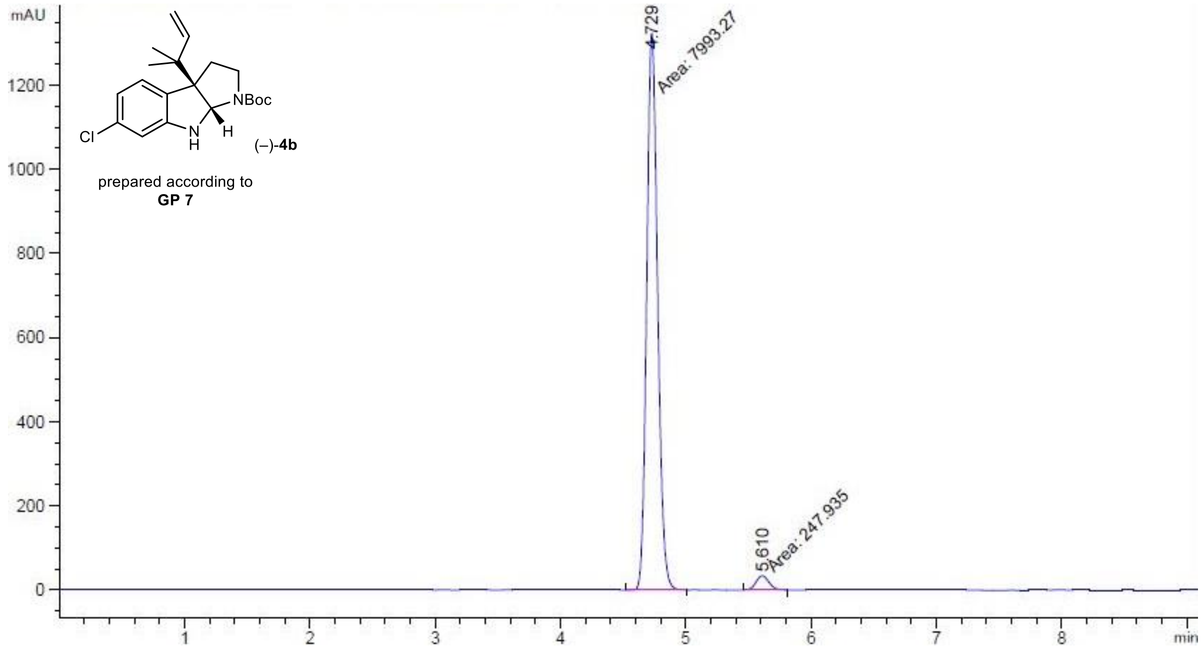

Peak #	RetTime [min]	Type	Width [min]	Area [mAU*s]	Height [mAU]	Area %
1	4.729	MF	0.1007	7993.27490	1323.19812	96.9915
2	5.610	MF	0.1204	247.93524	34.32889	3.0085

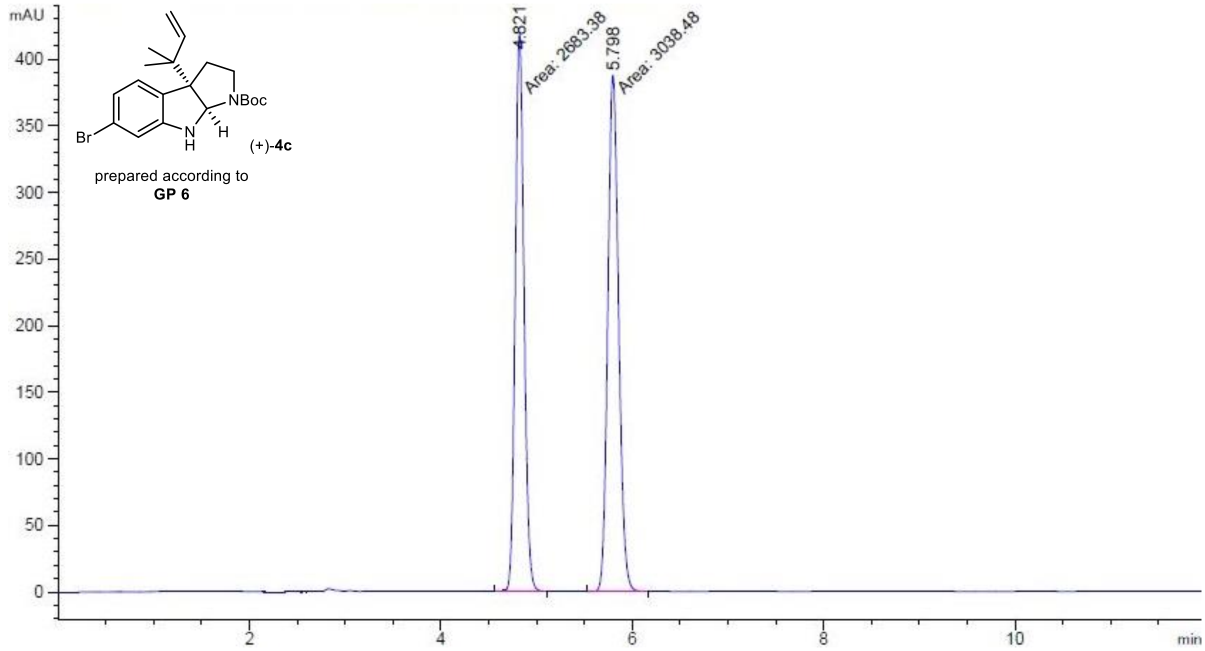

Peak #	RetTime [min]	Type	Width [min]	Area [mAU*s]	Height [mAU]	Area %
1	4.821	MF	0.1067	2683.37939	419.11984	46.8970
2	5.798	MF	0.1306	3038.47949	387.65286	53.1030

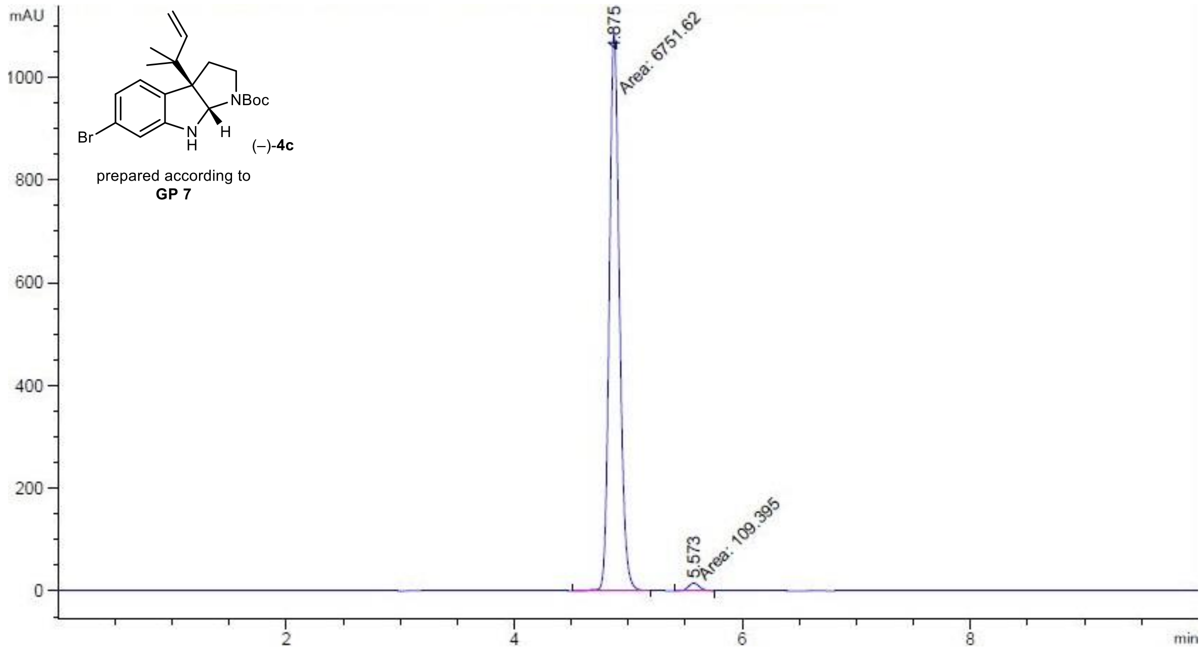

Peak #	RetTime [min]	Type	Width [min]	Area [mAU*s]	Height [mAU]	Area %
1	4.875	MF	0.1038	6751.62061	1084.19421	98.4056
2	5.573	MF	0.1187	109.39527	15.36544	1.5944

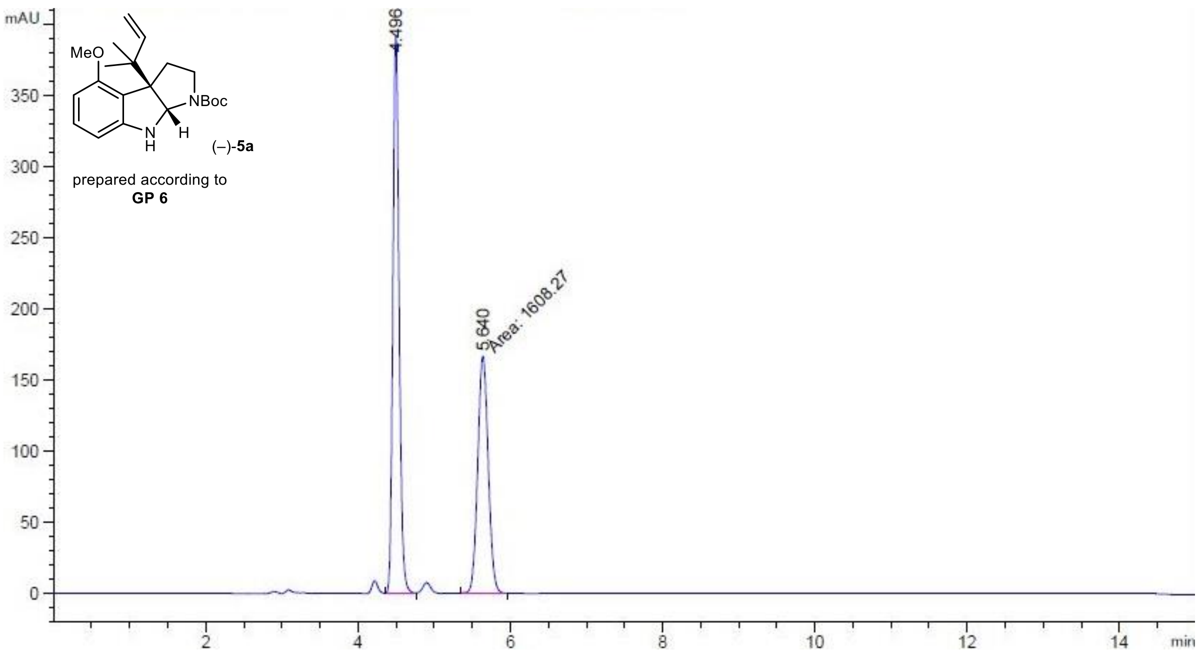

Peak #	RetTime [min]	Type	Width [min]	Area [mAU*s]	Height [mAU]	Area %
1	4.496	VV	0.0876	2238.79565	391.89224	58.1949
2	5.640	MF	0.1604	1608.27112	167.10759	41.8051

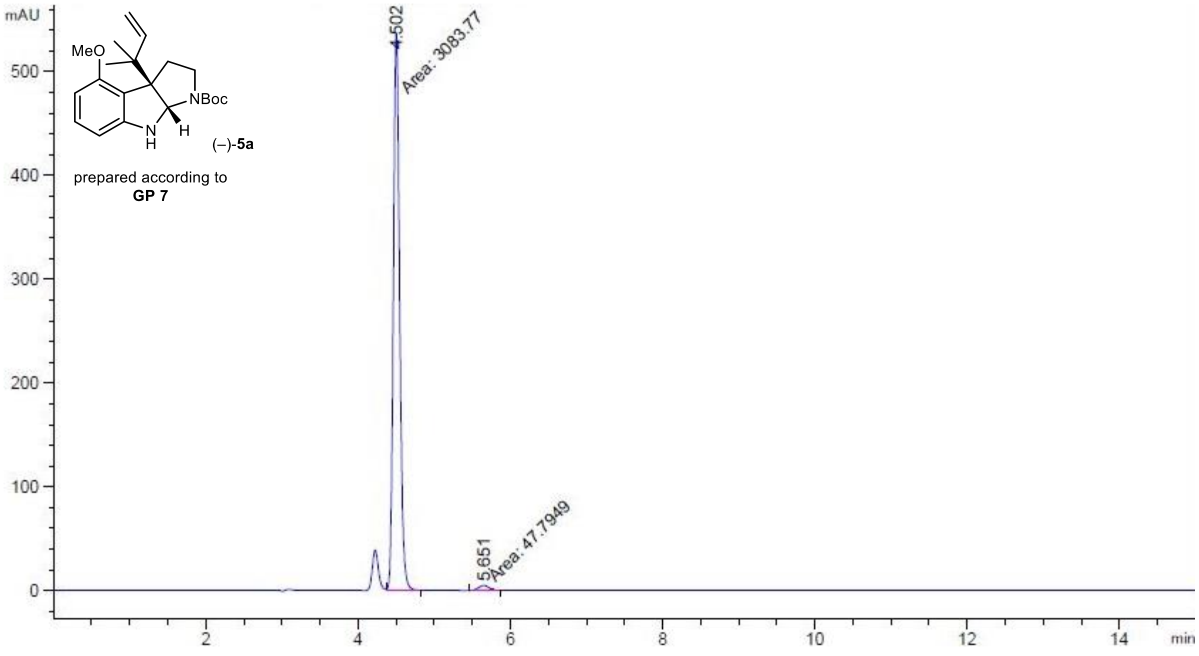

Peak #	RetTime [min]	Type	Width [min]	Area [mAU*s]	Height [mAU]	Area %
1	4.502	MF	0.0958	3083.77319	536.72101	98.4738
2	5.651	MF	0.1595	47.79491	4.99462	1.5262

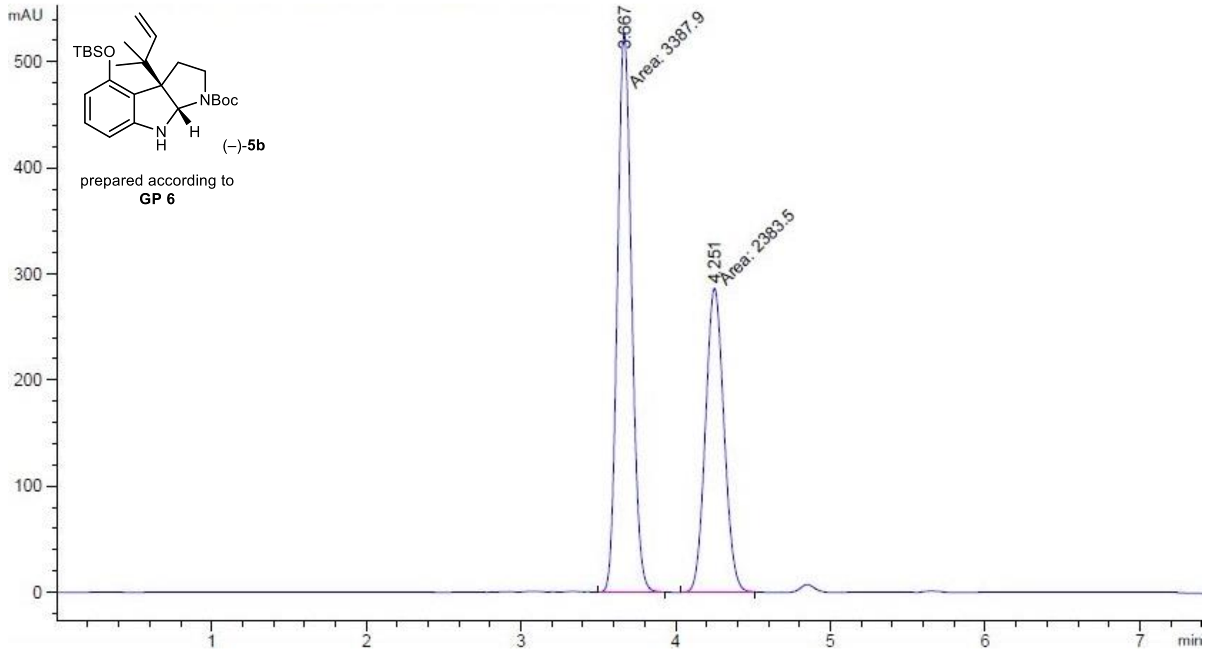

Peak #	RetTime [min]	Type	Width [min]	Area [mAU*s]	Height [mAU]	Area %
1	3.667	MF	0.1072	3387.89746	526.57062	58.7015
2	4.251	MF	0.1387	2383.50220	286.50870	41.2985

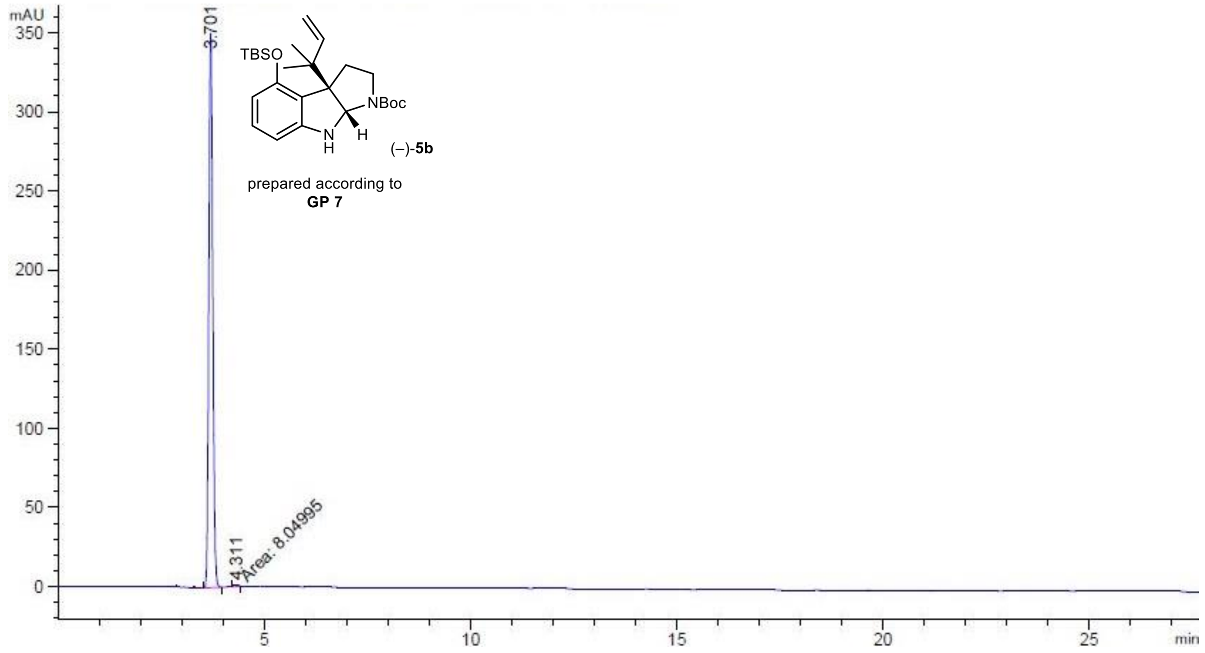

Peak #	RetTime [min]	Type	Width [min]	Area [mAU*s]	Height [mAU]	Area %
1	3.701	BB	0.1038	2334.54736	350.06018	99.6564
2	4.311	MM	0.1299	8.04995	1.03318	0.3436

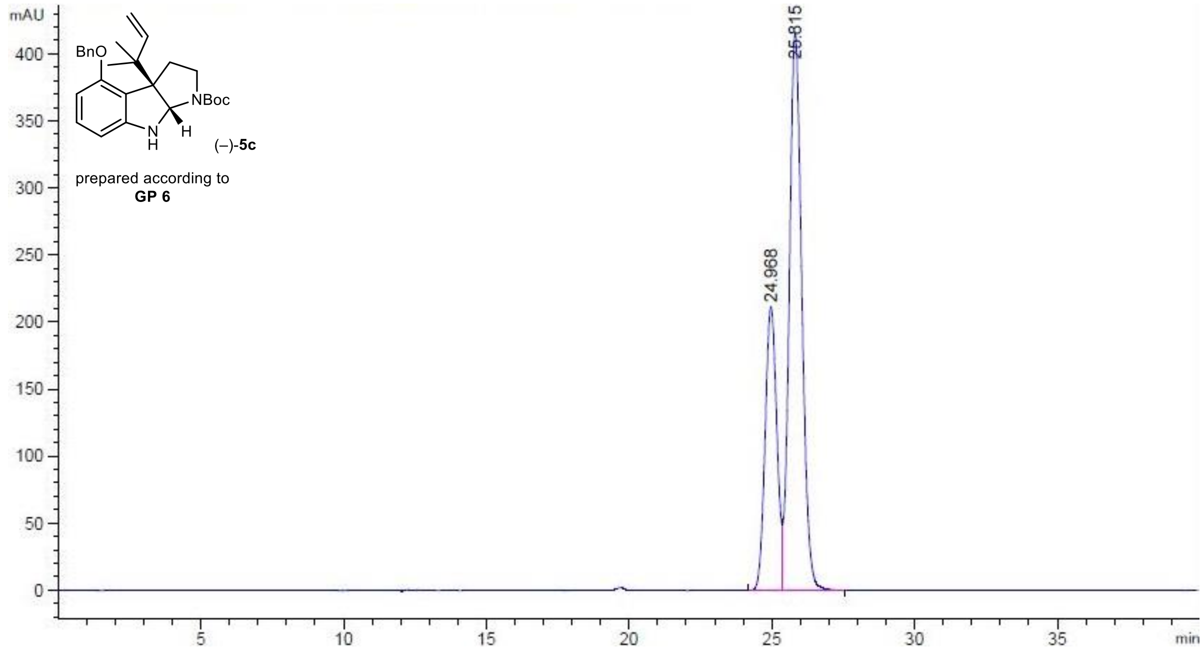

Peak #	RetTime [min]	Type	Width [min]	Area [mAU*s]	Height [mAU]	Area %
1	24.968	BV	0.4399	6010.67627	211.57755	32.0675
2	25.815	VB	0.4677	1.27332e4	415.33994	67.9325

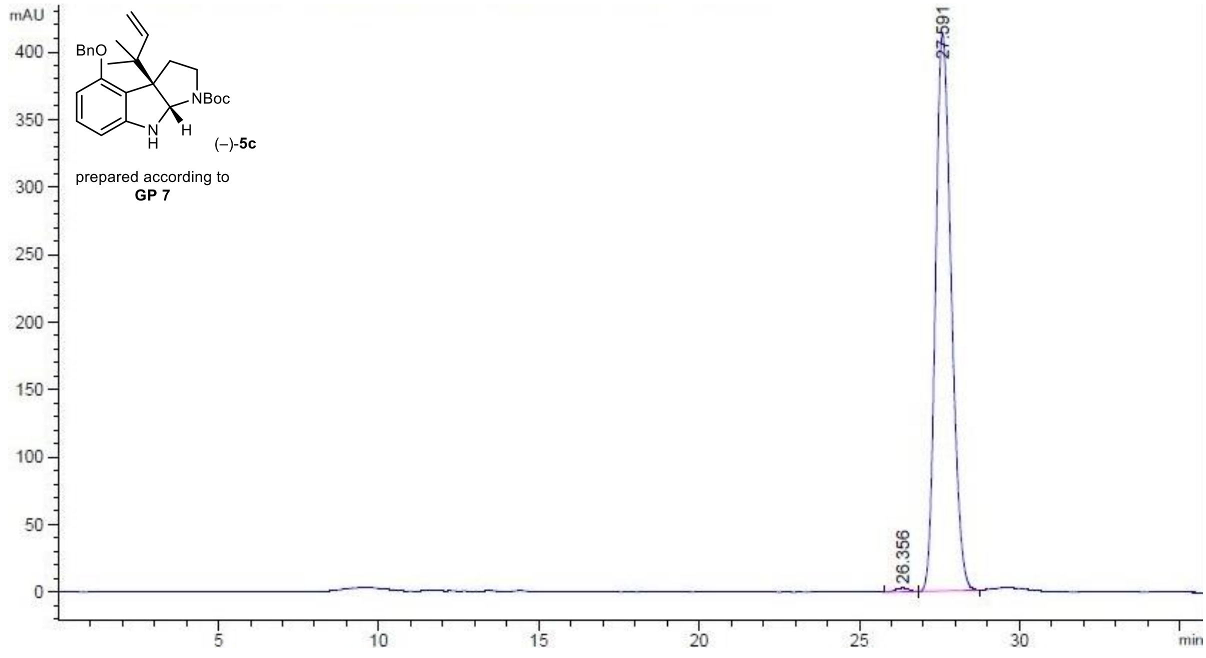

Peak #	RetTime [min]	Type	Width [min]	Area [mAU*s]	Height [mAU]	Area %
1	26.356	BB	0.3297	79.80290	2.84147	0.5616
2	27.591	BB	0.5223	1.41297e4	412.81949	99.4384

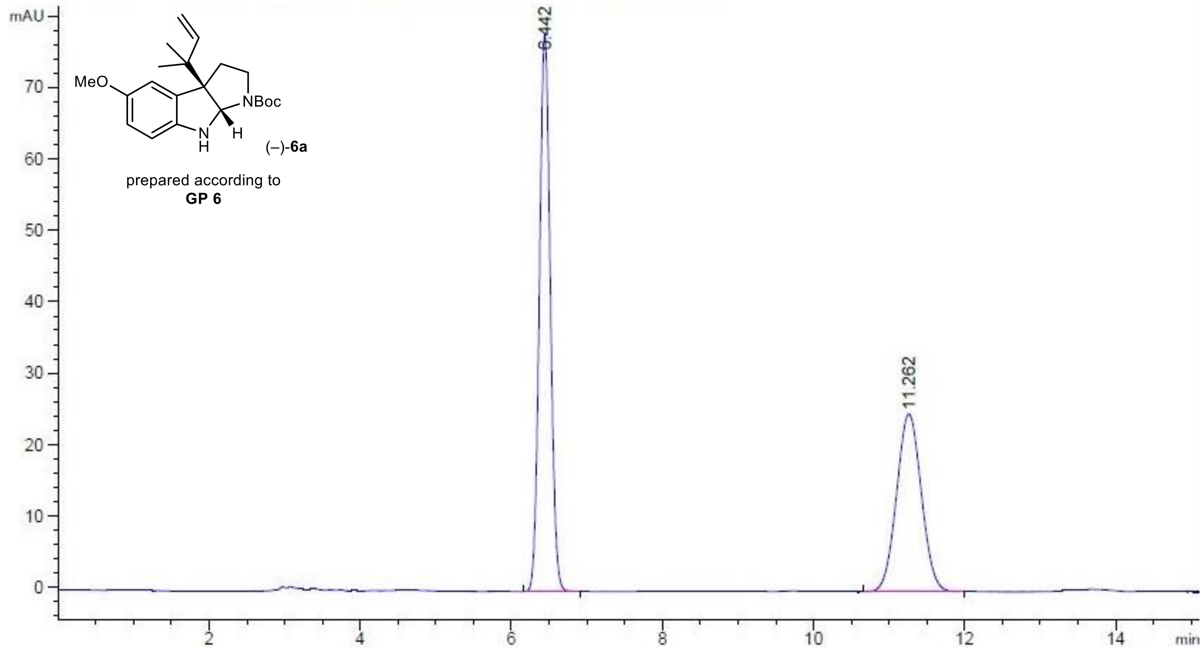

Peak #	RetTime [min]	Type	Width [min]	Area [mAU*s]	Height [mAU]	Area %
1	6.442	BB	0.1561	782.84637	77.98118	57.9262
2	11.262	BB	0.3429	568.60944	24.90454	42.0738

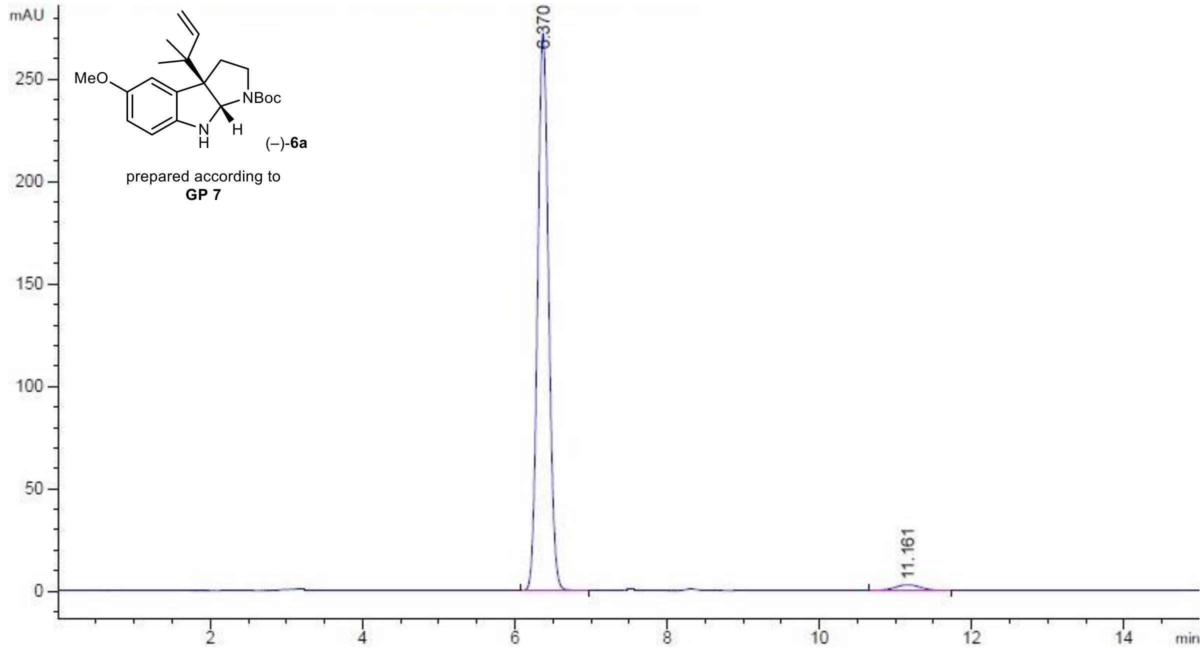

Peak #	RetTime [min]	Type	Width [min]	Area [mAU*s]	Height [mAU]	Area %
1	6.370	BB	0.1561	2717.45630	271.93881	97.7228
2	11.161	BB	0.2679	63.32289	2.78841	2.2772

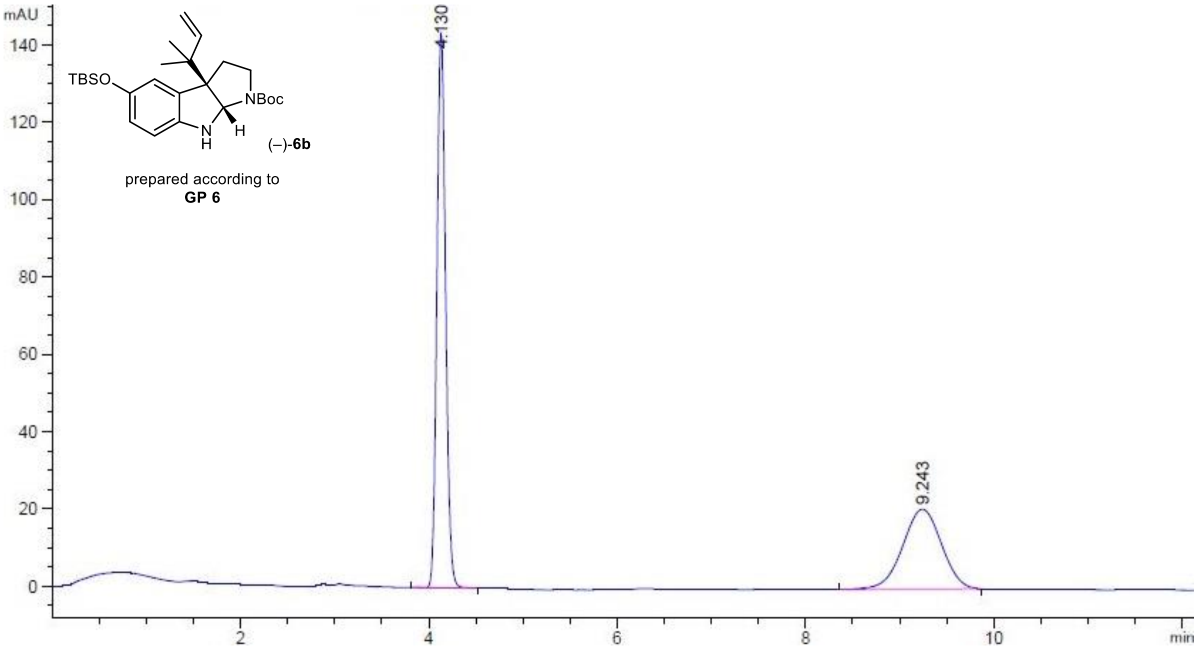

Peak #	RetTime [min]	Type	Width [min]	Area [mAU*s]	Height [mAU]	Area %
1	4.130	VB R	0.0995	922.29700	143.45454	60.9777
2	9.243	BB	0.4015	590.21741	20.61618	39.0223

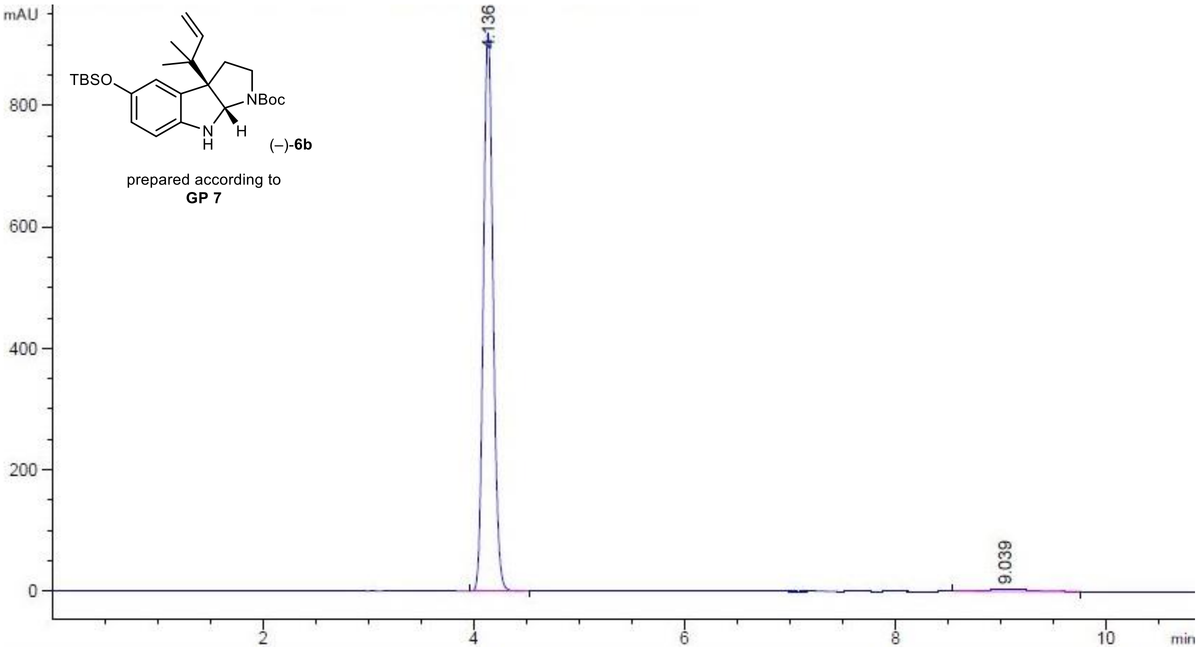

Peak #	RetTime [min]	Type	Width [min]	Area [mAU*s]	Height [mAU]	Area %
1	4.136	BB	0.0996	5879.97510	919.47052	98.3475
2	9.039	BB	0.2819	98.80160	4.37674	1.6525

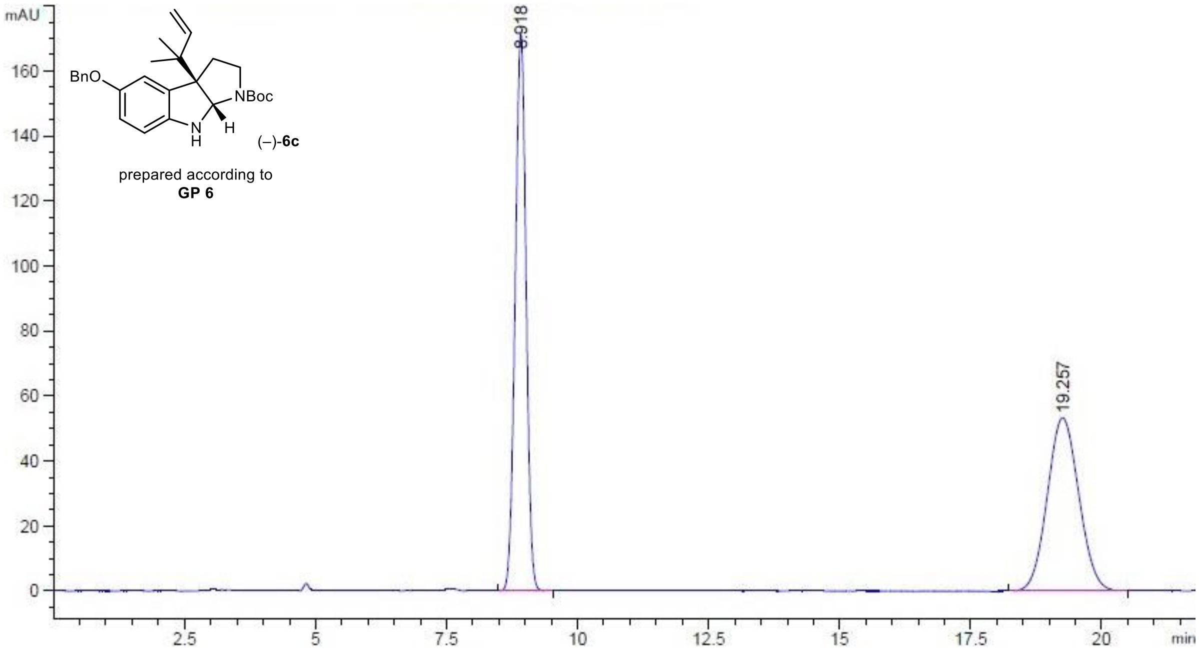

Peak #	RetTime [min]	Type	Width [min]	Area [mAU*s]	Height [mAU]	Area %
1	8.918	BB	0.2250	2475.84375	171.44070	52.6037
2	19.257	BB	0.5444	2230.75171	53.29073	47.3963

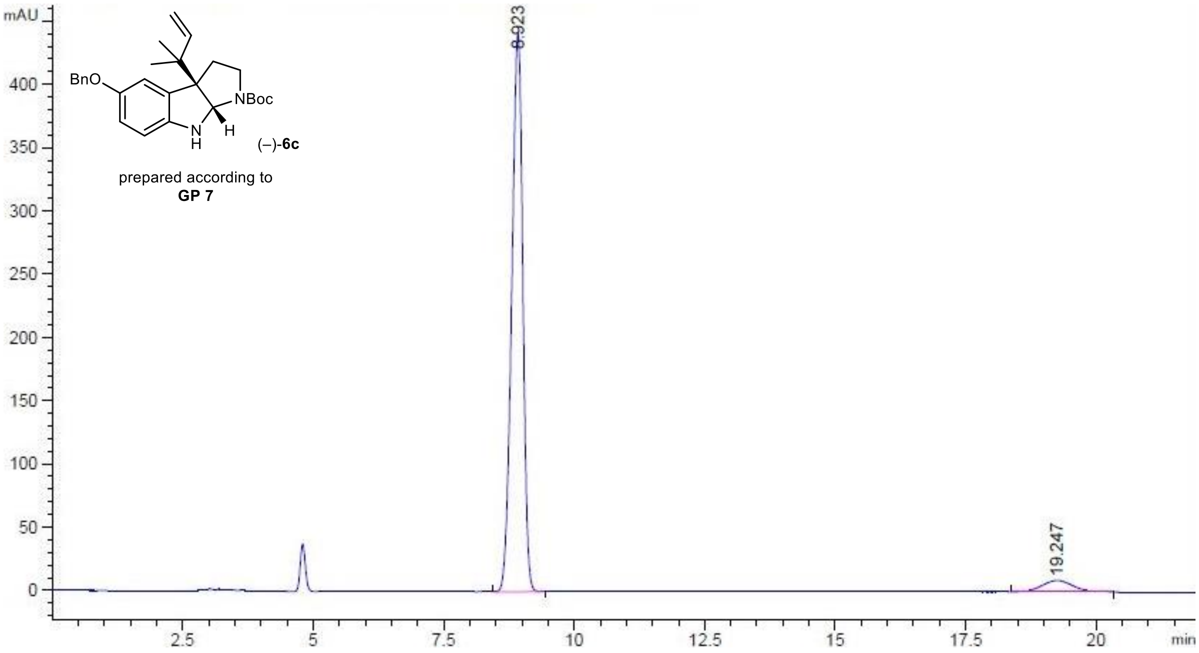

Peak #	RetTime [min]	Type	Width [min]	Area [mAU*s]	Height [mAU]	Area %
1	8.923	VB	0.2294	6484.39697	441.27197	94.5434
2	19.247	BB	0.4838	374.25009	9.05316	5.4566

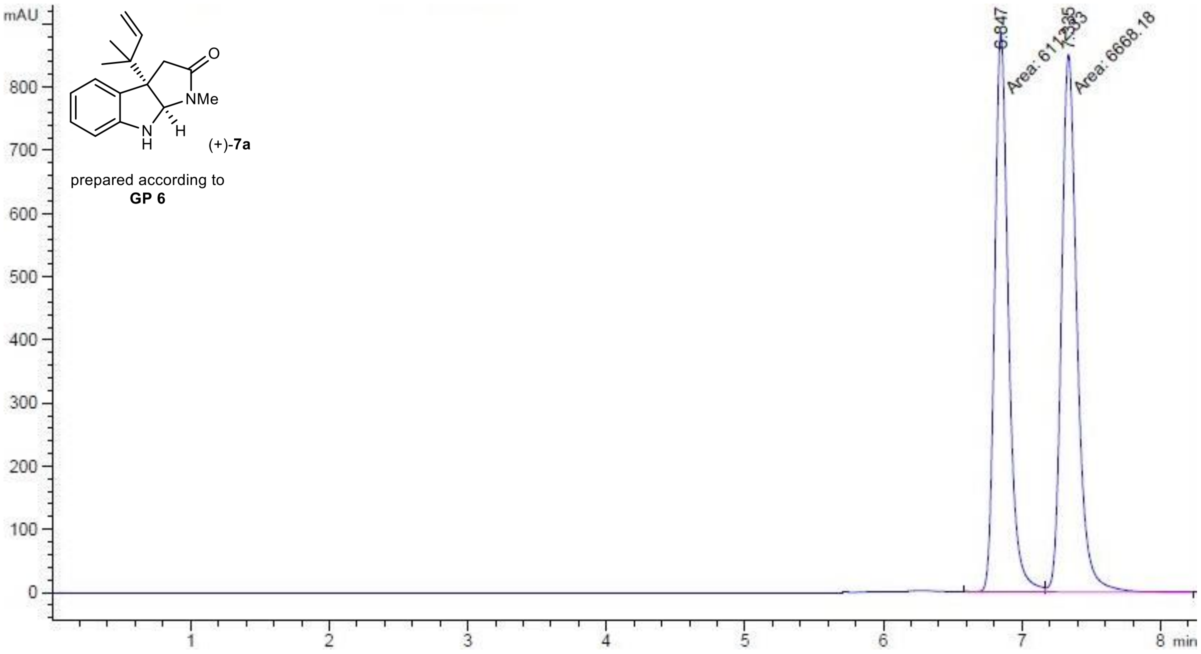

Peak #	RetTime [min]	Type	Width [min]	Area [mAU*s]	Height [mAU]	Area %
1	6.847	MF	0.1153	6112.32520	883.27747	47.8254
2	7.335	FM	0.1306	6668.17676	851.03613	52.1746

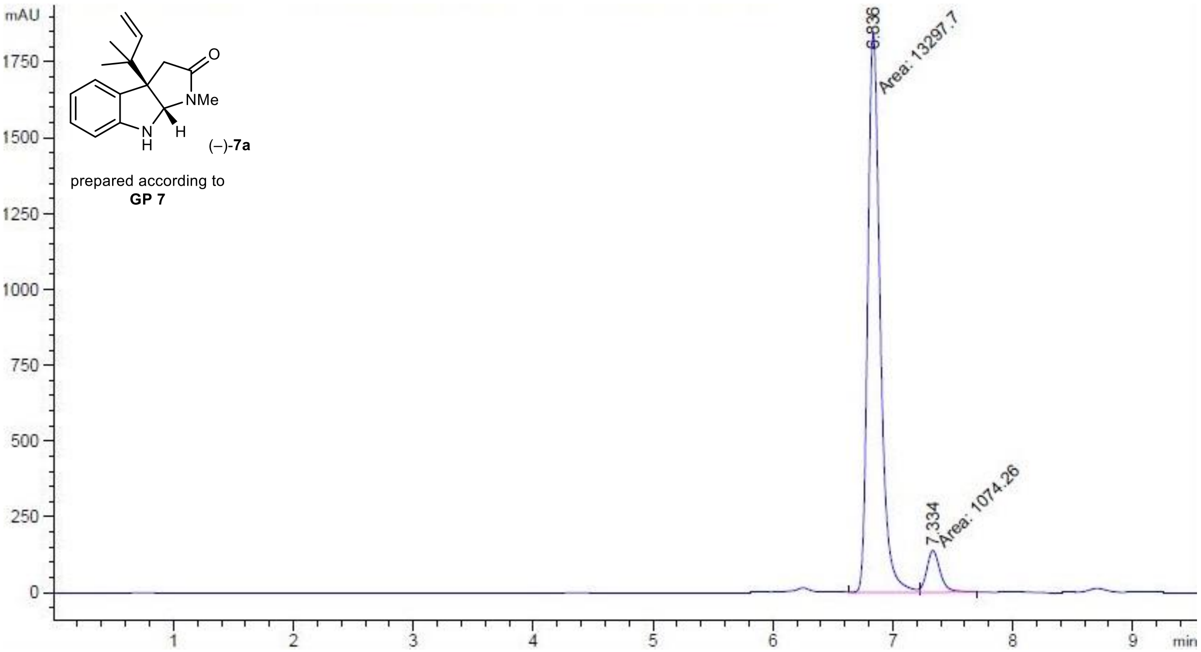

Peak #	RetTime [min]	Type	Width [min]	Area [mAU*s]	Height [mAU]	Area %
1	6.836	MF	0.1203	1.32977e4	1842.14954	92.5253
2	7.334	FM	0.1298	1074.26477	137.97604	7.4747

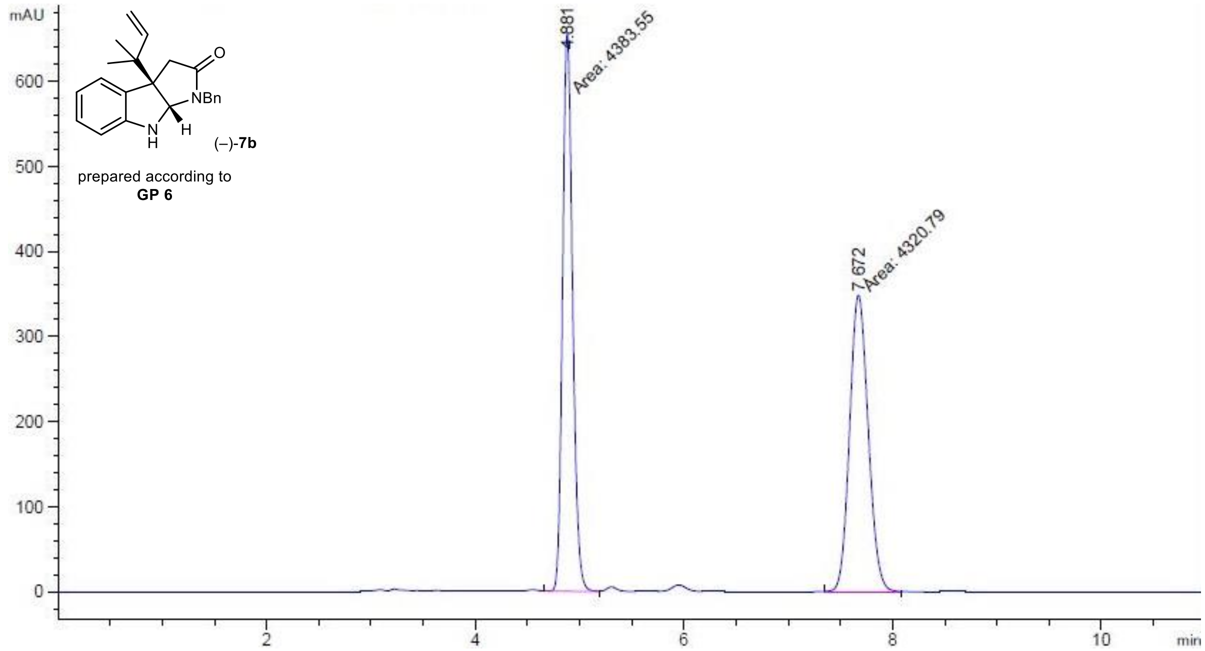

Peak #	RetTime [min]	Type	Width [min]	Area [mAU*s]	Height [mAU]	Area %
1	4.881	FM	0.1115	4383.55078	655.16620	50.3605
2	7.672	MF	0.2067	4320.79004	348.39203	49.6395

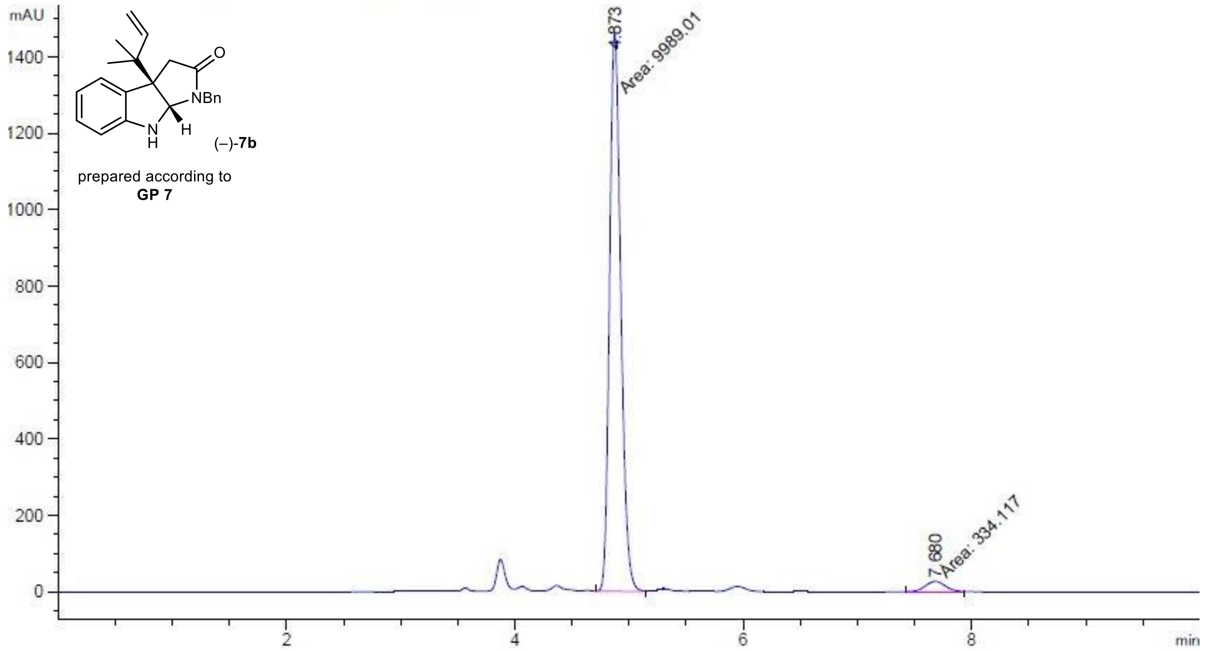

Peak #	RetTime [min]	Type	Width [min]	Area [mAU*s]	Height [mAU]	Area %
1	4.873	MF	0.1142	9989.00879	1458.45435	96.7634
2	7.680	MF	0.2055	334.11740	27.09901	3.2366

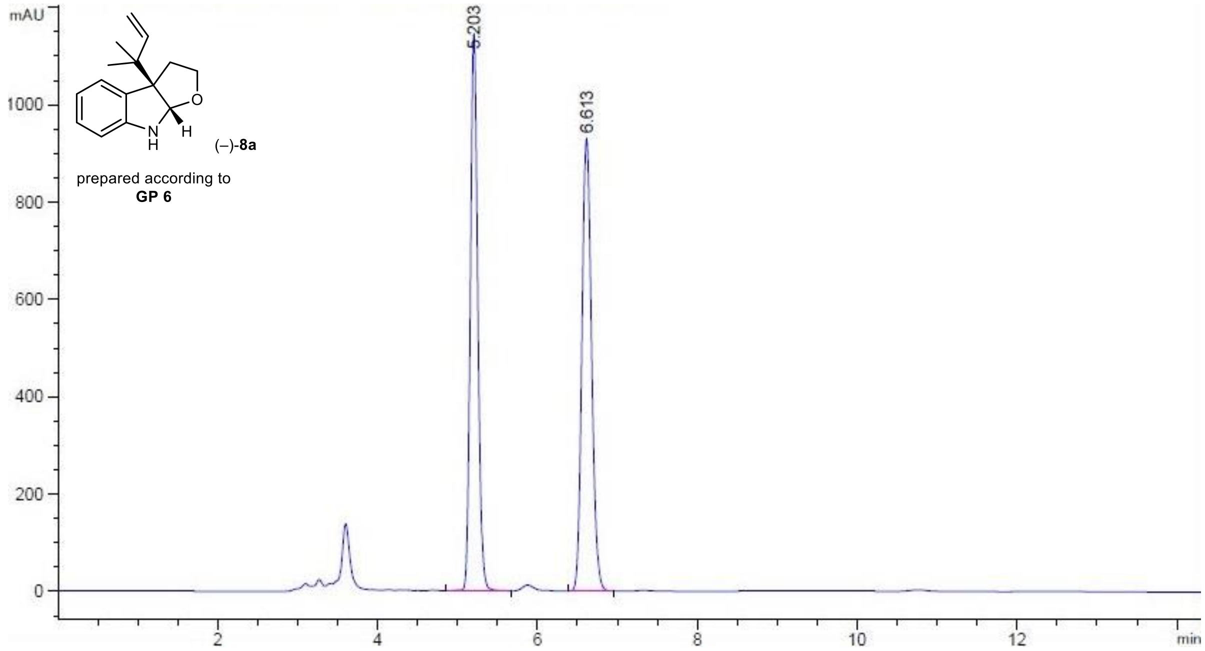

Peak #	RetTime [min]	Type	Width [min]	Area [mAU*s]	Height [mAU]	Area %
1	5.203	VV R	0.1006	7419.74414	1145.70825	48.8074
2	6.613	BB	0.1296	7782.34668	930.64459	51.1926

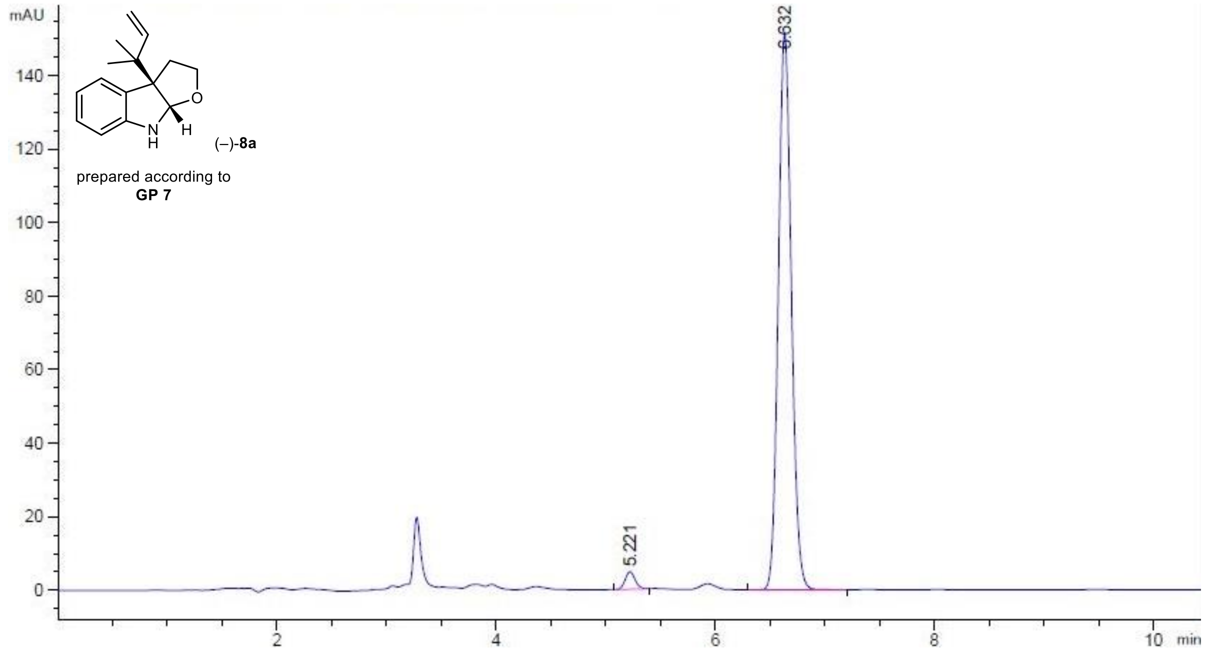

Peak #	RetTime [min]	Type	Width [min]	Area [mAU*s]	Height [mAU]	Area %
1	5.221	BB	0.0970	29.94556	4.78288	2.3387
2	6.632	BB	0.1284	1250.49133	151.35722	97.6613

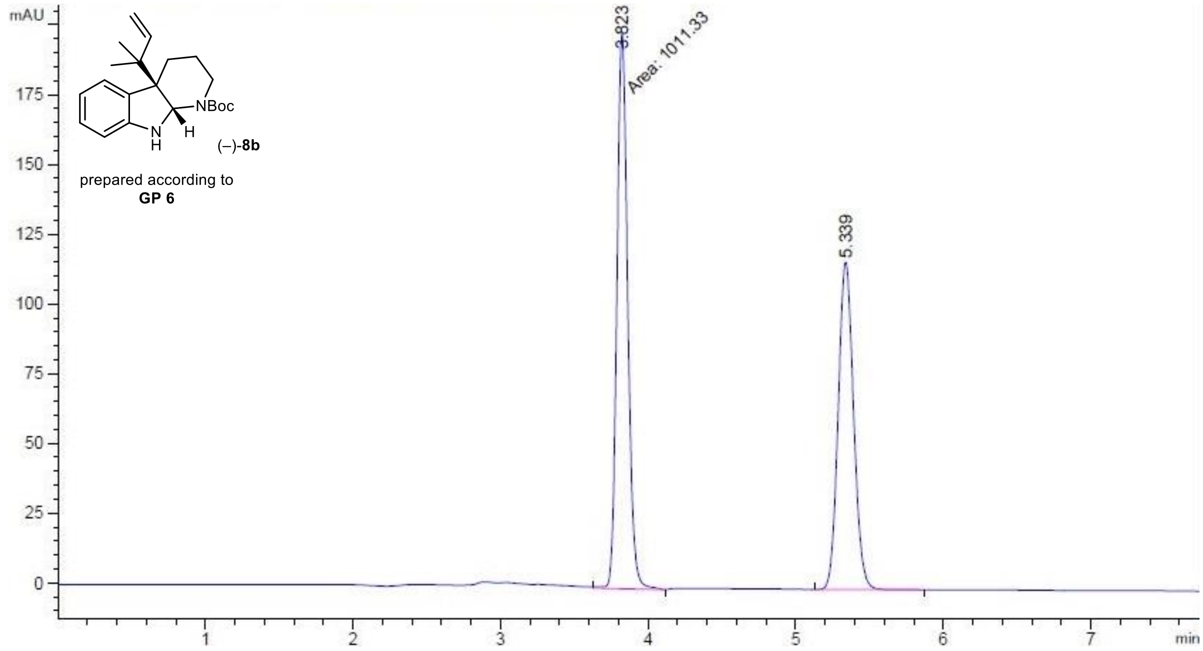

Peak #	RetTime [min]	Type	Width [min]	Area [mAU*s]	Height [mAU]	Area %
1	3.823	FM	0.0846	1011.32990	199.18814	54.2848
2	5.339	BB	0.1126	851.67603	117.39400	45.7152

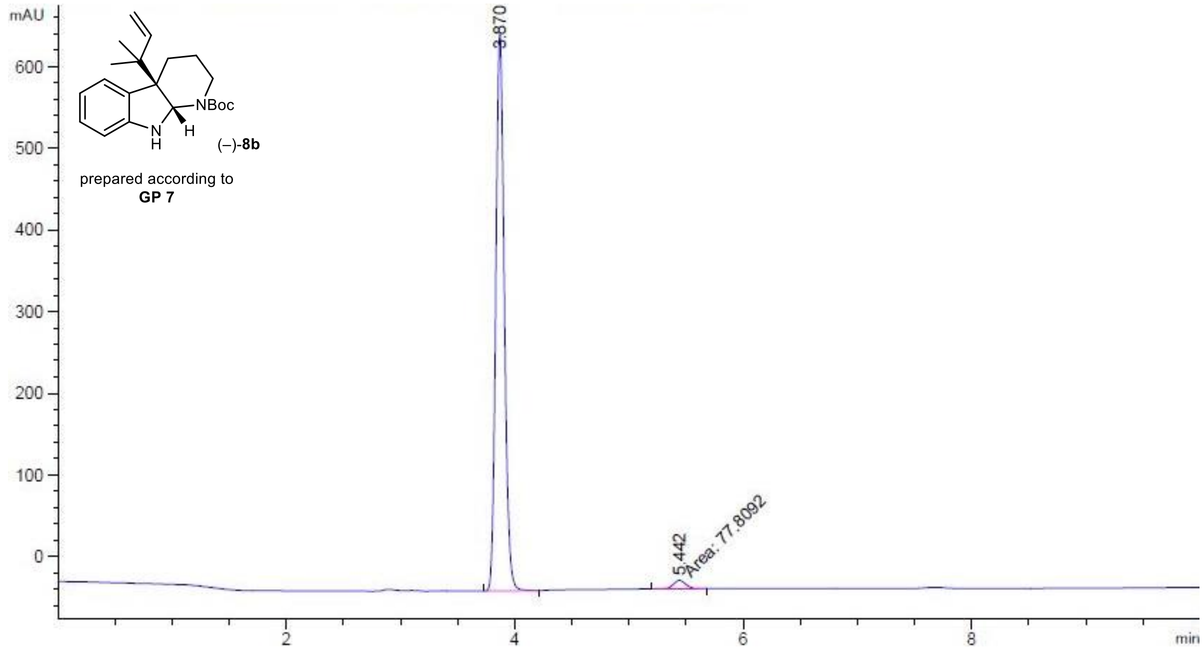

Peak #	RetTime [min]	Type	Width [min]	Area [mAU*s]	Height [mAU]	Area %
1	3.870	BB	0.0791	3518.38379	682.57666	97.8363
2	5.442	MM	0.1247	77.80916	10.40288	2.1637

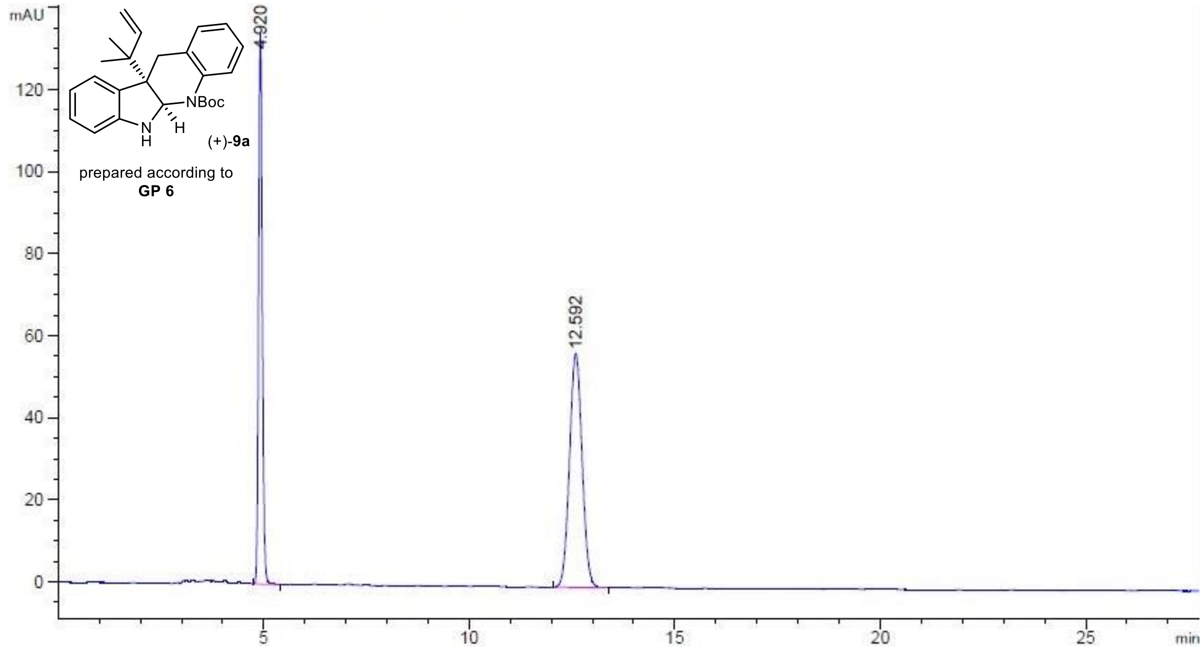

Peak #	RetTime [min]	Type	Width [min]	Area [mAU*s]	Height [mAU]	Area %
1	4.920	BB	0.0974	843.96246	134.21002	41.0409
2	12.592	BB	0.3238	1212.43225	56.97582	58.9591

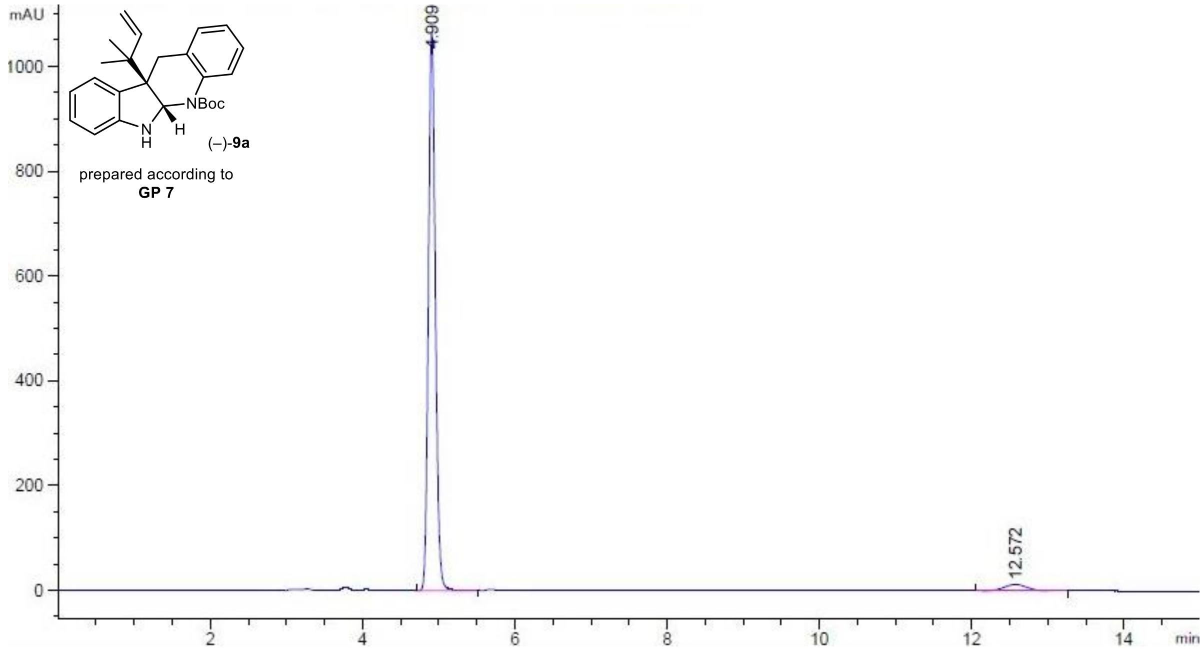

Peak #	RetTime [min]	Type	Width [min]	Area [mAU*s]	Height [mAU]	Area %
1	4.909	BB	0.0991	6795.86816	1063.16089	96.2251
2	12.572	BB	0.2750	266.60471	12.54209	3.7749

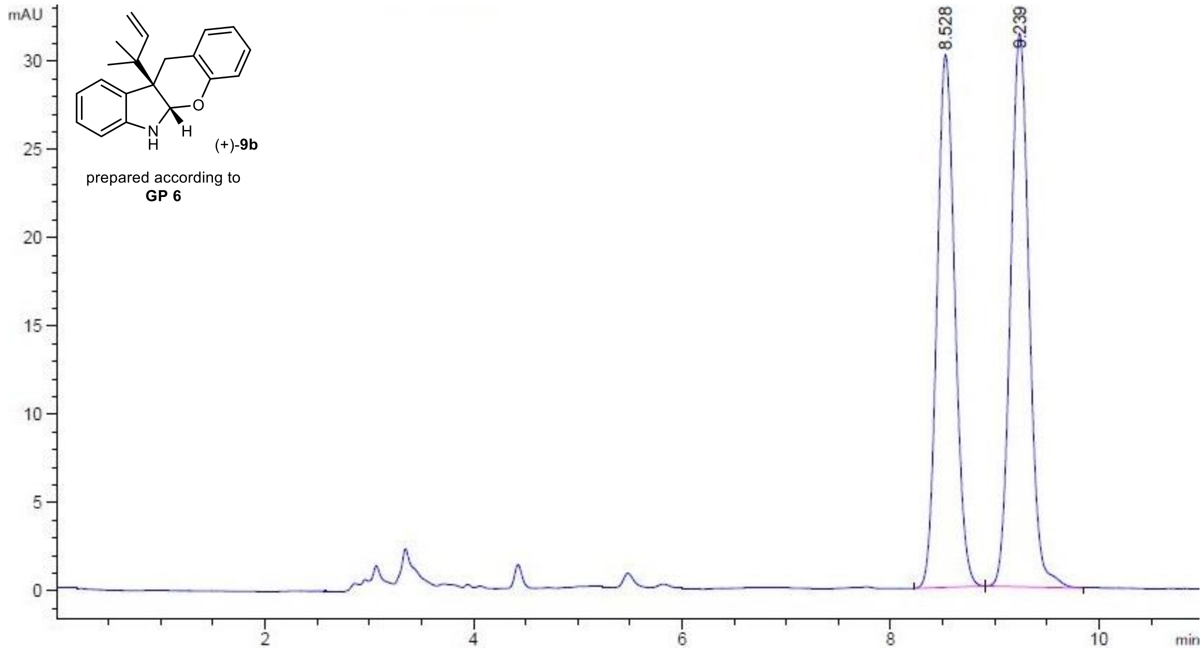

Peak #	RetTime [min]	Type	Width [min]	Area [mAU*s]	Height [mAU]	Area %
1	8.528	BB	0.1849	359.37067	30.18003	48.0888
2	9.239	BB	0.1909	387.93539	31.34585	51.9112

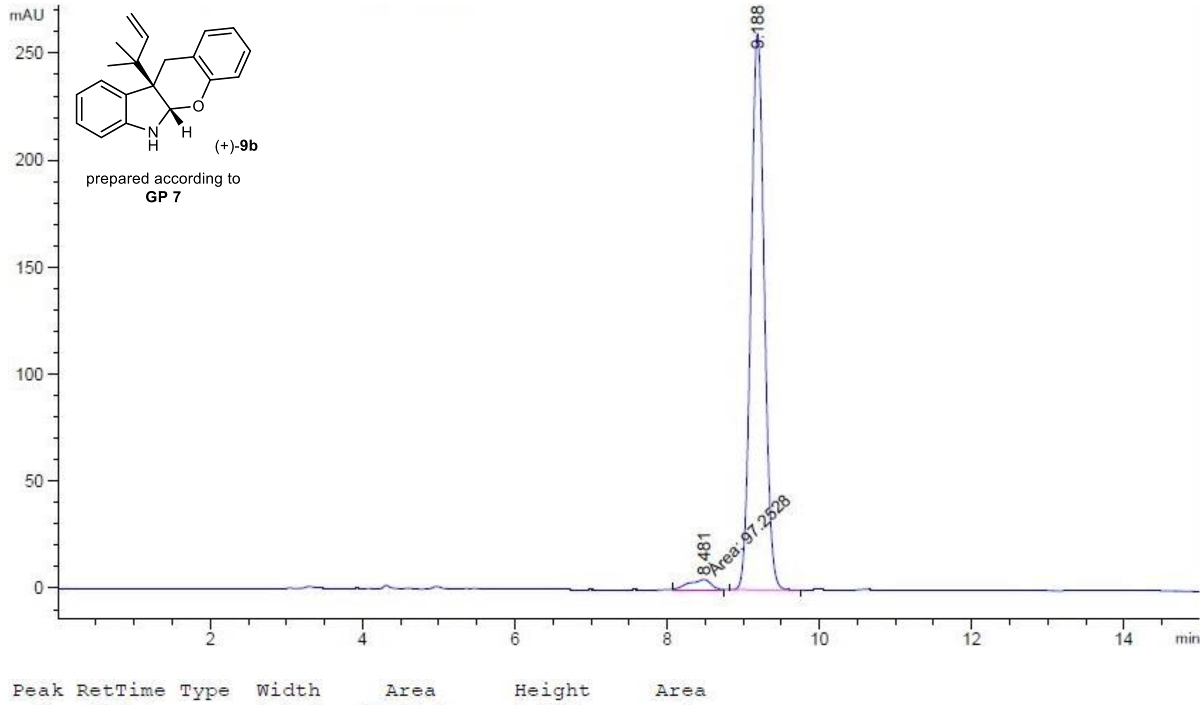

Peak #	RetTime [min]	Type	Width [min]	Area [mAU*s]	Height [mAU]	Area %
1	8.481	FM	0.3378	97.25278	4.79798	2.9670
2	9.188	BB	0.1902	3180.56860	259.98792	97.0330

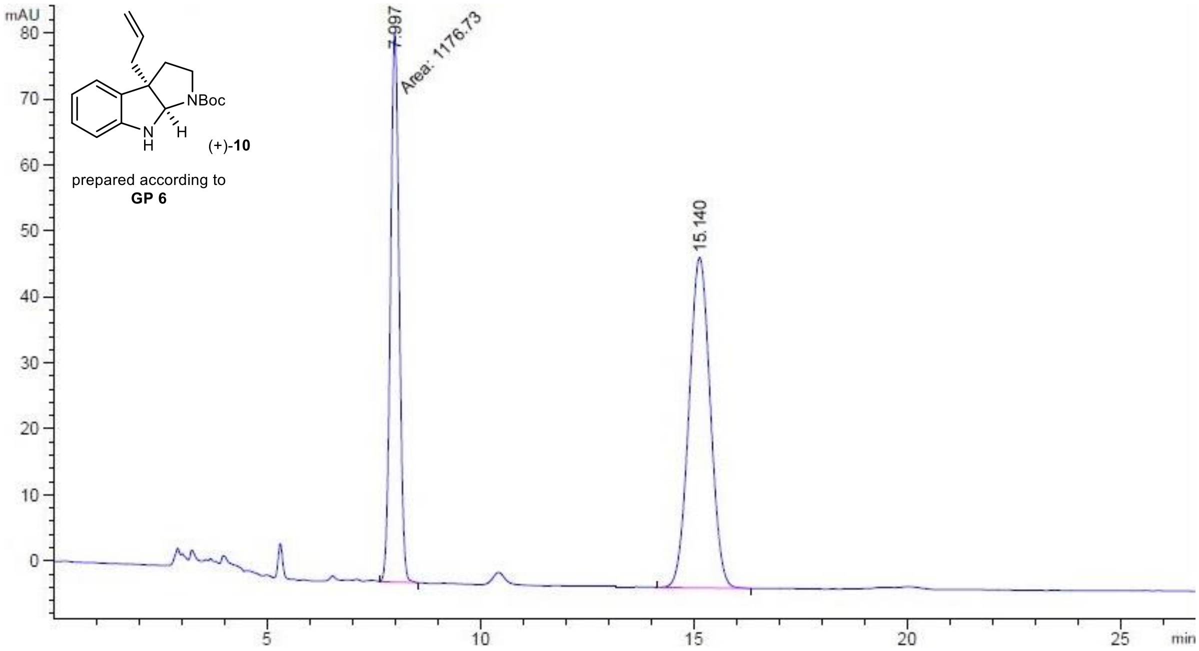

Peak #	RetTime [min]	Type	Width [min]	Area [mAU*s]	Height [mAU]	Area %
1	7.997	FM	0.2359	1176.73206	83.12414	40.4376
2	15.140	BB	0.5411	1733.25964	50.04599	59.5624

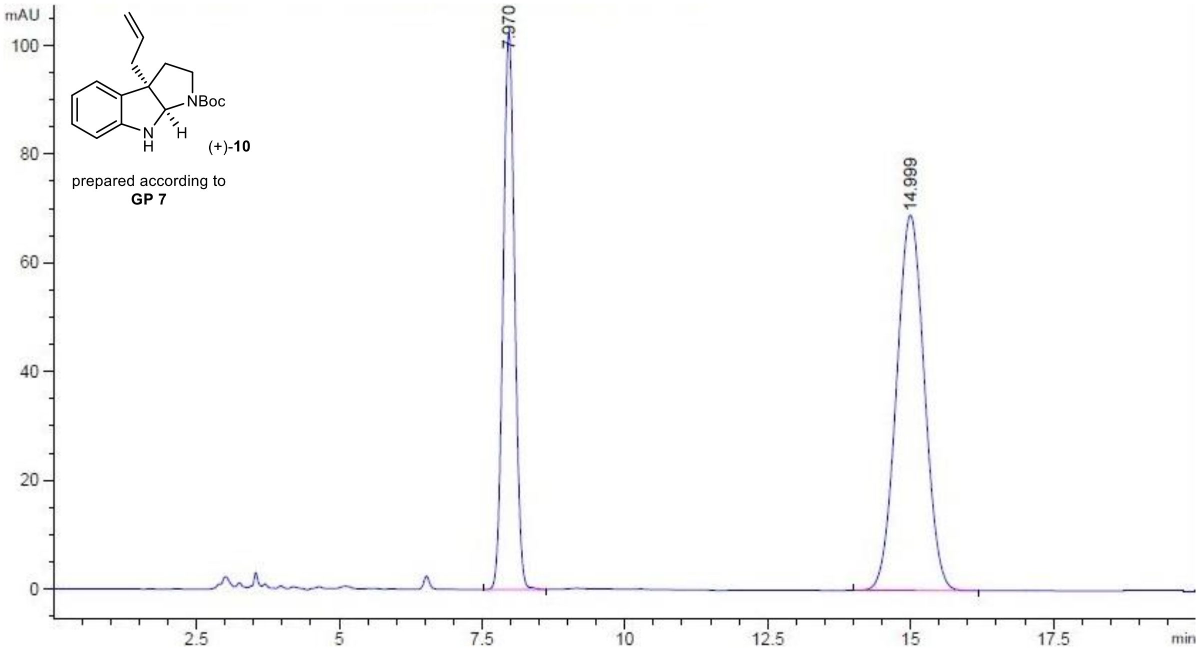

Peak #	RetTime [min]	Type	Width [min]	Area [mAU*s]	Height [mAU]	Area %
1	7.970	BB	0.2196	1435.02441	102.30805	38.0180
2	14.999	BB	0.5293	2339.56958	69.05221	61.9820

13.2. compound 11

Rationale for the determination of the enantiomeric ratios

Assuming that both diastereomers of **11** possess an identical UV-absorption, the assignment of the signals was made by correlating the HPLC-integrals with the diastereomeric ratios (*dr*) that were determined by ^{1}H-NMR spectroscopy according to equation (1a).

$$\frac{\text{integral}\ \#+\text{integral}\ \#}{\text{integral}\ \#+\text{integral}\ \#}=dr \qquad (1a)$$

Preparation of **11** according to **GP 6** resulted in a diastereomeric ratio of 2.8/1 (chromatogram A).

Preparation of **11** according to **GP 7** resulted in a diastereomeric ratio of 1/3.0 (chromatogram B).

Only the combination of peak 2/3 and peak 1/4 reflects the diastereomeric ratios that were determined by ^{1}H-NMR spectroscopy in both cases:

$$\frac{\text{integral}\ 2+\text{integral}\ 3}{\text{integral}\ 1+\text{integral}\ 4}=\frac{2.5}{1} \qquad \text{(chromatogram A)}$$

$$\frac{\text{integral}\ 2+\text{integral}\ 3}{\text{integral}\ 1+\text{integral}\ 4}=\frac{1}{3} \qquad \text{(chromatogram B)}$$

Hence, peaks 2/3 and peaks 1/4 originate from enantiomers.

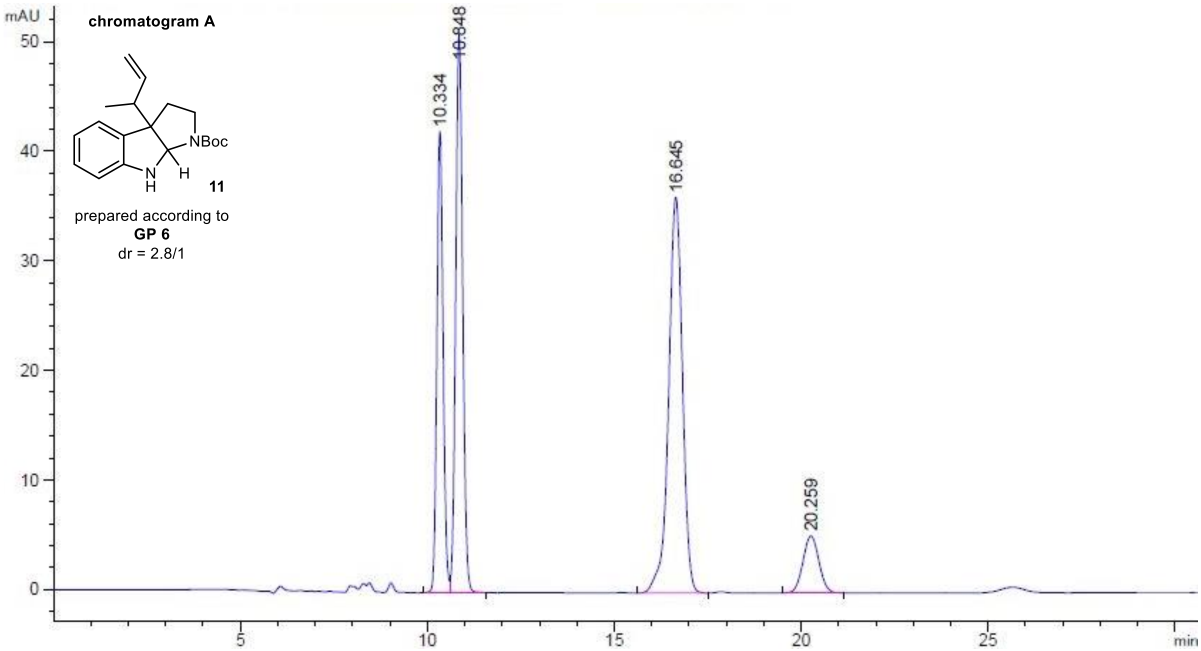

Peak #	RetTime [min]	Type	Width [min]	Area [mAU*s]	Height [mAU]	Area %
1	10.334	BV	0.1793	488.52792	42.11224	21.8628
2	10.848	VB	0.2021	664.33820	50.95086	29.7308
3	16.645	BB	0.3946	926.98285	36.09759	41.4848
4	20.259	BB	0.4622	154.66435	5.21176	6.9216

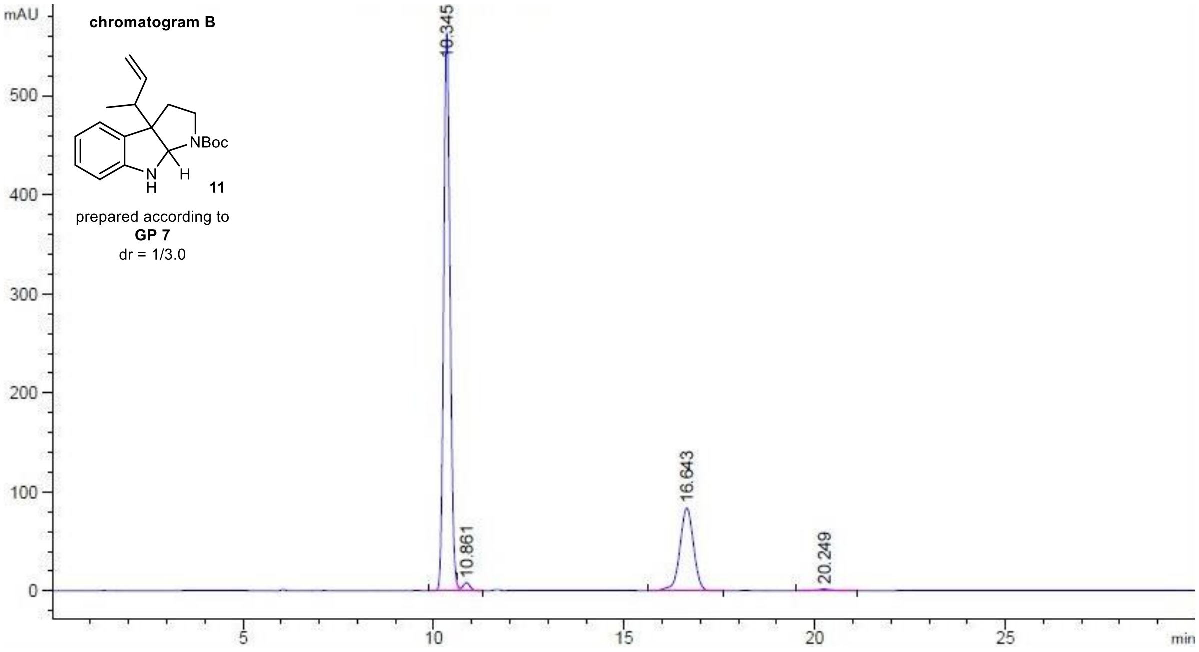

Peak #	RetTime [min]	Type	Width [min]	Area [mAU*s]	Height [mAU]	Area %
1	10.345	BV R	0.1811	6576.70264	563.70734	74.3655
2	10.861	VB E	0.2031	109.72273	8.30561	1.2407
3	16.643	BB	0.3879	2103.30225	83.78140	23.7829
4	20.249	BB	0.4483	54.03247	1.82555	0.6110

13.3. compound 12

Rationale for the determination of the enantiomeric ratios

Comound **12** was prepared according to **GP 7** with (*rac*)-**L1** with a diastereomeric ratio (diastereomer 1/diastereomer 2) = 1/2.7. Its HPLC-chromatogram A shows two separated signals (peak 2 and 3) whose ratio of integrals equals 1/2.7. Hence, these signals belong to distinct diastereomers and their UV-absorption is identical within the precision of measurements. Peak 1 contains two overlapping signals.

Comound **12** was prepared according to **GP 7** with (*R*)-**L1** with a diastereomeric ratio (diastereomer 1/diastereomer 2) = 1/1.46 (chromatogram B). The enantiomeric ratio for each diastereomer can be calculated after extracting the individual integrals that are contained within peak 1. For this purpose, the integrals are denoted with A, B, C and D as depicted in the chromatograms, leading to equation (1b).

$$\frac{\text{integral A} + \text{integral D}}{\text{integral B} + \text{integral C}} = \frac{1}{1.46} \qquad (1b)$$

Solvation of equation (1b) with $\text{integral A} + \text{integral B} = \text{X}$ leads to the individual integrals:

$$\text{integral A} = \frac{\text{X}}{2.46} + \frac{\text{integral C}}{2.46} - \frac{1.46 \text{ integral D}}{2.46} = 37.2352 \text{ Area\%}$$

$$\text{integral B} = \frac{1.46 \text{ X}}{2.46} - \frac{\text{integral C}}{2.46} + \frac{1.46 \text{ integral D}}{2.46} = 59.2853 \text{ Area\%}$$

Hence, the enantiomeric ratios are determined as:

er = 11/1 (diastereomer 1)

er = 922/1 (diastereomer 2)

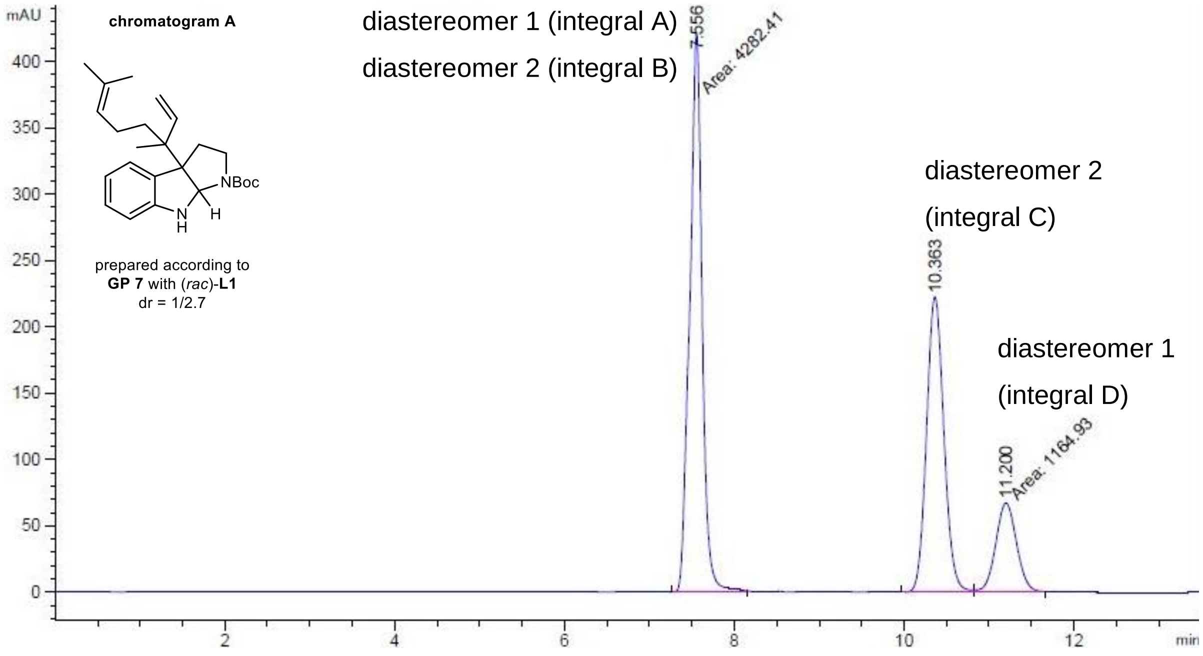

Peak #	RetTime [min]	Type	Width [min]	Area [mAU*s]	Height [mAU]	Area %
1	7.556	MF	0.1697	4282.41064	420.69257	49.6771
2	10.363	BV	0.2198	3173.14868	222.58778	36.8094
3	11.200	MF	0.2882	1164.92773	67.37809	13.5135

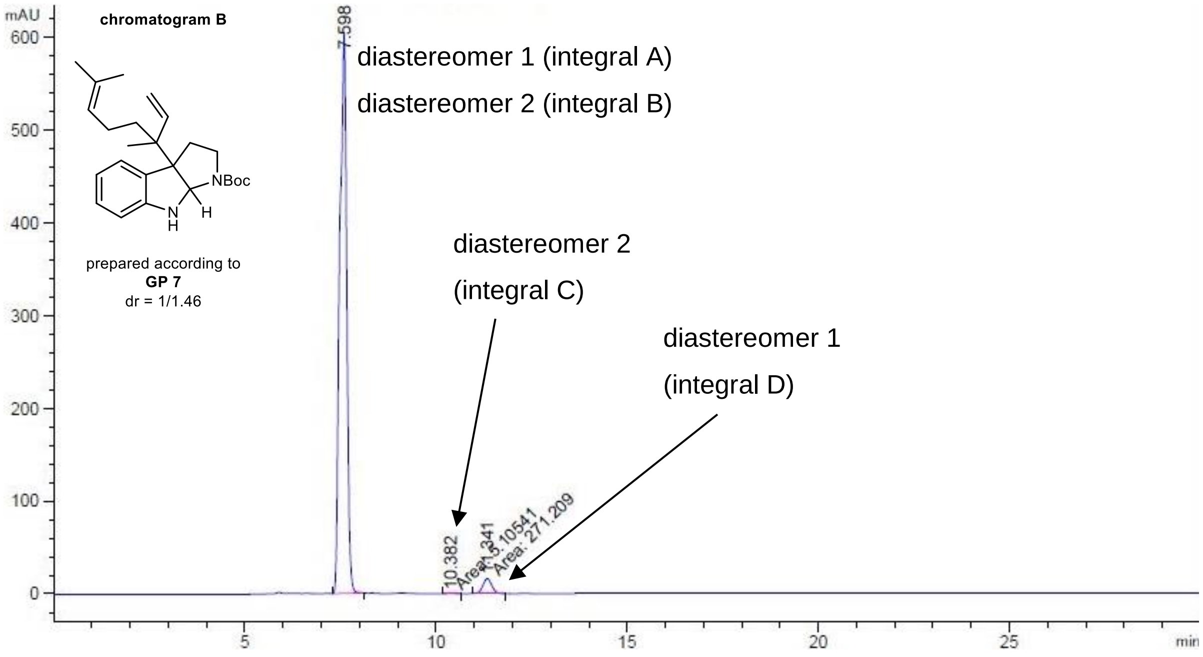

Peak #	RetTime [min]	Type	Width [min]	Area [mAU*s]	Height [mAU]	Area %
1	7.598	BB	0.1761	7664.95947	603.69458	96.5205
2	10.382	MM	0.2002	5.10541	4.25105e-1	0.0643
3	11.341	MF	0.2761	271.20874	16.37250	3.4152

13.4. compound 13

Rationale for the determination of the enantiomeric ratios

Assuming that both diastereomers of **13** possess an identical UV-absorption, the assignment of the signals was made by correlating the HPLC-integrals with the diastereomeric ratios (*dr*) that were determined by ^{1}H-NMR spectroscopy according to the equation (1a).

$$\frac{\text{integral \# + integral \#}}{\text{integral \# + integral \#}} = dr \qquad (1a)$$

Preparation of **13** according to **GP 7** (*rac*-**1e,** (*R*)-**L1**) resulted in a diastereomeric ratio of 1/1.1 (chromatogram A). It is concluded that the two major signals (peak 1 and 4) belong to diastereomers as their ratio of integrals equals 1/1.1.

Preparation of **13** under conditions that will be disclosed in a prospective report resulted in a diastereomeric ratio of 15/1 (chromatogram B). Only the combination of peak 2/4 and peak 1/3 reflects the diastereomeric ratio that was determined by ^{1}H-NMR spectroscopy:

$$\frac{\text{integral 2 + integral 4}}{\text{integral 1 + integral 3}} = \frac{13}{1} \qquad \text{(chromatogram B)}$$

Hence, peaks 2/4 and peaks 1/3 originate from enantiomers.

- Chromatogram C shows the analysis of **13** prepared according to **GP 7** with (*R*)-**1e** and (*R*)-**L1**. The minor enantiomer of either diastereomer could not be detected.

- Chromatogram D shows the analysis of **13** prepared according to **GP 7** with (*S*)-**1e** and (*R*)-**L1**.

- The correct identification of the four signals was validated by the preparation of **13** according to **GP 7** with (*S*)-**1e** and (*S*)-**L1** (chromatogram E). The minor enantiomer of either diastereomer could not be detected.

- Chromatogram F shows the analysis of **13** prepared according to **GP 8** with (*R*)-**1e** (5 equiv.) and **K-2b** (5 mol%). (no additional Bu_4NCl)

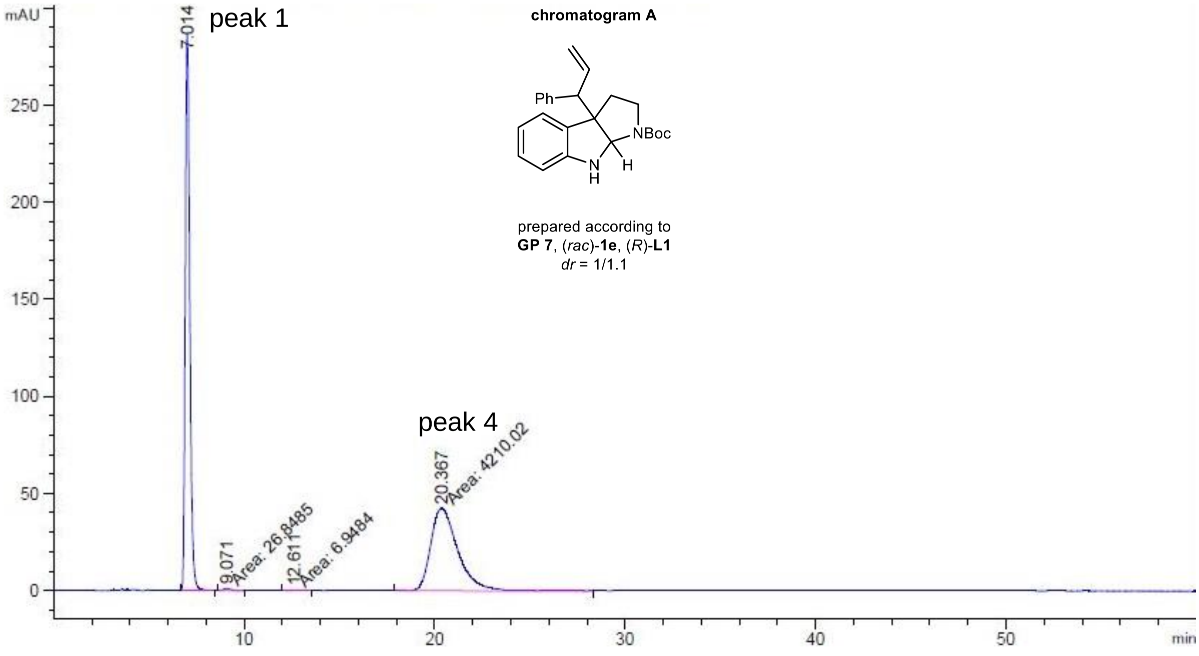

Peak #	RetTime [min]	Type	Width [min]	Area [mAU*s]	Height [mAU]	Area %
1	7.014	BB	0.2523	4727.21777	286.86401	52.6943
2	9.071	MM	0.4342	26.84849	1.03069	0.2993
3	12.611	MM	0.5644	6.94840	2.05201e-1	0.0775
4	20.367	MM	1.6502	4210.01514	42.51931	46.9290

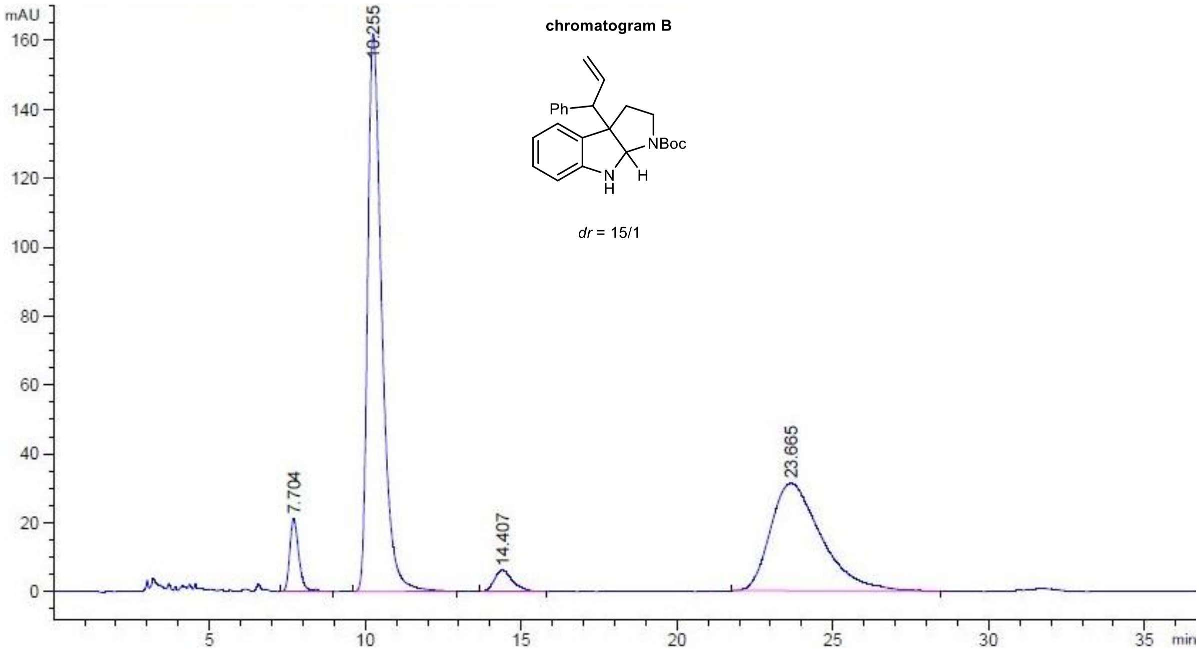

Peak #	RetTime [min]	Type	Width [min]	Area [mAU*s]	Height [mAU]	Area %
1	7.704	BV R	0.3059	424.95297	21.05819	4.5491
2	10.255	BB	0.4791	5124.97852	161.98309	54.8627
3	14.407	BB	0.5015	251.49713	6.11874	2.6923
4	23.665	BB	1.3262	3540.03906	31.23519	37.8960

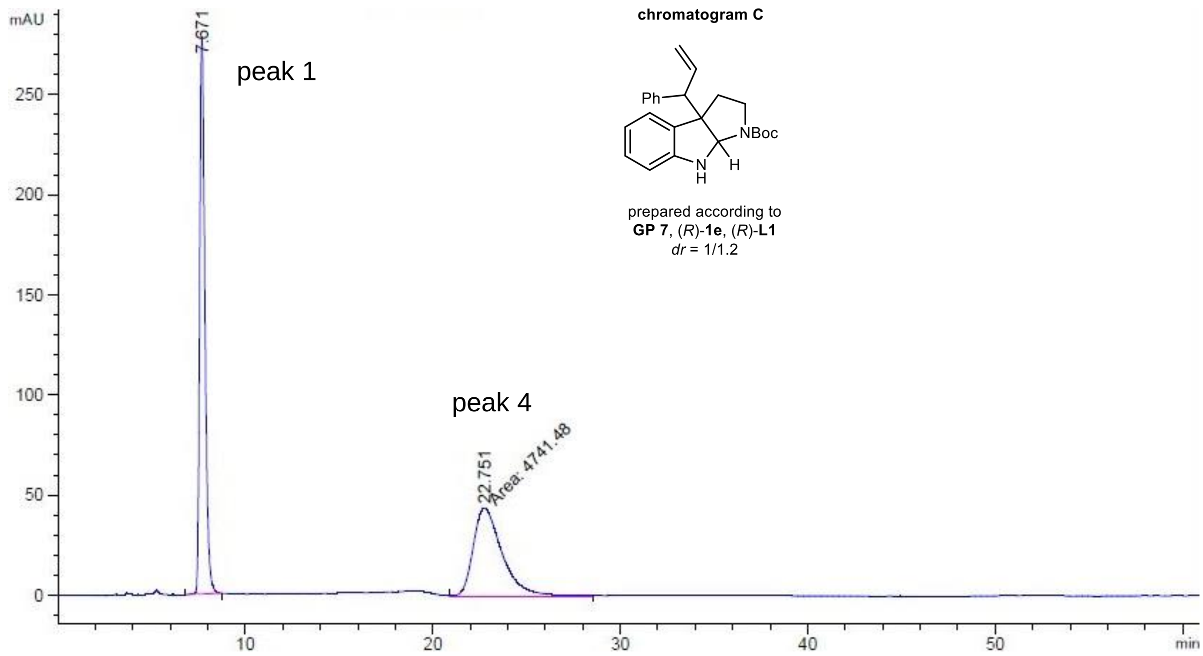

Peak #	RetTime [min]	Type	Width [min]	Area [mAU*s]	Height [mAU]	Area %
1	7.671	BB	0.3002	5442.82031	277.64468	53.4433
2	22.751	MM	1.7953	4741.47656	44.01693	46.5567

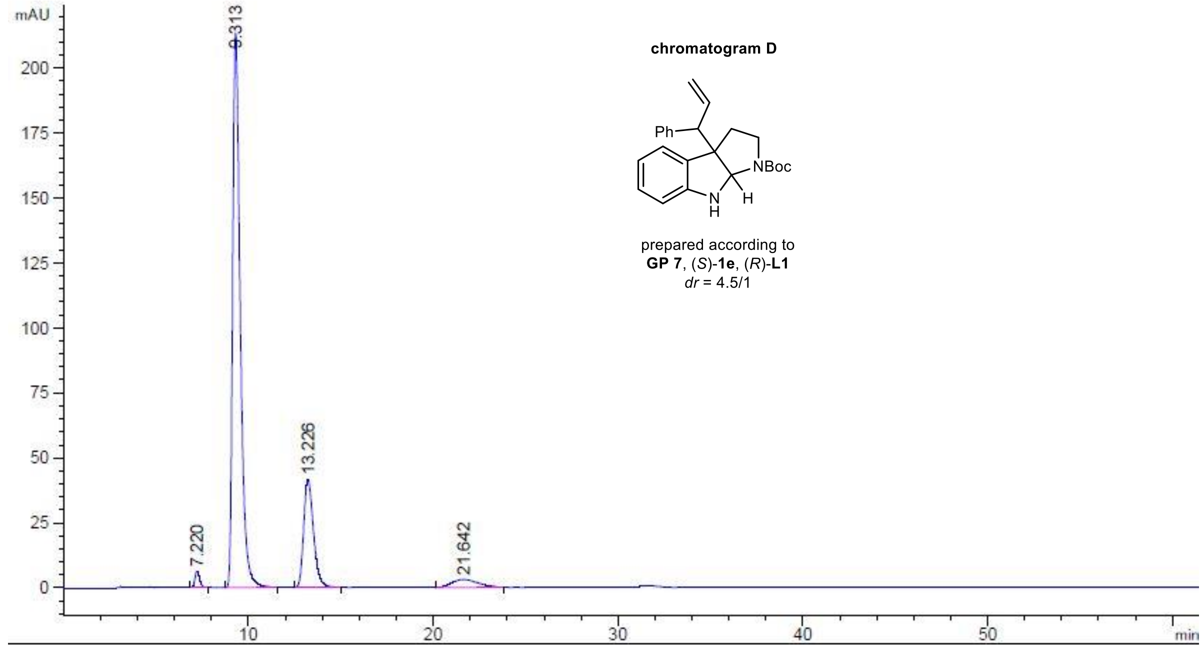

Peak #	RetTime [min]	Type	Width [min]	Area [mAU*s]	Height [mAU]	Area %
1	7.220	BB	0.2247	109.37569	6.23316	1.3673
2	9.313	BB	0.4297	6059.44678	213.43787	75.7483
3	13.226	BB	0.5060	1532.98218	41.59082	19.1636
4	21.642	BB	1.1428	297.64963	3.04265	3.7209

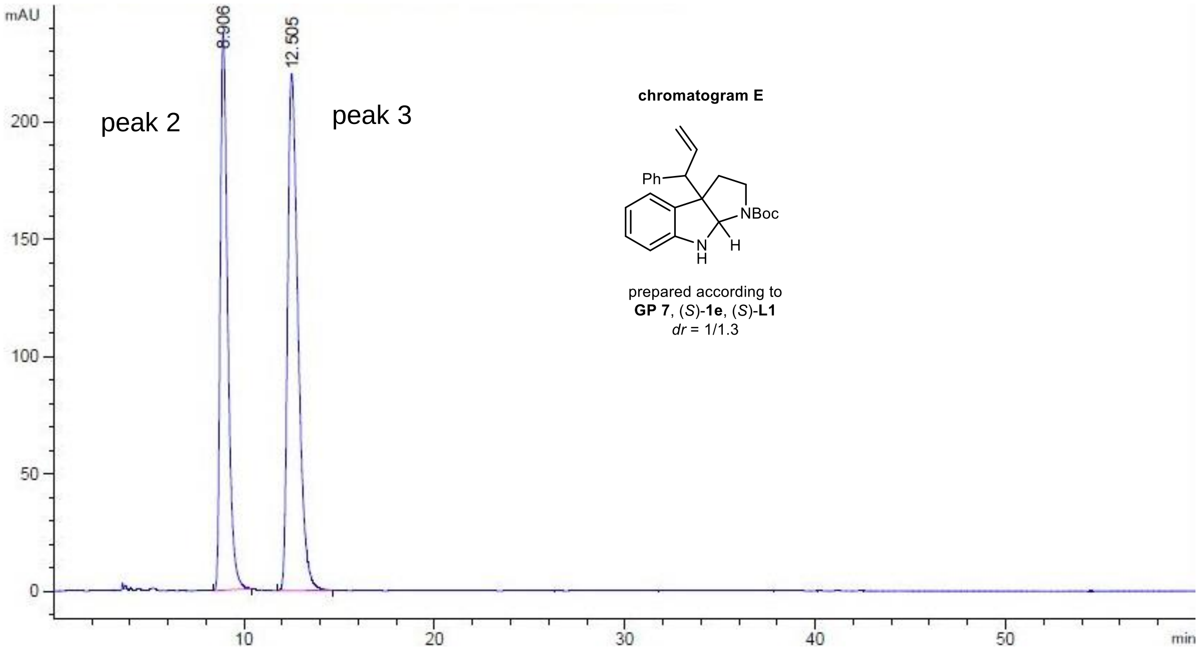

Peak #	RetTime [min]	Type	Width [min]	Area [mAU*s]	Height [mAU]	Area %
1	8.906	BB	0.4096	6466.10938	237.15575	43.4973
2	12.505	BB	0.5679	8399.42480	220.01448	56.5027

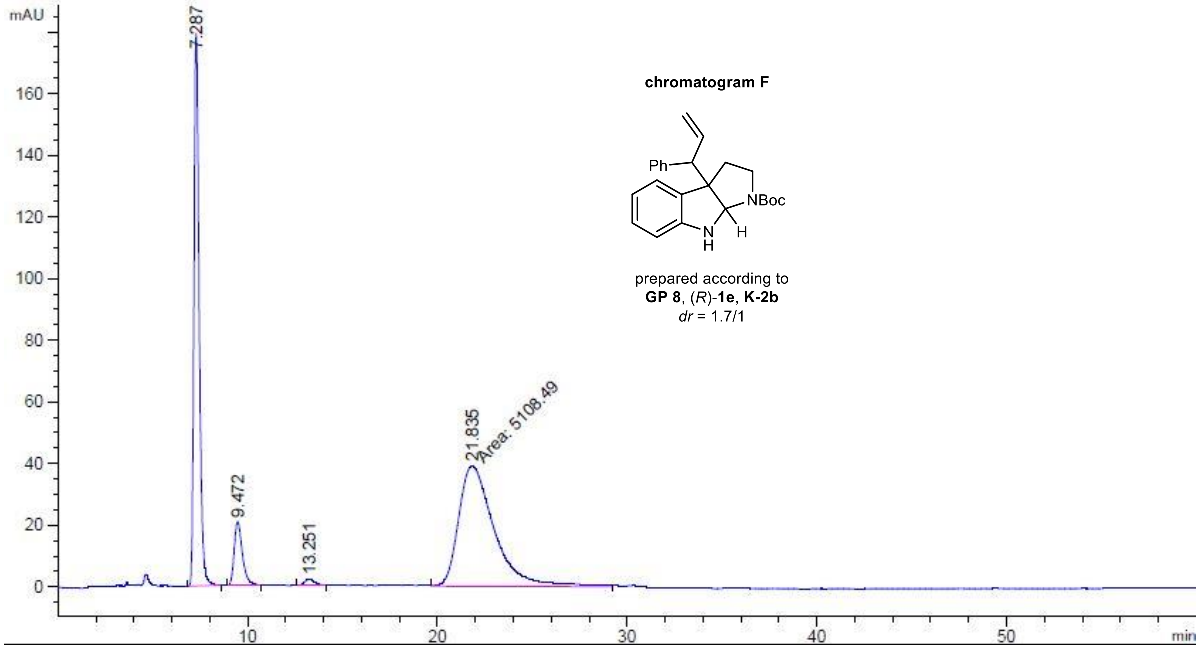

Peak #	RetTime [min]	Type	Width [min]	Area [mAU*s]	Height [mAU]	Area %
1	7.287	BB	0.2927	3420.40918	179.18848	37.1152
2	9.472	BB	0.4027	610.53955	20.48788	6.6250
3	13.251	BB	0.4560	76.22370	1.96535	0.8271
4	21.835	MM	2.1772	5108.49268	39.10661	55.4327

14. References

[1] J. E. Beaver, B. C. Peacor, J. V. Bain, L. I. James, M. L. Waters, *Org. Biomol. Chem.* **2015**, *13*, 3220.

[2] M. Agnes, A. Sorrenti, D. Pasini, K. Wurst, D. B. Amabilino, *CrystEngComm* **2014**, *16*, 10131.

[3] A. Urbano, S. Vallejo, M. J. Cabrera-Afonso, E. Yonte, *Org. Lett.* **2020**, *22*, 6122.

[4] G. Kumaraswamy, M. Sastry, N. Jena, K. Kumar, M. Vairamani, *Tetrahedron: Asymmetry* **2003**, *14*, 3797.

[5] C. Defieber, M. A. Ariger, P. Moriel, E. M. Carreira, *Angew. Chem. Int. Ed.* **2007**, *46*, 3139.

[6] T. J. Hoffman, E. M. Carreira, *Angew. Chem. Int. Ed.* **2011**, *50*, 10670.

[7] T. M. Beck, B. Breit, *Angew. Chem. Int. Ed.* **2017**, *56*, 1903.

[8] D. Albat, A. Köcher, J. Witt, H.-G. Schmalz, *Eur. J. Org. Chem.* **2022**, *2022*.

[9] W.-B. Liu, C. Zheng, C.-X. Zhuo, L.-X. Dai, S.-L. You, *J. Am. Chem. Soc.* **2012**, *134*, 4812.

[10] R. Matunas, A. J. Lai, C. Lee, *Tetrahedron* **2005**, *61*, 6298.

[11] J. M. Kraus, H. C. Gits, R. B. Silverman, *Tetrahedron Lett.* **2012**, *53*, 1319.

[12] B. W. H. Turnbull, J. Chae, S. Oliver, P. A. Evans, *Chem. Sci.* **2017**, *8*, 4001.

[13] B. M. Trost, S. Malhotra, W. H. Chan, *J. Am. Chem. Soc.* **2011**, *133*, 7328.

[14] J. Stambaský, A. V. Malkov, P. Kocovský, *J. Org. Chem.* **2008**, *73*, 9148.

[15] G. Y. Fang, O. A. Wallner, N. Di Blasio, X. Ginesta, J. N. Harvey, V. K. Aggarwal, *J. Am. Chem. Soc.* **2007**, *129*, 14632.

[16] R. J. Mayer, N. Hampel, A. R. Ofial, *Chem. - Eur. J.* **2021**, *27*, 4070.

[17] J. Graff, J. Müller, A. Sadurní, M. Rubin, I. A. Canivete Cuissa, C. Keller, M. Hartmann, S. Singer, J. Gertsch, K.-H. Altmann, *ChemMedChem* **2022**, *17*, e202200308.

[18] M. D. Lebar, B. J. Baker, *Aust. J. Chem.* **2010**, *63*, 862.

[19] S. Roy, S. Haque, G. Gribble, *Synthesis* **2006**, *2006*, 3948.

[20] L. Verotta, F. Orsini, M. Sbacchi, M. A. Scheildler, T. A. Amador, E. Elisabetsky, *Bioorg. Med. Chem.* **2002**, *10*, 2133.

[21] V. A. Ignatenko, P. Zhang, R. Viswanathan, *Tetrahedron Lett.* **2011**, *52*, 1269.

[22] C. Aubry, A. J. Wilson, P. R. Jenkins, S. Mahale, B. Chaudhuri, J.-D. Maréchal, M. J. Sutcliffe, *Org. Biomol. Chem.* **2006**, *4*, 787.

[23] E. C. Gentry, L. J. Rono, M. E. Hale, R. Matsuura, R. R. Knowles, *J. Am. Chem. Soc.* **2018**, *140*, 3394.

[24] Y. Kita, J. Haruta, H. Yasuda, K. Fukunaga, Y. Shirouchi, Y. Tamura, *J. Org. Chem.* **1982**, *47*, 2697.

[25] Y. Yang, X. Jiang, F.-L. Qing, *J. Org. Chem.* **2012**, *77*, 7538.

[26] J. Teichert, M. Fañanás-Mastral, B. Feringa, *Synthesis* **2012**, *44*, 409.

[27] D. C. Schuck, A. K. Jordão, M. Nakabashi, A. C. Cunha, V. F. Ferreira, C. R. S. Garcia, *Eur. J. Med. Chem.* **2014**, *78*, 375.

[28] T. Lebleu, H. Kotsuki, J. Maddaluno, J. Legros, *Tetrahedron Lett.* **2014**, *55*, 362.

[29] M. Fantacuzzi, B. de Filippis, M. Gallorini, A. Ammazzalorso, L. Giampietro, C. Maccallini, Z. Aturki, E. Donati, R. S. Ibrahim, E. Shawky, A. Cataldi, R. Amoroso, *Eur. J. Med. Chem.* **2020**, *185*, 111815.

[30] M. Yamaguchi, R. Hagiwara, H. Muto, K. Gayama, H. Konishi, K. Manabe, *Org. Lett.* **2023**, *25*, 4913.

[31] J.-K. Dai, W.-J. Dan, N. Li, H.-T. Du, J.-W. Zhang, J.-R. Wang, *Bioorg. Med. Chem. Lett.* **2016**, *26*, 580.

[32] D. B. C. Martin, C. D. Vanderwal, *J. Am. Chem. Soc.* **2009**, *131*, 3472.

[33] J. Wolfard, J. Xu, H. Zhang, C. K. Chung, *Org. Lett.* **2018**, *20*, 5431.

[34] R. Delgado, S. B. Blakey, *Eur. J. Org. Chem.* **2009**, *2009*, 1506.

[35] J. A. Rossi-Ashton, A. K. Clarke, R. J. K. Taylor, W. P. Unsworth, *Org. Lett.* **2020**, *22*, 1175.

[36] R. W. Schumacher, B. S. Davidson, *Tetrahedron* **1999**, *55*, 935.

[37] B. E. A. Burm, M. M. Meijler, J. Korver, M. J. Wanner, G.-J. Koomen, *Tetrahedron* **1998**, *54*, 6135.

[38] R. H. Pouwer, S. M. Deydier, P. van Le, B. D. Schwartz, N. C. Franken, R. A. Davis, M. J. Coster, S. A. Charman, M. D. Edstein, T. S. Skinner-Adams, K. T. Andrews, I. D. Jenkins, R. J. Quinn, *ACS Med. Chem. Lett.* **2014**, *5*, 178.

[39] M. Li, Y. Xu, M. Zuo, W. Liu, L. Wang, W. Zhu, *J. Nat. Prod.* **2019**, *82*, 2279.

[40] E. I. Silva-Lopez, A. O. Barden, J. A. Brozik, *Bioorg. Med. Chem. Lett.* **2013**, *23*, 773.

[41] N. Stuhr-Hansen, J. Andersen, M. B. Thygesen, K. Strømgaard, *ChemistrySelect* **2016**, *1*, 407.

[42] W. Cao, Y. Dou, C. Kouklovsky, G. Vincent, *Angew. Chem. Int. Ed.* **2022**, *61*, e202209135.

[43] S. Zhu, D. W. C. MacMillan, *J. Am. Chem. Soc.* **2012**, *134*, 10815.

[44] C. C. Tjin, R. F. Wissner, H. Jamali, A. Schepartz, J. A. Ellman, *ACS Med. Chem. Lett.* **2018**, *9*, 1013.

[45] N. Kumar, A. Maity, V. R. Gavit, A. Bisai, *Chem. Commun.* **2018**, *54*, 9083.

[46] M.-R. Ryan, D. Lynch, S. G. Collins, A. R. Maguire, *Org. Process Res. Dev.* **2024**, *28*, 1946.

[47] S. Takiguchi, T. Iizuka, Y. Kumakura, K. Murasaki, N. Ban, K. Higuchi, T. Kawasaki, *J. Org. Chem.* **2010**, *75*, 1126.

[48] T. Hino, K. HASUMI, H. YAMAGUCHI, M. TANIGUCHI, M. Nakagawa, *Chem. Pharm. Bull.* **1985**, *33*, 5202.

[49] M. S. Morales-Ríos, O. R. Suárez-Castillo, P. Joseph-Nathan, *Tetrahedron* **2002**, *58*, 1479.

[50] T. Ideguchi, T. Yamada, T. Shirahata, T. Hirose, A. Sugawara, Y. Kobayashi, S. Ōmura, T. Sunazuka, *J. Am. Chem. Soc.* **2013**, *135*, 12568.

[51] J. Ruchti, E. M. Carreira, *J. Am. Chem. Soc.* **2014**, *136*, 16756.

[52] B. M. Trost, J. Quancard, *J. Am. Chem. Soc.* **2006**, *128*, 6314.

[53] X. Zhang, W.-B. Liu, H.-F. Tu, S.-L. You, *Chem. Sci.* **2015**, *6*, 4525.

[54] L. Peters, G. M. König, H. Terlau, A. D. Wright, *J. Nat. Prod.* **2002**, *65*, 1633.

[55] M. S. Morales-Ríos, N. F. Santos-Sánchez, O. R. Suárez-Castillo, P. Joseph-Nathan, *Magnetic Reson in Chemistry* **2002**, *40*, 677.
